# The dual role of thiourea in the thiotrifluoromethylation of alkenes†
†Electronic supplementary information (ESI) available. CCDC 1474239–1474241. For ESI and crystallographic data in CIF or other electronic format see DOI: 10.1039/c6sc02790c



**DOI:** 10.1039/c6sc02790c

**Published:** 2016-09-30

**Authors:** Paolo Ricci, Tanatorn Khotavivattana, Lukas Pfeifer, Maurice Médebielle, John Richard Morphy, Véronique Gouverneur

**Affiliations:** a Chemistry Research Laboratory , Department of Chemistry , Oxford University , OX1 3TA , UK . Email: veronique.gouverneur@chem.ox.ac.uk ; Fax: +44 (0)1865 285002; b Université de Lyon , Université Claude Bernard Lyon I , ICBMS UMR CNRS – UCBL – CPE – INSA 5246 , Equipe Synthèse de Molécules d'Intérêt Thérapeutique (SMITh) , 43 bd du 11 Novembre 1918 , Villeurbanne 69622 , France; c Medicinal Chemistry , Eli Lilly and Company Limited , Erl Wood Manor, Sunninghill Road , Windlesham , Surrey GU20 6PH , UK

## Abstract

Alkenes substituted with a thiourea undergo C–CF_3_ followed by intramolecular C–S bond formation with the Togni reagent and trifluoroacetic acid (TFA) at room temperature; thiols and thioamides are not suitable S-sources for this reaction. This anti-addition process involves a CF_3_ radical, and affords CF_3_-substituted thiazolines and thiazines for medicinal applications. A metal or photoredox catalyst is not required as the thiourea acts as a reductant, as well as serving as an S-source capable of adding to a C-centered radical. Mechanistic work comparing the reactivity of thiourea, urea, thioamide and thiol in the context of alkene trifluoromethylation demonstrates that in this series, the thiourea is unique for its ability to release CF_3_ radical from the Togni reagent, and to orchestrate trifluoromethylation followed by *S*-cyclization with both activated and unactivated alkenes.

## Abstract 2

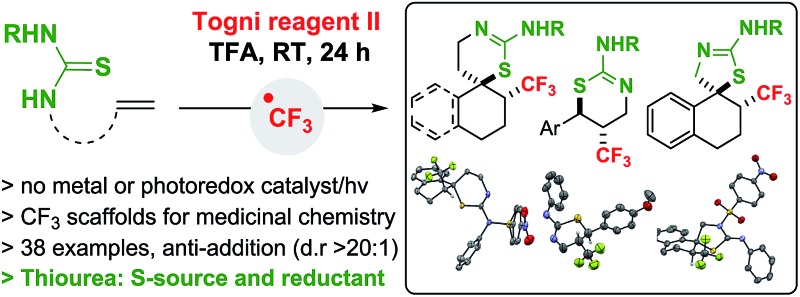
We report the stereoselective and metal-free trifluoromethylation of alkenes followed by *S*-cyclization using thiourea as the S-source and SET initiator.

## Introduction

**Scheme 1:**
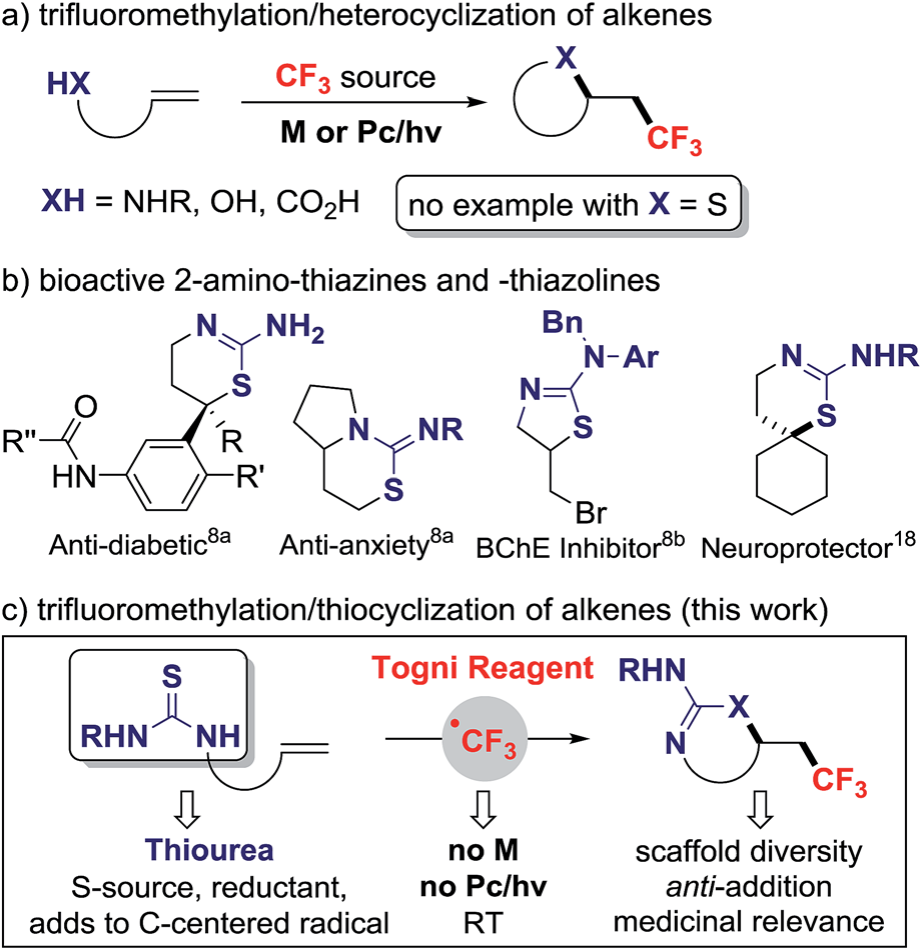
Trifluoromethylation/thiocyclization of alkenes (M = metal, Pc = photoredox catalyst).

A large number of pharmaceuticals contain a trifluoromethyl group because this structural motif affects the properties of organic molecules.^1^ The installation of trifluoromethyl groups onto sp^3^ hybridized carbon has progressed significantly with numerous addition reactions of CF_3_ across alkenes. Alkene vicinal functionalizations featuring C–CF_3_ combined with C–H, C–C or C–heteroatom bond formation have been disclosed, most requiring a transition metal or photoredox catalyst to activate the CF_3_ reagent (Scheme 1a).^2^ Vicinal difunctionalizations involving sulfur heteroatom are notoriously rare; this process is much more challenging as, in contrast to amines and alcohols, thiols undergo facile *S*-trifluoromethylation with the Togni or Umemoto reagents in the absence of catalyst.^3^ A case of alkene thiotrifluoromethylation was reported by Langlois in 2000.^4^ In this process, photolysis of CF_3_SO_2_SPh generates a CF_3_ radical (CF_3_˙) that adds to the alkene; this step affords a weakly nucleophilic radical that reacts with CF_3_SO_2_SPh to provide the thioether product and the chain propagating trifluoromethylsulfonyl radical. The reagent in this reaction serves both as CF_3_ and S-source, thereby minimizing S–CF_3_ bond formation. In a related approach, Zard reported the net addition of *S*-trifluoromethyl xanthates reagents onto alkenes, a process initiated with lauroyl peroxide.^5^ The abundance of sulfur containing heterocycles in medicinal chemistry^6^ prompted us to study alkene difunctionalization *via* C–CF_3_ and C–S bond formation where the CF_3_ and SR groups would not stem from a single reagent. In 2015, Liu and co-workers reported a case of intermolecular difunctionalization with the copper-catalyzed trifluoromethylthiocyanation of alkenes; this process requires trimethylsilylisocyanate, a silicon-based S-source that acts as Lewis acid to activate the Togni reagent.^7^

In our design plan, we opted to examine the reactivity of olefins with pending thioureas, a decision driven by synthetic and mechanistic considerations. Trifluoromethylation followed by C–S bond formation would afford novel trifluoromethylated 2-amino-thiazolines and 2-amino-thiazines for applications in medicinal chemistry.^8^ Selected 2-amino-thiazines and -thiazolines are important scaffolds in the development of aspartate beta-secretase enzyme (BACE-1) inhibitors, a therapeutic target for Alzheimer's disease,^9^ and are common motifs in several bioactive compounds (Scheme 1b). Mechanistically, the ability of thioureas to act as reducing agent^10^ and radical scavenger^11^ suggests that this group may induce the release of CF_3_˙ from the Togni reagent,^12^ and serve as an S-source capable of adding on a C-centered radical. Here we report that thiourea-substituted alkenes undergo C–CF_3_ followed by C–S bond formation with the Togni reagent and TFA. This operationally simple reaction does not require a metal catalyst, and affords diverse CF_3_-substituted 2-amino-thiazolines and thiazines resulting from overall anti-addition across the C=C π bond (Scheme 1c).

## Results and discussion

To identify suitable reaction conditions, we selected the unactivated alkene **1aa**, and the Togni **I**, **II** ^13^ and Umemoto **III** ^14^ reagents as CF_3_ source (Table 1).^15^ The desired 2-amino-thiazoline **2aa** resulting from trifluoromethylation followed by *S*-cyclization was formed in low yield when the reaction was carried out at room temperature in CHCl_3_ with **I**, **II** or **III** in the absence of catalyst or additive (Table 1, entries 1–3). No side-products resulting from oxidative dimerization or S–CF_3_ bond formation were detected. The conversion of **1aa** into **2aa** decreased at 60 °C (Table 1, entries 2 and 3).

**Table 1:** Optimization of reaction parameters

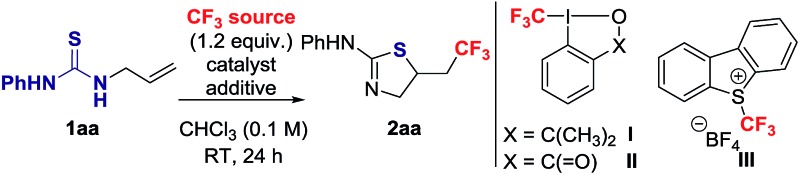				
Entry	CF_3_ source	Catalyst	Additive	Yield^*a*^ (%)
1	**I**	—	—	7
2	**II**	—	—	33, 21^*b*^
3	**III**	—	—	12, 7^*b*^
**4**	**II**	**—**	**TFA (2 equiv.)**	**76, 62** ^*c*^
5	**II**	—	TFA (1 equiv.)	69
6	**III**	—	TFA (2 equiv.)	13
7^*d*^	**II**	—	TFA (2 equiv.)	59
8	**II**	**A** (5 mol%)	—	21
9	**II**	**A** (100 mol%)	**B** (1 equiv.)	33
10^*e*^	**II**	**C** (5 mol%)	—	20
11^*e*^	**II**	**D** (2 mol%)	—	31
12^*e*^	**III**	**C** (5 mol%)	—	40
13^*e*^	**III**	**D** (2 mol%)	—	38

^*a*^Determined by ^19^F NMR integration relative to an internal standard (C_6_H_5_CF_3_).

^*b*^Reaction at 60 °C.

^*c*^Reaction time is 1 h.

^*d*^Reaction in CH_3_CN.

^*e*^14 W bulb as light source (*λ*_max_ = 452 nm). A = Cu(CH_3_CN)_4_PF_6_. B = 1,10-phenantroline. C = Ru(bpy)_3_(PF_6_)_2_. D = methylene blue. TFA = trifluoroacetic acid.

Activation of the Togni reagents by protonation with BrØnsted acid is well documented,^16^ but not typically considered for CF_3_ addition onto alkenes. We envisioned that upon protonation of **II** with trifluoroacetic acid (TFA), the resulting highly electrophilic iodine centre could undergo S–I(iii) coordination with the thiourea functionality followed by single electron transfer (SET) with more effective release of CF_3_ radical. Gratifyingly, 62% of **2aa** was observed after 1 h when the reaction was conducted in the presence of 2 equiv. of TFA, and the yield reached 76% after 24 h (Table 1, entry 4). The reaction was less effective using 1 equiv. of TFA (Table 1, entry 5). The presence of the acid did not induce protocyclization, and its benefit was not significant with Umemoto **III** (Table 1, entry 6).

**Scheme 2:**
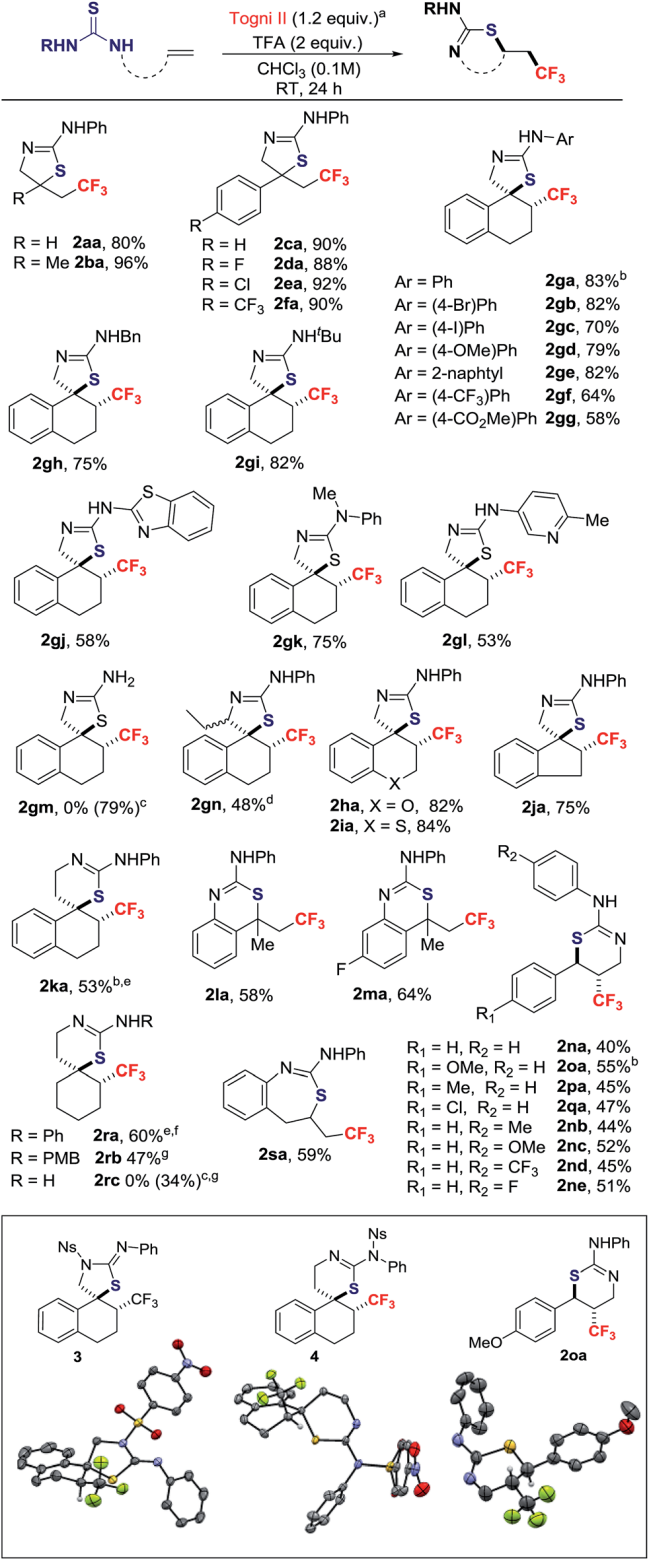
Substrate scope of the reaction. ^a^The reaction was performed on a 0.3 mmol scale; yield of isolated product; d.r. > 20 : 1 by ^19^F NMR of crude reaction. ^b^Relative configuration established by single crystal X-ray diffraction analysis; for **2ga** and **2ka**, analysis was performed on the derivatives **3** and **4**, respectively. ^c^ **2gm** and **2rc** were obtained by *in situ* deprotection of **2gi** and **2rb**, respectively; yields from the alkene. ^d^d.r. = 3.5 : 1. ^e^d.r. = 6 : 1. ^f^61% yield when the reaction was scaled up to 2.3 mmol. ^g^d.r. = 5 : 1. PMB = *para*-methoxybenzyl.

With the conditions described in entry 4 of Table 1, the scope of the thiotrifluoromethylation was investigated (Scheme 2). Allyl and metallyl thioureas afforded 2-amino-thiazolines **2aa** and **2ba** in 80% and 96%, respectively. A range of *para*-substituted styrenes underwent thiotrifluoromethylation with yields up to 92%. The reaction was extended to 1,2-dihydro-naphthalenes, 2*H*-chromene, 2*H-*thiochromene and indene; in this series, all thiazolines were formed as a single stereoisomer resulting from anti-addition (d.r. > 20 : 1).^17^ The 1,2-dihydro-naphthalene scaffold was selected to investigate the tolerance of the reaction to variation of the thiourea *N-*substituent. The resulting products anti-**2ga–2gl** were isolated in yields ranging from 53% to 83%. No reaction occurred with **1gm**, a substrate possessing the free NH_2_ sub-motif. The corresponding 2-amino-thiazoline **2gm** was obtained by a detour pathway involving *in situ* deprotection of the *N-t*Bu group of **2gi** under acidic conditions. The thiotrifluoromethylation of the chiral substrate 1-(1-(3,4-dihydronaphthalen-1-yl)propyl)-3-phenylthiourea provided adduct **2gn** in moderate yield as a mixture of diastereomers (ratio = 3.5 : 1).^15^ Thiazines are also within reach applying this methodology. The spirocyclic product **2ka** was obtained in 53% yield and an eroded d.r. = 6 : 1 favoring the anti-isomer. Styrenes, with different points of attachment for the thiourea, delivered additional trifluoromethylated scaffolds. The 2,2,2-trifluoroethyl-substituted 4*H*-benzo[*d*][1,3]-thiazin-2-amines **2la** and **2ma** were obtained in moderate yields. Products possessing the CF_3_ group on the thiazine ring itself were accessible from 3-substituted 1-cinnamyl-thioureas; for example, **2na** was isolated in 40% yield with a d.r. > 20 : 1. In this series, substituents on the aryl rings are well tolerated. The reaction with the internal alkyl-substituted alkene, (*E*)-1-(hex-2-en-1-yl)-3-phenylthiourea delivered a mixture of 5-*exo*- and 6-*endo*-regioisomers in a ∼1 : 1 ratio (isolated yields were 22% and 20%, respectively).^15^ The spirocyclic thiazine anti-**2ra**, a CF_3_-substituted analogue of a neuroprotector,^18^ was prepared in 60% yield (d.r. > 20 : 1, after purification). A larger scale reaction on 2.3 mmol provided consistent yield of **2ra** (61%), an indicator of the robustness of the process. Thiazine **2rc** is a trifluoromethylated analogue of a scaffold found in BACE-1 inhibitors for treating Alzheimer's disease;^19^ this thiazine was obtained by deprotection with CAN of the *N*-PMB group of **2rb**. Finally, the method also gave access to the CF_3_-containing thiazepine **2sa**.

## Mechanistic experiments

**Scheme 3:**
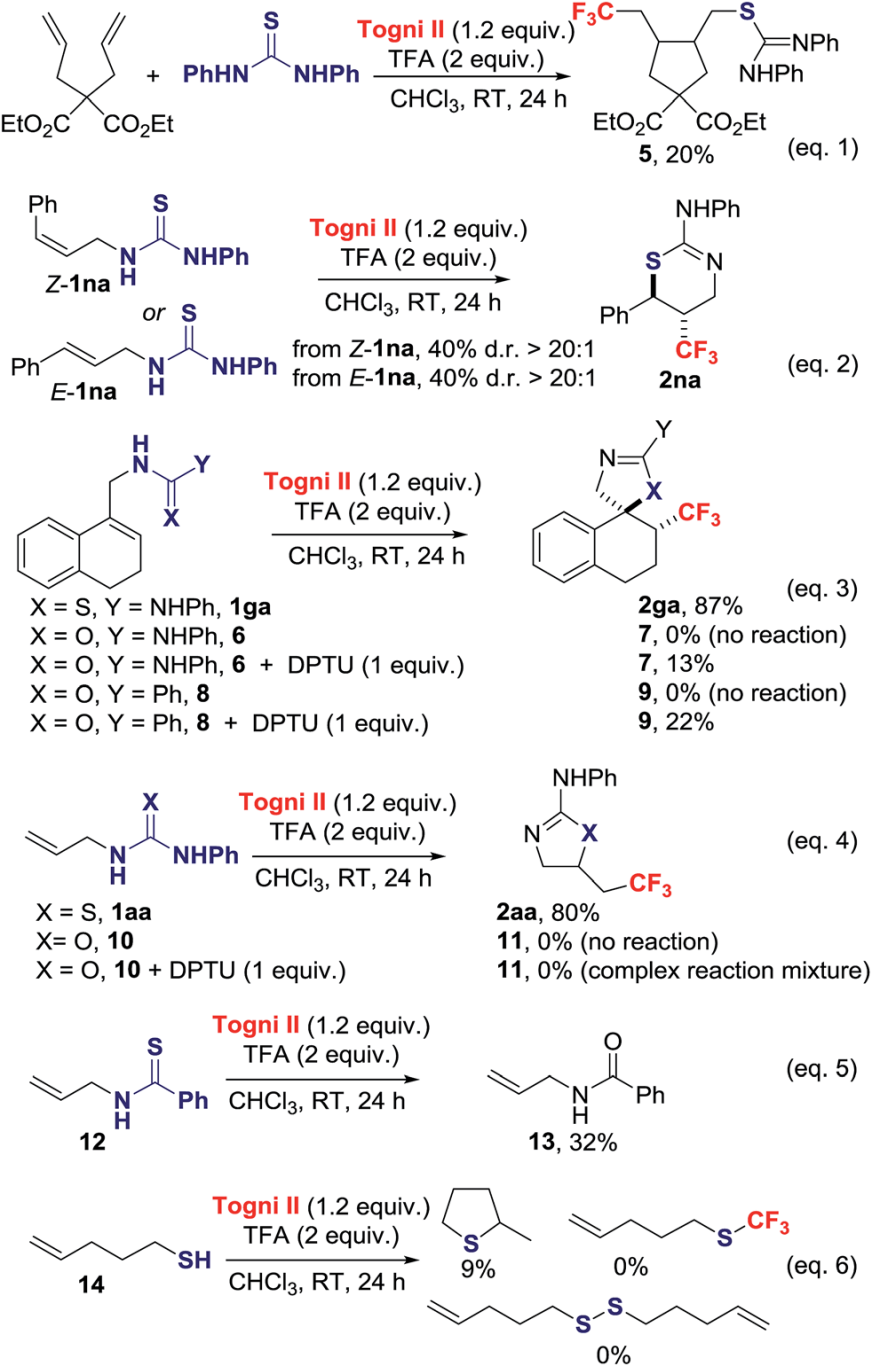
Mechanistic experiments.

We probed the mechanism of this reaction with a series of experiments (Scheme 3). The presence of 1 equiv. of TEMPO significantly inhibited the thiotrifluoromethylation of **1aa**, yielding 23% of TEMPO-CF_3_ and 6% of **2aa**.^15^ Complete inhibition for the formation of **2aa** was observed in the presence of benzoquinone. The cyclopentane **5** was isolated in 20% yield when diethyl 2,2-diallylmalonate was submitted to the reaction conditions in the presence of 1 equiv. of *N*,*N*-diphenylthiourea (DPTU);^20^ in the absence of thiourea, no reaction occurred (eqn (1)). Both *E*-**1na** and *Z*-**1na** gave anti-**2na** with d.r. > 20 : 1 (eqn (2)). Collectively, these data indicate that a CF_3_ radical is involved in the reaction.

Next, we investigated the uniqueness of the thiourea functionality for its ability to induce CF_3_˙ formation. We compared the reactivity of the thiourea **1ga** with the corresponding urea **6** and amide **8** (eqn (3)). We found that **6** and **8** did not react under the standard reaction conditions. Notably, the cyclized products **7** and **9** were isolated in 13% and 22% yield respectively, when the trifluoromethylation was performed in the presence of 1 equiv. of DPTU. In a similar vein, 1-allyl-3-phenylurea **10** did not react under the standard reaction conditions, but was consumed in the presence of DPTU with evidence that CF_3_ radical addition to the alkene took place, but cyclization to **11** did not occur (eqn (4)).^15^ The thiourea therefore acts as an activator leading to CF_3_˙ formation, and subsequent addition of this radical on the C=C π bond. The contrasting reactivity of thiourea and urea is consistent with their oxidation potentials (+1.19 V *vs.* SCE in CH_3_CN for thiourea **1aa** and +1.56 V *vs.* SCE in CH_3_CN for urea **10**); similar values were found for cyclic voltammetry measurements performed in CH_3_CN in the presence of TFA.^15^ Moreover, thioureas are superior to ureas for their ability to react with radical acceptor, an additional factor that accounts for the observed difference of reactivity. We considered next thioamides and thiols as alternative S-sources. Under our standard reaction conditions, the thioamide **12** failed to provide the product of thiotrifluoromethylation, but led instead to the corresponding amide **13** (eqn (5)).^15,21^ Pent-4-ene-1-thiol **14** underwent intramolecular thiol–ene ring closure and side reactions other than S–CF_3_ bond formation or oxidative S–S dimerization (eqn (6)).^15,22^ The thiourea is therefore unique to enable orchestrated alkene trifluoromethylation followed by *S*-cyclization.

**Scheme 4:**
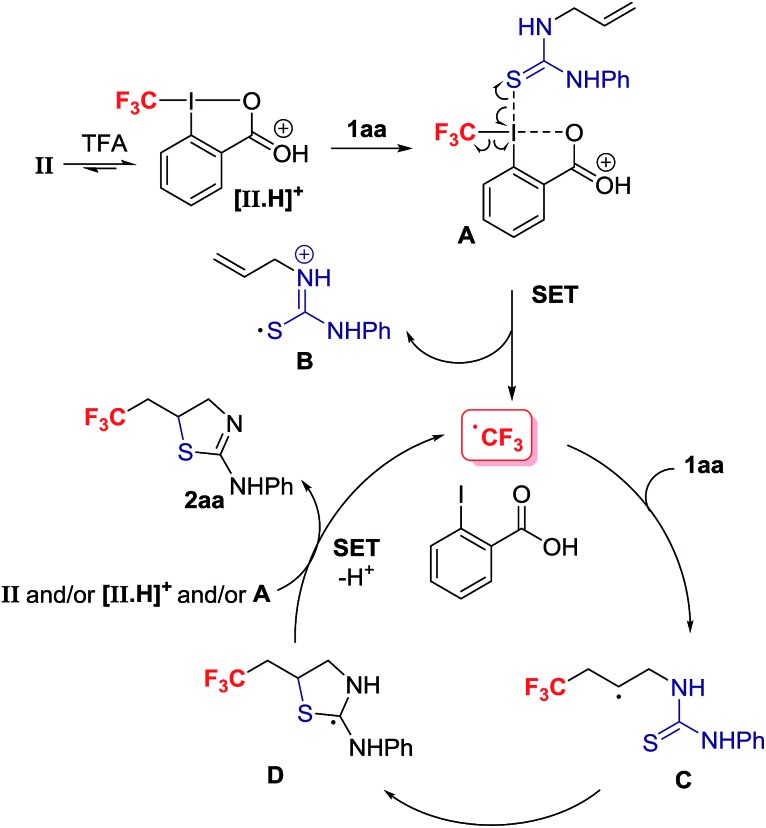
Proposed mechanism.

Mechanistically, we discarded the possibility of *S*-cyclization prior to trifluoromethylation because this sequence would convert alkenes such as **1na** into a thiazoline *via* 5-*exo*-trig cyclization, and the thiazine anti-**2na** is the only product observed in the crude reaction mixture (eqn (2)).^23^ We propose that activation of the Togni reagent **II** with TFA affords the highly electrophilic iodine(iii) species **[II.H]^+^** that can associate with **1aa** *via* iodine–sulphur coordination leading to **A**. Coordination of thiourea to the highly electrophilic I(iii) in **[II.H]^+^** is unprecedented, but S–I(iii) coordination has been evoked in the S–CF_3_ bond formation for thiols reacting with the Togni reagent.^24^ Homolytic dissociation releases **B**, iodobenzoic acid and the electrophilic radical CF_3_˙, which is suited to add regioselectively to the alkene substrate **1aa**. The alternative dissociative electron transfer pathway towards CF_3_ radical formation is also plausible. The resultant carbon radical **C** undergoes ring closure with C–S bond formation to provide adduct **D**, which should be easier to oxidize than **C**; SET to the Togni reagent **II**, **[II.H]^+^** and/or **A** affords after proton transfer **2aa**, and CF_3_˙ that starts a new reaction cycle.^25^ For radicals arising from CF_3_˙ addition to aryl-substituted alkenes, oxidation prior to *S*-cyclization is viable (Scheme 4).

## Conclusions

In summary, we developed the first trifluoromethylation followed by *S*-cyclization across C=C π bonds using thiourea as the S-source. The substrate itself, through its thiourea functionality, acts as an initiator, thereby avoiding metal species or light/photoredox catalysts to induce facile formation of the CF_3_ radical that adds to the alkene. Thiourea can react with C-centered radical, so a range of alkenes including unactivated systems underwent facile thio-trifluoromethylation. This reaction is an attractive method for medicinal and other applications, because of its broad substrate scope, anti-selectivity and operational simplicity. The discovery that *N*,*N*-diphenylthiourea is an effective additive to induce the trifluoromethylation-cyclization of ureas and benzamides opens the possibility to investigate the value of this category of activators for the development of novel metal-free trifluoromethylation across double bonds.
