# Supplementary material for: The dual role of thiourea in the thiotrifluoromethylation of alkenes
Source: Chem Sci. 2016 Sep 30;8(2):1195–9. doi: 10.1039/c6sc02790c (PMC5369526; doi:10.1039/c6sc02790c)

The Dual Role of Thiourea in Thiotrifluoromethylation of Alkenes

Paolo Ricci,[†∫] Tanatorn Khotavivattana,[†∫] Lukas Pfeifer,[†] Maurice Médebielle,[&] John Richard Morphy,[‡] and Véronique Gouverneur*,[†]

[†] Chemistry Research Laboratory, University of Oxford, 12 Mansfield Road, OX1 3TA, Oxford, United Kingdom

[‡] Medicinal Chemistry, Eli Lilly and Company Limited, Erl Wood Manor, Sunninghill Road, Windlesham, Surrey, GU20 6PH, United Kingdom

[&] Université Claude Bernard Lyon I, ICBMS UMR CNRS – UCBL – INSA 5246, Equipe "Synthèse de Molécules d'Intérêt Thérapeutique (SMITH)" 43 bd du 11 Novembre 1918 Villeurbanne 69622, France

Table of Contents

1. **General Experimental Information** **S2**
2. **General experimental Procedures and Characterisation Data** **S2**
3. **Mechanistic Experiments** **S52**
   - **3.1 Thiotrifluoromethylation of 1aa in presence of TEMPO** **S52**
   - **3.2 Thiotrifluoromethylation of Diethyl 2,2-diallyl Malonate** **S53**
   - **3.3 Trifluoromethylation-Cyclization of Urea 6 and Amide 8** **S55**
   - **3.4 Thiotrifluoromethylation of Thioamide 12** **S62**
   - **3.5 Thiotrifluoromethylation of Pent-4-ene-1-thiol 14** **S63**
   - **3.6 Thiotrifluoromethylation of 1aa with Different Acids** **S67**
   - **3.7 Additional Studies on the Selectivity** **S68**
4. **Cyclic Voltammetry Analysis** **S74**
5. **NMR Spectra of Novel Compounds** **S77**
6. **Crystallographic Data** **S177**

1. General Experimental Information

All NMR spectra were recorded on Bruker AVF400, AVB400 and AVC500 spectrometers. Proton and carbon-13 NMR spectra are reported as chemical shifts (δ) in parts per million (ppm) relative to the solvent peak using the Bruker internal referencing procedure (edlock). Fluorine-19 NMR spectra are referenced relative to $CFCl_3$ in $CDCl_3$. Coupling constants (*J*) are reported in units of hertz (Hz). The following abbreviations are used to describe multiplicities – s (singlet), d (doublet), t (triplet), q (quartet), m (multiplet), br s (broad singlet). High resolution mass spectra (HRMS, *m/z*) were recorded on a Bruker MicroTOF spectrometer using positive electrospray ionization (ESI^+) or on a Micromass GCT spectrometer using filed ionization (FI^+) or chemical ionization (CI^+). Infrared spectra were recorded either as the neat compound or in a solution using a Bruker Tensor 27 FT-IR spectrometer. Absorptions are reported in wavenumbers (cm^{-1}) and only peaks of interest are reported. Melting points of solids were measured on a Griffin apparatus and are uncorrected. IUPAC names were obtained using the ACD/I-Lab service. Solvents were purchased from Fisher, Acros or Sigma-Aldrich. When dry solvents were required they were purified by expression through an activated alumina column built according to the procedures described by Pangborn and Grubbs.[1] Chemicals were purchased from Acros, Alfa Aesar, Fisher, Fluorochem, Sigma-Aldrich and used as received. Reactions were monitored by thin-layer chromatography (TLC) carried out on Merck Kiesegel 60 F254 plates, silica gel column chromatography was performed over Merck silica gel C60 (40-60 µm).

[1] Pangborn, A. B.; Giardello M. A.; Grubbs, R. H.; Rosen, R. K.; Timmers, F. J. *Organometallics* **1996**, *15*, 1518.

2. General Experimental Procedures and Characterisation data

General procedure A *(Preparation of thioureas from amines)*
To a solution of appropriate amine (1.0−1.2 equiv.) in DCM (0.1 M) was added an appropriate isothiocyanate (1.0−1.2 equiv.) and the mixture was stirred at room temperature for 16 hours under nitrogen atmosphere. The reaction mixture was concentrated *in vacuo*. The crude product was purified by silica gel column chromatography or precipitation by redissolving in DCM followed by addition of Et_2O and *n*-pentane. The solid was collected by filtration and washed with *n*-pentane (x2).

General procedure B *(for the synthesis of thioureas* ***1ca, 1da, 1ea*** *and* ***1fa****)*
The amine precursor was synthesized by a known procedure.[2,3] To a solution of the corresponding methallyl bromide (5 mmol) in DCM (12.5 mL), PPh_3 (1.70 g, 6.5 mmol) was

added portionwise at 0 °C and the reaction was stirred at 0 °C for 3 hours. The solvent was removed *in vacuo* and the crude mixture was dissolved in a mixture 4:1 of THF:H_2O (15 mL) and treated with NaN_3 (420 mg, 6.5 mmol). The reaction was stirred at room temperature for 2 hours and then PPh_3 (2.20 g, 8.4 mmol) was added. The reaction was stirred for an additional 12 hours and then THF was removed *in vacuo*. The resulting oil was diluted with concentrated HCl (15 mL) and stirred for 30 minutes at room temperature. The suspension was then extracted with Et_2O (20 mL x 2). The aqueous layer was then basified by adding solid KOH and extracted with Et_2O (2 x 40 mL). The combined organic phase was then dried over Na_2SO_4 and concentrated *in vacuo* to provide the crude methallyl amine. DCM (20 mL) and phenyl isothiocyanate (0.676 g, 5 mmol) were added under N_2 atmosphere and the reaction was stirred for 16 hours at room temperature. Solvent was then removed and Et_2O (10 mL) was added. The corresponding solid was then filtered and washed with *n*-pentane (2 x 10 mL) affording the desired thiourea.

[2] Kawato, Y.; Kubota, A.; Ono, Y.; Egami, H.; Hamashima, Y. *Org. Lett.* **2015**, *17*, 1244-1247.
[3] Tripathi,C. B.; Mukherjee, S. *Angew. Chem., Int. Ed.* **2013**, *52*, 8450-8453.

General procedure C *(Preparation of isothiocyanates from amines)*
To a solution of appropriate amine (2.0 mmol) in EtOH (4 mL) was added triethylamine (0.28 mL, 2.0 mmol) and carbon disulfide (0.36 mL, 6.0 mmol). The mixture was stirred at room temperature for 2 hours then cooled to 0 °C. Di-tert-butyl dicarbonate (437 mg, 2.0 mmol) and 4-(dimethylamino)pyridine (7.3 mg, 0.06 mmol) were added then the mixture was stirred at room temperature for 4 hours. The reaction mixture was diluted with EtOAc (20 mL) and the organic layer was washed with H_2O (20 mL) and brine (20 mL). The organic layer was dried over $MgSO_4$, filtered and concentrated in vacuo. The crude product was purified by silica gel column chromatography.

General procedure D *(for the synthesis of thioureas **1la** and **1ma**)*
The amine precursor was synthesized by a known procedure.[4] A solution of methyl anthranilate (5 mmol) in Et_2O (20 mL, 0.25 M) under Ar was cooled to -40 °C and MeMgBr (3 M solution in Et_2O, 4.5 mL, 13.5 mmol) was added dropwise over 20 minutes while maintaining vigorous stirring. The mixture was heated to room temperature and stirred for 3 hours. The mixture was quenched with saturated $NaHCO_3$. The organic layer was separated and the aqueous layer was extracted with Et_2O (2 x 40 mL). The combined organic layers were washed with brine (50 mL), dried over $MgSO_4$ and filtered. The solvent was removed *in vacuo* and the crude product was then heated at 230 °C for 20 minutes without stirring. The flask was cooled down to room temperature, Et_2O (100 mL) was added and the organic layer was washed with brine (50 mL), dried over $MgSO_4$ and the solvent removed *in vacuo*. DCM (20 mL) and phenyl isothiocyanate (5 mmol) were added under N_2 atmosphere

and the reaction was stirred for 16 hours at room temperature. Solvent was then removed and Et_2O (10 mL) was added. The corresponding solid was then filtered and washed with *n*-pentane (2 x 10 mL) affording the desired thiourea.

[4] Mitsuhiro, A.; Fujii, Y.; Kato, H.; Fukuda, H.; Matsumoto, T.; Ito, M.; Abe, H.; Ito, H. Shuto, S. *Angew. Chem., Int. Ed.* **2013**, *52*, 1003-1007.

***General procedure E** (Reduction of aldehydes with $NaBH_4$)*

Into a solution of appropriate aldehyde (12.0 mmol) in MeOH (20 mL) was added $NaBH_4$ (302 mg, 8.0 mmol) at 0 °C. The reaction was stirred at room temperature for 3 hours then quenched with water and the mixture was extracted with Et_2O (x2). Combined organic layers were washed with brine, dried over $MgSO_4$ and concentrated *in vacuo*. The crude alcohol was used in the next step without further purification.

***General procedure F** (Synthesis of N-allyl phthalimides from allyl alcohols)*

To the solution of appropriate allyl alcohol (5.0 mmol) in THF (25 mL) was added PPh_3 (1.31 g, 5.0 mmol), phthalimide (736 mg, 5.0 mmol) and diethyl azodicarboxylate (0.79 mL, 5.0 mmol) at 0 °C. After stirring for 16 hours at room temperature, the reaction mixture was concentrated *in vacuo*. The crude product was either purified by silica gel column chromatography or used in the next step without further purification.

***General procedure G** (Hydrazinolysis of phathalimides)*

Into a solution of appropriate phthalimide (2.5 mmol) in MeOH (25 mL) was added hydrazine monohydrate (0.18 mL, 3.75 mmol) and the reaction mixture was stirred for 16 hours under reflux. The mixture was cooled, concentrated *in vacuo*, redissolved in 1M HCl (20 mL) and the resulting suspension was filtered. The filtrate was basified to pH = 10 using 5M NaOH and extracted with Et_2O (x2). Combined organic layers were washed with brine, dried over $MgSO_4$ and concentrated *in vacuo*. The crude amine was used in the next step without further purification.

1-Allyl-3-phenylthiourea (1aa)

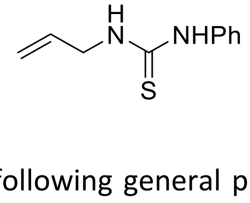

The title compound was prepared following general procedure A using allylamine (0.3 ml, 4.0 mmol), DCM (30 mL) and phenyl isothiocyanate (0.53 mL, 4.4 mmol). The crude product

was purified by precipitation to give the title compound (705 mg, 92 % yield) as a white solid.

(^{1}H NMR, 400 MHz, $CDCl_3$): δ (ppm) = 8.01 (br s, 1H), 7.43 (t, *J* = 7.6 Hz, 2H), 7.30 (t, *J* = 7.5 Hz, 1H), 7.22 (d, *J* = 7.6 Hz, 2H), 6.05 (br s, 1H), 5.87 (*app.* dq, *J* = 11.0, 5.7 Hz, 1H), 5.21-5.11 (m, 2H), 4.29 (dt, *J* = 5.7, 1.2 Hz, 2H); (^{13}C NMR, 101 MHz, $CDCl_3$): δ (ppm) = 180.4, 136.1, 133.1, 130.0, 127.1, 125.1, 116.9, 47.5; IR: 3208, 2360, 1644, 1529, 1313, 1235, 1192, 918 cm^{-1}; HRMS (ESI) for $C_{10}H_{13}N_2S$ $[M+H]^+$ requires 193.0790 found 193.0797; Mp. = 90–92 °C.

1-(2-Methylallyl)-3-phenylthiourea (1ba)

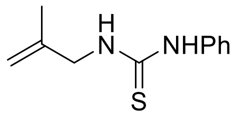

The title compound was prepared following general procedure A using 2-methylprop-2-en-1-amine (284 mg, 4.0 mmol), DCM (30 mL) and phenyl isothiocyanate (0.53 mL, 4.4 mmol). The crude product was purified by precipitation to give the title compound (745 mg, 90 % yield) as a white solid.

(^{1}H NMR, 400 MHz, $CDCl_3$): δ (ppm) = 8.32 (br s , 1H), 7.45 (t, *J* = 7.7 Hz, 2H), 7.33 (t, *J* = 7.5 Hz, 1H), 7.27 (d, *J* = 7.6 Hz, 2H), 6.12 (br s , 1H), 4.86 (s, 1H), 4.79 (s, 1H), 4.24 (d, *J* = 4.4 Hz, 2H), 1.76 (s, 3H); (^{13}C NMR, 101 MHz, $CDCl_3$): δ (ppm) = 180.9, 141.1, 135.9, 130.2, 127.4, 125.4, 111.1, 50.7, 20.5; IR: 3215, 2980, 2360, 1596, 1533, 1496, 1451, 1349, 1313, 1234, 1189, 1028, 943, 892 cm^{-1}; HRMS (ESI) for $C_{11}H_{15}N_2S$ $[M+H]^+$ requires 207.0951 found 207.0951; Mp. = 83–84 °C.

1-Phenyl-3-(2-phenylallyl)thiourea (1ca)

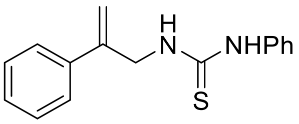

The title compound was prepared following general procedure B using (3-bromoprop-1-en-2-yl)benzene (980 mg, 5.0 mmol). The crude product was purified by precipitation to give the title compound (540 mg, 40 % overall yield) as a white solid.

(^{1}H NMR, 400 MHz, $CDCl_3$): δ (ppm) = 8.02 (br s, 1H), 7.44-7.31 (m, 5H), 7.29-16 (m, 3H), 6.91 (d, *J* = 7.6 Hz, 2H), 5.98 (br s, 1H), 5.42 (s, 1H), 5.21 (s, 1H), 4.73 (d, *J* = 5.2 Hz, 2H); (^{13}C NMR, 101 MHz, $CDCl_3$): δ (ppm) = 180.1, 143.5, 138.1, 135.9, 129.8, 128.4, 128.0, 126.7, 126.1, 124.6, 114.2, 49.1; IR = 3379, 3200, 3056, 1632, 1598, 1495, 1350, 1297, 779 cm^{-1}; HRMS (ESI) for $C_{16}H_{17}N_2S$ $[M+H]^+$ requires 269.1107 found 269.1106; Mp. 120-122°C.

1-(2-(4-Fluorophenyl)allyl)-3-phenylthiourea (1da)

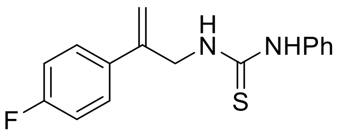

The title compound was prepared following general procedure B using 1-(3-bromoprop-1-en-2-yl)-4-fluorobenzene (1.08 g, 5.0 mmol), providing the title compound (472 mg, 33 % overall yield) as a white solid.

(^{1}H NMR, 400 MHz, $CDCl_3$): δ (ppm) = 7.76 (br s, 1H), 7.44-7.36 (m, 2H), 7.31 (t, *J* = 7.4 Hz, 2H), 7.30-7.22 (m, 1H), 7.05 (t, *J* = 8.1 Hz, 2H), 6.96 (d, *J* = 7.7 Hz, 2H), 5.92 (br s, 1H), 5.38 (s, 1H), 5.19 (br s, 1H), 4.72 (d, *J* = 5.3 Hz, 2H); (^{13}C NMR, 101 MHz, $CDCl_3$): δ (ppm) = 180.6, 162.7 (d, *J* = 248.4 Hz), 142.8, 135.8, 134.2 (d, *J* = 3.3 Hz), 130.1, 128.0 (d, *J* = 8.1 Hz), 127.2, 125.0, 115.4 (d, J = 21.4 Hz), 114.3, 49.3; (^{19}F NMR, 377 MHz, $CDCl_3$): δ (ppm) = −113.6 (m, 1F); IR: 3208, 2360, 1644, 1530, 1419, 1313, 1236, 1191, 993 cm^{-1}; HRMS (ESI) for $C_{16}H_{16}N_2FS$ $[M+H]^+$ requires 287.1013 found 287.1013; Mp. 118-120 °C.

.

1-(2-(4-chlorophenyl)allyl)-3-phenylthiourea (1ea)

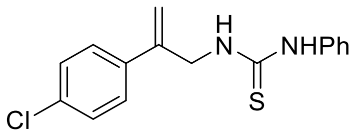

The title compound was prepared following general procedure B using 1-(3-bromoprop-1-en-2-yl)-4-chlorobenzene (1.16 g, 5.0 mmol). The crude product was purified by precipitation to give the title compound (560 mg, 36 % overall yield) as a white solid.

(^{1}H NMR, 400 MHz, $CDCl_3$): δ (ppm) = 7.81 (br s, 1H), 7.41-7.31 (m, 6H), 7.30-7.24 (m, 1H), 6.97 (d, *J* = 7.6 Hz, 2H), 5.95 (br s, 1H), 5.44 (s, 1H), 5.24 (s, 1H), 4.73 (d, *J* = 5.3 Hz, 2H); δ =; (^{13}C NMR, 101 MHz, $CDCl_3$): δ (ppm) = 180.6, 142.6, 136.8, 135.8, 134.0, 130.0, 128.7, 127.6, 127.2, 125.0, 114.9, 49.1; IR 3259, 2936, 2832, 1615, 1532, 1322, 1262, 1165, 1119, 1067, 1016, 839 cm^{-1}: ; HRMS (ESI) for $C_{16}H_{16}ClN_2S$ $[M+H]^+$ requires 303.0717 found 303.0717; Mp. 135-137°C.

1-phenyl-3-(2-(4-(trifluoromethyl)phenyl)allyl)thiourea (1fa)

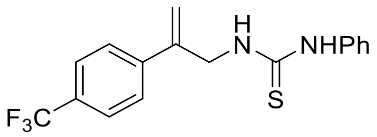

The title compound was prepared following general procedure B using 1-(3-bromoprop-1-en-2-yl)-4-(trifluoromethyl)benzene (1.31 g, 5.0 mmol). The crude product was purified by precipitation to give the title compound (420 mg, 25 % overall yield) as a white solid.
(^{1}H NMR, 400 MHz, $CDCl_3$): δ (ppm) = 7.67 (br s, 1H), 7.62 (d, *J* = 8.3 Hz, 2H), 7.53 (d, *J* = 8.3 Hz, 2H), 7.34-7.21 (m, 3H), 7.30-7.24 (m, 1H), 6.94 (d, *J* = 7.4 Hz, 2H), 5.90 (br s, 1H), 5.51 (s, 1H), 5.31 (s, 1H), 4.76 (d, *J* = 5.3 Hz, 2H); (^{13}C NMR, 101 MHz, $CDCl_3$ + DMSO-d_6 (5% v/v)): δ (ppm) = 180.9, 142.8, 141.9, 136.7, 130.0 (q, *J* =32.6 Hz), 129.6, 126.5, 126.4, 125.4 (q, *J* = 4.1 Hz), 125.3 (q, *J* = 272.1 Hz), 124.6, 116.0, 48.5; (^{19}F NMR, 377 MHz, $CDCl_3$): δ (ppm) = −62.6 (s, 3F); IR 3208, 2361, 1595, 1531, 1496, 1451, 1314, 1237, 1419, 1237, 1192, 1073, 993 cm^{-1}; HRMS (ESI) for $C_{16}H_{13}F_3N_2S$ $[M+H]^+$ requires 337.0976 found 337.0976; Mp. 112-114°C.

1-((3,4-Dihydronaphthalen-1-yl)methyl)-3-phenylthiourea (1ga)

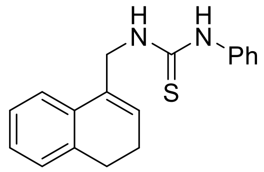

The title compound was prepared following general procedure A using (3,4-dihydronaphthalen-1-yl)methanamine[5] (796 mg, 5.0 mmol), DCM (50 mL) and phenyl isothiocyanate (0.60 mL, 5.0 mmol). The crude product was purified by precipitation to give the title compound (1.28 g, 87 % yield) as a white solid.
(^{1}H NMR, 400 MHz, $CDCl_3$): δ (ppm) = 8.45 (br s, 1H), 7.47−7.40 (m, 3H), 7.37−7.23 (m, 6H), 6.20 (br s , 1H), 6.16 (t, *J* = 4.4 Hz, 1H), 4.81 (d, *J* = 4.4 Hz, 2H), 2.87 (t, *J* = 8.1 Hz, 2H), 2.44−2.39 (m, 2H); (^{13}C NMR, 101 MHz, $CDCl_3$): δ (ppm) = 180.1, 136.3, 136.0, 132.6, 132.2, 130.0, 128.5, 127.7, 127.3, 126.9, 126.6, 124.7, 122.7, 48.0, 27.7, 22.9; IR: ν 3382, 3201, 2934, 2883, 2830, 1596, 1530, 1495, 1449, 1344, 1298, 1236, 1185, 1134, 1072, 1022, 970 cm^{-1}; HRMS (ESI) for $C_{18}H_{19}N_2S$ $[M+H]^+$ requires 295.1264 found 295.1264; Mp. 105−108 °C.

[5] Rauniyar, V; Lackner, A. D.; Hamilton, G. L. ; Toste, F. D. *Science* **2011**, *334*, 1681−1684.

1-(4-Bromophenyl)-3-((3,4-dihydronaphthalen-1-yl)methyl)thiourea (1gb)

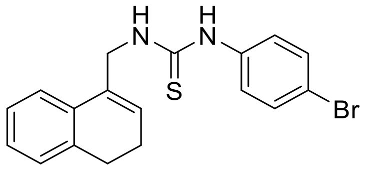

The title compound was prepared following general procedure A using (3,4-dihydronaphthalen-1-yl)methanamine[5] (478 mg, 3.0 mmol), DCM (30 mL) and 4-bromophenyl isothiocyanate (642 mg, 3.0 mmol). The crude product was purified by precipitation to give the title compound (821 mg, 73 % yield) as a white solid.
(^{1}H NMR, 400 MHz, $CDCl_3$): δ (ppm) = 8.36 (br s, 1H), 7.43 (dt, *J* = 8.6, 2.4 Hz, 2H), 7.29−7.17 (m, 4H), 7.00 (d, *J* = 8.3 Hz, 2H), 6.06 (t, *J* = 4.3 Hz, 1H), 6.00 (br s , 1H), 4.67 (d, *J* = 3.9 Hz, 2H), 2.77 (t, *J* = 8.1 Hz, 2H), 2.44−2.29 (m, 2H); (^{13}C NMR, 101 MHz, $CDCl_3$): δ (ppm) = 180.1, 136.4, 135.3, 133.2, 132.6, 132.1, 129.1, 127.9, 127.6, 126.8, 126.3, 122.8, 120.3, 48.2, 27.8, 23.0; IR: 3232, 2934, 1534, 1488, 1452, 1308, 1283, 1238, 1099, 1072, 1010, 970, 908, 823 cm^{-1}; HRMS (ESI) for $C_{18}H_{18}N_2BrS$ $[M+H]^+$ requires 373.0369 found 373.0370; Mp. 112−114 °C.

1-(4-Iodophenyl)-3-((3,4-dihydronaphthalen-1-yl)methyl)thiourea (1gc)

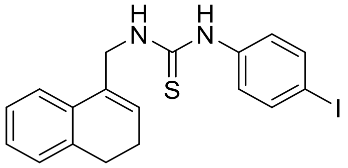

The title compound was prepared following general procedure A using (3,4-dihydronaphthalen-1-yl)methanamine[5] (478 mg, 3.0 mmol), DCM (30 mL) and 4-iodophenyl isothiocyanate (735 mg, 3.0 mmol). The crude product was purified by precipitation to give the title compound (913 mg, 72 % yield) as a white solid.
(^{1}H NMR, 400 MHz, $CDCl_3$): δ (ppm) = 8.32 (br s, 1H), 7.72 (d, *J* = 8.6 Hz, 2H), 7.39−7.27 (m, 4H), 6.86 (d, *J* = 7.8 Hz, 2H), 6.16 (t, *J* = 3.9 Hz, 1H), 6.11 (br s , 1H), 4.77 (d, *J* = 3.2 Hz, 2H), 2.87 (t, *J* = 7.9 Hz, 2H), 2.44−2.39 (m, 2H); (^{13}C NMR, 101 MHz, $CDCl_3$): δ (ppm) = 180.0, 139.0, 136.4, 135.9, 132.5, 132.0, 129.0, 127.9, 127.5, 126.7, 126.3, 122.7, 91.3, 48.2, 27.7, 22.9; IR: 3237, 3057, 2934, 2830, 2059, 1533, 1485, 1453, 1309, 1281, 1238, 1059, 1007, 970, 820 cm^{-1}; HRMS (ESI) for $C_{18}H_{18}N_2IS$ $[M+H]^+$ requires 421.0230 found 421.0223; Mp. 147−149 °C.

1-((3,4-Dihydronaphthalen-1-yl)methyl)-3-(4-methoxyphenyl)thiourea (1gd)

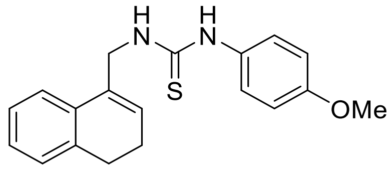

The title compound was prepared following general procedure C using (3,4-dihydronaphthalen-1-yl)methanamine[5] (478 mg, 3.0 mmol), DCM (30 mL) and 4-methoxyphenyl isothiocyanate (495 mg, 3.0 mmol). The crude product was purified by precipitation to give the title compound (749 mg, 77 % yield) as a white solid.
(^{1}H NMR, 400 MHz, $CDCl_3$): δ (ppm) = 7.67-7.53 (br s, 1H), 7.26−7.09 (m, 4H), 7.02 (d, *J* = 8.7 Hz, 2H), 6.83 (d, *J* = 8.7 Hz, 2H), 5.99 (t, *J* = 4.6 Hz, 1H), 5.78 (br s , 1H), 4.65 (d, *J* = 4.7 Hz, 2H), 3.77 (s, 3H), 2.72 (t, *J* = 7.9 Hz, 2H), 2.32−2.23 (m, 2H); (^{13}C NMR, 101 MHz, $CDCl_3$): δ (ppm) = 180.7, 158.6, 136.2, 132.7, 132.3, 128.4, 128.1, 127.6, 127.2 (2C), 126.6, 122.7, 115.1, 55.4, 47.9, 27.7, 22.9; IR: 3191, 2933, 2833, 1510, 1298, 1243, 1167, 1032, 971, 909, 831 cm^{-1}; HRMS (ESI) for $C_{19}H_{21}N_2OS$ [M+H]$^+$ requires 325.1369 found 325.1368; Mp.100-102 °C.

1-((3,4-Dihydronaphthalen-1-yl)methyl)-3-(naphthalen-2-yl)thiourea (1ge)

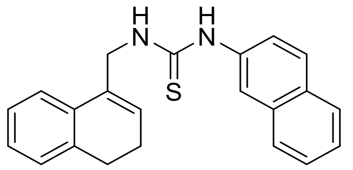

The title compound was prepared following general procedure A using (3,4-dihydronaphthalen-1-yl)methanamine[5] (478 mg, 3.0 mmol), DCM (30 mL) and 2-naphthyl isothiocyanate (555 mg, 3.0 mmol). The crude product was purified by precipitation to give the title compound (804 mg, 78 % yield) as a white solid.

(^{1}H NMR, 400 MHz, $CDCl_3$): δ (ppm) = 7.97 (br s, 1H), 7.93 (d, *J* = 8.5 Hz, 1H), 7.85 (d, *J* = 8.3 Hz, 1H), 7.81 (d, *J* = 8.3 Hz, 1H), 7.55-7.48 (m, 1H), 7.45-7.37 (m, 2H), 7.32 (d, *J* = 7.3 Hz, 1H), 7.24-7.19 (m, 1H), 7.18-7.05 (m, 3H), 5.86 (t, *J* = 4.4 Hz, 1H), 5.67 (br s , 1H), 4.64 (dd, *J* = 5.2, 1.1 Hz, 2H), 2.61 (t, *J* = 7.9 Hz, 2H), 2.21−2.11 (m, 2H); (^{13}C NMR, 101 MHz, $CDCl_3$) δ (ppm) = 181.3, 136.2, 134.5, 132.6, 132.3, 131.6, 129.7, 128.8, 128.3, 128.1, 127.6, 127.3, 127.2, 127.0, 126.6, 125.6, 124.9, 122.7, 122.5, 48.0, 27.6, 22.8; IR: 3377, 2932, 2361, 2341, 1737, 1595, 1531, 1341, 1200, 1017 cm^{-1}; HRMS (ESI) for $C_{22}H_{21}N_2S$ [M+H]$^+$ requires 345.1420 found 345.1421; Mp. 154-156 °C.

1-((3,4-Dihydronaphthalen-1-yl)methyl)-3-(4-(trifluoromethyl)phenyl)thiourea (1gf)

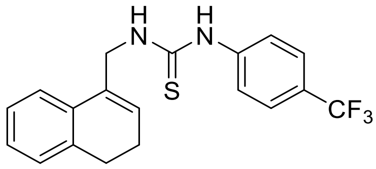

The title compound was prepared following general procedure A using (3,4-dihydronaphthalen-1-yl)methanamine[5] (478 mg, 3.0 mmol), DCM (30 mL) and 4-(trifluoromethyl)phenyl isothiocyanate (609 mg, 3.0 mmol). The crude product was purified by precipitation to give the title compound (965 mg, 89 % yield) as a white solid.

(^{1}H NMR, 400 MHz, $CDCl_3$): δ (ppm) = 8.07 (br s, 1H), 7.54 (d, *J* = 8.4 Hz, 2H), 7.30−7.14 (m, 6H), 6.11 (br s, 1H), 6.09 (t, *J* = 4.3 Hz, 1H), 4.68 (d, *J* = 4.2 Hz, 2H), 2.76 (t, *J* = 7.9 Hz, 2H), 2.35−2.27 (m, 2H); (^{13}C NMR, 101 MHz, $CDCl_3$): δ (ppm) = 180.0, 139.8, 136.4, 132.4, 131.9, 129.2, 127.9, 127.9 (q, *J* = 32.8 Hz), 127.8, 127.0, 126.7, 123.8 (q, *J* = 272.5 Hz), 123.5, 122.6, 48.1, 27.7, 22.9; (^{19}F NMR, 377 MHz, $CDCl_3$): δ (ppm) = −62.5 (s , 3F); IR: 3254, 2360, 1616, 1535, 1323, 1262, 1067, 1016, 840 cm^{-1}; HRMS (ESI) for $C_{19}H_{18}F_3N_2S$ $[M+H]^+$ requires 363.1137 found 363.1136; Mp. 120-122 °C.

Methyl 4-(3-((3,4-dihydronaphthalen-1-yl)methyl)thioureido)benzoate (1gg)

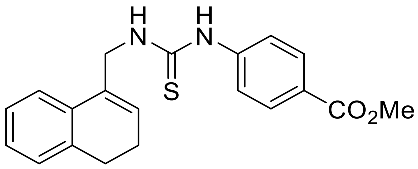

The title compound was prepared following general procedure A using (3,4-dihydronaphthalen-1-yl)methanamine[5] (478 mg, 3.0 mmol), DCM (30 mL) and methyl 4-isothiocyanatobenzoate (580 mg, 3.0 mmol). The crude product was purified by precipitation to give the title compound (862 mg, 82 % yield) as a white solid.

(^{1}H NMR, 400 MHz, $CDCl_3$): δ (ppm) = 8.04 (br s, 1H), 7.94 (d, *J* = 8.4 Hz, 2H), 7.30−7.07 (m, 6H), 6.17(br s , 1H), 6.08 (t, *J* = 4.4 Hz, 1H), 5.78 (br s , 1H), 4.68 (d, *J* = 4.0 Hz, 2H), 3.88 (s, 3H), 2.75 (t, *J* = 7.9 Hz, 2H), 2.35−2.25 (m, 2H); (^{13}C NMR, 101 MHz, $CDCl_3$): δ = 179.8, 166.1, 140.8, 136.3, 132.4, 132.0, 131.3, 129.2, 127.9, 127.5, 127.2, 126.7, 122.6, 122.5, 52.1, 48.1, 27.7, 22.9; IR 3301, 2947, 2360, 1717, 1607, 1528, 1435, 1280, 1176, 1113, 1017, 969, 910, 851, 770, 731 cm^{-1}; HRMS (ESI) for $C_{20}H_{21}N_2O_2S$ $[M+H]^+$ requires 353.1318 found 353.1318; Mp. 128-130 °C.

1-Benzyl-3-((3,4-Dihydronaphthalen-1-yl)methyl)thiourea (1gh)

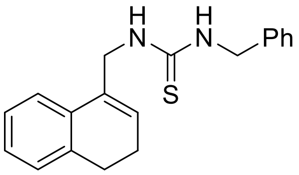

The title compound was prepared following general procedure A using (3,4-dihydronaphthalen-1-yl)methanamine[5] (318 mg, 2.0 mmol), DCM (20 mL) and benzyl isothiocyanate (0.27 mL, 2.0 mmol). The crude product was purified by silica gel column chromatography (eluent: 20 % EtOAc in *n*-pentane) to provide the title compound (318 mg, 52 % yield) as white solid.
(^{1}H NMR, 400 MHz, $CDCl_3$): δ (ppm) = 7.46−7.38 (m, 5H), 7.35−7.26 (m, 4H), 6.25 (br s, 1H), 6.15 (t, *J* = 4.3 Hz, 2H), 5.97 (br s , 1H), 4.75 (br s, 2H), 4.57 (br s, 2H), 2.84 (t, *J* = 8.1 Hz, 2H), 2.42−2.37 (m, 2H); (^{13}C NMR, 101 MHz, $CDCl_3$): δ (ppm) = 181.9, 136.7, 136.3, 132.4, 131.9, 128.8, 128.5, 127.8, 127.8, 127.5, 127.4, 126.7, 122.6, 48.5, 47.0, 27.7, 22.9; IR: 3251, 3061, 2932, 2830, 1547, 1492, 1463, 1344, 1277, 1023, 960, 822 cm^{-1}; HRMS (ESI) for $C_{19}H_{21}N_2S$ $[M+H]^+$ requires 309.1420 found 309.1419; Mp. 90−93 °C.

1-(*Tert*-Butyl)-3-((3,4-dihydronaphthalen-1-yl)methyl)thiourea (1gi)

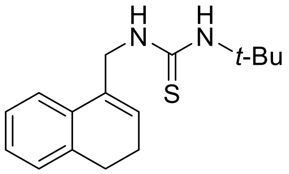

The title compound was prepared following general procedure A using (3,4-dihydronaphthalen-1-yl)methanamine[5] (318 mg, 2.0 mmol), DCM (20 mL) and *tert*-butyl isothiocyanate (0.25 mL, 2.0 mmol). The crude product was purified by precipitation to give the title compound (393 mg, 72 % yield) as a white solid.
(^{1}H NMR, 400 MHz, $CDCl_3$): δ (ppm) = 7.38−7.26 (m, 4H), 6.22 (t, *J* = 4.5 Hz, 1H), 6.17 (br s , 1H), 4.69 (s, 2H), 2.89 (t, *J* = 8.1 Hz, 2H), 2.46−2.41 (m, 2H), 1.43 (s, 9H); (^{13}C NMR, 101 MHz, $CDCl_3$): δ (ppm) = 180.9, 136.3, 132.5 (2C), 128.8, 127.8, 127.4, 126.7, 122.8, 52.7, 48.3, 29.5, 27.8, 23.0; IR 3270, 3061, 2931, 2831, 1535, 1491, 1450, 1392, 1345, 1280, 1198, 1120, 1021, 973, 922, 821 cm^{-1} ; HRMS (ESI) for $C_{16}H_{23}N_2S$ $[M+H]^+$ requires 275.1577 found 275.1575; Mp. 127−129 °C.

1-(Benzo[d]thiazol-2-yl)-3-((3,4-dihydronaphthalen-1-yl)methyl)thiourea (1gj)

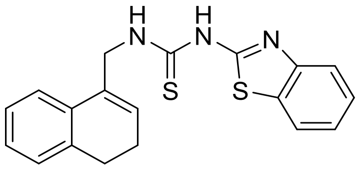

The title compound was prepared following general procedure A for 48 hours using (3,4-dihydronaphthalen-1-yl)methanamine[5] (478 mg mg, 3.0 mmol), DCM (30 mL) and 2-isothiocyanatobenzo[*d*]thiazole (570 mg, 3.0 mmol). The crude product was purified by precipitation to give the title compound (358 mg, 34 % yield) as a white solid.

(^{1}H NMR, 400 MHz, $CDCl_3$): δ (ppm) = 11.3 (br s, 1H), 7.68 (d, *J* = 7.9 Hz, 1H), 7.42 (d, *J* = 7.9 Hz, 1H), 7.38-7.32 (m, 2H), 7.30-7.17 (m, 4H), 6.23 (t, *J* = 4.5 Hz, 1H), 4.83 (dd, *J* = 4.9, 1.0 Hz, 2H), 2.83 (*J* = 7.9 Hz, 2H), 2.42-2.33 (m, 2H); (^{13}C NMR, 101 MHz, $CDCl_3$ + DMSO-d_6 (5% v/v)): δ (ppm) = 178.2, 160.5, 149.0, 136.2, 132.8, 131.9, 129.9, 127.7, 127.5, 127.0, 126.4, 125.9, 123.6, 122.5, 120.7, 120.0, 47.4, 27.6, 22.8; IR: 3168, 3031, 1555, 1524, 1457, 1314, 1017, 914 cm^{-1}; HRMS (ESI) for $C_{19}H_{18}N_3S_2$ $[M+H]^+$ requires 352.0937 found 352.0936; Mp.185-187 °C.

1-((3,4-Dihydronaphthalen-1-yl)methyl)-3-(6-methylpyridin-3-yl)thiourea (1gk)

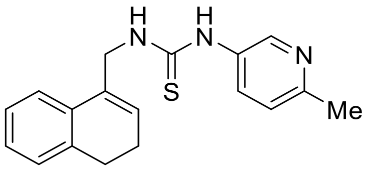

The title compound was prepared following general procedure A for 48 hours at 50°C using (3,4-dihydronaphthalen-1-yl)methanamine[5] (478 mg, 3.0 mmol), DCM (30 mL) and 5-isothiocyanato-2-methylpyridine (451 mg, 3.0 mmol). The crude product was purified by precipitation to give the title compound (708 mg, 78 % yield) as a white solid.

(^{1}H NMR, 400 MHz, $CDCl_3$): δ (ppm) = 8.53 (br s, 1H), 8.16 (d, *J* = 2.1 Hz, 1H), 7.45 (d, *J* = 7.5 Hz, 1H), 7.25-7.06 (m, 4H), 7.02 (d, *J* = 8.3 Hz, 1H), 6.39 (br s, 1H), 6.00 (t, *J* = 3.4 Hz, 1H), 4.62 (s, 1H), 2.67 (t, J = 8.4 Hz, 2H), 2.37 (s, 3H), 2.29-2.19 (m,2H); (^{13}C NMR, 101 MHz, $CDCl_3$): δ (ppm) = 181.0, 156.6, 145.4, 136.3, 133.1, 132.6, 132.0, 131.0, 128.7, 127.8, 127.4, 126.7, 123.7, 122.6, 47.9, 27.6, 23.7, 22.9; IR 3290, 3072, 2984, 1510, 1370,1352, 1314, 1054, 928 cm^{-1}; HRMS (ESI) for $C_{18}H_{20}N_3S$ $[M+H]^+$ requires 310.1372 found 310.1374; Mp. 79-81 °C.

3-((3,4-Dihydronaphthalen-1-yl)methyl)-1-methyl-1-phenylthiourea (1gl)

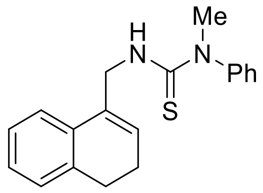

The title compound was prepared following general procedure C using (3,4-dihydronaphthalen-1-yl)methanamine[5] (318 mg, 2.0 mmol), EtOH (4 mL), triethylamine (0.28 mL, 2.0 mmol), carbon disulfide (0.36 mL, 6.0 mmol), di-*tert*-butyl dicarbonate (437 mg, 2.0 mmol) and 4-(dimethylamino)pyridine (7.3 mg, 0.06 mmol). The crude product was purified by silica gel column chromatography (eluent: 10 % Et_2O in *n*-pentane) to provide the isothiocyanate (285 mg, 71 % yield) as a colourless oil.

The isothiocyanate (242 mg, 1.2 mmol) was then dissolved in DCM (12 mL) and was added *N*-methylaniline (0.16 mL, 1.44 mmol) and triethylamine (0.25 mL, 1.8 mmol). The mixture was stirred at room temperature for 16 hours. The reaction mixture was concentrated *in vacuo* and the crude product was purified by redissolving in DCM followed by addition of Et_2O and *n*-pentane. The solid was collected by filtration and washed with *n*-pentane (x2) to give the title compound (324 mg, 87 % yield) as a white solid.

(^1H NMR, 400 MHz, $CDCl_3$): δ (ppm) = 7.50−7.40 (m, 3H), 7.32−7.23 (m, 6H), 6.01 (t, *J* = 4.4 Hz, 1H) 5.45 (br s , 1H), 4.74 (dd, *J* = 4.9, 1.0 Hz, 2H), 3.82 (s, 3H), 2.80 (t, *J* = 7.9 Hz, 2H), 2.38−2.32 (m, 2H); (^{13}C NMR, 101 MHz, $CDCl_3$): δ (ppm) = 181.7, 142.6, 136.3, 132.8, 132.7, 130.4, 128.3, 127.5, 127.4, 127.1, 126.9, 126.5, 122.7, 48.4, 43.3, 27.8, 22.9; IR:3396, 3057, 2931, 2829, 1594, 1513, 1490, 1450, 1384, 1345, 1304, 1280, 1236, 1103, 1072, 1051, 1020, 1001, 970, 812 cm^{-1}; HRMS (ESI) for $C_{19}H_{21}N_2S$ $[M+H]^+$ requires 309.1420 found 309.1419; Mp. 108−111 °C.

1-(1-(3,4-Dihydronaphthalen-1-yl)propyl)-3-phenylthiourea (1gn)

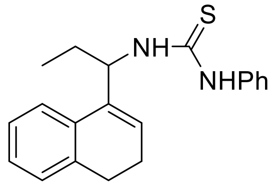

To alpha-tetralone (1.97 ml, 16.2 mmol) and AlCl3 (3 mg, 0.02 mmol) in an oven dried 100 ml round bottom flask under N2 was added TMSCN (1.4 ml, 17.8 mmol). The reaction mixture was warmed to 35 ºC and stirred for 20 h. To the reaction mixture was added 50 ml of Et_2O, and it was then cooled to 0 °C. EtMgBr (8 ml, 24 mmol, 3M solution in Et_2O) was added dropwise and the reaction was then stirred at reflux for 4h. The solution was then

cooled at RT and MeOH (100 ml) was added slowly. Then, $NaBH_4$ (1.25 g, 33 mmol) was added in two portions and the reaction was stirred overnight. 100 ml of water was then added and the mixture was extracted with Et_2O (3 x 100 ml) and the combined organic layers were dried on $MgSO_4$ and evaporated. The resulting oil was then dissolved in EtOH (100 ml), 10 ml of conc. HCl were added and the mixture was heated at 60°C overnight. The reaction mixture was concentrated to dryness by rotary evaporation and the resulting solid was washed with Et2O and cold CH_2Cl_2, providing the crude amine hydrochloride salt. The solid was then added to a solution of Et_3N (5.6 ml, 40 mmol) in DCM (70 ml) and stirred for 5 minutes. Phenyl isothiocyanate (2.15 ml, 18 mmol) was then added and the reaction was stirred for 3h at RT. The solvent was removed in vacuo and the crude product was purified by column chromatography (20% EtOAc in hexane) to give the title compound (1.56 g, 30 % yield) as a white solid.

(^{1}H NMR, 400 MHz, $CDCl_3$): δ (ppm) = 7.73 (br s, 1H), 7.38 (d, *J* = 7.5 Hz, 1H), 7.28 (t, *J* = 7.7 Hz, 2H), 7.05-7.22 (m, 4H), 7.01 (d, *J* = 7.5 Hz, 2H), 5.98 (br s, 1H), 5.86 (br s, 1H), 5.43 (br s, 1H), 2.51-2.72 (m, 2H), 2.15-2.28 (m, 2H), 1.70-1.85 (m, 2H), 0.88 (t, *J* = 7.2 Hz, 3H); (^{13}C NMR, 101 MHz, $CDCl_3$): δ (ppm) = 180.0, 136.7, 136.3, 136.0, 133.4, 130.0, 127.7, 127.1, 126.9, 126.8, 125.2, 124.0, 123.0, 56.7, 28.2, 26.7, 23.0, 10.7. IR: 3277, 3057, 2932, 2876, 1598, 1450, 1125, 1004, cm^{-1}; HRMS (ESI) for $C_{20}H_{23}N_2S$ [M+H]$^+$ requires 323.1576 found 323.1577; Mp. 68−70 °C.

1-((2H-Chromen-4-yl)methyl)-3-phenylthiourea (1ha)

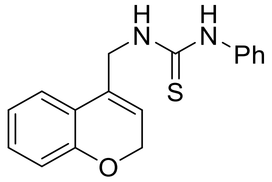

The title compound was prepared following general procedure A using (2H-thiochromen-4-yl)methanamine[5] (540 mg, 3.0 mmol), DCM (30 mL) and phenyl isothiocyanate (0.36 mL, 3.0 mmol). The crude product was purified by precipitation to give the title compound (560 mg, 67 % yield) as a white solid.
(^{1}H NMR, 400 MHz, $CDCl_3$): δ (ppm) = 7.79 (br s, 1H), 7.35 (t, *J* = 7.4 Hz, 2H), 7.26 (t, *J* = 6.6 Hz, 2H), 7.19 (dd, *J* = 7.6, 1.5 Hz, 1H), 7.15 (dt, *J* = 7.8, 1.5 Hz, 1H), 7.10 (d, *J* = 7.6 Hz, 2H), 6.93 (dt, *J* = 7.5, 1.1 Hz, 1H), 6.81 (dd, *J* = 8.1, 1.1 Hz, 1H), 5.98 (br s, 1H), 5.71 (t, *J* = 3.7 Hz, 1H), 4.77-4.72 (m, 2H), 4.70-4.63 (m, 2H); (^{13}C NMR, 101 MHz, $CDCl_3$ + DMSO-d_6 (5% v/v)) δ (ppm) = 181.1, 154.2, 137.9, 130.5, 129.4, 129.3, 125.8, 124.3, 123.5, 121.6, 121.5, 120.2, 116.0, 65.1, 45.7; IR: 3210, 2981, 2888, 2361, 2341, 2002, 1654, 1536, 1075, 966, 814 cm^{-1}; HRMS (ESI) for $C_{17}H_{17}N_2OS$ [M+H]$^+$ requires 297.1056 found 297.1054; Mp. 140-142 °C.

1-((2H-Thiochromen-4-yl)methyl)-3-phenylthiourea (1ia)

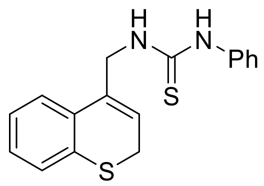

The title compound was prepared following general procedure A using (2H-chromen-4-yl)methanamine[5] (481 mg, 3.0 mmol), DCM (30 mL) and phenyl isothiocyanate (0.36 mL, 3.0 mmol). The crude product was purified by precipitation to give the title compound (674 mg, 72 % yield) as a pale yellow solid.

(^{1}H NMR, 400 MHz, $CDCl_3$): δ (ppm) = 7.85 (br s, 1H), 7.38-7.25 (m, 4H), 7.23-7.12 (m, 3H), 6.98 (d, *J* = 7.6 Hz, 2H), 6.01 (t, *J* = 5.6 Hz, 1H), 5.91 (br s, 1H), 4.71 (d, *J* = 4.8 Hz, 2H), 3.31 (d, *J* = 5.5 Hz, 2H); (^{13}C NMR, 101 MHz, $CDCl_3$): δ (ppm) = 180.0, 135.9, 134.0, 133.3, 131.2, 129.8, 127.8, 127.4, 126.7, 125.7, 124.8, 124.5, 121.5, 48.1, 24.5; IR: 3208, 2981, 2887, 2360, 2342, 1736, 1598, 1473, 1074, 1028, 810 cm^{-1}; HRMS (ESI) for $C_{17}H_{17}N_2S_2$ $[M+H]^+$ requires 313.0828 found 313.0827; Mp. 120-122°C.

1-((1H-Inden-3-yl)methyl)-3-phenylthiourea (1ja)

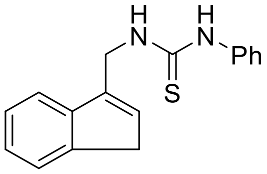

The title compound was prepared following general procedure A using (1H-inden-3-yl)methanamine[5] (435 mg, 3.0 mmol), DCM (30 mL) and phenyl isothiocyanate (0.36 mL, 3.0 mmol). The crude product was purified by precipitation to give the title compound (674 mg, 72 % yield) as a pale yellow solid.

^{1}H NMR, 400 MHz, $CDCl_3$): δ (ppm) = 7.94 (br s, 1H), 7.49-7.35 (m, 4H), 7.35-7.16 (m, 5H), 6.33 (t, *J* = 1.5 Hz, 1H), 6.20 (br s, 1H), 4.90-4.84 (m, 2H), 3.37-3.32 (m, 2H); (^{13}C NMR, 101 MHz, $CDCl_3$): δ (ppm) = 180.7, 143.8, 143.0, 140.1, 137.5, 130.0, 128.9, 125.9, 125.4, 124.7, 124.0, 123.5, 119.0, 42.5, 37.4; IR: 3290, 1599, 1536, 1498, 1454, 1352, 1314, 1050, 1004, 821 cm^{-1}; HRMS (ESI) for $C_{17}H_{17}N_2S$ $[M+H]^+$ requires 281.1118 found 281.1106; Mp. 140-142°C.

1-(2-(3,4-Dihydronaphthalen-1-yl)ethyl)-3-phenylthiourea (1ka)

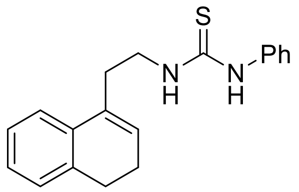

The title compound was prepared following general procedure A using 2-(3,4-dihydronaphthalen-1-yl)ethan-1-amine[6,7] (520 mg, 3.0 mmol), DCM (30 mL) and phenyl isothiocyanate (0.36 mL, 3.0 mmol). The crude product was purified by silica gel column chromatography (eluent: 20 % EtOAc in *n*-pentane) to provide the title compound (646 mg, 70 % yield) as a white solid.

(^{1}H NMR, 400 MHz, $CDCl_3$): δ (ppm) = 7.96 (br s , 1H), 7.32 (t, *J* = 7.6 Hz, 2H), 7.23−7.16 (m, 2H), 7.13−7.02 (m, 5H), 6.03 (br s , 1H), 5.68 (t, *J* = 4.4 Hz, 1H), 3.71 (q, *J* = 6.0 Hz, 2H), 2.66 (t, *J* = 6.2 Hz, 2H), 2.49 (t, *J* = 7.9 Hz, 2H), 2.08−2.02 (m, 2H); (^{13}C NMR, 101 MHz, $CDCl_3$): δ (ppm) = 180.2, 136.6, 135.9, 133.5, 133.4, 130.1, 127.8, 127.7, 127.4, 127.3, 126.6, 125.6, 122.7, 43.6, 31.9, 28.1, 23.0; IR: 3200, 2934, 1531, 1496, 1450, 1314, 1255, 1179, 1109, 910 cm^{-1}; HRMS (ESI) for $C_{19}H_{21}N_2S$ $[M+H]^+$ requires 309.1420 found 309.1417; Mp. 76−78 °C.

[6] N. Noto, K. Miyazawa, T. Koike, M. Akita, *Org. Lett.* **2015**, *17*, 3710−3713.
[7] J. L. Douglas, J. Meunier, *Can. J. Chem.* **1975**, *53*, 3681−3692

1-Phenyl-3-(2-(prop-1-en-2-yl)phenyl)thiourea (1la)

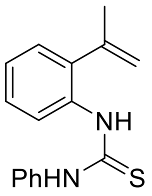

The title compound was prepared following general procedure D using methyl anthranilate (760 mg, 5 mmol), providing the title compound (683 mg, 51% overall yield) as a white solid.

(^{1}H NMR, 400 MHz, $CDCl_3$): δ (ppm) = 8.10 (br s , 1H), 7.99 (br s , 1H), 7.83 (d, *J* = 7.3 Hz, 1H), 7.45−7.26 (m, 8H), 5.19 (s, 1H), 4.93 (s, 1H), 2.05 (s, 3H); (^{13}C NMR, 101 MHz, $CDCl_3$): δ (ppm) = 179.6, 142.7, 139.1, 136.8, 133.7, 129.5, 128.9, 127.9, 127.2, 126.9, 126.3, 125.6, 116.8, 23.7; IR: 3163, 1594, 1530, 1497, 1446, 1352, 1311, 1254, 1221, 1194, 1090, 1028, 907 cm^{-1}; HRMS (ESI) for $C_{16}H_{17}N_2S$ $[M+H]^+$ requires 269.1107 found 269.1106; Mp. 129−130 °C.

1-(5-Fluoro-2-(prop-1-en-2-yl)phenyl)-3-phenylthiourea (1ma)

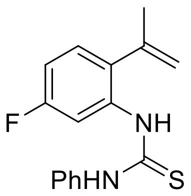

The title compound was prepared following general procedure D using methyl 2-amino-4-fluorobenzoate (845 mg, 5 mmol), providing the title compound (600 mg, 42% overall yield) as a white solid.

(^{1}H NMR, 400 MHz, $CDCl_3$): δ (ppm) = 8.24 (br s , 1H), 7.84 (br s , 1H), 7.76 (dd, J_1 = 5.6 Hz, J_2 = 7.8 Hz, 1H) 7.45 (t, *J* = 7.7 Hz, 2H), 7.34 (t, *J* = 6.6 Hz, 3H), 7.03 (td, *J* = 8.4, 2.9 Hz, 1H), 6.96 (dd, *J* = 9.1, 2.9 Hz, 1H), 5.20 (s, 1H), 4.94 (s, 1H), 2.04 (s, 3H); (^{13}C NMR, 101 MHz, $CDCl_3$): δ (ppm) = 180.0, 160.9 (d, *J* = 248.4 Hz), 141.9, 141.4 (d, *J* = 9.2 Hz), 136.5, 130.0, 129.7, 129.0 (d, *J* = 8.7 Hz), 127.4, 125.7, 117.3, 115.4 (d, *J* = 23.1 Hz), 114.6 (d, *J* = 22.2 Hz), 23.4; (^{19}F NMR, 377 MHz, $CDCl_3$): δ (ppm) = −114.3 (m, 1F); IR: 3179, 2360, 1595, 1525, 1497, 1448, 1420, 1350, 1312, 1259, 1189, 1073, 1028, 958, 910, 884, 819 cm^{-1}; HRMS (ESI) for $C_{16}H_{16}N_2FS$ $[M+H]^+$ requires 287.1013 found 287.1012; Mp. 122−124 °C.

(*Z*)-1-Phenyl-3-m(3-phenylallyl)thiourea (*Z*-1na)

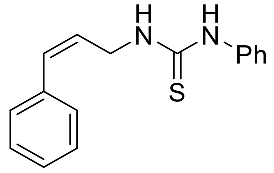

The title compound was prepared following general procedure F using 3-phenyl-2-propyn-1-ol (1.50 mL, 12.0 mmol), THF (60 mL), PPh_3 (3.15 g, 12.0 mmol), phthalimide (1.77 g, 12.0 mmol) and diethyl azodicarboxylate (1.89 mL, 12.0 mmol). The crude product was purified by silica gel column chromatography (eluent: 10 % EtOAc in *n*-pentane) to provide (2-(3-phenylprop-2-yn-1-yl)isoindoline-1,3-dione (1.88 g, 60 % yield) as a white solid.

(^{1}H NMR, 400 MHz, $CDCl_3$): δ (ppm) = 7.83 (dd, *J* = 5.4, 2.9 Hz, 2H), 7.67 (dd, *J* = 5.4, 2.9 Hz, 2H), 7.36−7.33 (m, 2H), 7.23−7.17 (m, 3H), 4.61 (s, 2H); (^{13}C NMR, 101 MHz, $CDCl_3$): δ (ppm) = 167.1, 134.2, 132.1, 131.9, 128.5, 128.2, 123.5, 122.3, 83.0, 82.6, 27.9.

Data consistent with literature values.[8]

[8] Moody, C. J. ; Rahimtoola, K. F.; Porter, B.;. Ross, B. C. *J. Org. Chem.* **1992**, *57*, 2105−2114.

The title compound was prepared following general procedure G using (2-(3-phenylprop-2-yn-1-yl)isoindoline-1,3-dione (783 mg, 3.0 mmol), hydrazine monohydrate (161 µL, 3.3 mmol) and MeOH (25 mL). To the solution of crude 3-phenylprop-2-yn-1-amine (347 mg, 2.65 mmol) in DCM (6 mL) and MeOH (6 mL) was added Et_3N (38 µL, 0.1 mmol) and Lindlar catalyst (44 mg). The resulting suspension was stirred under H_2 balloon for 16 hr then filtered through a pad of Celite®, concentrated *in vacuo* to provide crude (*Z*)-3-phenylprop-2-en-1-amine (350 mg) which was used in the next step without further purification. The crude (*Z*)-3-phenylprop-2-en-1-amine (306 mg, 2.3 mmol) was subjected to the conditions described by general procedure A using phenyl isothiocyanate (0.30 mL, 2.53 mmol) and DCM (23 mL). The crude product was purified by silica gel column chromatography (eluent: 25 % EtOAc in *n*-pentane) to give the title compound (478 mg, 78 % yield) as a white solid.
(^{1}H NMR, 400 MHz, $CDCl_3$): δ (ppm) = 8.18 (brs, 1H), 7.44−7.17 (m, 10H), 6.61 (d, *J* = 11.5 Hz, 1H), 6.09 (br s , 1H), 5.74 (dt, *J* = 11.7, 6.6, 1H), 4.60 (t, *J* = 5.0 Hz, 2H); (^{13}C NMR, 101 MHz, $CDCl_3$): δ (ppm) = 180.4, 136.0, 135.9, 132.2, 130.2, 128.7, 128.3, 127.4, 127.3, 126.9, 125.2, 43.6; IR: 3205, 1596, 1527, 1494, 1448, 1314, 1238, 1190, 1111, 1075, 1028, 915 cm^{-1}; HRMS (ESI) for $C_{16}H_{17}N_2S$ $[M+H]^+$ requires 269.1107 found 269.1109; Mp. 87−88 °C.

1-Cinnamyl-3-phenylthiourea (*E*-1na)

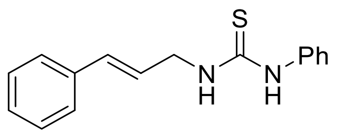

The title compound was prepared following general procedure A using (*E*)-3-phenylprop-2-en-1-amine[8] (666 mg, 5.0 mmol), DCM (50 mL) and phenyl isothiocyanate (0.60 mL, 5.0 mmol). The crude product was purified by precipitation to give the title compound (1.29 g, 96 % yield) as a white solid.
(^{1}H NMR, 400 MHz, $CDCl_3$): δ (ppm) = 8.33 (br s , 1H), 7.48−7.44 (m, 2H), 7.39−7.25 (m, 8H), 6.55 (d, *J* = 15.9 Hz, 1H), 6.27 (dt, *J* = 15.9, 6.4 Hz, 1H), 6.20 (br s , 1H), 4.49 (t, *J* = 5.4 Hz, 2H); (^{13}C NMR, 101 MHz, $CDCl_3$): δ (ppm) = 180.5, 136.2, 135.9, 132.9, 130.2, 128.5, 127.8, 127.3, 126.4, 125.3, 124.3, 47.6; IR: 3211, 3027, 2055, 1596, 1531, 1495, 1450, 1347, 1314, 1260, 1185, 1073, 1028, 966, 910 cm^{-1}; HRMS (ESI) for $C_{16}H_{17}N_2S$ $[M+H]^+$ requires 269.1107 found 269.1110; Mp. 96−97 °C.

(*E*)-1-(3-(4-Methoxyphenyl)allyl)-3-phenylthiourea (1oa)

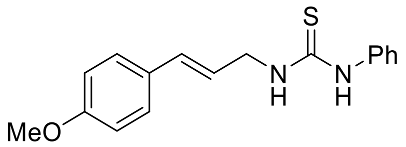

(*E*)-3-(4-Methoxyphenyl)prop-2-en-1-ol was prepared following general procedure E using (*E*)-3-(4-methoxyphenyl)acrylaldehyde (813 mg, 5.0 mmol), $NaBH_4$ (126 mg, 3.3 mmol) and (MeOH 10 mL). The crude alcohol was then subjected to conditions described in general procedure F using THF (25 mL), PPh_3 (1.31 g, 5.0 mmol), phthalimide (736 mg, 5.0 mmol) and diethyl azodicarboxylate (0.79 mL, 5.0 mmol). The crude product was purified by silica gel column chromatography (eluent: 10 % EtOAc in *n*-pentane) to provide (*E*)-2-(3-(4-methoxyphenyl)allyl)isoindoline-1,3-dione (340 mg, 23 % yield) as a white solid.
(^1H NMR, 400 MHz, $CDCl_3$): δ (ppm) = 7.86 (dd, *J* = 5.4, 2.9 Hz, 2H), 7.72 (dd, *J* = 5.4, 3.2 Hz, 2H), 7.30 (d, *J* = 8.8 Hz, 2H), 6.83 (d, *J* = 8.8 Hz, 2H), 6.62 (d, *J* = 15.7 Hz, 1H), 6.13 (dt, *J* = 15.7, 6.6 Hz, 1H), 4.43 (dd, J_1 = 1.2 Hz, J_2 = 6.6 Hz, 2H), 3.79 (s, 3H); (^{13}C NMR, 101 MHz, $CDCl_3$): δ (ppm) = 168.0, 159.4, 133.9, 133.3, 132.2, 129.0, 127.7, 123.2, 120.4, 113.9, 55.2, 39.7.
Data consistent with literature values.[9]

The title compound was prepared following general procedure G using (*E*)-2-(3-(4-methoxyphenyl)allyl)isoindoline-1,3-dione (323 mg, 1.1 mmol), hydrazine monohydrate (80 µL, 1.65 mmol) and MeOH (10 mL). The crude amine was subjected to the conditions described by general procedure A using phenyl isothiocyanate (0.116 mL, 0.97 mmol) and DCM (9 mL). The crude product was purified by precipitation to give the title compound (253 mg, 77 % yield) as a white solid.
(^1H NMR, 400 MHz, $CDCl_3$): δ (ppm) = 8.13 (br s , 1H), 7.45 (t, *J* = 7.8 Hz, 2H), 7.34−7.25 (m, 5H), 6.86 (d, *J* = 8.8 Hz, 2H), 6.49 (d, *J* = 15.9 Hz, 1H), 6.13 (dt, *J* = 15.7, 6.5 Hz, 1H), 6.13 (br s , 1H), 4.45 (t, *J* = 5.6 Hz, 2H), 3.82 (s, 3H); (^{13}C NMR, 101 MHz, $CDCl_3$): δ (ppm) = 180.4, 159.4, 135.9, 132.6, 130.2, 128.9, 127.6, 127.4, 125.3, 121.9, 114.0, 55.2, 47.8; IR: 3205, 2981, 2361, 1606, 1529, 1509, 1452, 1420, 1298, 1246, 1175, 1031, 967, 840 cm^{-1}; HRMS (ESI) for $C_{17}H_{19}ON_2S$ $[M+H]^+$ requires 299.1213 found 299.1212; Mp. 130−132 °C.

[9] Z. Jiang, L. Zhang, C. Dong, B. Ma, W. Tang, L. Xua, Q. Fan, J. Xiao, *Tetrahedron* **2012**, *68*, 4919–4926.

(*E*)-1-Phenyl-3-(3-(*p*-tolyl)allyl)thiourea (1pa)

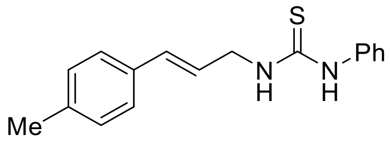

(*E*)-3-(*p*-Tolyl)prop-2-en-1-ol was prepared following general procedure E using (*E*)-3-(*p*-tolyl)acrylaldehyde (1.462 g, 10.0 mmol), $NaBH_4$ (252 mg, 6.7 mmol) and (MeOH 20 mL). The crude alcohol (815 mg, 5.5 mmol) was then dissolved in THF (30 mL) and to the solution was added PPh_3 (1.59 g, 6.05 mmol), phthalimide (890 mg, 6.05 mmol) and diethyl azodicarboxylate (0.95 mL, 6.05 mmol). After stirring for 2 hours at room temperature, hydrazine monohydrate (0.8 mL, 16.5 mmol) was added and the reaction mixture was stirred at room temperature for 16 hours. The mixture was diluted with H_2O (15 mL) and conc. HCl (3 mL) was added. After stirring for 30 minutes, the suspension was filtered and the filtrate was washed with EtOAc (x2), basified to pH = 10 using 5 M NaOH and extracted with Et_2O (x2). Combined organic layers were washed with brine, dried over $MgSO_4$ and concentrated *in vacuo*. The crude amine (589 mg, 4.0 mmol) was subjected to the conditions described by general procedure A using phenyl isothiocyanate (0.525 mL, 4.4 mmol) and DCM (40 mL). The crude product was purified by precipitation to give the title compound (819 mg, 72 % yield) as a yellow solid.

(^1H NMR, 400 MHz, $CDCl_3$): δ (ppm) = 8.11 (br s , 1H), 7.45 (t, *J* = 7.8 Hz, 2H), 7.34−7.25 (m, 5H), 7.14 (d, *J* = 7.8 Hz, 2H), 6.51 (d, *J* = 15.7 Hz, 1H), 6.20 (dt, *J* = 15.7, 6.5 Hz, 1H), 6.14 (br s , 1H), 4.47 (t, *J* = 5.6 Hz, 2H), 2.35 (s, 3H); (^{13}C NMR, 101 MHz, $CDCl_3$): δ (ppm) = 180.5, 137.8, 135.9, 133.4, 132.9, 130.2, 129.3, 127.4, 126.3, 125.3, 123.2, 47.8, 21.2; IR: 3120, 3024, 2360, 1595, 1529, 1496, 1451, 1345, 1313, 1260, 1183, 1119, 968 cm^{-1}; HRMS (ESI) for $C_{17}H_{19}N_2S$ $[M+H]^+$ requires 283.1264 found 283.1266; Mp. 147−149 °C.

(*E*)-1-(3-(4-Chlorophenyl)allyl)-3-phenylthiourea (1qa)

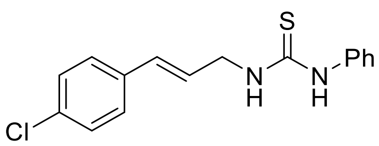

(*E*)-3-(4-Chlorophenyl)prop-2-en-1-ol was prepared following general procedure E using (*E*)-3-(4-chlorophenyl)acrylaldehyde (1.00 g, 6.0 mmol), $NaBH_4$ (151 mg, 4.0 mmol) and (MeOH 10 mL). The crude alcohol (742 mg, 4.4 mmol) was then dissolved in THF (25 mL) and to the solution was added PPh_3 (1.27 g, 4.84 mmol), phthalimide (712 mg, 4.84 mmol) and diethyl azodicarboxylate (0.76 mL, 4.84 mmol). After stirring for 2 hours at room temperature,

hydrazine monohydrate (0.64 mL, 13.2 mmol) was added and the reaction mixture was stirred at room temperature for 16 hours. The mixture was diluted with H_2O (15 mL) and conc. HCl (3 mL) was added. After stirring for 30 minutes, the suspension was filtered and the filtrate was washed with EtOAc (x2), basified to pH = 10 using 5 M NaOH and extracted with Et_2O (x2). Combined organic layers were washed with brine, dried over $MgSO_4$ and concentrated *in vacuo*. The crude amine (642 mg, 3.83 mmol) was subjected to the conditions described by general procedure A using phenyl isothiocyanate (0.50 mL, 4.21 mmol) and DCM (38 mL). The crude product was purified by precipitation to give the title compound (981 mg, 85 % yield) as a pale yellow solid.

(^{1}H NMR, 400 MHz, $CDCl_3$): δ (ppm) = 8.30 (br s , 1H), 7.45 (t, *J* = 7.8 Hz, 2H), 7.34−7.25 (m, 7H), 6.48 (d, *J* = 15.9 Hz, 1H), 6.24 (dt, *J* = 15.9, 6.4 Hz, 1H), 6.20 (br s , 1H), 4.47 (t, *J* = 5.5 Hz, 2H); (^{13}C NMR, 101 MHz, $CDCl_3$): δ (ppm) = 180.6, 135.9, 134.7, 133.4, 131.5, 130.2, 128.7, 127.6, 127.4, 125.3, 125.2, 47.4; IR: ν 3214, 2981, 2360, 1715, 1594, 1531, 1492, 1451, 1380, 1345, 1313, 1258, 1233, 1184, 1091, 1012, 967, 844 cm^{-1}; HRMS (ESI) for $C_{16}H_{16}N_2ClS$ $[M+H]^+$ requires 303.0717 found 303.0720; Mp. 121−123 °C.

1-Cinnamyl-3-(*p*-tolyl)thiourea (1nb)

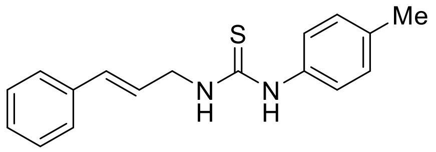

The title compound was prepared following general procedure A using (*E*)-3-phenylprop-2-en-1-amine (533 mg, 4.0 mmol), DCM (30 mL) and *p*-tolyl isothiocyanate (657 mg, 4.4 mmol). The crude product was purified by precipitation to give the title compound (838 mg, 74 % yield) as a white solid.

(^{1}H NMR, 400 MHz, $CDCl_3$): δ (ppm) = 8.09 (br s, 1H), 7.38−7.24 (m, 7H), 7.15 (d, *J* = 8.3 Hz, 2H), 6.53 (d, *J* = 15.9 Hz, 1H), 6.25 (dt, *J* = 15.9, 6.4 Hz, 1H), 6.08 (br s , 1H), 4.47 (t, *J* = 5.5 Hz, 2H), 2.38 (s, 3H); (^{13}C NMR, 101 MHz, $CDCl_3$): δ (ppm) = 180.7, 137.7, 136.2, 133.1, 132.8, 130.8, 128.5, 127.8, 126.4, 125.6, 124.5, 47.6, 21.0; IR: 3198, 3026, 1510, 1449, 1311, 1259, 1188, 966, 910, 819 cm^{-1}; HRMS (ESI) for $C_{17}H_{19}N_2S$ $[M+H]^+$ requires 283.1264 found 283.1268; Mp. 117−118 °C.

1-Cinnamyl-3-(4-methoxyphenyl)thiourea (1nc)

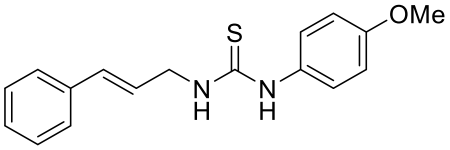

The title compound was prepared following general procedure A using (*E*)-3-phenylprop-2-en-1-amine (533 mg, 4.0 mmol), DCM (30 mL) and 4-Methoxyphenyl isothiocyanate (727 mg, 4.4 mmol). The crude product was purified by precipitation to give the title compound (1.05 g, 88 % yield) as a white solid.

(^{1}H NMR, 400 MHz, $CDCl_3$): δ (ppm) = 8.06 (br s , 1H), 7.37−7.24 (m, 5H), 7.20 (d, *J* = 8.8 Hz, 2H), 6.95 (d, *J* = 8.8 Hz, 2H), 6.52 (d, *J* = 15.9 Hz, 1H), 6.24 (dt, *J* = 15.9, 6.5 Hz, 1H), 5.96 (br s , 1H), 4.46 (t, *J* = 5.6 Hz, 2H), 3.82 (s, 3H); (^{13}C NMR, 101 MHz, $CDCl_3$): δ (ppm) = 181.1, 159.0, 136.2, 132.7, 128.5, 128.2, 127.8, 127.8, 126.4, 124.5, 115.3, 55.5, 47.5; IR: 3190, 1507, 1292, 1240, 1166, 1105, 1030, 966, 910, 830 cm^{-1}; HRMS (ESI) for $C_{17}H_{19}ON_2S$ $[M+H]^+$ requires 299.1213 found 299.1213; Mp. 97−98 °C.

1-Cinnamyl-3-(4-(trifluoromethyl)phenyl)thiourea (1nd)

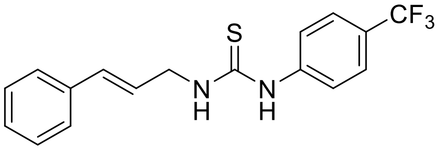

The title compound was prepared following general procedure A using (*E*)-3-phenylprop-2-en-1-amine (533 mg, 4.0 mmol), DCM (30 mL) and 4-(trifluoromethyl)phenyl isothiocyanate (894 mg, 4.4 mmol). The crude product was purified by precipitation to give the title compound (952 mg, 71 % yield) as a white solid.

(^{1}H NMR, 400 MHz, $CDCl_3$): δ = 8.65 (br s , 1H), 7.68 (d, *J* = 8.3 Hz, 2H), 7.41−7.26 (m, 7H), 6.60 (d, *J* = 15.7 Hz, 1H), 6.35 (br s , 1H), 6.28 (dt, *J* = 15.9, 6.5 Hz, 1H), 4.48 (t, *J* = 4.9 Hz, 2H); (^{13}C NMR, 101 MHz, $CDCl_3$): δ (ppm) = 180.3, 139.6, 136.0, 133.6, 128.6, 128.3, 128.1, 127.3, 126.4, 124.2, 123.7, 123.6 (q, *J* = 271.8 Hz), 47.7; (^{19}F NMR, 377 MHz, $CDCl_3$): δ (ppm) = −62.5 (s, 3F); IR: 3267, 1619, 1552, 1333, 1162, 1115, 1071, 1015, 967, 840 cm^{-1}; HRMS (ESI) for $C_{17}H_{16}N_2F_3S$ $[M+H]^+$ requires 337.0981 found 337.0981; Mp. 123−124 °C.

1-Cinnamyl-3-(4-fluorophenyl)thiourea (1ne)

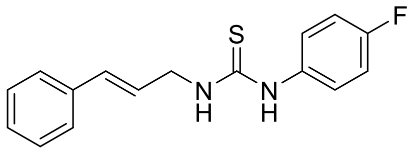

The title compound was prepared following general procedure A using (*E*)-3-phenylprop-2-en-1-amine (533 mg, 4.0 mmol), DCM (30 mL) and 4-fluorophenyl isothiocyanate (674 mg, 4.4 mmol). The crude product was purified by silica gel column chromatography (eluent: 20 % EtOAc in *n*-pentane) to provide the title compound (879 mg, 77 % yield) as a white solid.

(^{1}H NMR, 400 MHz, $CDCl_3$): δ (ppm) = 8.43 (br s, 1H), 7.37−7.31 (m, 4H), 7.29−7.25 (m, 3H), 7.13 (t, *J* = 8.6 Hz, 2H), 6.54 (d, *J* = 15.9 Hz, 1H), 6.25 (dt, *J* = 15.8, 6.5 Hz, 1H), 6.04 (br s, 1H), 4.46 (t, *J* = 5.6 Hz, 2H); (^{13}C NMR, 101 MHz, $CDCl_3$): δ (ppm) = 180.8, 161.4 (d, *J* = 248.0 Hz), 136.1, 133.0, 131.9, 128.6, 127.9 (d, *J* = 7.2 Hz), 127.7, 126.4, 124.2, 117.0 (d, *J* = 23.1 Hz), 47.5; (^{19}F NMR, 377 MHz, $CDCl_3$): δ (ppm) = −113.0 (br s, 1F); IR: 3216, 1529, 1504, 1340, 1214, 1153, 966, 910, 834 cm^{-1}; HRMS (ESI) for $C_{16}H_{16}N_2FS$ [M+H]$^+$ requires 287.1013 found 287.1015; Mp. 90−91 °C.

1-(2-(Cyclohex-1-en-1-yl)ethyl)-3-phenylthiourea (1ra)

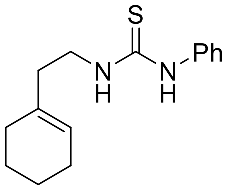

The title compound was prepared following general procedure A using 2-(cyclohex-1-en-1-yl)ethan-1-amine (372 mg, 3.0 mmol), DCM (30 mL) and phenyl isothiocyanate (0.36 mL, 3.0 mmol). The crude product was purified by precipitation to give the title compound (670 mg, 89 % yield) as a white solid.

(^{1}H NMR, 400 MHz, $CDCl_3$): δ (ppm) = 7.78 (br s, 1H), 7.41 (t, *J* = 7.5 Hz, 2H), 7.29 (t, *J* = 7.5 Hz, 1H), 7.17 (t, *J* = 7.5 Hz, 2H), 6.24 (br s, 1H), 5.33-5.25 (m, 1H), 3.70-3.62 (m, 2H), 2.27 (t, J = 6.3 Hz, 2H), 2.06-1.89 (m, 4H), 1.68-1.48 (m, 4H), 1.36 (s, 9H); (^{13}C NMR, 101 MHz, $CDCl_3$): δ (ppm) = 179.8, 136.0, 134.4, 129.8, 126.8, 125.0, 124.5, 42.3, 36.7, 27.1, 25.0, 22.5, 22.0; IR: 3170, 3019, 2923, 2852, 1594, 1522, 1495, 1396, 1249, 1156, 906 cm^{-1}; HRMS (ESI) for $C_{15}H_{21}N_2S$ [M+H]$^+$ requires 261.1420 found 261.1424; Mp. = 100−102 °C.

1-(2-(cyclohex-1-en-1-yl)ethyl)-3-(4-methoxybenzyl)thiourea (1rb)

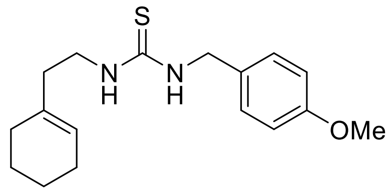

The title compound was prepared following general procedure A using 2-(cyclohex-1-en-1-yl)ethan-1-amine (372 mg, 3.0 mmol), DCM (30 mL) and *p*-methoxybenzyl isothiocyanate (0.47 mL, 3.0 mmol). The crude product was purified by silica gel column chromatography (eluent: 30 % Et_2O in *n*-pentane) to provide the title compound (740 mg, 81 % yield) as a thick oil.

(^{1}H NMR, 400 MHz, $CDCl_3$): δ (ppm) = 7.22 (d, *J* = 8.4 Hz, 2H), 6.86 (d, *J* = 8.4 Hz, 2H), 6.16 (br s, 1H), 5.72 (br s, 1H), 5.98 (br s, 1H), 5.62 (br s, 1H), 5.46-5.34 (m, 1H), 4.50 (s, 2H), 3.78 (s, 3H), 2.15 (t, *J* = 5.8 Hz, 2H), 197-1.80 (m, 4H), 1.61-1.50 (m, 4H); (^{13}C NMR, 101 MHz, $CDCl_3$): δ (ppm) = 181.2, 159.1, 134.0, 128.6, 124.1, 114.1 (2C), 55.2, 47.6, 42.0, 36.8, 27.5, 25.0, 22.5, 22.1; IR: 3247, 2926, 2884, 1611, 1544, 1511, 1245, 1174, 1084, 818 cm^{-1}; HRMS (ESI) for $C_{17}H_{25}N_2OS$ $[M+H]^+$ requires 305.1682 found 305.1679.

1-(2-Allyl-4-fluorophenyl)-3-phenylthiourea (1sa)

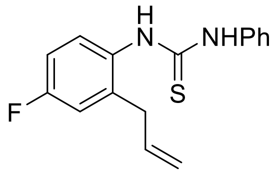

2-allyl-4-fluoroaniline was prepared following a modified literature procedure.[10]

A round-bottomed flask equipped with a reflux condenser was charged with *N*-allyl-4-fluoroaniline (1.21 g, 8.0 mmol) and BF_3•OEt_2 (1.48 mL, 12.0 mmol). The reaction mixture was heated at 140 °C for 12 hours, then treated with a sat. $Na_2CO_{3(aq)}$ and extracted with DCM (x2). Combined organic layers were washed with brine, dried over $MgSO_4$ and concentrated *in vacuo*. The crude product was purified by silica gel column chromatography (eluent: 10 % Et_2O in *n*-pentane) to provide the title compound (654 mg, 54 % yield) as a yellow oil.

(^{1}H NMR, 400 MHz, $CDCl_3$): δ (ppm) = 6.82−6.76 (m, 2H), 6.64−6.60 (m, 1H), 5.99−5.89 (m, 1H), 5.19−5.09 (m, 2H), 3.53 (br s , 2H), 3.29 (dt, *J* = 6.1, 1.6 Hz, 2H); (^{13}C NMR, 101 MHz, $CDCl_3$): δ (ppm) = 156.4 (d, *J* = 235.2 Hz), 140.7 (d, *J* = 1.6 Hz), 135.0, 125.6 (d, *J* = 6.4 Hz), 116.6, 116.5 (d, *J* = 8.0 Hz), 116.3 (d, *J* = 23.1 Hz), 36.1.

Data consistent with literature values.[10]

The title compound was prepared following general procedure A using 2-allyl-4-fluoroaniline (605 mg, 4.0 mmol), DCM (30 mL) and phenyl isothiocyanate (0.53 mL, 4.4 mmol). The crude product was purified by precipitation to give the title compound (853 mg, 74 % yield) as a white solid.

(^{1}H NMR, 400 MHz, $CDCl_3$): δ (ppm)= 8.18 (br s , 1H), 7.68 (br s , 1H), 7.46−7.40 (m, 3H), 7.35 (d, J = 7.3 Hz, 2H), 7.30 (t, J = 7.2 Hz, 1H), 7.02−6.97 (m, 2H), 5.87−5.77 (m, 1H), 4.99 (d, J = 10.0 Hz, 1H), 4.89 (d, J = 17.1 Hz, 1H), 3.35 (d, J = 6.1 Hz, 2H); (^{13}C NMR, 101 MHz, $CDCl_3$): δ = 180.6, 161.7 (d, J = 248.0 Hz), 138.9, 136.7, 135.0, 131.7, 130.2 (d, J = 8.7 Hz), 129.7, 127.3, 125.5, 117.2, 117.0, 114.3 (d, J = 23.1 Hz), 36.2; (^{19}F NMR, 377 MHz, $CDCl_3$): δ (ppm) = −113.1 (br s , 1F); IR: 3192, 2980, 2361, 1593, 1524, 1496, 1448, 1349, 1314, 1263, 1219, 1147, 998, 967, 922, 867 cm^{-1}; HRMS (ESI) for $C_{16}H_{16}N_2FS$ $[M+H]^+$ requires 287.1013 found 287.1010; Mp. 129−130 °C.

[10] W. R. Martínez, G. C. G. Militão, T. G. da Silva, R. O. Silva, P. H. Menezes, *RSC Adv.* **2014**, *4*, 14715−14718.

General procedure H *(Trifluoromethylation-cyclisation with Togni II reagent)*
Into a vial containing appropriate thiourea (0.3 mmol), 1-trifluoromethyl-1,2-benziodoxol-3-(1H)-one (113.8 mg, 0.36 mmol), 4Å MS (30 mg) and a stirrer bar was added a solution of trifluoroacetic acid (45.9 µL, 0.6 mmol) in $CDCl_3$ (3.0 mL) under Ar atmosphere. The vial was sealed and allowed to stir at room temperature for 24 hours. The reaction mixture was then concentrated and purified by silica gel column chromatography.

_N_-Phenyl-5-(2,2,2-trifluoroethyl)-4,5-dihydrothiazol-2-amine (2aa)

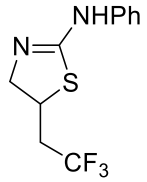

The title compound was prepared following general procedure H using 1-allyl-3-phenylthiourea (57.7 mg, 0.3 mmol), 1-trifluoromethyl-1,2-benziodoxol-3-(1H)-one (113.8 mg, 0.36 mmol), 4Å MS (30 mg), trifluoroacetic acid (45.9 µL, 0.6 mmol) and $CDCl_3$ (3.0 mL). The crude product was purified by silica gel column chromatography (eluent: 1% Et_3N and 25 % EtOAc in *n*-pentane) to provide the title compound (62.8 mg, 80 % yield) as a white solid.

(^{1}H NMR, 400 MHz, $CDCl_3$): δ (ppm) = 7.30 (t, J = 7.8 Hz, 2H), 7.14 (d, J = 7.6 Hz, 2H), 7.08 (t, J = 7.3 Hz, 1H), 6.66 (br s, 1H), 4.04−3.95 (m, 2H), 3.69−3.64 (m, 1H), 2.64−2.48 (m, 2H); (^{13}C NMR, 101 MHz, $CDCl_3$): δ (ppm) = 159.9, 146.2, 129.0, 125.5 (q, J = 277.4 Hz), 123.5, 120.9, 56.7, 41.9, 38.9 (q, J = 27.8 Hz); (^{19}F NMR, 377 MHz, $CDCl_3$): δ (ppm) = −64.9 (t, J = 10.4 Hz, 3F); IR: 3246, 3194, 3128, 3008, 2979, 2861, 2362, 1591, 1557, 1497, 1446, 1377, 1331, 1265, 1244, 1191, 1174, 1154, 1141, 1096, 1072, 1036, 982, 904, 839 cm^{-1}; HRMS (ESI) for $C_{11}H_{12}N_2F_3S$ [M+H]$^+$ requires 261.0668 found 261.0667; Mp. 129−130 °C.

5-Methyl-*N*-phenyl-5-(2,2,2-trifluoroethyl)-4,5-dihydrothiazol-2-amine (2ba)

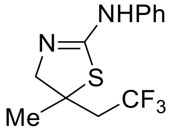

The title compound was prepared following general procedure H using 1-(2-methylallyl)-3-phenylthiourea (61.9 mg, 0.3 mmol), 1-trifluoromethyl-1,2-benziodoxol-3-(1H)-one (113.8 mg, 0.36 mmol), 4Å MS (30 mg), trifluoroacetic acid (45.9 µL, 0.6 mmol) and $CDCl_3$ (3.0 mL). The crude product was purified by silica gel column chromatography (eluent: 30 % EtOAc in *n*-pentane) to provide the title compound (78.7 mg, 96 % yield) as a white solid.
(^{1}H NMR, 400 MHz, $CDCl_3$): δ (ppm) = 7.23−7.18 (m, 2H), 7.02−6.95 (m, 3H), 3.60 (d, A of AB J_{AB} = 11.4 Hz, 1H), 3.50 (d, B of AB J_{AB} = 11.4 Hz, 1H), 2.65−2.52 (m, 2H), 1.58 (s, 3H); (^{13}C NMR, 101 MHz, $CDCl_3$): δ (ppm) = 160.4, 147.0, 129.0, 125.4 (q, J = 279.0 Hz), 123.4, 121.1, 61.9, 53.8, 43.6 (q, J = 27.8 Hz), 25.5; (^{19}F NMR, 377 MHz, $CDCl_3$): δ (ppm) = −61.1 (t, J = 11.3 Hz, 3F); IR: 3032, 2858, 2360, 1639, 1590, 1492, 1445, 1390, 1370, 1312, 1257, 1173, 1148, 1106, 1089, 1053, 975, 902, 858 cm^{-1}; HRMS (ESI) for $C_{12}H_{14}N_2F_3S$ [M+H]$^+$ requires 275.0824 found 275.0822; Mp. 118−119 °C.

***N*,5-Diphenyl-5-(2,2,2-trifluoroethyl)-4,5-dihydrothiazol-2-amine (2ca)**

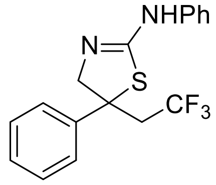

The title compound was prepared following general procedure H using 1-phenyl-3-(2-phenylallyl)thiourea (80.5 mg, 0.3 mmol), 1-trifluoromethyl-1,2-benziodoxol-3-(1H)-one (113.8 mg, 0.36 mmol), 4Å MS (30 mg), trifluoroacetic acid (45.9 µL, 0.6 mmol) and $CDCl_3$

(3.0 mL). The crude product was purified by silica gel column chromatography (eluent: 1% Et_3N and 10 % EtOAc in *n*-pentane) to provide the title compound (90.8 mg, 90 % yield) as a white solid.

(^{1}H NMR, 400 MHz, $CDCl_3$): δ (ppm) = 7.40−7.30 (m, 7H), 7.15−7.09 (m, 3H), 4.16 (dd, J_1 = 11.7 Hz, J_2 = 15.9 Hz, 2H), 3.20−3.00 (m, 2H); (^{13}C NMR, 101 MHz, $CDCl_3$): δ = 160.0, 146.5, 139.6, 129.1, 128.6, 128.0, 126.3, 124.9 (q, J = 279.8 Hz), 123.6, 121.1, 60.8, 60.2, 44.9 (q, J = 26.8 Hz); (^{19}F NMR, 377 MHz, $CDCl_3$): δ (ppm) = −60.4 (t, J = 10.4 Hz, 3F); IR: ν 3030, 2865, 1639, 1590, 1556, 1494, 1446, 1362, 1322, 1256, 1256, 1218, 1183, 1126, 1081, 1037, 995, 857; HRMS (ESI) for $C_{17}H_{16}N_2F_3S$ [M+H]$^+$ requires 337.0981 found 337.0975; Mp. 82−84 °C.

5-(4-Fluorophenyl)-N-phenyl-5-(2,2,2-trifluoroethyl)-4,5-dihydrothiazol-2-amine (2da)

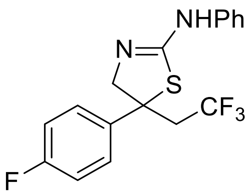

The title compound was prepared following general procedure H using 1-(2-(4-fluorophenyl)allyl)-3-phenylthiourea (86 mg, 0.3 mmol), 1-trifluoromethyl-1,2-benziodoxol-3-(1H)-one (113.8 mg, 0.36 mmol), 4Å MS (30 mg), trifluoroacetic acid (45.9 µL, 0.6 mmol) and $CDCl_3$ (3.0 mL). The crude product was purified by silica gel column chromatography (eluent: 10% Et_2O in DCM) to provide the title compound (94 mg, 88 % yield) as a thick colourless oil.

(^{1}H NMR, 400 MHz, $CDCl_3$): δ (ppm) = 7.26−7.18 (m, 4H), 7.09−6.91 (m, 5H), 4.07 (d, A of AB J_{AB} = 12.1 Hz, 1H), 4.03 (d, B of AB J_{AB} = 12.1 Hz, 1H), 3.10−2.85 (m, 2H); (^{13}C NMR, 101 MHz, $CDCl_3$): δ = 162.2 (d, J = 248.2 Hz), 159.4, 145.9, 135.6 (d, J = 3.4 Hz), 129.1, 128.2 (d, J = 8.2 Hz), 124.9 (q, J = 279.8 Hz), 123.7, 120.9, 115.5 (d, J = 21.8 Hz), 61.3, 60.5, 44.9 (q, J = 27.0 Hz); (^{19}F NMR, 377 MHz, $CDCl_3$): δ (ppm) = −60.5 (t, J = 10.4 Hz, 3F), -113.9 (m, 1F); IR: ν 3001, 2964, 1641, 1500, 1489, 1436, 1352, 1215, 1163, 1146, 1081; HRMS (ESI) for $C_{17}H_{15}N_2F_4S$ [M+H]$^+$ requires 355.0887 found 355.0881.

5-(4-Chlorophenyl)-N-phenyl-5-(2,2,2-trifluoroethyl)-4,5-dihydrothiazol-2-amine (2ea)

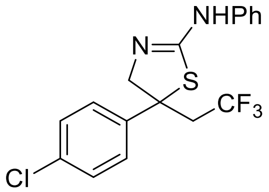

The title compound was prepared following general procedure H using 1-(2-(4-chlorophenyl)allyl)-3-phenylthiourea (90 mg, 0.3 mmol), 1-trifluoromethyl-1,2-benziodoxol-3-(1H)-one (113.8 mg, 0.36 mmol), 4Å MS (30 mg), trifluoroacetic acid (45.9 µL, 0.6 mmol) and $CDCl_3$ (3.0 mL). The crude product was purified by silica gel column chromatography (eluent: 10% Et_2O in DCM) to provide the title compound (102 mg, 90 % yield) as a thick colourless oil.

(^{1}H NMR, 400 MHz, $CDCl_3$): δ (ppm) = 7.26−7.13 (m, 6H), 7.06−6.93 (m, 3H), 4.03 (d, A of AB J_{AB} = 11.7 Hz, 1H), 3.97 (d, B of AB J_{AB} = 11.7 Hz, 1H), 3.23−2.85 (m, 2H); (^{13}C NMR, 101 MHz, $CDCl_3$): δ (ppm) = 159.5, 145.9, 138.4, 133.9, 129.1, 128.7, 127.8, 126.1, 124.9 (q, J = 279.8 Hz), 123.7, 61.2, 60.5, 44.7 (q, J = 26.8 Hz); (^{19}F NMR, 377 MHz, $CDCl_3$): δ (ppm) = −60.4 (t, J = 10.4 Hz, 3F); IR: ν 2987, 2831, 1669, 1595, 1546, 1454, 1266, 1382, 1356, 1216, , 1146, 1080, 921.

***N-P*henyl-5-(2,2,2-trifluoroethyl)-5-(4-(trifluoromethyl)phenyl)-4,5-dihydrothiazol-2-amine (2fa)**

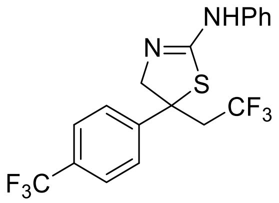

The title compound was prepared following general procedure H using 1-phenyl-3-(2-(4-(trifluoromethyl)phenyl)allyl)thiourea (101 mg, 0.3 mmol), 1-trifluoromethyl-1,2-benziodoxol-3-(1H)-one (113.8 mg, 0.36 mmol), 4Å MS (30 mg), trifluoroacetic acid (45.9 µL, 0.6 mmol) and $CDCl_3$ (3.0 mL). The crude product was purified by silica gel column chromatography (eluent: 10% Et_2O in DCM) to provide the title compound (109 mg, 90 % yield) as a thick colourless oil.

(^{1}H NMR, 400 MHz, $CDCl_3$): δ (ppm) = 7.53 (d, J = 8.2 Hz, 2H), 7.40 (d, J = 8.2 Hz, 2H), 7.22 (t, J = 8.2 Hz , 2H), 7.06 (d, J = 8.2 Hz , 2H), 7.00 (d, J = 7.9 Hz, 2H), 4.12 (d, A of AB J_{AB} = 12.1 Hz, 1H), 4.06 (d, B of AB J_{AB} = 12.1 Hz, 1H), 3.14-2.89 (m, 2H); (^{13}C NMR, 101 MHz, $CDCl_3$): δ = (^{13}C NMR, 101 MHz, $CDCl_3$): δ (ppm) = 158.9, 145.3, 143.9, 130.3 (q, J = 32.1 Hz), 129.2, 126.9, 125.6 (q, J = 3.5 Hz), 124.7 (q, J = 279.1 Hz), 123.9, 123.8 (q, J = 272.5 Hz), 120.8, 62.2,

61.1, 44.7 (q, J = 26.8 Hz); (^{19}F NMR, 377 MHz, $CDCl_3$): δ (ppm) = −60.4 (t, J = 10.4 Hz, 3F), -62.7 (s, 3F); IR: ν 3016, 2978, 1631, 1580, 1534, 1484, 1456, 1362, 1300, 1278, 1249, 1225, 1091, 1057, 986; HRMS (ESI) for $C_{18}H_{15}N_2F_6S$ $[M+H]^+$ requires 405.0855 found 405.0854.

(1*S,2*S**)-*N*-Phenyl-2-(trifluoromethyl)-3,4-dihydro-2H,4'H-spiro[naphthalene-1,5'-thiazol]-2'-amine (2ga)**

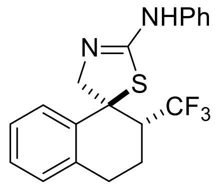

The title compound was prepared following general procedure H using 1-((3,4-dihydronaphthalen-1-yl)methyl)-3-phenylthiourea (88.2 mg, 0.3 mmol), 1-trifluoromethyl-1,2-benziodoxol-3-(1H)-one (113.8 mg, 0.36 mmol), 4Å MS (30 mg), trifluoroacetic acid (45.9 µL, 0.6 mmol) and $CDCl_3$ (3.0 mL). The crude product was purified by silica gel column chromatography (eluent: 1% Et_3N and 15 % EtOAc in *n*-pentane) to provide the title compound (90.5 mg, 83 % yield) as a white solid.

(^{1}H NMR, 400 MHz, $CDCl_3$): δ (ppm) = 7.66−7.62 (m, 1H), 7.20−7.06 (m, 6H), 7.00−6.93 (m, 2H), 4.30 (d, A of AB, J_{AB} = 13.0 Hz, 1H), 4.23 (d, B of AB, J_{AB} = 13.0 Hz, 1H), 3.13−3.04 (m, 1H), 2.92−2.76 (m, 2H), 2.27−2.19 (m, 1H), 2.09 (br s, 1H); (^{13}C NMR, 101 MHz, $CDCl_3$): δ (ppm) = 158.8, 145.9, 137.9, 134.4, 128.9, 128.8, 127.7, 126.9, 126.5 (q, J = 283.7 Hz), 126.0, 123.3, 121.0, 61.1, 60.6, 47.9 (q, J = 24.6 Hz), 25.5, 22.7; (^{19}F NMR, 377 MHz, $CDCl_3$): δ (ppm) = −63.7 (d, J = 10.4 Hz, 3F); IR: 2931, 1636, 1589, 1491, 1445, 1303, 1263, 1191, 1150, 1117, 1035, 896 cm^{-1}; HRMS (ESI) for $C_{19}H_{18}N_2F_3S$ $[M+H]^+$ requires 363.1137 found 363.1134; Mp. 117−121 °C.

(1*S,2*S**)-*N*-(4-Bromophenyl)-2-(trifluoromethyl)-3,4-dihydro-2H,4'H-spiro[naphthalene-1,5'-thiazol]-2'-amine (2gb)**

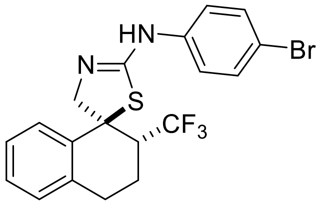

The title compound was prepared following general procedure H using 1-(4-bromophenyl)-3-((3,4-dihydronaphthalen-1-yl)methyl)thiourea (112.0 mg, 0.3 mmol), 1-trifluoromethyl-

1,2-benziodoxol-3-(1H)-one (113.8 mg, 0.36 mmol), 4Å MS (30 mg), trifluoroacetic acid (45.9 µL, 0.6 mmol) and $CDCl_3$ (3.0 mL). The crude product was purified by silica gel column chromatography (eluent: 1% Et_3N and 15 % EtOAc in *n*-pentane) to provide the title compound (109 mg, 82 % yield) as a white solid.
(^1H NMR, 400 MHz, $CDCl_3$): δ (ppm) = 7.72 (dt, J_1 = 3.7 Hz, J_2 = 9.3 Hz, 1H), 7.36 (dt, J = 9.3, 2.3 Hz, 2H), 7.21 (dt, J = 9.3, 3.7 Hz, 2H), 7.09 (dd, J_1 = 5.6, 3.9 Hz, 1H), 7.01 (d, J = 8.8 Hz, 2H), 4.25 (d, A of AB, J_{AB} = 12.6 Hz, 1H), 4.18 (d, B of AB, J_{AB} = 12.6 Hz, 1H), 3.19−3.12 (m, 1H), 2.97−2.87 (m, 2H), 2.35−2.27 (m, 1H), 2.19 (br s , 1H); (^{13}C NMR, 126 MHz, $CDCl_3$): δ (ppm) = 157.8, 145.4, 137.6, 134.6, 131.9, 129.0, 127.9, 127.0, 126.5 (q, J = 283.9 Hz), 126.0, 122.5, 116.0, 61.4, 60.3, 48.0 (q, J = 24.9 Hz), 25.6, 22.7; (^{19}F NMR, 377 MHz, $CDCl_3$): δ (ppm) = −63.8 (d, J = 10.4 Hz, 3F); IR: 3059, 2934, 2361, 1636, 1579, 1542, 1487, 1457, 1397, 1373, 1340, 1307, 1263, 1191, 1151, 1117, 1071, 1034, 1007, 934 cm^{-1}; HRMS (ESI) for $C_{19}H_{17}N_2BrF_3S$ $[M+H]^+$ requires 441.0242 found 441.0248; Mp. 179−181 °C.

(1*S,2*S**)-*N*-(4-Iodophenyl)-2-(trifluoromethyl)-3,4-dihydro-2H,4'H-spiro[naphthalene-1,5'-thiazol]-2'-amine (2gc)**

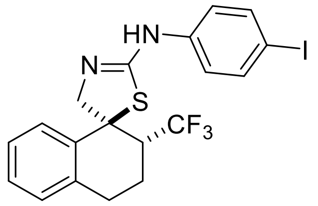

The title compound was prepared following general procedure H using 1-(4-iodomophenyl)-3-((3,4-dihydronaphthalen-1-yl)methyl)thiourea (126.1 mg, 0.3 mmol), 1-trifluoromethyl-1,2-benziodoxol-3-(1H)-one (113.8 mg, 0.36 mmol), 4Å MS (30 mg), trifluoroacetic acid (45.9 µL, 0.6 mmol) and $CDCl_3$ (3.0 mL). The crude product was purified by silica gel column chromatography (eluent: 1% Et_3N and 15 % EtOAc in *n*-pentane) to provide the title compound (103 mg, 70 % yield) as a white solid.
(^1H NMR, 400 MHz, $CDCl_3$): δ (ppm) = 7.71 (dt, J_2 = 9.3, 3.7 Hz, 1H), 7.55 (d, J = 8.8 Hz, 2H), 7.21 (dt, J= 9.3, 3.7 Hz, 2H), 7.09 (dd, J = 9.1, 3.7 Hz, 1H), 6.89 (d, J = 8.8 Hz, 2H), 4.23 (d, A of AB, J_{AB} = 12.6 Hz, 1H), 4.16 (d, B of AB, J_{AB} = 12.6 Hz, 1H),3.18−3.11 (m, 1H), 3.01−2.85 (m, 2H), 2.34−2.27 (m, 1H), 2.19 (br s, 1H); (^{13}C NMR, 101 MHz, $CDCl_3$): δ (ppm) = 158.1, 146.2, 137.9, 137.6, 134.6, 129.0, 127.9, 127.0, 126.5 (q, J = 283.2 Hz), 126.0, 123.0, 86.6, 61.3, 60.1, 48.0 (q, J = 24.9 Hz), 25.6, 22.7; (^{19}F NMR, 377 MHz, $CDCl_3$): δ (ppm) = −63.8 (d, J = 8.7 Hz, 3F); IR: 3024, 1635, 1575, 1538, 1514, 1483, 1451, 1394, 1372, 1341, 1308, 1263, 1191, 1150, 1118, 1035, 1003, 935 cm^{-1}; HRMS (ESI) for $C_{19}H_{17}N_2F_3IS$ $[M+H]^+$ requires 489.0104 found 489.0101; Mp. 164−166 °C.

(1S,2S)-N-(4-methoxyphenyl)-2-(trifluoromethyl)-3,4-dihydro-2H,4'H-spiro[naphthalene-1,5'-thiazol]-2'-amine (2gd)

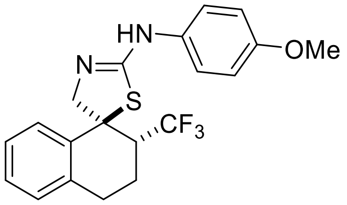

The title compound was prepared following general procedure H using 1-((3,4-dihydronaphthalen-1-yl)methyl)-3-(4-methoxyphenyl)thiourea (96.8 mg, 0.3 mmol), 1-trifluoromethyl-1,2-benziodoxol-3-(1H)-one (113.8 mg, 0.36 mmol), 4Å MS (30 mg), trifluoroacetic acid (45.9 µL, 0.6 mmol) and $CDCl_3$ (3.0 mL). The crude product was purified by silica gel column chromatography (eluent: Et_2O 10% in DCM) to provide the title compound (93 mg, 79 % yield) as a white solid.

(^{1}H NMR, 400 MHz, $CDCl_3$): δ (ppm) = 7.72−7.62 (m, 1H), 7.13−7.05 (m, 2H), 7.00−6.92 (m, 3H), 6.75-6.68 (m, 2H), 4.23 (d, A of AB, J_{AB} = 13.1 Hz, 1H), 4.17 (d, B of AB, J_{AB} = 13.1 Hz, 1H), 3.66 (s, 3H), 3.17−2.96 (m, 1H), 2.92−2.72 (m, 2H), 2.30-2.05 (m, 2H); (^{13}C NMR, 101 MHz, $CDCl_3$): δ (ppm) = 159.1, 156.0, 139.0, 138.1, 134.3, 127.9, 127.6, 126.9, 126.6 (q, J = 283.7 Hz), 126.0, 125.1, 122.7, 114.1, 61.3, 48.0 (q, J = 24.6 Hz), 25.5, 22.7 (q, J = 2.8 Hz); (^{19}F NMR, 377 MHz, $CDCl_3$): δ (ppm) = −63.7 (d, J = 10.0 Hz, 3F); IR: 2931, 2361, 1635, 1588, 1496, 1462, 1263, 1222, 1158, 1120, 1033, 860 cm^{-1}; HRMS (ESI) for $C_{20}H_{20}N_2F_3OS$ $[M+H]^+$ requires 393.1248 found 393.1259; Mp. 146−148 °C.

(1S*,2S*)-*N*-(naphthalen-2-yl)-2-(trifluoromethyl)-3,4-dihydro-2H,4'H-spiro[naphthalene-1,5'-thiazol]-2'-amine (2ge)

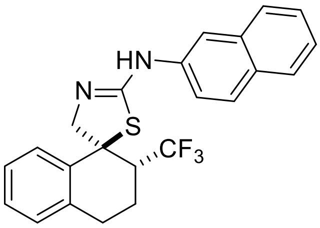

The title compound was prepared following general procedure H using 1-((3,4-dihydronaphthalen-1-yl)methyl)-3-(naphthalen-2-yl)thiourea(103 mg, 0.3 mmol), 1-trifluoromethyl-1,2-benziodoxol-3-(1H)-one (113.8 mg, 0.36 mmol), 4Å MS (30 mg), trifluoroacetic acid (45.9 µL, 0.6 mmol) and $CDCl_3$ (3.0 mL). The crude product was purified

by silica gel column chromatography (eluent: Et_2O 5% in DCM) to provide the title compound (101 mg, 82 % yield) as a white solid.
(^1H NMR, 400 MHz, $CDCl_3$): δ (ppm) = 8.13-8.03 (m, 1H), 7.71 (d, *J* = 8.2 Hz, 1H), 7.48 (d, *J* = 8.6 Hz, 1H), 7.42-7.33 (m, 2H), 7.27 (t, *J* = 7.6 Hz, 1H), 7.19-7.05 (m, 3H), 7.01 (d, *J* = 8.2 Hz, 1H), 6.93 (d, *J* = 7.6 Hz, 1H) 7.13−7.05 (m, 2H), 4.07 (d, A of AB, J_{AB} = 11.5 Hz, 1H), 4.02 (d, B of AB, J_{AB} = 11.5 Hz, 1H), 3.07−2.92 (m, 1H), 2.90−2.61 (m, 2H), 2.18-1.97 (m, 1H); (^{13}C NMR, 101 MHz, $CDCl_3$): δ (ppm) = 161.1, 146.1, 137.3, 134.7, 134.2, 128.9, 128.6, 127.9, 127.7, 126.8, 126.5 (q, *J* = 284.1 Hz), 126.1, 126.0, 125.8, 125.4, 123.9, 123.4, 117.1, 58.4, 55.7, 48.0 (q, *J* = 24.6 Hz), 22.0 (q, *J* = 2.8 Hz) ; (^{19}F NMR, 377 MHz, $CDCl_3$): δ (ppm) = −63.7 (d, *J* = 9.6 Hz, 3F); IR: 3156, 3025, 1675, 1558, 1498, 1421, 1250, 1242, 1132, 1120, 1023, 960 cm^{-1}; HRMS (ESI) for $C_{23}H_{20}N_2F_3S$ $[M+H]^+$ requires 413.1294 found 413.1290; Mp. 148-150 °C.

(1S*,2S*)-2-(trifluoromethyl)-*N*-(4-(trifluoromethyl)phenyl)-3,4-dihydro-2H,4'H-spiro[naphthalene-1,5'-thiazol]-2'-amine (2gf)

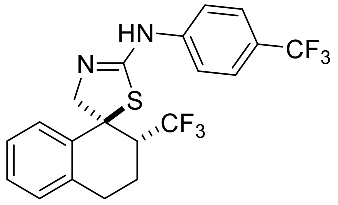

The title compound was prepared following general procedure H using 1-((3,4-dihydronaphthalen-1-yl)methyl)-3-(4-(trifluoromethyl)phenyl)thiourea (109.1 mg, 0.3 mmol), 1-trifluoromethyl-1,2-benziodoxol-3-(1H)-one (113.8 mg, 0.36 mmol), 4Å MS (30 mg), trifluoroacetic acid (45.9 µL, 0.6 mmol) and $CDCl_3$ (3.0 mL). The crude product was purified by silica gel column chromatography (eluent: Et_2O 5% in DCM) to provide the title compound (82 mg, 64 % yield) as a yellow thick oil.
(^1H NMR, 400 MHz, $CDCl_3$): δ (ppm) = 7.62-7.56 (m, 1H), 7.45-7.30 (m, 4H), 7.17−6.92 (m, 3H), 4.36 (d, A of AB, J_{AB} = 13.1 Hz, 1H), 4.31 (d, B of AB, J_{AB} = 13.1 Hz, 1H), 3.19−2.98 (m, 1H), 2.92−2.75 (m, 2H), 2.31-2.05 (m, 2H); ^{13}C NMR, 101 MHz, $CDCl_3$): δ (ppm) = 158.6, 149.6, 137.4, 134.7, 129.1, 128.0, 127.0, 126.3 (q, *J* = 3.7 Hz), 125.3, 126.0, 120.8, 120.6 (q, *J* = 283.8 Hz), 61.3, 59.9, 48.1 (q, *J* = 24.6 Hz), 25.7, 22.6; (^{19}F NMR, 377 MHz, $CDCl_3$): δ (ppm) = -61.7 (s, 3F), −63.9 (d, *J* = 9.4 Hz, 3F); IR: 2991, 2341, 1625, 1578, 1490, 1452, 1283, 1212, 1138, 1110, 1013, 900 cm^{-1}; HRMS (ESI) for $C_{23}H_{20}N_2F_3S$ $[M+H]^+$ requires 413.1294 found 413.1290; Mp. 148-150 °C.

Methyl 4-(((1*S,2*S**)-2-(trifluoromethyl)-3,4-dihydro-2H,4'H-spiro[naphthalene-1,5'-thiazol]-2'-yl)amino)benzoate (2gg)**

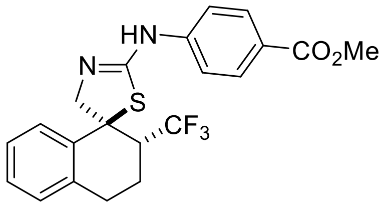

The title compound was prepared following general procedure H for 48h using methyl 4-(3-((3,4-dihydronaphthalen-1-yl)methyl)thioureido)benzoate (107 mg, 0.3 mmol), 1-trifluoromethyl-1,2-benziodoxol-3-(1H)-one (113.8 mg, 0.36 mmol), 4Å MS (30 mg), trifluoroacetic acid (45.9 µL, 0.6 mmol) and $CDCl_3$ (3.0 mL). The crude product was purified by silica gel column chromatography (eluent: Et_2O 10% in DCM) to provide the title compound (73 mg, 58 % yield) as a white solid.

(^{1}H NMR, 400 MHz, $CDCl_3$): δ (ppm) = 7.95 (dt, *J* = 8.6, 2.0 Hz, 2H), 7.70 (dt, *J* = 9.3, 3.7 Hz, 1H), 7.23−7.19 (m, 4H), 7.11−7.08 (m, 1H), 4.30 (d, A of AB, J_{AB} = 13.2 Hz, 1H), 4.23 (d, B of AB, J_{AB} = 13.2 Hz, 1H), 3.88 (s, 3H), 3.20−3.13 (m, 1H), 2.97−2.90 (m, 2H), 2.35−2.28 (m, 1H), 2.18 (br s , 1H); (^{13}C NMR, 101 MHz, $CDCl_3$): δ (ppm) = 166.8, 157.5, 149.8, 137.6, 134.5, 130.9, 129.0, 128.0, 127.0, 126.4 (q, *J* = 282.9 Hz), 126.0, 124.6, 119.9, 61.9, 61.3, 51.9, 47.9 (q, *J* = 25.4 Hz), 25.6, 22.8; (^{19}F NMR, 377 MHz, $CDCl_3$): δ (ppm) = −63.9 (d, *J* = 8.7 Hz, 3F); IR: 3328, 3024, 2949, 1715, 1639, 1593, 1539, 1436, 1413, 1280, 1265, 1192, 1175, 1116, 1103, 858 cm^{-1}; HRMS (ESI) for $C_{21}H_{20}O_2N_2F_3S$ $[M+H]^+$ requires 421.1192 found 421.1180; Mp. 195−196 °C.

(1*S,2*S**)-*N*-Benzyl-2-(trifluoromethyl)-3,4-dihydro-2H,4'H-spiro[naphthalene-1,5'-thiazol]-2'-amine (2gh)**

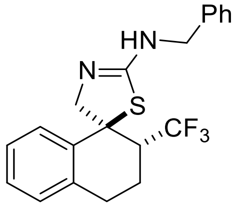

The title compound was prepared following general procedure H using 1-benzyl-3-((3,4-dihydronaphthalen-1-yl)methyl)thiourea (92.5 mg, 0.3 mmol), 1-trifluoromethyl-1,2-benziodoxol-3-(1H)-one (113.8 mg, 0.36 mmol), 4Å MS (30 mg), trifluoroacetic acid (45.9 µL, 0.6 mmol) and $CDCl_3$ (3.0 mL). The crude product was purified by silica gel column

chromatography (eluent: 1% Et_3N and 15 % EtOAc in *n*-pentane) to provide the title compound (84.7 mg, 75 % yield) as a white solid.
(^{1}H NMR, 400 MHz, $CDCl_3$): δ (ppm) = 7.64−7.62 (m, 1H), 7.37−7.27 (m, 5H), 7.24−7.19 (m, 2H), 7.10−7.08 (m, 2H), 4.34 (s, 2H), 4.30 (d, A of AB, J_{AB} = 14.2 Hz, 1H), 4.24 (d, B of AB, J_{AB} = 14.2 Hz, 1H), 3.23−3.13 (m, 1H), 3.02−2.87 (m, 2H), 2.40−2.32 (m, 1H), 2.16−2.08 (m, 1H); (^{13}C NMR, 101 MHz, $CDCl_3$): δ (ppm) = 158.6, 139.3, 138.3, 133.6, 128.6, 128.5, 127.6, 127.5, 127.4, 126.8, 126.5 (q, *J* = 283.7 Hz), 125.8, 68.4, 66.2, 48.5, 47.8 (q, *J* = 24.9 Hz), 25.5, 23.9; (^{19}F NMR, 377 MHz, $CDCl_3$): δ (ppm) = −63.9 (d, *J* = 8.7 Hz, 3F); IR: 3195, 3028, 2938, 1625, 1541, 1491, 1454, 1372, 1338, 1298, 1264, 1178, 1147, 1117, 1032, 933 cm^{-1}; HRMS (ESI) for $C_{20}H_{20}N_2F_3S$ [M+H]$^+$ requires 377.1294 found 377.1303; Mp. 67−68 °C.

(1*S,2*S**)-*N*-(*tert*-butyl)-2-(trifluoromethyl)-3,4-dihydro-2H,4'H-spiro[naphthalene-1,5'-thiazol]-2'-amine (2gi)**

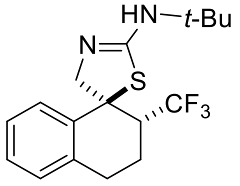

The title compound was prepared following general procedure H using 1-(*tert*-butyl)-3-((3,4-dihydronaphthalen-1-yl)methyl)thiourea (82.3 mg, 0.3 mmol), 1-trifluoromethyl-1,2-benziodoxol-3-(1H)-one (113.8 mg, 0.36 mmol), 4Å MS (30 mg), trifluoroacetic acid (45.9 µL, 0.6 mmol) and $CDCl_3$ (3.0 mL). The crude product was purified by silica gel column chromatography (eluent: 1% Et_3N and 10 % EtOAc in *n*-pentane) to provide the title compound (84.2 mg, 82 % yield) as a white solid.
(^{1}H NMR, 400 MHz, $CDCl_3$): δ (ppm) = 7.64 (dt, *J* = 9.3, 3.6 Hz, 1H), 7.19 (dt, *J* = 9.3, 3.6 Hz, 2H), 7.08−7.06 (m, 1H), 4.38 (d, A of AB, J_{AB} = 14.2 Hz, 1H), 4.33 (d, B of AB, J_{AB} = 14.2 Hz, 1H), 4.12 (br s , 1H), 3.19−3.09 (m, 1H), 3.00−2.86 (m, 2H), 2.39−2.31 (m, 1H), 2.15−2.07 (m, 1H), 1.40 (s, 9H); (^{13}C NMR, 101 MHz, $CDCl_3$): δ (ppm) = 155.2, 139.5, 133.7, 128.6, 127.4, 126.7, 126.5 (q, *J* = 283.7 Hz), 125.8, 70.1, 65.3, 53.0, 47.8 (q, *J* = 24.6 Hz), 28.9, 25.6, 24.1 (q, *J* = 2.4 Hz); (^{19}F NMR, 377 MHz, $CDCl_3$): δ (ppm) = −64.1 (d, *J* = 10.4 Hz, 3F); IR: 3279, 2965, 1638, 1490, 1453, 1391, 1364, 1339, 1298, 1247, 1219, 1201 1189, 1178, 1148, 1117, 1106, 1024, 933 cm^{-1}; HRMS (ESI) for $C_{17}H_{22}N_2F_3S$ [M+H]$^+$ requires 343.1450 found 343.1447; Mp. 114−116 °C.

(1*S,2*S**)-*N*-(Benzo[d]thiazol-2-yl)-2-(trifluoromethyl)-3,4-dihydro-2H,4'H-spiro[naphthalene-1,5'-thiazol]-2'-amine (2gj)**

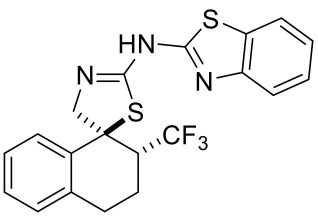

The title compound was prepared following general procedure H using 1-(benzo[d]thiazol-2-yl)-3-((3,4-dihydronaphthalen-1-yl)methyl)thiourea (105.4 mg, 0.3 mmol), 1-trifluoromethyl-1,2-benziodoxol-3-(1H)-one (113.8 mg, 0.36 mmol), 4Å MS (30 mg), trifluoroacetic acid (45.9 µL, 0.6 mmol) and $CDCl_3$ (3.0 mL). The crude product was purified by silica gel column chromatography (eluent: 1% Et_3N and 20 % EtOAc in *n*-pentane) to provide the title compound (68.4 mg, 54 % yield) as a yellow solid.

(^{1}H NMR, 400 MHz, DMSO-d6): δ (ppm) = 9.31 (br s , 1H), 7.80 (d, *J* = 7.8 Hz, 1H), 7.70 (d, *J* = 6.1 Hz, 1H), 7.59 (d, *J* = 7.8 Hz, 1H), 7.32 (t, *J* = 7.6 Hz, 1H), 7.25−7.13 (m, 4H), 4.15 (br s , 2H), 3.55−3.49 (m, 1H), 3.10-2.71 (m, 2H), 2.20 (app q, *J* = 5.6 Hz, 2H); (^{13}C NMR, 126 MHz, DMSO-d6): δ (ppm) = 170.9, 165.6, 151.6, 138.1, 135.5, 132.9, 129.4, 128.2, 127.4 (q, *J* = 283.9 Hz), 127.2, 126.3, 126.1, 123.4, 121.9, 120.5, 57.8, 53.7, 46.5 (q, *J* = 24.2 Hz), 25.7, 21.7; (^{19}F NMR, 377 MHz, DMSO-d6): δ (ppm) = −63.3 (d, *J* = 9.5 Hz, 3F); IR: 2980, 1611, 1495, 1441, 1325, 1247, 1178, 1151, 1113, 1093, 1034, 915 cm^{-1}; HRMS (ESI) for $C_{20}H_{17}N_3F_3S_2$ $[M+H]^+$ requires 420.0811 found 420.0808; Mp. cannot be determined due to decomposition at >250 °C.

(1*S,2*S**)-*N*-(6-Methylpyridin-3-yl)-2-(trifluoromethyl)-3,4-dihydro-2H,4'H-spiro[naphthalene-1,5'-thiazol]-2'-amine (2gk)**

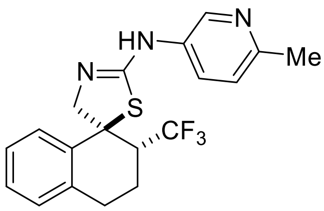

The title compound was prepared following general procedure H using 1-((3,4-dihydronaphthalen-1-yl)methyl)-3-(6-methylpyridin-3-yl)thiourea (92.8 mg, 0.3 mmol), 1-trifluoromethyl-1,2-benziodoxol-3-(1H)-one (113.8 mg, 0.36 mmol), 4Å MS (30 mg), trifluoroacetic acid (45.9 µL, 0.6 mmol) and $CDCl_3$ (3.0 mL). The crude product was purified

by silica gel column chromatography (eluent: 1% Et_3N and 20 % EtOAc in DCM) to provide the title compound (60.0 mg, 53 % yield) as a white solid.

(^{1}H NMR, 400 MHz, $CDCl_3$): δ (ppm) = 8.21 (d, *J* = 2.5 Hz, 1H), 7.74−7.71 (m, 1H), 7.37 (dd, *J* = 8.1, 2.7 Hz, 1H), 7.27−7.17 (m, 2H), 7.08−7.02 (m, 2H), 6.40 (br s , 1H), 4.31 (d, A of AB, J_{AB} = 12.6 Hz, 1H), 4.25 (d, B of AB, J_{AB} = 12.6 Hz, 1H), 3.17−3.08 (m, 1H), 2.99−2.83 (m, 2H), 2.48 (s, 3H), 2.31−2.23 (m, 1H), 2.16 (br s , 1H); (^{13}C NMR, 101 MHz, $CDCl_3$): δ (ppm) = 160.1, 152.9, 142.3, 141.5, 137.2, 134.7, 129.0, 129.0, 127.9, 126.9, 126.4 (q, *J* = 283.7 Hz), 126.0, 123.0, 60.4, 58.3, 47.9 (q, *J* = 25.4 Hz), 25.6, 23.6, 22.4; (^{19}F NMR, 377 MHz, $CDCl_3$): δ (ppm) = −63.8 (d, *J* = 10.4 Hz, 3F); IR: 3020, 2925, 1633, 1547, 1490, 1451, 1373, 1302, 1264, 1190, 1149, 1117, 1034, 934, 895, 834 cm^{-1}; HRMS (ESI) for $C_{19}H_{19}N_3F_3S$ $[M+H]^+$ requires 378.1246 found 378.1243; Mp. 193−194 °C.

(1*S,2*S**)-*N*-Methyl-*N*-phenyl-2-(trifluoromethyl)-3,4-dihydro-2H,4'H-spiro[naphthalene-1,5'-thiazol]-2'-amine (2gl)**

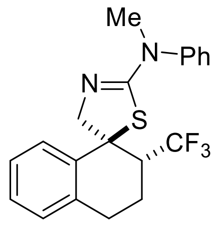

The title compound was prepared following general procedure H using 3-((3,4-dihydronaphthalen-1-yl)methyl)-1-methyl-1-phenylthiourea (92.5 mg, 0.3 mmol), 1-trifluoromethyl-1,2-benziodoxol-3-(1H)-one (113.8 mg, 0.36 mmol), 4Å MS (30 mg), trifluoroacetic acid (45.9 µL, 0.6 mmol) and $CDCl_3$ (3.0 mL). The crude product was purified by silica gel column chromatography (eluent: 1% Et_3N and 10 % EtOAc in *n*-pentane) to provide the title compound (95.6 mg, 85 % yield) as a pale yellow oil.

(^{1}H NMR, 400 MHz, $CDCl_3$): δ (ppm) = 7.72 (d, *J* = 7.8 Hz, 1H), 7.33−7.30 (m, 2H), 7.23−7.16 (m, 5H), 7.03 (d, *J* = 7.3 Hz, 1H), 4.58 (d, A of AB, J_{AB} = 15.6 Hz, 1H), 4.54 (d, B of AB, J_{AB} = 15.6 Hz, 1H), 3.46 (s, 3H), 3.16−3.09 (m, 1H), 2.96−2.78 (m, 2H), 2.32−2.24 (m, 1H), 2.06 (br s , 1H); (^{13}C NMR, 101 MHz, $CDCl_3$): δ (ppm) = 160.4, 145.9, 139.6, 133.8, 129.1, 128.7, 127.5, 126.9, 126.7, 126.7 (q, *J* = 283.5 Hz), 126.3, 126.2, 69.4 (q, *J* = 2.4 Hz), 66.5, 48.2 (q, *J* = 24.6 Hz), 40.2, 25.8, 23.8 (q, J = 3.2 Hz); (^{19}F NMR, 377 MHz, $CDCl_3$): δ (ppm) = −63.9 (d, *J* = 10.4 Hz, 3F); IR: 2938, 1617, 1590, 1496, 1453, 1355, 1264, 1178, 1146, 1116, 1030, 933 cm^{-1}; HRMS (ESI) for $C_{20}H_{20}N_2F_3S$ $[M+H]^+$ requires 377.1294 found 377.1299.

(1*S,2*S**)-2-(Trifluoromethyl)-3,4-dihydro-2H,4'H-spiro[naphthalene-1,5'-thiazol]-2'-amine (2gm)**

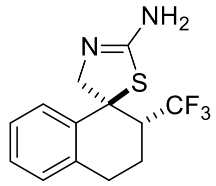

Into a vial containing (1*S**,2*S**)-*N*-(*tert*-butyl)-2-(trifluoromethyl)-3,4-dihydro-2H,4'H-spiro[naphthalene-1,5'-thiazol]-2'-amine (57.3 mg, 0.2 mmol) was added conc. HCl (0.5 mL) and the suspension was stirred at 110 °C for 30 min. The mixture was cooled in ice bath and basified to pH 10 with sat. $NaHCO_{3(aq)}$ (5 mL). The mixture was extracted with EtOAc (x2) and combined organic layers were washed with brine, dried over $MgSO_4$ and concentrated *in vacuo*. The crude solid was dissolved in minimum amount of $CHCl_3$ then precipitated by addition of *n*-pentane. After filtration, the title compound was obtained (44.2 mg, 77 % yield) as a white solid.

(^{1}H NMR, 400 MHz, $CDCl_3$): δ (ppm) = 7.63−7.59 (m, 1H), 7.22−7.18 (m, 2H), 7.09−7.07 (m, 1H), 4.58 (br s , 2H), 4.44 (d, A of AB, J_{AB} = 14.5 Hz, 1H), 4.39 (d, B of AB, J_{AB} = 14.5 Hz, 1H), 3.23−3.14 (m, 1H), 3.01−2.87 (m, 2H), 2.39−2.31 (m, 1H), 2.14−2.08 (m, 1H); (^{13}C NMR, 126 MHz, $CDCl_3$): δ (ppm) = 158.1, 139.2, 133.8, 128.8, 127.8, 127.0, 126.5 (q, *J* = 283.2 Hz), 126.0, 68.9 (q, *J* = 2.9 Hz), 67.7, 48.1 (q, *J* = 24.9 Hz), 25.7, 23.9 (q, *J* = 2.9 Hz); (^{19}F NMR, 377 MHz, $CDCl_3$): δ (ppm) = −64.0 (d, *J* = 10.4 Hz, 3F); IR: 3452, 2937, 1658, 1490, 1458, 1345, 1303, 1262, 1244, 1177, 1145, 1112, 1031, 991, 932, 894, 832 cm^{-1}; HRMS (ESI) for $C_{13}H_{14}N_2F_3S$ $[M+H]^+$ requires 287.0824 found 287.0824; Mp. 180−182 °C.

4'-Ethyl-N-phenyl-3,4-dihydro-2H,4'H-spiro[naphthalene-1,5'-thiazol]-2'-amine (2gn)

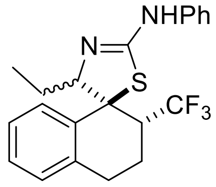

Into a vial containing 1-(1-(3,4-dihydronaphthalen-1-yl)propyl)-3-phenylthiourea **1gn** (162 mg, 0.5 mmol), 1-trifluoromethyl-1,2-benziodoxol-3-(1H)-one (190 mg, 0.6 mmol) and a stirrer bar was added a solution of trifluoroacetic acid (76.8 µL, 1 mmol) in $CHCl_3$ (5.0 mL) under Ar atmosphere. The vial was sealed and allowed to stir at room temperature for 24 hours. The reaction mixture was then concentrated and purified by silica gel column chromatography (eluent: EtOAc:hexane:Et_3N = 80:20:1) to provide 4'-ethyl-N-phenyl-3,4-

dihydro-2H,4'H-spiro[naphthalene-1,5'-thiazol]-2'-amine (93 mg, 48 % yield) as a pale yellow oil (93 mg, pale yellow oil) as a mixture of diastereomers (d.r. = 3.5:1, crude d.r. = 1.2:1).

(^{1}H NMR, 400 MHz, $CDCl_3$): δ (ppm) = 7.79-7.84 (m, 1H (major)), 6.85-7.48 (m, 8H (major) + 9H (minor)), 4.26 (d, *J* = 9.6 Hz, 1H (minor)), 4.13 (dd, *J* = 10.3, 2.2 Hz, 1H (major)), 3.11-3.25 (m, 1H (minor)), 2.71-2.94 (m, 3H (major) + 2H (minor)), 1.94-2.30 (m, 2H (major) + 2H (minor)), 1.69-1.83 (m, 1H, (minor)) 1.30-1.44 (m, 1H (major)), 1.06 (t, *J* = 2.4 Hz, 3H (minor)), 0.91-1.03 (m, 1H (major) + 1H (minor), 0.78 (t, *J* = 7.4 Hz, 3H (major)); (^{13}C NMR, 101 MHz, $CDCl_3$): δ (ppm) = 157.6 (major), 155.6 (minor), 147.1 (major), 146.6 (minor), 137.8 (minor), 136.0 (major), 135.9 (minor), 135.1 (major), 130.0 (major), 129.0 (major), 128.9 (major + minor), 128.7 (minor), 127.9 (major), 127.8 (minor), 127.0 (q, *J* = 282.4 Hz, (major)), 126.8 (q, *J* = 282.4 Hz, (minor)), 126.1 (minor), 126.0 (major), 124.3 (minor), 123.2 (major), 123.0 (minor), 121.3 (major), 120.9 (minor), 69.4 (major), 68.4 (minor), 64.4 (minor), 63.9 (major), 49.6 (q, J = 25.7 Hz), 43.6 (*J* = 25.7 Hz), 27.8 (major), 27.7 (major), 24.5 (minor), 23.6 (minor), 22.0 (major), 21.8 (minor), 12.4 (major), 11.2 (minor); (^{19}F NMR, 377 MHz, $CDCl_3$): δ (ppm) = −63.6 (d, *J* = 9.1 Hz, 3F (minor), −63.7 (d, *J* = 9.1 Hz, 3F (major)); IR: 2968, 2361, 1638, 1447, 1374, 1111, 1021, 951 cm^{-1}; HRMS (ESI) for $C_{21}H_{22}N_2F_3S$ $[M+H]^+$ requires 391.1450 found 391.1463;

(3S*,4S*)-*N*-Phenyl-3-(trifluoromethyl)-4'H-spiro[chromane-4,5'-thiazol]-2'-amine (2ha)

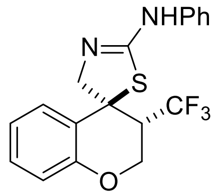

The title compound was prepared following general procedure H using 1-((2H-chromen-4-yl)methyl)-3-phenylthiourea (88 mg, 0.3 mmol), 1-trifluoromethyl-1,2-benziodoxol-3-(1H)-one (113.8 mg, 0.36 mmol), 4Å MS (30 mg), trifluoroacetic acid (45.9 µL, 0.6 mmol) and $CDCl_3$ (3.0 mL). The crude product was purified by silica gel column chromatography (eluent: 1% Et_3N and 20 % EtOAc in *n*-pentane) to provide the title compound (68.4 mg, 54 % yield) as a yellow solid.
(^{1}H NMR, 400 MHz, $CDCl_3$): δ (ppm) = 7.87 (dd, *J* = 8.1, 1.2 Hz, 1H), 7.29 (t, *J* = 7.8 Hz, 2H), 7.20 (td, *J* = 7.7, 1.4 Hz, 1H), 7.15 (d, *J* = 7.6 Hz, 2H), 7.07 (t, *J* = 7.5 Hz, 1H), 6.98 (td, *J* = 7.7, 1.0 Hz, 1H), 6.85 (d, *J* = 8.3 Hz, 1H), 4.65 (dd, *J* = 12.2, 3.2 Hz, 1H), 4.39−4.24 (m, 3H), 3.19−3.12 (m, 1H); (^{13}C NMR, 101 MHz, $CDCl_3$): δ (ppm) = 158.3, 153.3, 145.9, 129.6, 129.1, 127.0, 125.2 (q, *J* = 283.2 Hz), 123.7, 122.1, 121.8, 121.0, 117.3, 64.0 (q, *J* = 2.9 Hz), 59.1, 57.4, 47.2 (q, *J* = 25.7 Hz); (^{19}F NMR, 377 MHz, $CDCl_3$): δ (ppm) = −63.4 (d, *J* = 8.7 Hz, 3F); IR: 3034, 2889, 1639, 1590, 1489, 1449, 1358, 1313, 1277, 1236, 1164, 1130, 1108, 1073, 1040,

908, 856 cm^{-1}; HRMS (ESI) for $C_{18}H_{16}ON_2F_3S$ $[M+H]^+$ requires 365.0930 found 365.0928; Mp. 144−146 °C.

(3'R*,5S*)-*N*-Phenyl-3'-(trifluoromethyl)-4H-spiro[thiazole-5,4'-thiochroman]-2-amine (2ia)

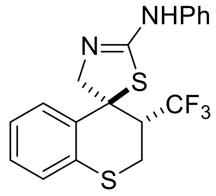

The title compound was prepared following general procedure H using (1-((2H-thiochromen-4-yl)methyl)-3-phenylthiourea (94 mg, 0.3 mmol), 1-trifluoromethyl-1,2-benziodoxol-3-(1H)-one (113.8 mg, 0.36 mmol), 4Å MS (30 mg), trifluoroacetic acid (45.9 µL, 0.6 mmol) and $CDCl_3$ (3.0 mL). The crude product was purified by silica gel column chromatography (eluent: 1% Et_3N and 20 % EtOAc in *n*-pentane) to provide the title compound (68.4 mg, 54 % yield) as a yellow solid.
(^1H NMR, 400 MHz, $CDCl_3$): δ (ppm) = 7.40 (d, J = 8.1 Hz, 1H), 7.16 (t, J = 7.0 Hz, 2H), 7.23-7.20 (m, 5H), 6.93 (t, J = 8.0 Hz, 1H), 4.52 (d, A of AB, J_{AB} = 13.2 Hz, 1H), 4.08 (d, B of AB, J_{AB} = 13.2 Hz, 1H), 3.44–3.26 (m, 3H), 3.21 (dd, J = 13.5, 3.0 Hz 1H); (^{13}C NMR, 101 MHz, $CDCl_3$): δ (ppm) = 157.8, 144.1, 134.4, 132.1, 129.0, 128.3, 127.1, 125.6, 125.4 (q, J = 283.2 Hz), 125.2, 123.5, 120.4, 61.9, 61.6, 47.0 (q, J = 25.7 Hz), 25.9 (q, J = 2.8 Hz); (^{19}F NMR, 377 MHz, $CDCl_3$): δ (ppm) = −64.8 (d, J = 8.7 Hz, 3F); IR: 3015, 2899, 1626, 1550, 1519, 1489, 1378, 1388, 1279, 1296, 1194, 1160, 1148, 1004, 978, 841 cm^{-1}; HRMS (ESI) for $C_{18}H_{16}N_2F_3S_2$ $[M+H]^+$ requires 381.0707 found 381.0712; Mp. 170-172 °C.

(1S*,2S*)-*N*-Phenyl-2-(trifluoromethyl)-2,3-dihydro-4'H-spiro[indene-1,5'-thiazol]-2'-amine (2ja)

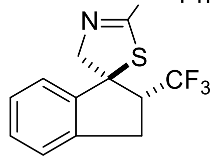

The title compound was prepared following general procedure H using 1-((1H-inden-3-yl)methyl)-3-phenylthiourea (84.0 mg, 0.3 mmol), 1-trifluoromethyl-1,2-benziodoxol-3-(1H)-

one (113.8 mg, 0.36 mmol), 4Å MS (30 mg), trifluoroacetic acid (45.9 µL, 0.6 mmol) and $CDCl_3$ (3.0 mL). The crude product was purified by silica gel column chromatography (eluent: 1% Et_3N and 20 % EtOAc in DCM) to provide the title compound (78.4 mg, 75 % yield) as a white solid.

(^1H NMR, 400 MHz, $CDCl_3$): δ (ppm) = 7.41 (d, J = 7.8 Hz, 1H), 7.23-7.14 (m, 2H), 7.15-6.92 (m, 6H), 3.50-3.31 (m, 2H), (m, 1H), 7.20−7.06 (m, 6H), 7.00−6.93 (m, 2H), 4.56 (d, A of AB, J_{AB} = 13.2 Hz, 1H), 4.12 (d, B of AB, J_{AB} = 13.2 Hz, 1H), 3.48-3.30 (m, 2H), 3.25 (dd, J = 13.4, 2.7 Hz, 1H); (^{13}C NMR, 101 MHz, $CDCl_3$): δ (ppm) = 157.1, 143.6, 134.5, 132.1, 129.1, 128.4, 127.1, 125.6, 125.4 (q, J = 284.7 Hz), 125.3, 123.4, 120.1, 62.9, 62.2, 47.1 (q, J = 26.0 Hz), 26.0 (q, J = 3.0 Hz); (^{19}F NMR, 377 MHz, $CDCl_3$): δ (ppm) = −66.3 (br s, 3F); IR: 2981, 1676, 1589, 1497, 1467, 1345, 1283, 1171, 1130, 1127, 1055, 996 cm^{-1}; HRMS (ESI) for $C_{18}H_{16}N_2F_3S$ $[M+H]^+$ requires 349.0981 found 349.0977; Mp. 138-140 °C.

(1*S,2*S**)-*N*-Phenyl-2-(trifluoromethyl)-3,4,4',5'-tetrahydro-2H-spiro[naphthalene-1,6'-[1,3]thiazin]-2'-amine (2ka)**

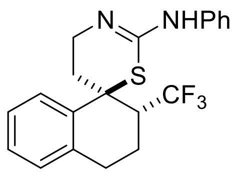

The title compound was prepared following general procedure H using *N*-((3,4-dihydronaphthalen-1-yl)methyl)benzamide (92.5 mg, 0.3 mmol), 1-trifluoromethyl-1,2-benziodoxol-3-(1H)-one (113.8 mg, 0.36 mmol), 4Å MS (30 mg), trifluoroacetic acid (45.9 µL, 0.6 mmol) and $CDCl_3$ (3.0 mL). The crude product was purified by silica gel column chromatography (eluent: 1% Et_3N and 10 % EtOAc in *n*-pentane) to provide the title compound (59.5 mg, 53 % yield) as a white solid.

(^1H NMR, 400 MHz, $CDCl_3$): δ (ppm) = 7.47 (d, J = 7.6 Hz, 1H), 7.17−7.06 (m, 6H), 6.98 (d, J = 7.1 Hz, 1H), 6.90 (t, J = 7.2 Hz, 1H), 6.46 (br s , 1H), 3.81−3.71 (m, 2H), 3.08−2.99 (m, 1H), 2.89−2.71 (m, 3H), 2.54 (br s , 1H), 2.34 (d, J = 14.4 Hz, 1H), 2.19−2.12 (m, 1H); (^{13}C NMR, 101 MHz, $CDCl_3$): δ (ppm) = 149.7, 143.4, 137.1, 135.8, 129.2, 128.8, 127.6, 126.8 (q, J = 284.6 Hz), 126.7, 126.6, 122.8, 121.2, 50.6, 45.7 (q, J = 24.0 Hz), 41.8, 30.7, 25.3, 20.0 (q, J = 2.9 Hz); (^{19}F NMR, 377 MHz, $CDCl_3$): δ (ppm) = −61.4 (d, J = 8.7 Hz, 3F); IR: 2980, 1625, 1587, 1495, 1439, 1367, 1309, 1261, 1200, 1176, 1141, 1121, 1107, 1078, 1019, 947, 837 cm^{-1}; HRMS (ESI) for $C_{20}H_{20}N_2F_3S$ $[M+H]^+$ requires 377.1294 found 377.1299; Mp. 136−139 °C.

4-Methyl-*N*-phenyl-4-(2,2,2-trifluoroethyl)-4H-benzo[d][1,3]thiazin-2-amine (2la)

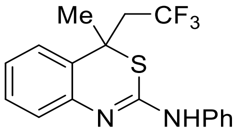

The title compound was prepared following general procedure H using 1-phenyl-3-(2-(prop-1-en-2-yl)phenyl)thiourea (80.5 mg, 0.3 mmol), 1-trifluoromethyl-1,2-benziodoxol-3-(1H)-one (113.8 mg, 0.36 mmol), 4Å MS (30 mg), trifluoroacetic acid (45.9 µL, 0.6 mmol) and $CDCl_3$ (3.0 mL). The crude product was purified by silica gel column chromatography (eluent: 1% Et_3N and 5 % EtOAc in *n*-pentane) to provide the title compound (58.8 mg, 58 % yield) as a white solid.

(^{1}H NMR, 400 MHz, $CDCl_3$): δ (ppm) = 7.51 (d, *J* = 7.8 Hz, 2H), 7.37 (t, *J* = 8.1 Hz, 3H), 7.31 (t, *J* = 7.6 Hz, 1H), 7.21−7.11 (m, 3H), 2.69 (ddq, *J* = 63.0, 15.4, 10.8 Hz, 2H), 1.98 (s, 3H); (^{13}C NMR, 101 MHz, $CDCl_3$): δ (ppm) = 150.4, 142.8, 141.3, 129.0, 128.6, 126.8, 125.1 (q, *J* = 279.7 Hz), 124.4, 123.9, 123.8, 122.7, 120.9, 44.7, 43.5 (q, *J* = 27.0 Hz), 23.1; (^{19}F NMR, 377 MHz, $CDCl_3$): δ (ppm) = −60.1 (t, *J* = 10.4 Hz, 3F); IR: 2980, 2361, 1617, 1578, 1521, 1496, 1478, 1439, 1359, 1315, 1257, 1232, 1127, 1105, 1074, 1032, 940 cm^{-1}; HRMS (ESI) for $C_{17}H_{16}N_2F_3S$ $[M+H]^+$ requires 337.0981 found 337.0983; Mp. 82−84 °C.

7-Fluoro-4-methyl-*N*-phenyl-4-(2,2,2-trifluoroethyl)-4H-benzo[d][1,3]thiazin-2-amine (2ma)

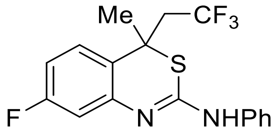

The title compound was prepared following general procedure H using 1-(5-fluoro-2-(prop-1-en-2-yl)phenyl)-3-phenylthiourea (85.9 mg, 0.3 mmol), 1-trifluoromethyl-1,2-benziodoxol-3-(1H)-one (113.8 mg, 0.36 mmol), 4Å MS (30 mg), trifluoroacetic acid (45.9 µL, 0.6 mmol) and $CDCl_3$ (3.0 mL). The crude product was purified by silica gel column chromatography (eluent: 1% Et_3N and 10 % EtOAc in *n*-pentane) to provide the title compound (68.3 mg, 64 % yield) as a white solid.

(^{1}H NMR, 400 MHz, $CDCl_3$): δ (ppm) = 7.60 (d, *J* = 7.8 Hz, 2H), 7.37 (t, *J* = 7.9 Hz, 2H), 7.24 (dd, *J* = 8.6, 5.6 Hz, 1H), 7.13 (t, *J* = 7.6 Hz, 1H), 7.09−7.01 (m, 2H), 6.92 (br s , 1H), 2.65 (ddq, *J* = 58.8, 15.4, 10.8 Hz, 2H), 1.95 (s, 3H); (^{13}C NMR, 101 MHz, $CDCl_3$): δ (ppm) = 161.2, 158.8, 149.2, 140.1 (d, *J* = 2.4 Hz), 139.9, 129.0, 126.3 (d, *J* = 8.0 Hz), 125.0 (q, *J* = 279.8 Hz), 123.8,

120.5, 115.3 (d, J = 22.3 Hz), 109.7 (d, J = 24.6 Hz), 44.5, 43.2 (q, J = 27.0 Hz), 23.0; (^{19}F NMR, 377 MHz, $CDCl_3$): δ (ppm) = −60.0 (t, J = 10.4 Hz, 3F), −117.5 (q, J = 7.5 Hz, 1F); IR: 2981, 2360, 1616, 1587, 1521, 1498, 1480, 1439, 1360, 1316, 1257, 1187, 1145, 1091, 1035, 958 cm^{-1}; HRMS (ESI) for $C_{17}H_{15}N_2F_4S$ [M+H]$^+$ requires 355.0887 found 355.0880; Mp. 133−135 °C.

(5*R,6*S**)-*N*,6-Diphenyl-5-(trifluoromethyl)-5,6-dihydro-4H-1,3-thiazin-2-amine (2na)**

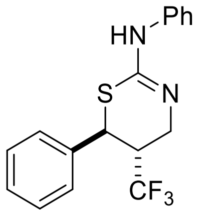

The title compound was prepared following general procedure H using 1-cinnamyl-3-phenylthiourea (80.5 mg, 0.3 mmol), 1-trifluoromethyl-1,2-benziodoxol-3-(1H)-one (113.8 mg, 0.36 mmol), 4Å MS (30 mg), trifluoroacetic acid (45.9 µL, 0.6 mmol) and $CDCl_3$ (3.0 mL). The crude product was purified by silica gel column chromatography (eluent: 1% Et_3N and 10 % EtOAc in *n*-pentane) to provide the title compound (40.0 mg, 40 % yield) as a white solid.

(^{1}H NMR, 400 MHz, $CDCl_3$): δ (ppm) = 7.28−7.15 (m, 9H), 6.96−6.92 (m, 1H), 5.65 (br s , 1H), 4.46 (d, J = 8.8 Hz, 1H), 3.88 (dd, A of ABX, J_{AB} = 13.7 Hz, J_{AX} = 4.4 Hz, 1H), 3.56 (B of ABX, J_{AB} = 13.7 Hz, J_{BX} = 7.8 Hz, 1H), 2.89−2.81 (m, 1H); (^{13}C NMR, 101 MHz, $CDCl_3$): δ (ppm) = 152.7, 142.5, 139.9, 129.0, 128.9, 128.4, 127.7, 126.3 (q, J = 281.7 Hz), 123.2, 120.6, 46.1 (q, J = 24.2 Hz), 45.4, 44.8; (^{19}F NMR, 377 MHz, $CDCl_3$): δ (ppm) = −68.5 (d, J = 6.9 Hz, 3F); IR: 2981, 1626, 1589, 1520, 1496, 1456, 1439, 1382, 1308, 1248, 1167, 1118, 1079, 995, 955 cm^{-1}; HRMS (ESI) for $C_{17}H_{16}N_2F_3S$ [M+H]$^+$ requires 337.0981 found 337.0976; Mp. 116−118 °C.

(5*R,6*S**)-6-(4-Methoxyphenyl)-*N*-phenyl-5-(trifluoromethyl)-5,6-dihydro-4H-1,3-thiazin-2-amine (2oa)**

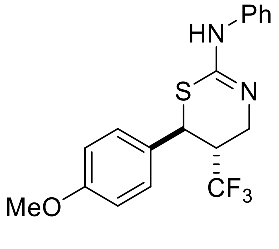

The title compound was prepared following general procedure H using (*E*)-1-(3-(4-methoxyphenyl)allyl)-3-phenylthiourea (89.5 mg, 0.3 mmol), 1-trifluoromethyl-1,2-benziodoxol-3-(1H)-one (113.8 mg, 0.36 mmol), 4Å MS (30 mg), trifluoroacetic acid (45.9 µL,

0.6 mmol) and $CDCl_3$ (3.0 mL). The crude product was purified by silica gel column chromatography (eluent: 1% Et_3N and 10 % EtOAc in *n*-pentane) to provide the title compound (60.4 mg, 55 % yield) as a white solid.
(^1H NMR, 400 MHz, $CDCl_3$): δ (ppm) = 7.33−7.28 (m, 6H), 7.08−7.03 (m, 1H), 6.90 (dt, *J* = 8.6, 2.6 Hz, 2H), 6.07 (br s , 1H), 4.56 (d, *J* = 9.1 Hz, 1H), 4.00 (dd, *J* = 13.9, 4.2 Hz, 1H), 3.83 (s, 3H), 3.67 (dd, *J* = 13.7, 8.1 Hz, 1H), 2.99−2.88 (m, 1H); (^{13}C NMR, 101 MHz, $CDCl_3$): δ (ppm) = 159.6, 153.0, 142.6, 131.6, 129.0, 129.0, 126.3 (q, *J* = 282.1 Hz), 123.2, 120.6, 114.4, 55.3, 46.3 (q, *J* = 27.8 Hz), 45.0, 44.9; (^{19}F NMR, 377 MHz, $CDCl_3$): δ (ppm) = −68.4 (d, *J* = 6.9 Hz, 3F); IR: 2980, 2360, 1611, 1588, 1497, 1459, 1439, 1381, 1306, 1247, 1176, 1109, 1033, 995, 835 cm^{-1}; HRMS (ESI) for $C_{18}H_{18}ON_2F_3S$ $[M+H]^+$ requires 367.1087 found 367.1083; Mp. 136−138 °C.

(5*R,6*S**)-*N*-Phenyl-6-(*p*-tolyl)-5-(trifluoromethyl)-5,6-dihydro-4H-1,3-thiazin-2-amine (2pa)**

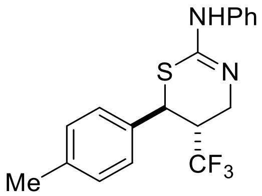

The title compound was prepared following general procedure H using (*E*)-1-Phenyl-3-(3-(*p*-tolyl)allyl)thiourea (84.7 mg, 0.3 mmol), 1-trifluoromethyl-1,2-benziodoxol-3-(1H)-one (113.8 mg, 0.36 mmol), 4Å MS (30 mg), trifluoroacetic acid (45.9 µL, 0.6 mmol) and $CDCl_3$ (3.0 mL). The crude product was purified by silica gel column chromatography (eluent: 1% Et_3N and 10 % EtOAc in *n*-pentane) to provide the title compound (49.2 mg, 47 % yield) as a white solid.
(^1H NMR, 400 MHz, $CDCl_3$): δ (ppm) = 7.21−7.13 (m, 6H), 7.06 (d, *J* = 8.1 Hz, 2H), 6.96−6.92 (m, 1H), 4.44 (d, *J* = 9.1 Hz, 1H), 3.89 (dd, A of ABX, J_{AB} = 13.7 Hz, J_{AX} = 4.2 Hz, 1H), 3.55 (dd, B of ABX, J_{AB} = 13.7 Hz, J_{BX} = 8.1 Hz, 1H), 2.87−2.82 (m, 1H), 2.25 (s, 3H); (^{13}C NMR, 101 MHz, $CDCl_3$): δ (ppm) = 153.0, 142.6, 138.3, 136.8, 129.6, 128.9, 127.6, 126.3 (q, *J* = 281.3 Hz), 123.1, 120.5, 46.2 (q, *J* = 23.8 Hz), 45.2, 44.9, 21.1; (^{19}F NMR, 377 MHz, $CDCl_3$): δ (ppm) = −68.5 (d, *J* = 6.9 Hz, 3F); IR: 2981, 2360, 1627, 1589, 1515, 1496, 1438, 1438, 1381, 1307, 1248, 1166, 1111, 1074, 995, 955, 844 cm^{-1}; HRMS (ESI) for $C_{18}H_{18}N_2F_3S$ $[M+H]^+$ requires 351.1137 found 351.1140; Mp. 117−119 °C.

(5R*,6S*)-6-(4-Chlorophenyl)-N-phenyl-5-(trifluoromethyl)-5,6-dihydro-4H-1,3-thiazin-2-amine (2qa)

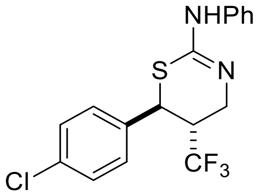

The title compound was prepared following general procedure H using (E)-1-(3-(4-chlorophenyl)allyl)-3-phenylthiourea (90.8 mg, 0.3 mmol), 1-trifluoromethyl-1,2-benziodoxol-3-(1H)-one (113.8 mg, 0.36 mmol), 4Å MS (30 mg), trifluoroacetic acid (45.9 µL, 0.6 mmol) and $CDCl_3$ (3.0 mL). The crude product was purified by silica gel column chromatography (eluent: 1% Et_3N and 10 % EtOAc in n-pentane) to provide the title compound (55.8 mg, 50 % yield) as a white solid.

(^1H NMR, 400 MHz, $CDCl_3$): δ (ppm) = 7.25−7.17 (m, 8H), 6.95 (t, J = 6.7 Hz, 1H), 4.44 (d, J = 8.8 Hz, 1H), 3.89 (dd, A of ABX, J_{AB} = 13.9 Hz, J_{AX} = 4.4 Hz, 1H), 3.54 (dd, A of ABX, J_{AB} = 13.9 Hz, J_{AX} = 4.4 Hz, 1H), 2.82−2.75 (m, 1H); (^{13}C NMR, 101 MHz, $CDCl_3$): δ (ppm) = 152.4, 142.3, 138.4, 134.3, 129.2, 129.1, 129.0, 126.1 (q, J = 281.3 Hz), 123.3, 120.5, 46.1 (q, J = 24.4 Hz), 44.8, 44.8; (^{19}F NMR, 377 MHz, $CDCl_3$): δ (ppm) = −68.4 (d, J = 8.7 Hz, 3F); IR: 2981, 2888, 2360, 1628, 1589, 1520, 1493, 1438, 1381, 1310, 1248, 1167, 1119, 1074, 1015, 995, 955, 841 cm^{-1}; HRMS (ESI) for $C_{17}H_{15}N_2ClF_3S$ $[M+H]^+$ requires 371.0591 found 371.0591; Mp. 98−99 °C.

(5R*,6S*)-6-phenyl-N-(p-tolyl)-5-(trifluoromethyl)-5,6-dihydro-4H-1,3-thiazin-2-amine (2nb)

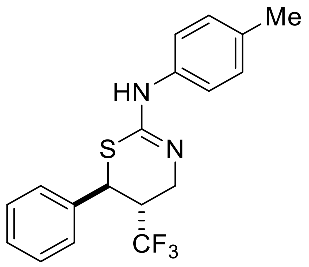

The title compound was prepared following general procedure H using 1-cinnamyl-3-(p-tolyl)thiourea (84.7 mg, 0.3 mmol), 1-trifluoromethyl-1,2-benziodoxol-3-(1H)-one (113.8 mg, 0.36 mmol), 4Å MS (30 mg), trifluoroacetic acid (45.9 µL, 0.6 mmol) and $CDCl_3$ (3.0 mL). The crude product was purified by silica gel column chromatography (eluent: 1% Et_3N and 20 % EtOAc in n-pentane) to provide the title compound (45.6 mg, 44 % yield) as a white solid.

(^{1}H NMR, 400 MHz, $CDCl_3$): δ (ppm) = 7.38−7.29 (m, 5H), 7.15 (d, *J* = 7.6 Hz, 2H), 7.09 (d, *J* = 8.3 Hz, 2H), 4.55 (d, *J* = 9.1 Hz, 1H), 3.99 (dd, A of ABX, J_{AB} = 13.7 Hz, J_{AX} = 3.7 Hz, 1H), 3.65 (dd, B of ABX, J_{AB} = 13.7 Hz, J_{AX} = 8.1 Hz, 1H), 2.98−2.92 (m, 1H), 2.30 (s, 3H); (^{13}C NMR, 101 MHz, $CDCl_3$): δ (ppm) = 152.9, 140.0, 139.8, 132.9, 129.5, 128.9, 128.4, 127.7, 126.3 (q, *J* = 281.3 Hz), 120.8, 46.2 (q, *J* = 24.6 Hz), 45.4 (q, *J* = 24.6 Hz), 44.8 (q, *J* = 1.0 Hz), 20.8; (^{19}F NMR, 377 MHz, $CDCl_3$): δ (ppm) = −68.5 (d, *J* = 8.7 Hz, 3F); IR: 2981, 1628, 1605, 1510, 1456, 1382, 1301, 1247, 1168, 1117, 1048, 998, 825 cm^{-1}; HRMS (ESI) for $C_{18}H_{18}N_2F_3S$ $[M+H]^+$ requires 351.1137 found 351.1133; Mp. 126−128 °C.

(5*R,6*S**)-*N*-(4-methoxyphenyl)-6-phenyl-5-(trifluoromethyl)-5,6-dihydro-4H-1,3-thiazin-2-amine (2nc)**

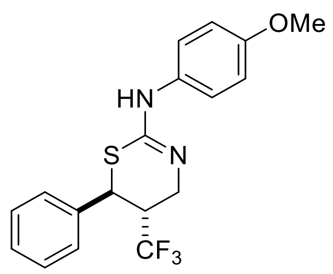

The title compound was prepared following general procedure H using 1-cinnamyl-3-(4-methoxyphenyl)thiourea (89.5 mg, 0.3 mmol), 1-trifluoromethyl-1,2-benziodoxol-3-(1H)-one (113.8 mg, 0.36 mmol), 4Å MS (30 mg), trifluoroacetic acid (45.9 µL, 0.6 mmol) and $CDCl_3$ (3.0 mL). The crude product was purified by silica gel column chromatography (eluent: 1% Et_3N and 20 % EtOAc in *n*-pentane) to provide the title compound (57.3 mg, 52 % yield) as a white solid.

(^{1}H NMR, 400 MHz, $CDCl_3$): δ (ppm) = 7.38−7.29 (m, 5H), 7.17 (d, *J* = 8.3 Hz, 2H), 6.84 (dt, J_1 = 2.6 Hz, J_2 = 8.8 Hz, 2H), 4.54 (d, *J* = 8.8 Hz, 1H), 3.97 (dd, A of ABX, J_{AB} = 13.2 Hz, J_{AX} = 4.2 Hz, 1H), 3.78 (s, 3H), 3.64 (dd, B of ABX, J_{AB} = 13.9 Hz, J_{AX} = 8.1 Hz, 1H), 2.97−2.90 (m, 1H); (^{13}C NMR, 101 MHz, $CDCl_3$): δ (ppm) = 156.0, 153.6, 139.9, 135.8, 128.9, 128.4, 127.8, 126.3 (q, *J* = 281.3 Hz), 122.8, 114.2, 55.4, 46.2 (q, *J* = 23.8 Hz), 45.3, 44.8; (^{19}F NMR, 377 MHz, $CDCl_3$): δ (ppm) = −68.4 (d, *J* = 6.9 Hz, 3F); IR: 2933, 1625, 1507, 1456, 1382, 1240, 1168, 1117, 1035, 832 cm^{-1}; HRMS (ESI) for $C_{18}H_{18}ON_2F_3S$ $[M+H]^+$ requires 367.1087 found 367.1086; Mp. 140−142 °C.

(5*R,6*S**)-6-Phenyl-5-(trifluoromethyl)-*N*-(4-(trifluoromethyl)phenyl)-5,6-dihydro-4H-1,3-thiazin-2-amine (2nd)**

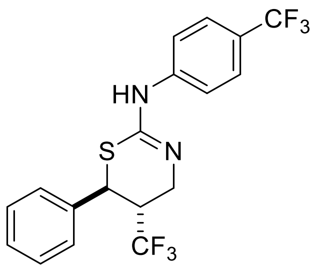

The title compound was prepared following general procedure H using 1-cinnamyl-3-(4-(trifluoromethyl)phenyl)thiourea (100.9 mg, 0.3 mmol), 1-trifluoromethyl-1,2-benziodoxol-3-(1H)-one (113.8 mg, 0.36 mmol), 4Å MS (30 mg), trifluoroacetic acid (45.9 µL, 0.6 mmol) and $CDCl_3$ (3.0 mL). The crude product was purified by silica gel column chromatography (eluent: 1% Et_3N and 10 % EtOAc in *n*-pentane) to provide the title compound (49.3 mg, 41 % yield) as a white solid.

(^{1}H NMR, 400 MHz, $CDCl_3$): δ (ppm) = 7.53 (d, J = 8.3 Hz, 2H), 7.39−7.33 (m, 7H), 4.58 (d, J = 8.8 Hz, 1H), 3.99 (dd, A of ABX, J_{AB} = 13.9 Hz, J_{AX} = 4.2 Hz, 1H), 3.68 (dd, A of ABX, J_{AB} = 13.9 Hz, J_{AX} = 7.6 Hz, 1H), 3.02−2.95 (m, 1H); (^{13}C NMR, 101 MHz, $CDCl_3$): δ (ppm) = 153.0, 146.3, 139.3, 129.1, 128.7, 127.8, 126.3, 126.2 (q, J = 282.1 Hz), 126.2, 124.3 (q, J = 271.0 Hz), 120.3, 46.2 (q, J = 25.4 Hz), 45.6, 44.4; (^{19}F NMR, 377 MHz, $CDCl_3$): δ (ppm) = −61.8 (s, 3F), −68.5 (d, J = 6.9 Hz, 3F); IR: 2927, 1631, 1598, 1525, 1456, 1410, 1382, 1249, 1162, 1111, 1064, 1014, 844 cm^{-1}; HRMS (ESI) for $C_{18}H_{15}N_2F_6S$ $[M+H]^+$ requires 405.0855 found 405.0851; Mp. 87−88 °C.

(5*R,6*S**)-*N*-(4-Fluorophenyl)-6-phenyl-5-(trifluoromethyl)-5,6-dihydro-4H-1,3-thiazin-2-amine (2ne)**

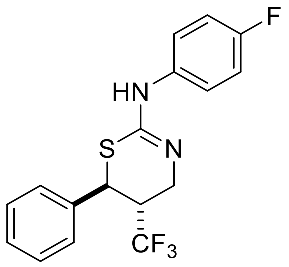

The title compound was prepared following general procedure H using 1-cinnamyl-3-(4-fluorophenyl)thiourea (85.9 mg, 0.3 mmol), 1-trifluoromethyl-1,2-benziodoxol-3-(1H)-one (113.8 mg, 0.36 mmol), 4Å MS (30 mg), trifluoroacetic acid (45.9 µL, 0.6 mmol) and $CDCl_3$ (3.0 mL). The crude product was purified by silica gel column chromatography (eluent: 1%

1% Et_3N and 10 % EtOAc in *n*-pentane) to provide the title compound (47.8 mg, 45 % yield) as a white solid.

(^{1}H NMR, 400 MHz, $CDCl_3$): δ (ppm) = 7.38−7.30 (m, 5H), 7.17 (dd, *J* = 8.1, 4.7 Hz, 2H), 7.00−6.94 (m, 2H), 4.55 (d, *J* = 9.1 Hz, 1H), 3.94 (dd, A of ABX, J_{AB} = 13.9 Hz, J_{AX} = 4.7 Hz, 1H), 3.64 (dd, B of ABX, J_{AB} = 13.9 Hz, J_{AX} = 7.6 Hz, 1H), 3.03−2.94 (m, 1H); (^{13}C NMR, 101 MHz, $CDCl_3$): δ (ppm) = 159.0 (d, *J* = 241.6 Hz), 153.6, 139.5, 139.2, 129.0, 128.5, 127.7, 126.2 (q, *J* = 282.1 Hz), 122.5 (d, *J* = 8.0 Hz), 115.5 (d, *J* = 23.1 Hz), 46.4 (q, *J* = 24.6 Hz), 45.4, 44.2; (^{19}F NMR, 377 MHz, $CDCl_3$): δ (ppm) = −68.6 (d, *J* = 8.7 Hz, 3F), −119.9 (br s , 1F); IR: 2981, 1625, 1504, 1456, 1382, 1305, 1249, 1215, 1169, 1119, 837 cm^{-1}; HRMS (ESI) for $C_{17}H_{15}N_2F_4S$ $[M+H]^+$ requires 355.0887 found 355.0884; Mp. 131−132 °C.

(6*S,7*S**)-*N*-Phenyl-7-(trifluoromethyl)-1-thia-3-azaspiro[5.5]undec-2-en-2-amine (2ra)**

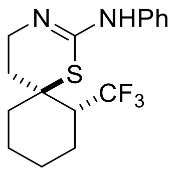

The title compound was prepared following general procedure H using 1-(2-(cyclohex-1-en-1-yl)ethyl)-3-(4-methoxybenzyl)thiourea (92.0 mg, 0.3 mmol), 1-trifluoromethyl-1,2-benziodoxol-3-(1H)-one (113.8 mg, 0.36 mmol), 4Å MS (30 mg), trifluoroacetic acid (45.9 µL, 0.6 mmol) and $CDCl_3$ (3.0 mL). The crude product (*d.r.* = 8:1) was purified by silica gel column chromatography (eluent: 1% 1% Et_3N and 25 % EtOAc in *n*-pentane) to provide the title compound (58.7 mg, 60 % yield) as a white solid (*d.r.* = 20:1).

(^{1}H NMR, 400 MHz, $CDCl_3$): δ (ppm) = 7.18 (t, *J* = 7.8 Hz, 2H), 7.12 (br s , 2H), 6.93−6.89 (m, 1H), 3.72-3.34 (m, 2H), 2.49−2.44 (m, 1H), 2.03−1.72 (m, 6H), 1.63−1.40 (m, 4H); (^{13}C NMR, 101 MHz, $CDCl_3$): δ (ppm) = 149.2, 143.4, 128.8, 127.0 (q, *J* = 283.9 Hz), 122.5, 120.8, 50.0, 48.3 (q, *J* = 23.5 Hz), 41.6, 37.0, 31.4, 22.5 (q, *J* = 2.9 Hz), 21.7, 21.2; (^{19}F NMR, 377 MHz, $CDCl_3$): δ (ppm) = −60.3 (br s , 3F); IR: 2942, 2361, 1624, 1586, 1518, 1496, 1436, 1375, 1342, 1309, 1276, 1238, 1199, 1167, 1138, 1091, 1069, 1037, 987, 899, 832 cm^{-1}; HRMS (ESI) for $C_{16}H_{20}N_2F_3S$ $[M+H]^+$ requires 329.1294 found 329.1290; Mp. 98−99 °C.

(6S*,7S*)-N-(4-methoxybenzyl)-7-(trifluoromethyl)-1-thia-3-azaspiro[5.5]undec-2-en-2-amine (2rb)

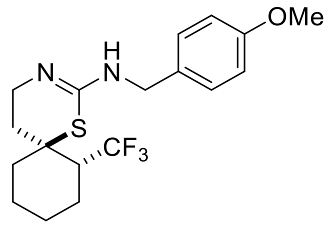

The title compound was prepared following general procedure H using 1-(2-(cyclohex-1-en-1-yl)ethyl)-3-(4-methoxybenzyl)thiourea (91.0 mg, 0.3 mmol), 1-trifluoromethyl-1,2-benziodoxol-3-(1H)-one (113.8 mg, 0.36 mmol), 4Å MS (30 mg), trifluoroacetic acid (45.9 µL, 0.6 mmol) and $CDCl_3$ (3.0 mL) for 2h. The crude product (*d.r.* = 6:1) was purified by silica gel column chromatography (eluent: 1% Et_3N and 10 % Et_2O in DCM) to provide the title compound (58.7 mg, 47 % yield) as a yellow oil (*d.r.* = 6:1).

(^{1}H NMR, 400 MHz, $CDCl_3$): δ (ppm) = 7.23 (d, *J* = 8.6 Hz, 2H), 6.86 (d, *J* = 8.6 Hz, 2H), 4.33 (s, 2H), 3.80 (s, 3H), 3.80-3.72 (m, 1H), 3.62-3.54 (m, 1H), 2.60-2.44 (m, 1H), 2.11-1.43 (m, 1H); (^{13}C NMR, 101 MHz, $CDCl_3$): δ (ppm) (major diastereoisomer) = 158.9, 150.1, 130.9, 128.1, 127.1 (q, *J* = 282.5 Hz), 55.2, 49.7, 48.6 (q, *J* = 23.3 Hz), 45.8, 43.4, 37.0, 31.2, 22.5, 21.5, 21.1; (^{19}F NMR, 377 MHz, $CDCl_3$): δ (ppm) = −60.3 (br s , 3F); IR: 3219, 2980, 2360, 1613, 1586, 1299, 1167, 1135, 1090 cm^{-1}; HRMS (ESI) for $C_{18}H_{24}ON_2F_4S$ $[M+H]^+$ requires 373.1554 found 373.1556;

(6S*,7S*)-7-(trifluoromethyl)-1-thia-3-azaspiro[5.5]undec-2-en-2-amine (2rc)

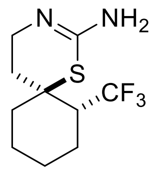

The title compound was prepared following general procedure H using 1-(2-(cyclohex-1-en-1-yl)ethyl)-3-(4-methoxybenzyl)thiourea (91.0 mg, 0.3 mmol), 1-trifluoromethyl-1,2-benziodoxol-3-(1H)-one (113.8 mg, 0.36 mmol), 4Å MS (30 mg), trifluoroacetic acid (45.9 µL, 0.6 mmol) and $CDCl_3$ (3.0 mL) for 2h. The crude product was filtered through a plug of silica and the solvents was removed in *vacuo*. The resulting oil was dissolved in CH_3CN (10 ml) and a solution of ceric ammonium nitrate (1.62 g, 3 mmol) in 2 ml of water was added. After 20 minutes, a solution of saturated $NaHCO_3$ was added, followed by EtOAc (40 ml). After extraction with AcOEt (2 x 20 ml), the combined organic phases were dried over $MgSO_4$ and

the solvent was removed by rotavapor. The crude product (*d.r.* = 6:1) was purified by silica gel column chromatography (eluent: 1% Et_3N and 10 % Et_2O in DCM) to provide the title compound (22.1 mg, 34 % yield) as a yellow oil (*d.r.* = 5:1).
(^1H NMR, 400 MHz, $CDCl_3$): δ (ppm) = 4.02-3.71 (br s , 2H), 3.68-3.48 (m, 2H), 2.54–2.40 (m, 1H), 2.10-1.32 (m, 10H); major diastereoisomer: (^{13}C NMR, 101 MHz, $CDCl_3$): δ (ppm) = 151.0, 127.0 (q, *J* = 284.3 Hz), 53.0, 49.4, 48.0 (q, *J* = 23.5 Hz), 42.9, 36.7, 30.5, 22.3 (q, *J* = 2.9 Hz), 21.6, 21.0; (^{19}F NMR, 377 MHz, $CDCl_3$): δ (ppm) = −60.3 (br s , 3F); IR: 2981, 2364, 1612, 1546, 1348, 1312, 1245, 1090, 941, 871 cm^{-1}; HRMS (ESI) for $C_{10}H_{16}N_2F_3S$ $[M+H]^+$ requires 253.0981 found 253.0980;

7-Fluoro-*N*-phenyl-4-(2,2,2-trifluoroethyl)-4,5-dihydrobenzo[d][1,3]thiazepin-2-amine (2sa)

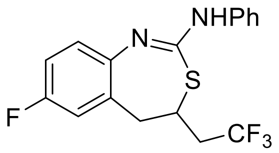

The title compound was prepared following general procedure H using 1-(2-allyl-4-fluorophenyl)-3-phenylthiourea (85.9 mg, 0.3 mmol), 1-trifluoromethyl-1,2-benziodoxol-3-(1H)-one (113.8 mg, 0.36 mmol), 4Å MS (30 mg), trifluoroacetic acid (45.9 µL, 0.6 mmol) and $CDCl_3$ (3.0 mL). The crude product was purified by silica gel column chromatography (eluent: 20 % Et_2O in *n*-pentane) to provide the title compound (62.3 mg, 59 % yield) as a pale yellow resin.
(^1H NMR, 400 MHz, $CDCl_3$): δ (ppm) = 7.73 (d, *J* = 7.6 Hz, 2H), 7.37 (t, *J* = 8.1 Hz, 2H), 7.12 (t, *J* = 7.5 Hz, 1H), 7.05−7.00 (m, 2H), 6.93 (dd, *J* = 8.6, 2.5 Hz, 1H), 4.21−4.14 (m, 1H), 3.07 (dd, A of ABX, J_{AB} = 13.7 Hz, J_{AX} = 5.4 Hz, 1H), 2.82 (dd, A of ABX, J_{AB} = 13.7 Hz, J_{AX} = 8.9 Hz, 1H), 2.73−2.52 (m, 2H); (^{13}C NMR, 101 MHz, $CDCl_3$): δ (ppm) = 158.8 (d, *J* = 242.4 Hz), 146.0, 144.4, 139.7, 130.6 (d, *J* = 7.2 Hz), 129.0, 125.4 (q, *J* = 278.2 Hz), 125.1 (d, *J* = 8.0 Hz), 123.6, 119.5, 115.9 (d, *J* = 22.3 Hz), 114.8 (d, *J* = 22.3 Hz), 49.2, 41.4 (q, *J* = 27.8 Hz), 37.1; (^{19}F NMR, 377 MHz, $CDCl_3$): δ (ppm) = −63.4 (t, *J* = 10.4 Hz, 3F), −119.9 (q, *J* = 7.5 Hz, 1F); IR: 3424, 1619, 1593, 1512, 1486, 1435, 1380, 1345, 1312, 1262, 1212, 1136, 1101, 1006, 964, 873, 829 cm^{-1}; HRMS (ESI) for $C_{17}H_{15}N_2F_4S$ $[M+H]^+$ requires 355.0887 found 355.0883.

(1*S,2*S**,*Z*)-3'-((4-Nitrophenyl)sulfonyl)-N-phenyl-2-(trifluoromethyl)-3,4-dihydro-2H-spiro[naphthalene-1,5'-thiazolidin]-2'-imine (3)**

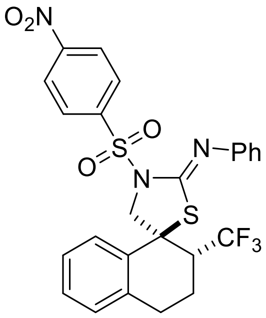

4-nitrobenzenesulfonyl chloride (58.9 mg, 0.27 mmol) and 4-dimethylaminopyridine (21.6 mg, 0.18 mmol) were added to a solution of (1*S**,2*S**)-*N*-phenyl-2-(trifluoromethyl)-3,4-dihydro-2H,4'H-spiro[naphthalene-1,5'-thiazol]-2'-amine (64.1 mg, 0.18 mmol) in DCM (1 mL). The reaction mixture was stirred at room temperature for 16 hours. The crude mixture was concentrated *in vacuo* and purified by silica gel column chromatography (eluent: 5 % EtOAc in *n*-pentane) to provide the title compound (19.0 mg, 20 % yield) as a white solid.
(^{1}H NMR, 400 MHz, $CDCl_3$): δ (ppm) = 8.49−8.41 (m, 4H), 7.77−7.75 (m, 1H), 7.32−7.10 (m, 4H), 7.15−7.10 (m, 2H), 6.77−6.74 (m, 2H), 4.46 (d, A of AB, J_{AB} = 16.5 Hz, 1H), 4.41 (d, B of AB, J_{AB} = 16.5 Hz, 1H), 3.06−2.85 (m, 2H), 2.89 (dt, J_1 = 6.4 Hz, J_2 = 17.4 Hz, 1H), 2.24 (q, J = 6.4 Hz, 2H); (^{13}C NMR, 126 MHz, $CDCl_3$): δ (ppm) = 150.8, 150.2, 148.9, 143.4, 136.0, 134.6, 133.2, 130.9, 129.4, 129.1, 129.1 (q, J = 282.4 Hz), 128.6, 127.3, 126.4, 124.9, 123.7, 120.7, 57.4, 54.1, 48.2 (q, J = 24.9 Hz), 26.0, 21.3; (^{19}F NMR, 377 MHz, $CDCl_3$): δ (ppm) = −63.8 (d, J = 8.7 Hz, 3F); IR: 2920, 1645, 1592, 1532, 1488, 1349, 1313, 1264, 1177, 1120, 1095, 1044, 1014, 909, 855 cm^{-1}; HRMS (ESI) for $C_{25}H_{21}O_4N_3F_3S_2$ $[M+H]^+$ requires 548.0920 found 548.0915; Mp. >250 (decomp.).

4-Nitro-*N*-phenyl-*N*-((1*S,2*S**)-2-(trifluoromethyl)-3,4,4',5'-tetrahydro-2H-spiro[naphthalene-1,6'-[1,3]thiazin]-2'-yl)benzenesulfonamide (4)**

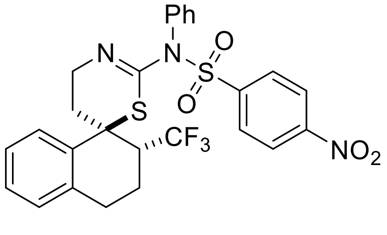

To the solution of (1*S**,2*S**)-*N*-phenyl-2-(trifluoromethyl)-3,4,4',5'-tetrahydro-2H-spiro[naphthalene-1,6'-[1,3]thiazin]-2'-amine (27.3 mg, 0.073 mmol) in DCM (0.4 mL) was

added 4-nitrobenzenesulfonyl chloride (24.1 mg, 0.109 mmol) and 4-dimethylaminopyridine (8.9 mg, 0.073 mmol). The reaction mixture was stirred at room temperature for 16 hours. The crude mixture was concentrated *in vacuo* and purified by silica gel column chromatography (eluent: 10 % EtOAc in *n*-pentane) to provide the title compound (29.6 mg, 73 % yield) as a white solid.

(^{1}H NMR, 400 MHz, $CDCl_3$): δ (ppm) = 8.30 (dt, *J* = 8.8, 2.2 Hz, 2H), 8.00 (dt, *J* = 9.1, 2.1 Hz, 2H), 7.43−7.35 (m, 4H), 7.25−7.23 (m, 2H), 7.19−7.14 (m, 2H), 7.04−7.02 (m, 1H), 4.21−4.02 (m, 2H), 2.93−2.82 (m, 2H), 2.77−2.65 (m, 2H), 2.36−2.25 (m, 2H), 2.18−2.11 (m, 1H); (^{13}C NMR, 126 MHz, $CDCl_3$): δ (ppm) = 150.2, 145.4, 136.1, 135.9, 135.8, 130.4, 129.6, 129.6, 129.3, 129.3, 127.9, 126.8, 126.7 (q, J = 284.6 Hz) 126.5, 123.4, 120.5, 50.9, 45.7 (q, *J* = 2.9 Hz), 45.1 (q, *J* = 23.5 Hz), 28.3, 25.1, 19.8 (q, *J* = 2.9 Hz); (^{19}F NMR, 377 MHz, $CDCl_3$): δ (ppm) = −61.5 (d, *J* = 9.2 Hz, 3F); IR: 2931, 1647, 1531, 1488, 1454, 1402, 1368, 1349, 1313, 1262, 1175, 1119, 1087, 1017, 953, 910, 855 cm^{-1}; HRMS (ESI) for $C_{26}H_{23}O_4N_3F_3S_2$ $[M+H]^+$ requires 562.1077 found 562.1076; Mp. 141−142 °C.

3. Mechanistic Experiments.

3.1. Thiotrifluoromethylation of 1aa with TEMPO .

To a vial containing appropriate allyl thiourea **1aa** (0.1 mmol, 19.2 mg), 1-trifluoromethyl-1,2-benziodoxol-3-(1H)-one (37.9 mg, 0.12 mmol), TEMPO (1 equiv.), 4Å MS (10 mg) and a stirrer bar, was added a solution of trifluoroacetic acid (15.3 µL, 0.2 mmol) in $CDCl_3$ (3.0 mL) under Ar atmosphere. The vial was sealed and allowed to stir at room temperature for 24 hours. The reaction mixture was filtered through cotton and analysed by 19F NMR, using $C_6H_5CF_3$ as internal standard (1 equiv.)

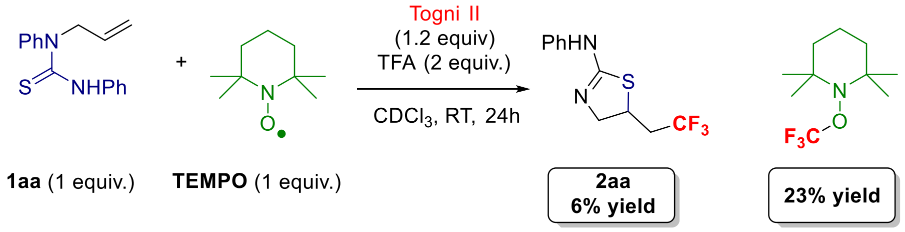

^{19}F NMR spectrum of the crude reaction is reported below:

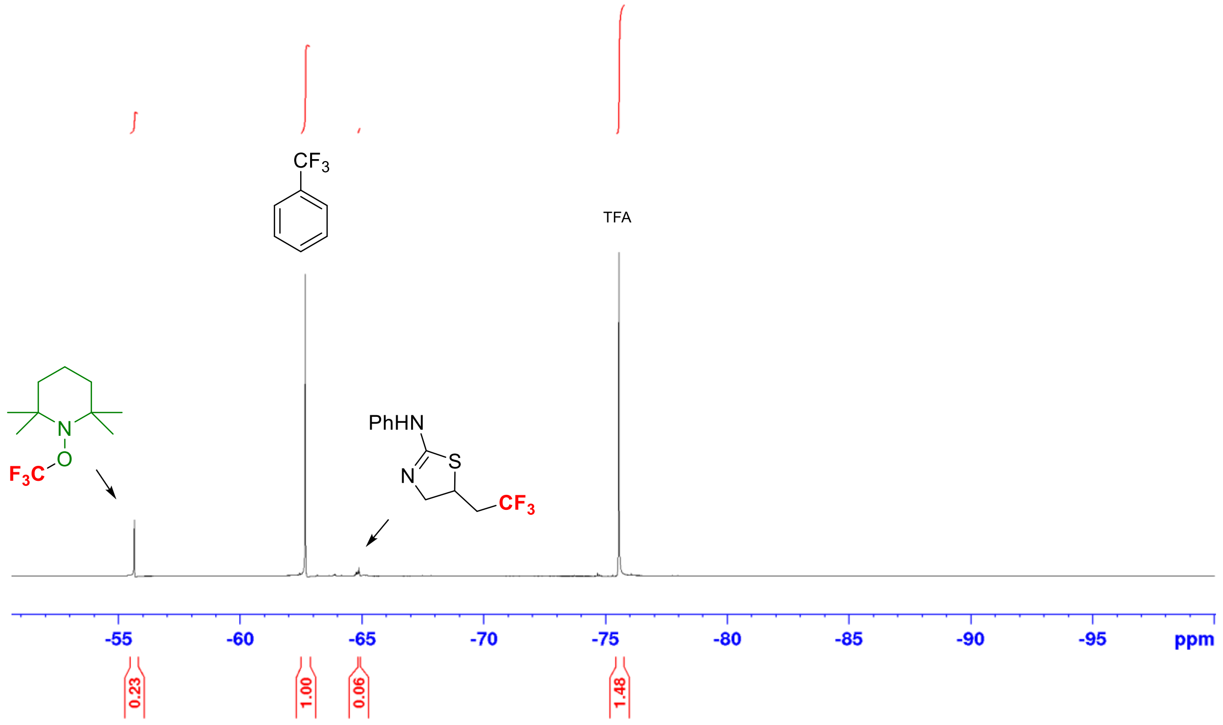

3.2. Thiotrifluoromethylation of Diethyl 2,2-Diallyl Malonate.

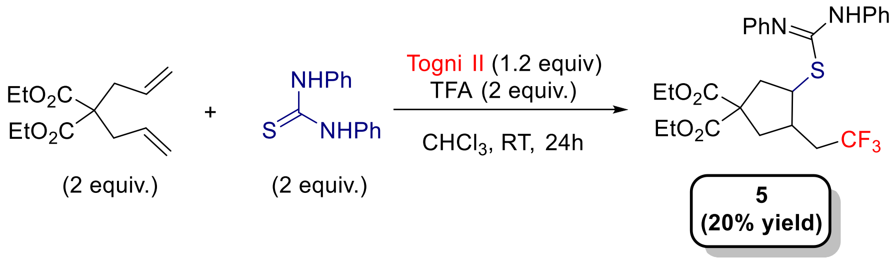

The title compound was prepared following general procedure H using diethyl diallyl malonate (144 mg, 0.6 mmol), diphenylthiourea (136.8 mg, 0.6 mmol), 1-trifluoromethyl-1,2-benziodoxol-3-(1H)-one (113.8 mg, 0.36 mmol), trifluoroacetic acid (45.9 µL, 0.6 mmol) and $CDCl_3$ (3.0 mL). The crude product was purified by silica gel column chromatography (eluent: 15 % EtOAc in *n*-pentane) to provide the title compound (31.7 mg, 20 % yield) as a colourless oil.

Diethyl-3-(((N,N'-diphenylcarbamimidoyl)thio)methyl)-4-(2,2,2-trifluoroethyl) cyclopentane-1,1-dicarboxylate (5)

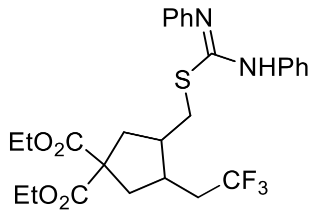

(^{1}H NMR, 400 MHz, CD_3OD): δ (ppm) = 7.16 (t, *J* = 7.8 Hz, 6H), 7.11 (br s, 1H), 6.91 (t, *J* = 7.5 Hz, 4H), 4.06 (q, *J* = 7.1 Hz, 2H), 4.03 (q, *J* = 7.1 Hz, 2H), 2.44−1.76 (m, 10H), 1.11 (t, J = 7.1 Hz, 3H), 1.10 (t, J = 7.1 Hz, 3H); (^{13}C NMR, 101 MHz, CD_3OD): δ (ppm) = 172.2, 172.1, 150.3, 128.4, 127.2 (q, *J* = 276.6 Hz), 122.8, 121.1 (br), 61.5, 61.4, 58.4, 42.0, 37.8, 37.4, 36.0 (q, *J* = 2.2 Hz), 32.7 (q, *J* = 27.9 Hz), 31.3, 12.9; (^{19}F NMR, 377 MHz, CD_3OD): δ (ppm) = −65.8 (t, *J* = 11.5 Hz, 3F); IR: 2982, 2938, 1728, 1627, 1587, 1525, 1515, 1498, 1485, 1437, 1392, 1368, 1312, 1255, 1220, 1184, 1149, 1128, 1095, 1055, 1027, 1007, 914, 861, 838 cm^{-1} ; HRMS (ESI) for $C_{27}H_{32}O_4N_2F_3S$ $[M+H]^+$ requires 537.2029 found 537.2022.

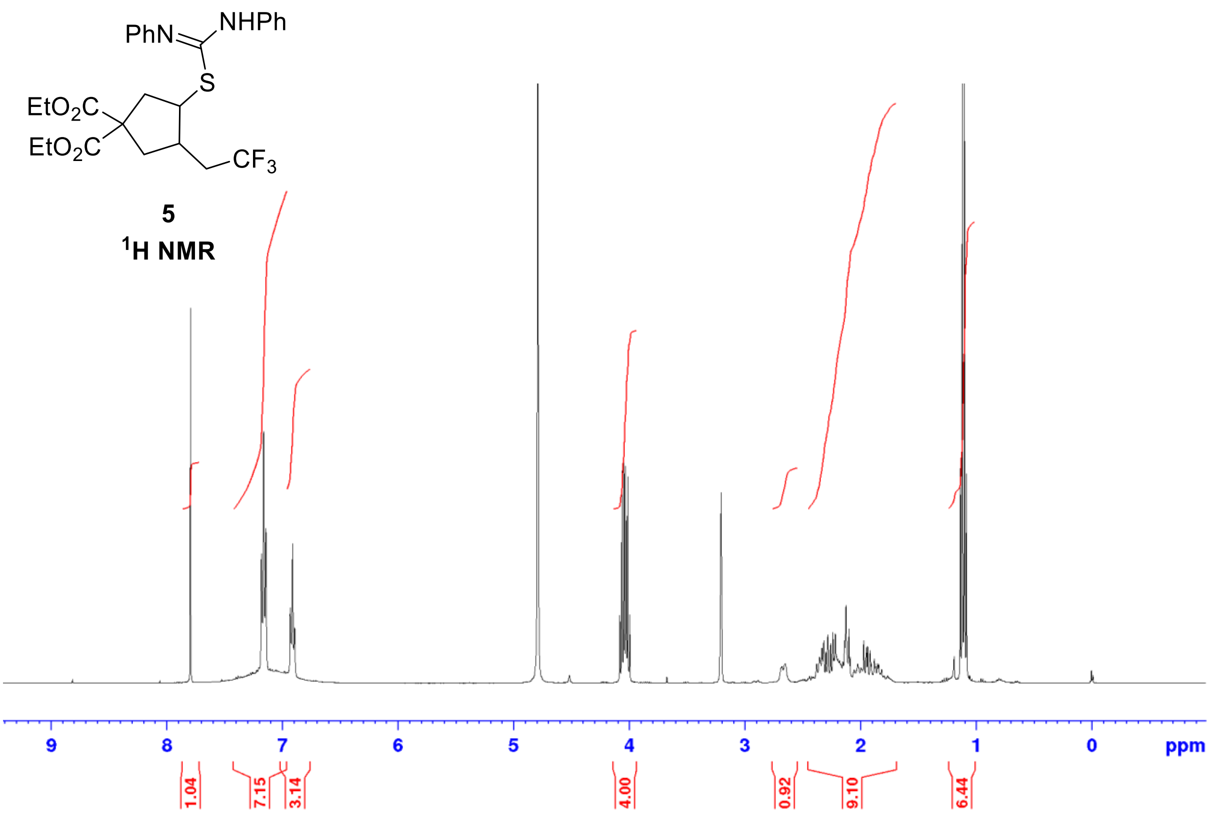

5

^{1}H NMR

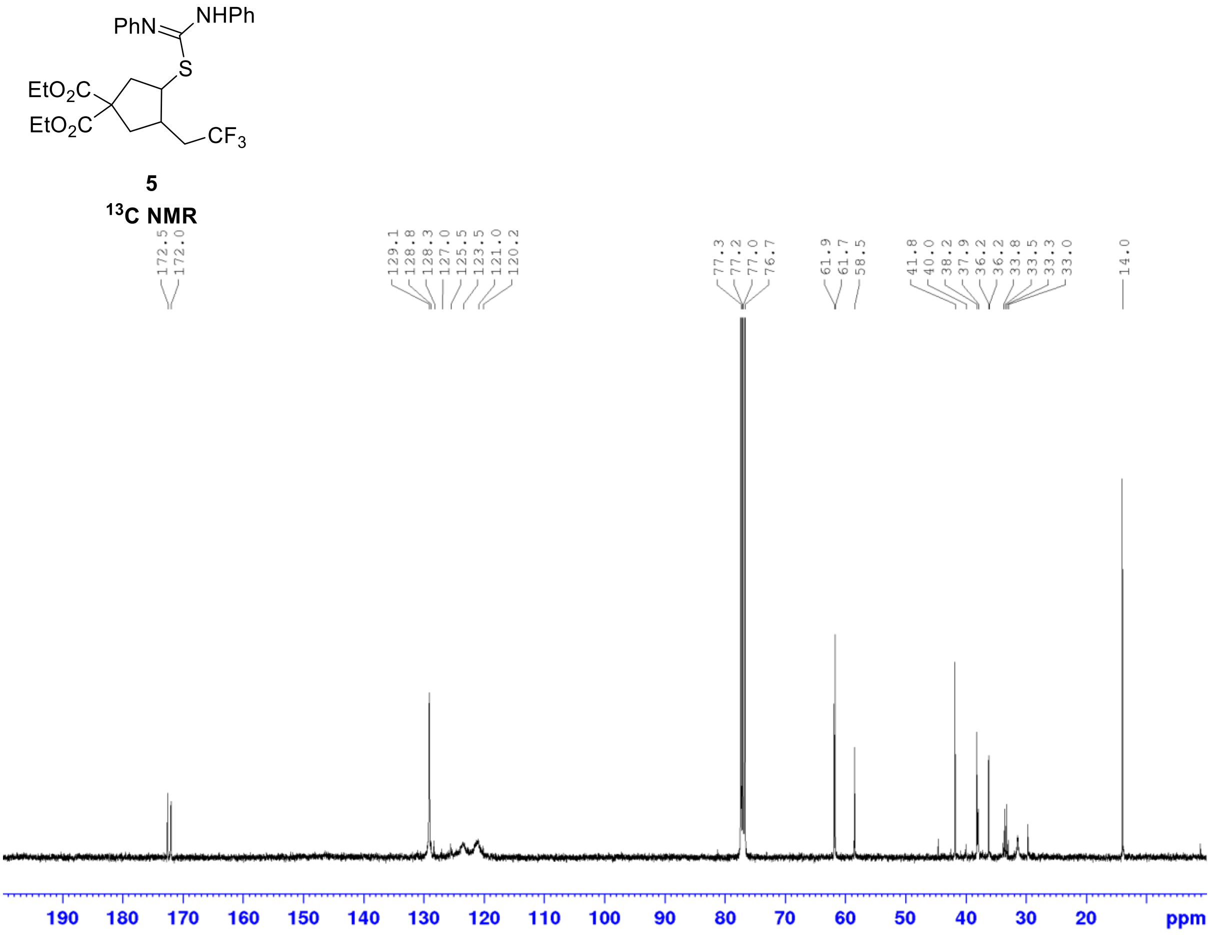

5

^{13}C NMR

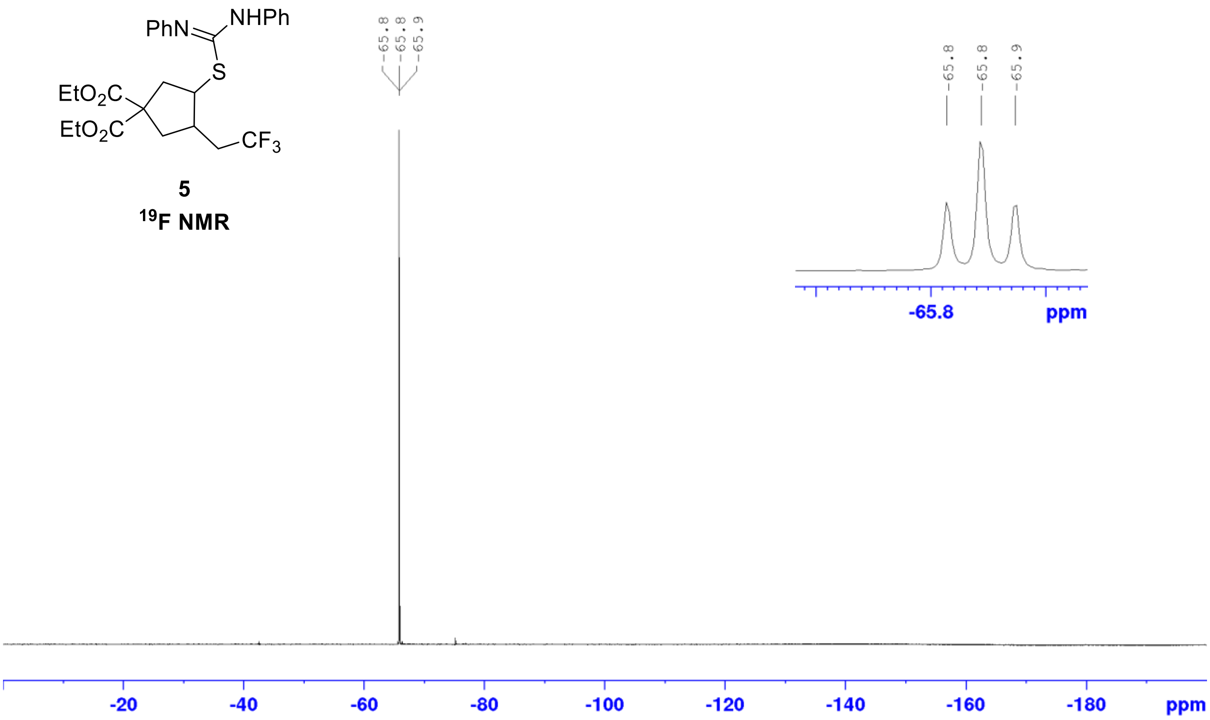

3.3 Thiotrifluoromethylation of Urea 6 and Amide 8.

1-((3,4-Dihydronaphthalen-1-yl)methyl)-3-phenylurea (6)

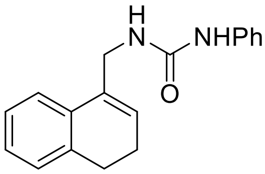

The title compound was prepared following general procedure A using (3,4-dihydronaphthalen-1-yl)methanamine (392 mg, 2.0 mmol), DCM (20 mL) and phenyl isocyanate (0.22 mL, 2.0 mmol). The crude product was purified by precipitation to give the title compound (374 mg, 67 % yield) as a white solid.

(^{1}H NMR, 400 MHz, DMSO-d6): δ (ppm) = 7.41 (d, *J* = 7.6 Hz, 2H), 7.30 (d, *J* = 6.9 Hz, 1H), 7.25−7.18 (m, 5H), 6.90 (t, *J* = 6.6 Hz, 1H), 6.29 (br s , 1H), 6.05 (br s , 1H), 4.15 (br s , 2H), 2.71 (t, *J* = 7.3 Hz, 2H), 2.25 (br s , 2H); (^{13}C NMR, 101 MHz, DMSO-d6): δ (ppm) = 154.9, 140.4, 136.0, 134.0, 133.2, 128.7, 127.6, 127.0, 126.5, 126.0, 122.5, 121.0, 117.5, 40.9, 27.5, 22.4; IR: 3295, 1631, 1596, 1568, 1527, 1495, 1442, 1295, 1264, 1236, 1170, 1053, 1019, 819 cm^{-1}; HRMS (ESI) for $C_{18}H_{19}ON_2$ [M+H]$^+$ requires 279.1492 found 279.1493; Mp. 148−150 °C.

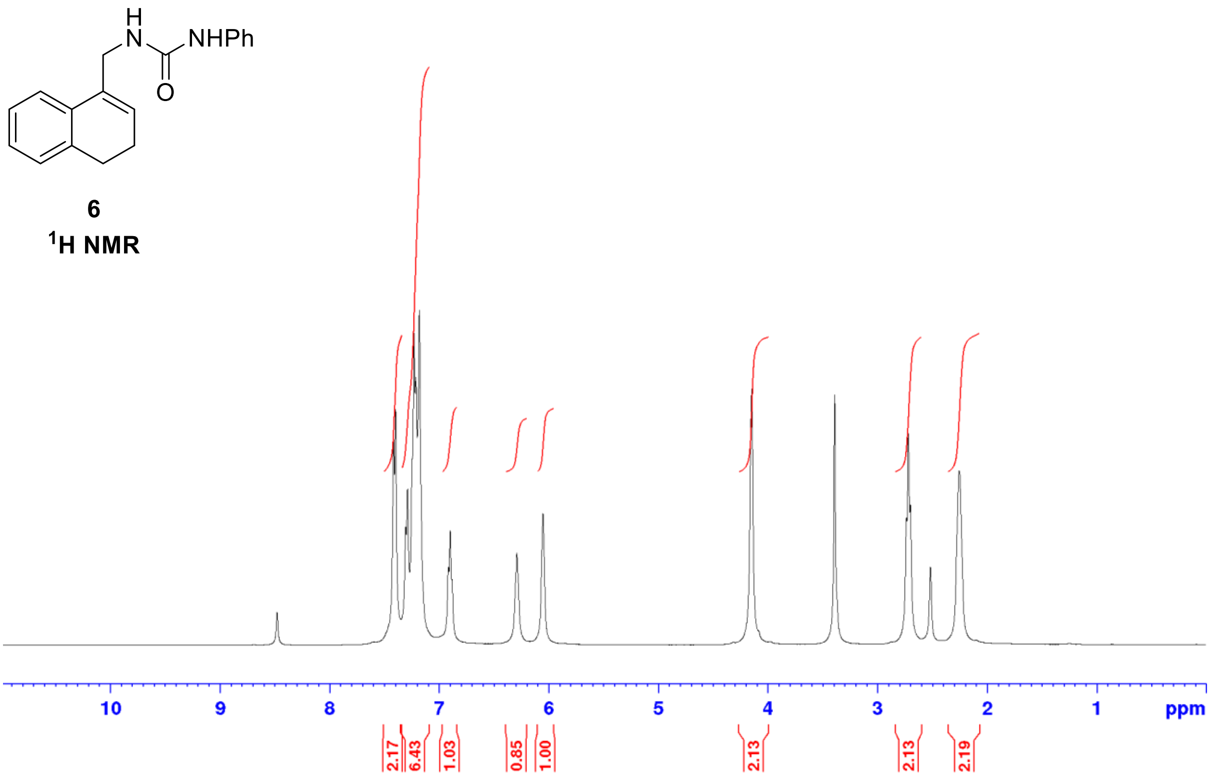

6
^{1}H NMR

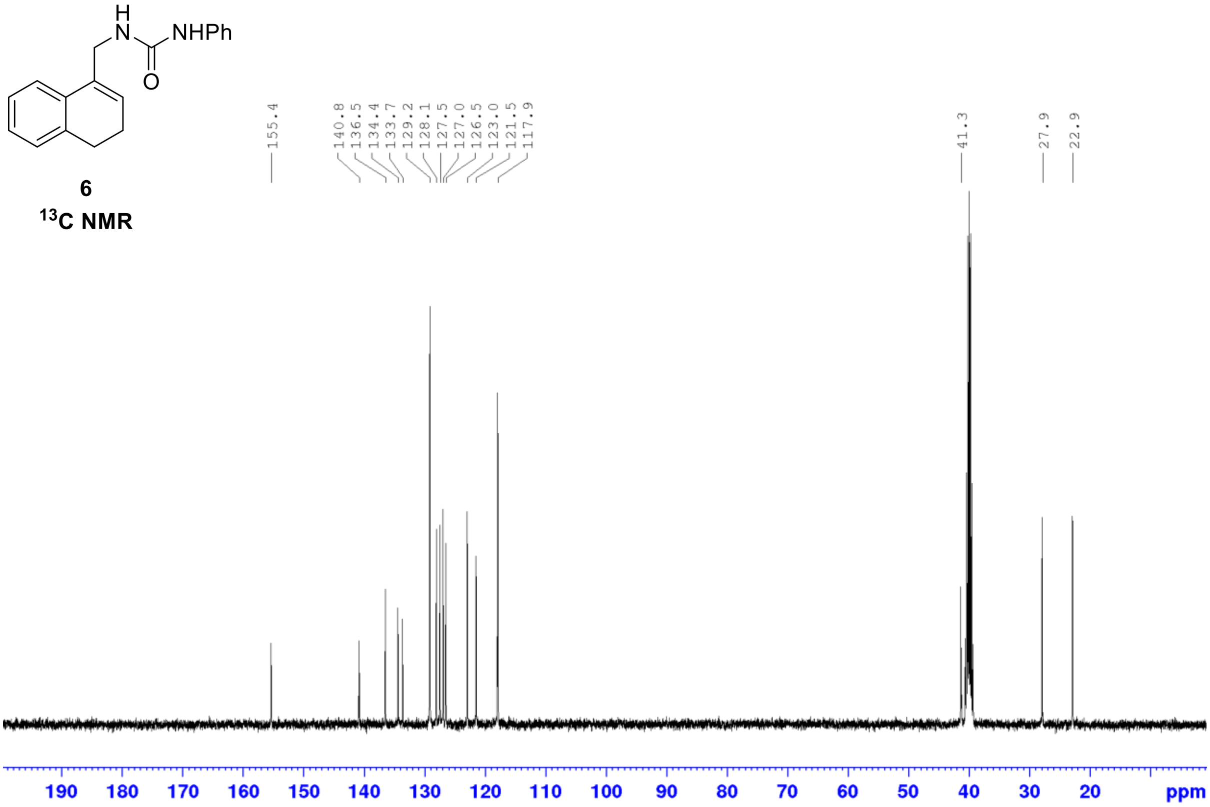

6
^{13}C NMR

(1*S,2*R**)-*N*-Phenyl-2-(trifluoromethyl)-3,4-dihydro-2H,4'H-spiro[naphthalene-1,5'-oxazol]-2'-amine (7)**

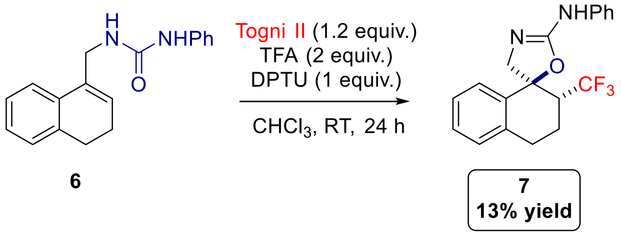

(eq. 3)

Into a vial containing 1-((3,4-dihydronaphthalen-1-yl)methyl)-3-phenylurea (139 mg, 0.5 mmol) (4), *N*,*N'*-diphenylthiourea (114 mg, 0.5 mmol), 1-trifluoromethyl-1,2-benziodoxol-3-(1H)-one (190 mg, 0.6 mmol) and a stirrer bar was added a solution of trifluoroacetic acid (76.6 µL, 1.0 mmol) in $CHCl_3$ (5.0 mL) under Ar atmosphere. The vial was sealed and allowed to stir at room temperature for 24 hours. The reaction mixture was then concentrated and purified by silica gel column chromatography (eluent: 25 % EtOAc in *n*-pentane) to provide the title compound (24.8 mg, 14 % yield) as a pale yellow resin.

(^1H NMR, 500 MHz, $CDCl_3$): δ (ppm) = 7.45 (d, *J* = 6.9 Hz, 3H), 7.32–7.26 (m, 5H), 7.13 (d, *J* = 6.6 Hz, 1H), 7.02 (t, *J* = 7.3 Hz, 1H), 4.50 (d, *J* = 13.3 Hz, 1H), 3.94 (d, *J* = 13.3 Hz, 1H), 2.99–2.87 (m, 3H), 2.33–2.28 (m, 1H), 1.98–1.90 (m, 1H); (^{13}C NMR, 158 MHz, $CDCl_3$): δ (ppm) = 155.7, 140.0, 139.1, 134.4, 129.0, 128.4, 128.2, 127.4, 126.7 (q, *J* = 281.3 Hz), 125.3, 122.5, 118.3, 84.0, 63.4, 46.5 (q, *J* = 25.8 Hz), 27.9, 20.8 (q, *J* = 2.9 Hz); (^{19}F NMR, 377 MHz, $CDCl_3$): δ (ppm) = −66.6 (d, *J* = 9.0 Hz, 3F); IR: 3063, 2940, 2360, 1676, 1601, 1555, 1499, 1448, 1416, 1389, 1342, 1310, 1269, 1247, 1229, 1188, 1167, 1138, 1112, 1091, 1038, 993, 956, 909, 832 cm^{-1} ; HRMS (CI) for $C_{19}H_{18}ON_2F_3$ $[M+H]^+$ requires 347.1371 found 347.1370.

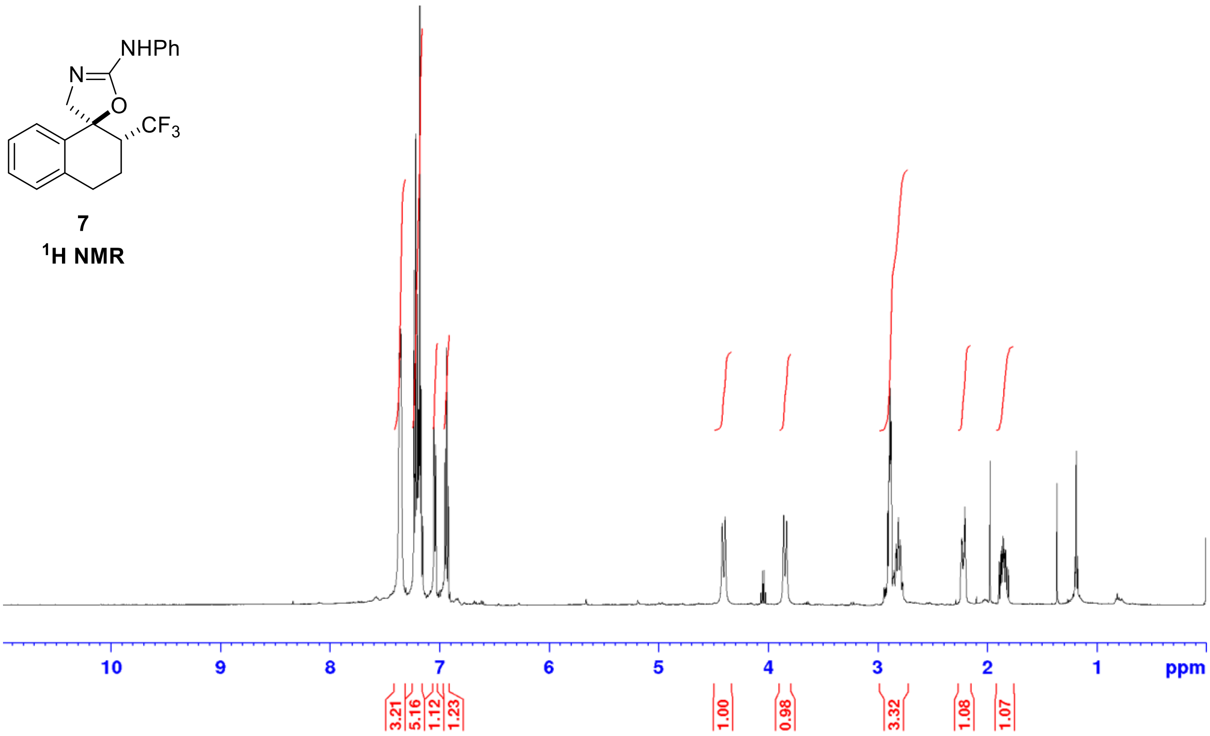

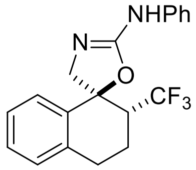

7

^{13}C NMR

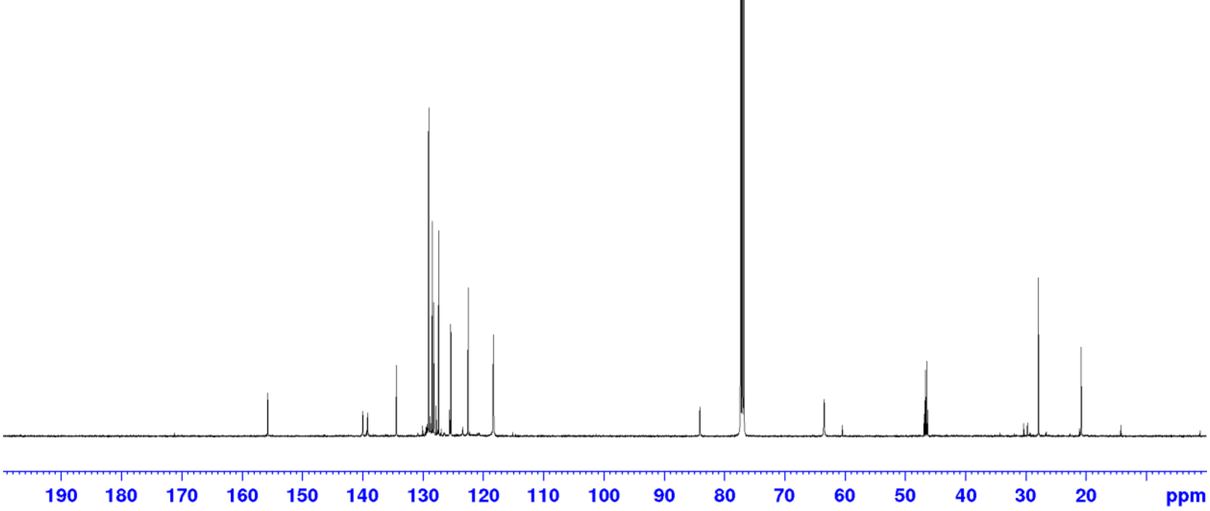

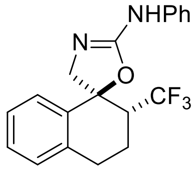

7

^{19}F NMR

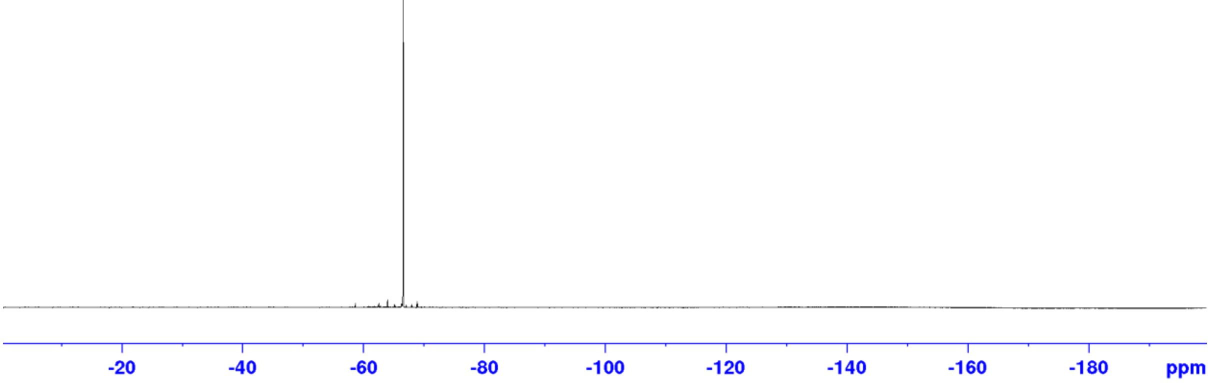

N-((3,4-Dihydronaphthalen-1-yl)methyl)benzamide (8)

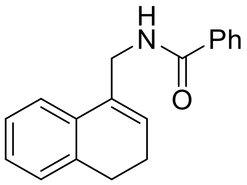

The title compound was prepared following a previously reported procedure.[15] To a mixture of (1,2-Dihydronaphthalen-4-yl)methanammonium hydrochloride (392 mg, 2.0 mmol), Et_3N (1.12 ml, 8 mmol) and DMAP (24.4 mg, 0.2 mmol), benzoyl chloride (0.23 ml, 2 mmol) was added dropwise at 0 °C. The mixture was stirred for 3h at room temperature. The reaction was quenched by the addition of saturated NH_4Cl aq (50 mL). The aqueous phase was extracted with CH_2Cl_2 (50 mL × 3). The organic phase was combined and reduced to a volume of approximate 20 mL. The solution was washed with K_2CO_3 aq (50 mL) and the aqueous phase was extracted with CH_2Cl_2 (50 mL × 3). The combined organic phase was dried over $MgSO_4$, filtered and concentrated. The crude product was purified by flash column chromatography (25% EtOAc in hexane) to provide the title compound (390 mg, 70% yield) as a white solid. Data consistent with literature values.[11]

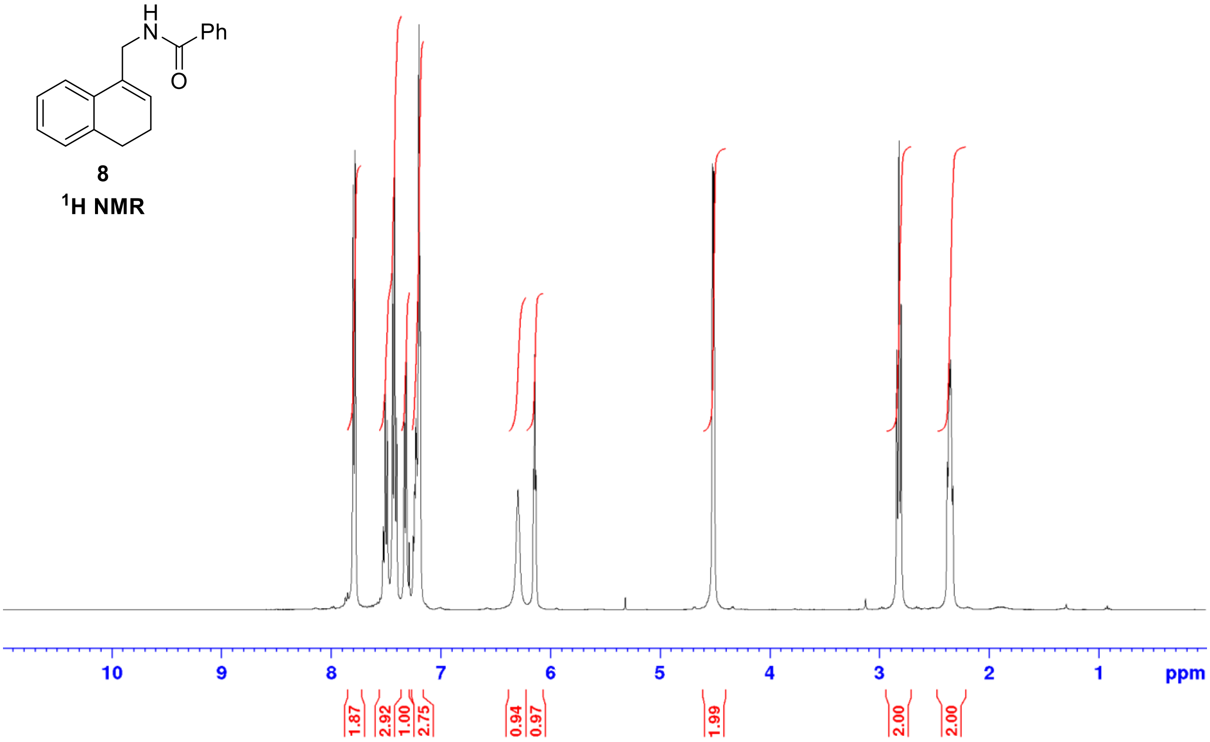

(1S*,2R*)-2'-phenyl-2-(trifluoromethyl)-3,4-dihydro-2H,4'H-spiro[naphthalene-1,5'-oxazole] (9)

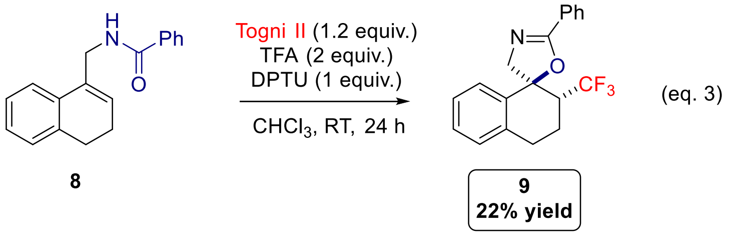

Into a vial containing *N*-((3,4-dihydronaphthalen-1-yl)methyl)benzamide (79.0 mg, 0.3 mmol), *N*,*N'*-diphenylthiourea (68.5 mg, 0.3 mmol), 1-trifluoromethyl-1,2-benziodoxol-3-(1H)-one (113.8 mg, 0.36 mmol), 4Å MS (30 mg) and a stirrer bar was added a solution of trifluoroacetic acid (45.9 µL, 0.6 mmol) in $CDCl_3$ (3.0 mL) under Ar atmosphere. The vial was sealed and allowed to stir at room temperature for 24 hours. The reaction mixture was then concentrated and purified by silica gel column chromatography (eluent: 1% NEt_3 and 10 % EtOAc in *n*-pentane) to provide the title compound (25.4 mg, 22 % yield) as a colourless oil. (^{1}H NMR, 400 MHz, $CDCl_3$): δ (ppm) = 7.93 (d, *J* = 8.4 Hz, 2H), 7.44 (t, *J* = 7.3 Hz, 1H), 7.37 (t, *J* = 7.4 Hz, 2H), 7.25−7.22 (m, 1H), 7.18−7.14 (m, 2H), 7.07−7.05 (m, 1H), 4.56 (d, *J* = 15.8 Hz, 1H), 3.97 (d, *J* = 15.8 Hz, 1H), 2.95−2.86 (m, 3H), 2.28−2.22 (m, 1H), 1.95−1.82 (m, 1H); (^{13}C NMR, 126 MHz, $CDCl_3$): δ (ppm) = 163.7, 140.5, 134.2, 131.5, 128.4, 128.4, 128.3, 128.1, 127.6, 127.4, 126.8 (q, *J* = 281.3 Hz), 125.3, 84.8, 65.5, 46.6 (q, *J* = 24.9 Hz), 28.0, 20.9 (q, *J* = 2.9 Hz); (^{19}F NMR, 377 MHz, $CDCl_3$): δ (ppm) = −66.6 (d, *J* = 8.2 Hz, 3F); IR: 2926, 1655, 1494, 1450, 1389, 1347, 1309, 1266, 1227, 1195, 1165, 1137, 1111, 1090, 1064, 1039, 1027, 980, 931, 895, 833 cm^{-1}; HRMS (ESI) for $C_{19}H_{17}ONF_3$ [M+H]$^+$ requires 332.1257 found 332.1256. Data consistent with literature values.[11]

[11] Noto, N.; Miyazawa, K.; Koike, T.; Akita, M. *Org. Lett.* **2015**, *17*, 3710.

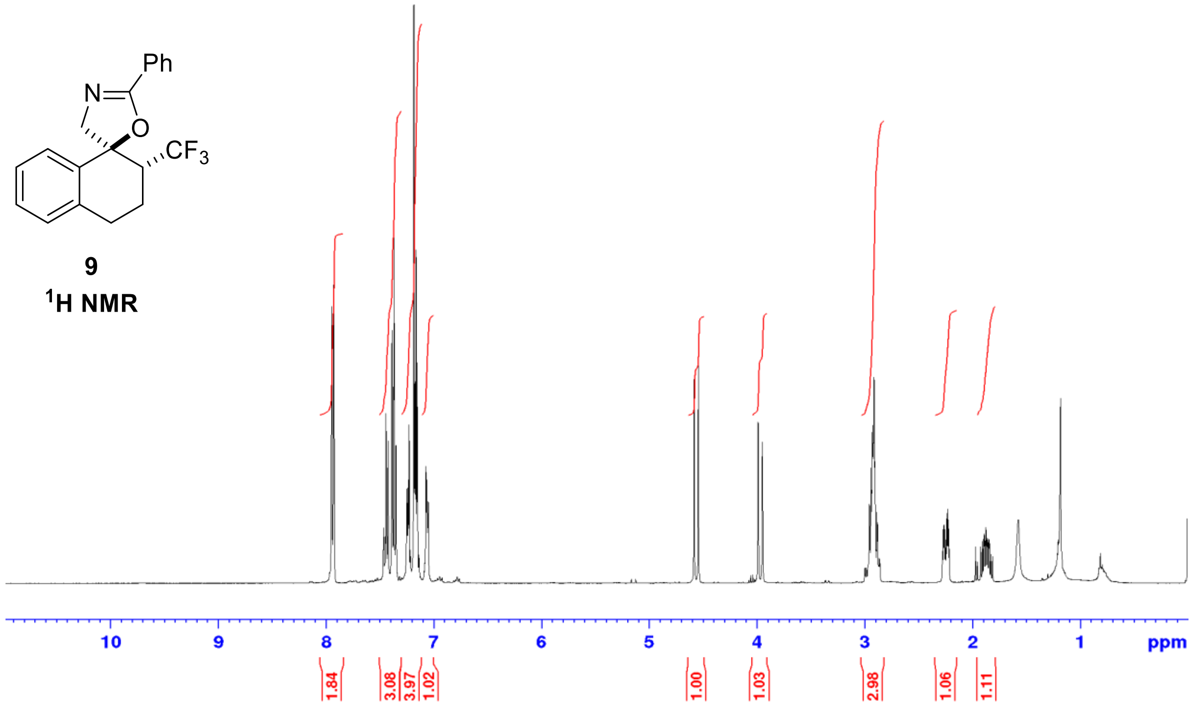

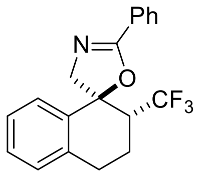

9

^{13}C NMR

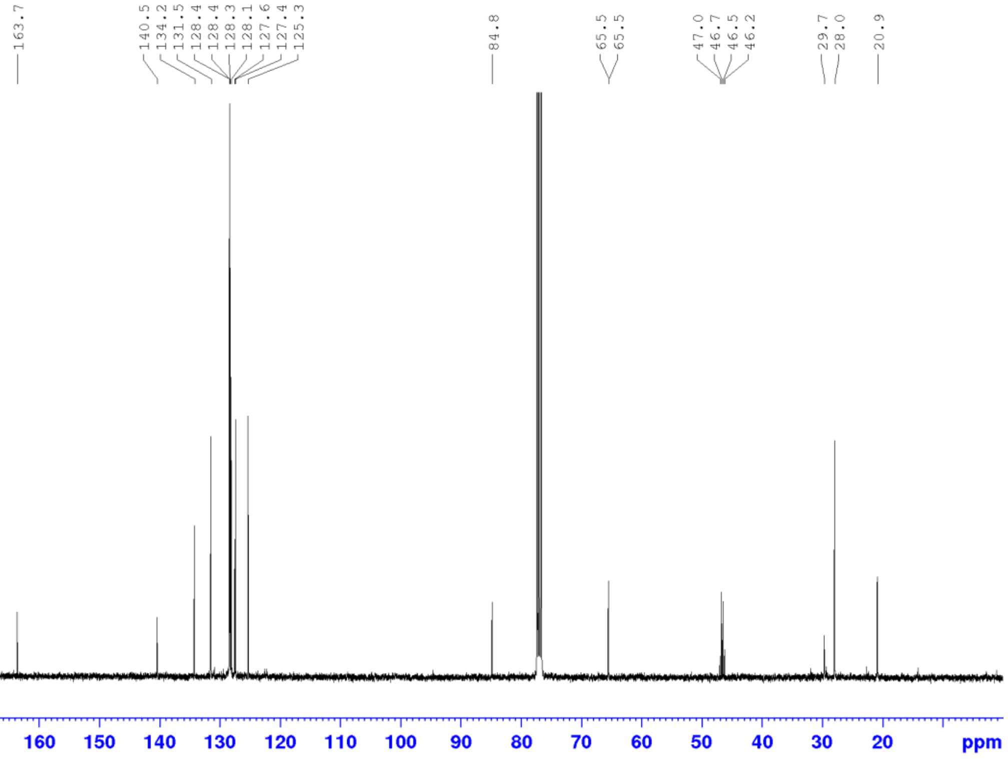

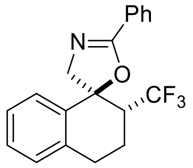

9

^{19}F NMR

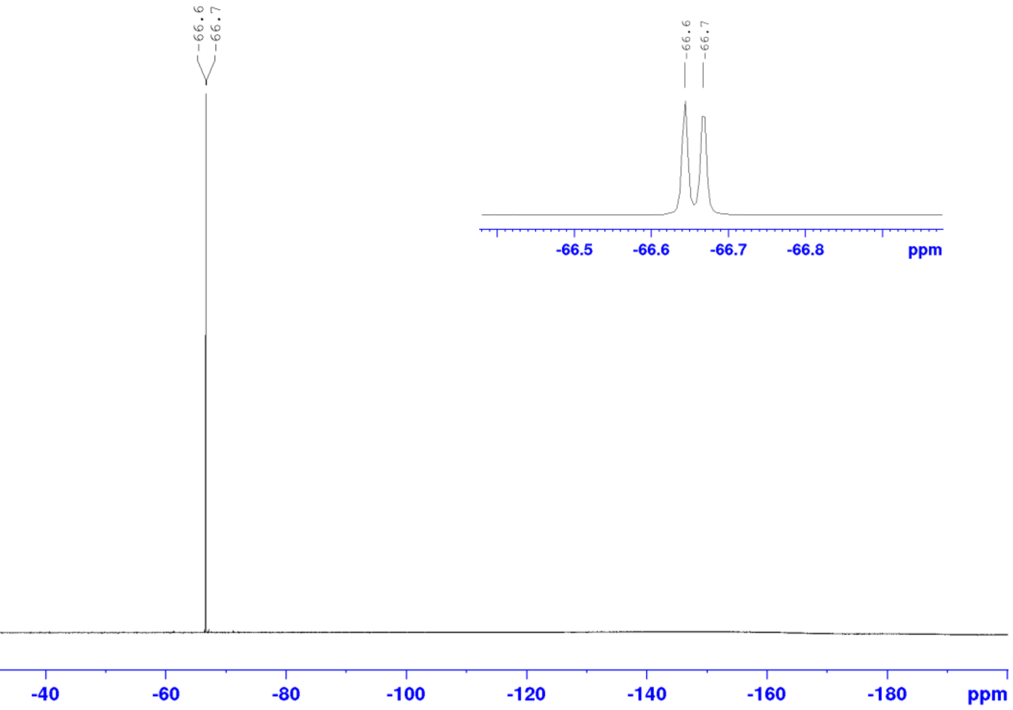

3.4. Thiotrifluoromethylation of Thioamide 12.

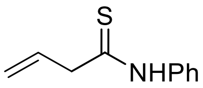

Benzaldehyde (1.1 ml, 10 mmol) was added to a solution of allylamine (11 mmol) in DMF (5 ml) at room temperature. Sulfur (0.352 g, 11 mmol) was then added and the mixture was heated at 80 ^{o}C for 6 hours with vigorous stirring. The reaction mixture solution was cooled down and poured into Et_2O (20 ml) and the organic layer was washed with sat. $NaHCO_3$ (50 ml) and HCl (2 ml, 35% v/v). The organic layer was dried with $MgSO_4$, and concentrated under reduced pressure. The residual oil was purified by column (20% Et_2O in hexane) to provide the title compound (1.43 g, 81% yield) as a pale yellow oil. Thioamide **12** has been previously reported in the literature.[12]

[12] Jianpeng, W.; Yiming, L.; Xuefeng, J. *Org. Lett.* **2016**, *18*, 340.

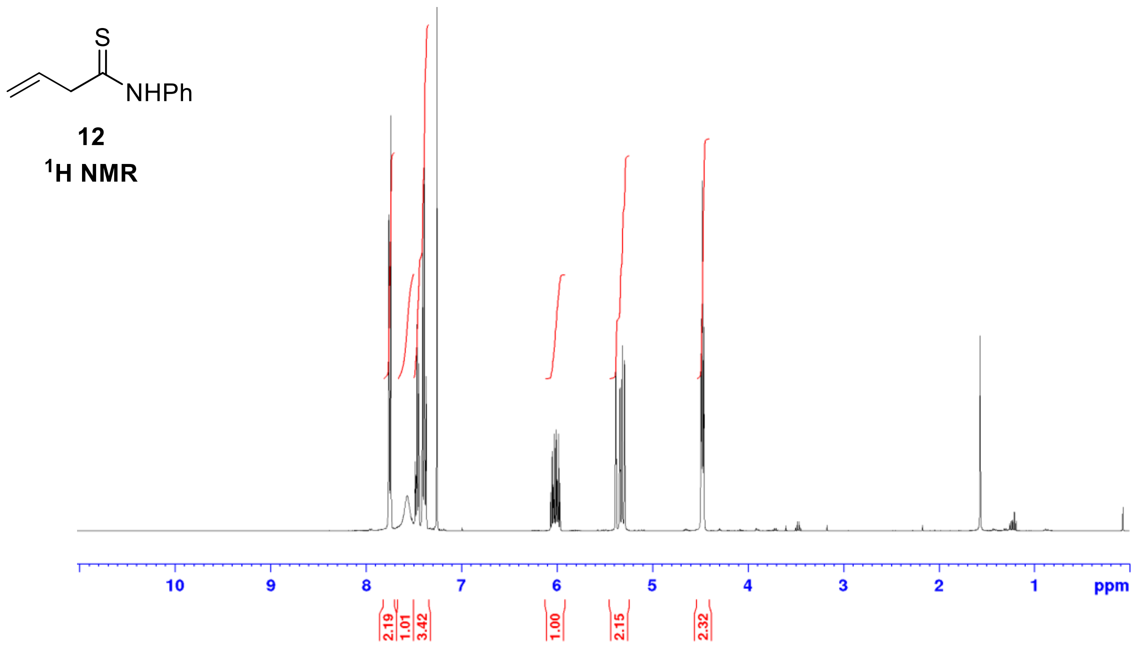

Thioamide **12** (11.5 mg, 0.1 mmol) was subjected to general procedure H for 24h. The crude reaction was analysed by ^{1}H NMR and ^{19}F NMR, using $C_6H_5CF_3$ (1 equiv.) as internal standard for ^{19}F NMR and mesitylene (1 equiv.) as internal standard for 1H NMR. ^{19}F NMR analysis showed no fluorinated adducts. ^{1}H NMR showed formation of amide **13**.

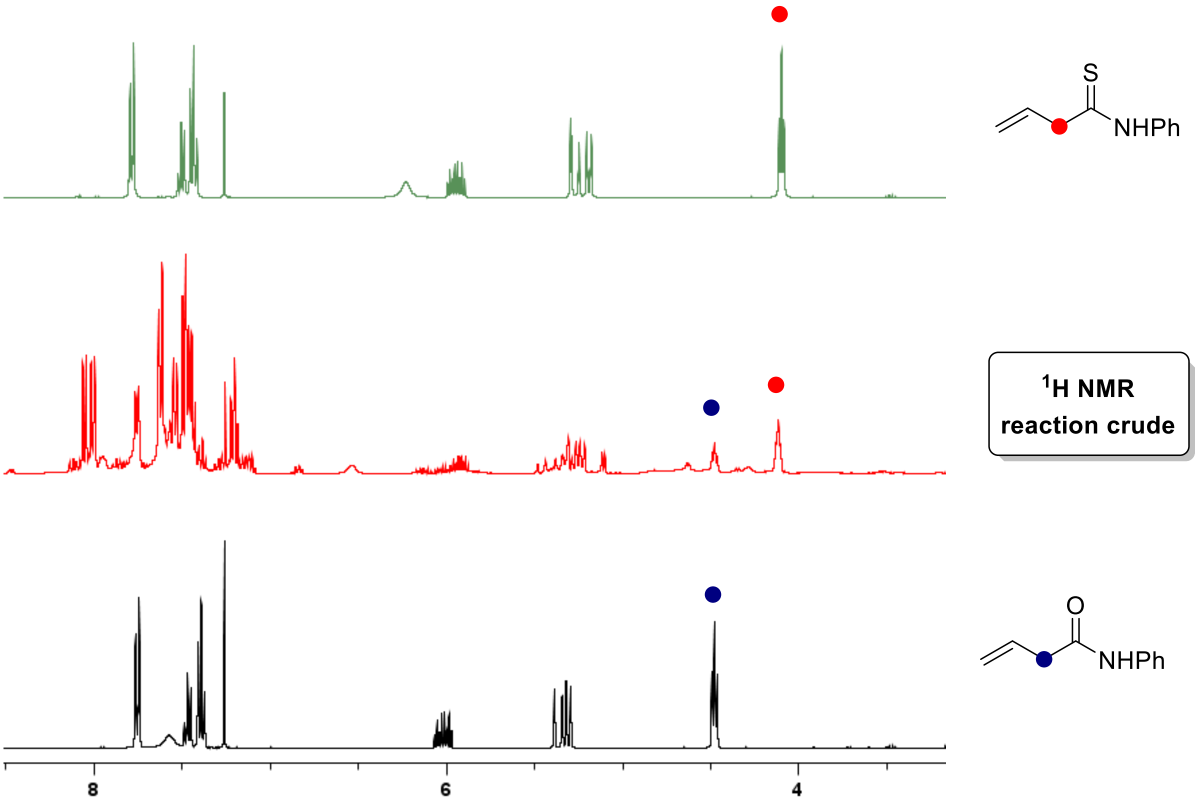

3.5. Thiotrifluoromethylation of Pent-4-ene-1-thiol 14.

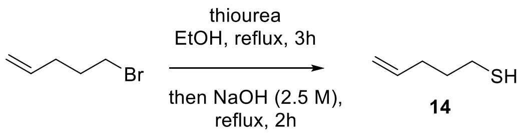

5-bromopent-1-ene (1.2 ml, 10 mmol), thiourea (800 mg, 10.5 mmol) were added to a flask containing ethanol and the mixture was refluxed for 3h. Then a solution of 2.5 M of NaOH in water (6 ml) was added and the reaction refluxed for an additional two hours. The acqueous layer was separated and acidified with aqueous HCl to pH= 1. The aqueous layer was extracted with pentane (2 x 20 ml) and the organic phase was washed with brine.

Removal of solvent under vacuum afforded pure pent-4-ene-1-thiol[13] **14** as colourless oil (520 mg, 51% yield). Thiol **14** has been previously reported in the literature.

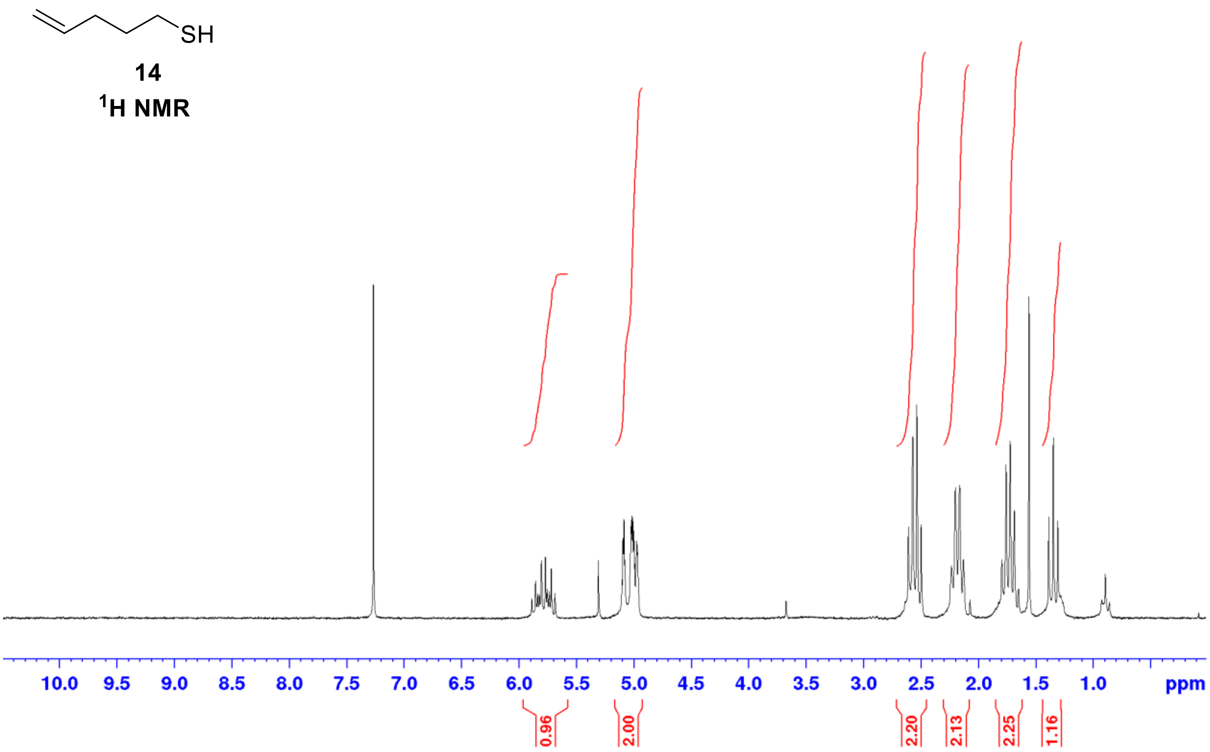

General procedure for the trifluoromethylation of **14**: a flame-dried flask was loaded with molecular sieves (10 mg), Togni II (38 mg, 0.12 mmol) and filled with argon. $CDCl_3$ (1 ml) was added and the flask was set up at the appropriate temperature (-78°C for entry 1 and 2, RT for entry 3 and 4). Thiol **14** (10.2 mg, 0.1 mmol) was added by syringe, followed by the addition of TFA (15 µl) where required (entry 2 and 4). The reaction was stirred at the required temperature for 16h and the crude mixture was then analysed by ^{1}H and ^{19}F NMR.

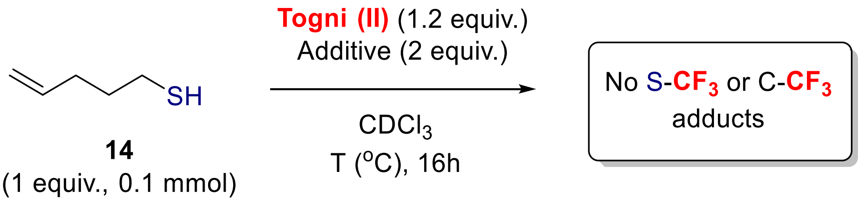

Entry	T (°C)	additive	^{19}F NMR yield (%)
A	-78 °C	TFA (2 equiv.)	0%
B	-78 °C	--	0%
C	RT	TFA (2 equiv.)	0%
D	RT	--	0%

^{19}F NMR of the crude reactions showed the absence of CF_3-containing adducts (either S-CF_3 or C-CF_3). The analysis of **^{1}H NMR** of the crude reactions showed: 1) complete consumption of SM 2) similar distribution of products for the reactions with/without TFA and at -78°C/RT.

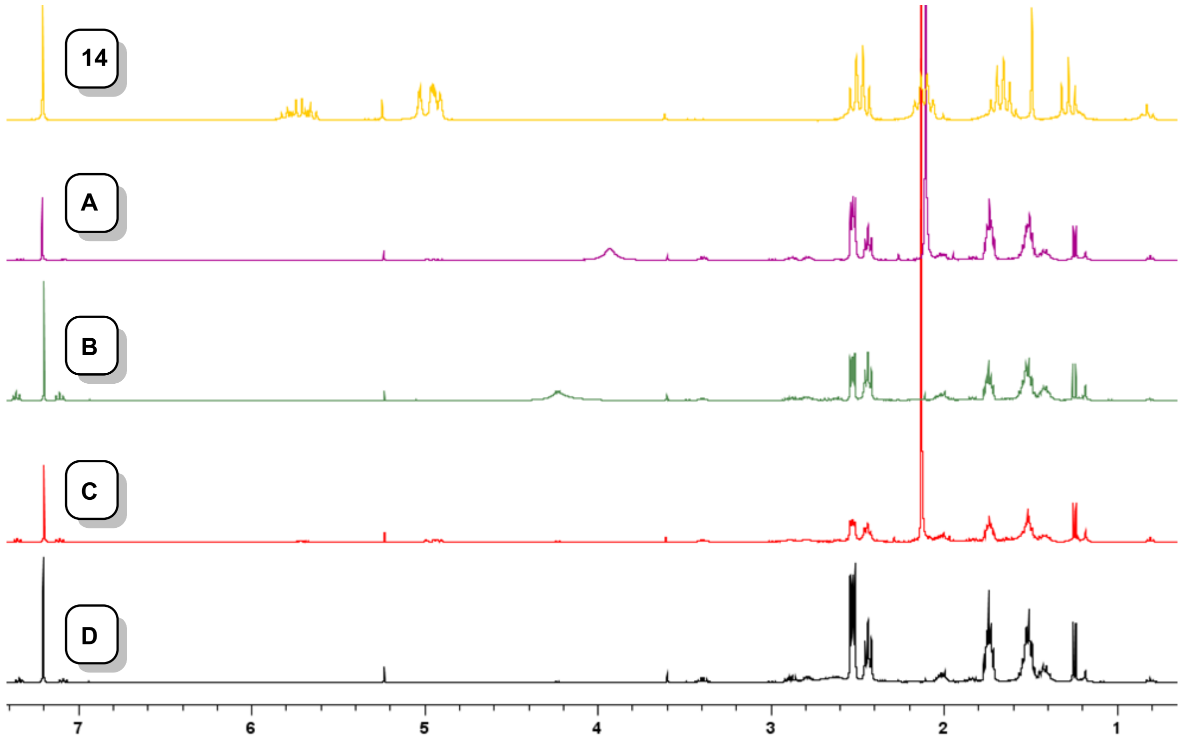

We can exclude the formation of S-CF_3 or C-CF_3 adducts by ^{19}F NMR, as well as the formation of S-S adducts by ^{1}H NMR. *The aliphatic nature of the 1H NMR peaks suggests an alternative pathway that involves* ***thiol-ene*** *type reactions.*

As known from the literature, thiol **14** can undergo provides thiol-ene[14] products **15** and **16** under acidic conditions[15], or undergo thiol-ene polymerization in presence of light[16].

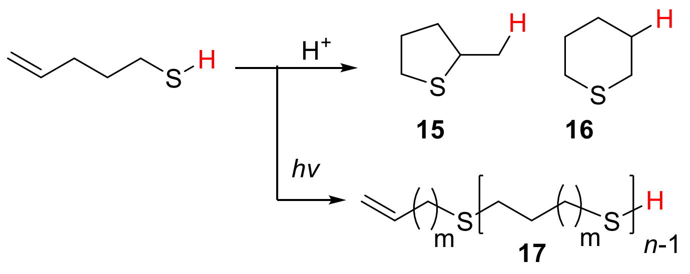

The presence of 9% by crude 1H NMR of compound **13** supports a thiol-ene type mechanistic pathway.

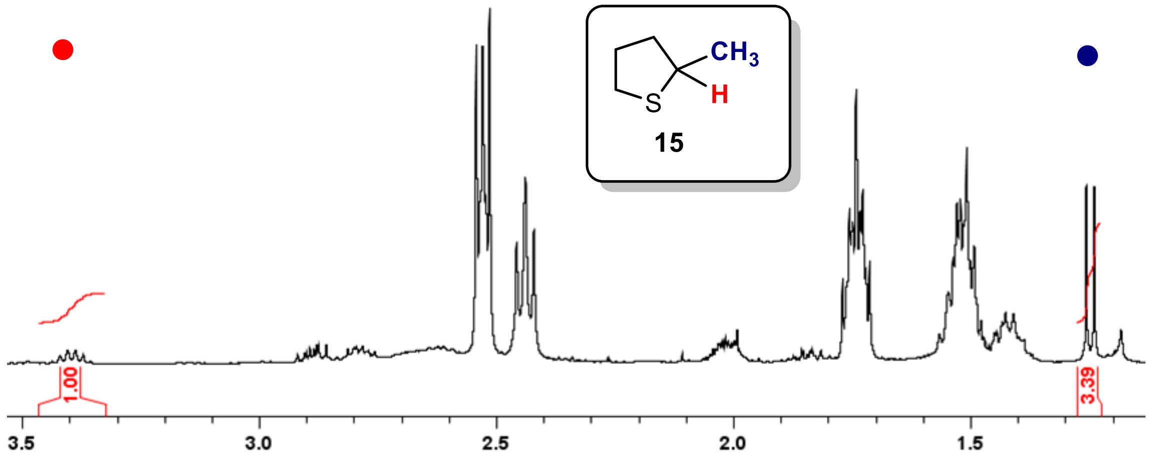

[13] Carta P.; Puljic, N.; Carline, R.; Dhimane, A.-L.; Ollivier, C.; Fensterbank, L.; Lacote, E.; Malacria, M. Tetrahedron **2008**, *64*, 11865
[14] Hoyle, C. E.; Bowman, C. N. Angew. Chem. Int. Ed. **2010**, *49*, 1540
[15] Dronov, V. I., Krigonovov, V. P. *Organ. Soedin. Sery, Riga*, **1980**, *2*, 58
[16] Deubel F.; Bretzler, V.; Holzner, R.; Helbich, T.; Nuyken, O.; Rieger, B.; Jordan, R. *Macromol. Rapid Commun.* **2013**, *34*, 1020-1025.

3.6. Thiotrifluoromethylation of 1aa with Different Acids.

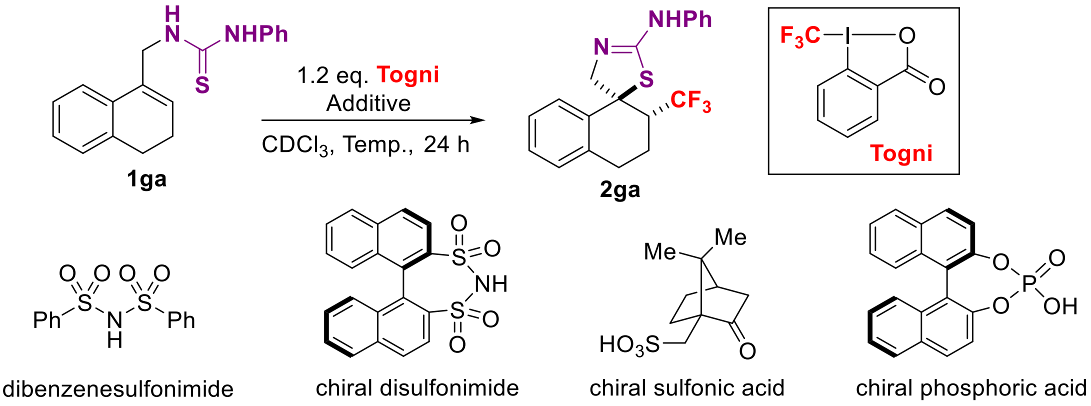

Entry	Temp.	Additive	^{19}F NMR Yield of 2ga
**1**	rt	-	11 %
**2**	rt	20 mol% TFA	49 %
**3**	rt	50 mol% TFA	61 %
**4**	rt	1 eq. TFA	74 %
**5**	rt	2 eq. TFA	79 %
**6**	rt	2 eq. dibenzenesulfonimide	76 %
**7**	rt	2 eq. chiral disulfonimide	48 %
**8**	rt	2 eq. chiral sulfonic acid	52 %
**9**	rt	2 eq. chiral phosphoric acid	14 %

3.7. Additional Studies on the Selectivity.

(*E*)-1-(Hex-2-en-1-yl)-3-phenylthiourea (15)

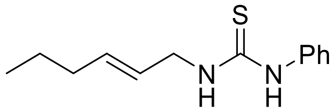

15

(*E*)-2-(hex-2-en-1-yl)isoindoline-1,3-dione was prepared following general procedure F using THF (30 mL), PPh_3 (1.59 g, 6.1 mmol), phthalimide (890 mg, 6.1 mmol) and diethyl azodicarboxylate (0.95 mL, 6.1 mmol). The crude product was used on the next reaction without further purification. To the crude mixture was added hydrazine monohydrate (0.8 mL, 16.5 mmol) and stirred at room temperature overnight. The crude amine was subjected to the conditions described by general procedure A using phenyl isothiocyanate (0.78 mL, 6.5 mmol) and DCM (50 mL). The crude product was purified by column chromatography (20% EtOAc in hexane) to give the title compound **15** (509 mg, 43 % yield) as a pale yellow solid.

(^{1}H NMR, 400 MHz, $CDCl_3$): δ (ppm) = 8.17 (br s, 1H), 7.43–7.30 (m, 2H), 7.26–7.19 (m, 1H), 7.18–7.13 (m, 2H), 5.97 (br s, 1H), 5.62–5.48 (m, 1H), 5.45–5.37 (m, 1H), 4.13 (t, *J* = 5.9 Hz, 2H), 1.97–1.83 (m, 2H), 1.33–1.25 (m, 2H), 0.79 (t, *J* = 7.4 Hz, 3H); (^{13}C NMR, 101 MHz, $CDCl_3$): δ (ppm) = 180.25, 136.16, 134.73, 130.17, 127.20, 125.21, 124.65, 47.53, 34.27, 22.16, 13.61. IR: 3211, 2957, 2927, 2870, 1596, 1528, 1496, 1452, 1346, 1313, 1258, 1235, 1185, 1073, 1028, 969, 934 cm^{-1}; HRMS (ESI) for $C_{13}H_{18}N_2S$ $[M+H]^+$ requires 235.1263 found 235.1263. Mp. 78−79 °C.

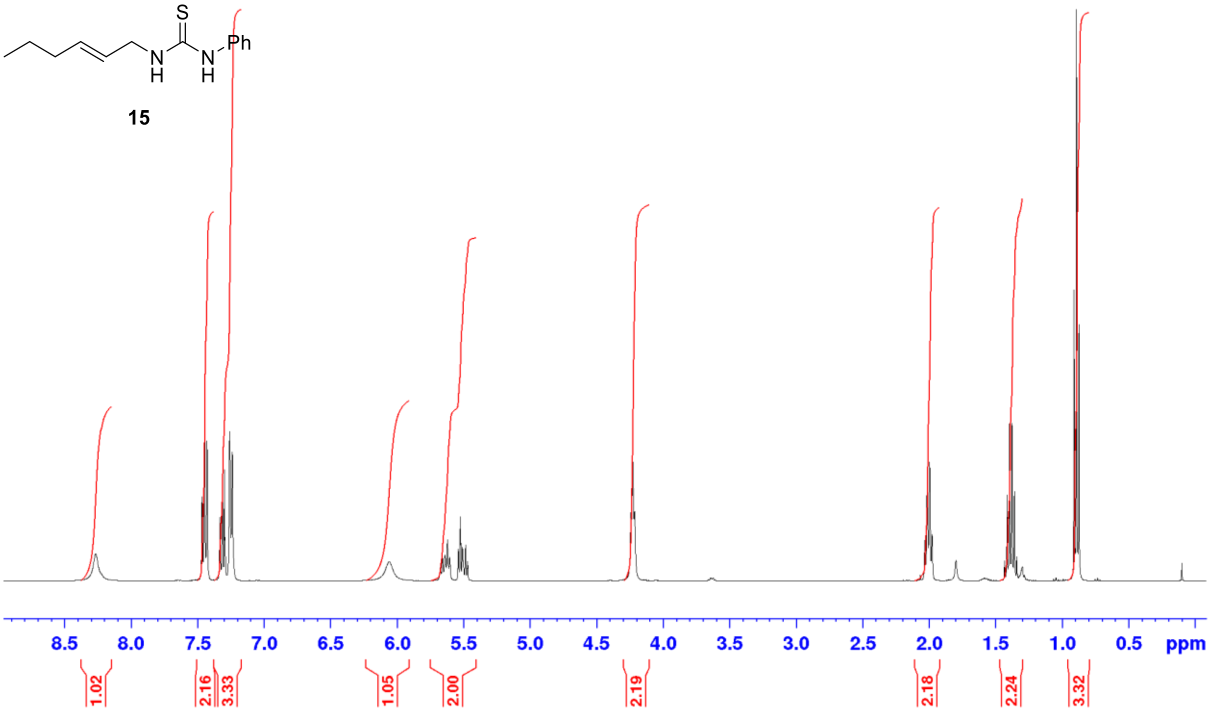

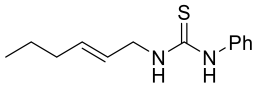

15

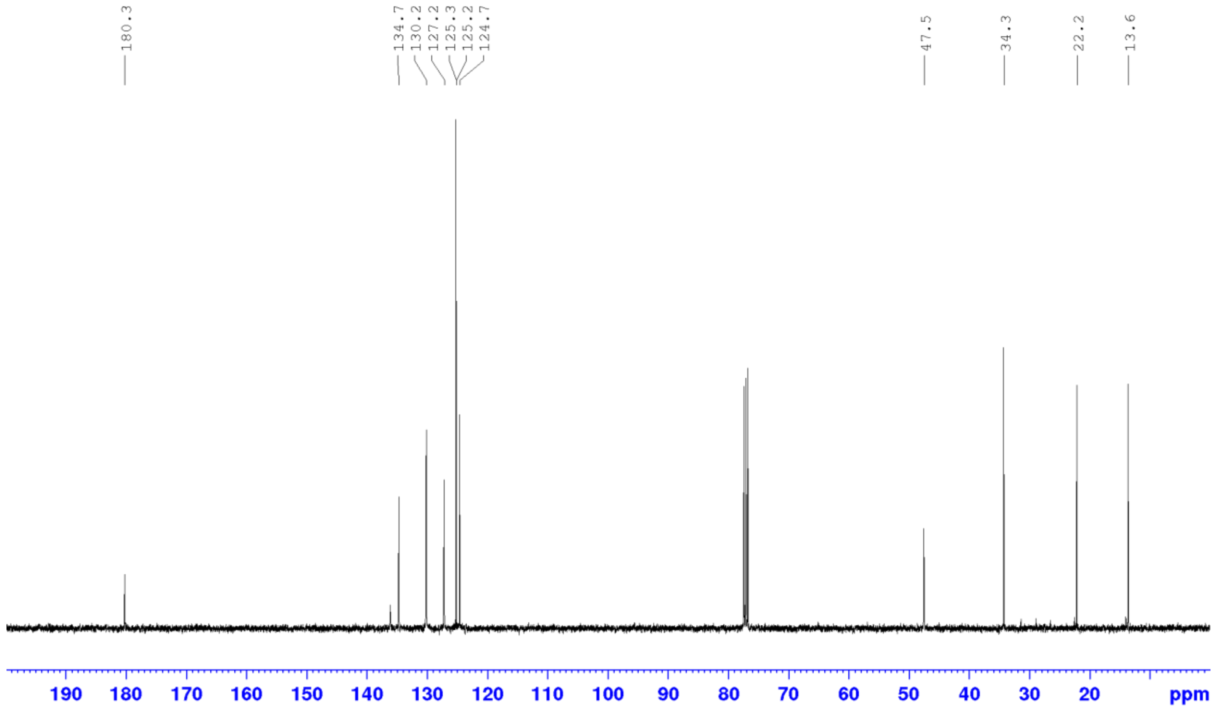

N-Phenyl-5-(1,1,1-trifluoropentan-2-yl)-4,5-dihydrothiazol-2-amine (16a) and N-phenyl-6-propyl-5-(trifluoromethyl)-5,6-dihydro-4H-1,3-thiazin-2-amine (16b)

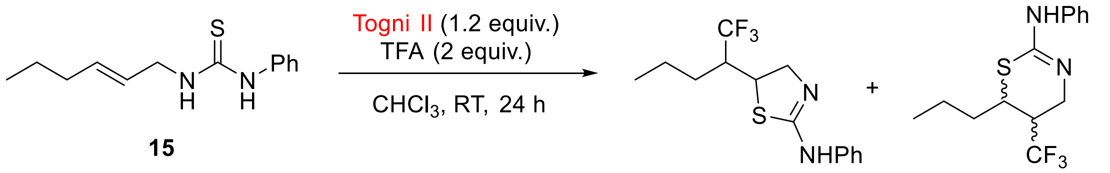

Into a vial containing (*E*)-1-(hex-2-en-1-yl)-3-phenylthiourea **15** (141 mg, 0.6 mmol), 1-trifluoromethyl-1,2-benziodoxol-3-(1H)-one (228 mg, 0.72 mmol) and a stirrer bar was added a solution of trifluoroacetic acid (91.8 µL, 1.2 mmol) in $CHCl_3$ (6.0 mL) under Ar atmosphere. The vial was sealed and allowed to stir at room temperature for 24 hours. The reaction mixture was then concentrated and purified by silica gel column chromatography (eluent: 25 % EtOAc in *n*-pentane) to provide *N*-phenyl-5-(1,1,1-trifluoropentan-2-yl)-4,5-dihydrothiazol-2-amine **16a** (40.6 mg, 22 % yield) as a pale yellow oil and *N*-phenyl-6-propyl-5-(trifluoromethyl)-5,6-dihydro-4H-1,3-thiazin-2-amine **16b** (36.2 mg, pale yellow oil) as a mixture of diastereomer (d.r. = 1.5:1).

N-Phenyl-5-(1,1,1-trifluoropentan-2-yl)-4,5-dihydrothiazol-2-amine (16a)

(^{1}H NMR, 400 MHz, $CDCl_3$): δ (ppm) = 7.28–7.13 (m, 4H), 7.02–6.89 (m, 1H), 4.77 (br s, 1H), 3.71 (dd, J_1 = 4.7, J_2 = 13.5 Hz, 1H), 3.47 (dd, J_1 = 8.4, J_2 = 13.4, Hz, 1H), 3.34 (ddd, J_1 = 4.7, J_2 = 6.2, J_3 = 9.0 Hz, 1H), 2.45–2.14 (m, 1H), 1.79–1.66 (m, 1H), 1.66–1.51 (m, 1H), 1.51–1.37 (m, 1H), 1.37–1.24 (m, 1H), 0.85 (t, J = 7.3 Hz, 3H); (^{13}C NMR, 158 MHz, $CDCl_3$): δ (ppm) = 153.5, 142.3, 129.1, 129.0, 126.5 (q, J = 281.3 Hz), 123.1, 120.4, 44.5 (q, J = 25.3 Hz), 41.4, 39.8, 19.7, 13.5; (^{19}F NMR, 377 MHz, $CDCl_3$): δ (ppm) = −68.5 (d, J = 8.0 Hz, 3F); IR: 2963, 2933, 2875, 2363, 1626, 1590, 1519, 1497, 1459, 1439, 1383, 1311, 1251, 1166, 1128, 1090, 971, 899, 847 cm^{-1} ; HRMS (ESI) for $C_{14}H_{17}N_2SF_3$ $[M+H]^+$ requires 303.1137 found 303.1138.

N-Phenyl-6-propyl-5-(trifluoromethyl)-5,6-dihydro-4H-1,3-thiazin-2-amine (16b)

Major: (^{1}H NMR, 400 MHz, $CDCl_3$): δ (ppm) = 7.26–7.19 (m, 2H), 7.10–7.02 (m, 2H), 6.99 (t, J = 7.5 Hz, 1H), 5.42 (s, 1H), 4.12–4.01 (m, 1H), 3.80 (dd, J_1 = 7.2, J_2 = 11.6 Hz, 1H), 3.62 (dd, J_1 = 8.3, J_2 = 11.6 Hz, 1H), 2.41–2.23 (m, 1H), 1.67–1.25 (m, 4H), 0.83 (t, J = 7.2 Hz, 3H); (^{13}C NMR, 158 MHz, $CDCl_3$): δ (ppm) = 158.7, 145.4, 128.0, 126.1 (q, J = 281.3 Hz), 122.4, 120.0, 54.8, 47.2, 45.1 (q, J = 25.0 Hz), 28.7, 19.4, 13.0 (^{19}F NMR, 377 MHz, $CDCl_3$): δ (ppm) = −67.7 (d, J = 9.1 Hz, 3F);

Minor: (^{1}H NMR, 400 MHz, $CDCl_3$): δ (ppm) = 7.26–7.19 (m, 2H), 7.10–7.02 (m, 2H), 6.99 (t, J = 7.5 Hz, 1H), 5.42 (s, 1H), 4.12–4.01 (m, 1H), 3.89 (dd, J_1 = 7.2, J_2 = 11.6 Hz, 1H), 3.56 (dd, J_1 = 7.8, J_2 = 11.8 Hz, 1H), 2.41–2.23 (m, 1H), 1.67–1.25 (m, 4H), 0.86 (t, J = 7.2 Hz, 3H); (^{13}C NMR, 158 MHz, $CDCl_3$): δ (ppm) = 158.9, 144.9, 128.0, 126.3 (q, J = 281.3 Hz), 122.4, 119.9, 52.9, 45.9, 45.0 (q, J = 25.0 Hz), 26.1, 20.0, 13.0; (^{19}F NMR, 377 MHz, $CDCl_3$): δ (ppm) = −67.8 (d, J = 9.1 Hz, 3F);

IR: 2968, 2876, 2360, 1642, 1591, 1555, 1495, 1468, 1445, 1381, 1321, 1251, 1168, 1132, 1068, 1038, 968, 900, 855 cm^{-1} ; HRMS (ESI) for $C_{14}H_{17}N_2SF_3$ $[M+H]^+$ requires 303.1137 found 303.1139.

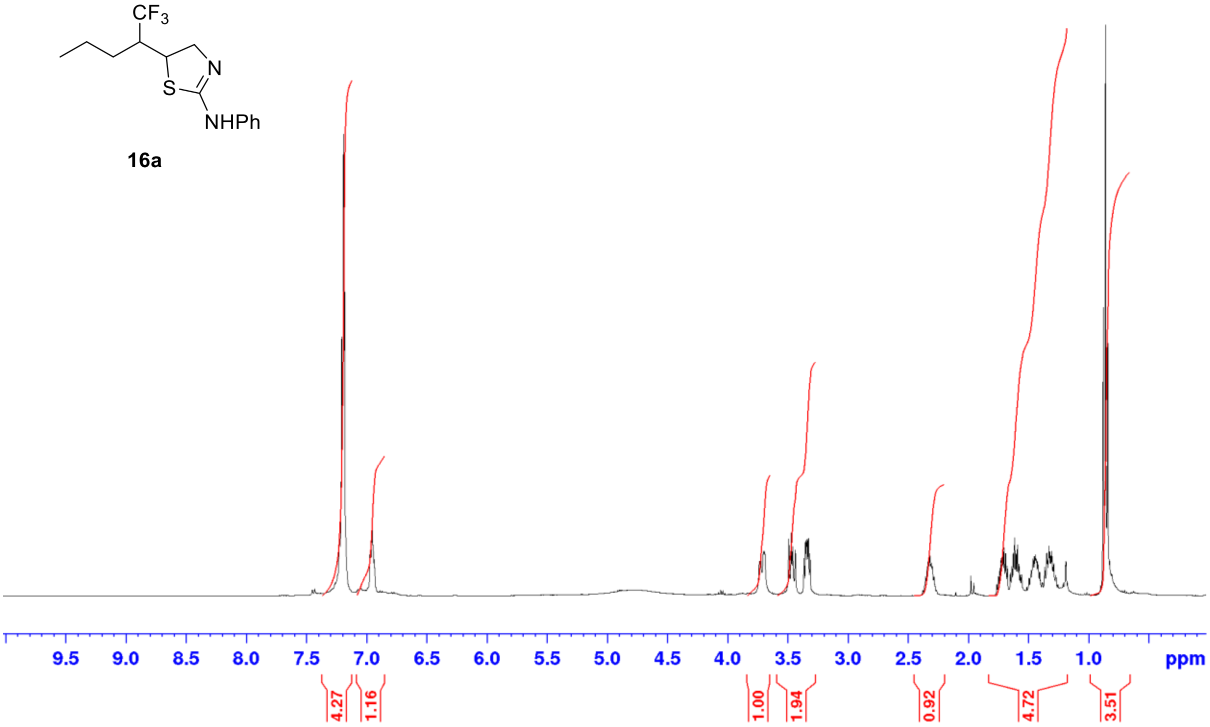
CF3
N
S
NHPh
16a
9.5 9.0 8.5 8.0 7.5 7.0 6.5 6.0 5.5 5.0 4.5 4.0 3.5 3.0 2.5 2.0 1.5 1.0 ppm
4.27 1.16 1.00 1.94 0.92 4.72 3.51

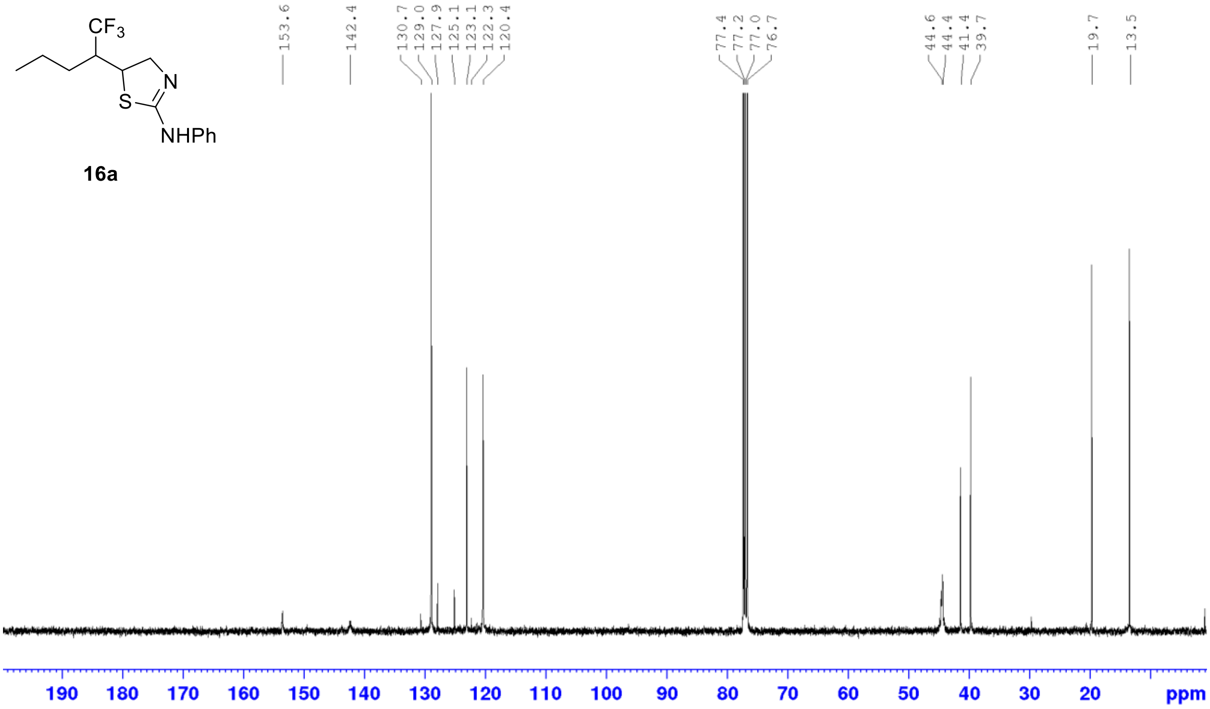
CF3
N
S
NHPh
16a
153.6
142.4
130.7 129.0 127.9 125.1 123.1 122.3 120.4
77.4 77.2 77.0 76.7
44.6 44.4 41.4 39.7
19.7
13.5
190 180 170 160 150 140 130 120 110 100 90 80 70 60 50 40 30 20 ppm

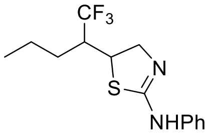

16a

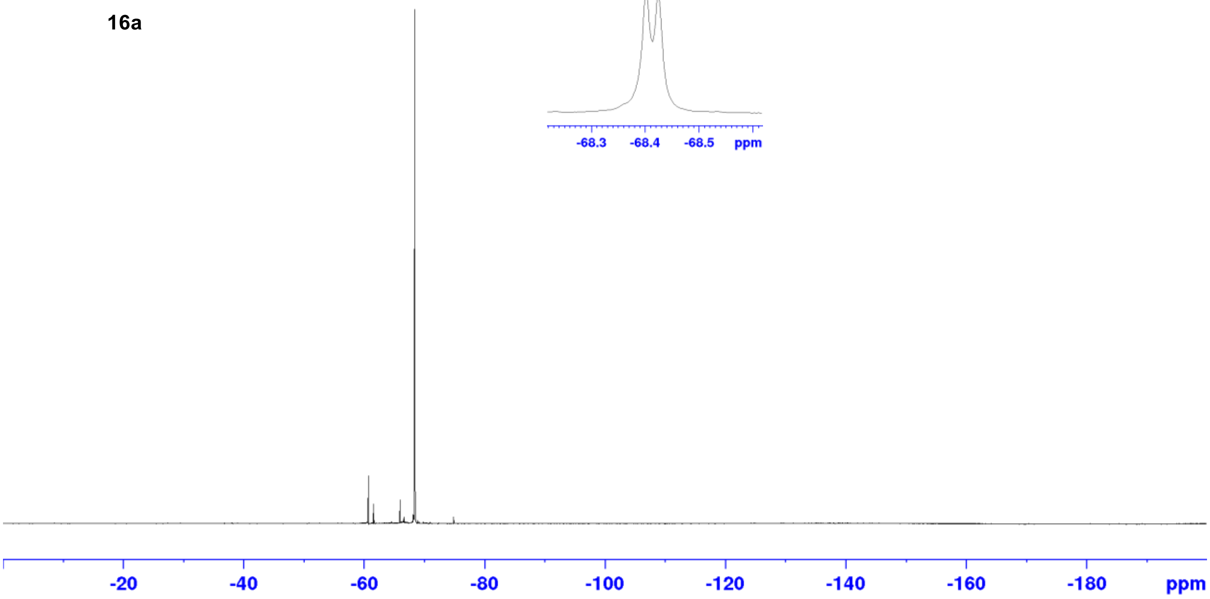

-68.4
-68.4
-68.3
-68.4
-68.5
ppm
-20
-40
-60
-80
-100
-120
-140
-160
-180
ppm

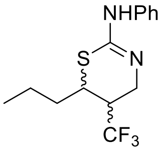

16b

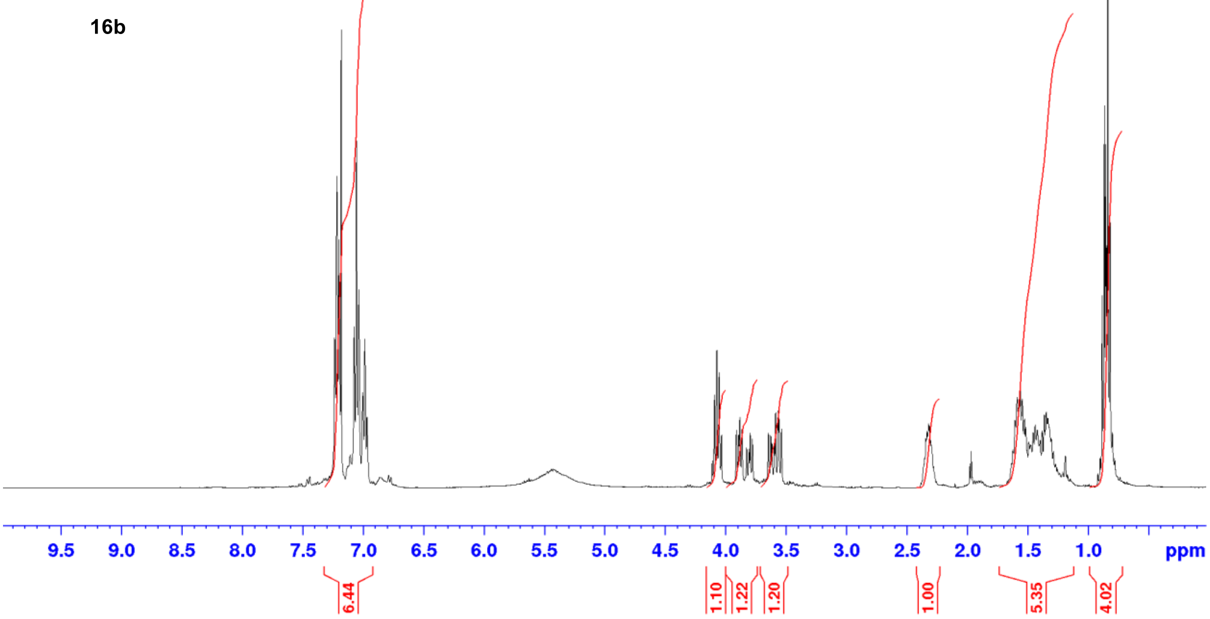

9.5
9.0
8.5
8.0
7.5
7.0
6.5
6.0
5.5
5.0
4.5
4.0
3.5
3.0
2.5
2.0
1.5
1.0
ppm
6.44
1.10
1.22
1.20
1.00
5.35
4.02

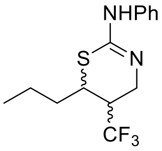
NHPh
S
N
CF3

16b

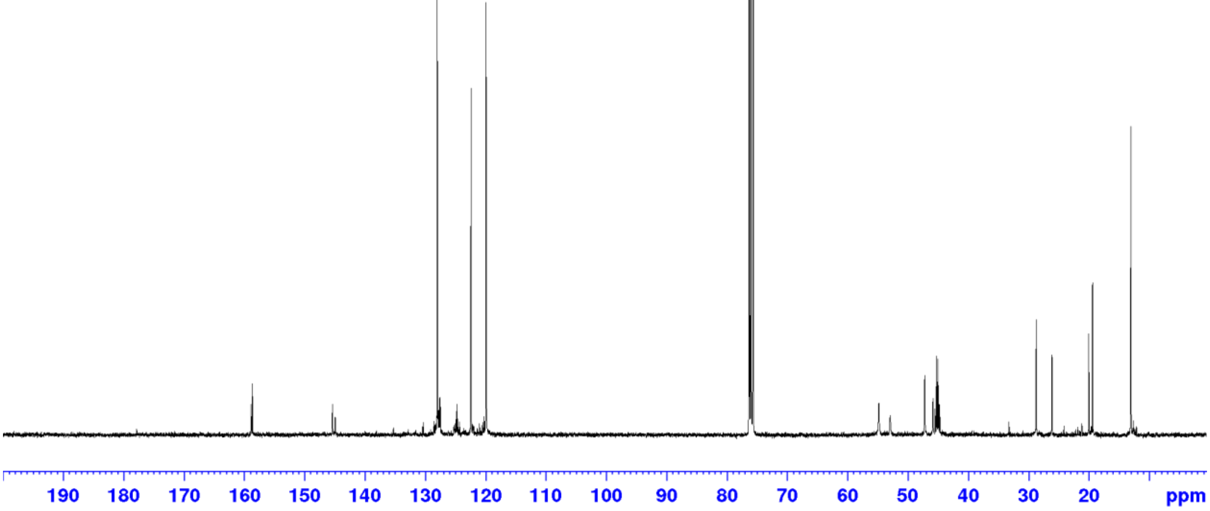
158.9
158.7
145.4
144.9
130.5
130.3
128.0
127.7
127.5
124.9
124.7
122.5
120.0
119.9
54.8
52.9
47.2
45.9
45.5
45.4
45.3
45.2
45.0
44.9
44.8
28.7
26.1
26.1
20.0
19.4
13.0
190
180
170
160
150
140
130
120
110
100
90
80
70
60
50
40
30
20
ppm

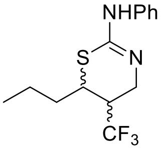
NHPh
S
N
CF3

16b

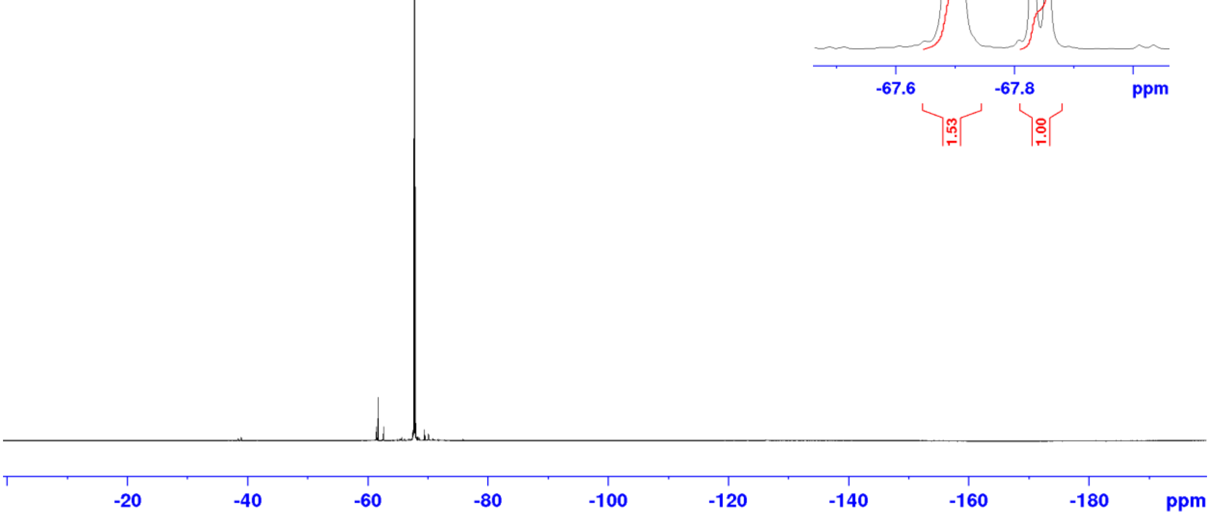
-67.7
-67.7
-67.8
-67.9
-67.7
-67.7
-67.8
-67.9
-67.6
-67.8
ppm
1.53
1.00
-20
-40
-60
-80
-100
-120
-140
-160
-180
ppm

4. Cyclic Voltammetry Analysis

Electrochemical measurements were performed using an EG & G-Princeton Applied Research 263A all-in-one potentiostat, using a standard three-electrode setup with a glassy carbon electrode (diameter = 3 mm) or a platinum electrode (diameter = 1.6 mm), a platinum wire auxiliary electrode and a non-aqueous Ag/Ag^+ (0.1 M NBu_4ClO_4 + 0.01 M $AgNO_3$) system in acetonitrile as the reference electrode. All solutions of the compounds under the study were 0.1 M in the supporting electrolyte *n*-Et_4NBF_4 with the voltage scan rate of 0.2 $V.s^{-1}$. Solutions were thoroughly bubbled with dry argon for 15 minutes to remove any oxygen before any experiment and kept under positive pressure of argon. Under these experimental conditions the ferrocene/ferricinium couple, used as internal reference for potential measurements, was located at $E_{1/2}$ = + 0.046 V in CH_3CN. For conversion factors (SCE *vs* Ag/Ag+), see: V. V. Palvlishchuk and A. W. Adison, *Inorg. Chim. Acta*, **2000**, 298, 97.

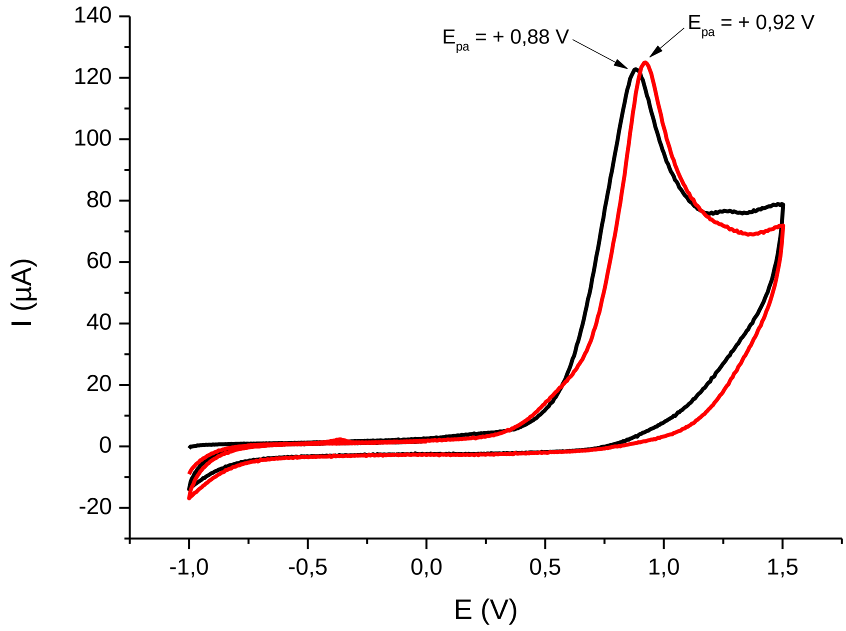

Figure S1. Cyclic voltammetry of thiourea **1aa** C = 3.5 mM in CH_3CN + *n*-Et_4NBF_4 0.1 M + in the absence (black curve) and presence (red curve) of TFA (2 eq); glassy carbon electrode at 0.2 V/s. In the absence of TFA, E_{pox} = + 0.88 V *vs* Ag/Ag^+ = + 1.19 V *vs* SCE and in the presence of TFA, E_{pox} = + 0.92 V *vs* Ag/Ag^+ = **+ 1.23 V *vs* SCE**.

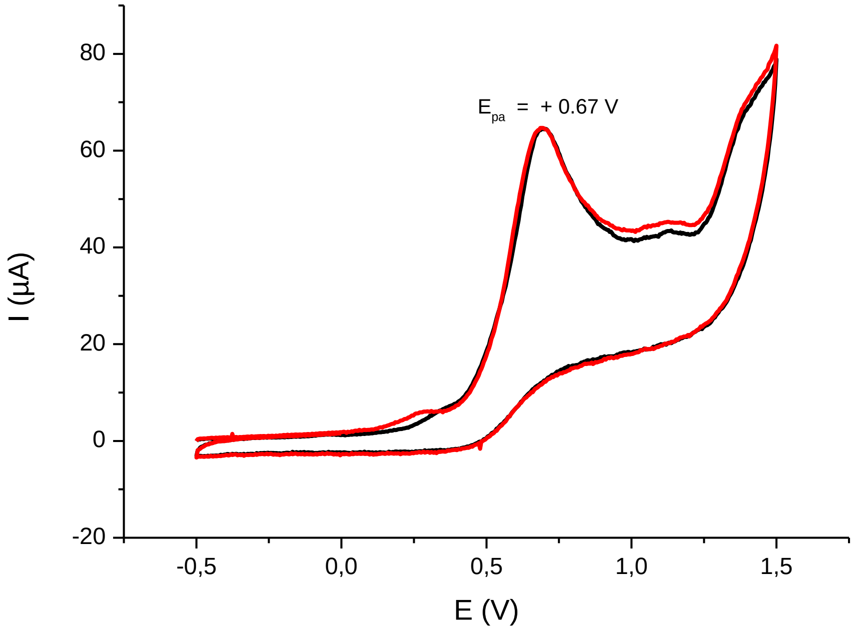

Figure S2. Cyclic voltammetry of thiourea **1ga** *C* = 1.29 mM in CH_3CN + *n*-Et_4NBF_4 0.1 M in the absence (black curve) and presence (red curve) of TFA (2 eq); glassy carbon electrode at 0.2 V/s; E_{pox} = + 0.67 V *vs* Ag/Ag^+ = **+ 0.98 V *vs* SCE**.

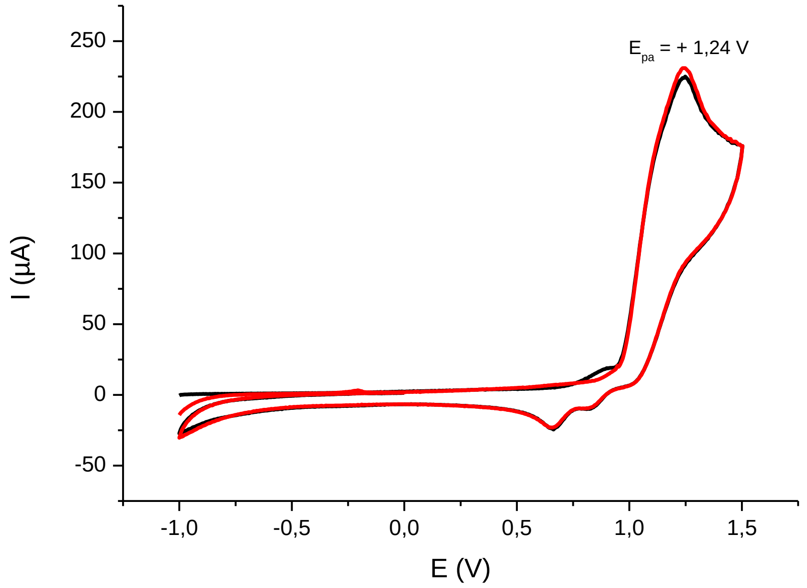

Figure S3. Cyclic voltammetry of urea **6** *C* = 3.06 mM in CH_3CN + *n*-Et_4NBF_4 0.1 M in the absence (black curve) and presence (red curve) of TFA (2 eq); glassy carbon electrode at 0.2 V/s; E_{pox} = + 1.24 V *vs* Ag/Ag^+ = **+ 1.56 V *vs* SCE**.

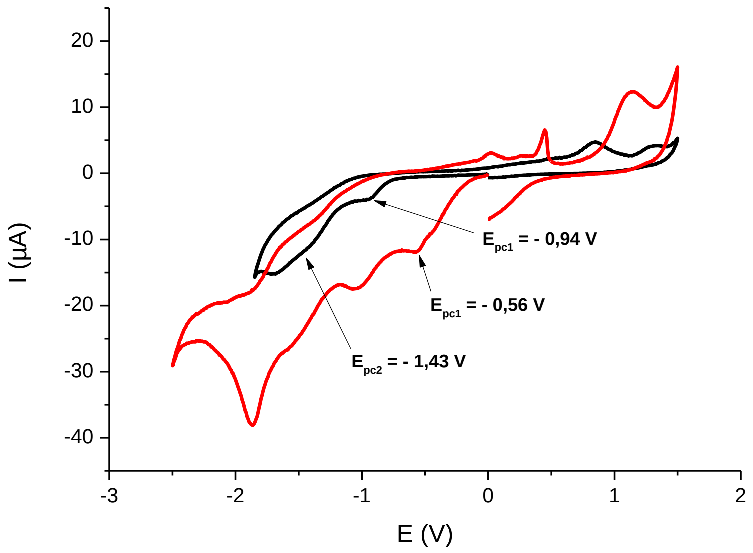

Figure S4. Cyclic voltammetry of Togni reagent **II** *C* = 1.77 mM in CH_3CN + *n*-Et_4NBF_4 0.1 M in the absence (black curve) and presence of TFA (2 eq) (red curve) and presence of TFA (2 eq) + thiourea (1 eq) **1ga** (xxx curve); platinum electrode at 0.2 V/s.

For Togni reagent alone: E_{pc1} = - 0.94 V *vs* Ag/Ag^+ = **- 0.63 V *vs* SCE**; E_{pc2} = - 1.43 V *vs* Ag/Ag^+ = **- 1.12 V *vs* SCE**

For Togni reagent in the presence of TFA: E_{pc1} = - 0.56 V *vs* Ag/Ag^+ = **- 0.25 V *vs* SCE**

5. NMR Spectra of Novel Compounds

1-Allyl-3-phenylthiourea (1aa)

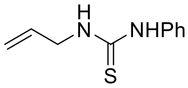

1aa

^{1}H NMR

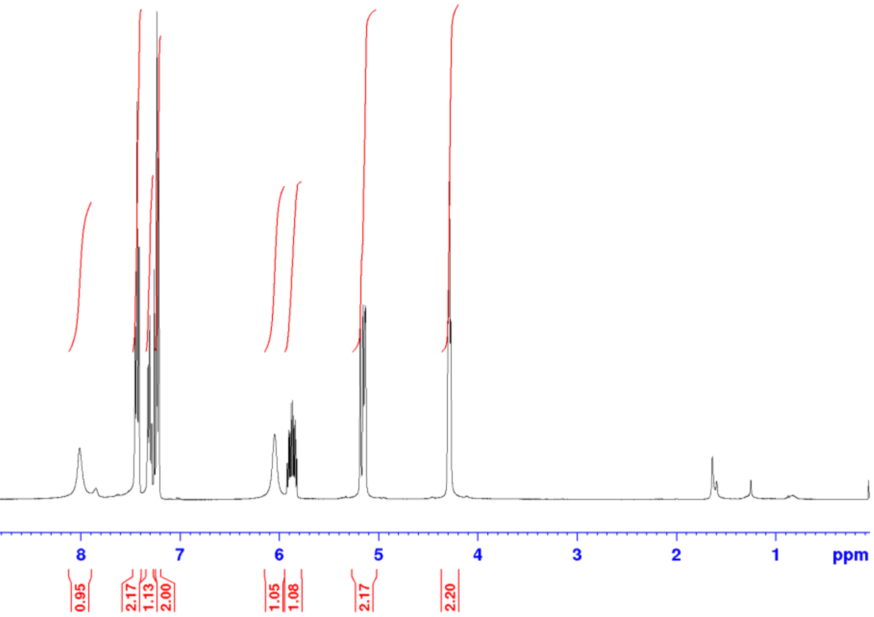

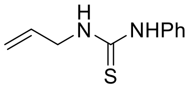

1aa

^{13}C NMR

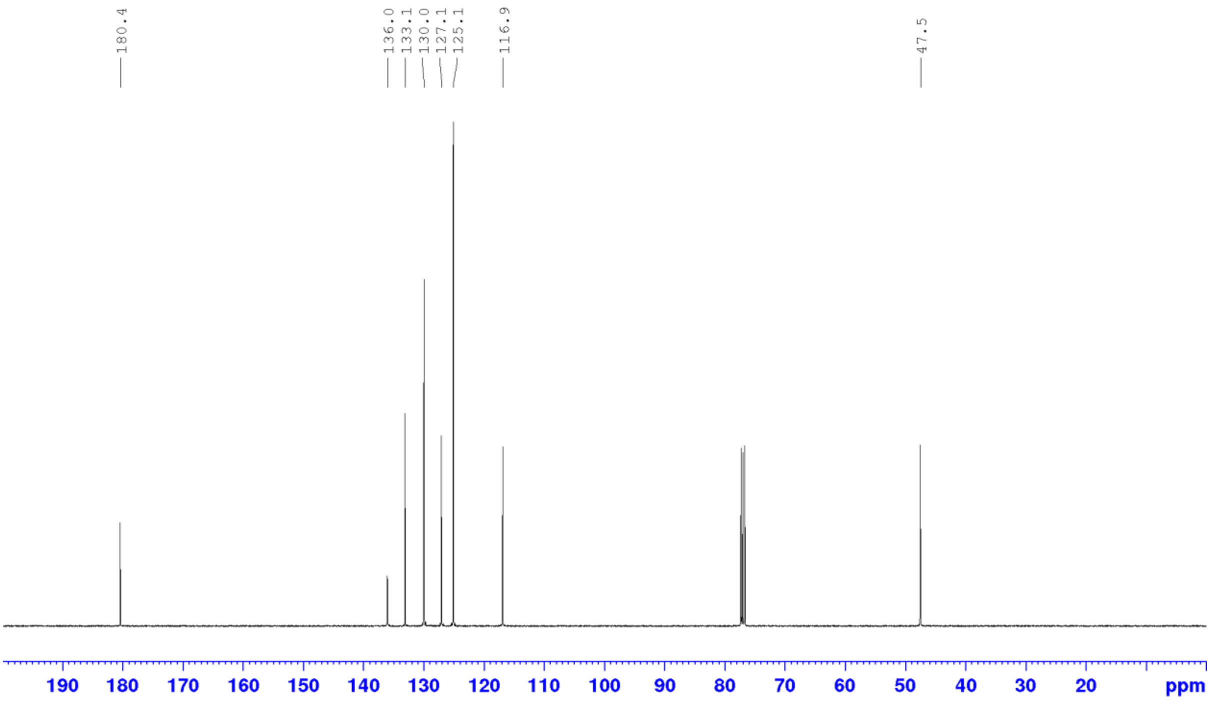

1-(2-Methylallyl)-3-phenylthiourea (1ba)

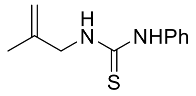

1ba
^{1}H NMR

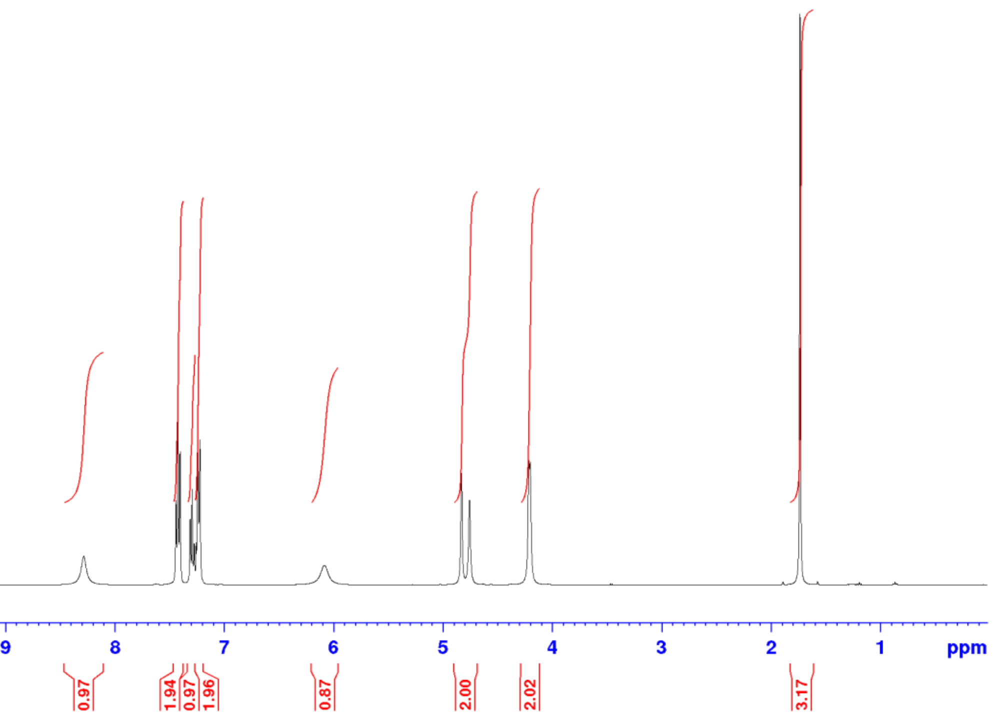

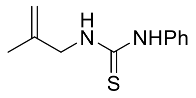

1ba
^{13}C NMR

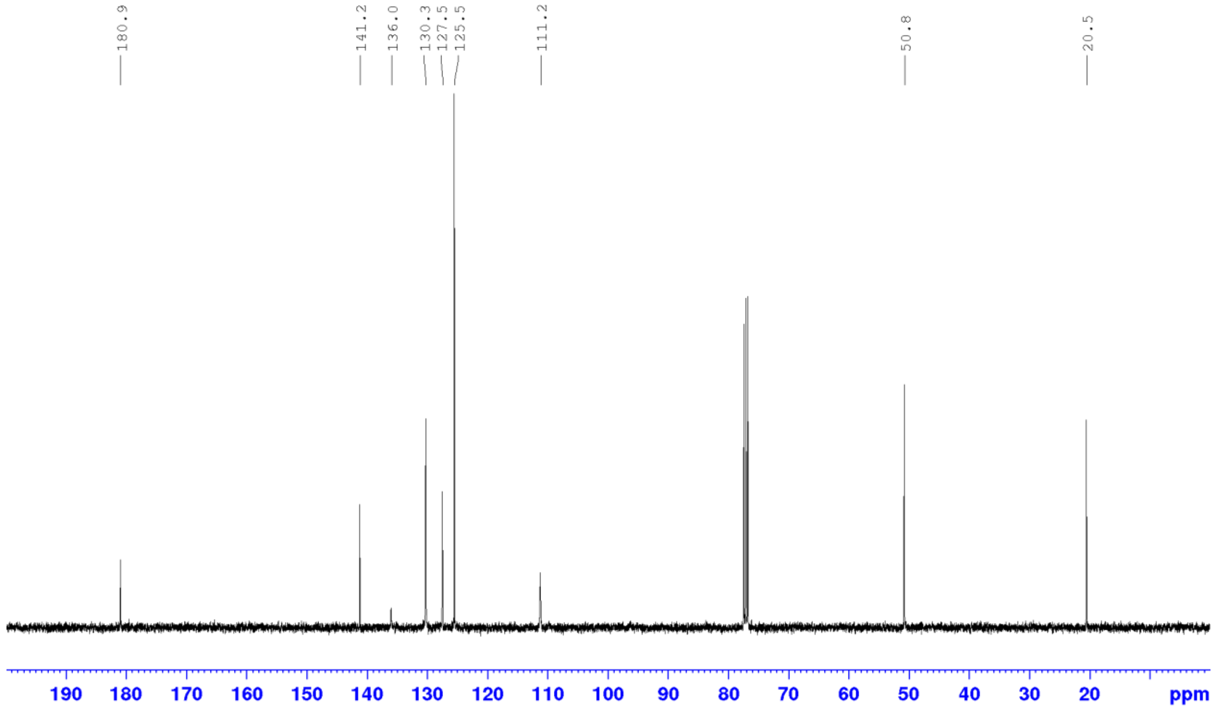

1-Phenyl-3-(2-phenylallyl)thiourea (1ca)

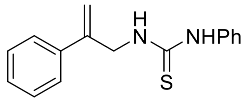

1ca
^{1}H NMR

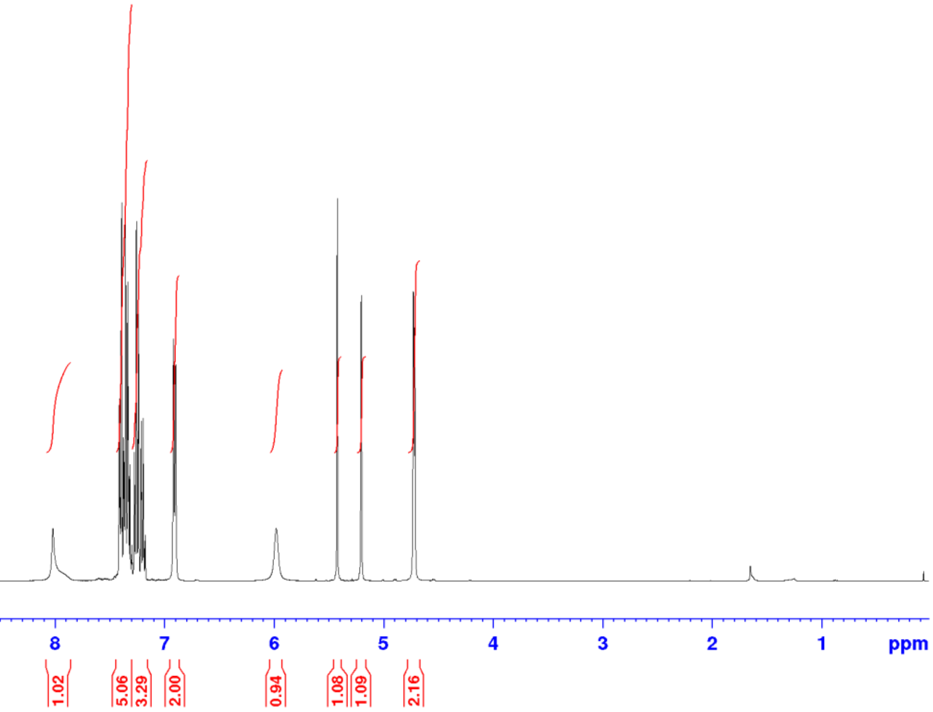

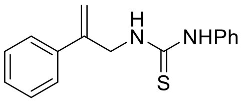

1ca
^{13}C NMR

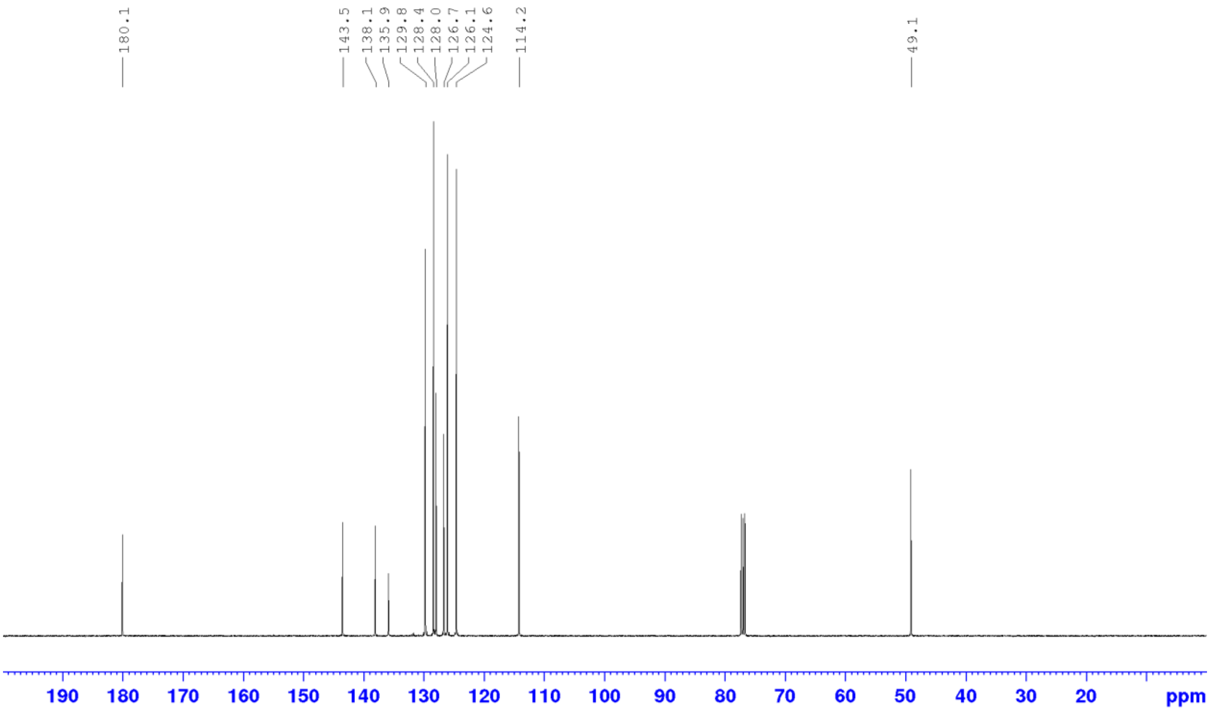

1-(2-(4-fluorophenyl)allyl)-3-phenylthiourea (1da)

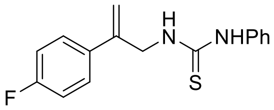

1da
^{1}H NMR

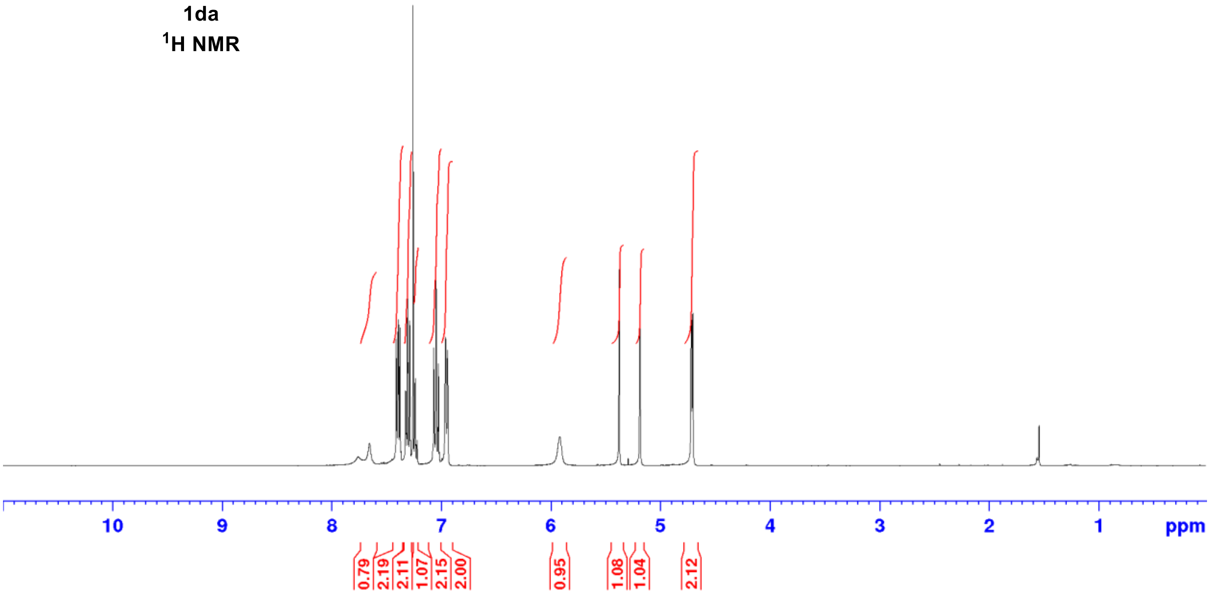

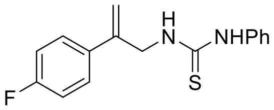

1da
^{13}C NMR

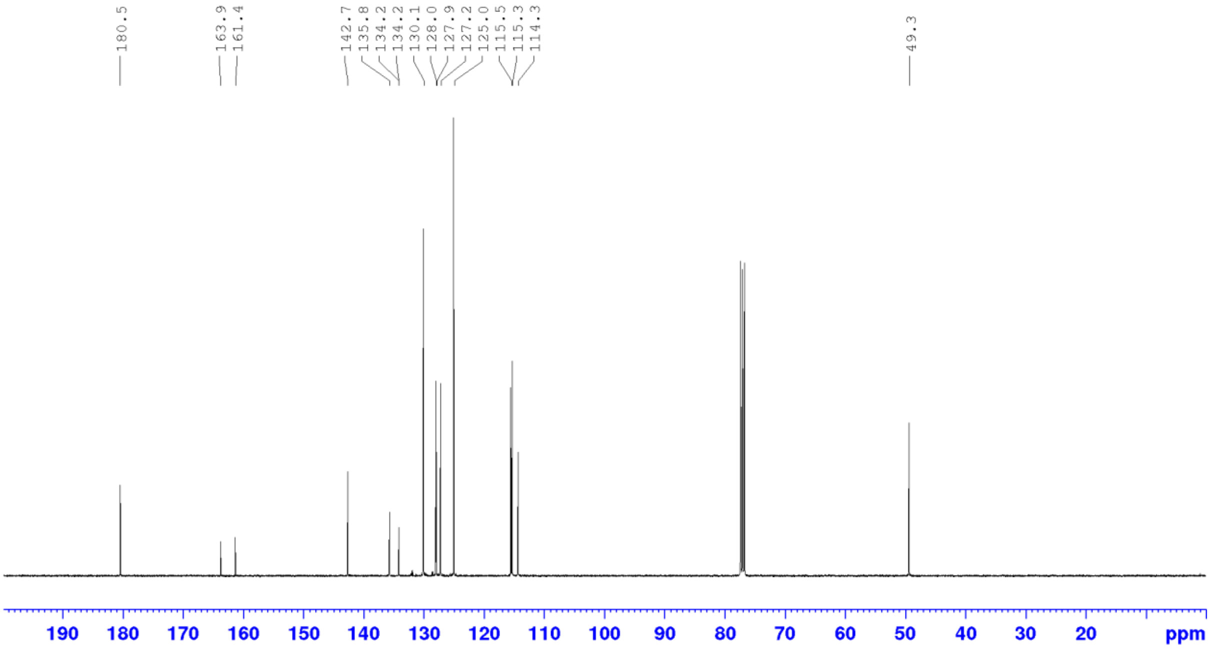

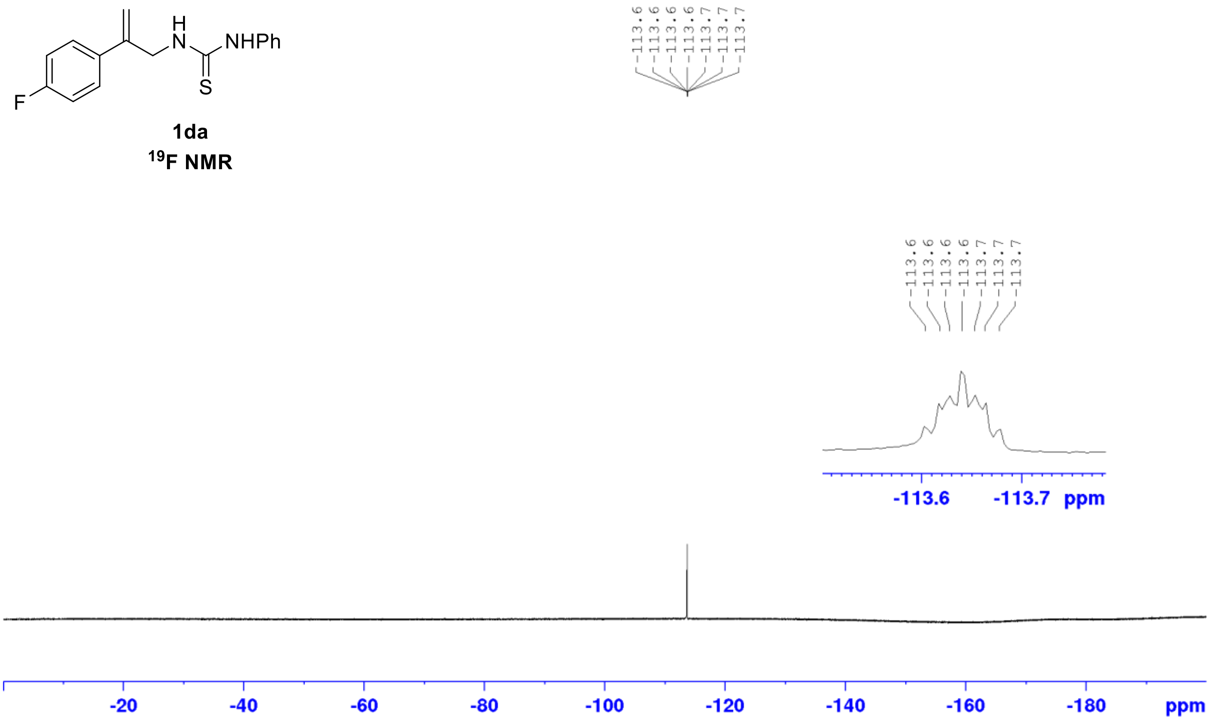

1-(2-(4-chlorophenyl)allyl)-3-phenylthiourea (1ea)

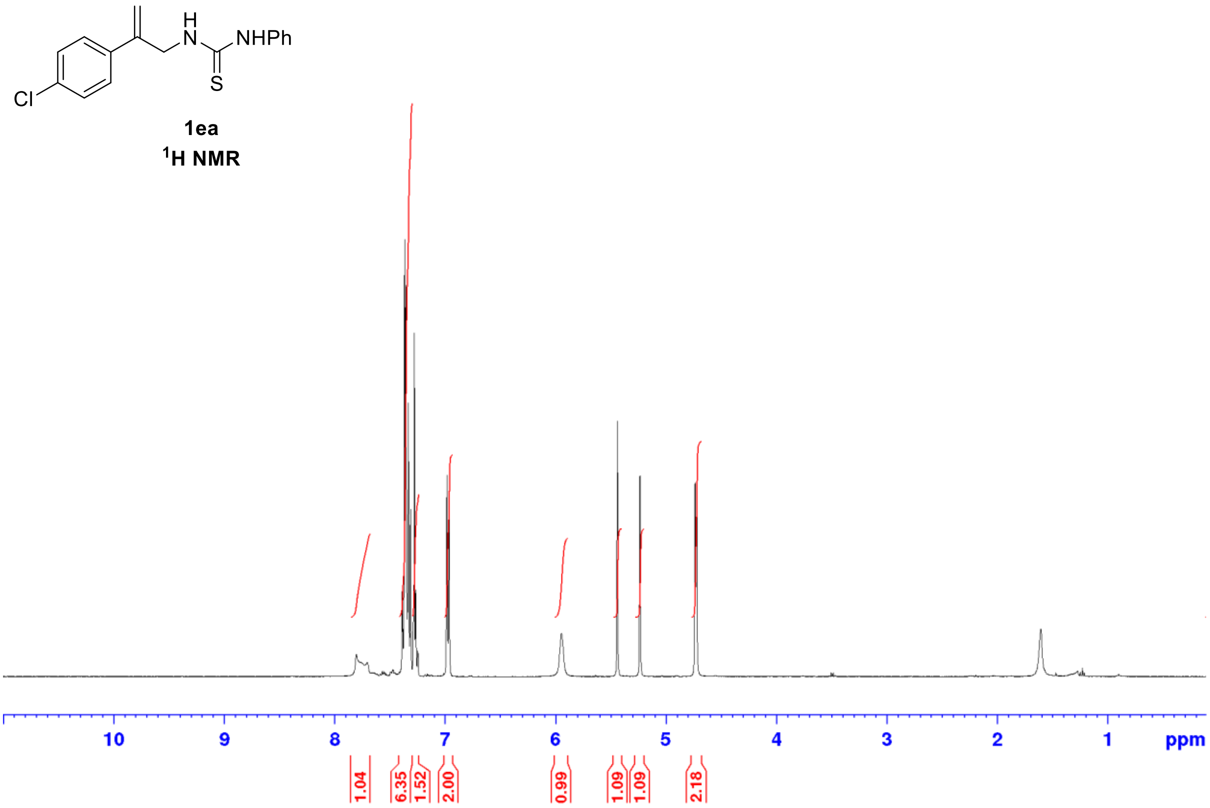

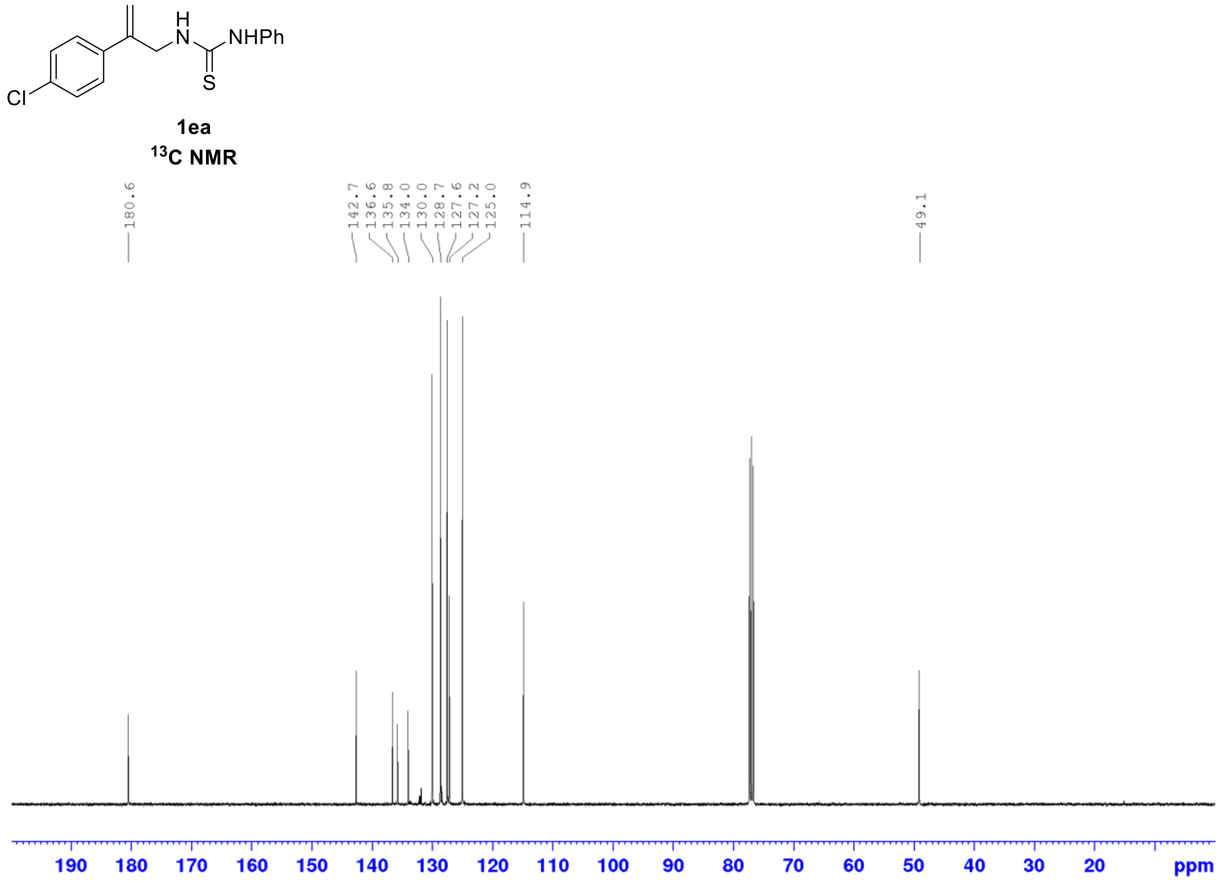

1-phenyl-3-(2-(4-(trifluoromethyl)phenyl)allyl)thiourea (1fa)

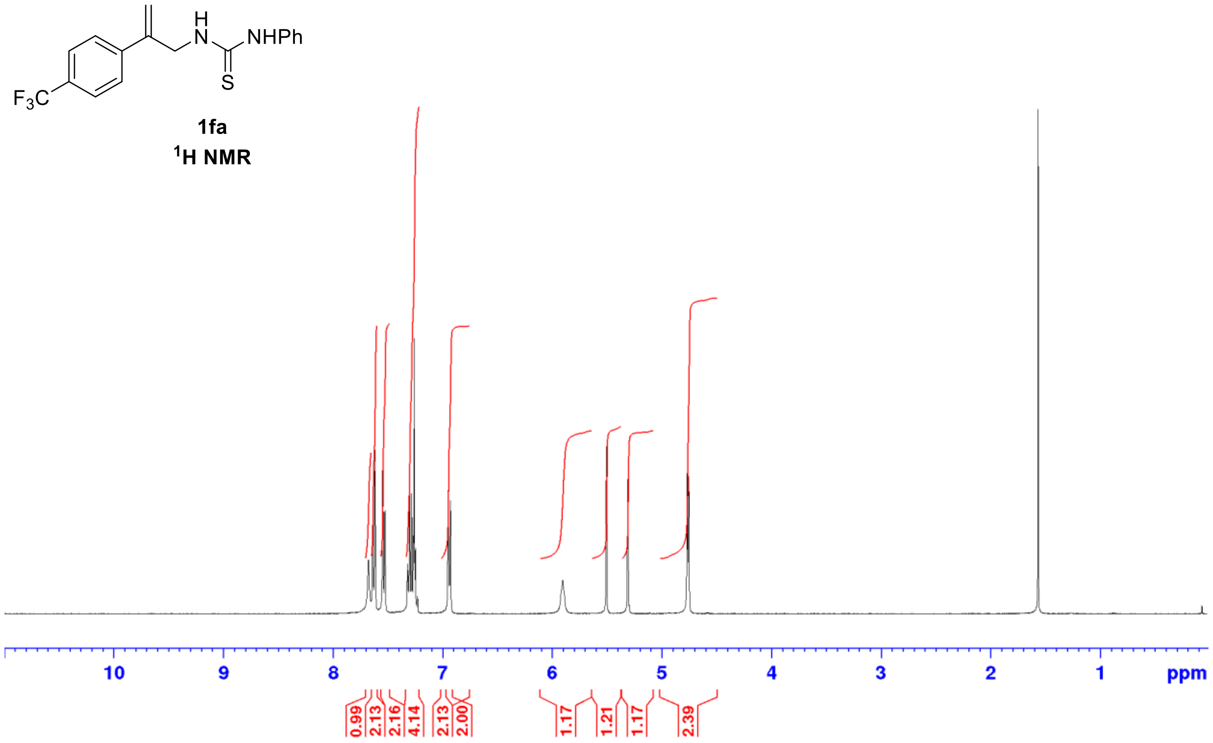

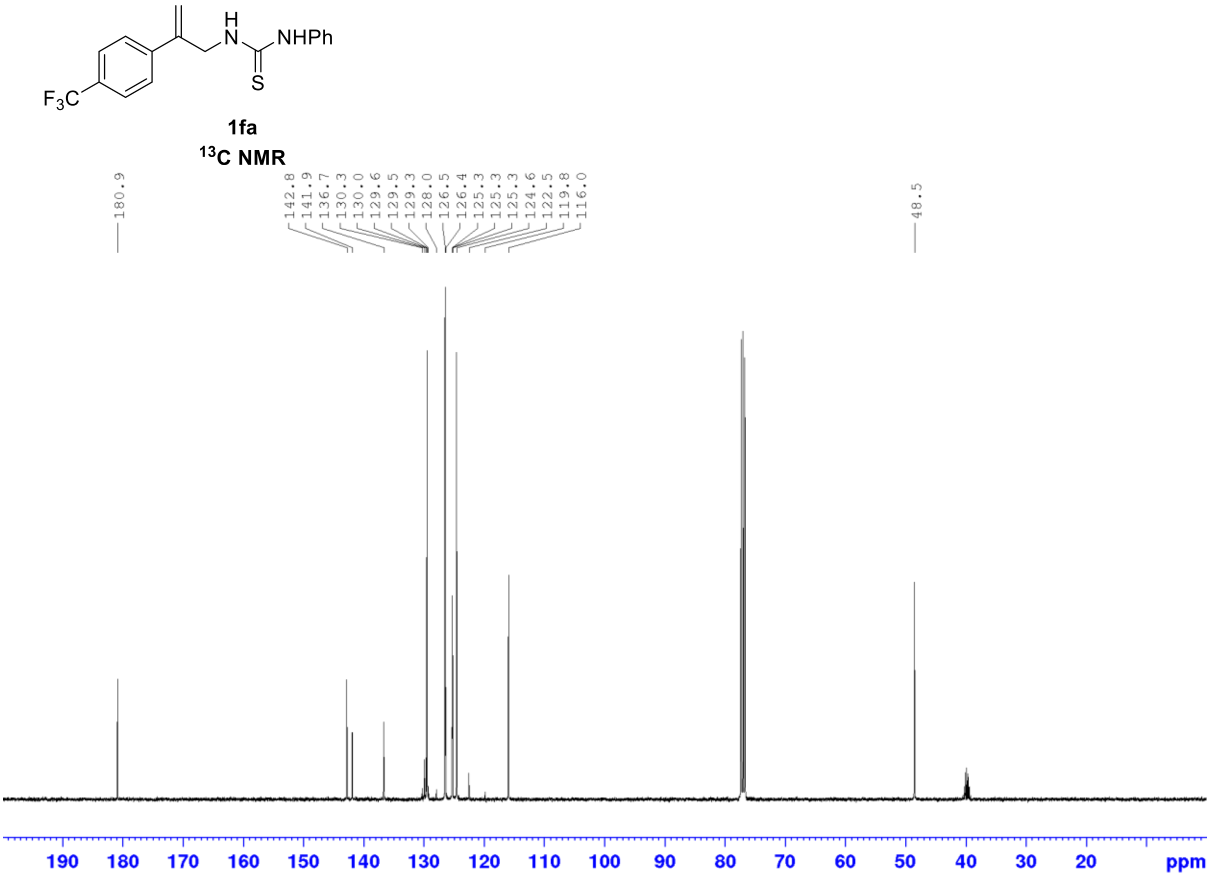

NHPh
H
N
S
F_3C
1fa
^{13}C NMR
180.9
142.8
141.9
136.7
130.3
130.0
129.6
129.5
129.3
128.0
126.5
126.4
125.3
125.3
125.3
124.6
122.5
119.8
116.0
48.5
190 180 170 160 150 140 130 120 110 100 90 80 70 60 50 40 30 20 ppm

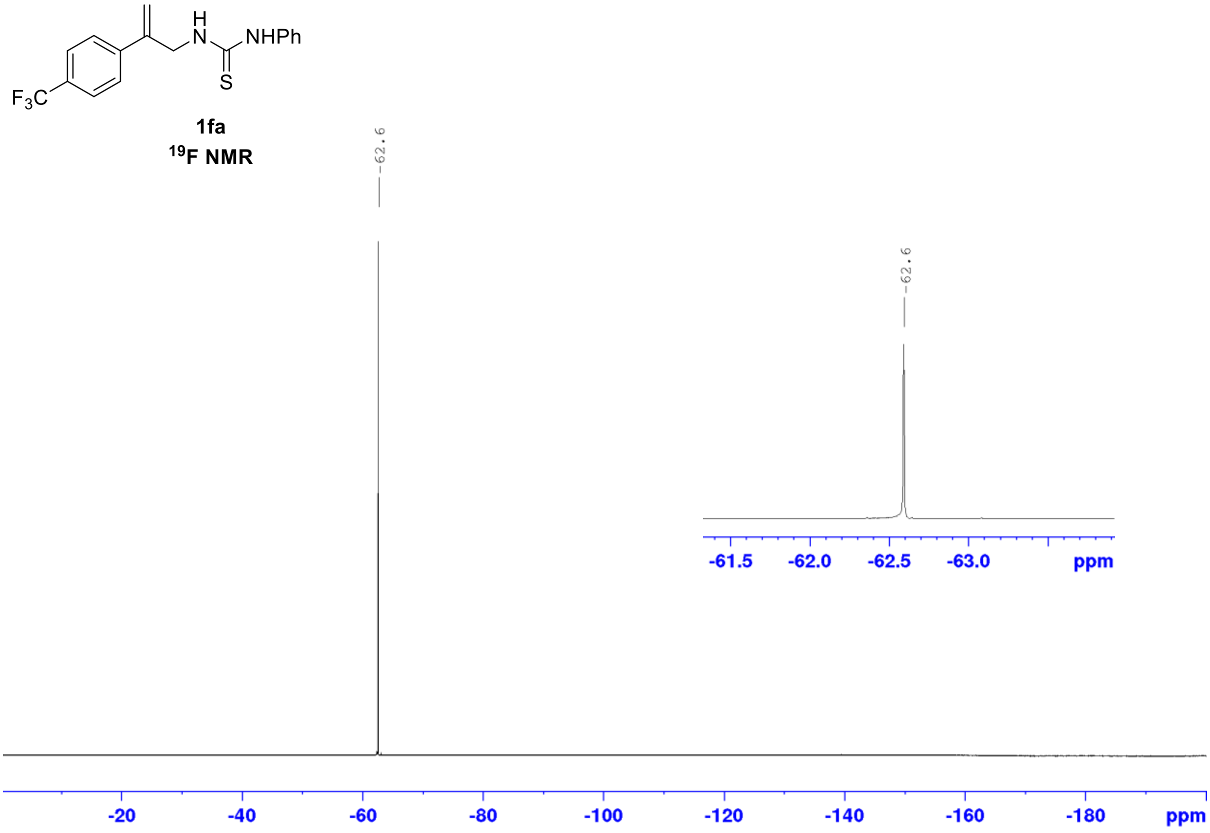

NHPh
H
N
S
F_3C
1fa
^{19}F NMR
-62.6
-62.6
-61.5 -62.0 -62.5 -63.0 ppm
-20 -40 -60 -80 -100 -120 -140 -160 -180 ppm

1-((3,4-Dihydronaphthalen-1-yl)methyl)-3-phenylthiourea (1ga)

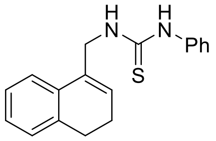

1ga

^{1}H NMR

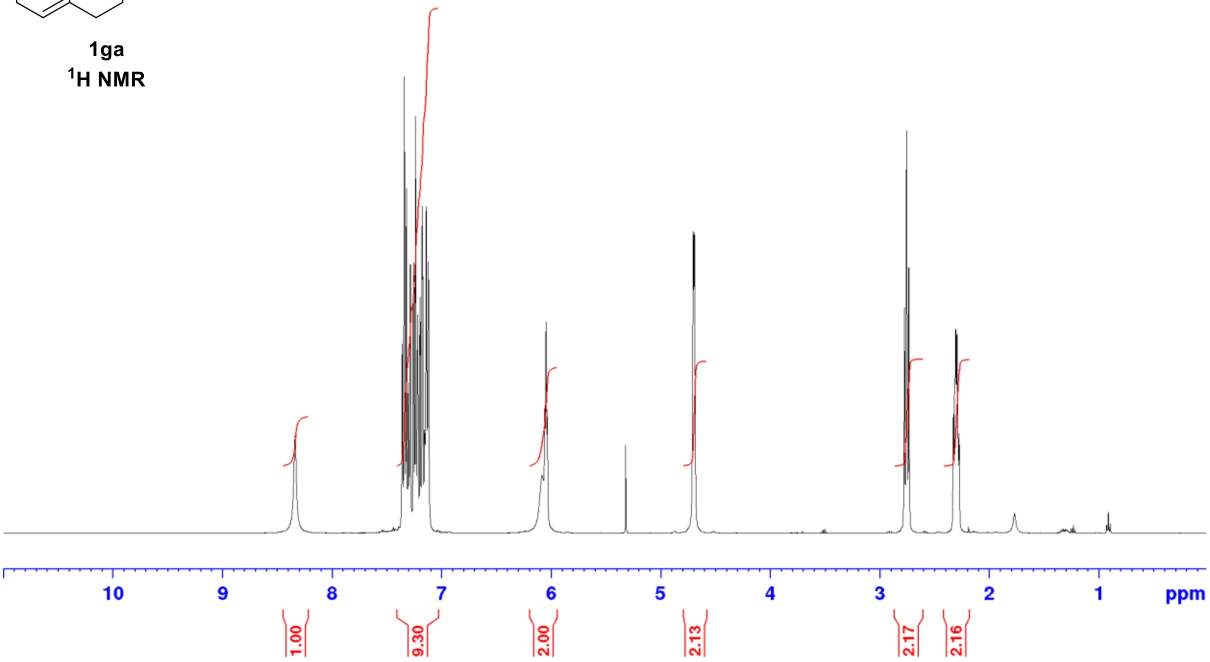

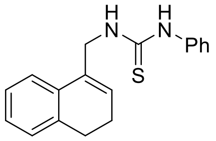

1ga

^{13}C NMR

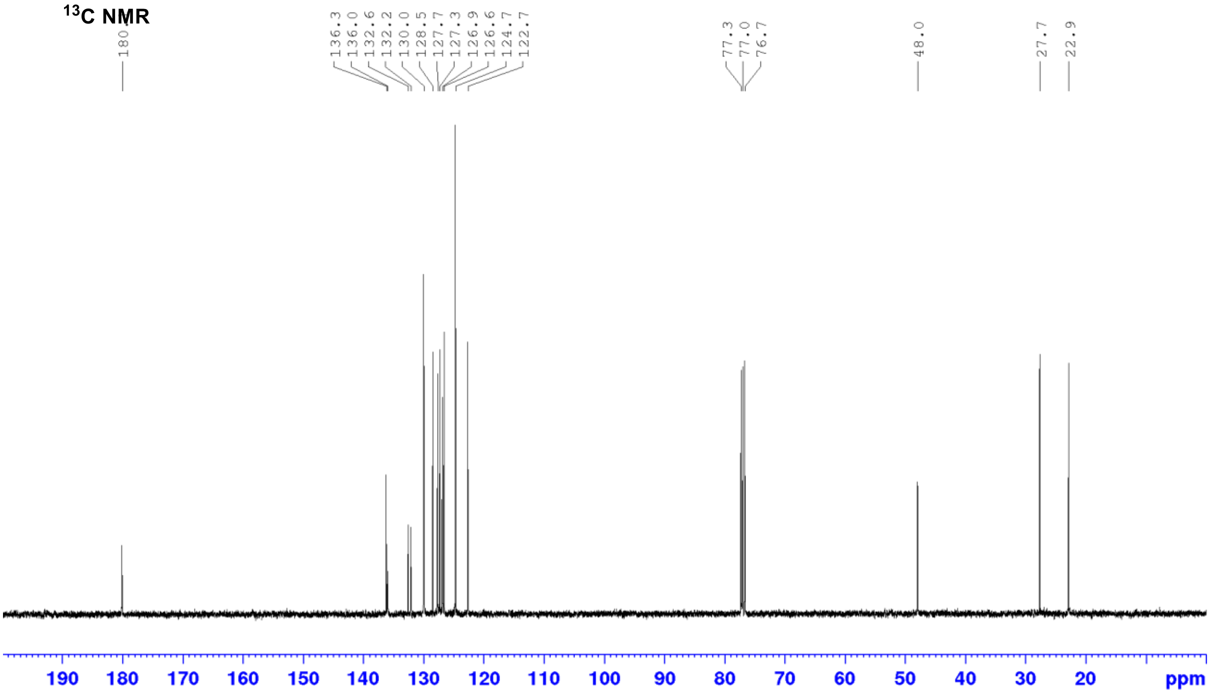

1-(4-Bromophenyl)-3-((3,4-dihydronaphthalen-1-yl)methyl)thiourea (1gb)

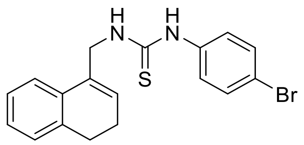

1gb
^{1}H NMR

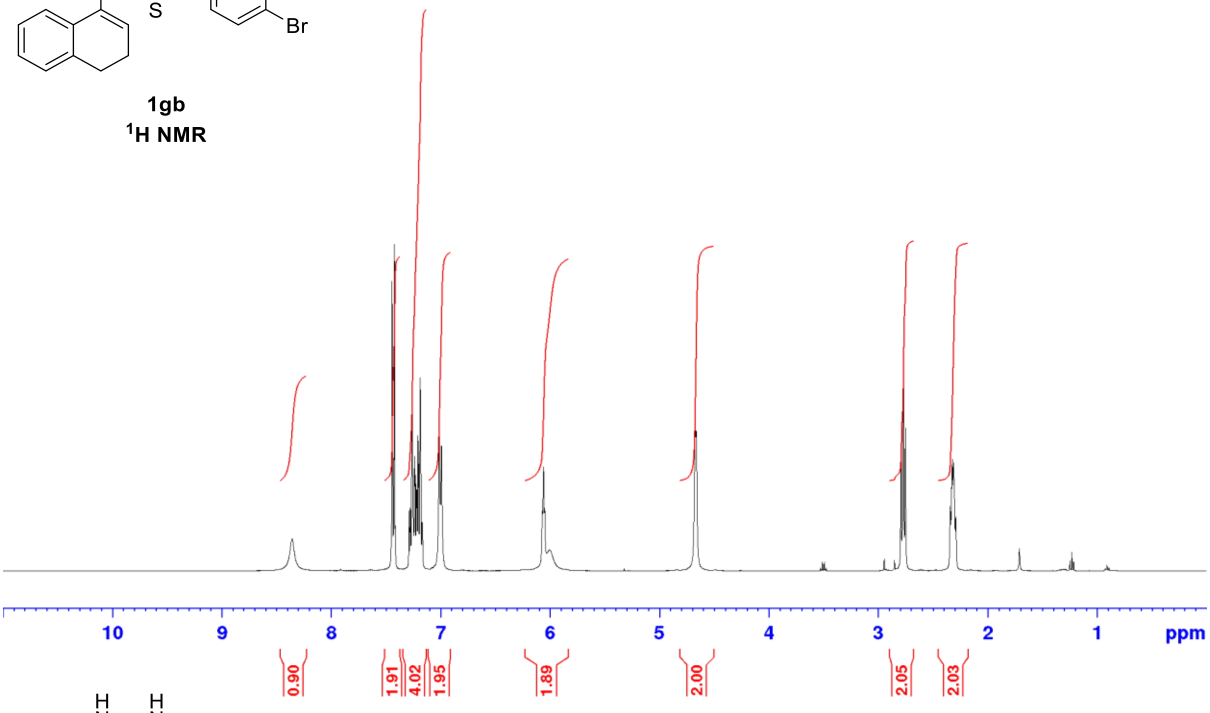

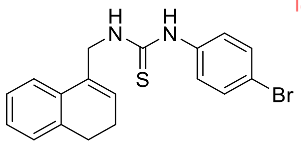

1gb
^{13}C NMR

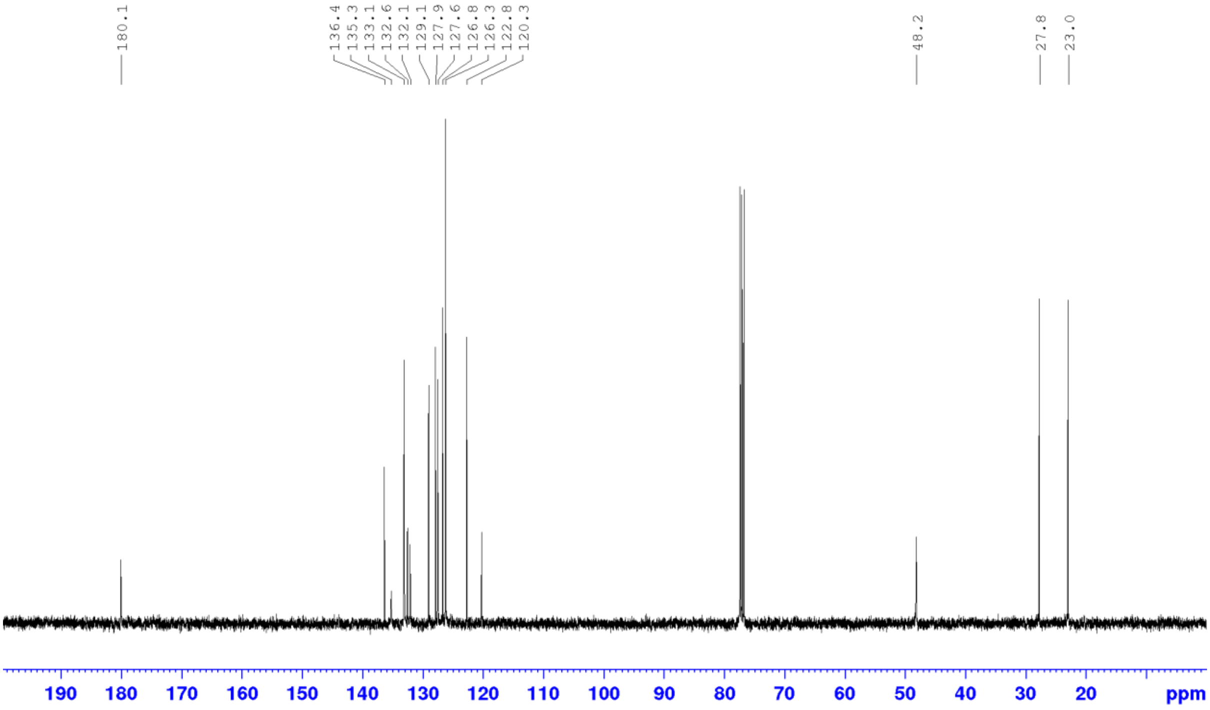

1-(4-Iodophenyl)-3-((3,4-dihydronaphthalen-1-yl)methyl)thiourea (1gc)

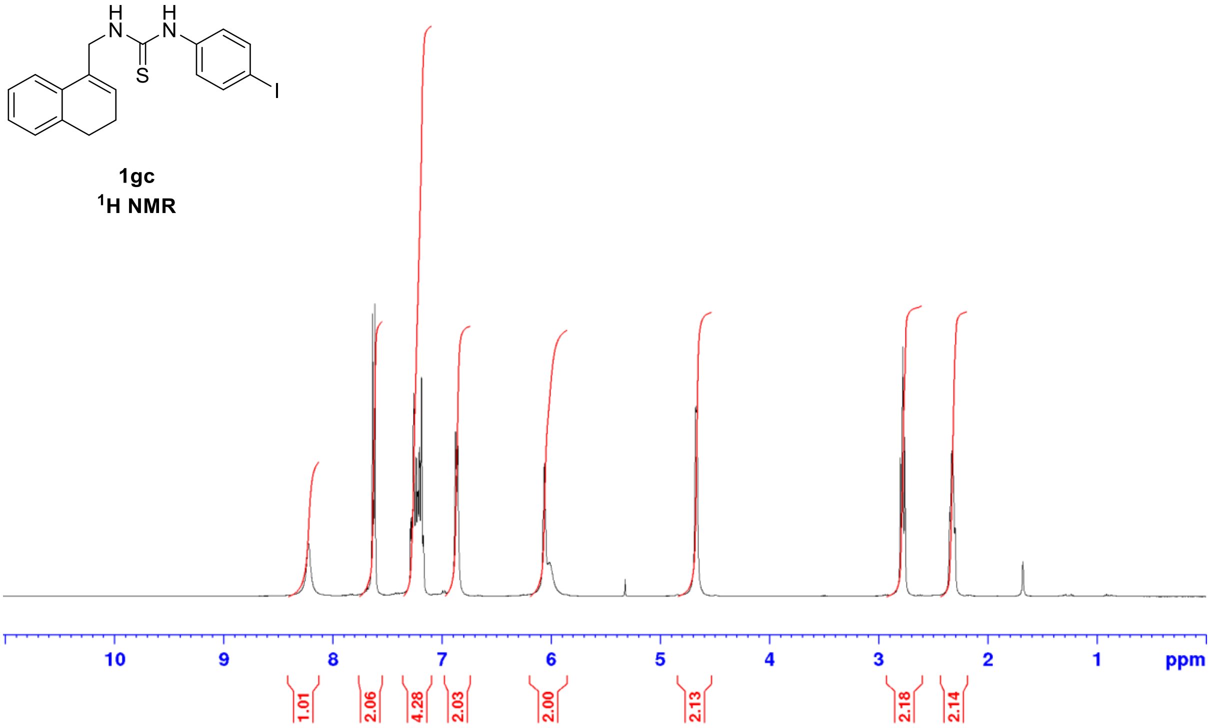

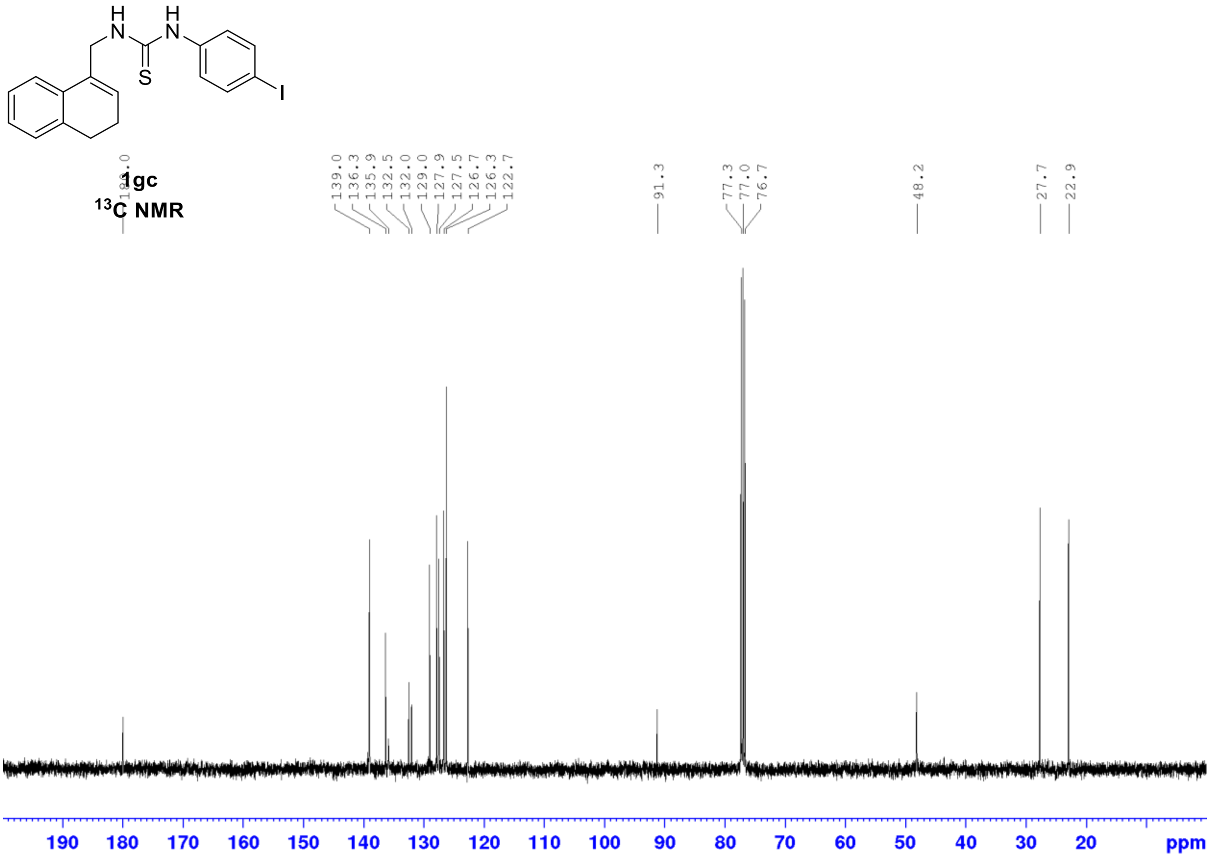

1-((3,4-Dihydronaphthalen-1-yl)methyl)-3-(4-methoxyphenyl)thiourea (1gd)

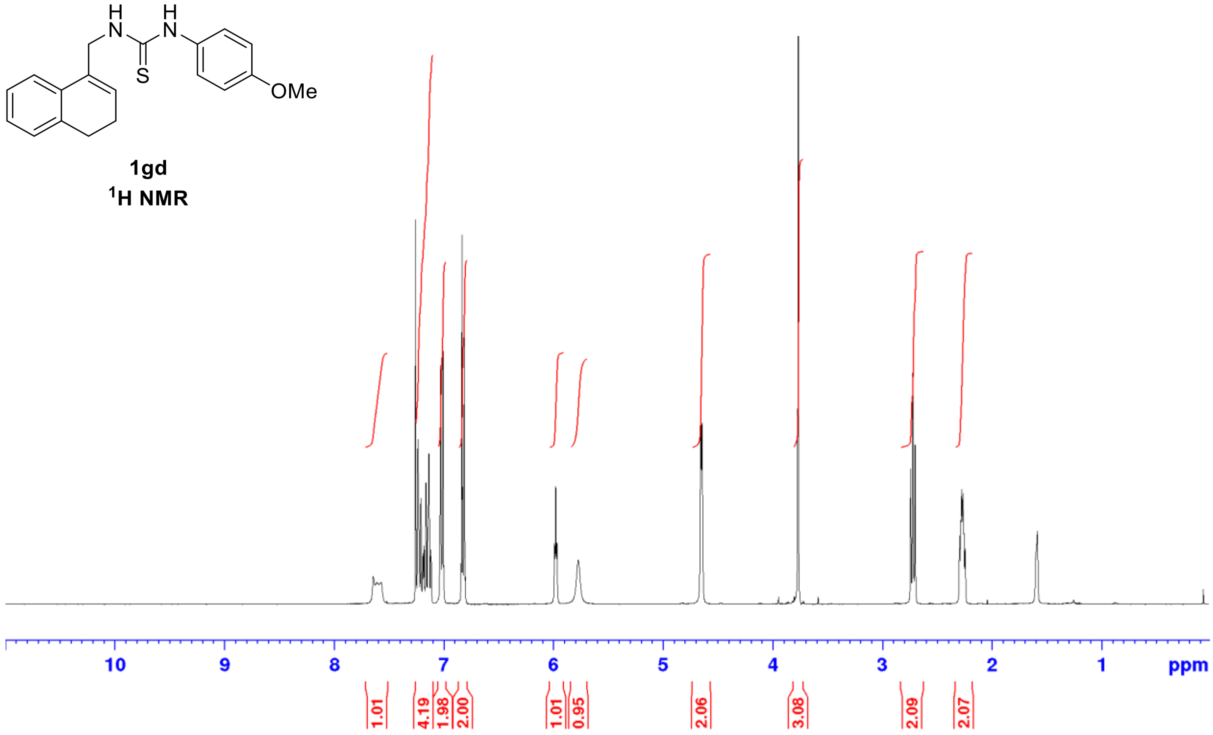

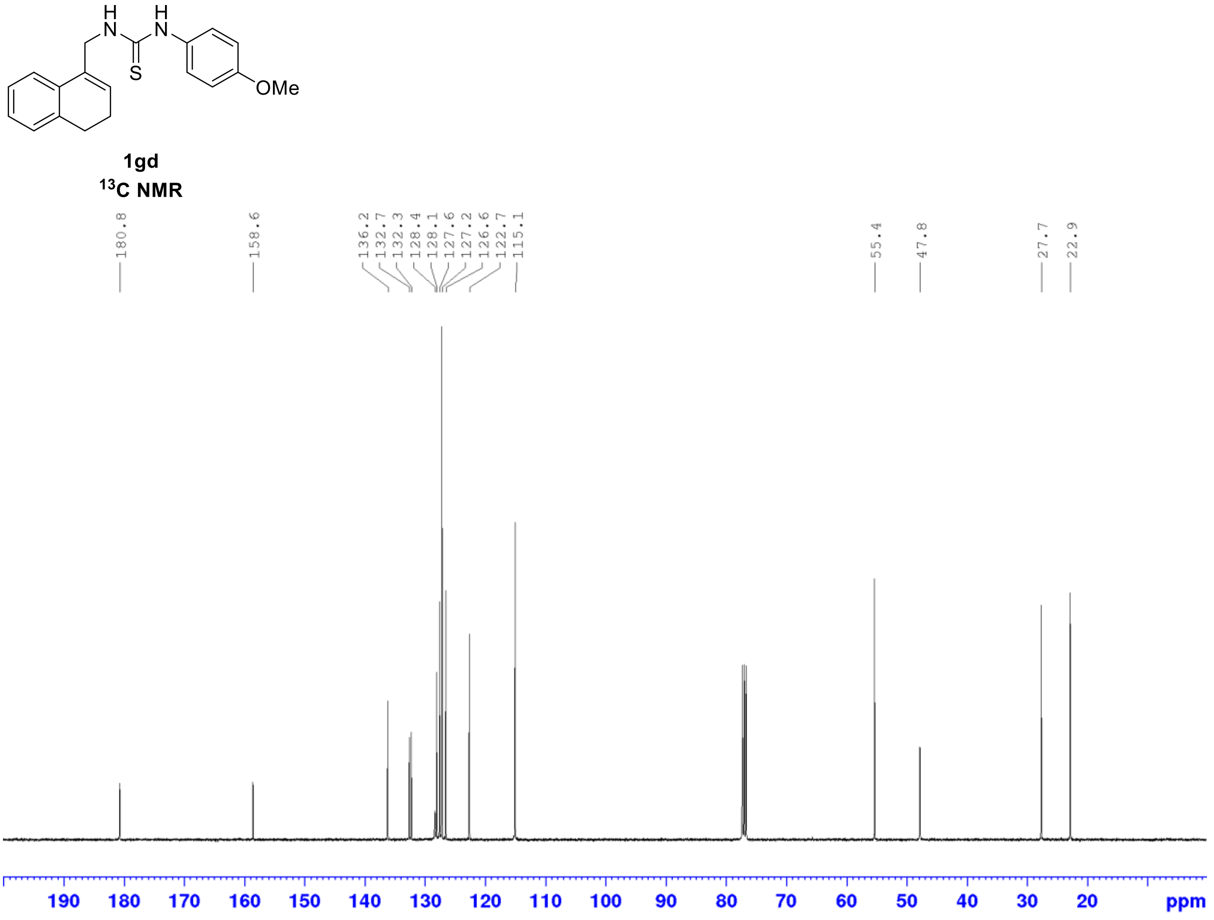

1-((3,4-Dihydronaphthalen-1-yl)methyl)-3-(naphthalen-2-yl)thiourea (1ge)

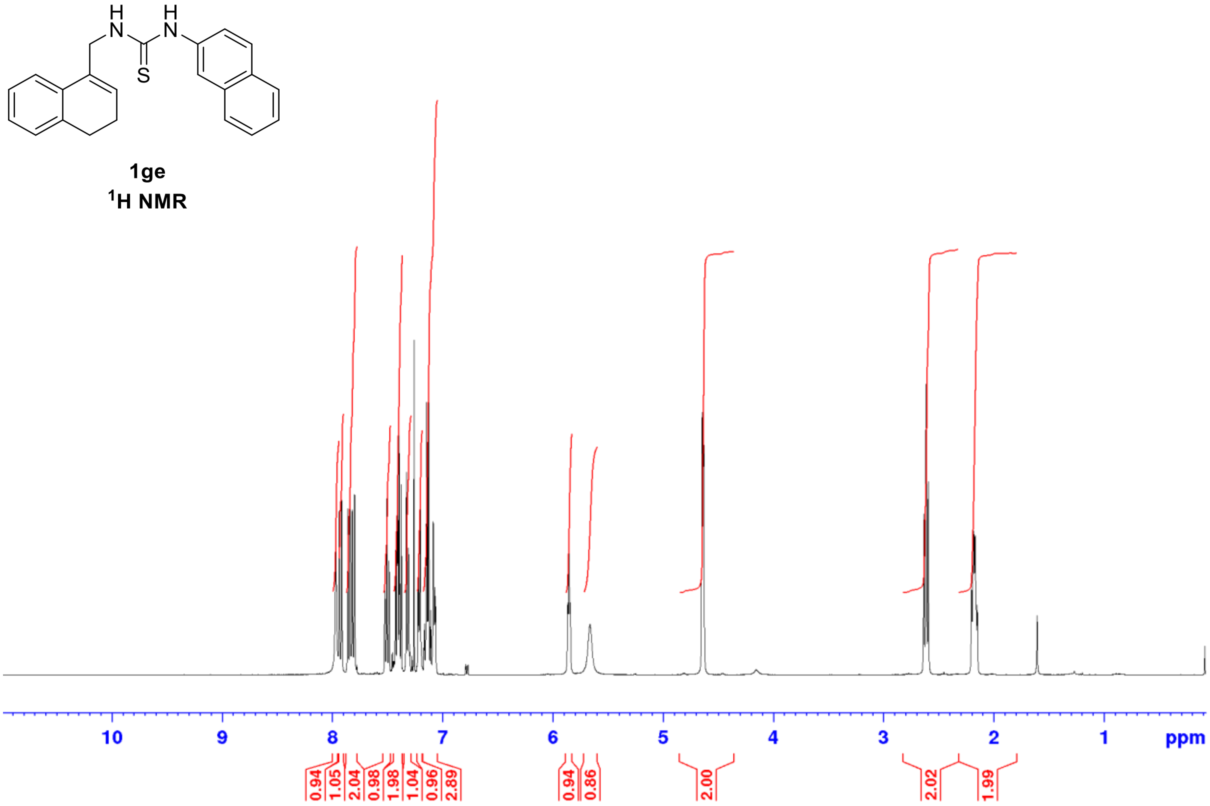

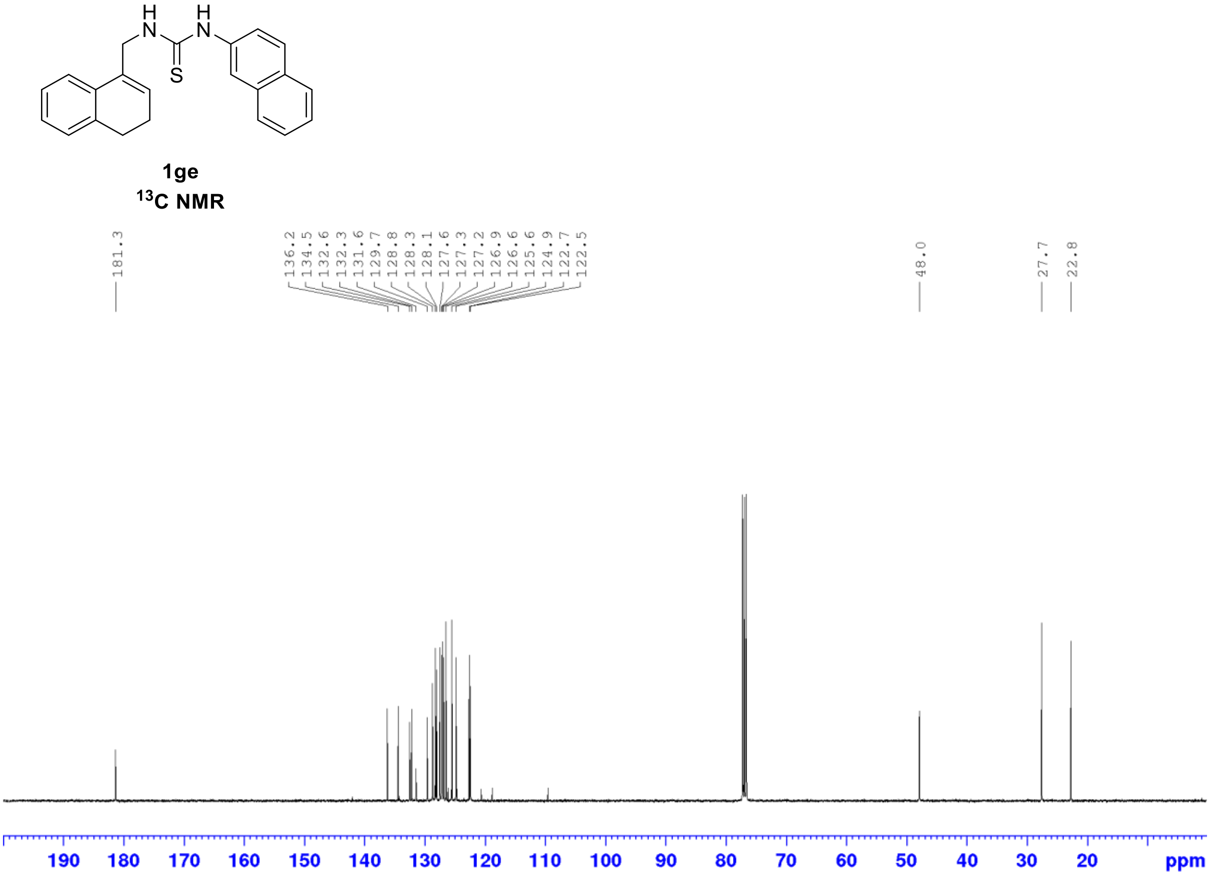

1-((3,4-Dihydronaphthalen-1-yl)methyl)-3-(4-(trifluoromethyl)phenyl)thiourea (1gf)

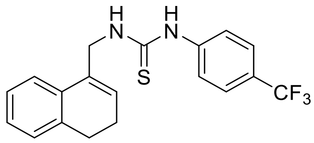

1gf
^{1}H NMR

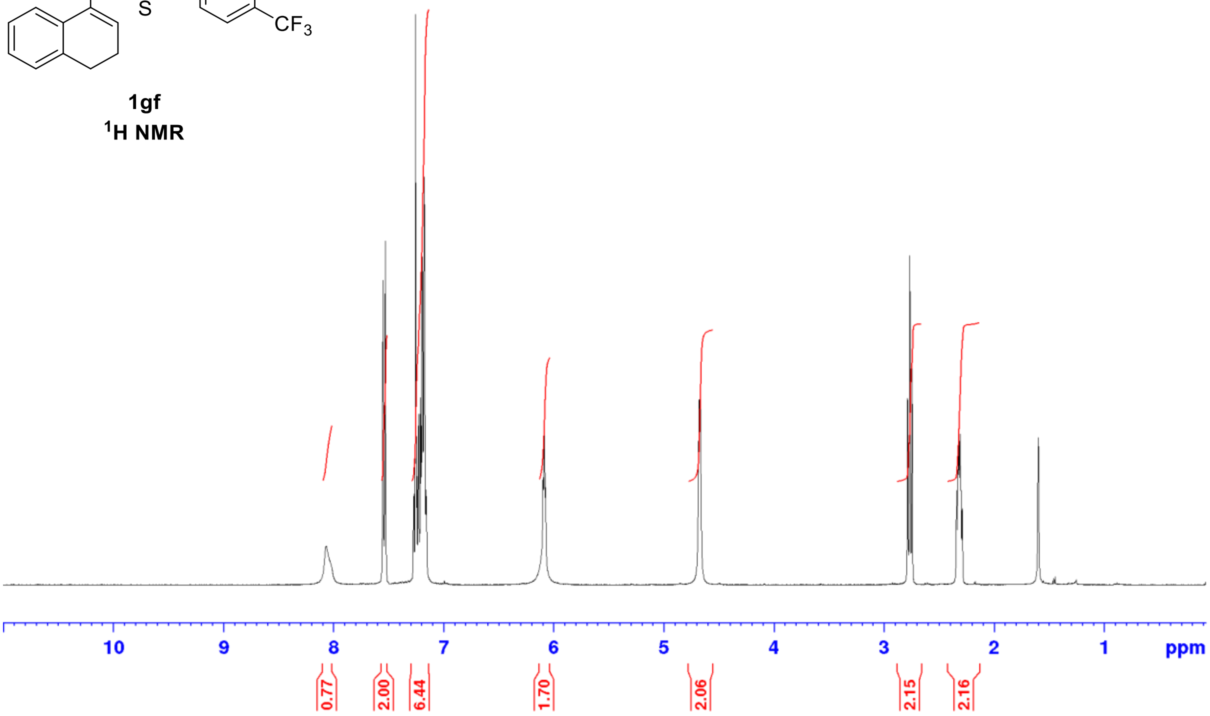

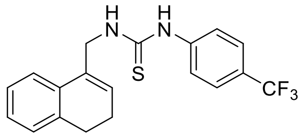

1gf
^{13}C NMR

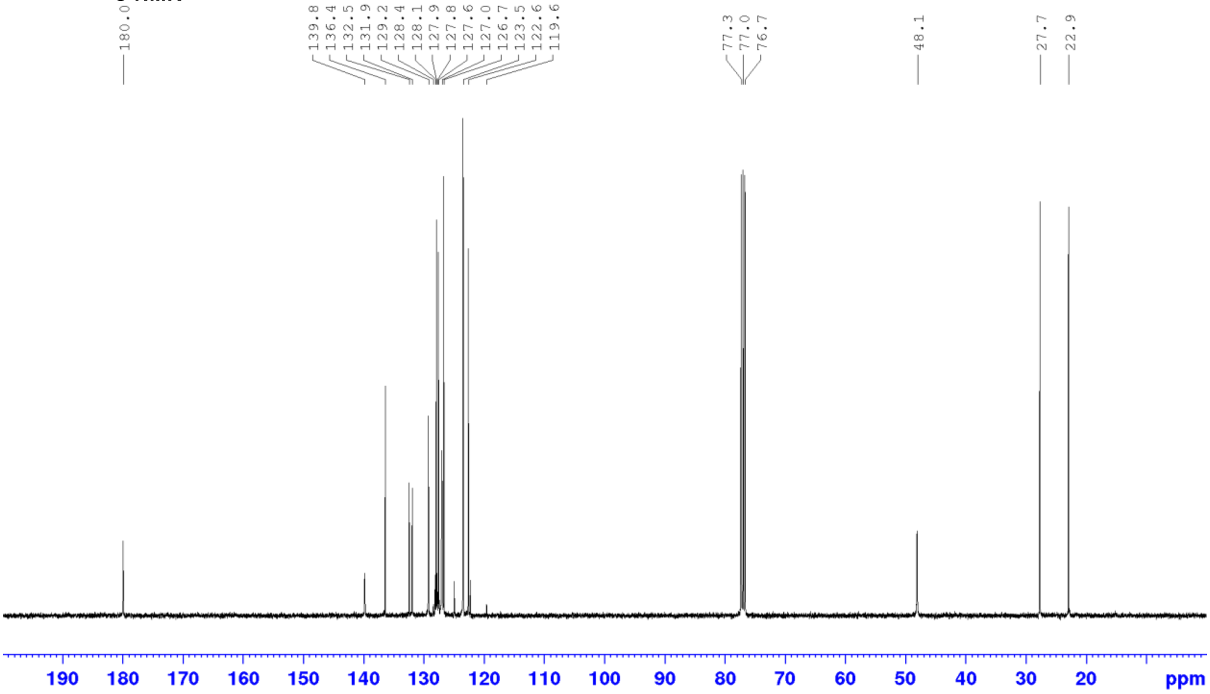

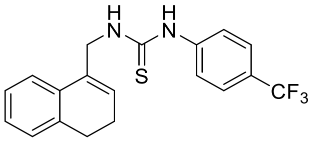

1gf

^{19}F NMR

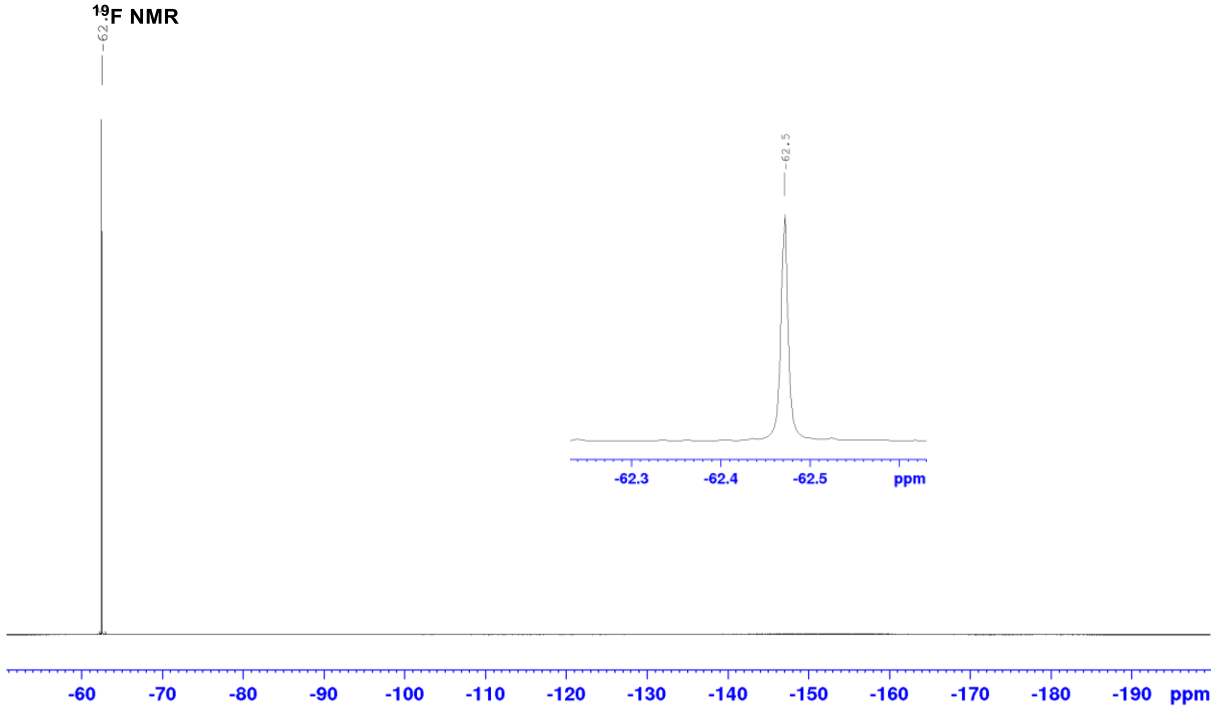

Methyl 4-(3-((3,4-dihydronaphthalen-1-yl)methyl)thioureido)benzoate (1gg)

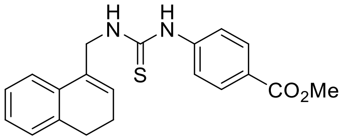

1gg

^{1}H NMR

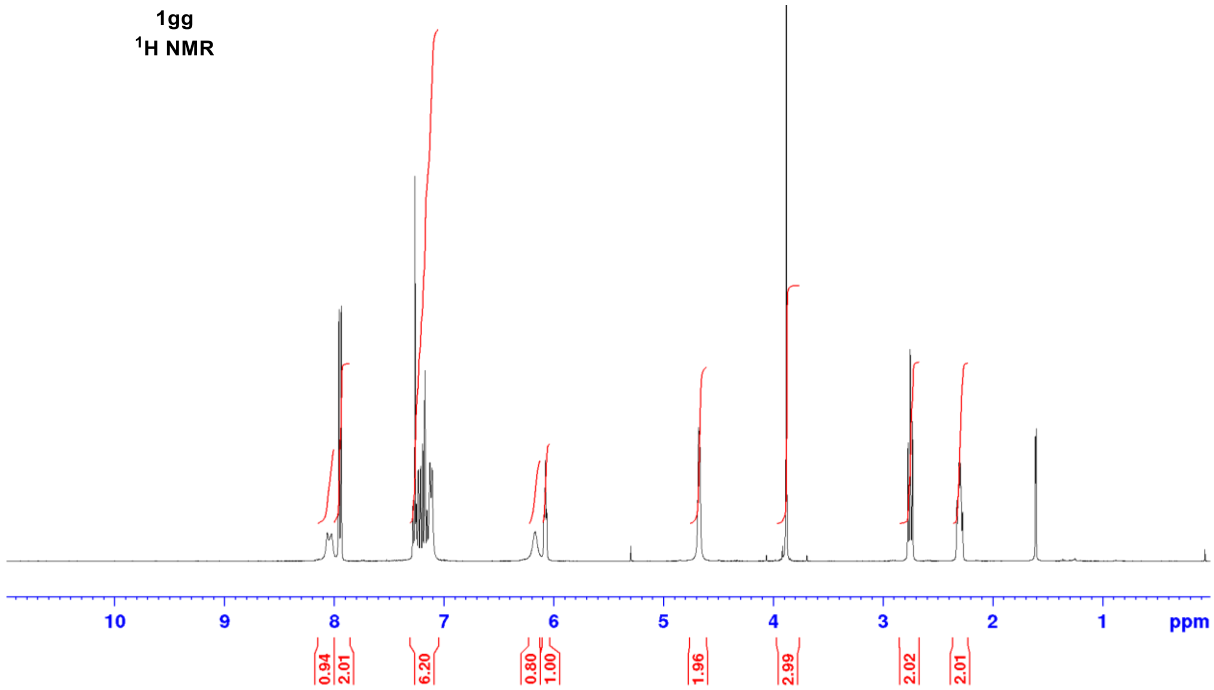

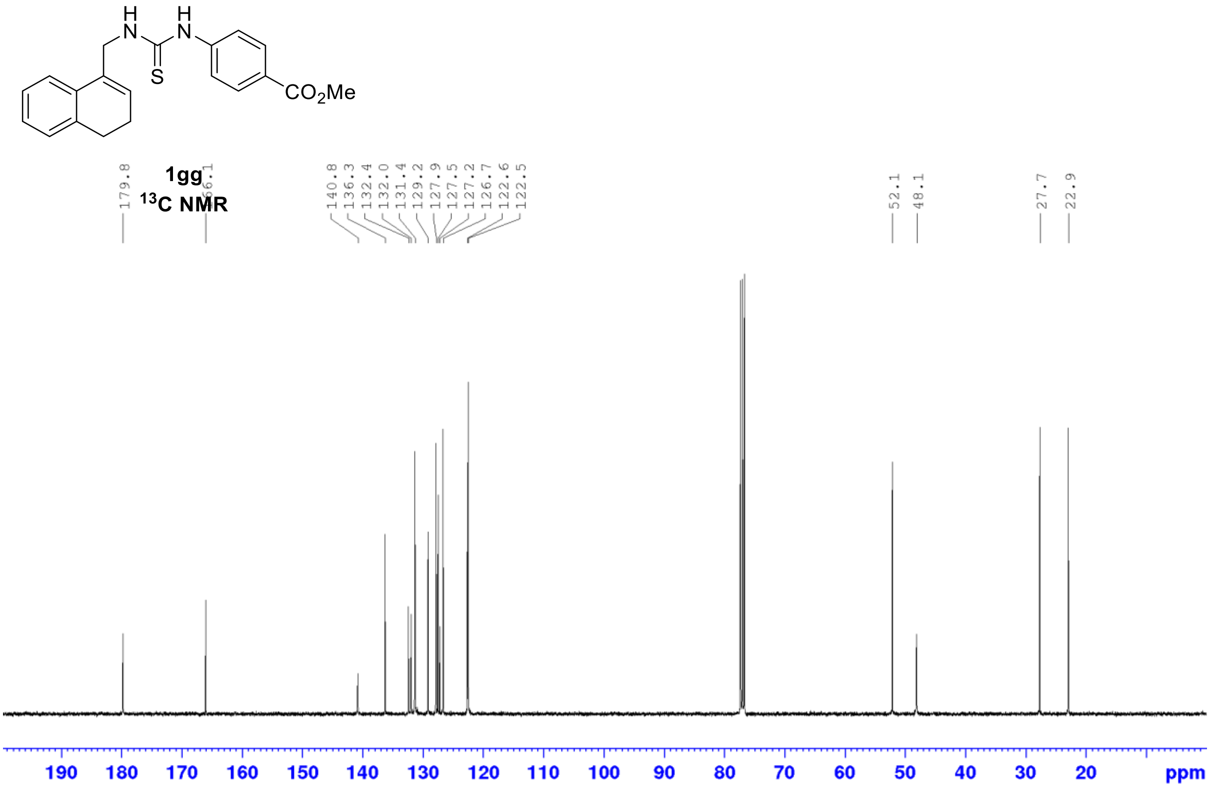

1-Benzyl-3-((3,4-Dihydronaphthalen-1-yl)methyl)thiourea (1gh)

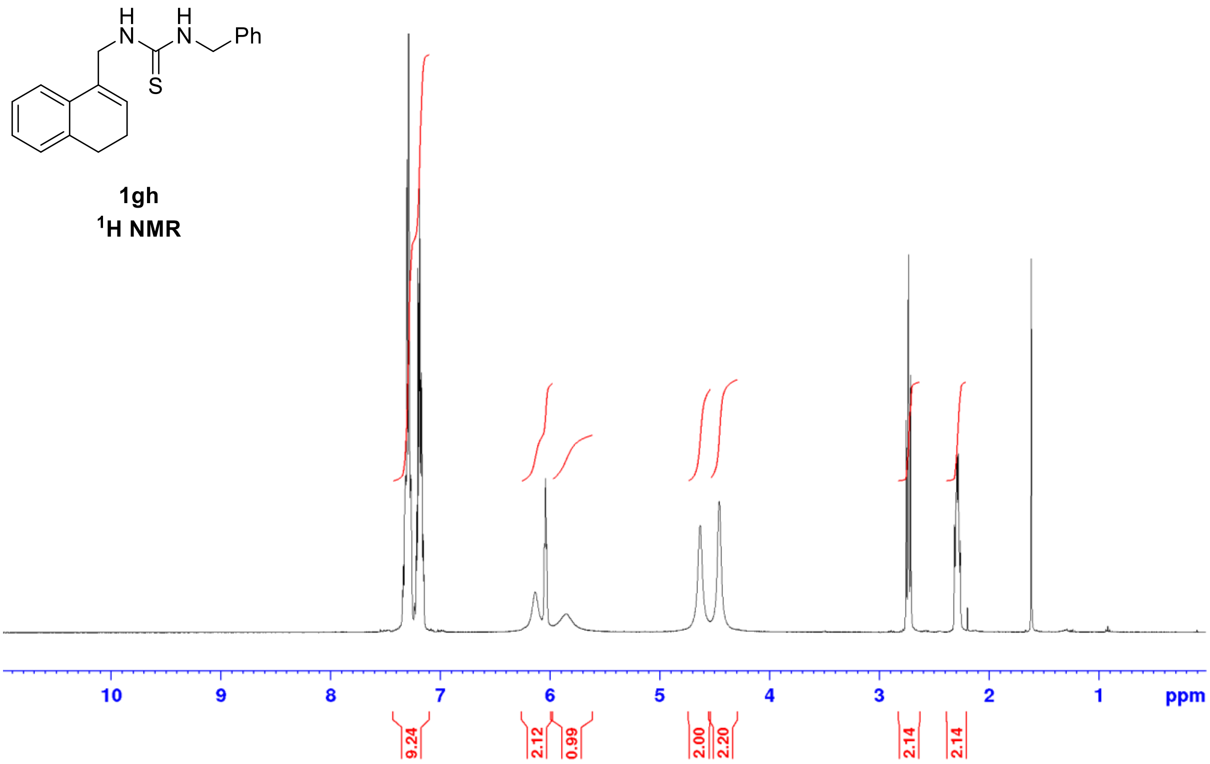

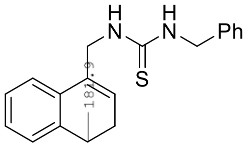

1gh
^{13}C NMR

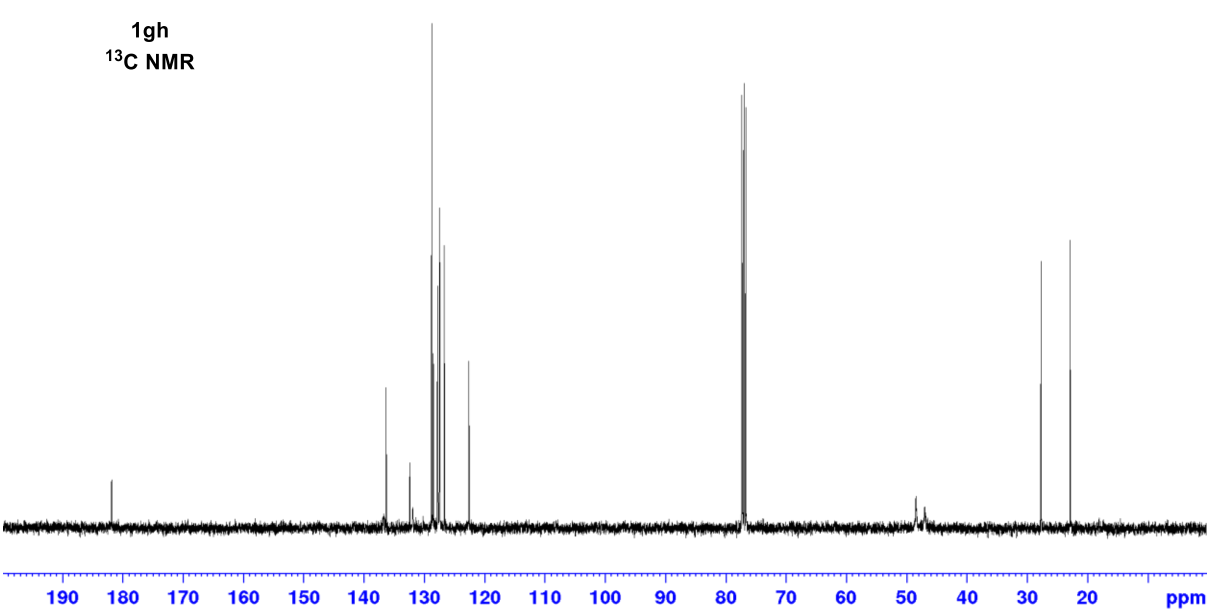

1-(*tert*-Butyl)-3-((3,4-dihydronaphthalen-1-yl)methyl)thiourea (1gi)

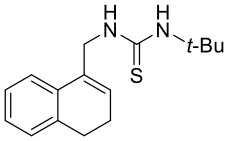

1gi
^{1}H NMR

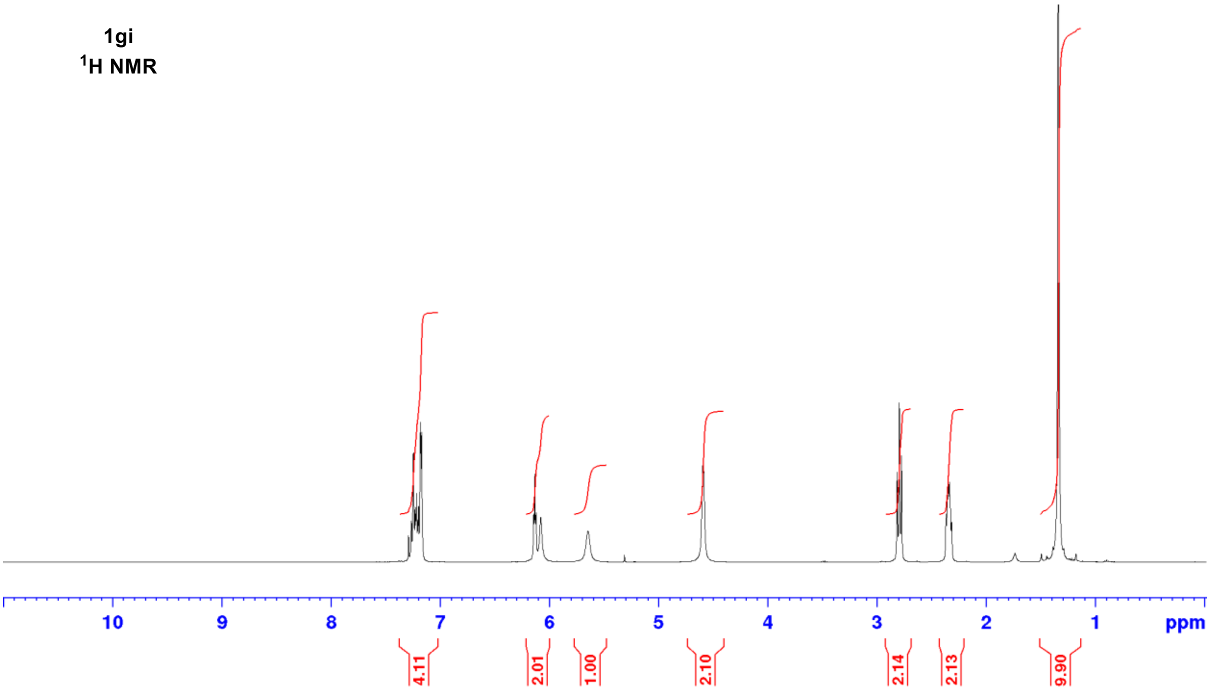

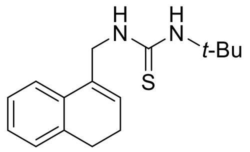

1gi
^{13}C NMR

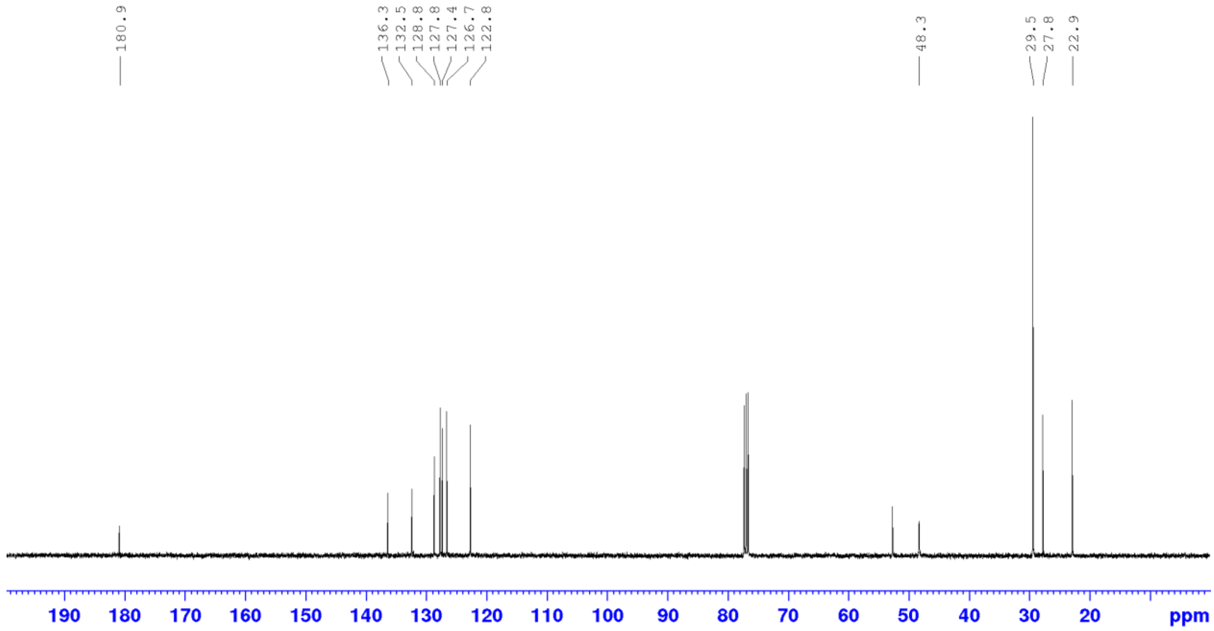

1-(Benzo[d]thiazol-2-yl)-3-((3,4-dihydronaphthalen-1-yl)methyl)thiourea (1gj)

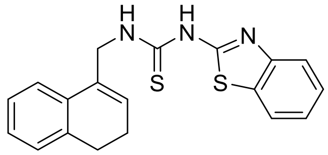

1gj
^{1}H NMR

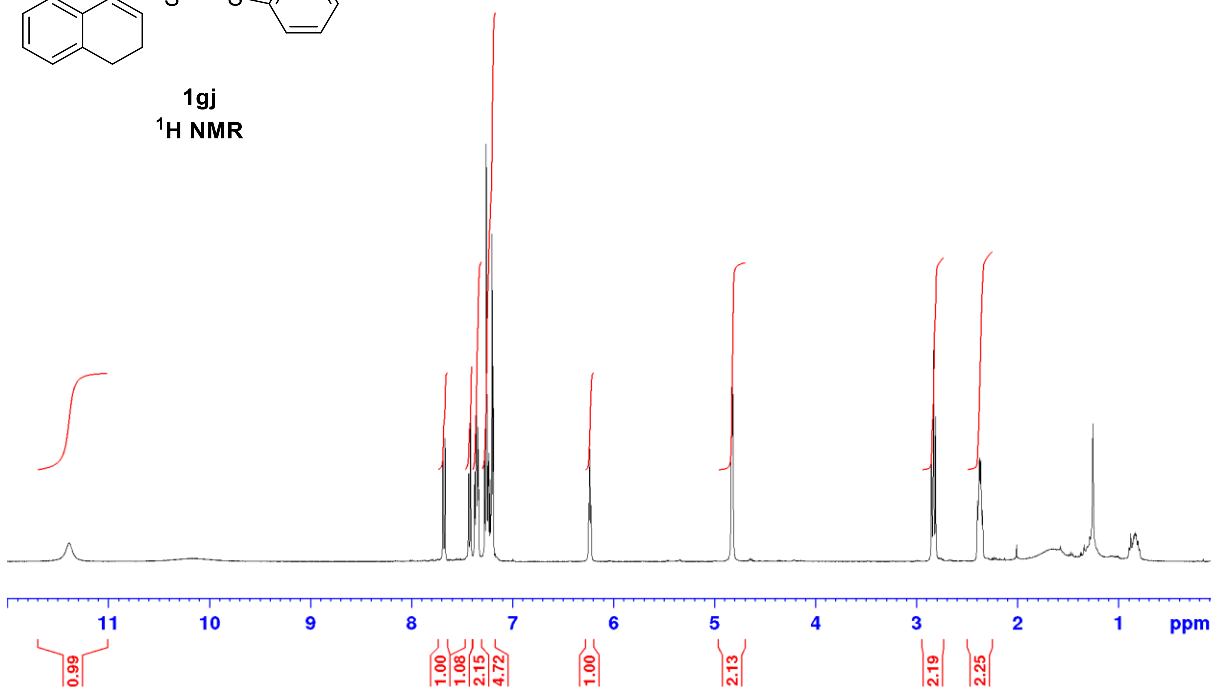

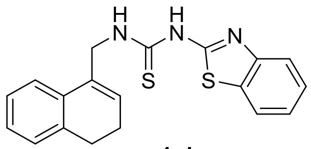

1gj

^{13}C NMR

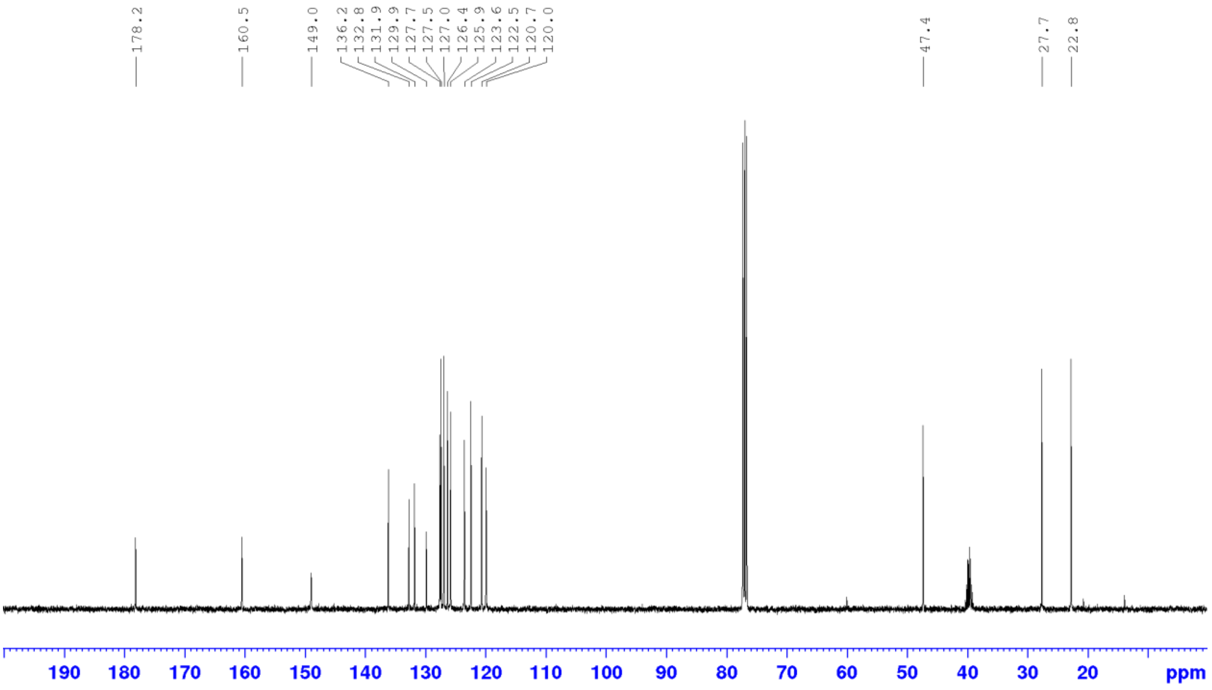

1-((3,4-dihydronaphthalen-1-yl)methyl)-3-(6-methylpyridin-3-yl)thiourea (1gk)

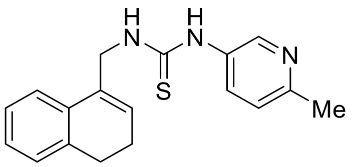

1gk

^{1}H NMR

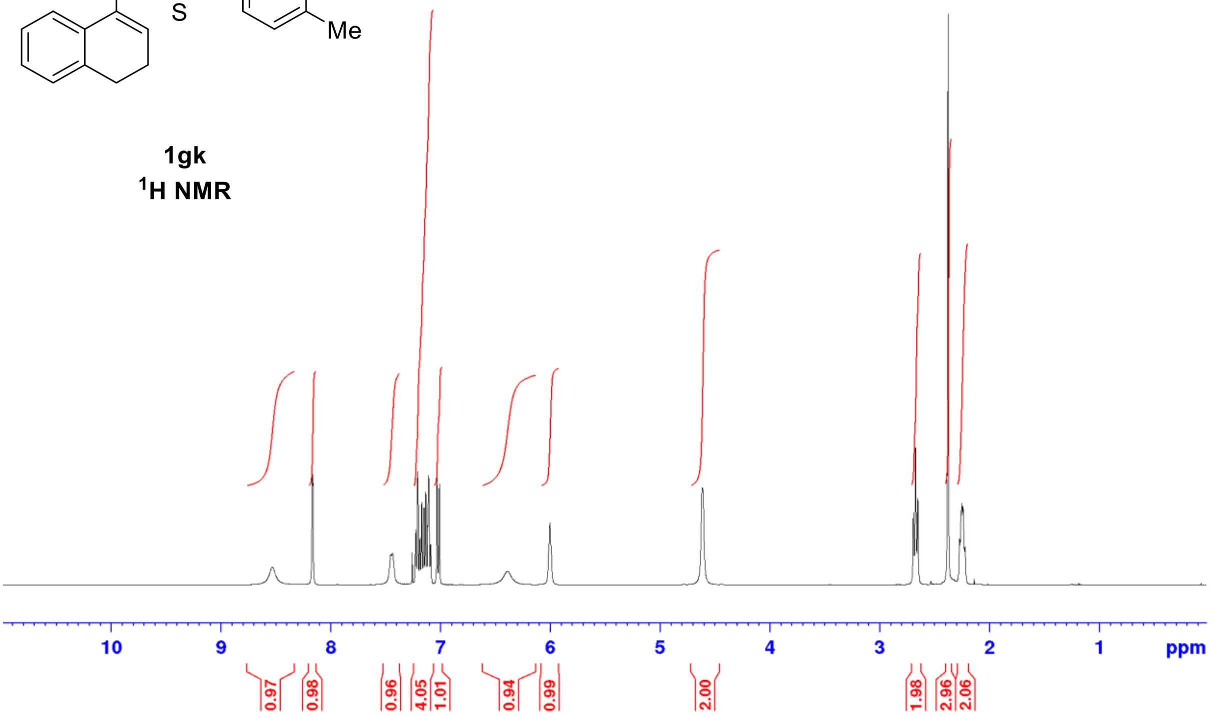

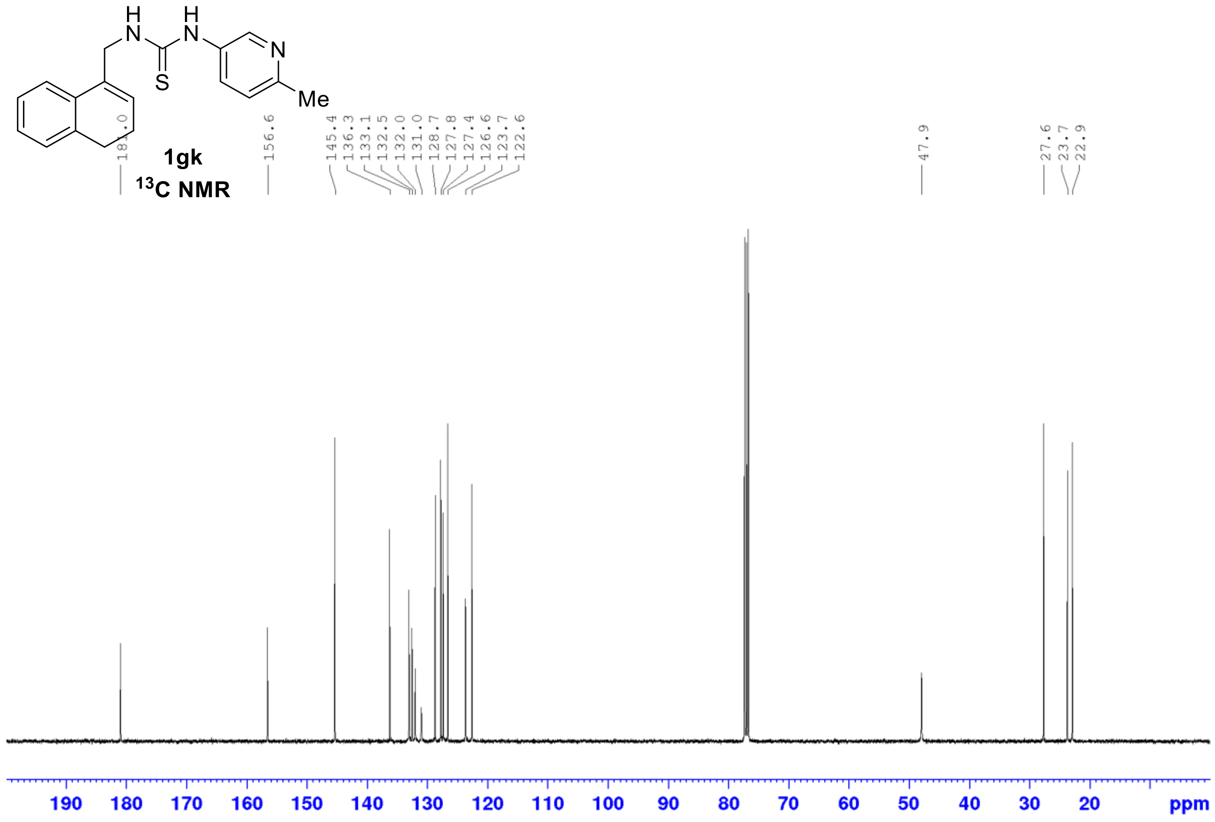

3-((3,4-Dihydronaphthalen-1-yl)methyl)-1-methyl-1-phenylthiourea (1gl)

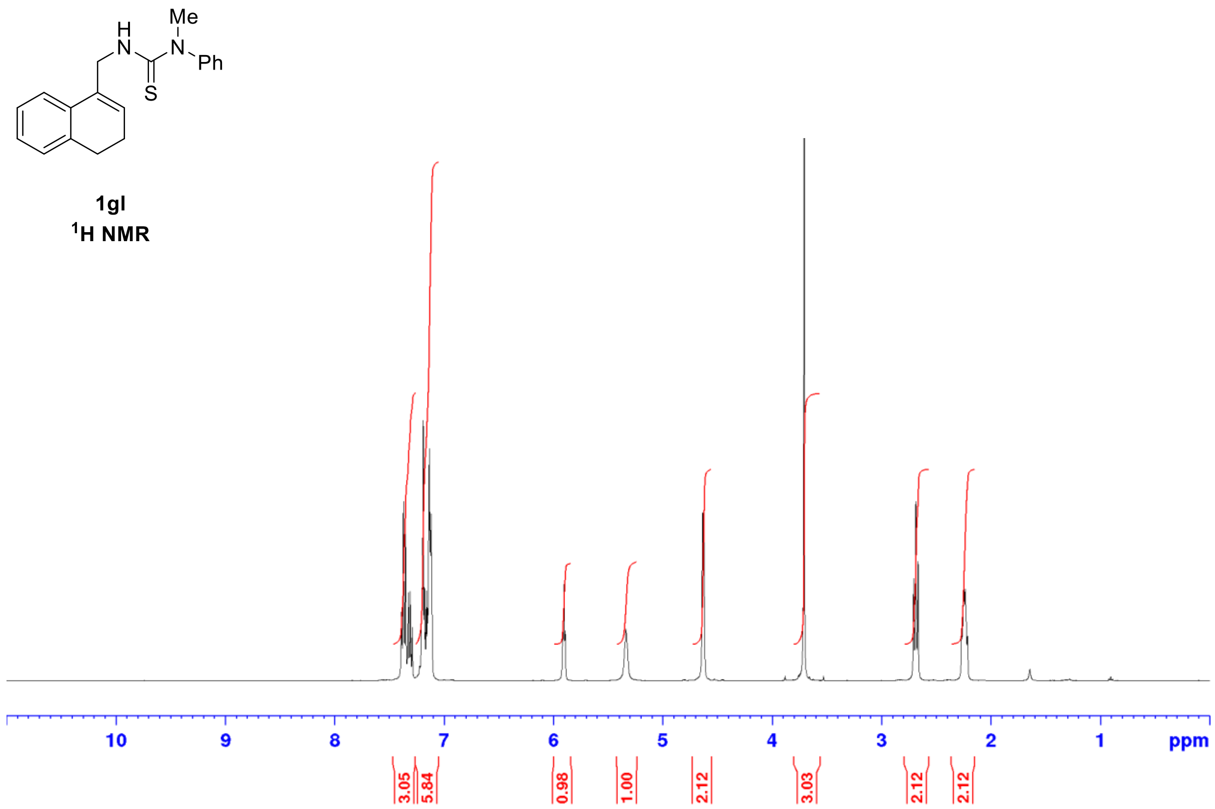

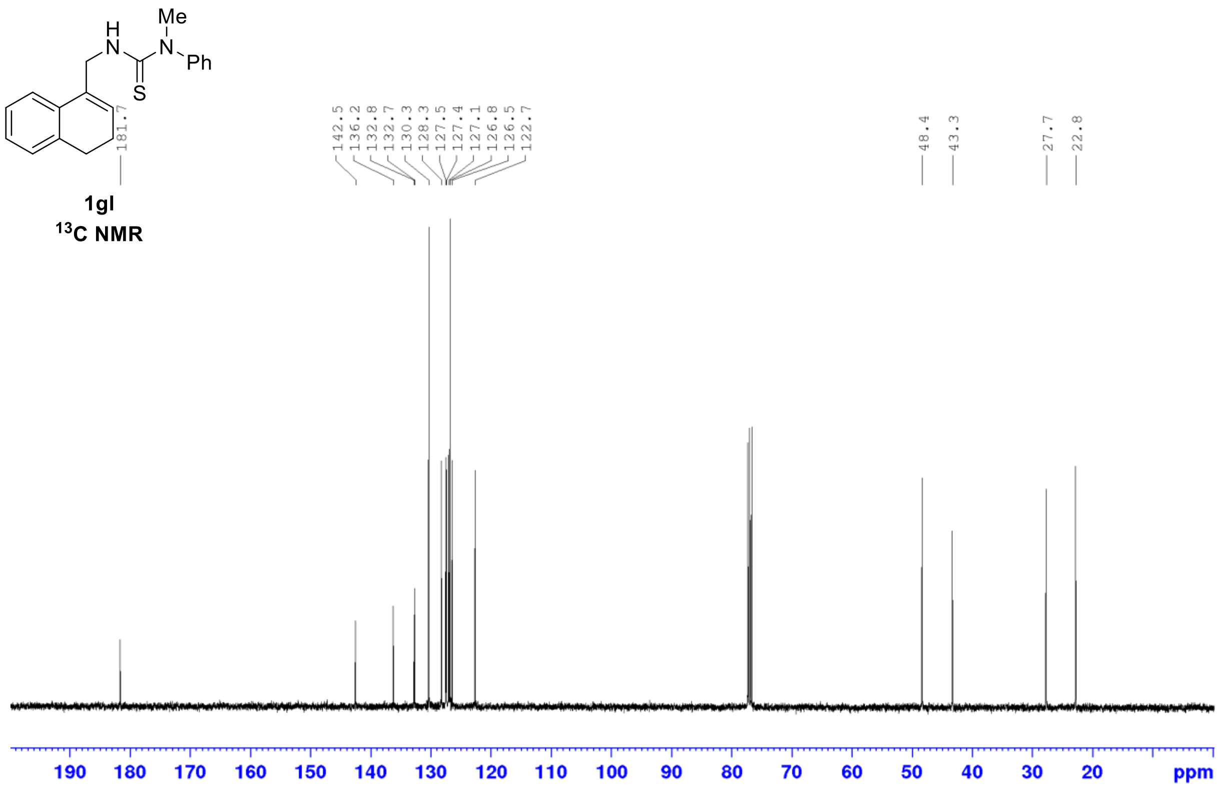

1-(1-(3,4-Dihydronaphthalen-1-yl)propyl)-3-phenylthiourea (1gn)

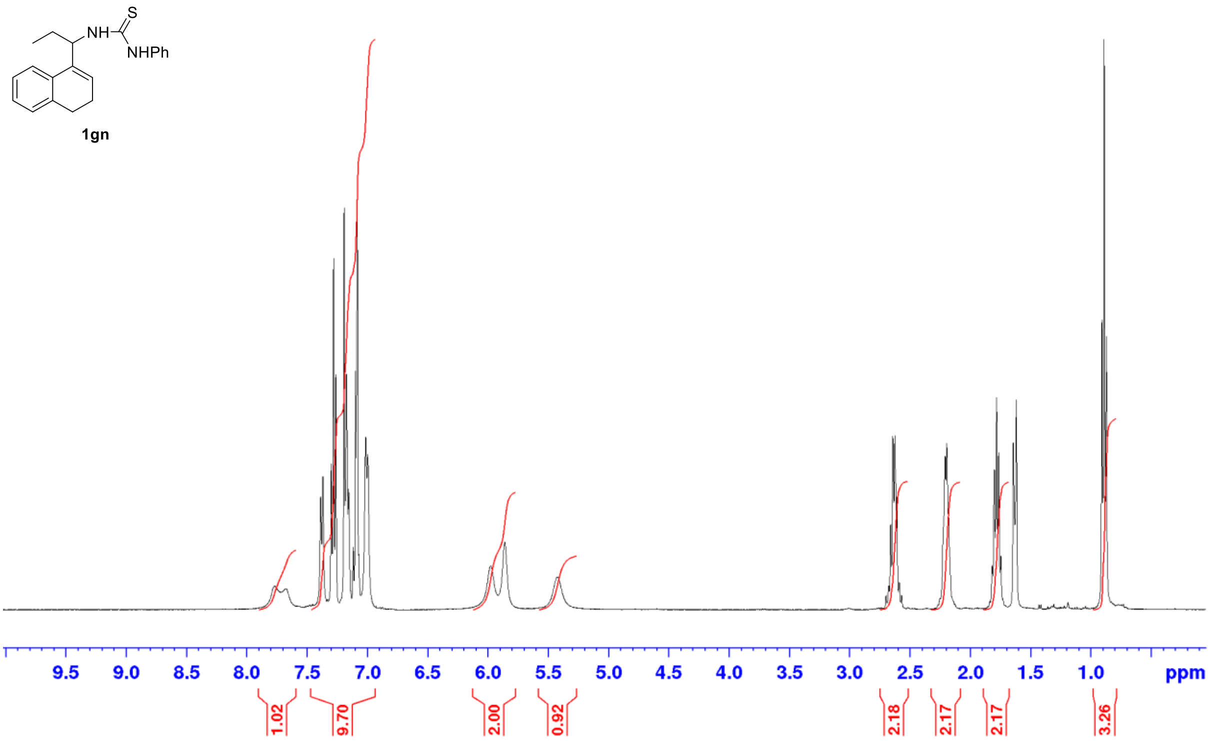

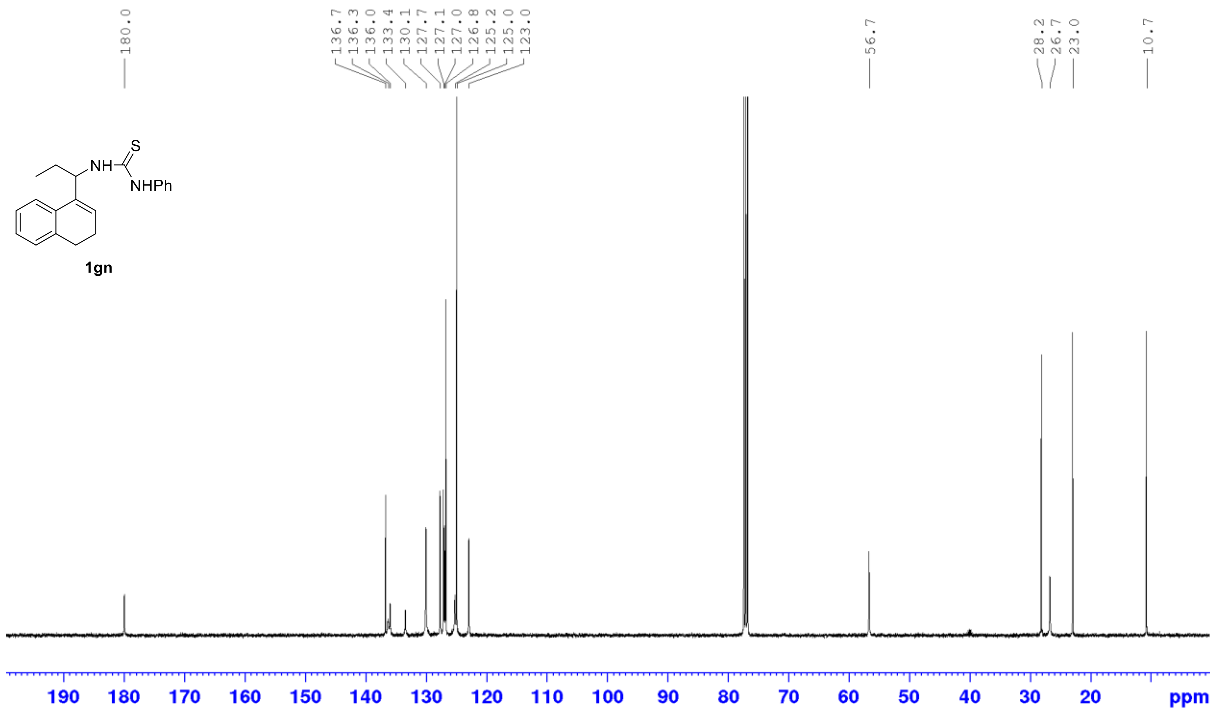

1-((2H-Chromen-4-yl)methyl)-3-phenylthiourea (1ha)

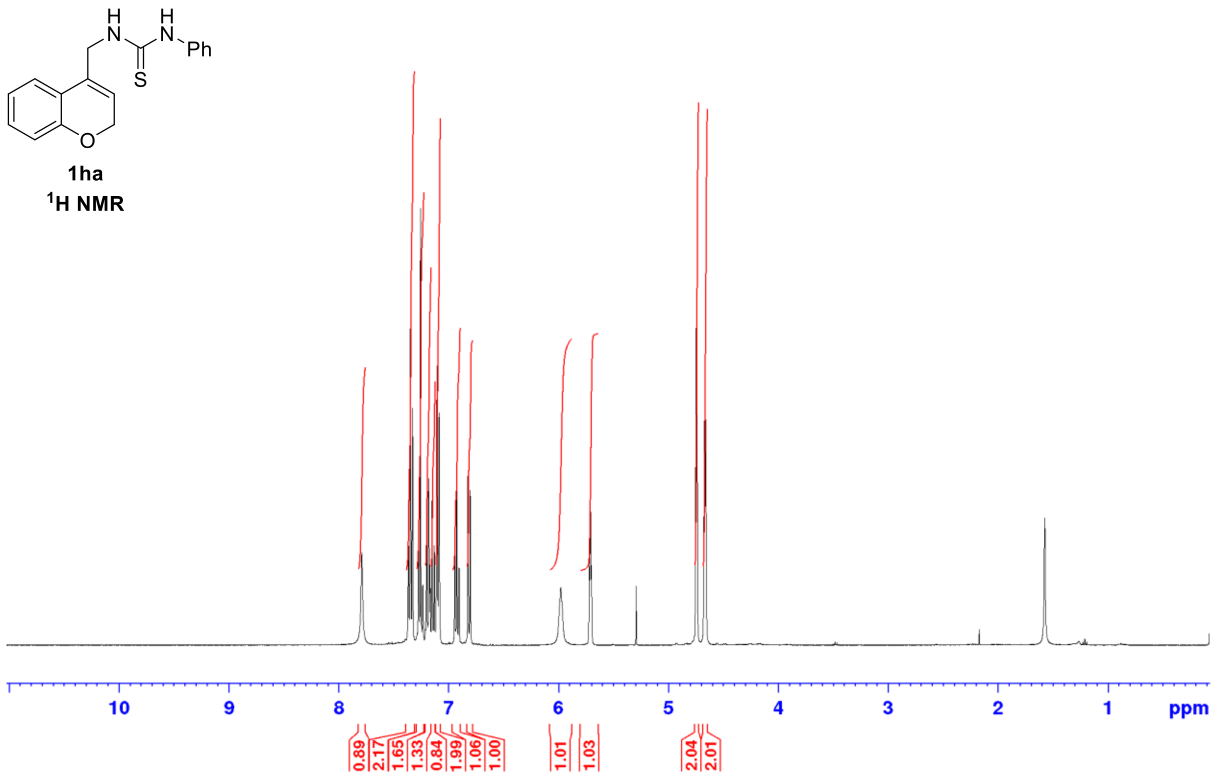

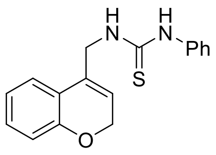

1ha

^{13}C NMR

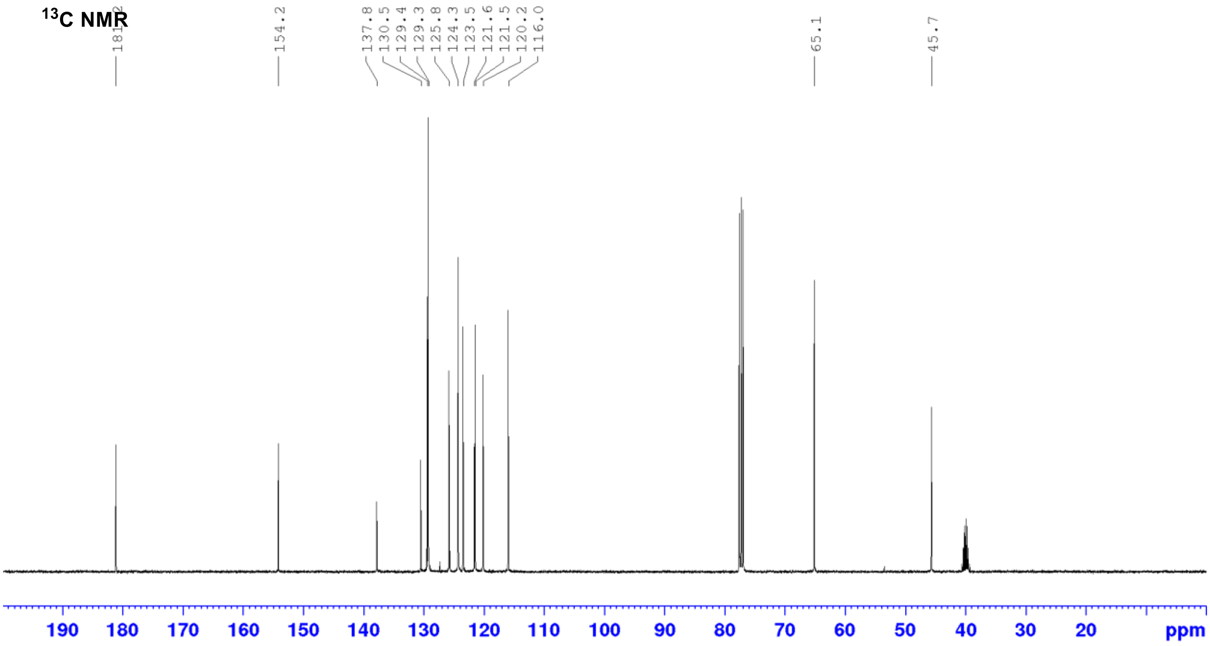

1-((2H-Thiochromen-4-yl)methyl)-3-phenylthiourea (1ia)

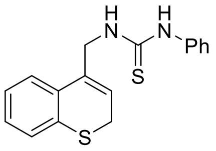

1ia

^{1}H NMR

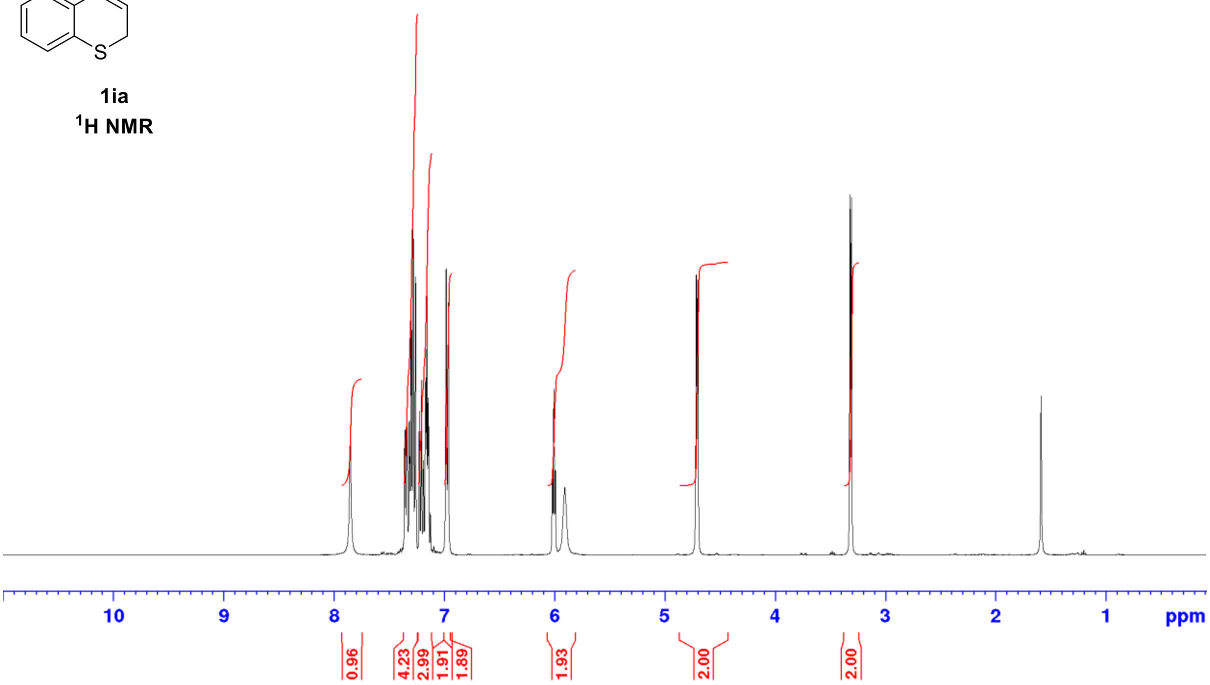

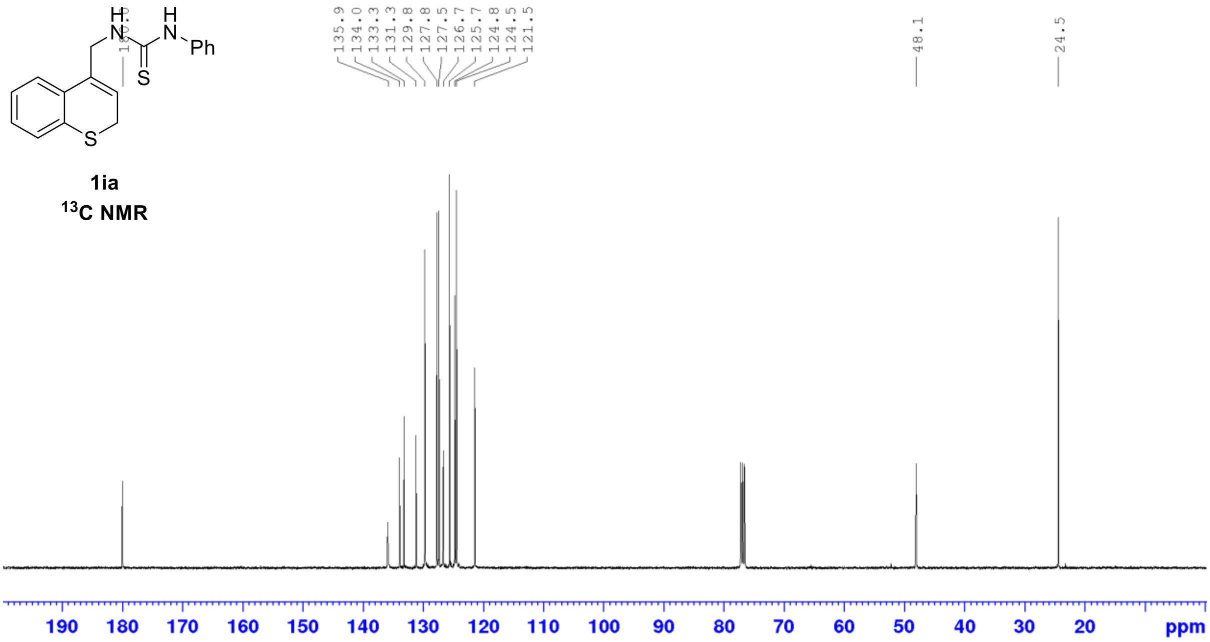

1-((1H-Inden-3-yl)methyl)-3-phenylthiourea (1ja)

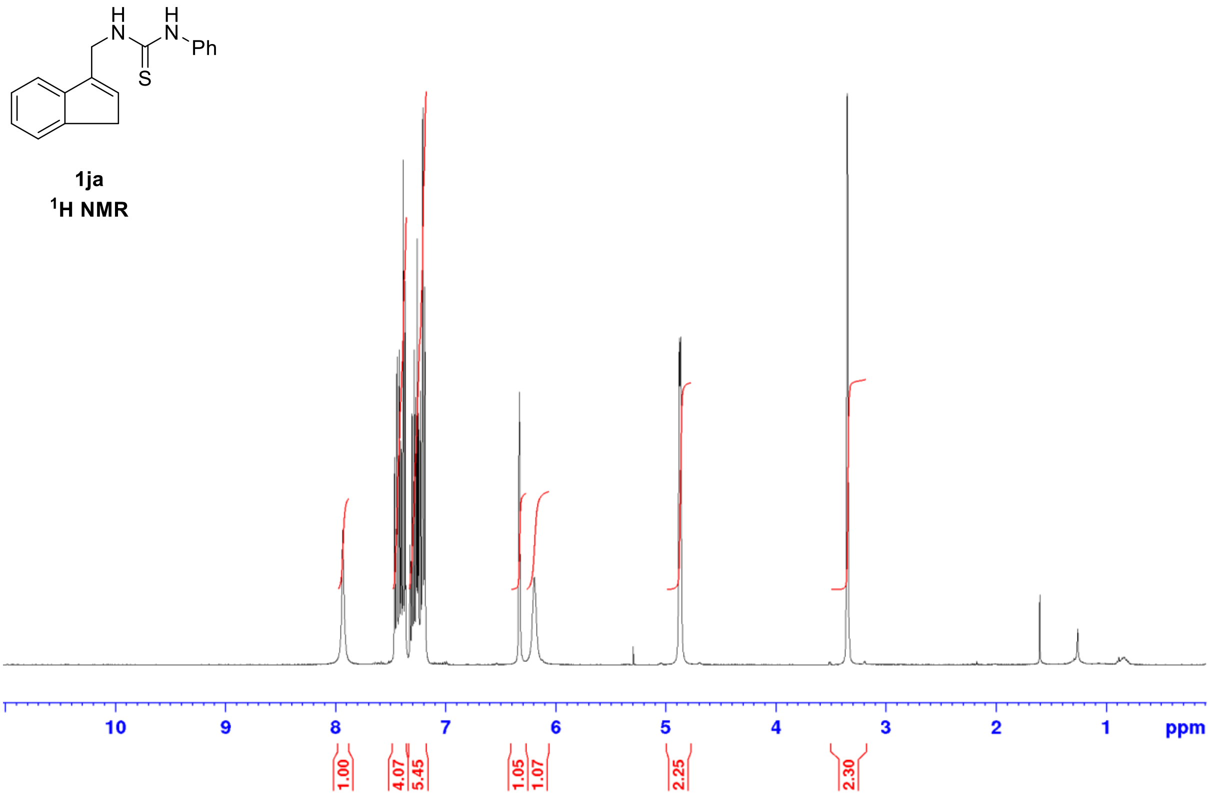

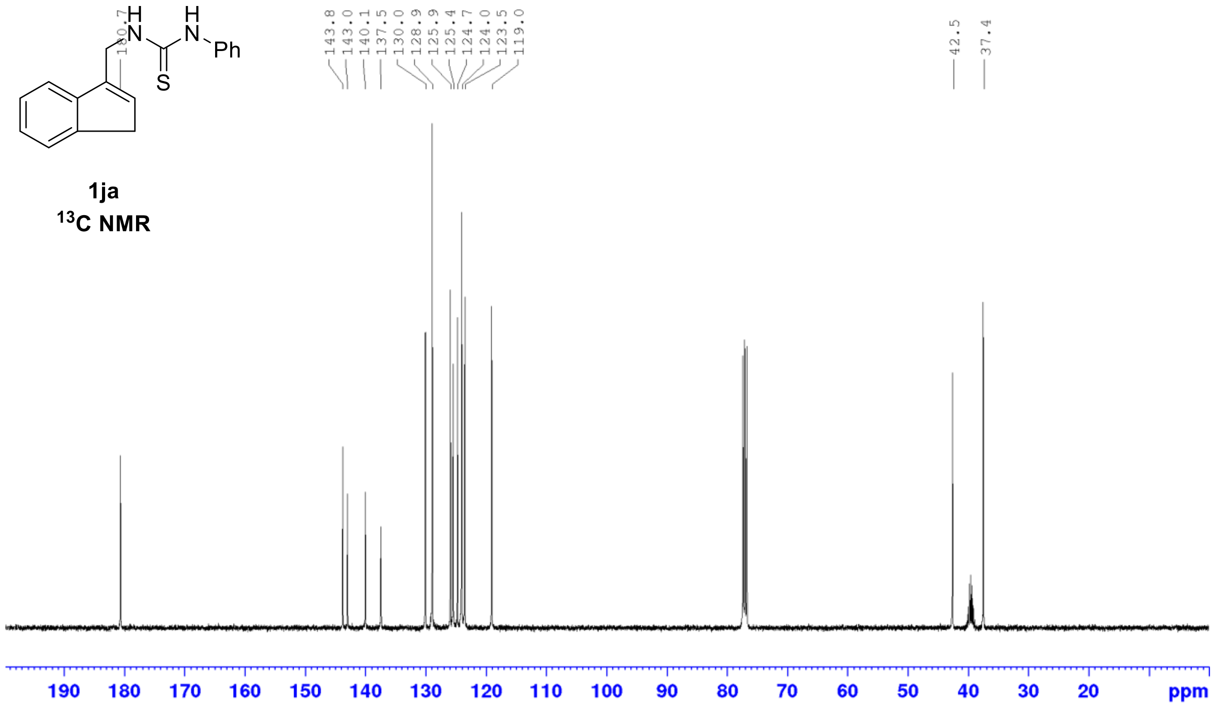

1-(2-(3,4-Dihydronaphthalen-1-yl)ethyl)-3-phenylthiourea (1ka)

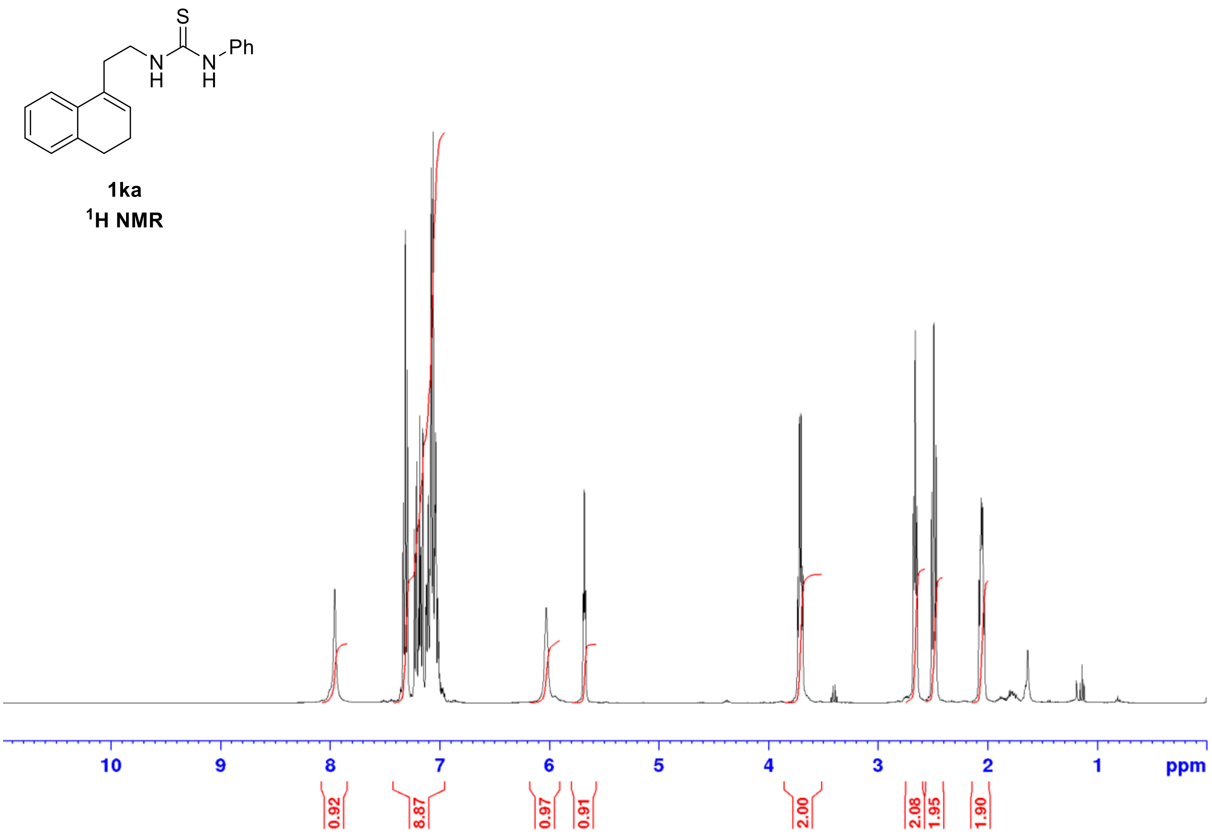

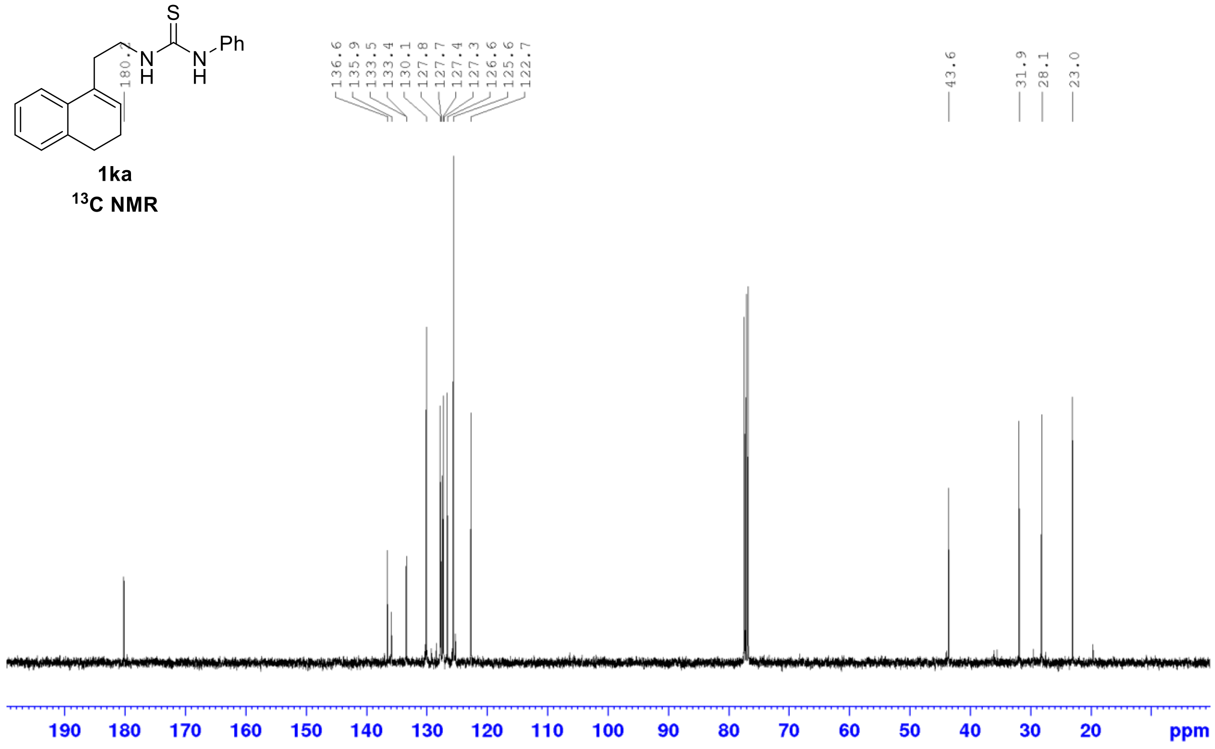

1-Phenyl-3-(2-(prop-1-en-2-yl)phenyl)thiourea (1la)

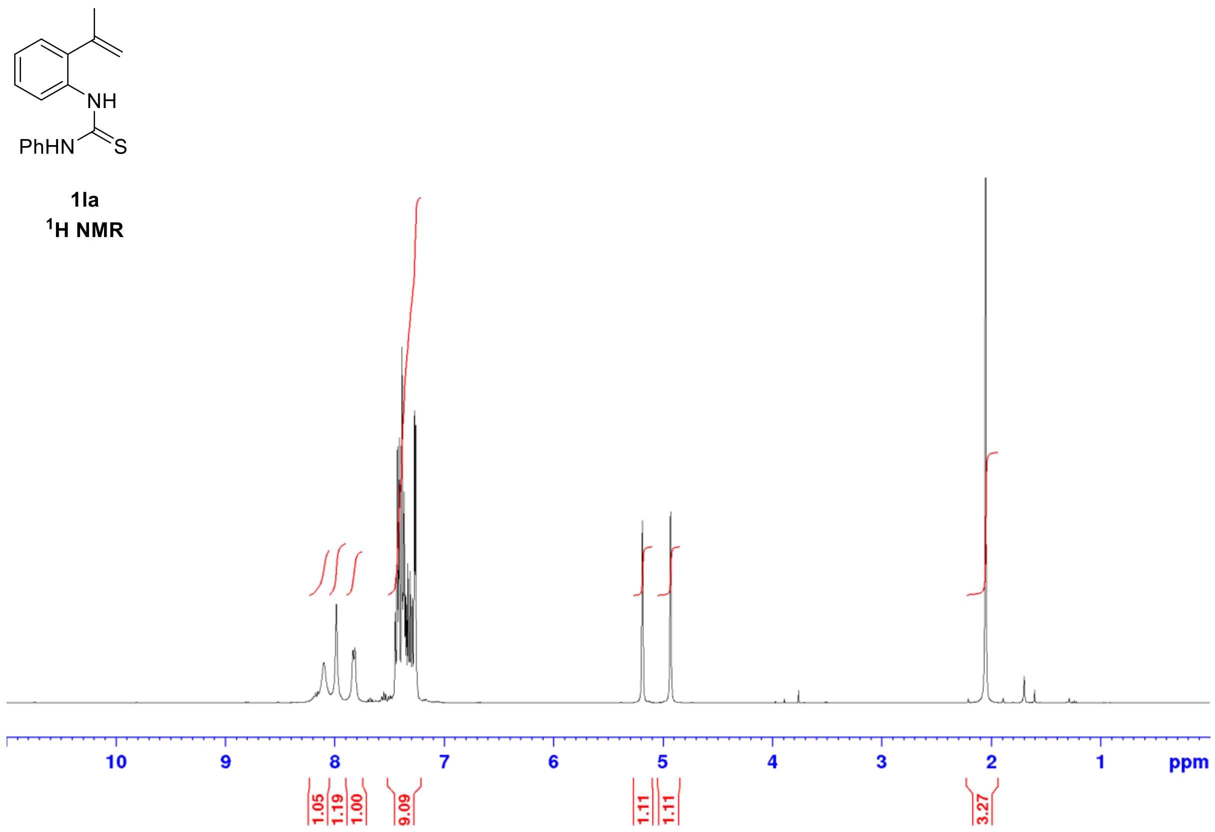

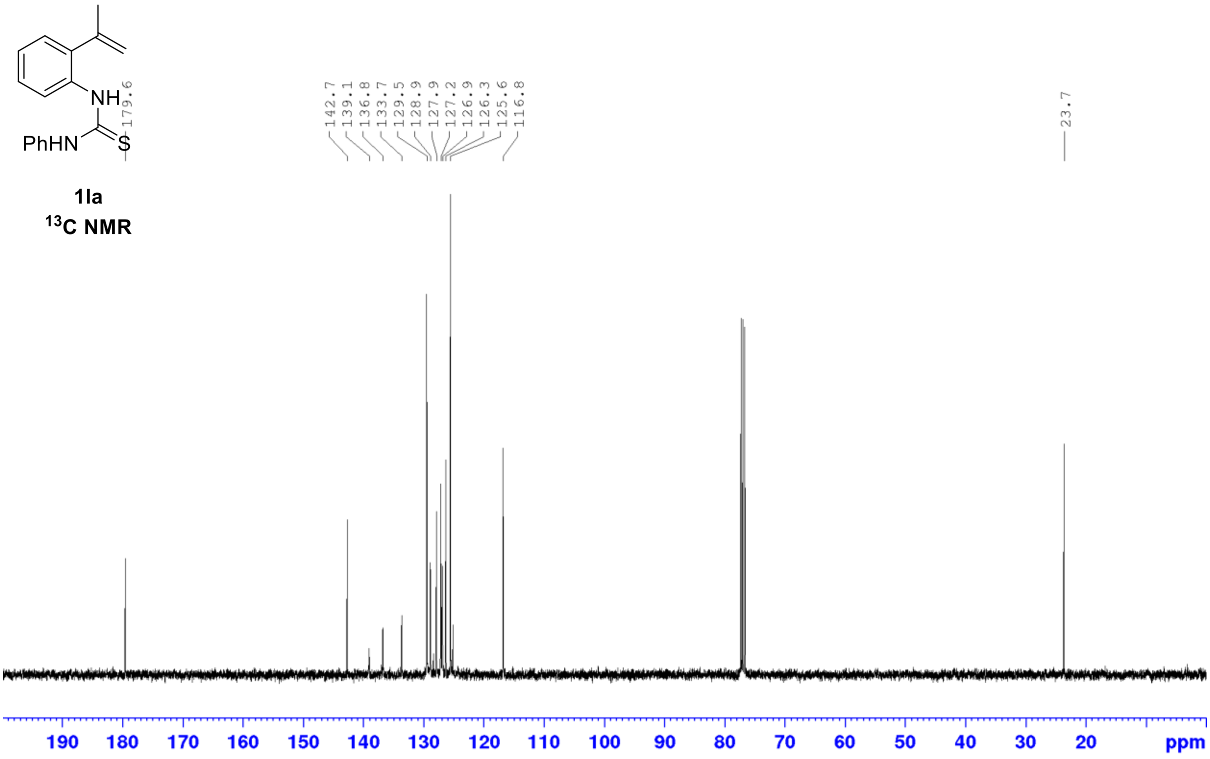

1-(5-fluoro-2-(prop-1-en-2-yl)phenyl)-3-phenylthiourea (1ma)

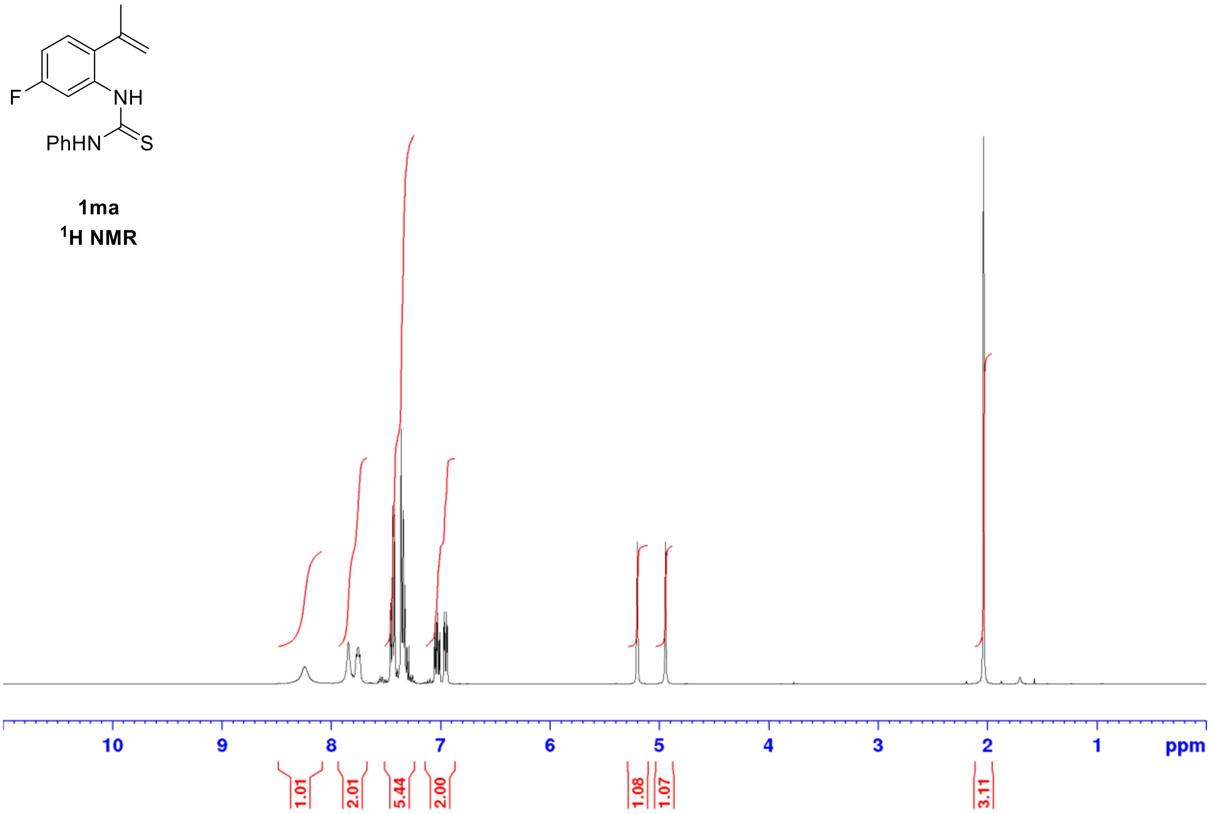

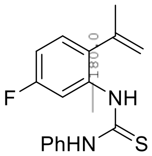

1ma
^{13}C NMR

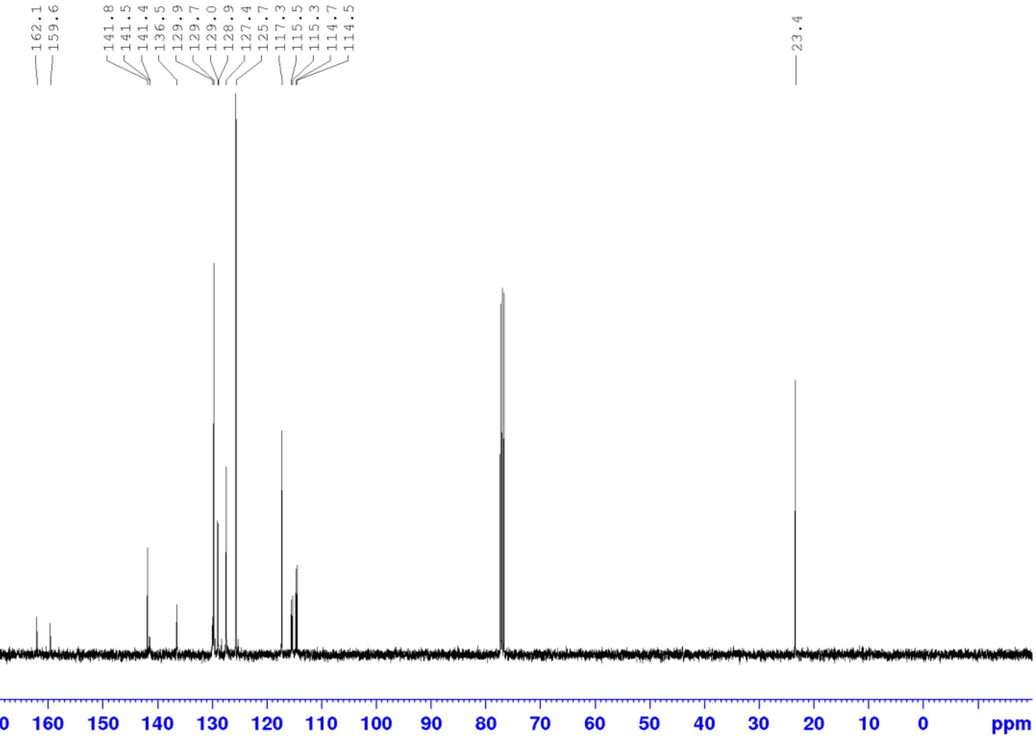

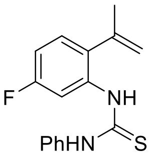

1ma
^{19}F NMR

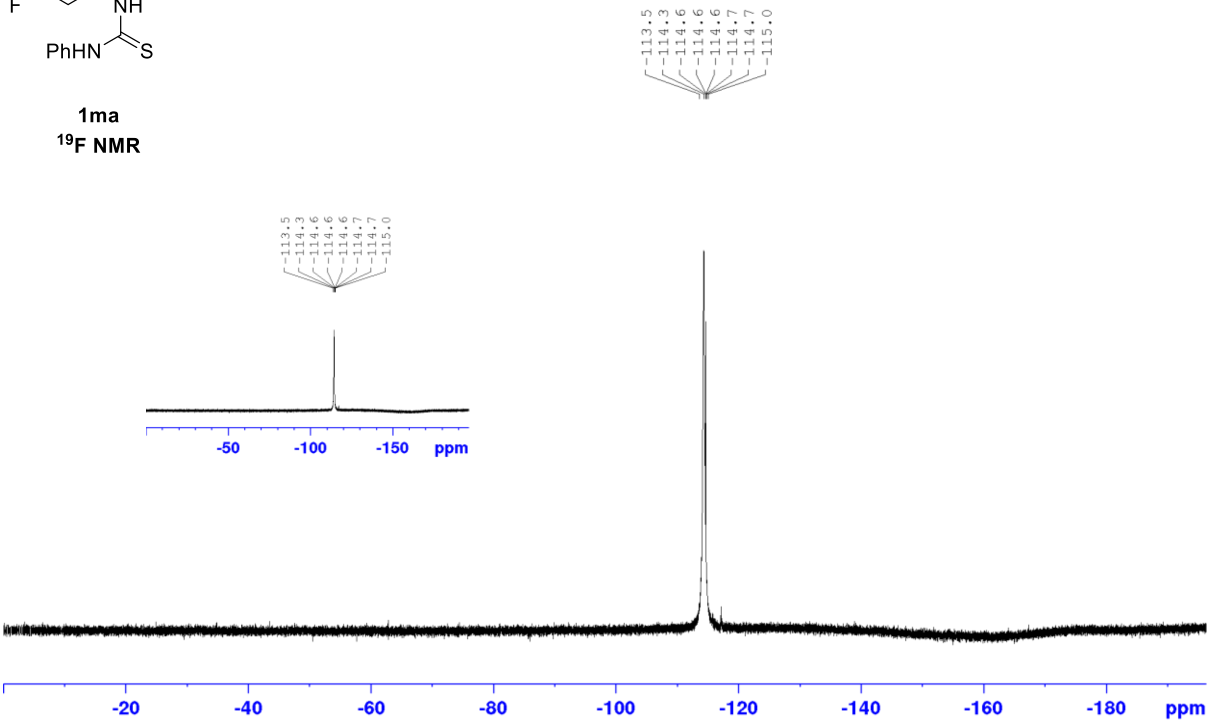

(*Z*)-1-Phenyl-3-m(3-phenylallyl)thiourea (*Z*-1na)

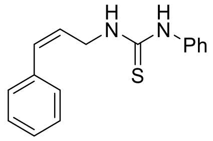

(*Z*)-1na
^{1}H NMR

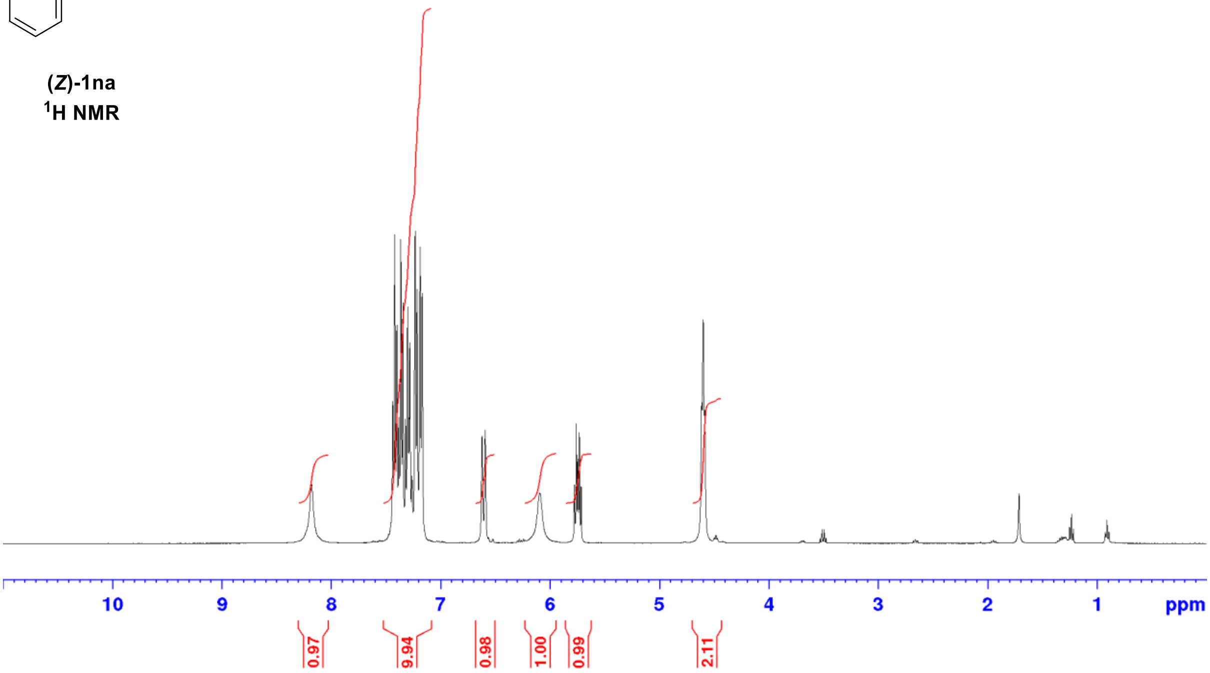

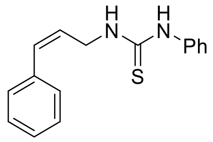

(*Z*)-1na
^{13}C NMR

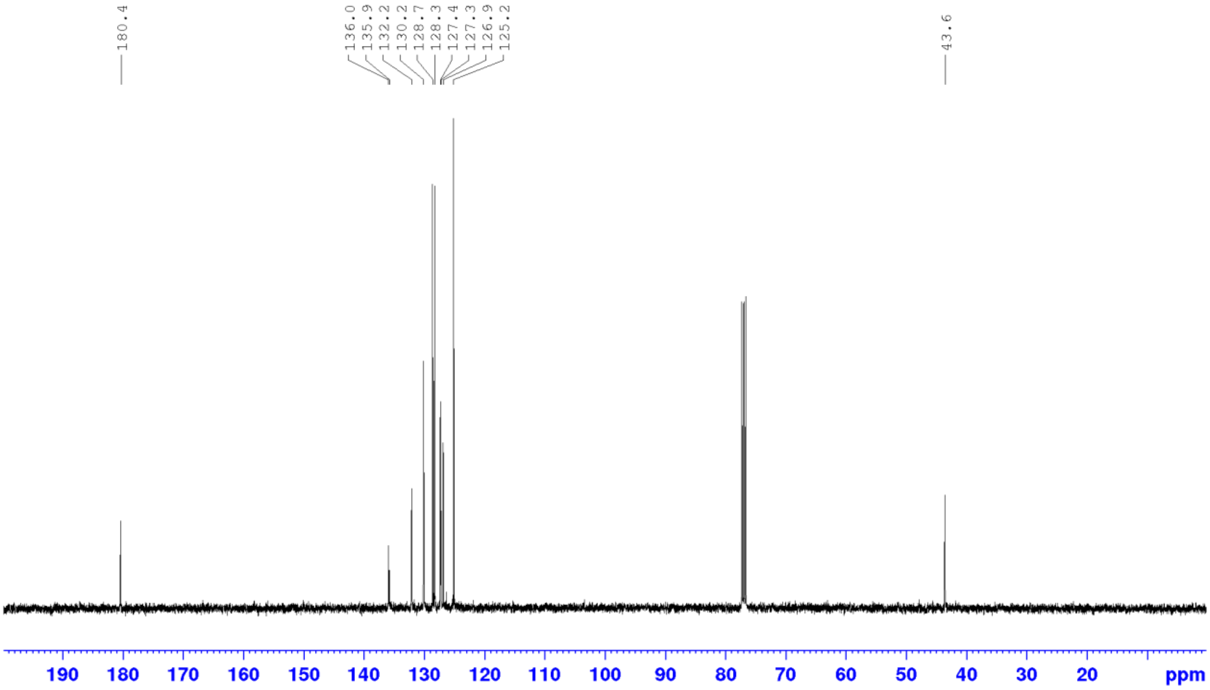

1-Cinnamyl-3-phenylthiourea (*E*-1na)

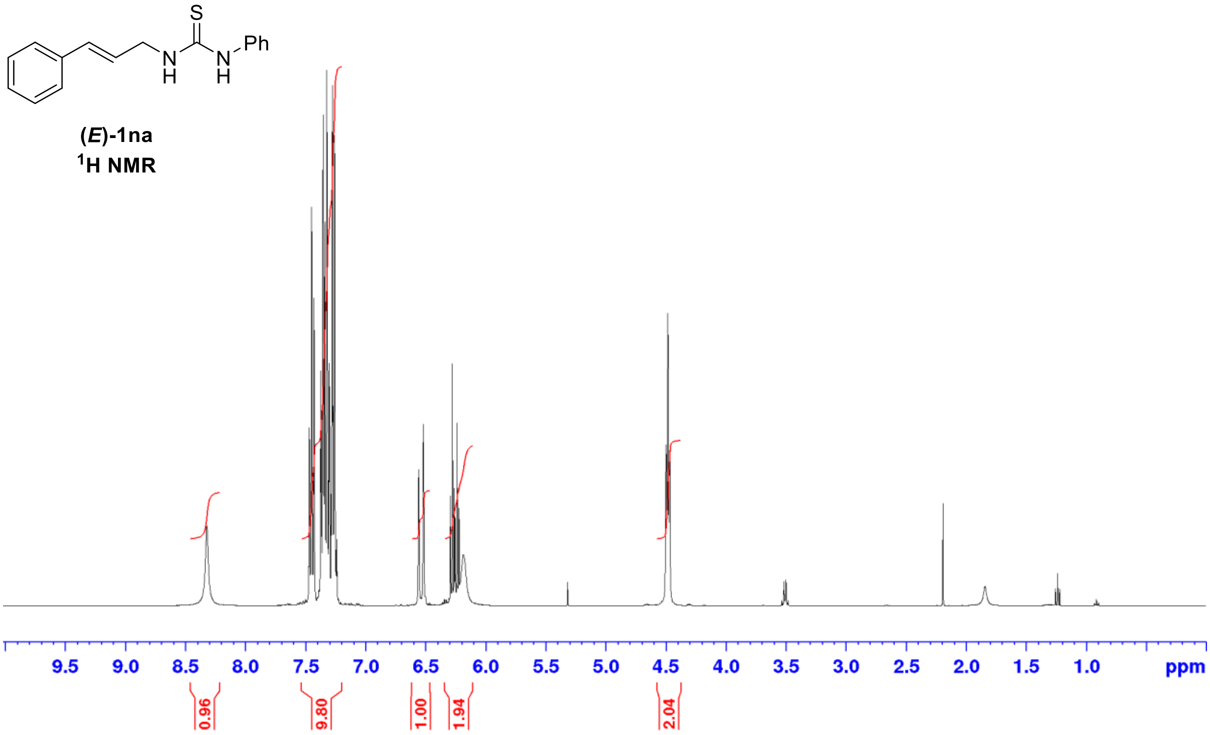

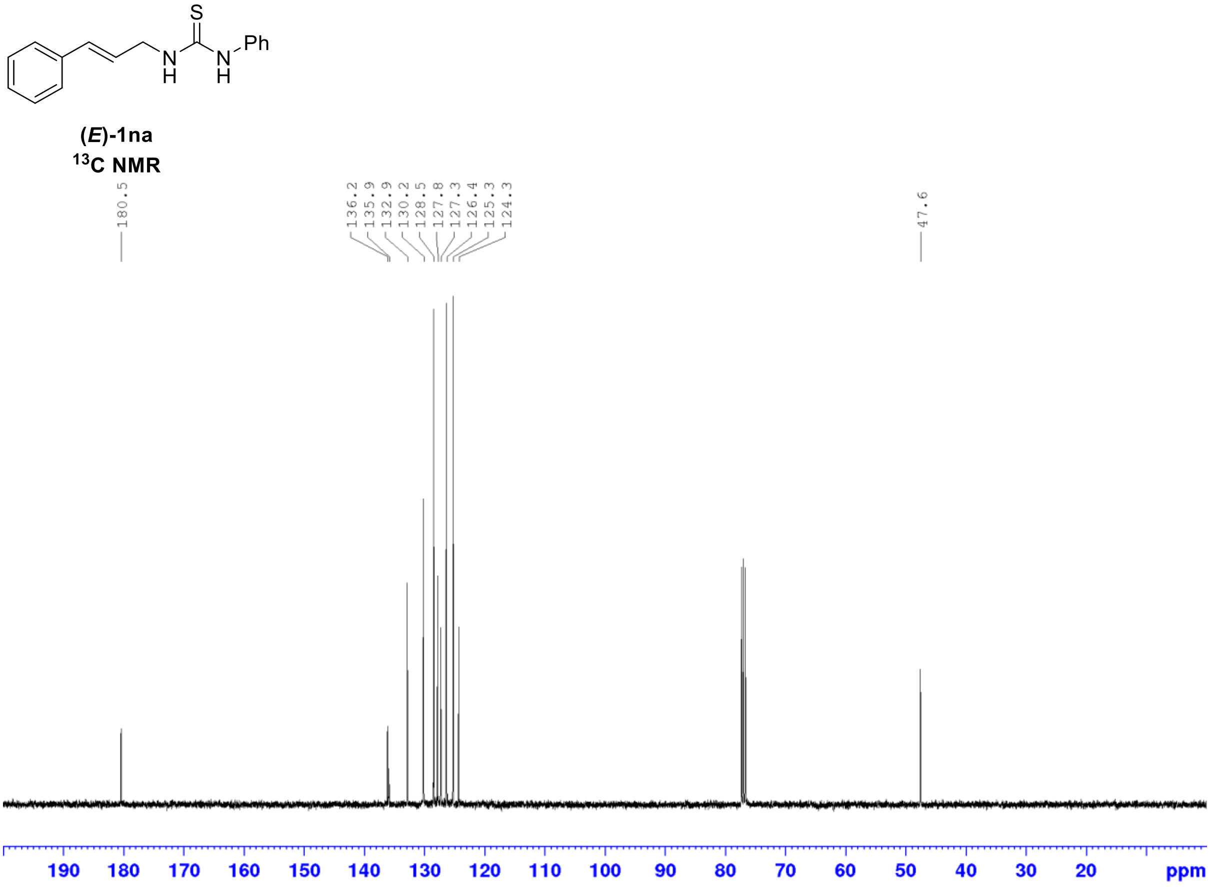

(*E*)-1-(3-(4-Methoxyphenyl)allyl)-3-phenylthiourea (1oa)

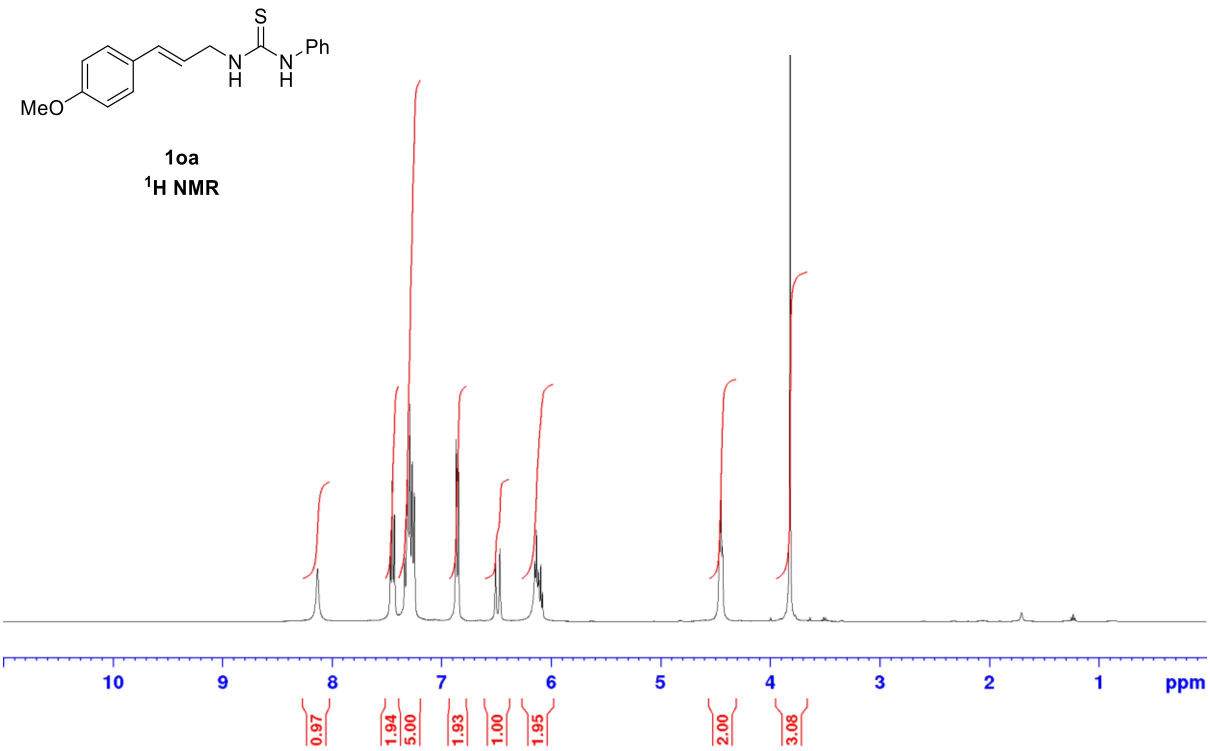

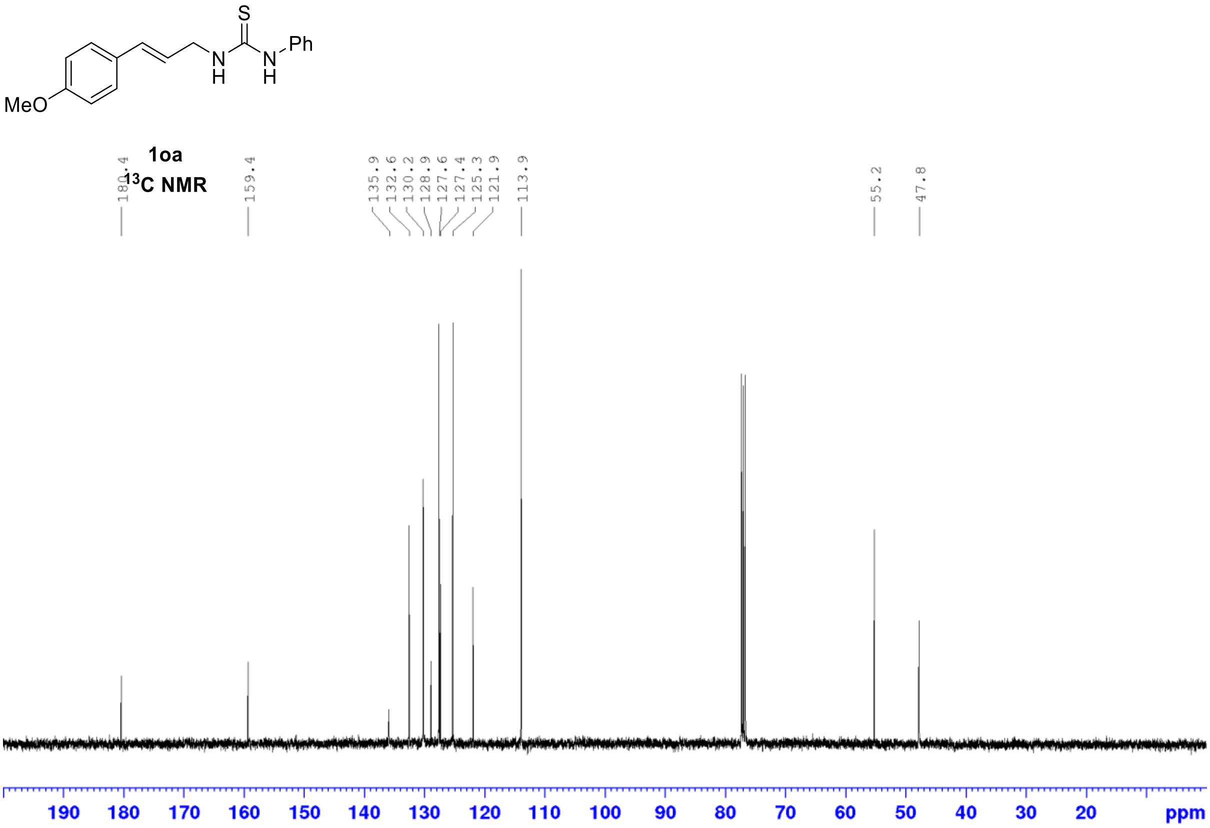

(*E*)-1-Phenyl-3-(3-(*p*-tolyl)allyl)thiourea (1pa)

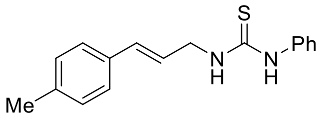

1pa

^{1}H NMR

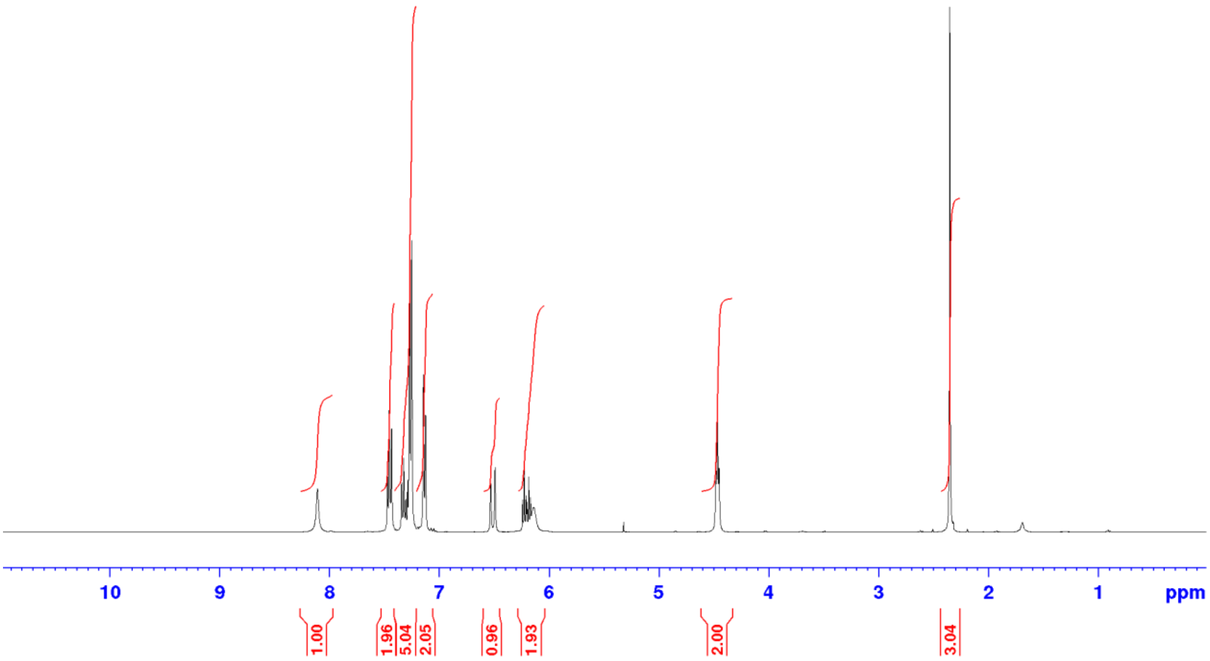

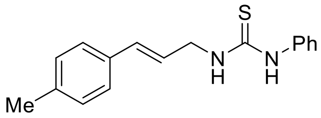

1pa

^{13}C NMR

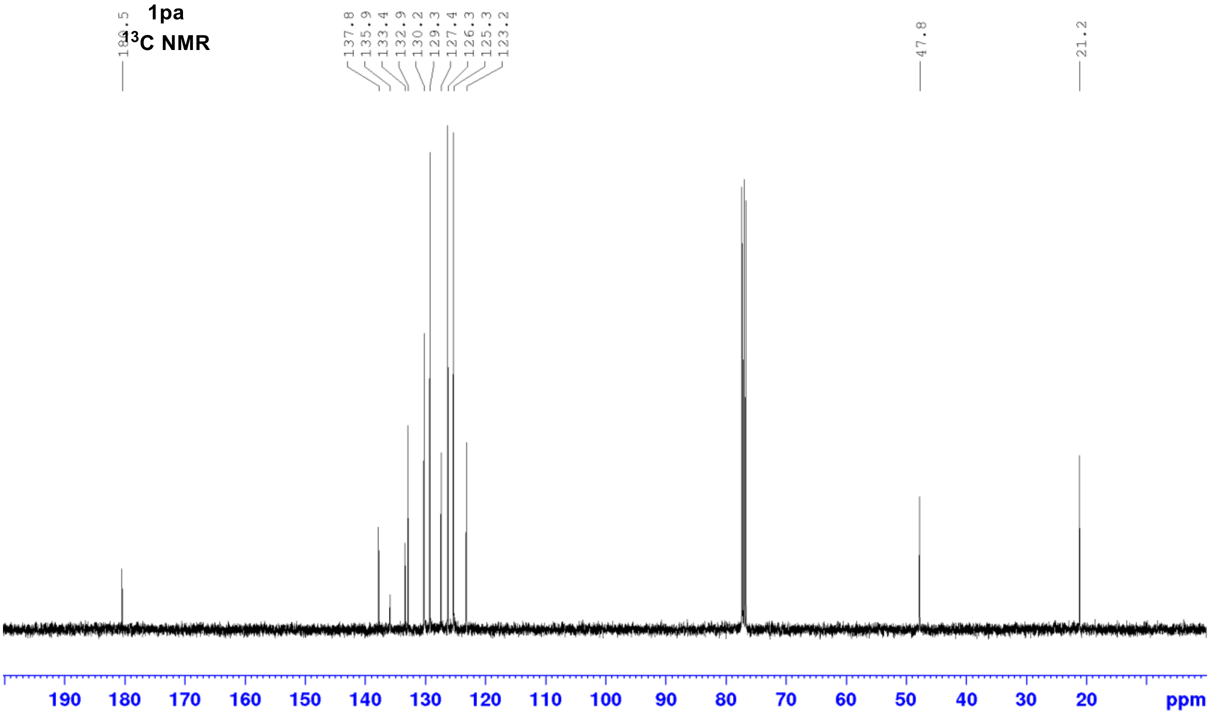

(*E*)-1-(3-(4-Chlorophenyl)allyl)-3-phenylthiourea (1qa)

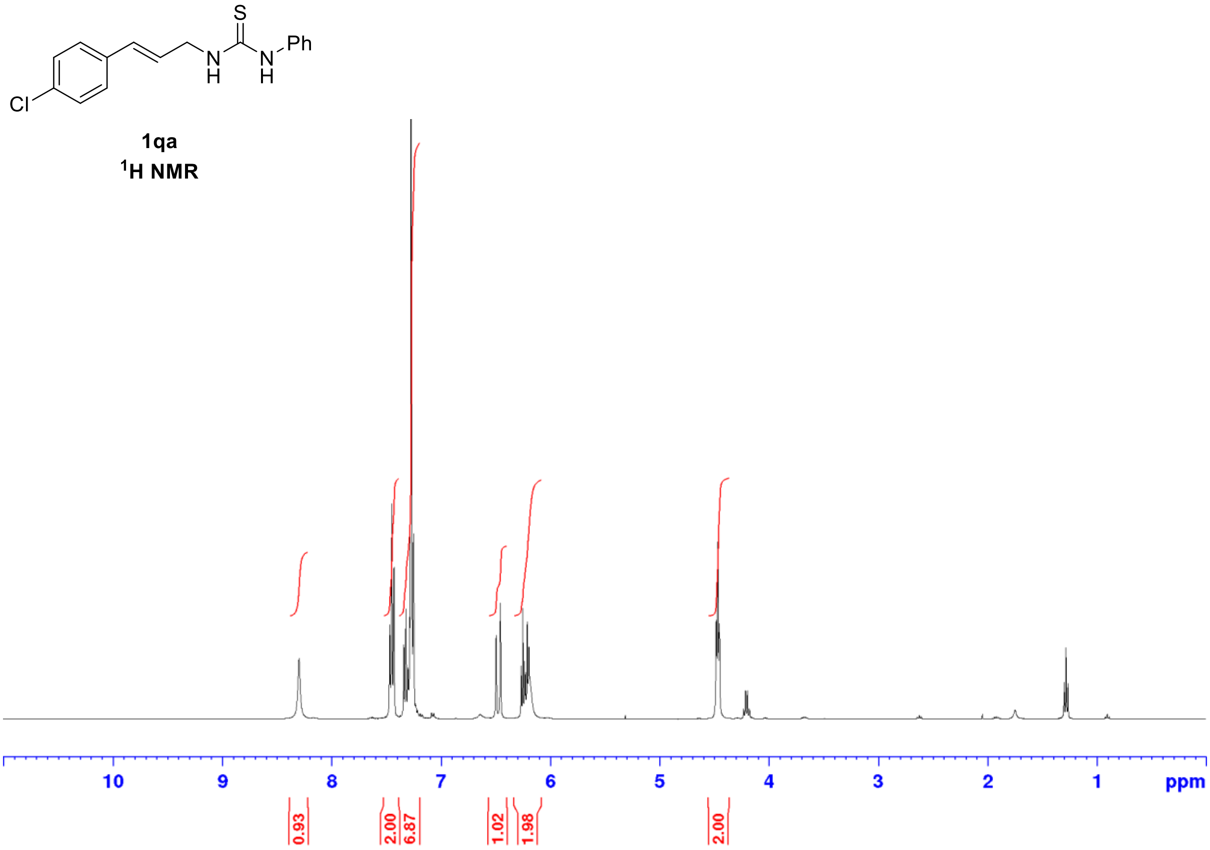

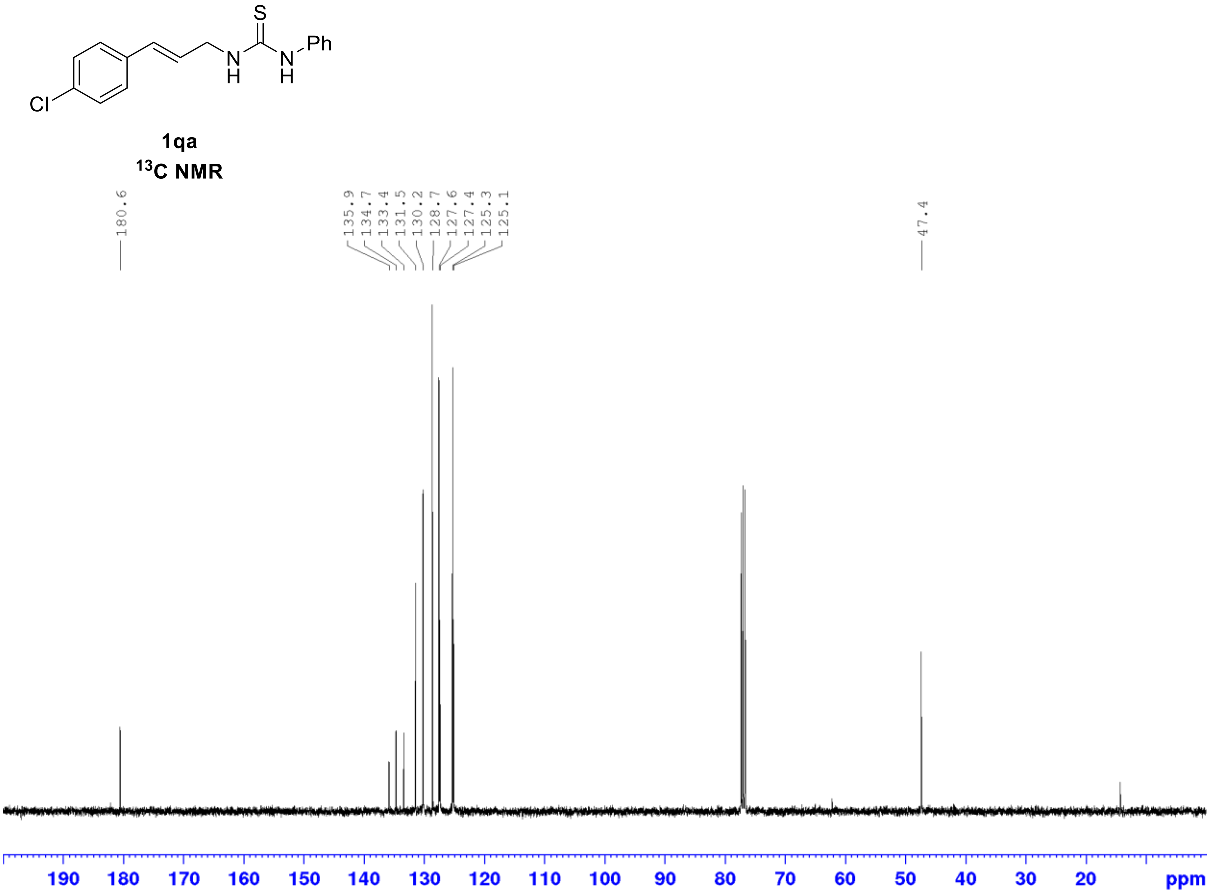

1-Cinnamyl-3-(*p*-tolyl)thiourea (1nb)

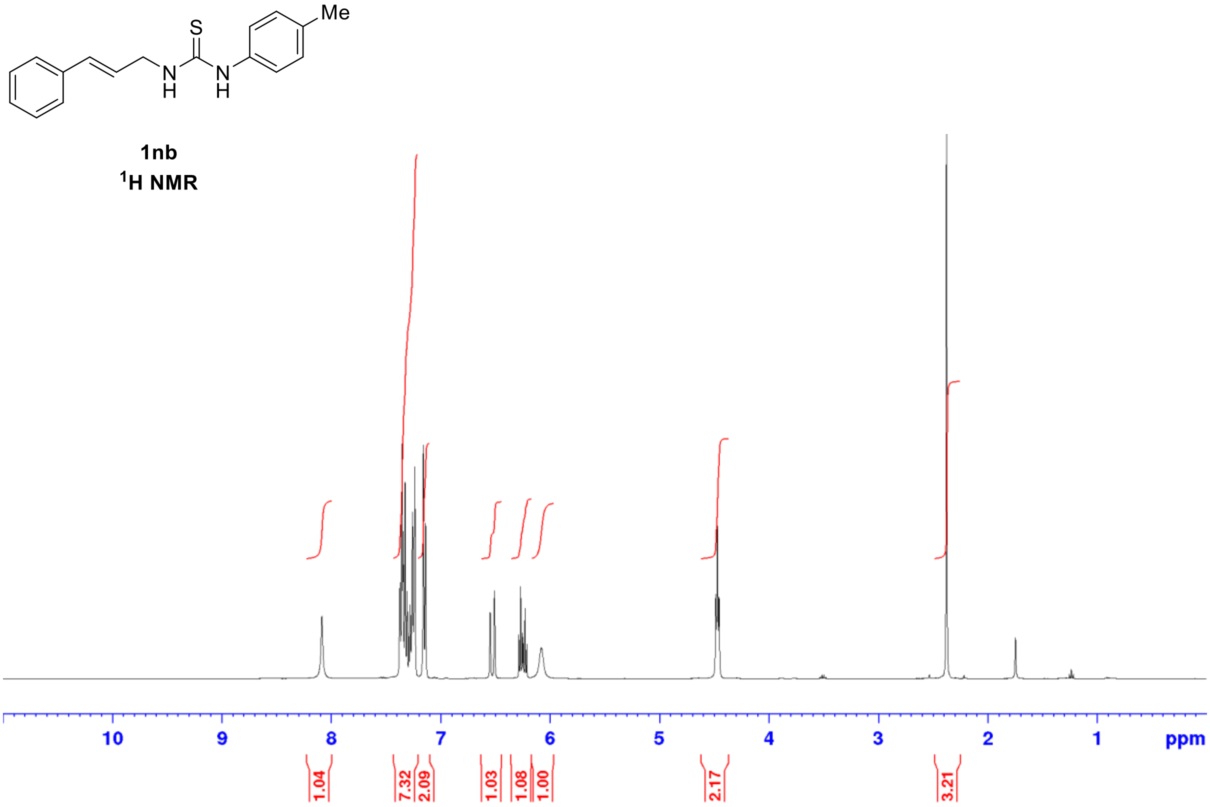

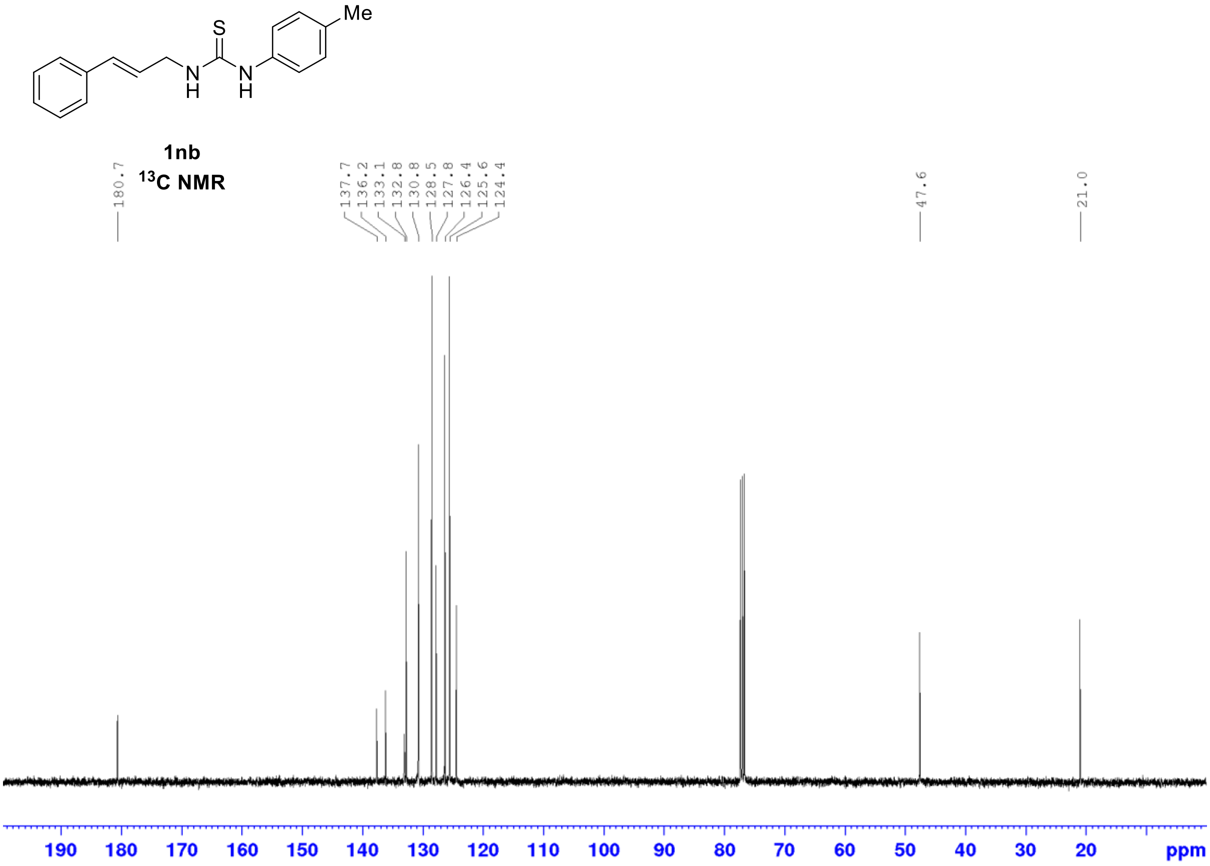

1-Cinnamyl-3-(4-methoxyphenyl)thiourea (1nc)

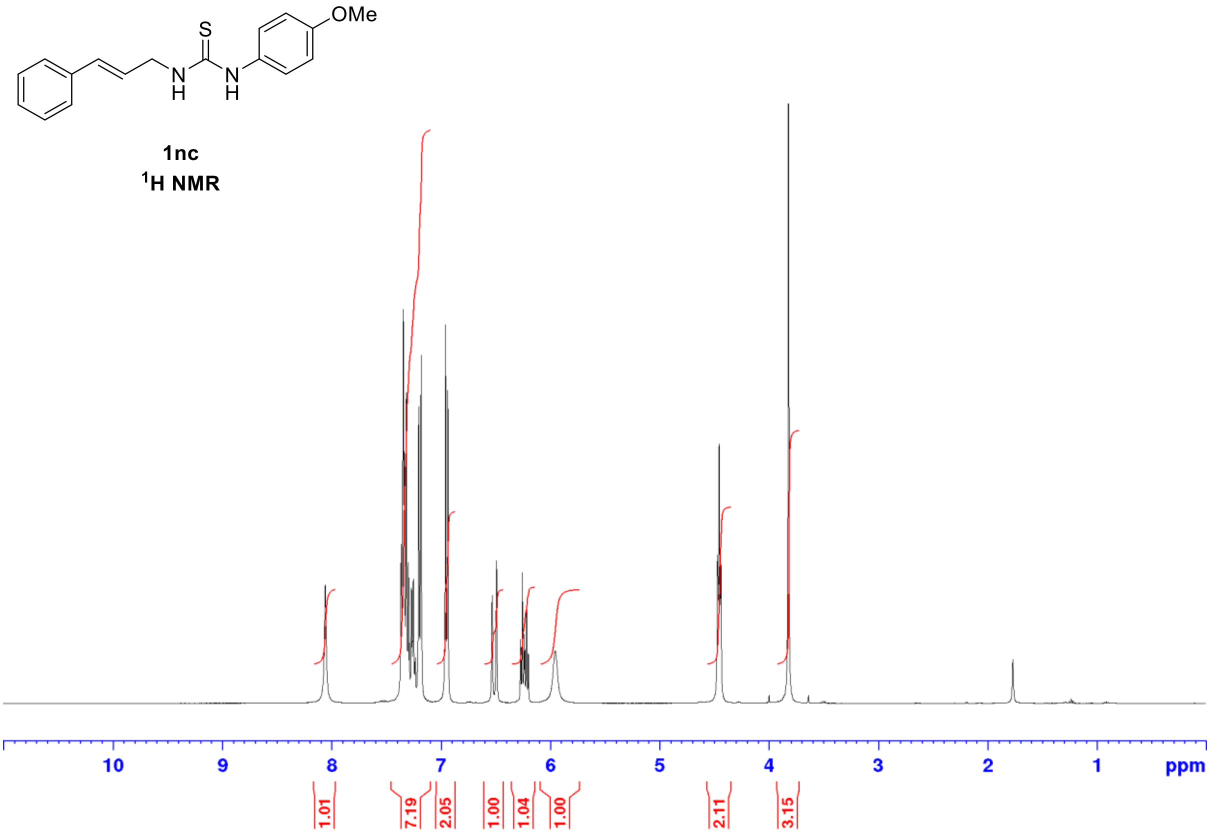

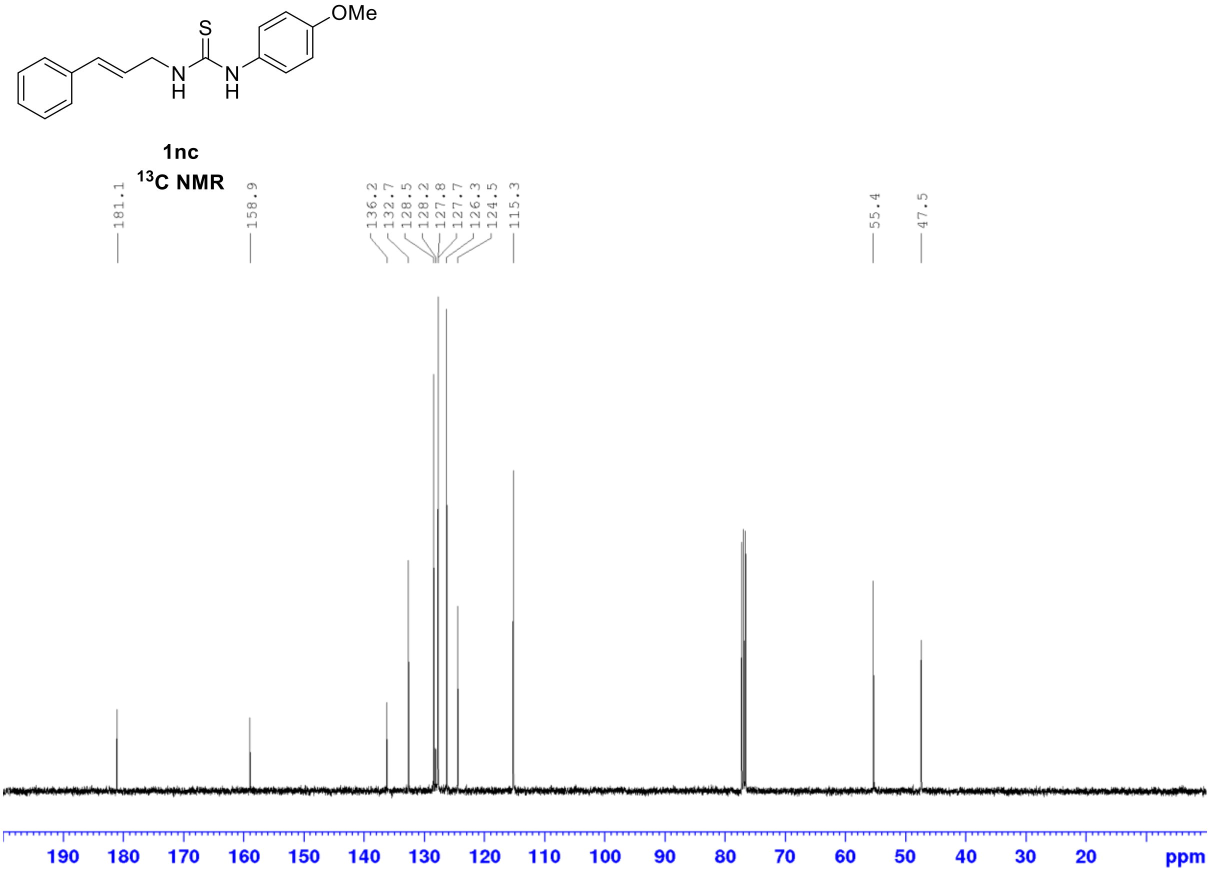

1-Cinnamyl-3-(4-(trifluoromethyl)phenyl)thiourea (1nd)

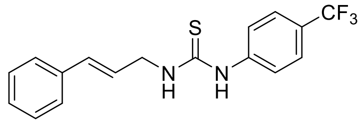

1nd

^{1}H NMR

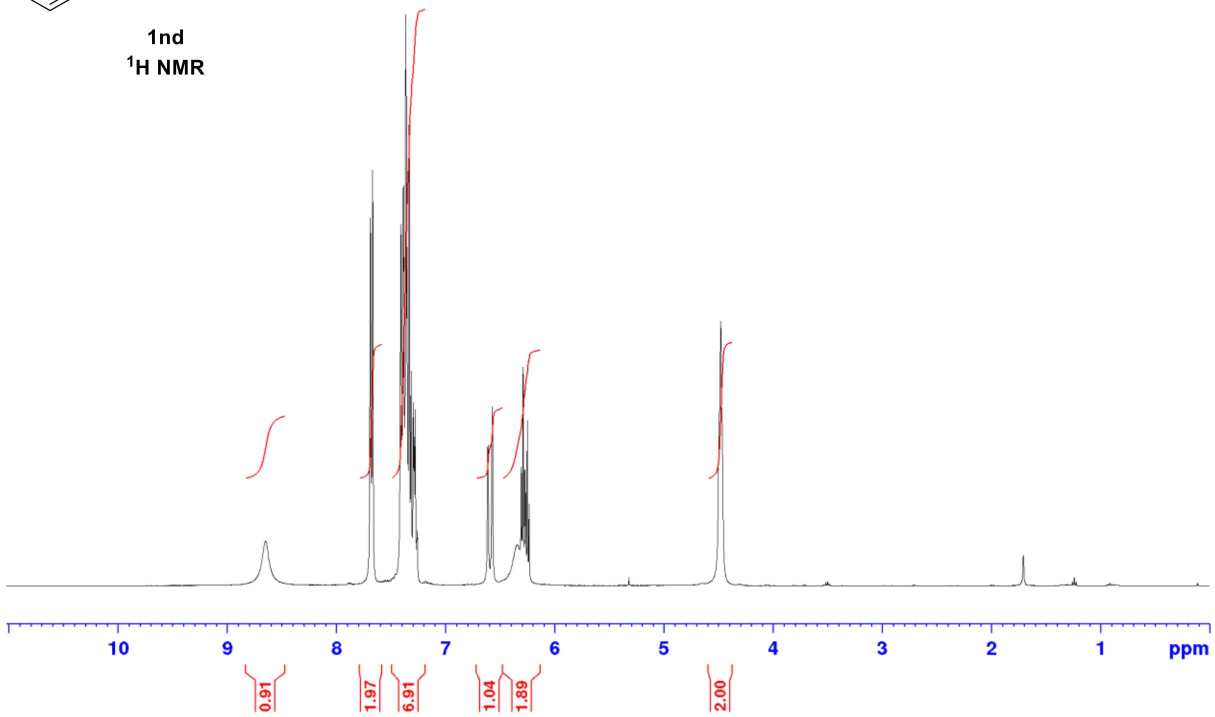

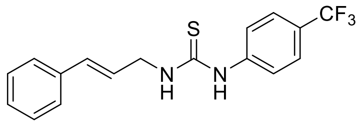

1nd

^{13}C NMR

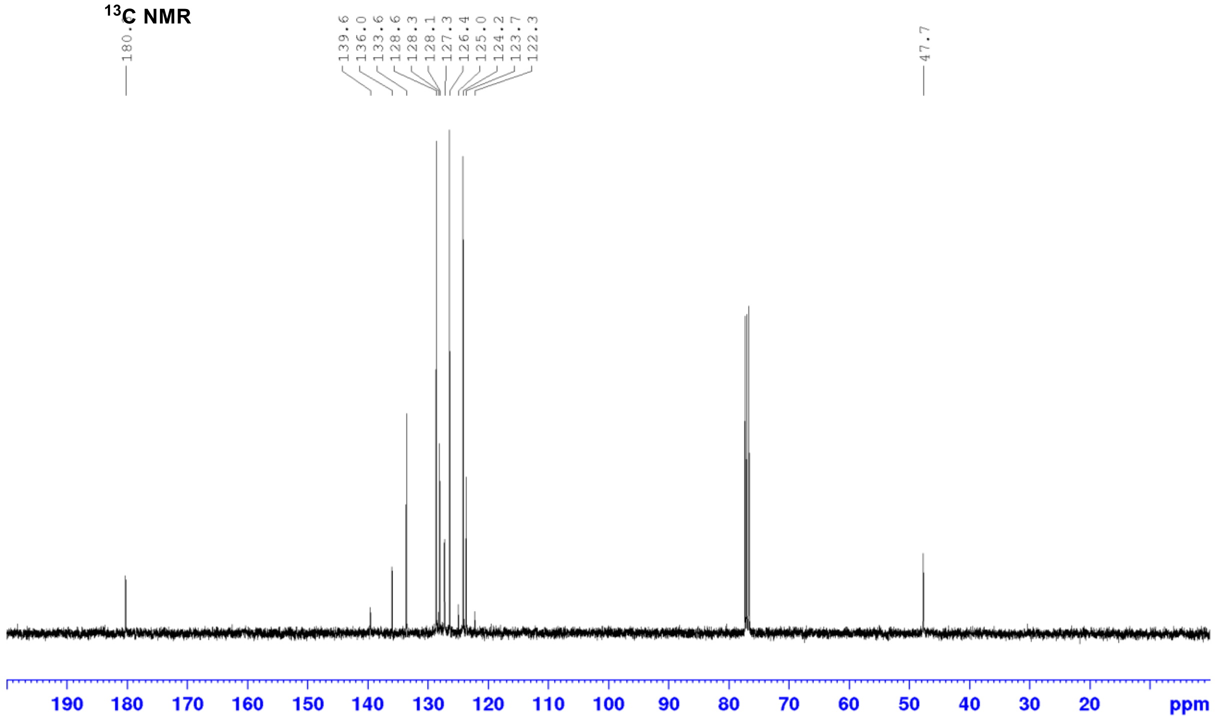

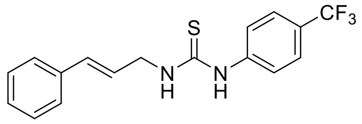

1nd
^{19}F NMR

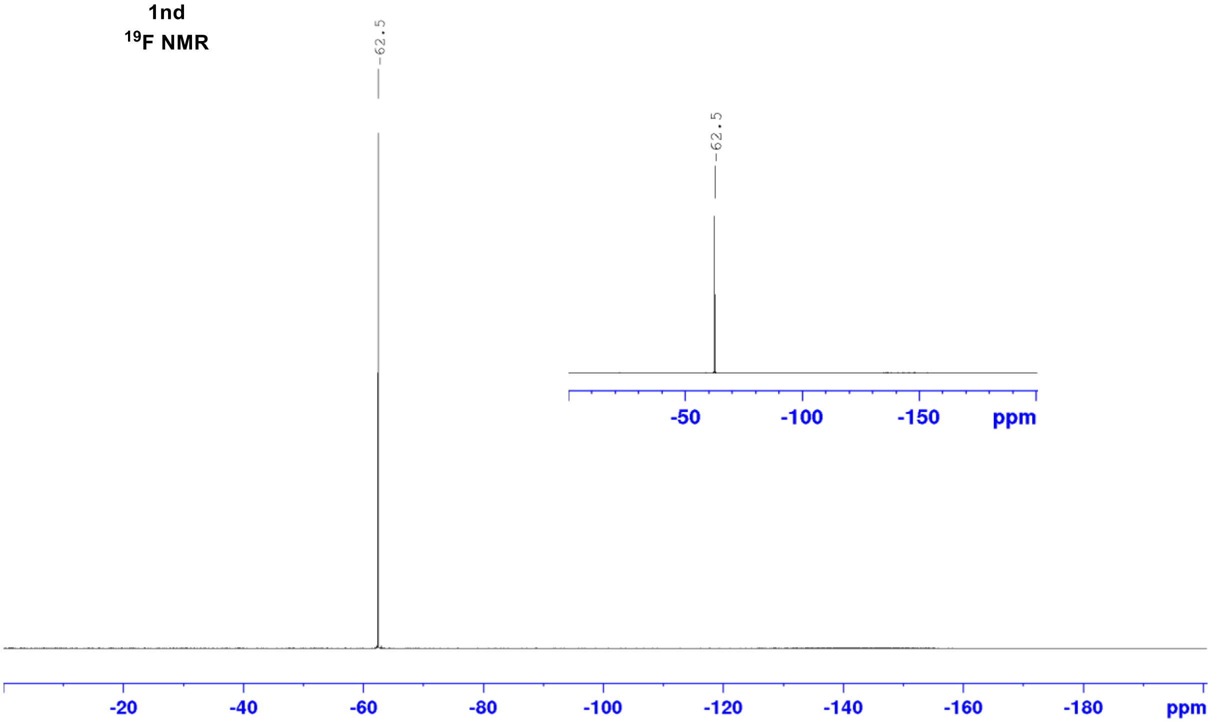

1-Cinnamyl-3-(4-fluorophenyl)thiourea (1ne)

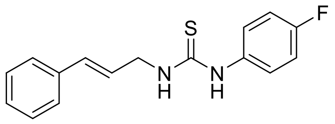

1ne
^{1}H NMR

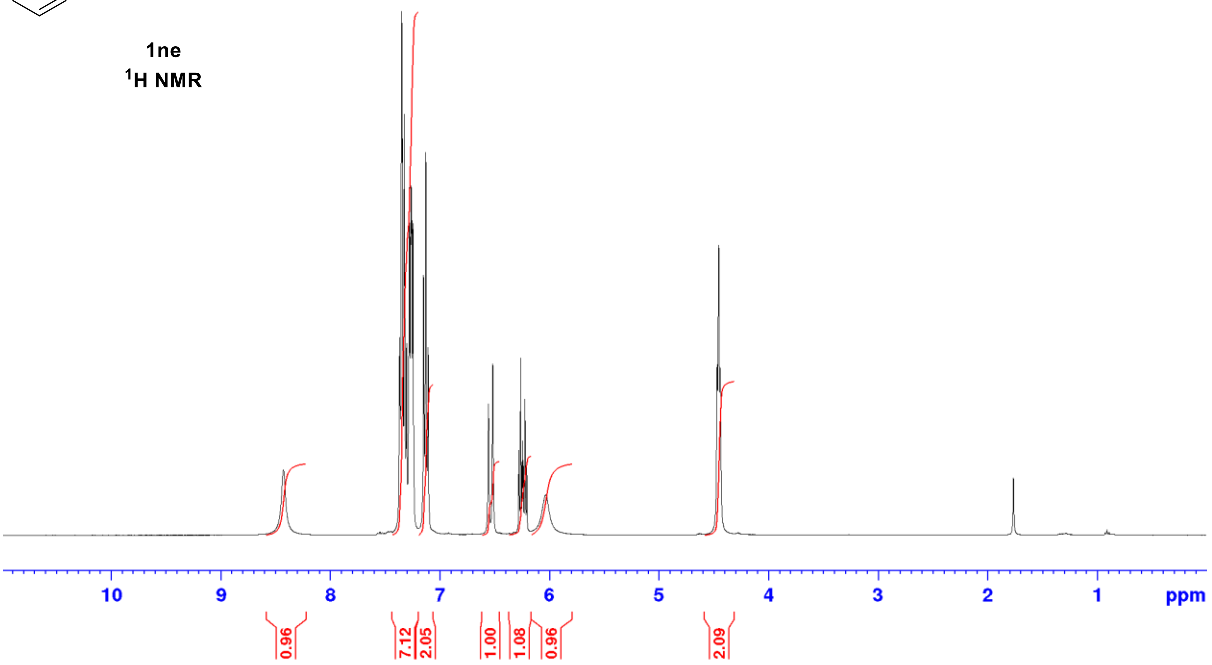

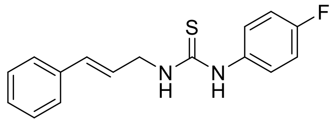

1ne

^{13}C NMR

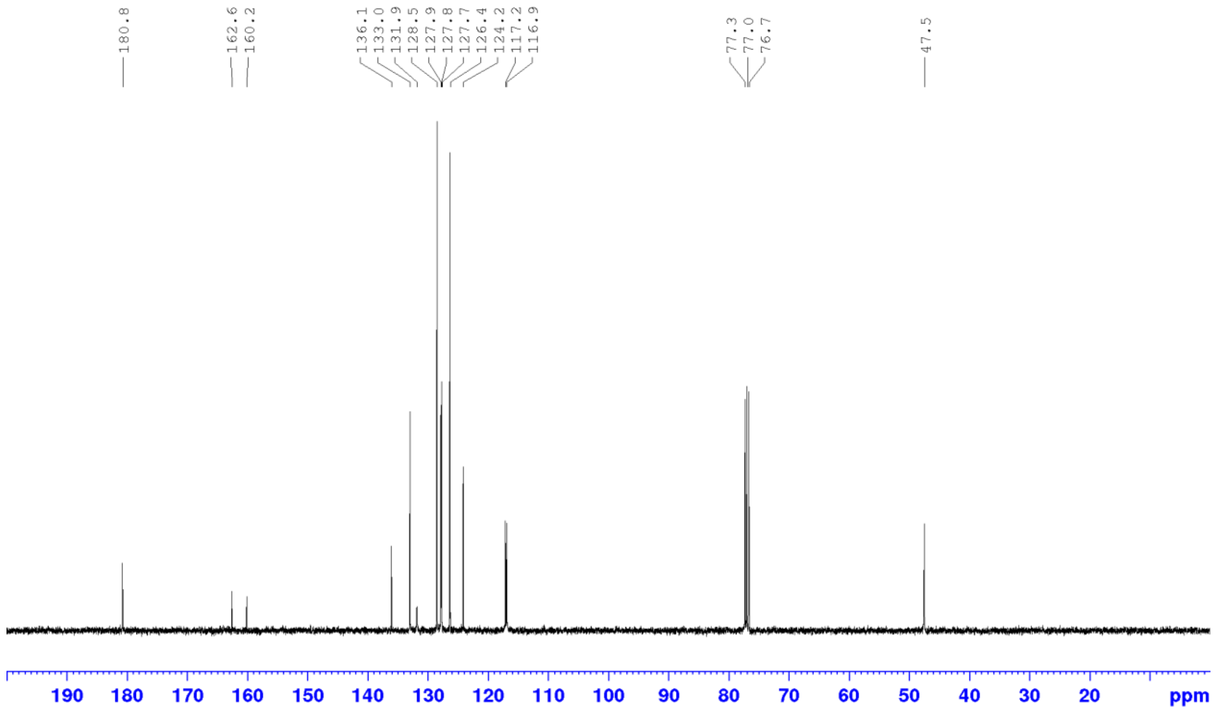

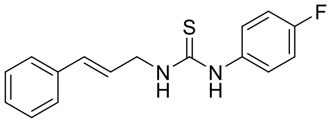

1ne

^{19}F NMR

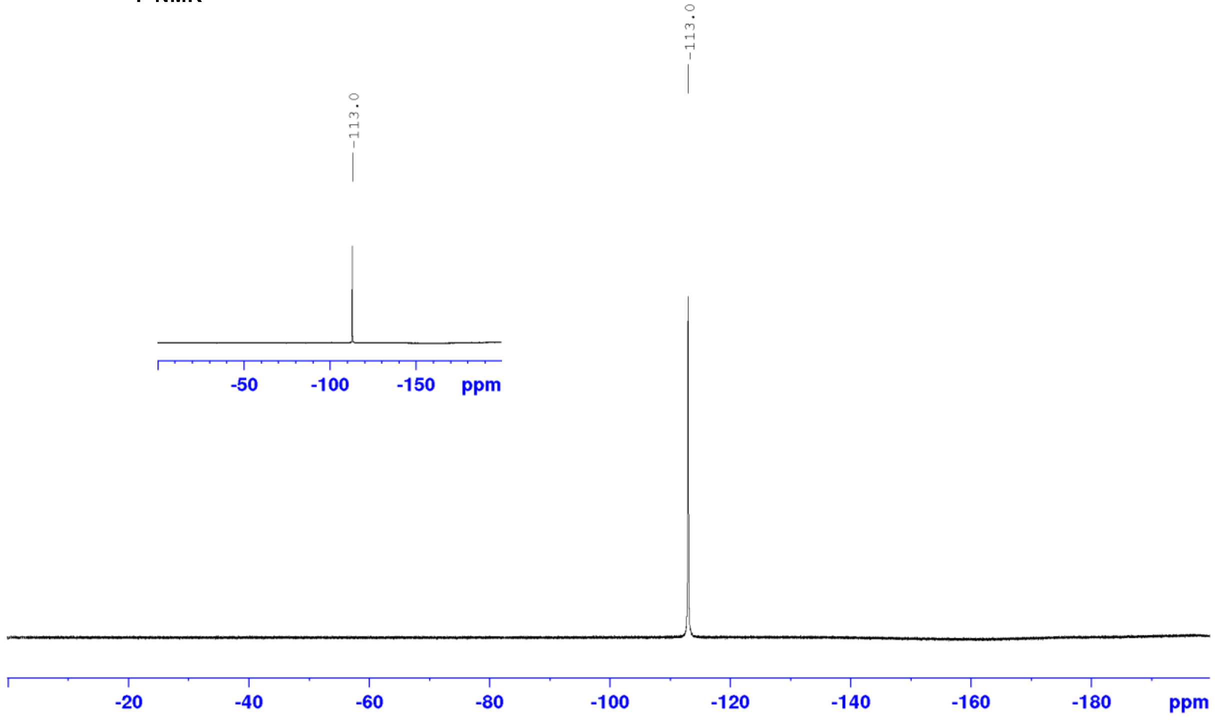

1-(2-(Cyclohex-1-en-1-yl)ethyl)-3-phenylthiourea (1ra)

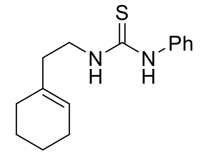

1ra
^{1}H NMR

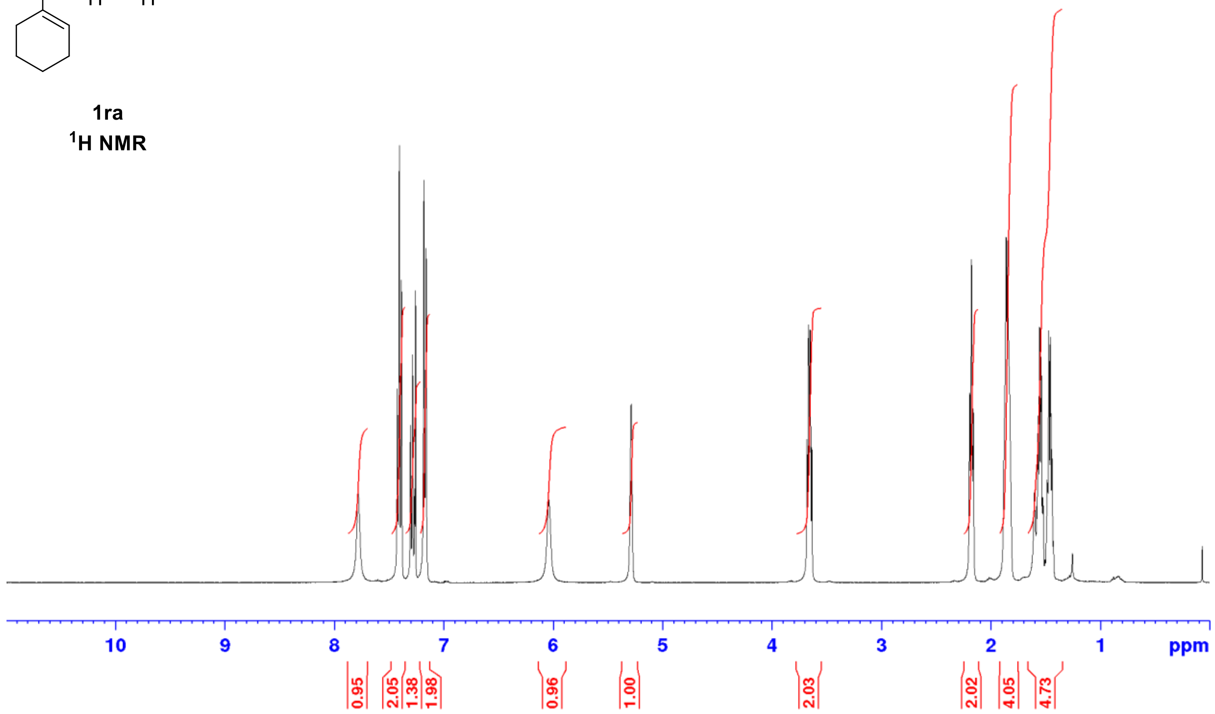

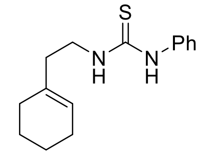

1ra
^{13}C NMR

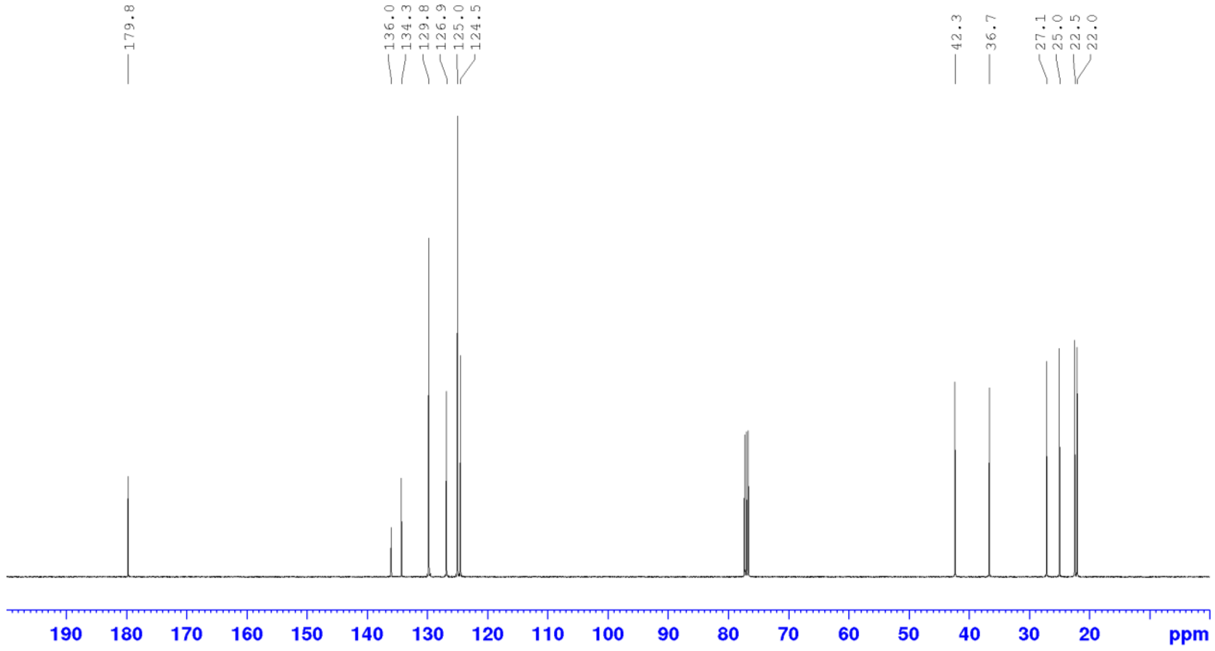

1-(Tert-butyl)-3-(2-(cyclohex-1-en-1-yl)ethyl)thiourea (1rb)

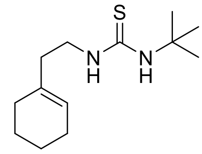

1rb
^{1}H NMR

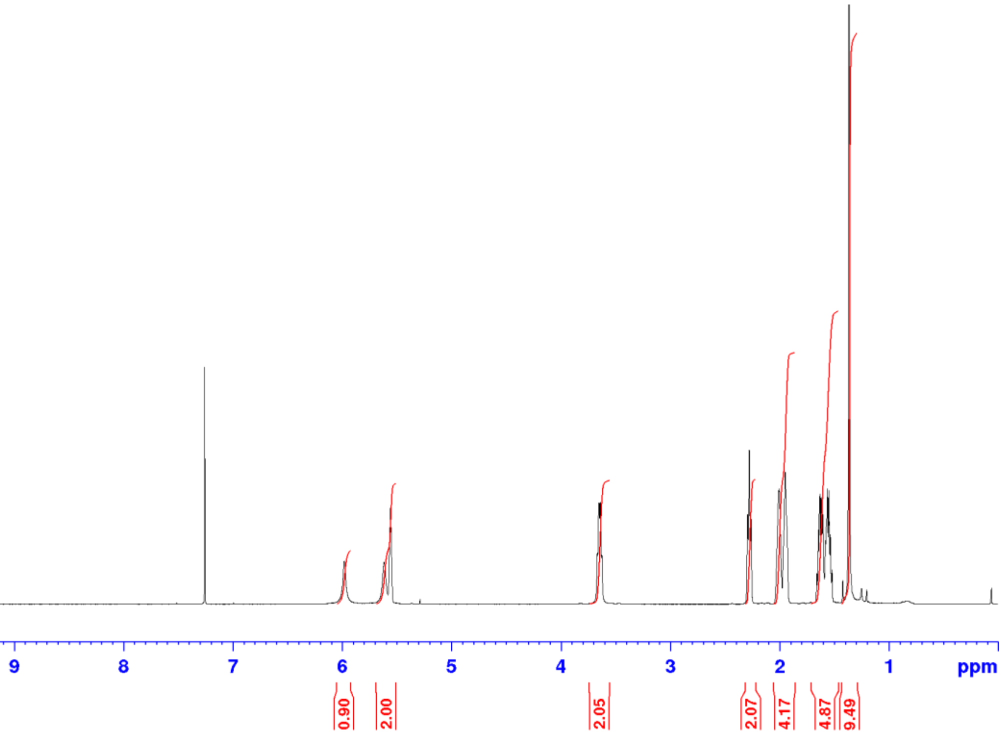

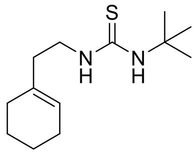

1rb
^{13}C NMR

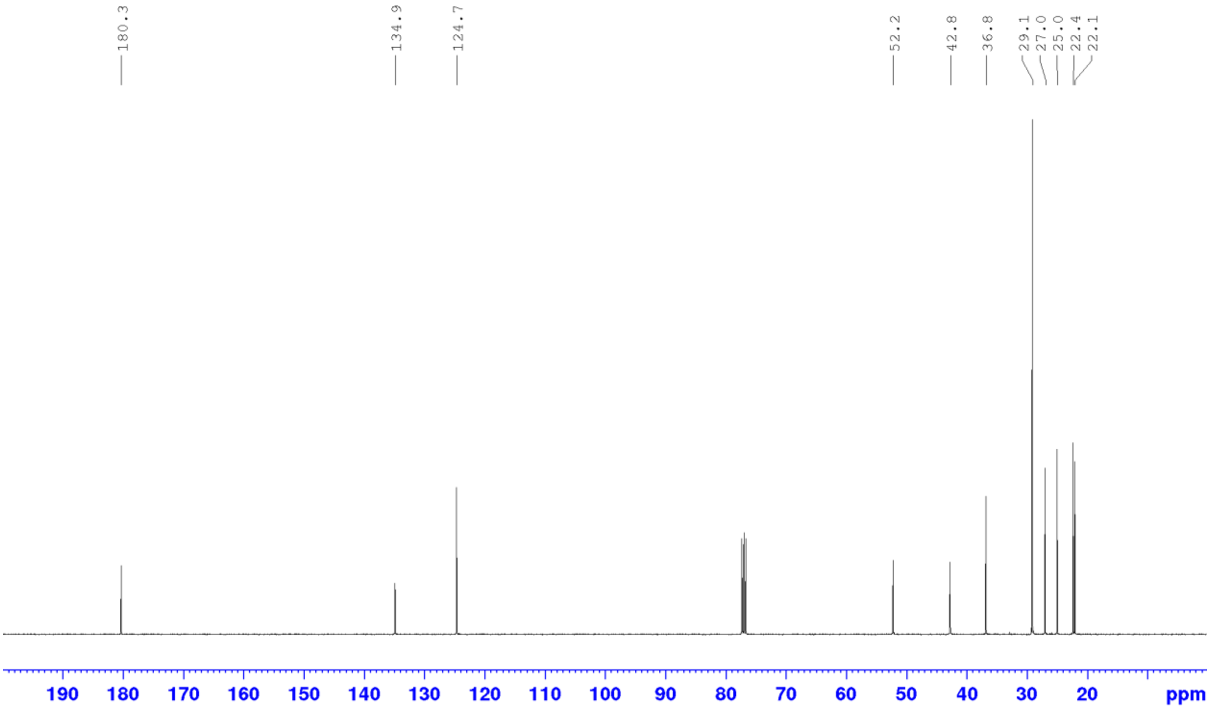

1-(2-Allyl-4-fluorophenyl)-3-phenylthiourea (1sa)

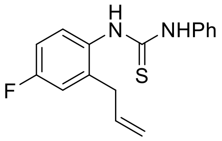

1sa
^{1}H NMR

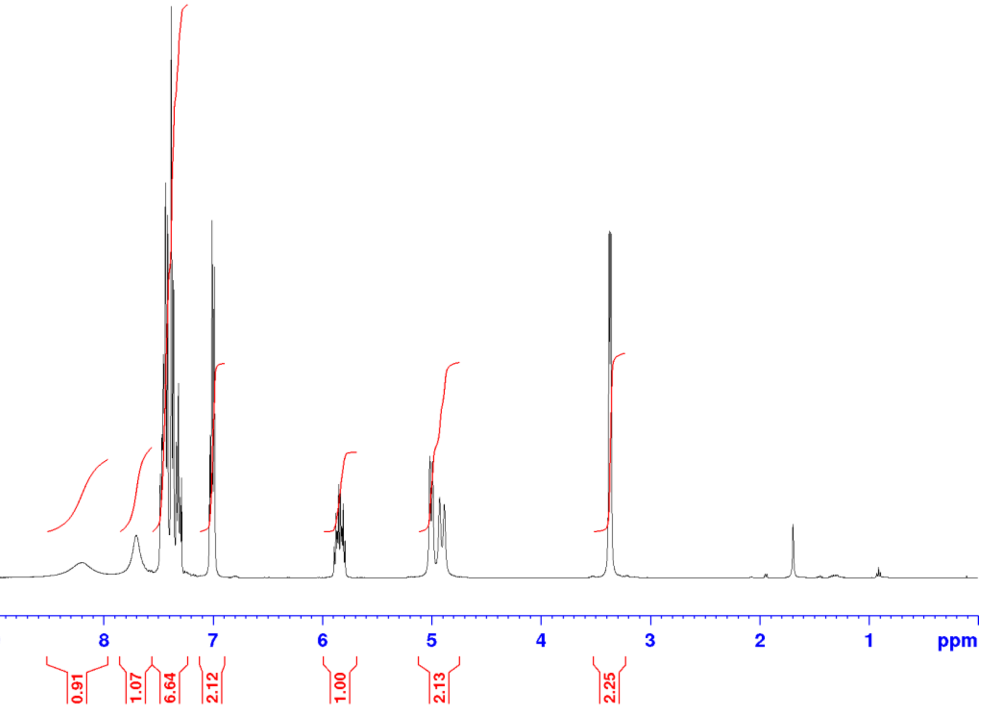

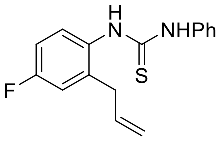

1sa
^{13}C NMR

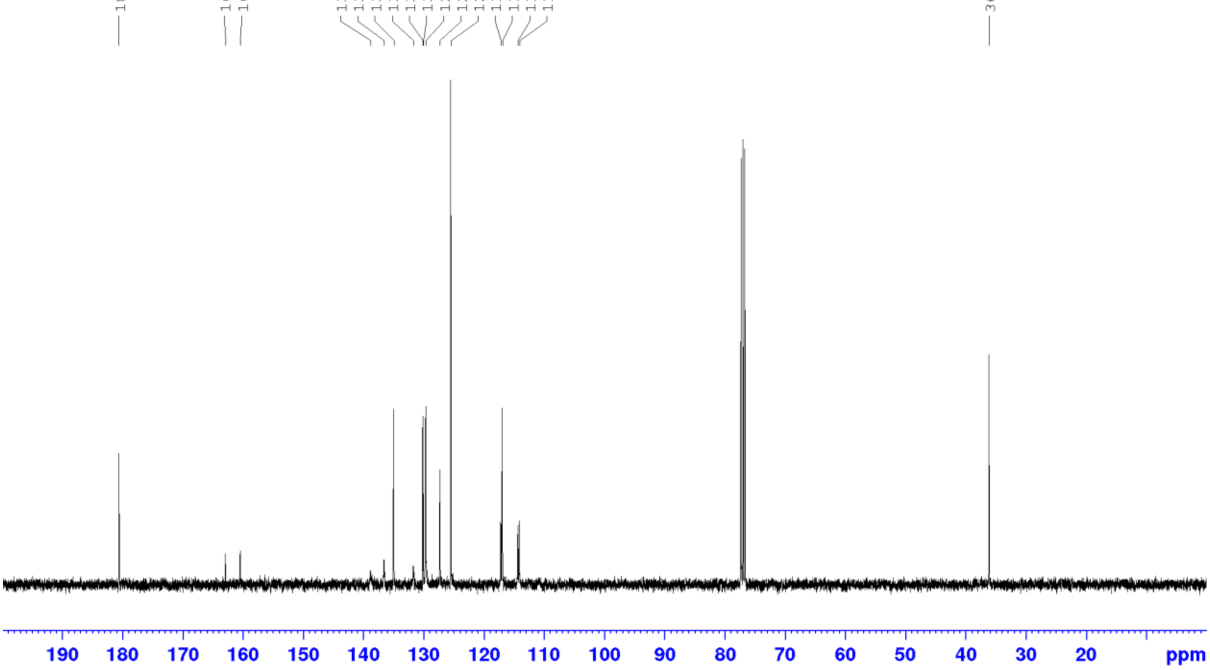

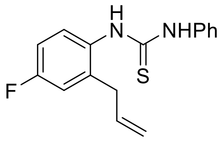

1sa

^{19}F NMR

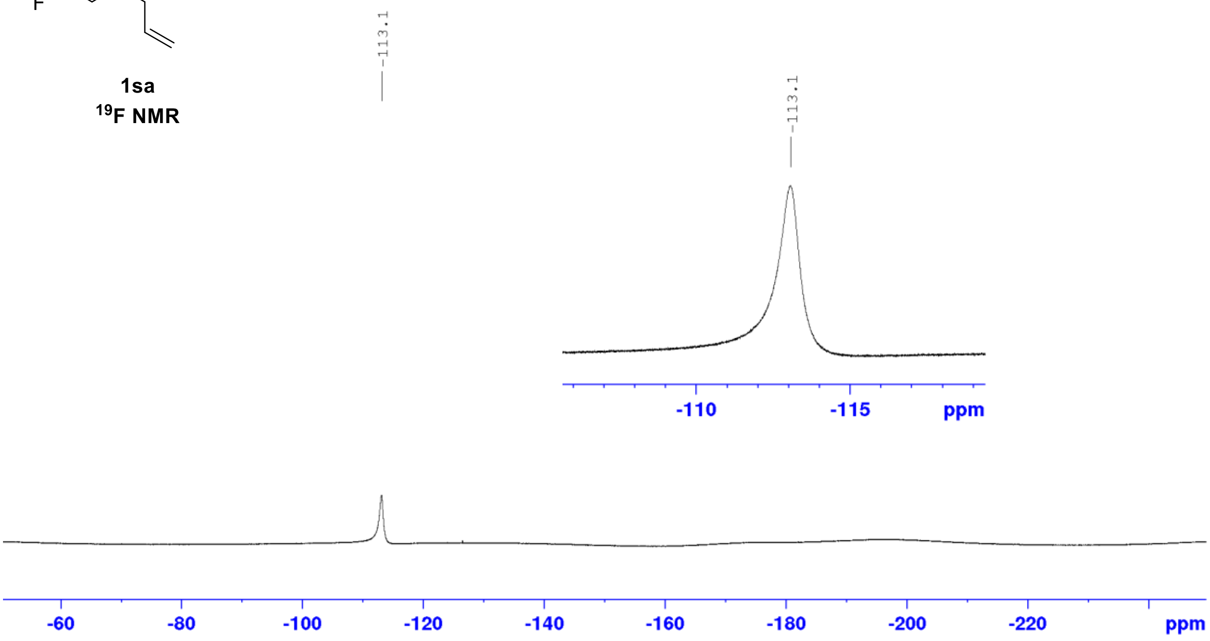

N-Phenyl-5-(2,2,2-trifluoroethyl)-4,5-dihydrothiazol-2-amine (2aa)

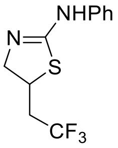

2aa

^{1}H NMR

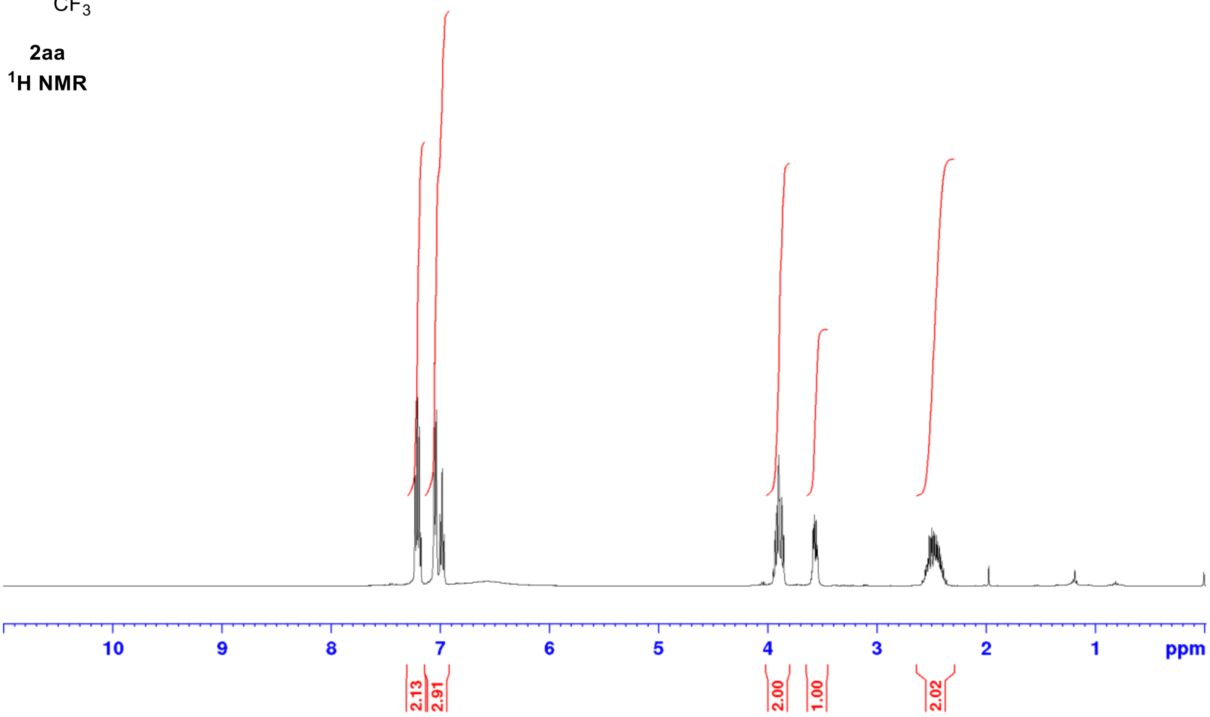

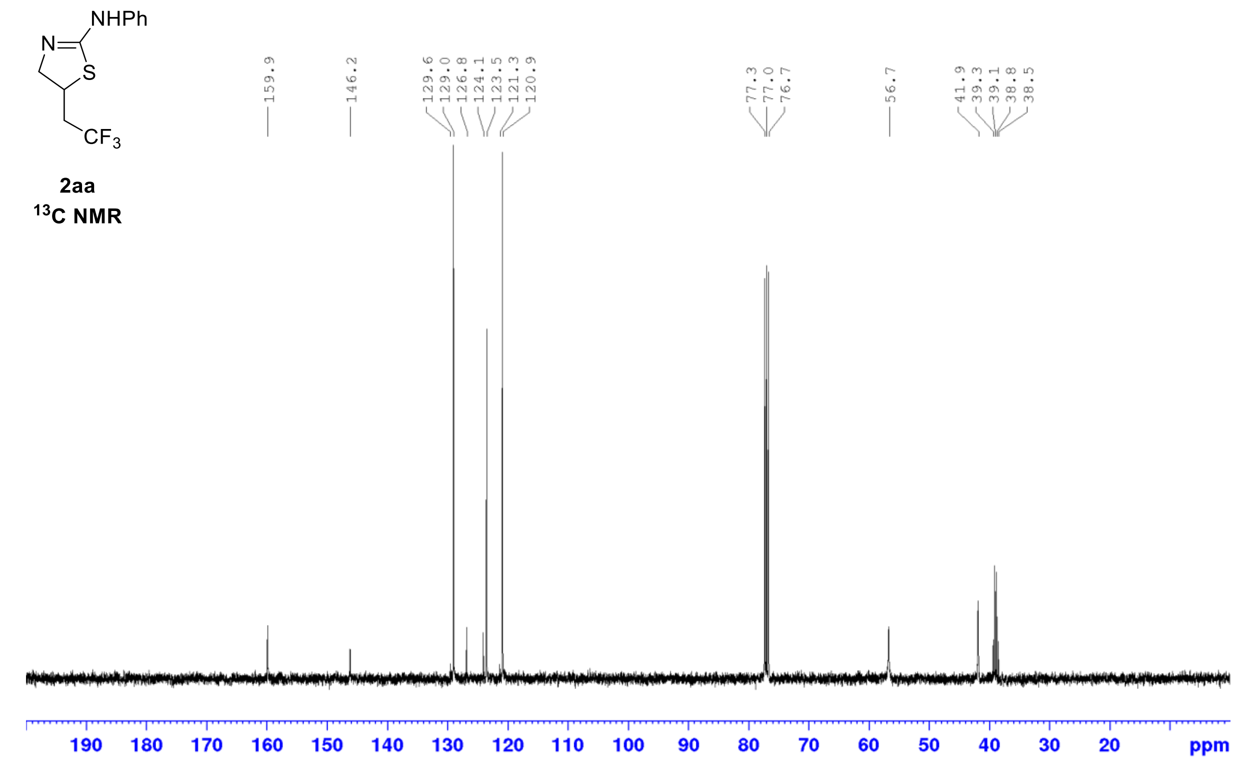
NHPh
N
S
CF3
2aa
13C NMR
159.9
146.2
129.6
129.0
126.8
124.1
123.5
121.3
120.9
77.3
77.0
76.7
56.7
41.9
39.3
39.1
38.8
38.5
190 180 170 160 150 140 130 120 110 100 90 80 70 60 50 40 30 20 ppm

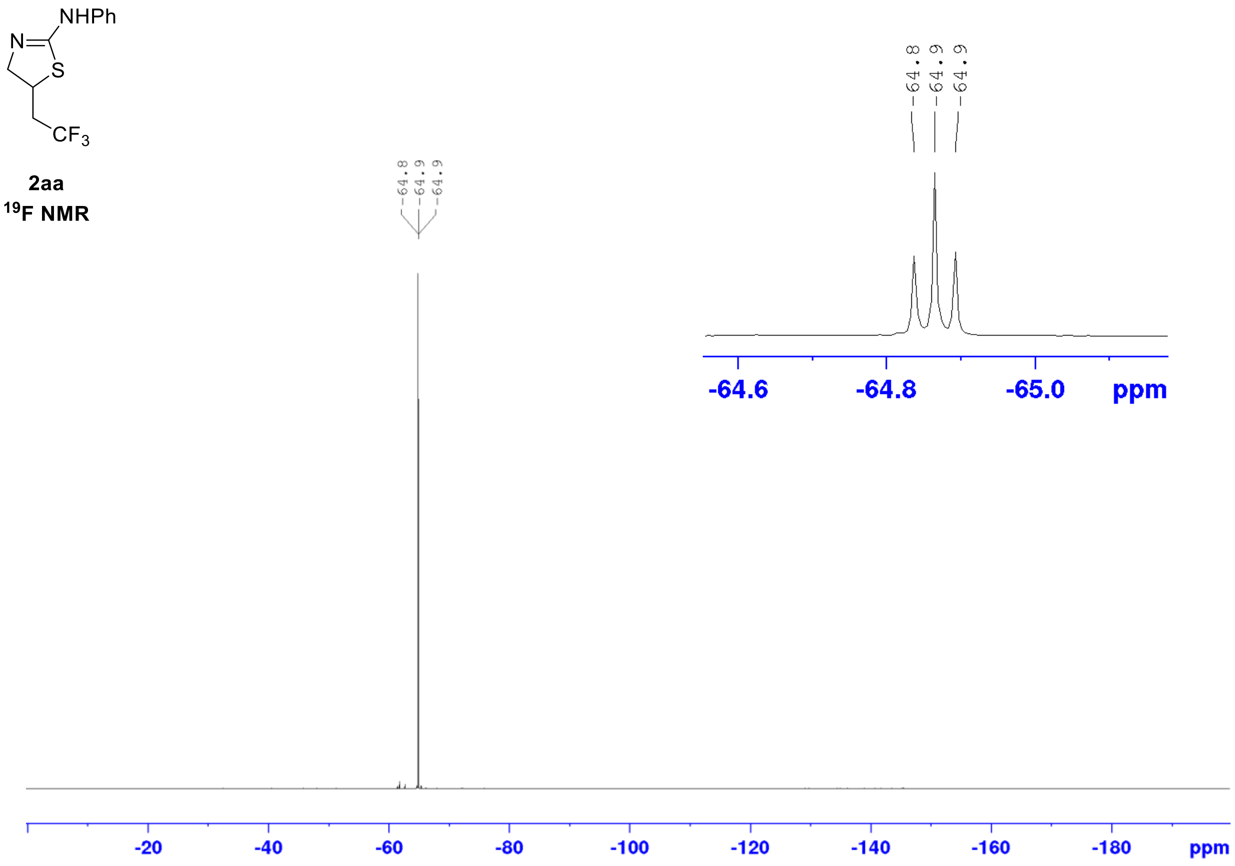
NHPh
N
S
CF3
2aa
19F NMR
-64.8
-64.9
-64.9
-64.6
-64.8
-65.0
ppm
-20 -40 -60 -80 -100 -120 -140 -160 -180 ppm

5-Methyl-*N*-phenyl-5-(2,2,2-trifluoroethyl)-4,5-dihydrothiazol-2-amine (2ba)

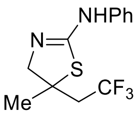

2ba
^{1}H NMR

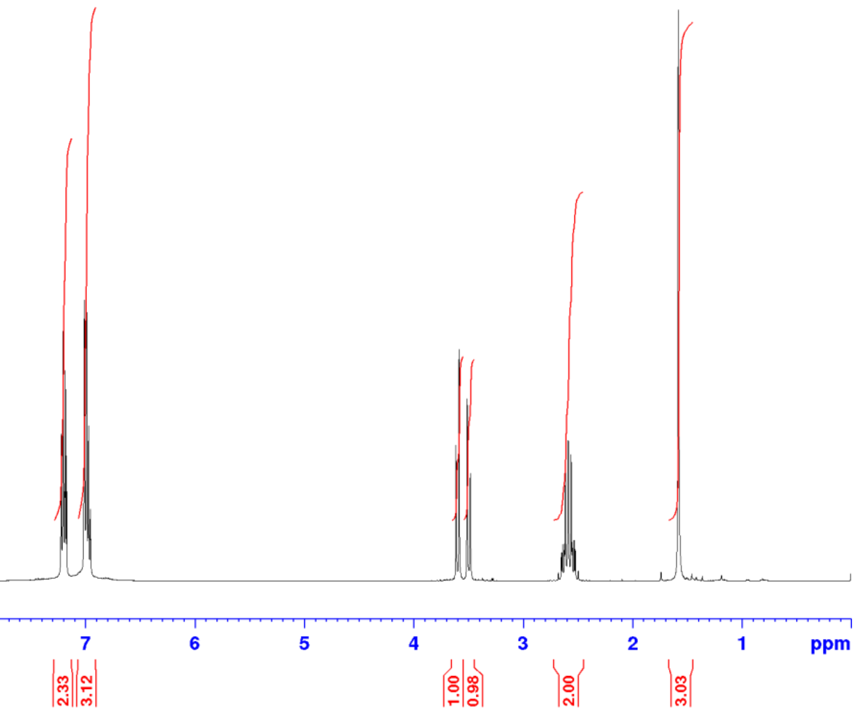

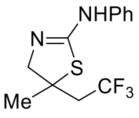

2ba
^{13}C NMR

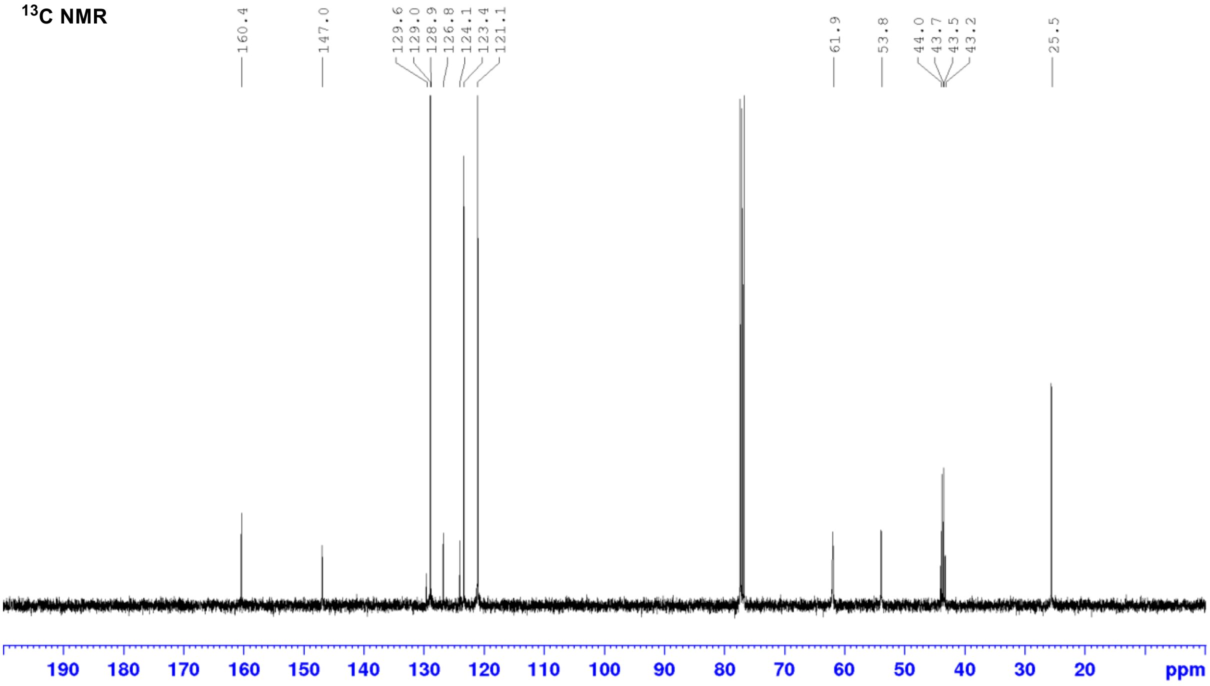

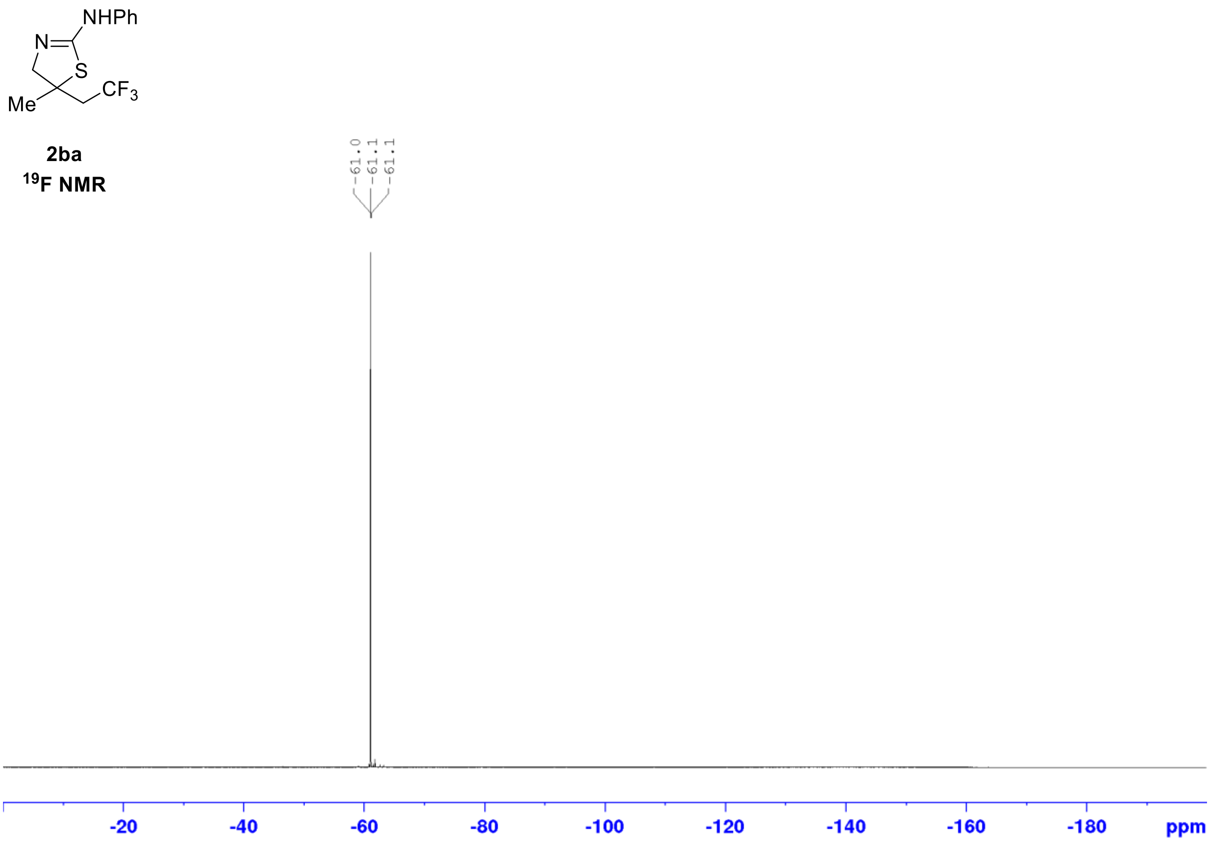

N,5-Diphenyl-5-(2,2,2-trifluoroethyl)-4,5-dihydrothiazol-2-amine (2ca)

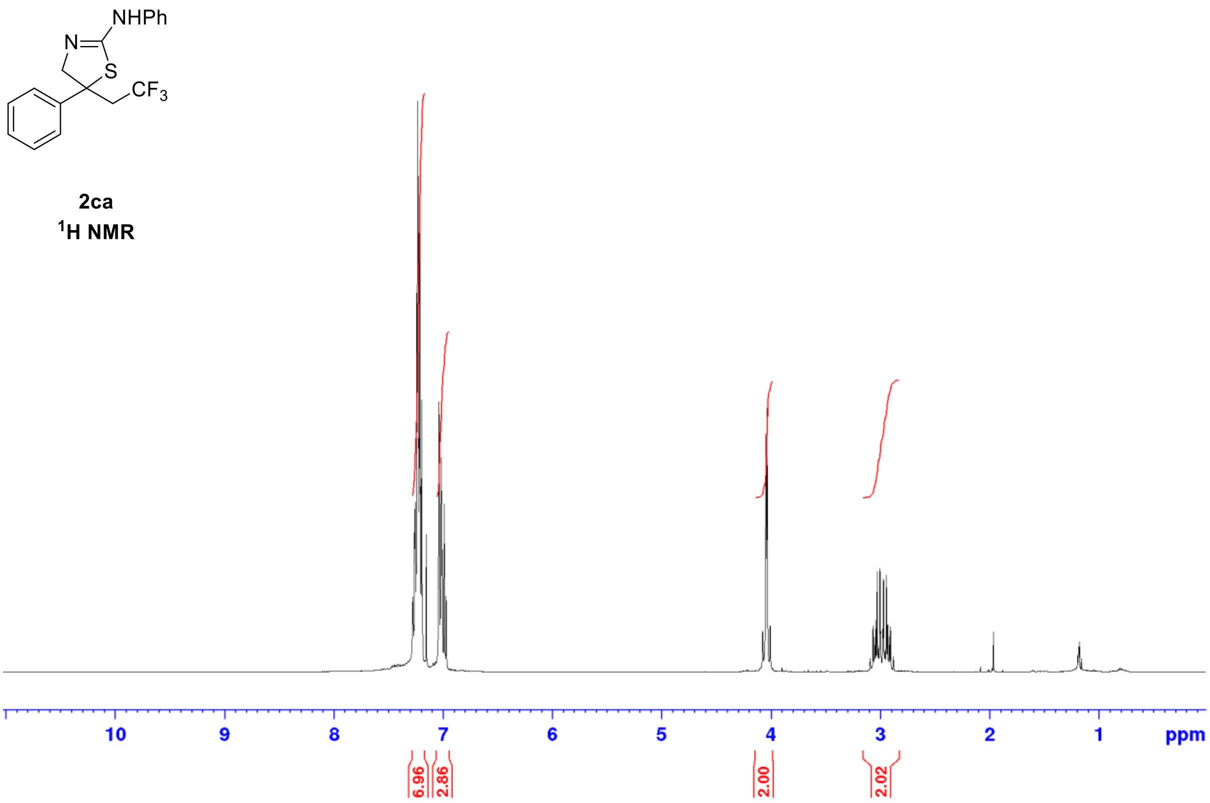

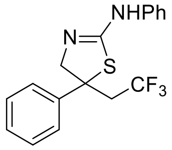

2ca
^{13}C NMR

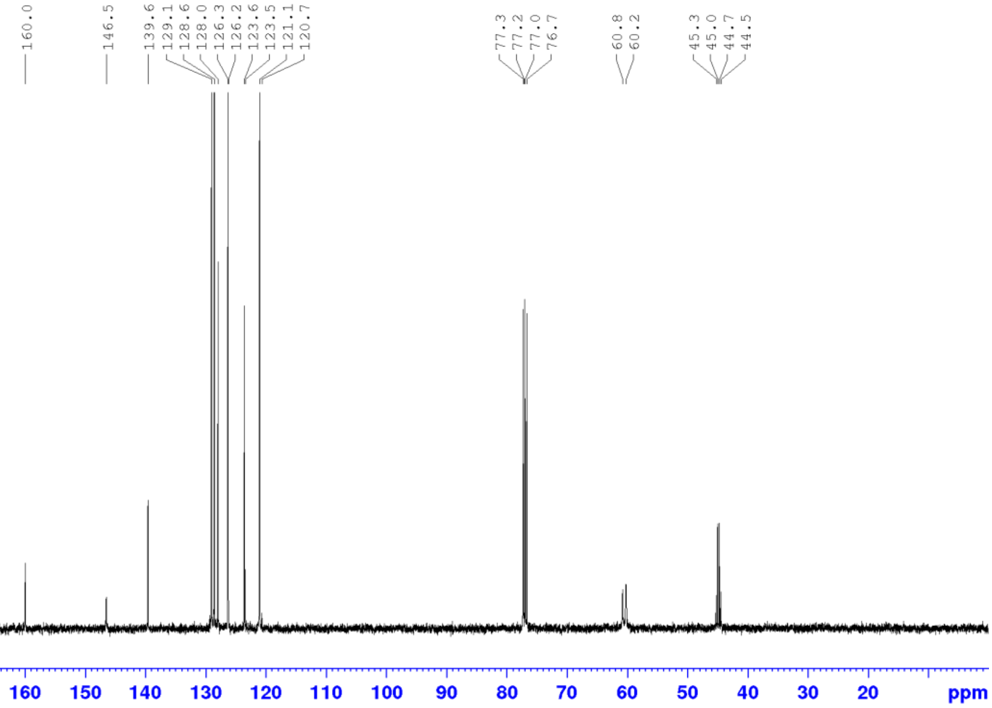

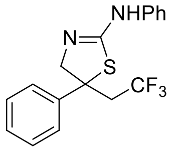

2ca
^{19}F NMR

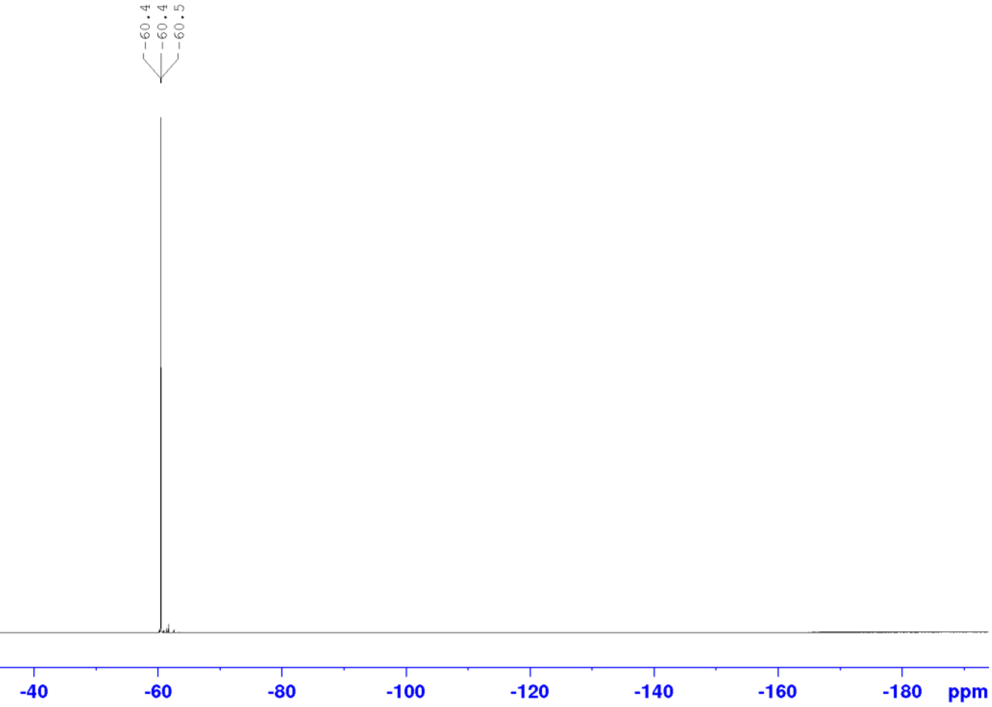

5-(4-Fluorophenyl)-N-phenyl-5-(2,2,2-trifluoroethyl)-4,5-dihydrothiazol-2-amine (2da)

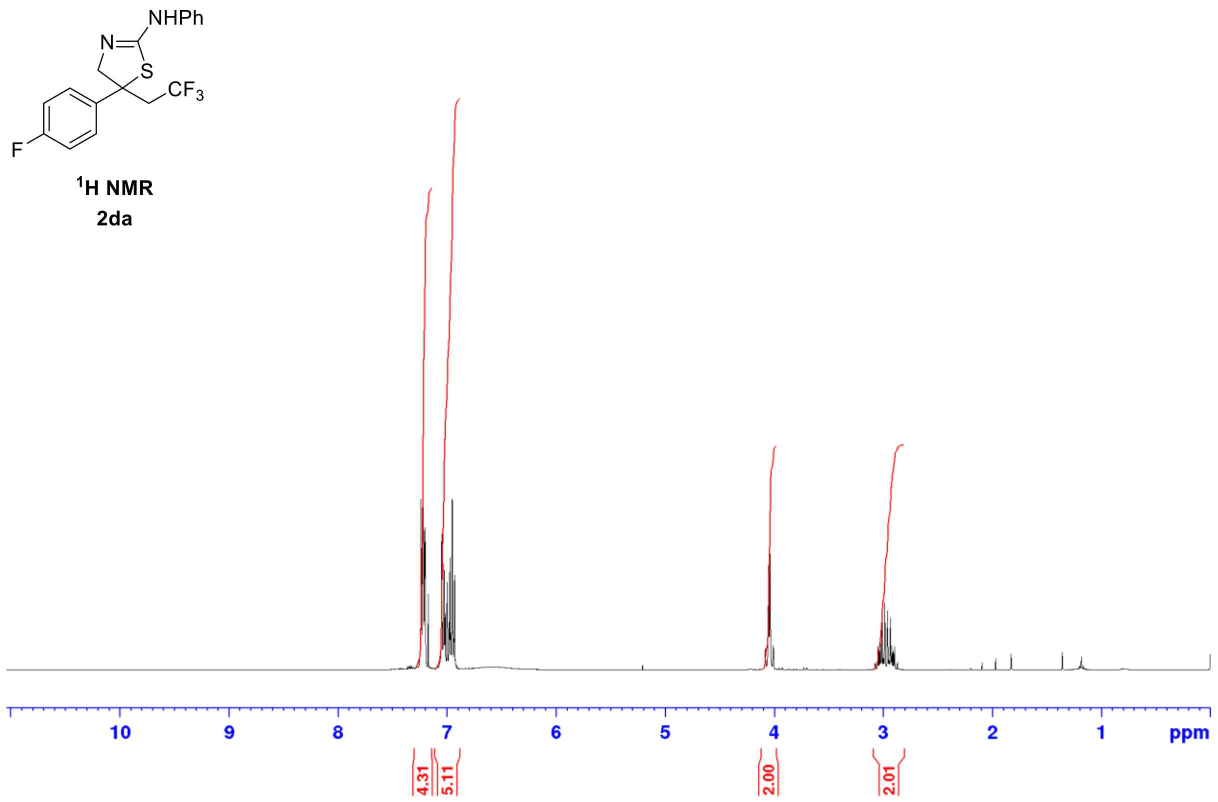

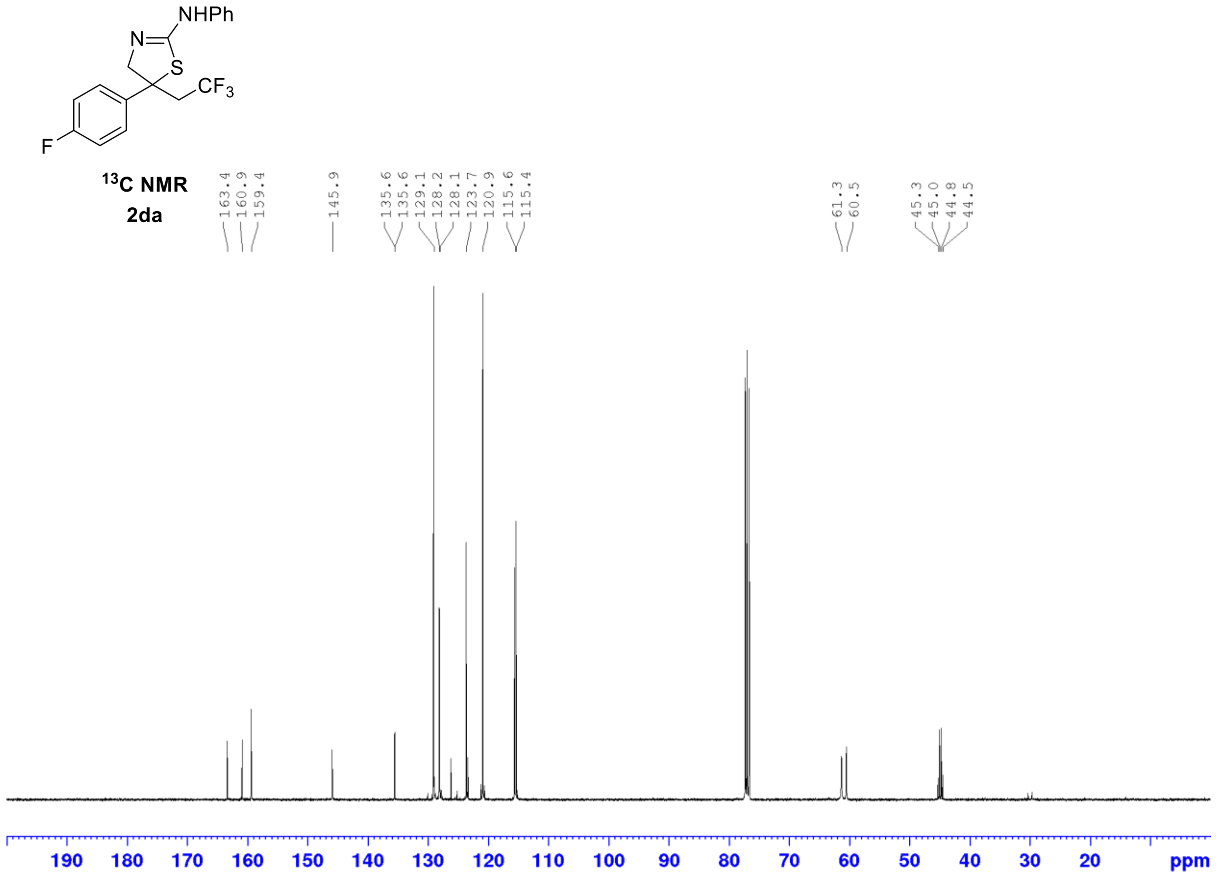

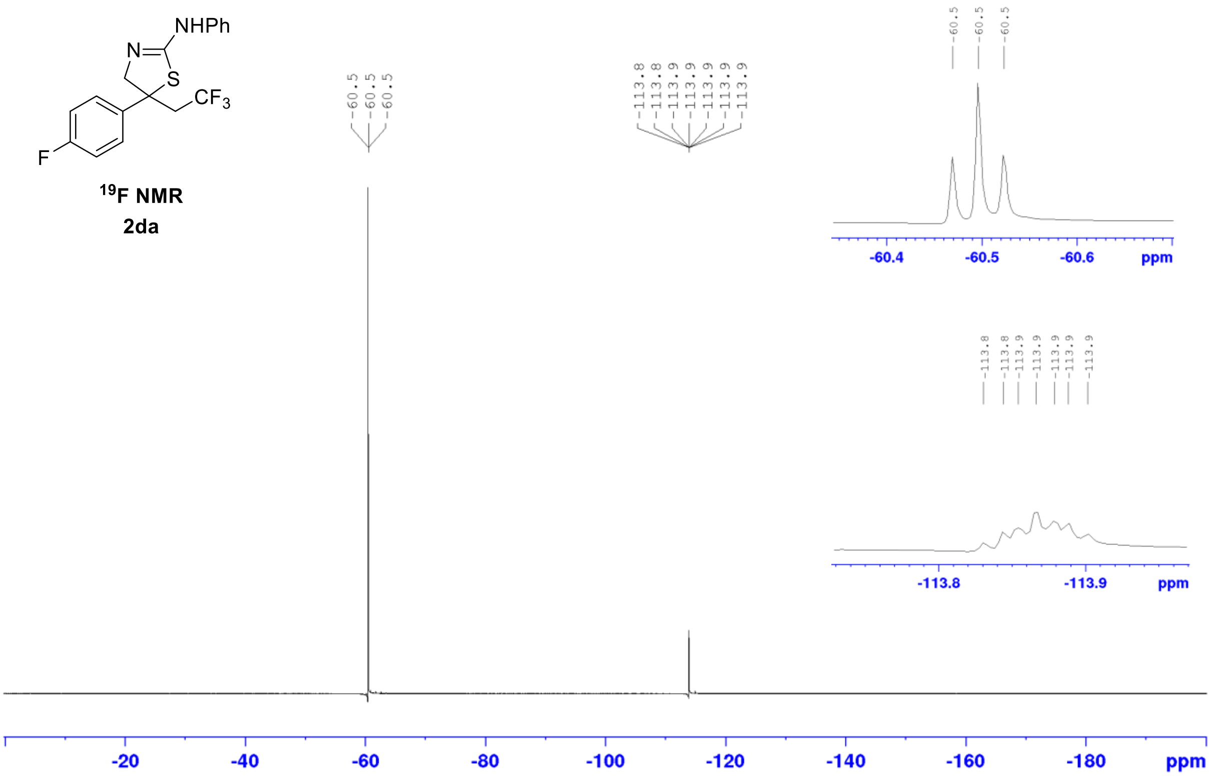

5-(4-Chlorophenyl)-N-phenyl-5-(2,2,2-trifluoroethyl)-4,5-dihydrothiazol-2-amine (2ea)

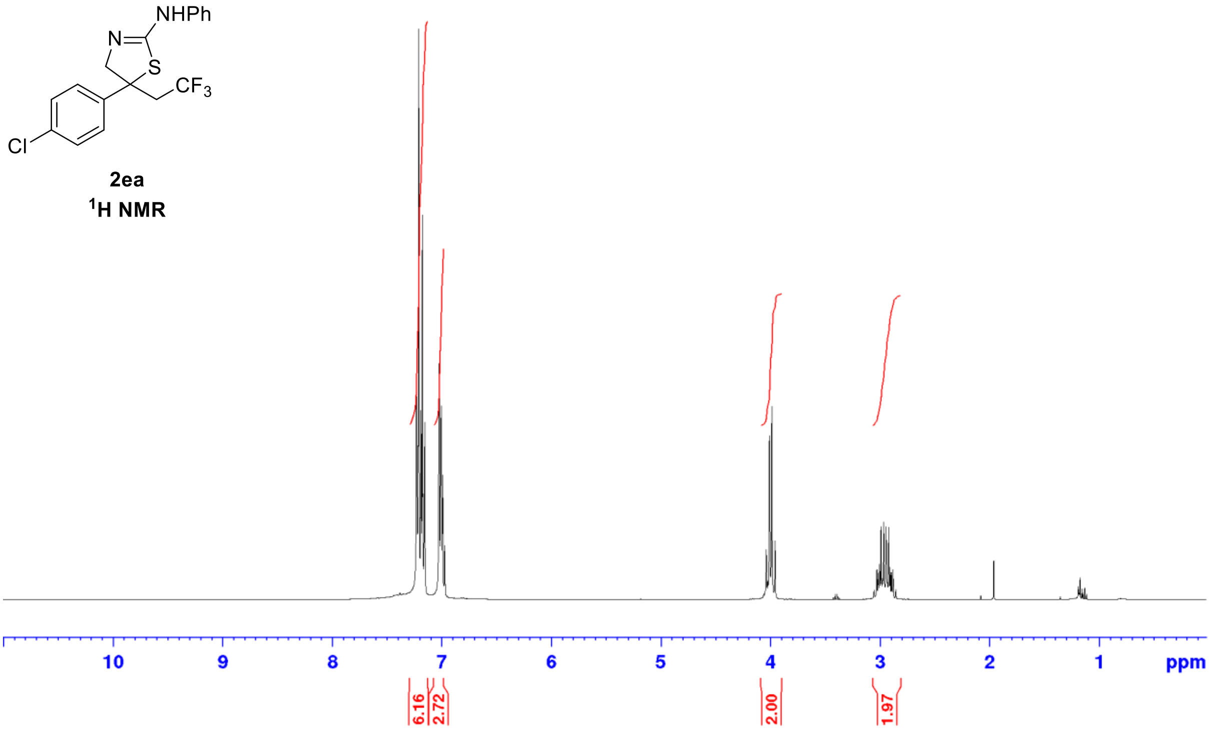

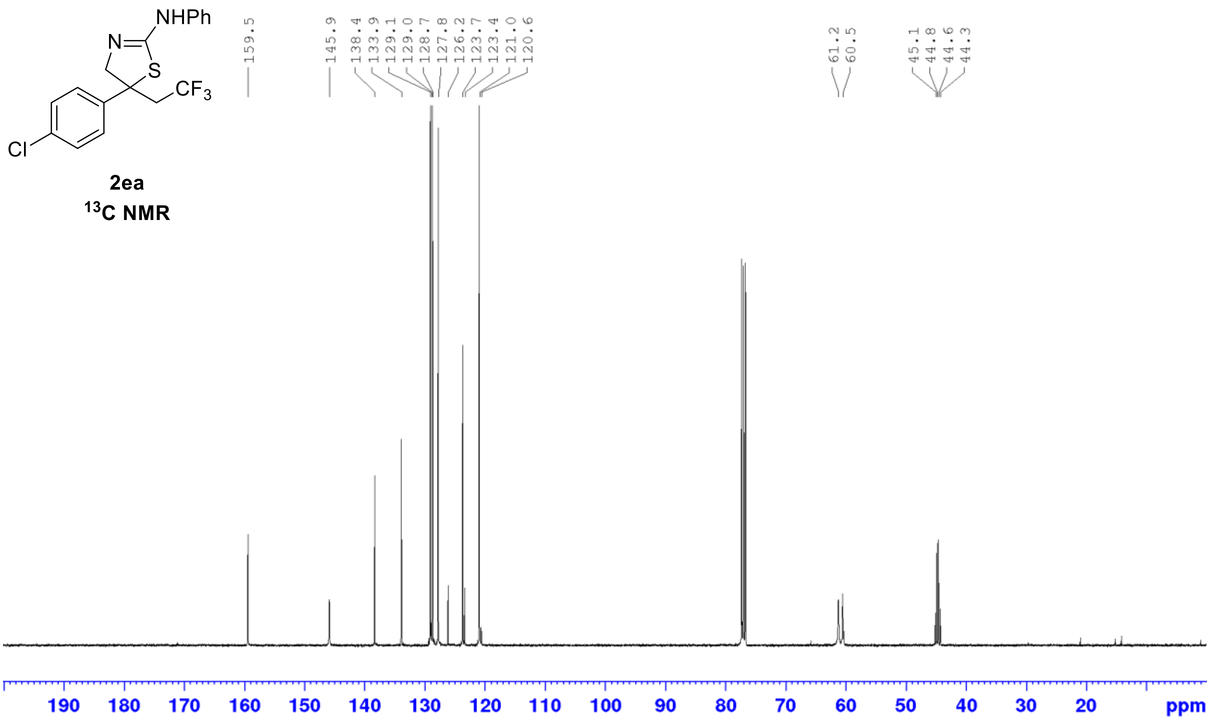

2ea
^{13}C NMR

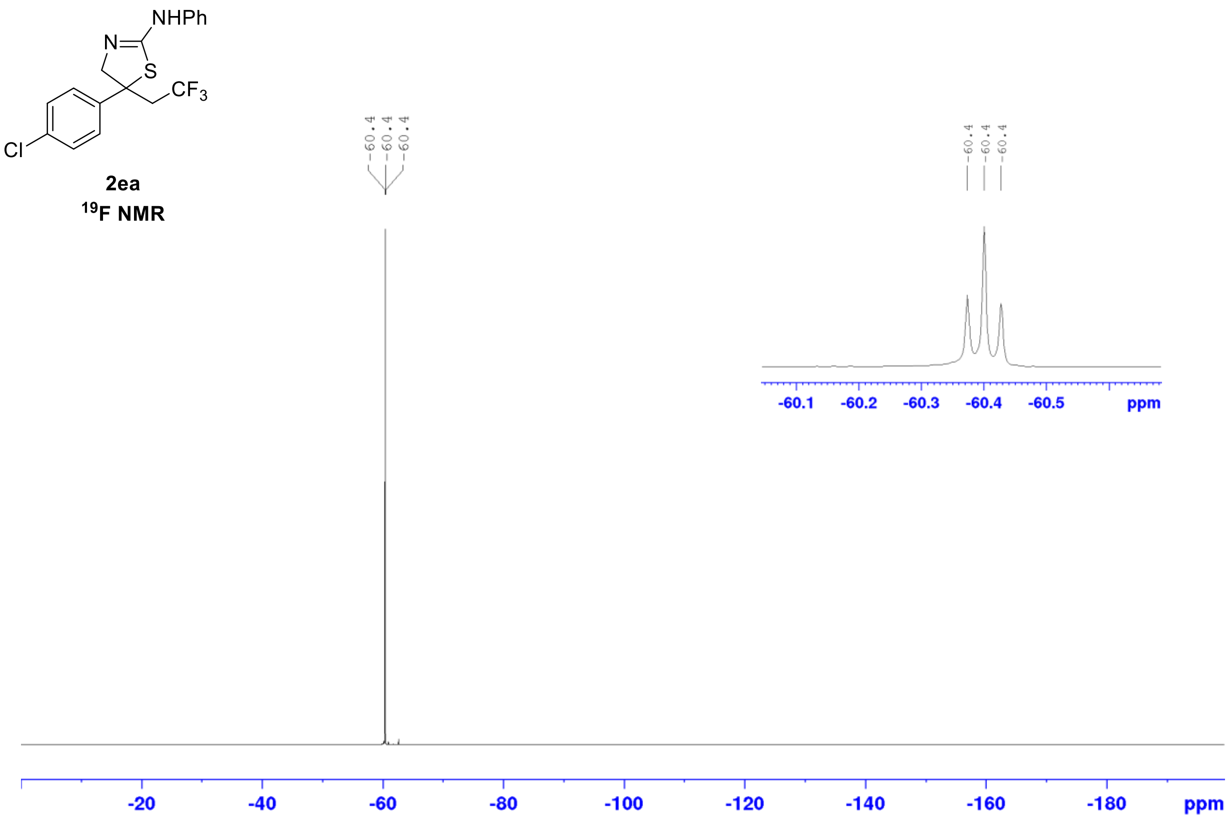

2ea
^{19}F NMR

_N-P_henyl-5-(2,2,2-trifluoroethyl)-5-(4-(trifluoromethyl)phenyl)-4,5-dihydrothiazol-2-amine (2fa)

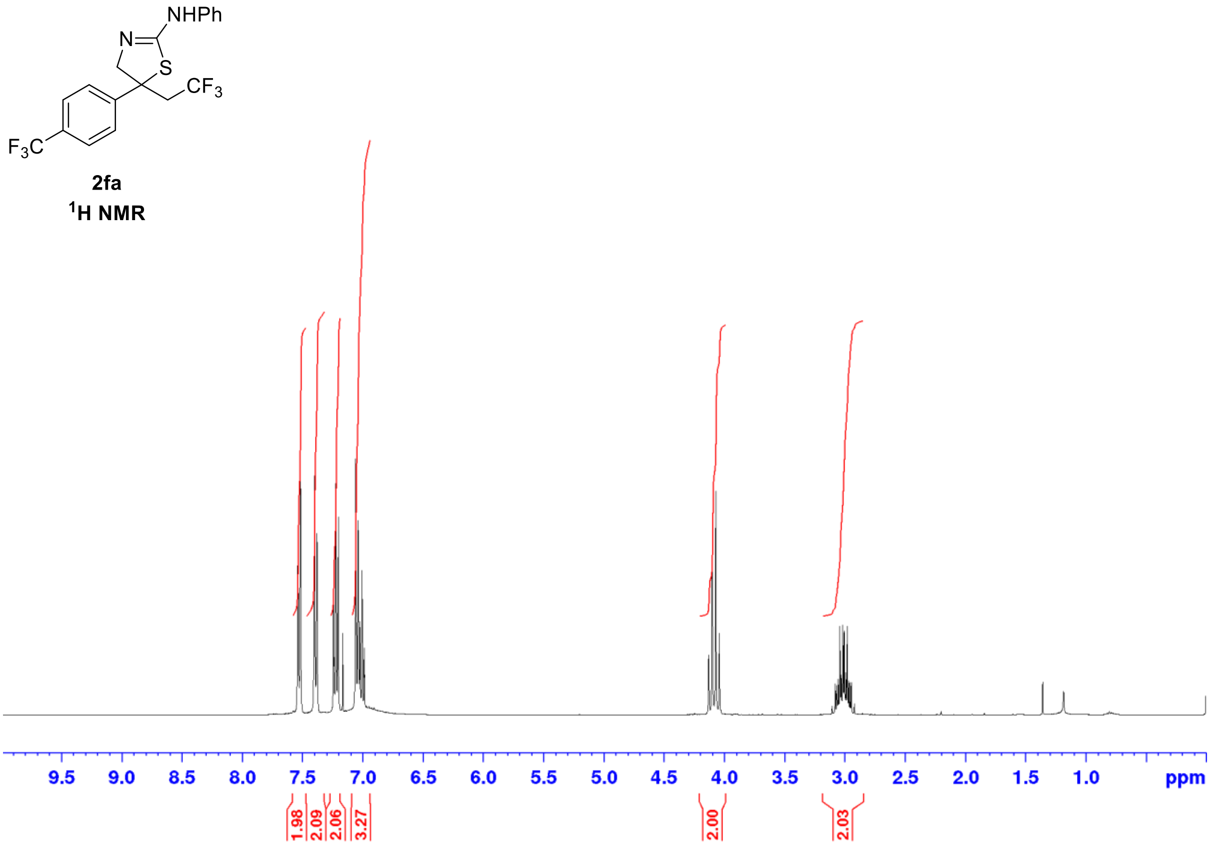

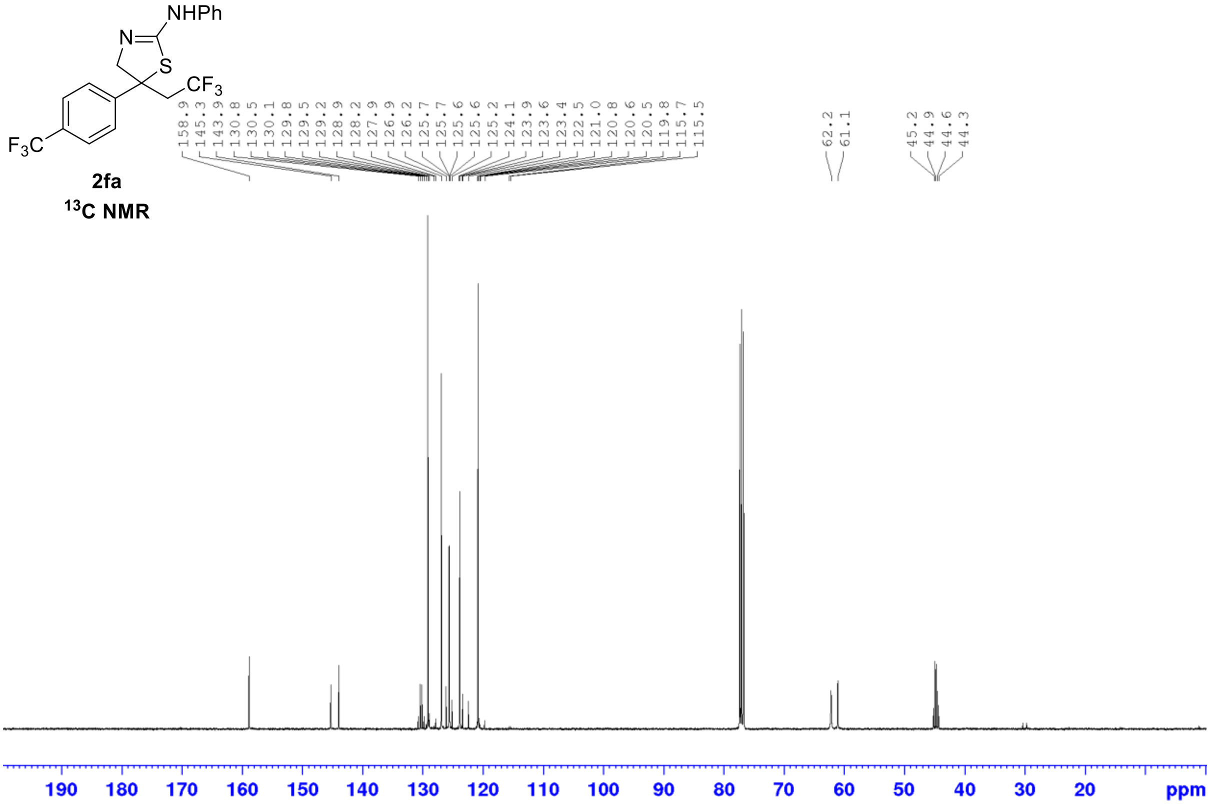

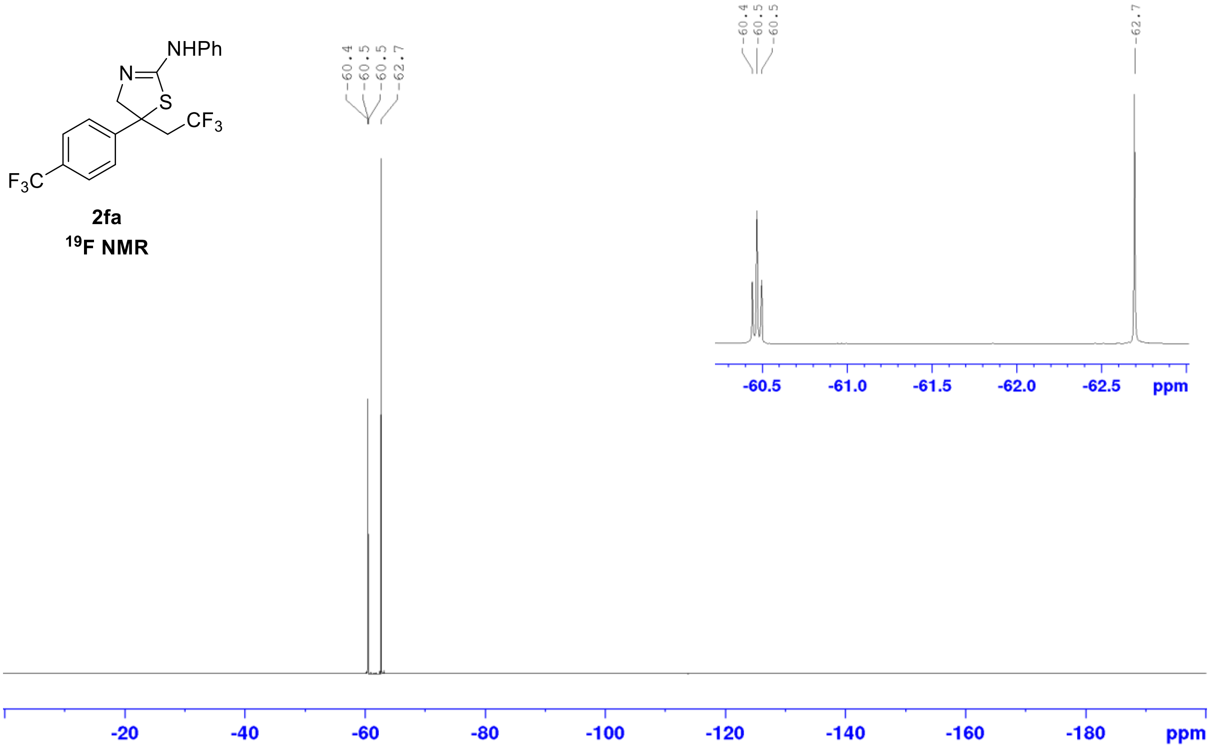

(1*S,2*S**)-*N*-Phenyl-2-(trifluoromethyl)-3,4-dihydro-2H,4'H-spiro[naphthalene-1,5'-thiazol]-2'-amine (2ga)**

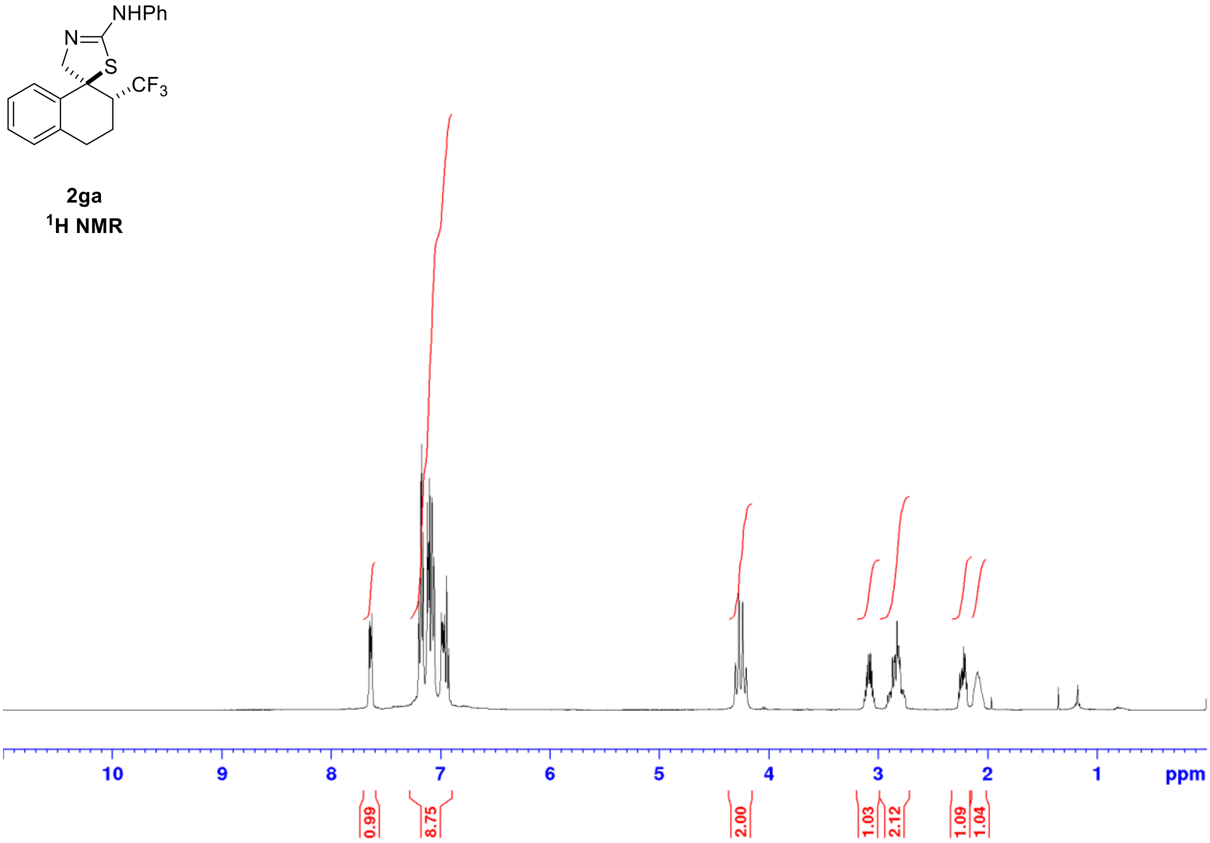

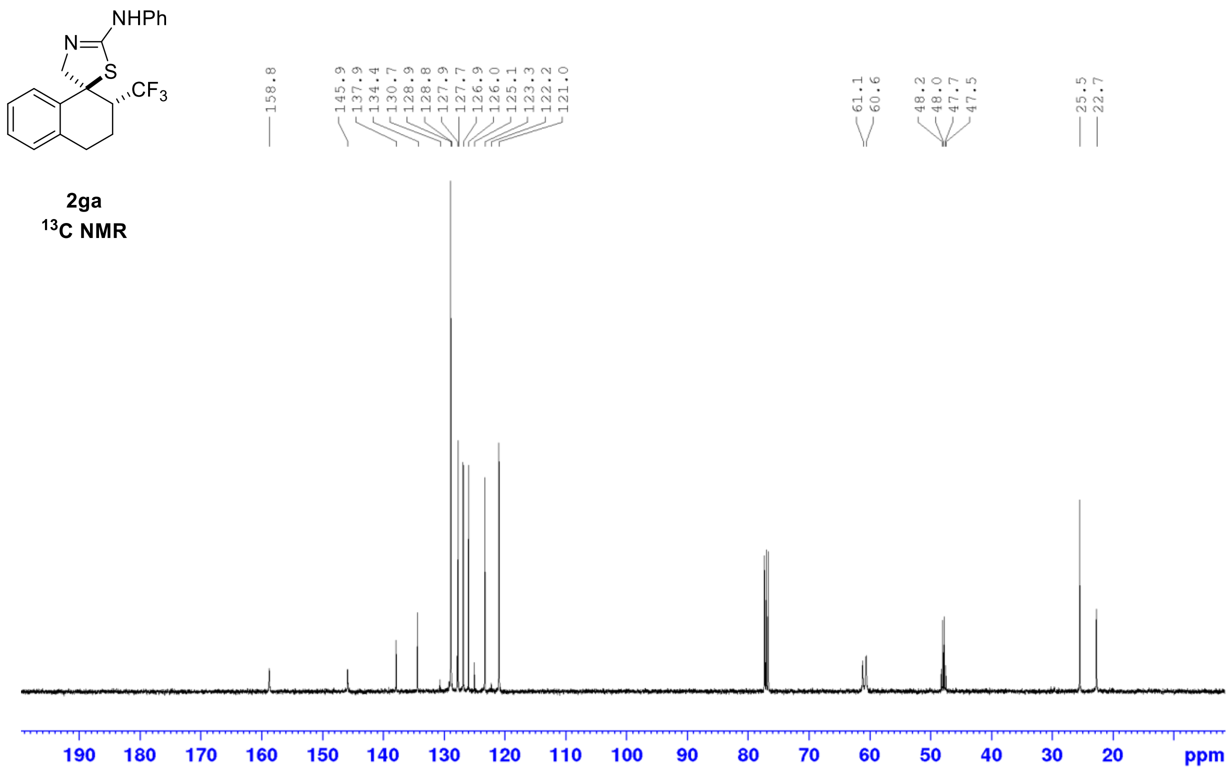

NHPh
N
S
CF3
2ga
13C NMR
158.8
145.9
137.9
134.4
130.7
128.9
128.8
127.9
127.7
126.9
126.0
125.1
123.3
122.2
121.0
61.1
60.6
48.2
48.0
47.7
47.5
25.5
22.7
190 180 170 160 150 140 130 120 110 100 90 80 70 60 50 40 30 20 ppm

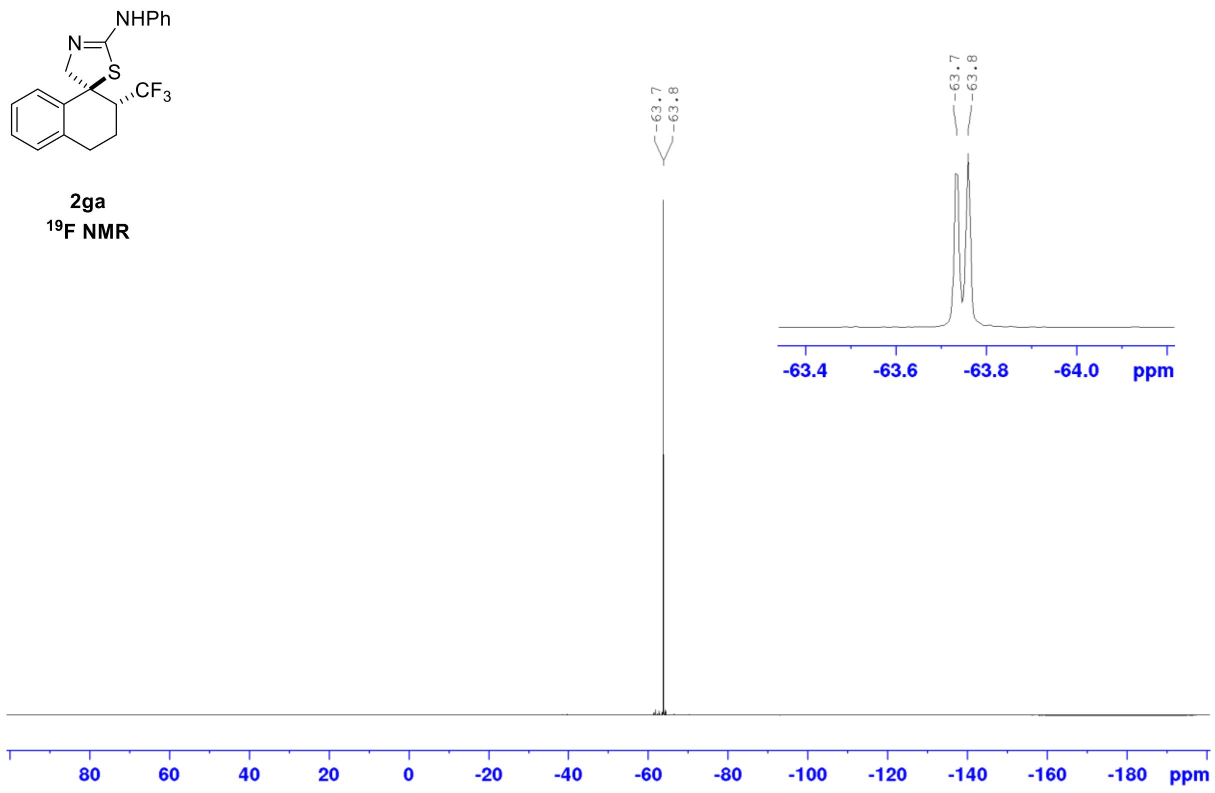

NHPh
N
S
CF3
2ga
19F NMR
-63.7
-63.8
-63.7
-63.8
-63.4 -63.6 -63.8 -64.0 ppm
80 60 40 20 0 -20 -40 -60 -80 -100 -120 -140 -160 -180 ppm

(1*S,2*S**)-*N*-(4-Bromophenyl)-2-(trifluoromethyl)-3,4-dihydro-2H,4'H-spiro[naphthalene-1,5'-thiazol]-2'-amine (2gb)**

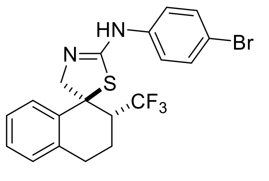

2gb
^{1}H NMR

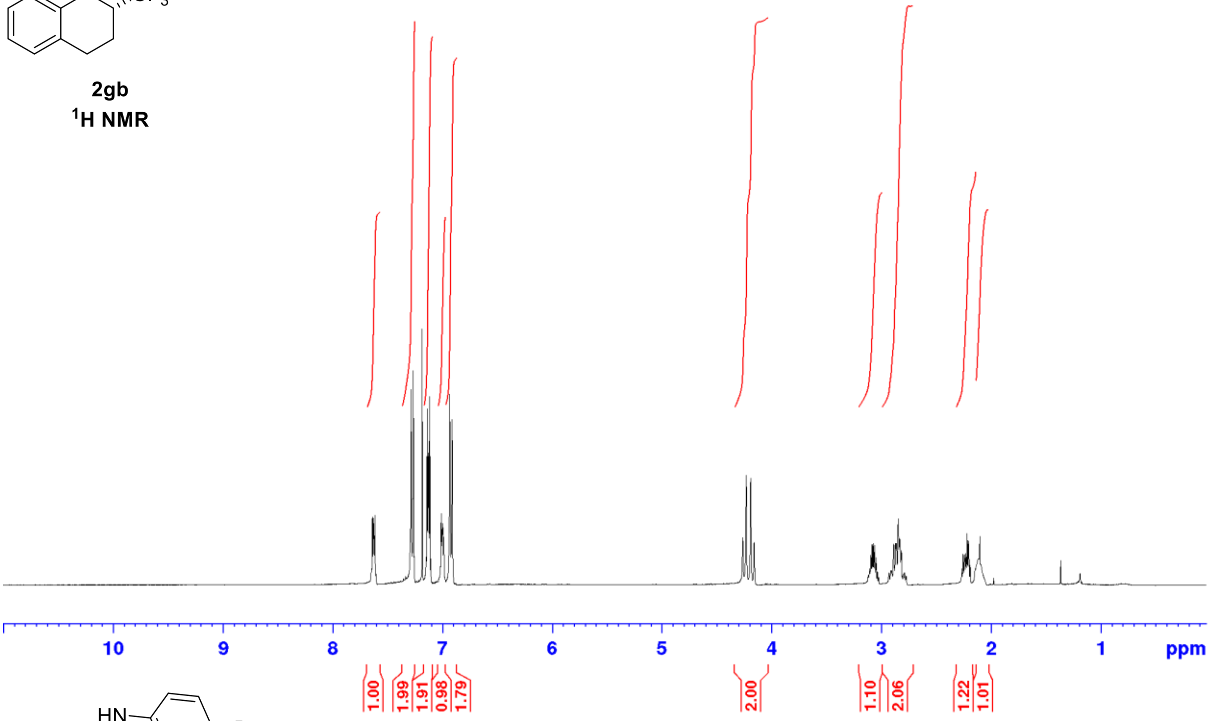

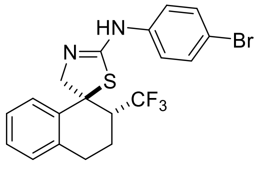

2gb
^{13}C NMR

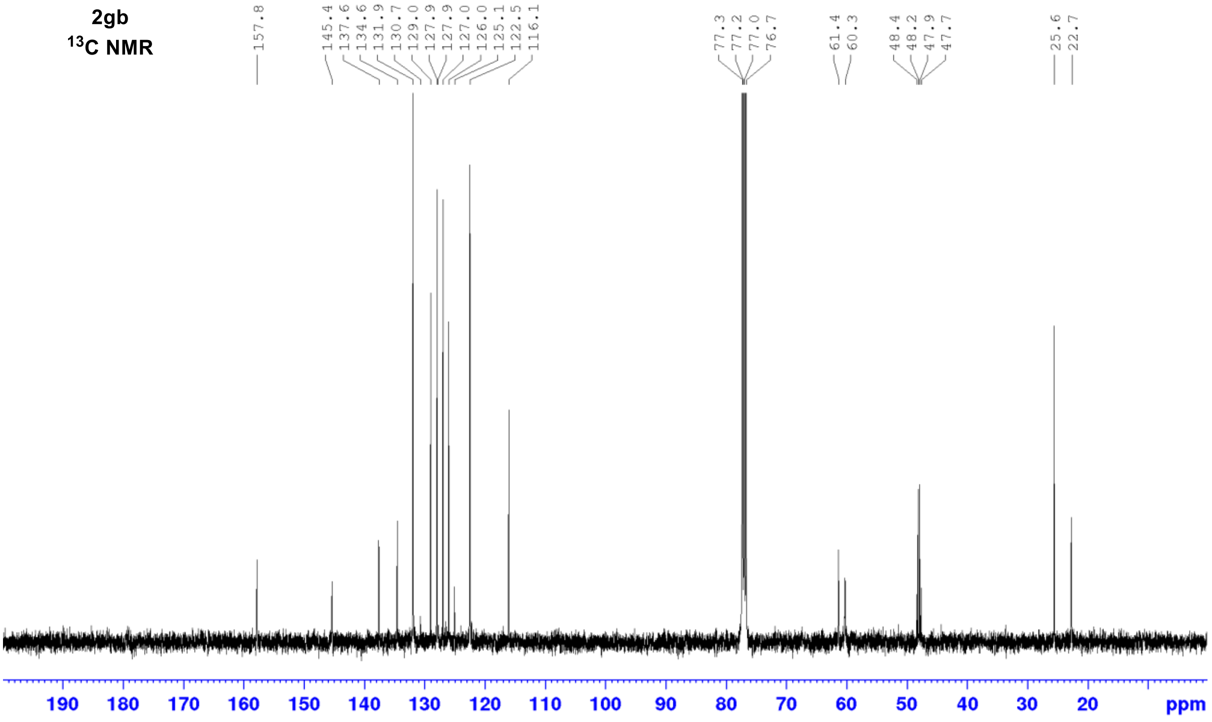

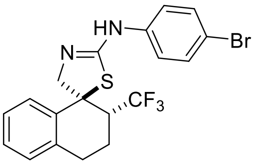

2gb
^{19}F NMR

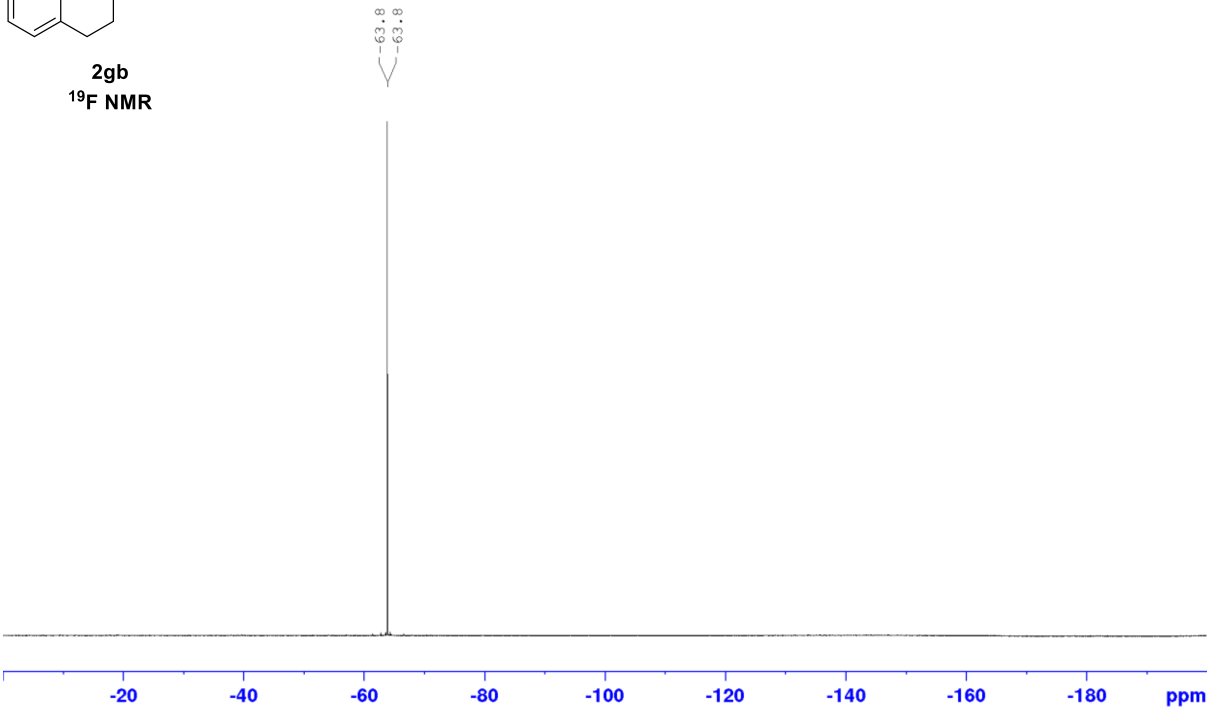

(1*S,2*S**)-*N*-(4-Iodophenyl)-2-(trifluoromethyl)-3,4-dihydro-2H,4'H-spiro[naphthalene-1,5'-thiazol]-2'-amine (2gc)**

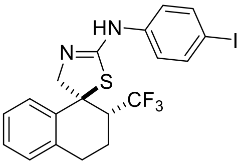

2gc
^{1}H NMR

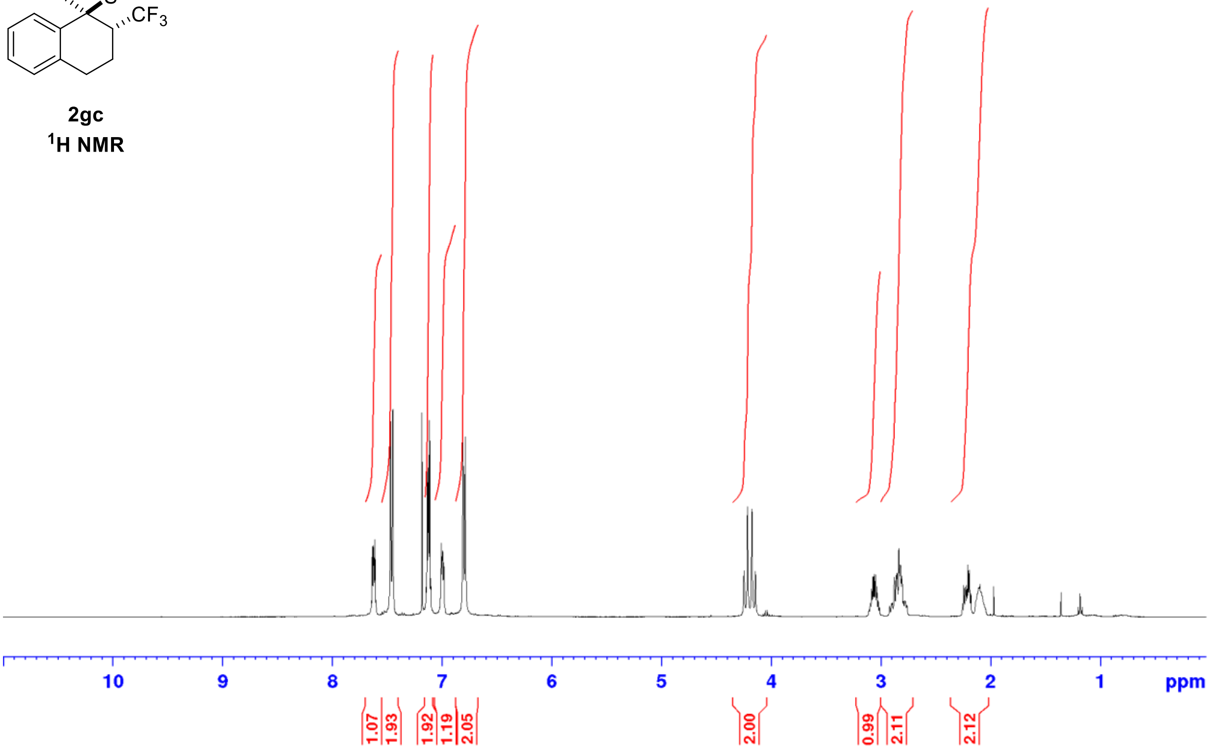

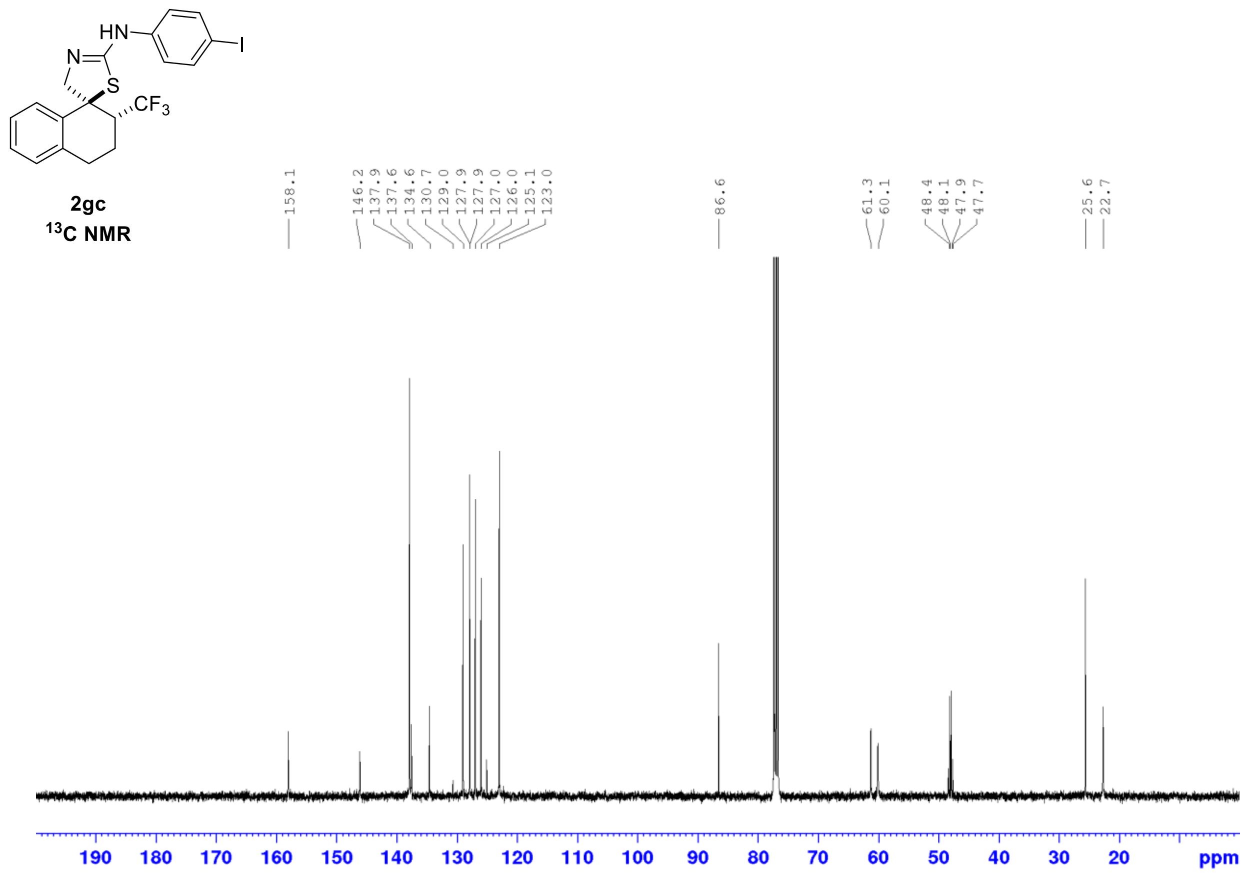

2gc

^{13}C NMR

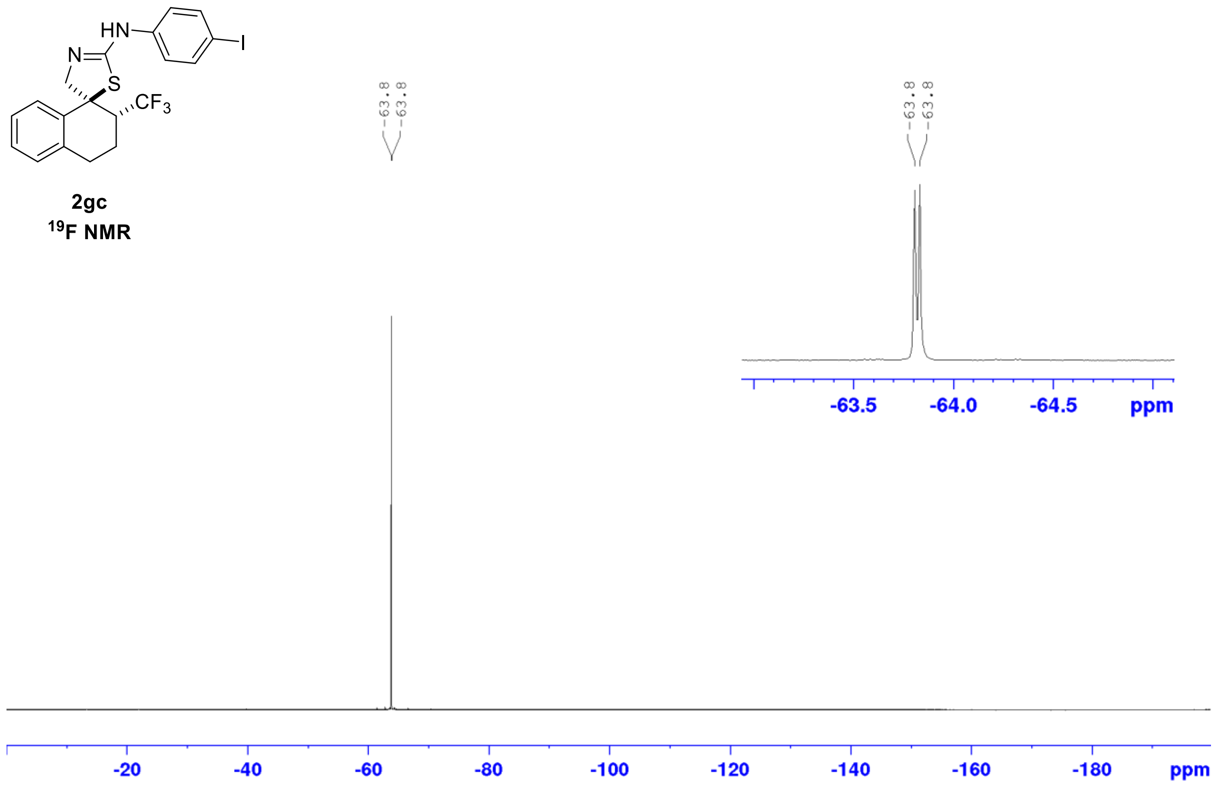

2gc

^{19}F NMR

(1S,2S)-N-(4-methoxyphenyl)-2-(trifluoromethyl)-3,4-dihydro-2H,4'H-spiro[naphthalene-1,5'-thiazol]-2'-amine (2gd)

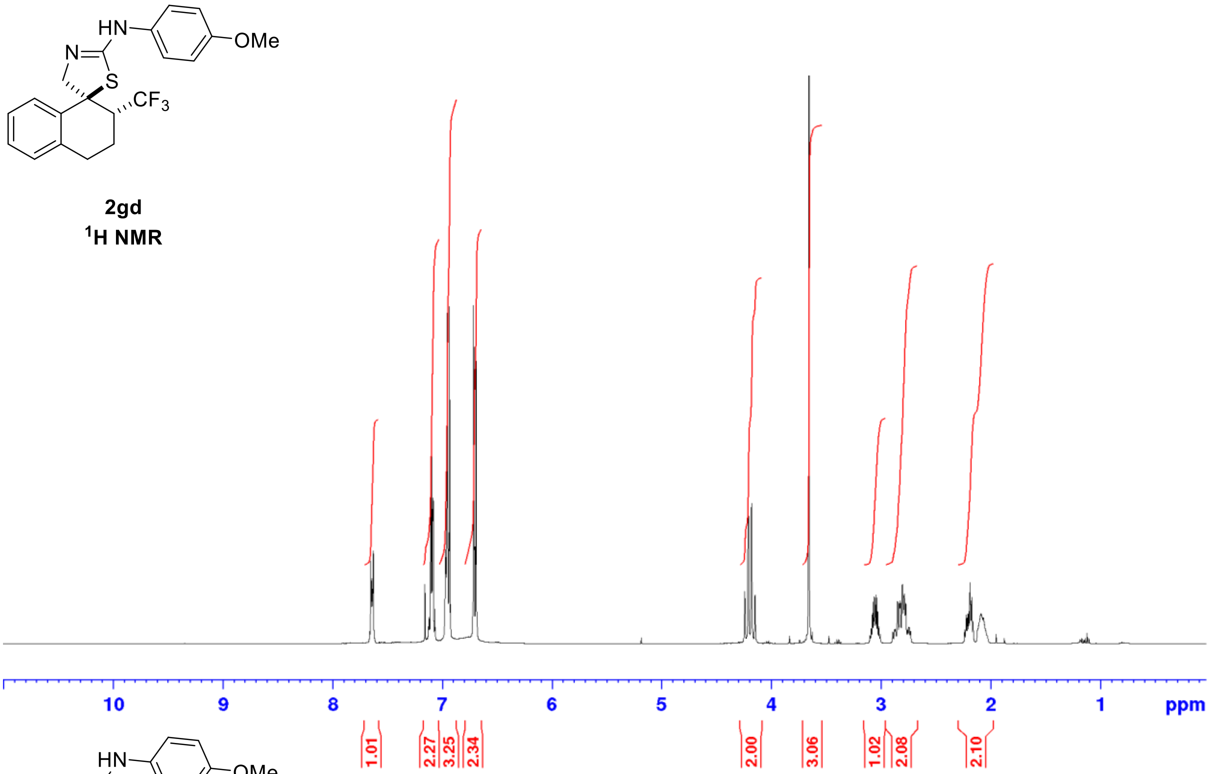

2gd
^{1}H NMR

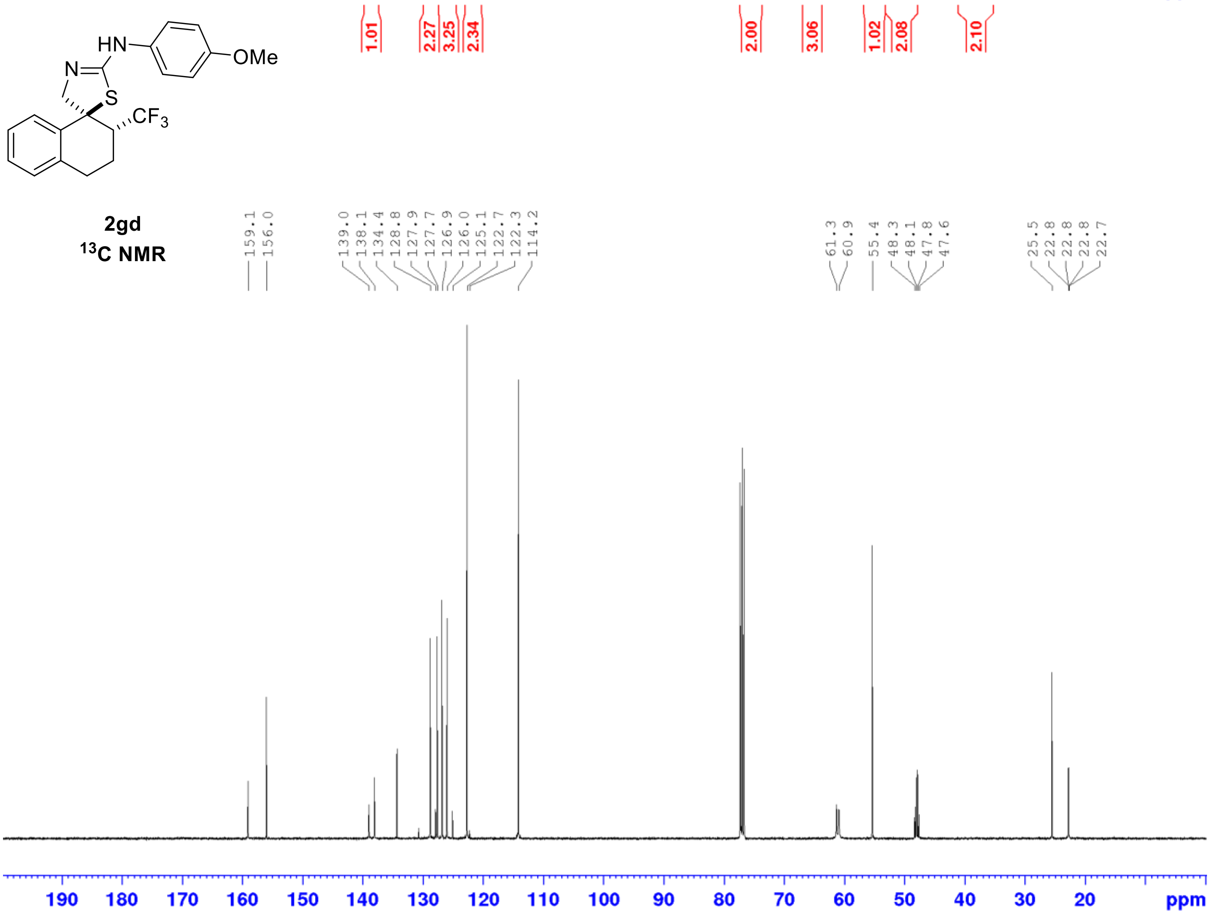

2gd
^{13}C NMR

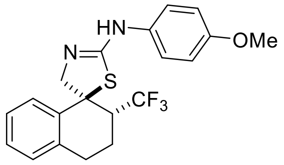

2gd
^{19}F NMR

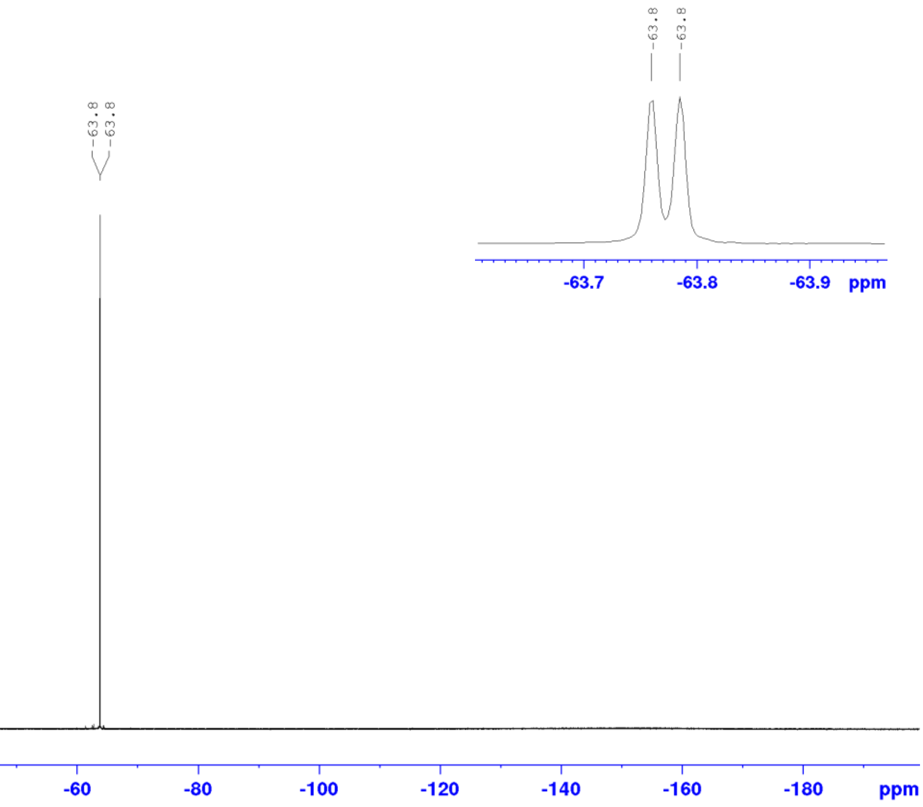

(1S*,2S*)-*N*-(naphthalen-2-yl)-2-(trifluoromethyl)-3,4-dihydro-2H,4'H-spiro[naphthalene-1,5'-thiazol]-2'-amine (2ge)

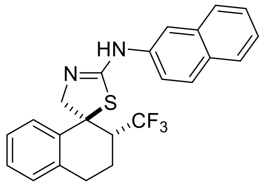

2ge
^{1}H NMR

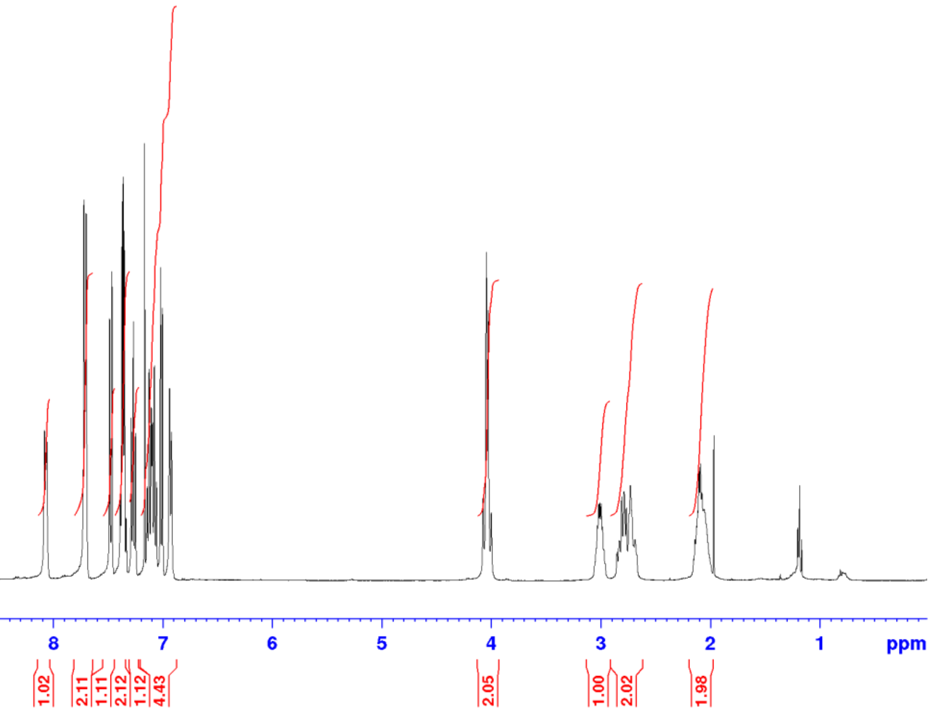

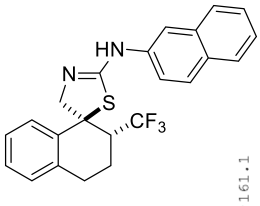
HN
N
S
CF3

2ge
^{13}C NMR

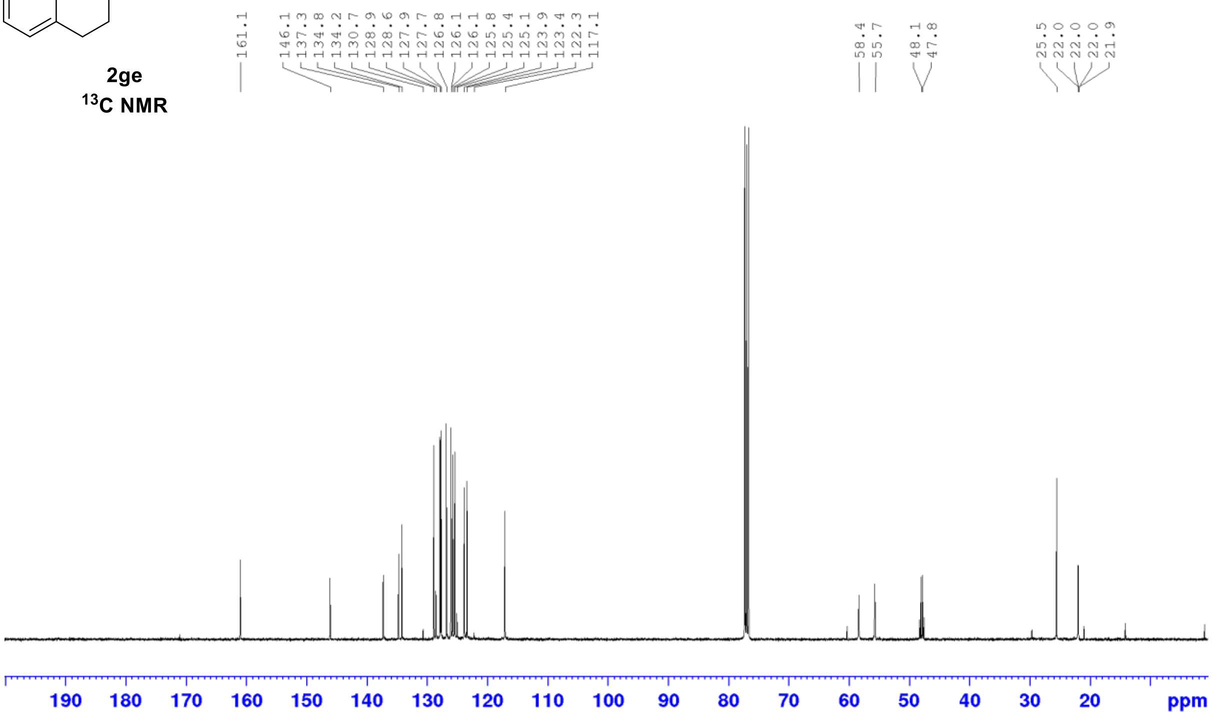
161.1
146.1
137.3
134.8
134.2
130.7
128.9
128.6
127.9
127.7
126.8
126.1
126.1
125.8
125.4
125.1
123.9
123.4
122.3
117.1
58.4
55.7
48.1
47.8
25.5
22.0
22.0
22.0
21.9
190
180
170
160
150
140
130
120
110
100
90
80
70
60
50
40
30
20
ppm

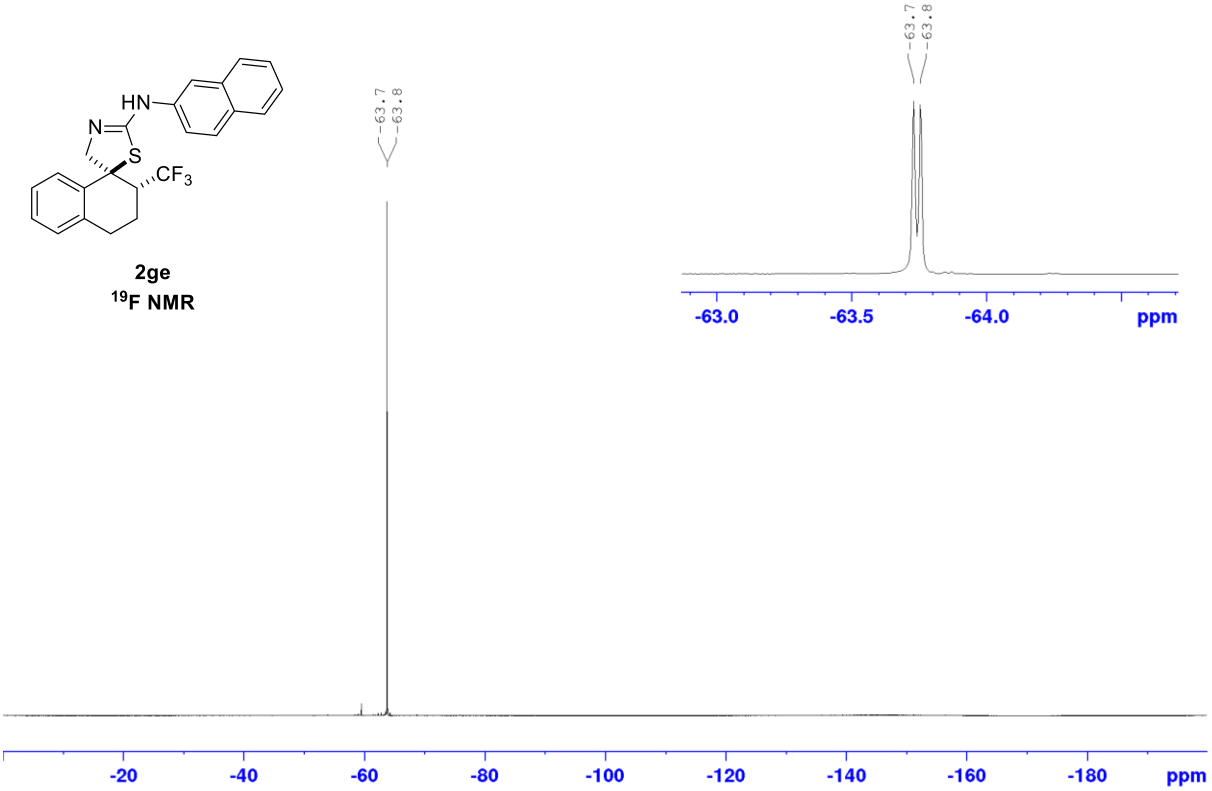
HN
N
S
CF3
2ge
19F NMR
-63.7
-63.8
-63.0
-63.5
-64.0
ppm
-20
-40
-60
-80
-100
-120
-140
-160
-180
ppm

(1S*,2S*)-2-(Trifluoromethyl)-*N*-(4-(trifluoromethyl)phenyl)-3,4-dihydro-2H,4'H-spiro[naphthalene-1,5'-thiazol]-2'-amine (2gf)

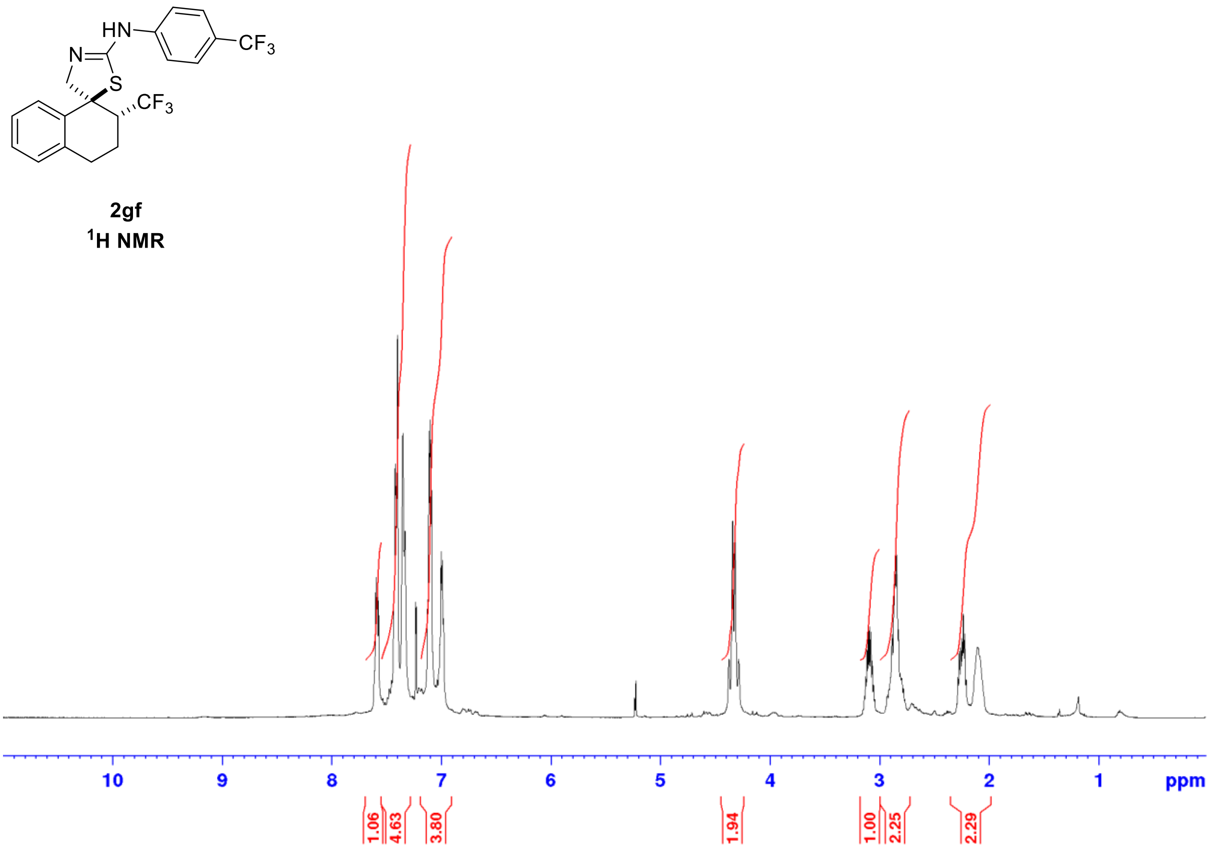

2gf
^{1}H NMR

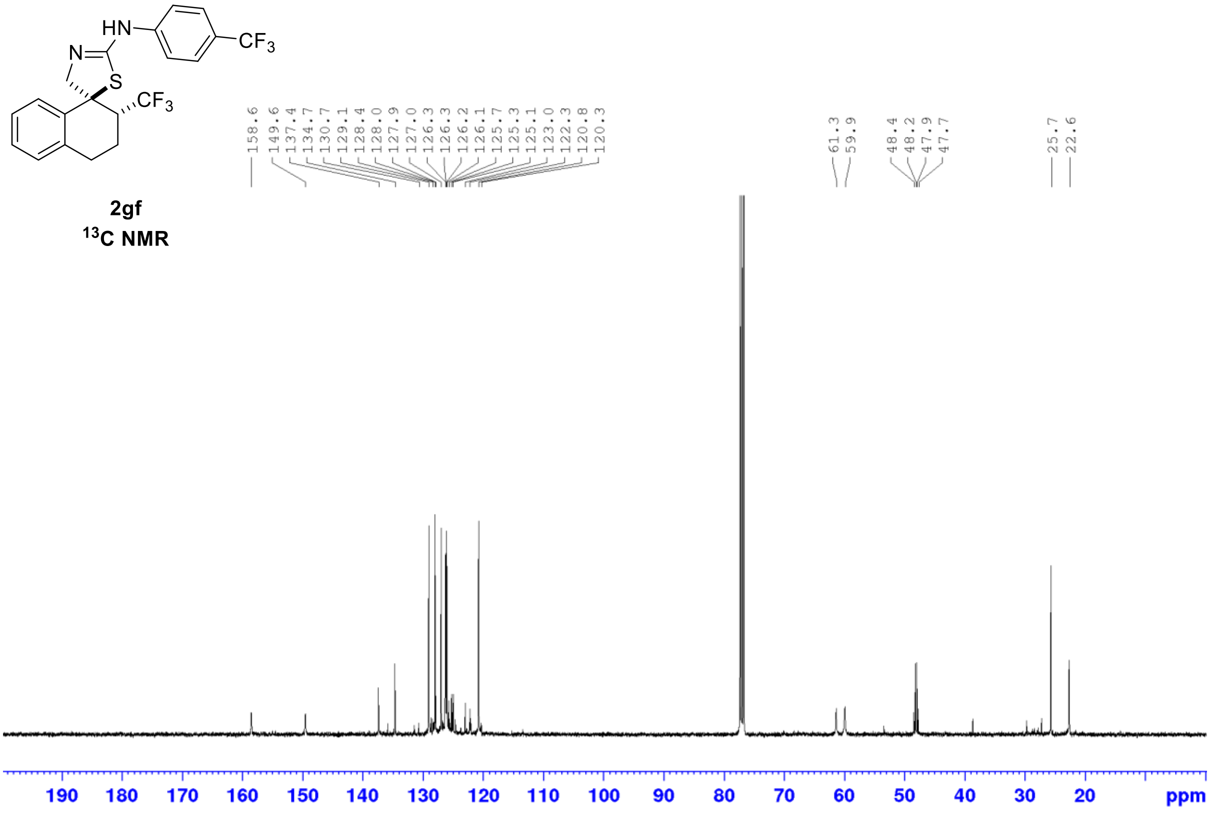

2gf
^{13}C NMR

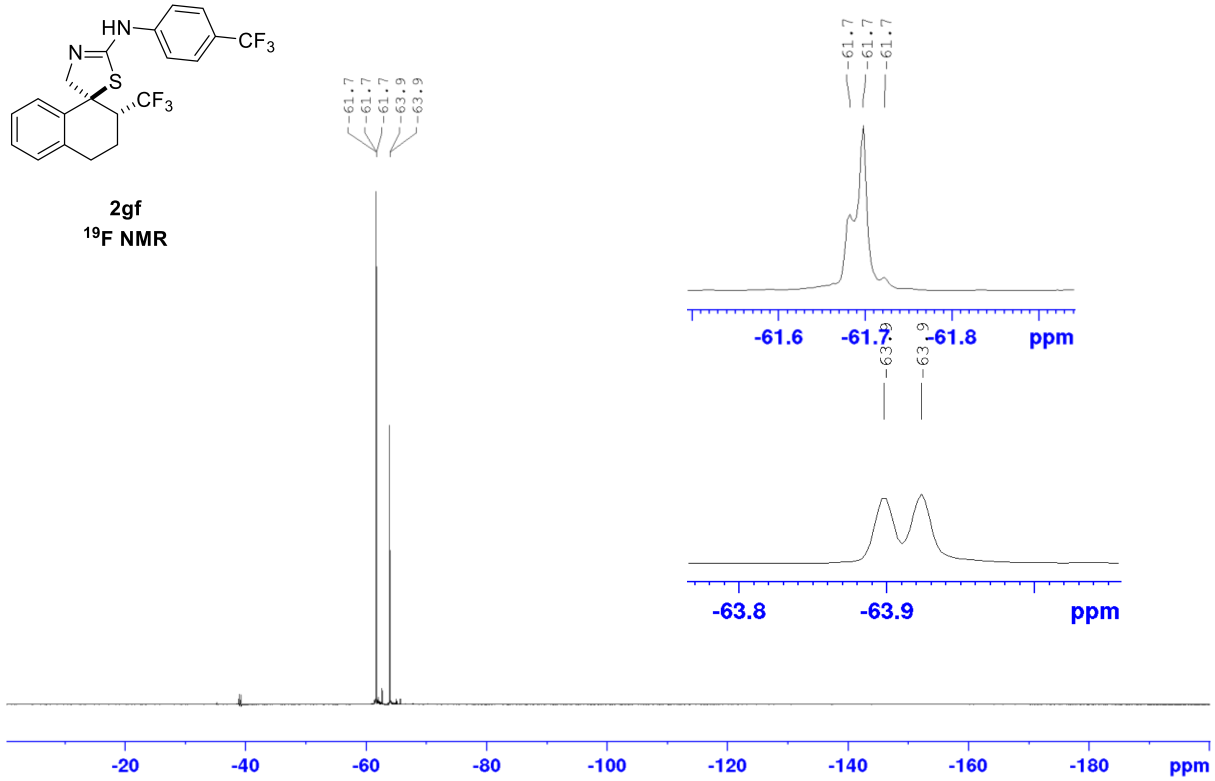

Methyl 4-(((1*S,2*S**)-2-(trifluoromethyl)-3,4-dihydro-2H,4'H-spiro[naphthalene-1,5'-thiazol]-2'-yl)amino)benzoate (2gg)**

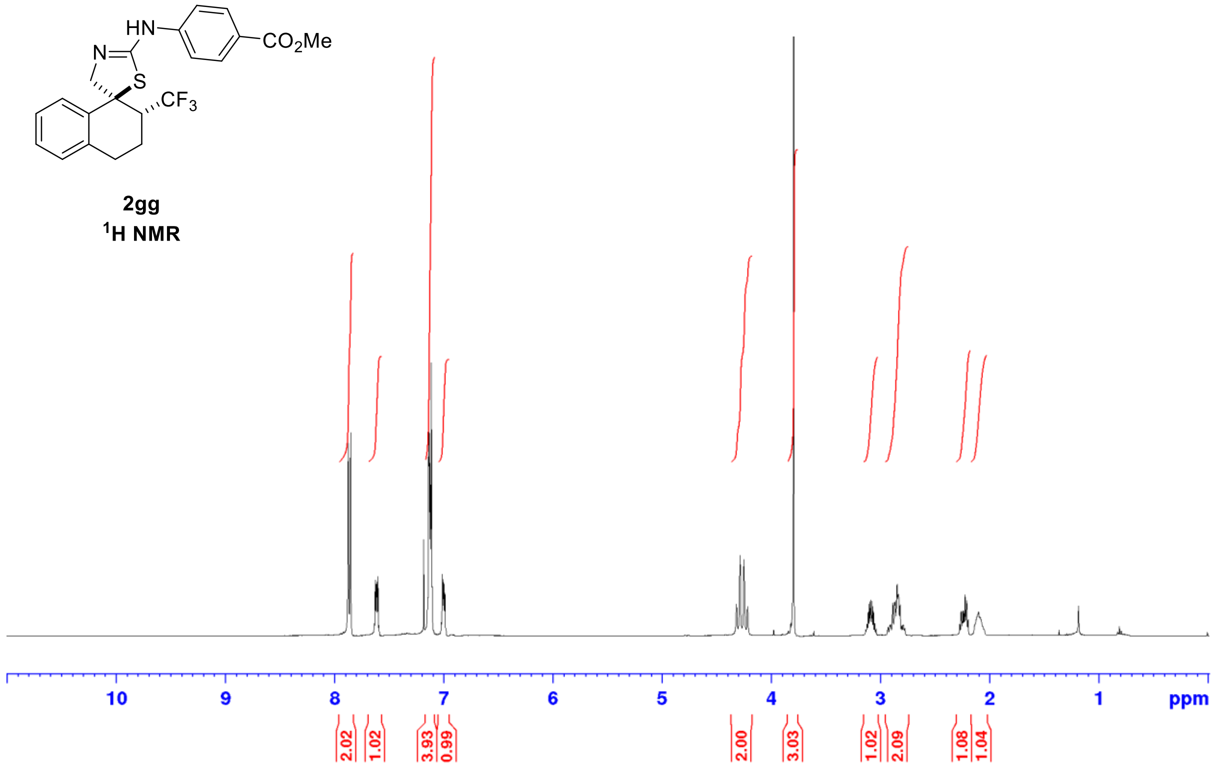

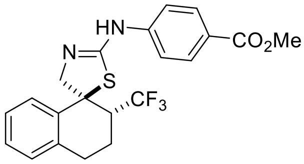

2gg
^{13}C NMR

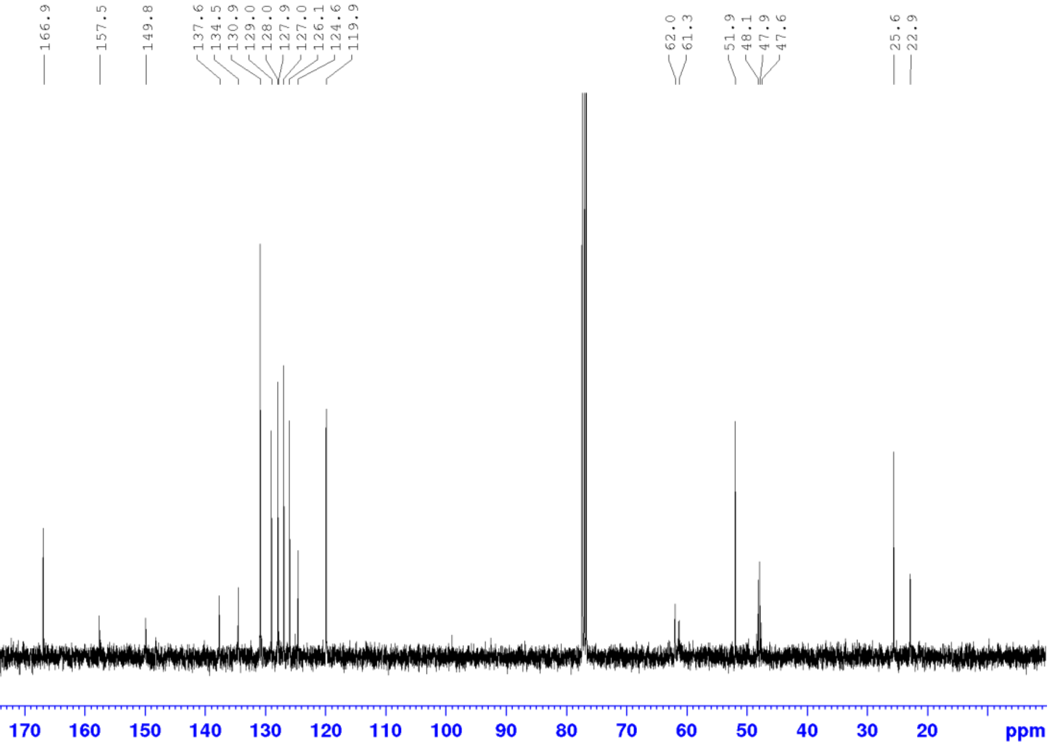

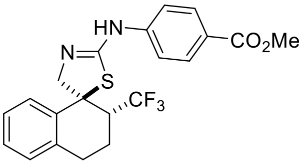

2gg
^{19}F NMR

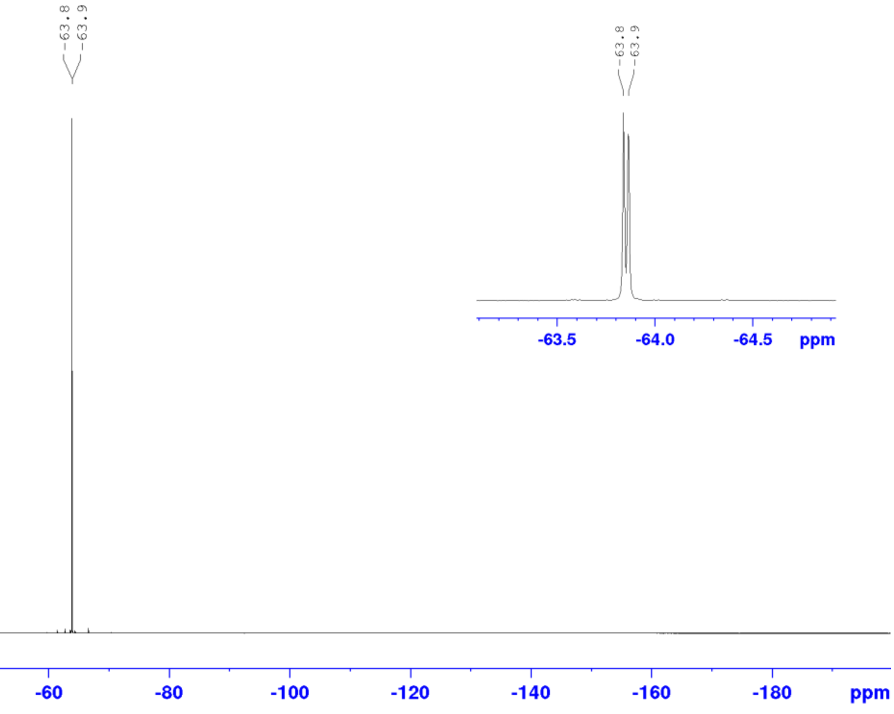

(1*S,2*S**)-*N*-Benzyl-2-(trifluoromethyl)-3,4-dihydro-2H,4'H-spiro[naphthalene-1,5'-thiazol]-2'-amine (2gh)**

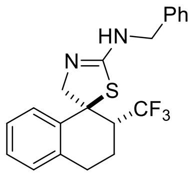

^{1}**H NMR**
2gh

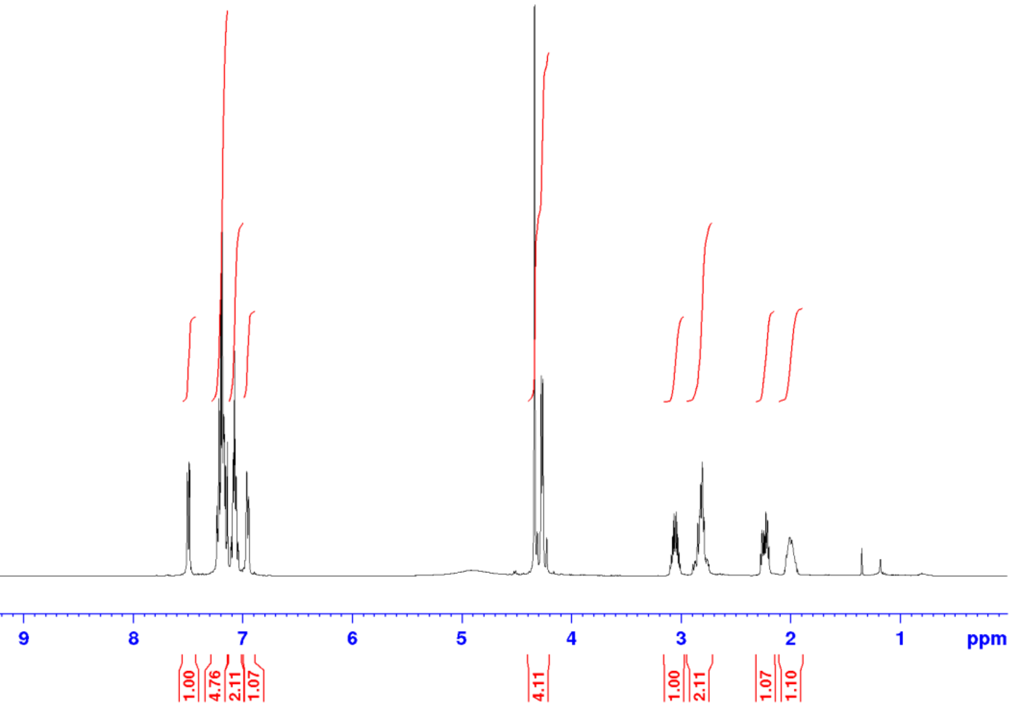

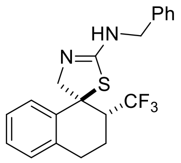

^{13}**C NMR**
2gh

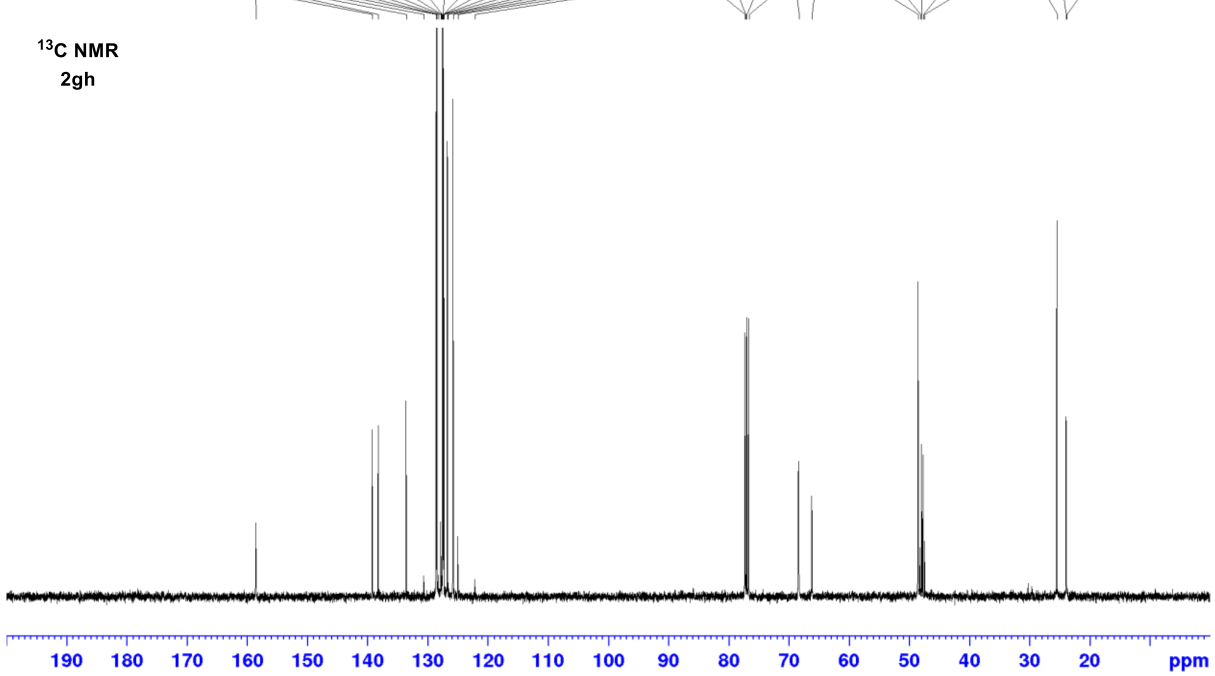

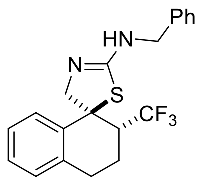

^{19}F NMR
2gh

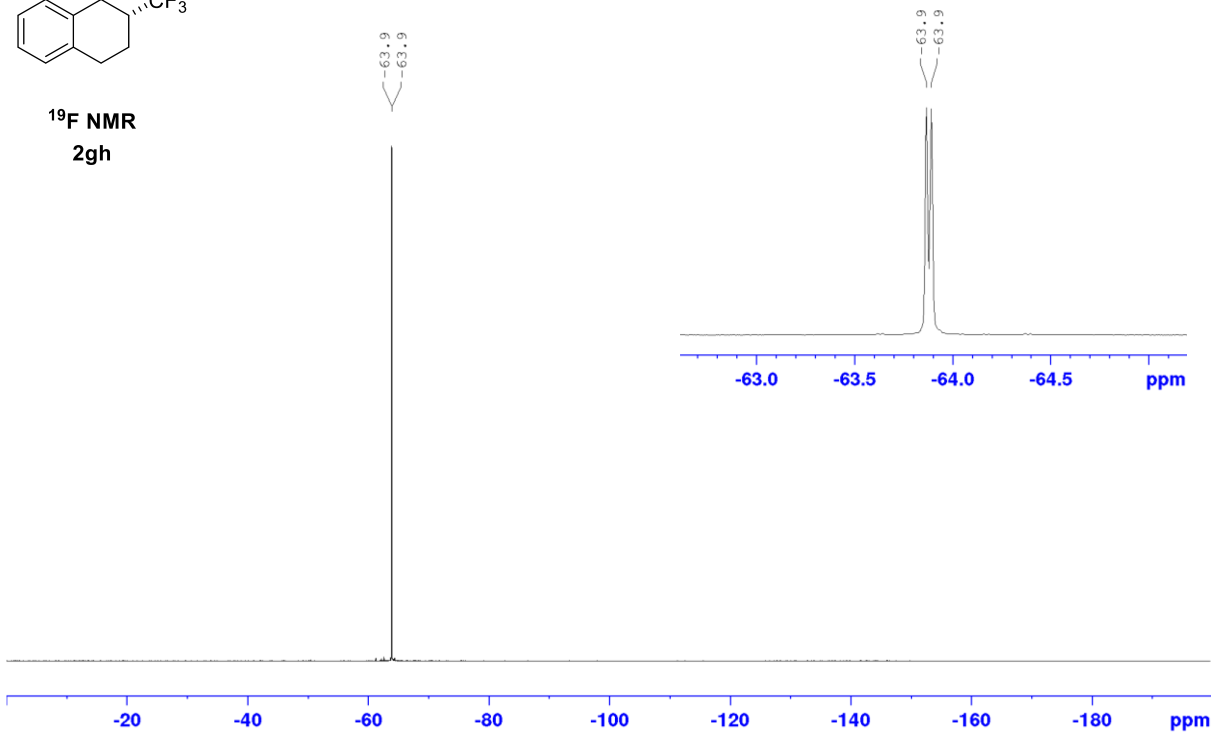

(1*S,2*S**)-*N*-(*tert*-butyl)-2-(trifluoromethyl)-3,4-dihydro-2H,4'H-spiro[naphthalene-1,5'-thiazol]-2'-amine (2gi)**

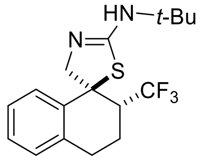

2gi
^{1}H NMR

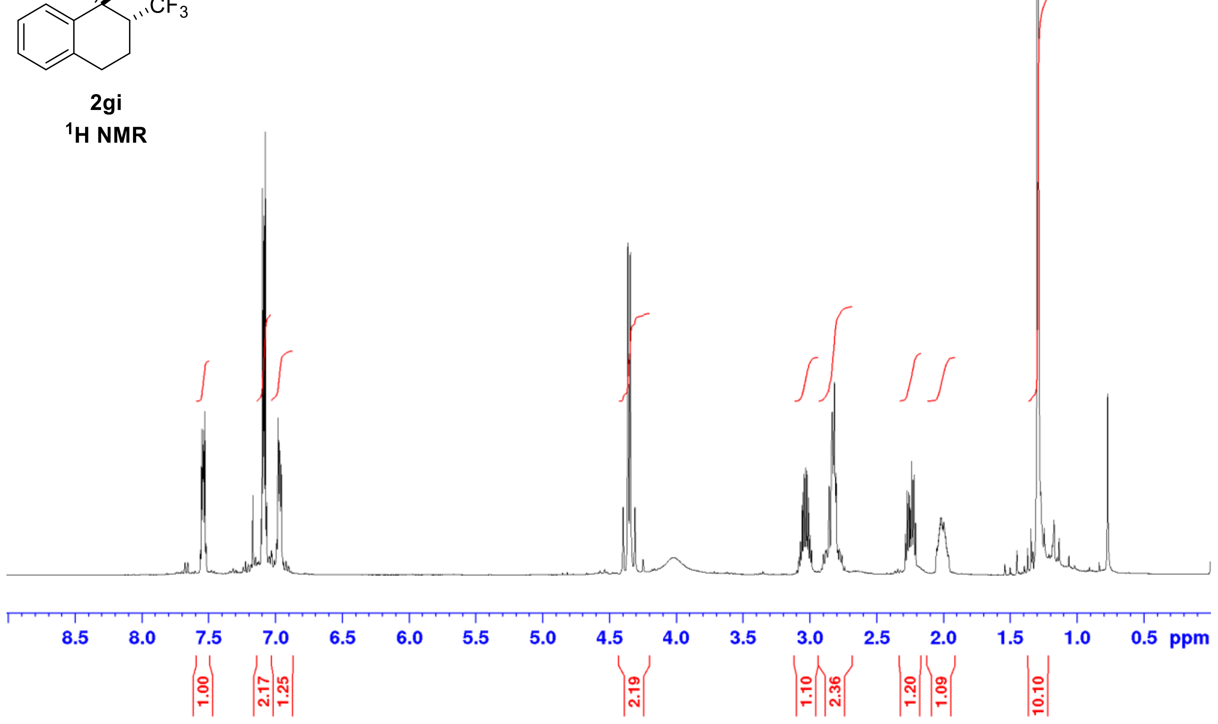

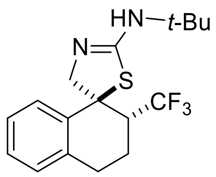

2gi
^{13}C NMR

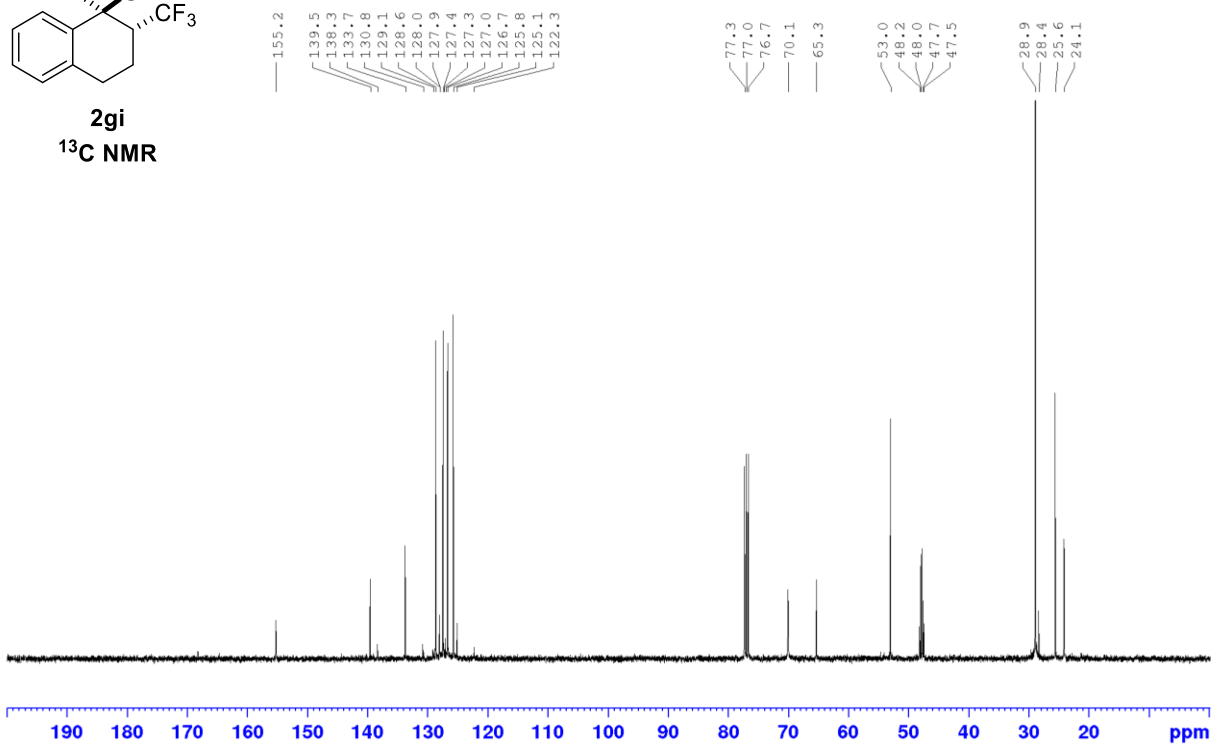

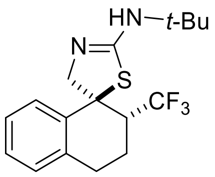

2gi
^{19}F NMR

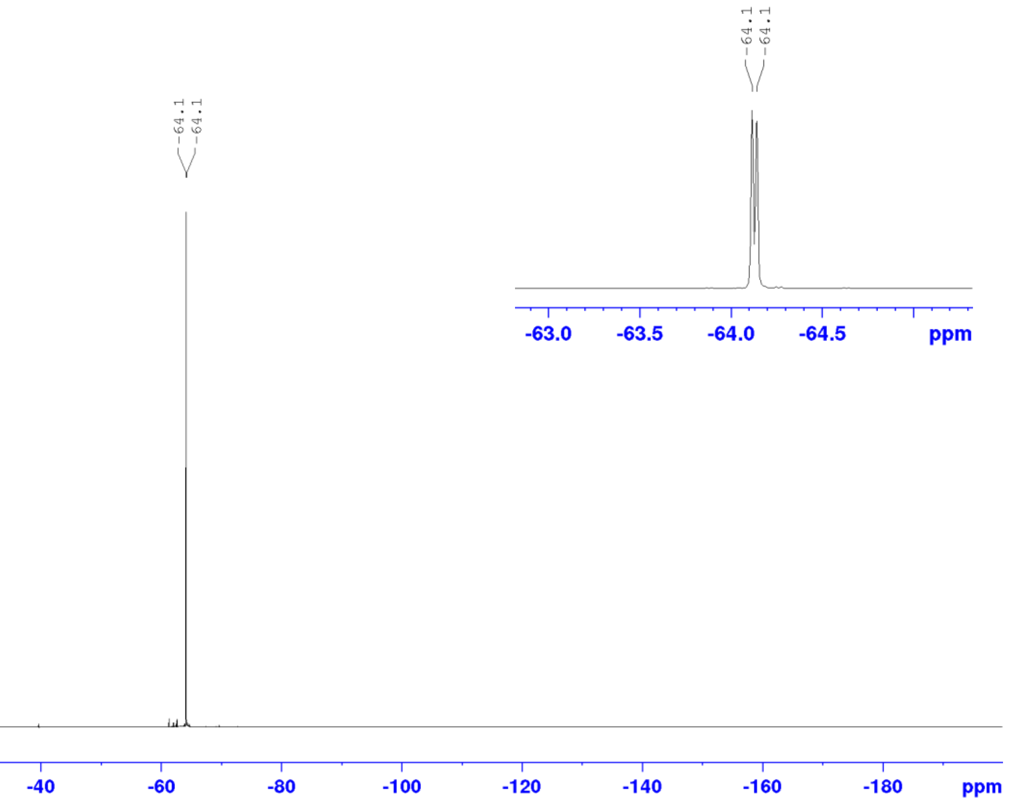

(1*S,2*S**)-*N*-(Benzo[d]thiazol-2-yl)-2-(trifluoromethyl)-3,4-dihydro-2H,4'H-spiro[naphthalene-1,5'-thiazol]-2'-amine (2gj)**

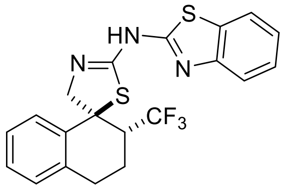

2gj
^{1}H NMR

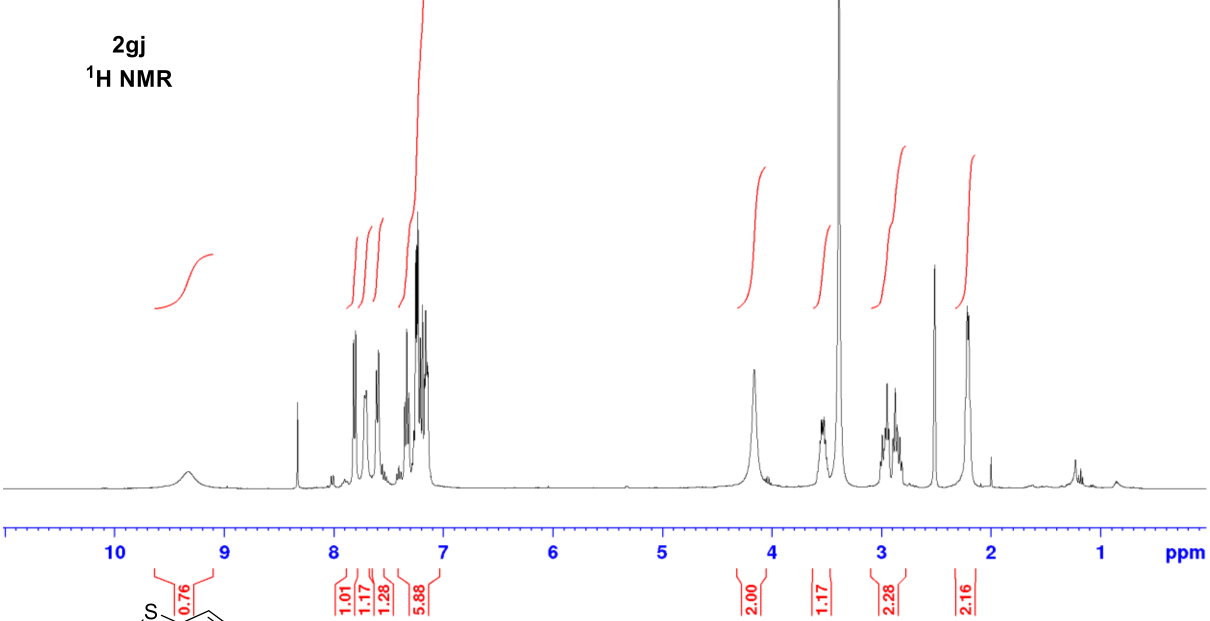

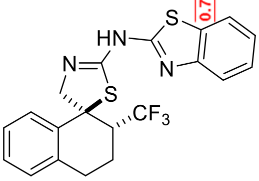

2gj
^{13}C NMR

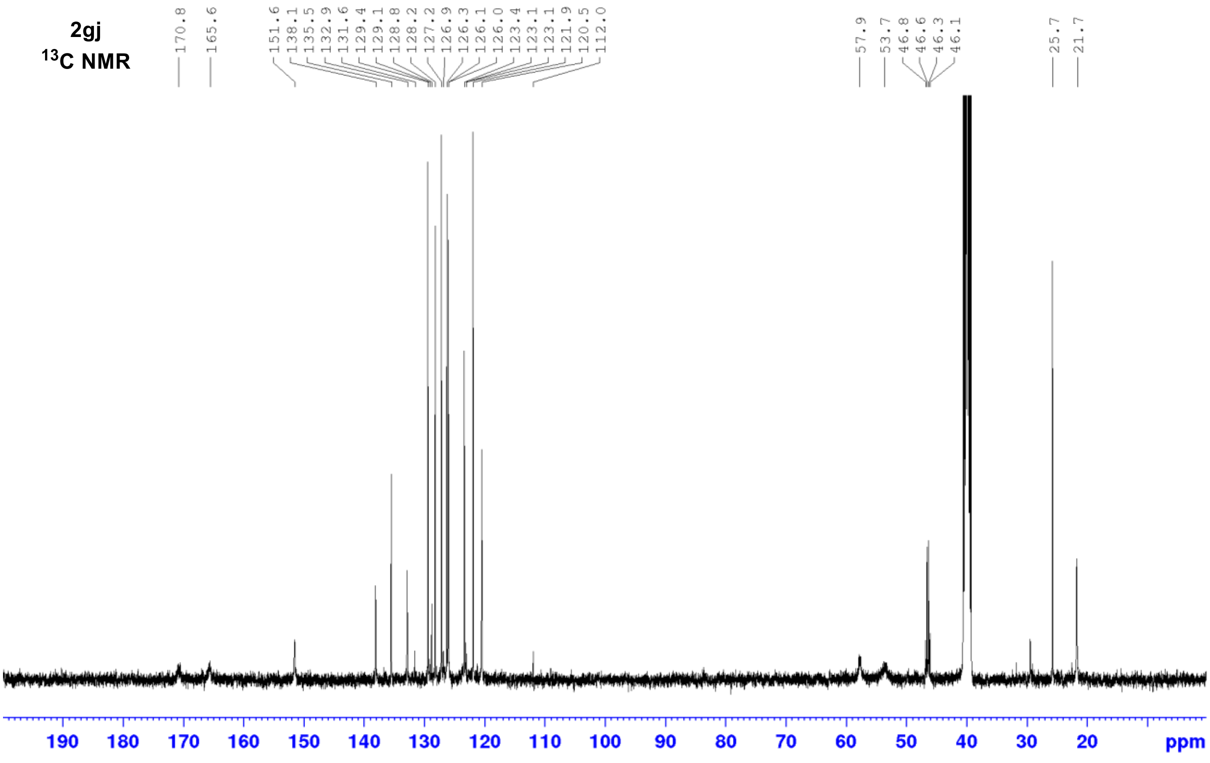

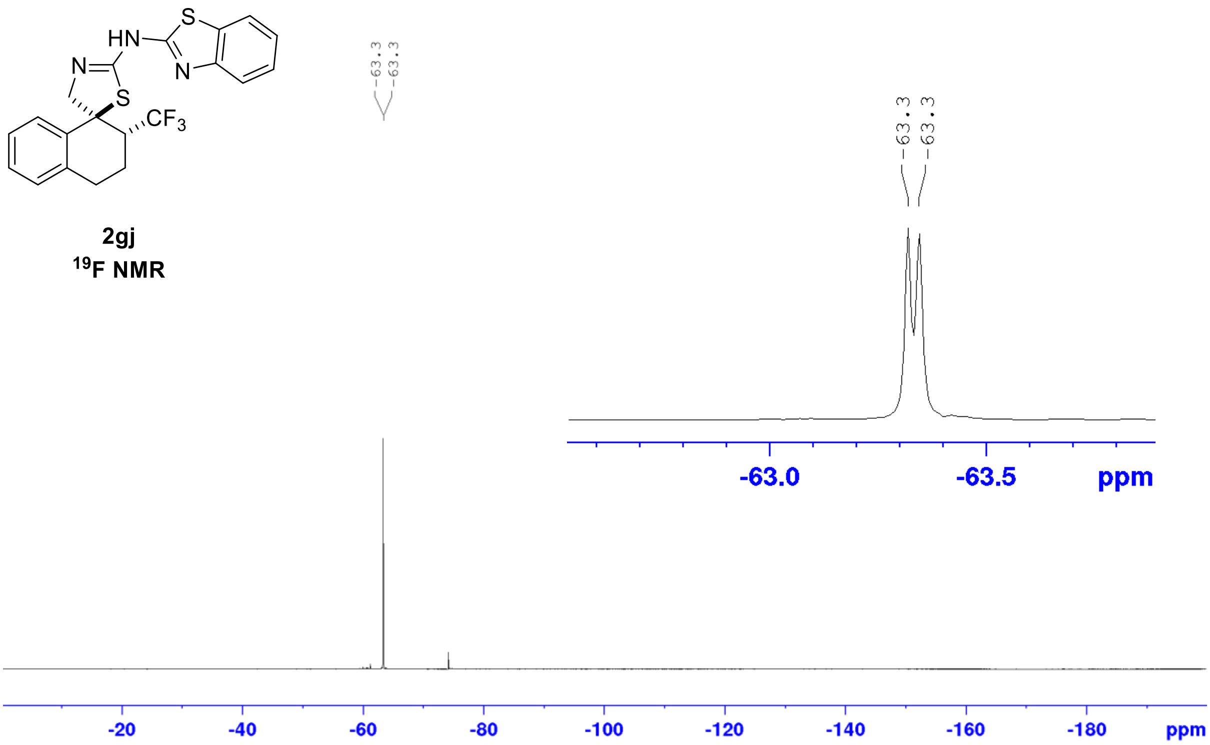

(1*S,2*S**)-*N*-(6-Methylpyridin-3-yl)-2-(trifluoromethyl)-3,4-dihydro-2H,4'H-spiro[naphthalene-1,5'-thiazol]-2'-amine (2gk)**

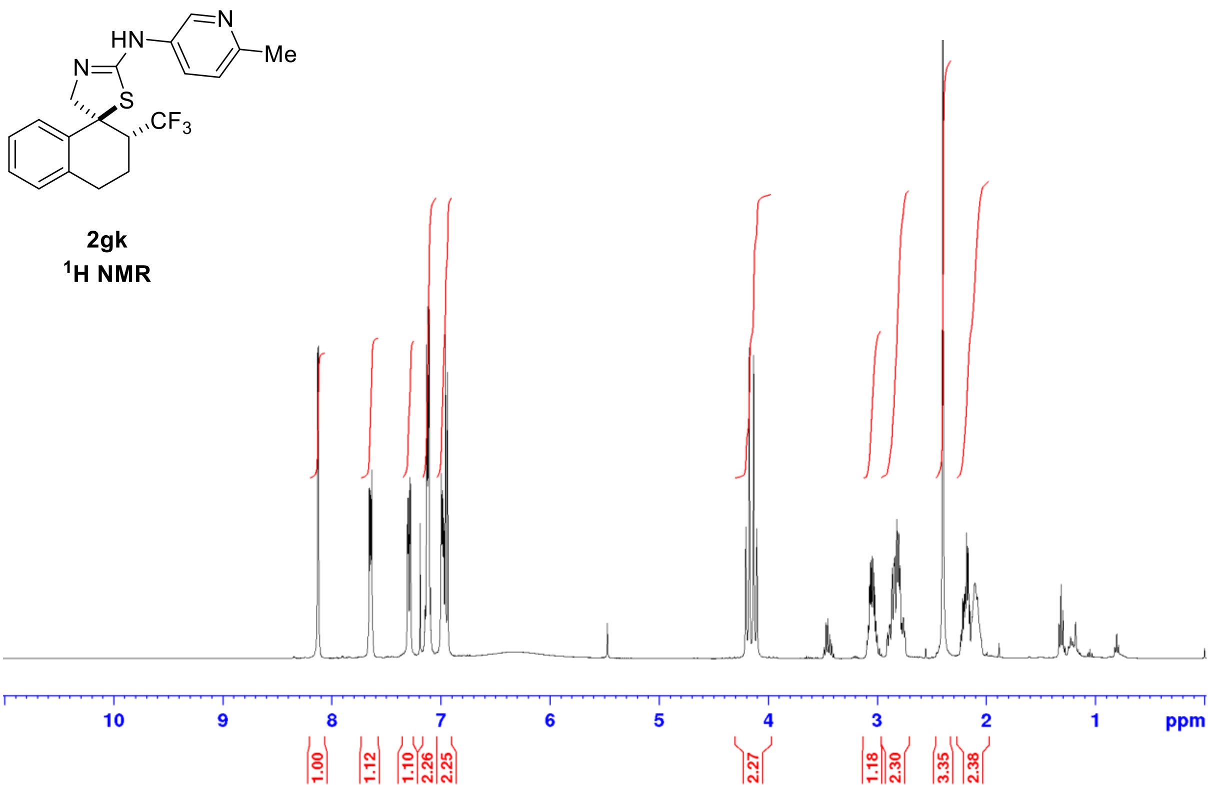

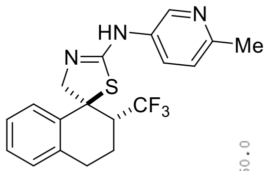

2gk
^{13}C NMR

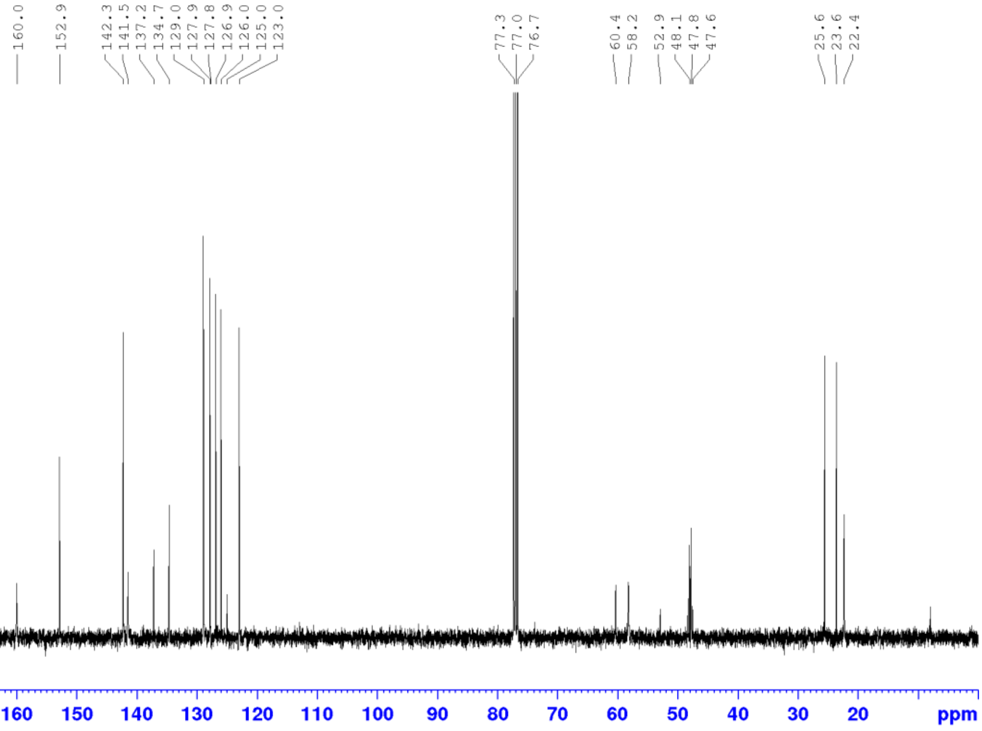

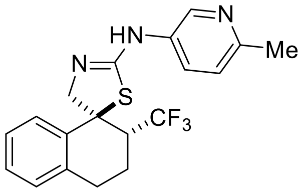

2gk
^{19}F NMR

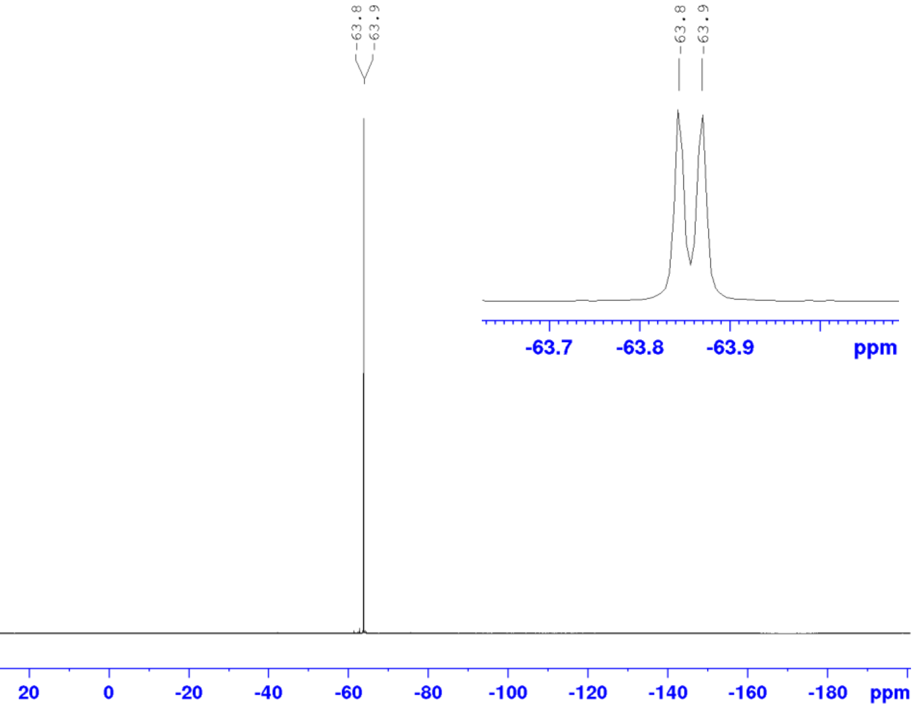

(1*S,2*S**)-*N*-Methyl-*N*-phenyl-2-(trifluoromethyl)-3,4-dihydro-2H,4'H-spiro[naphthalene-1,5'-thiazol]-2'-amine (2gl)**

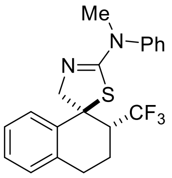

2gl
^{1}H NMR

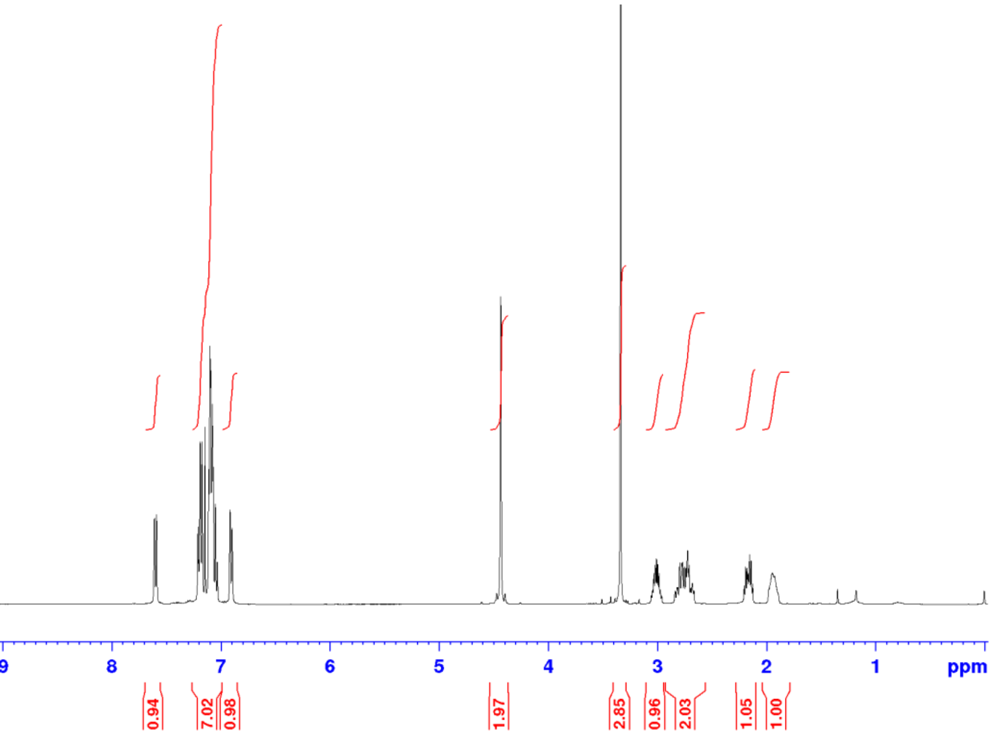

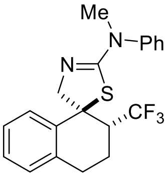

2gl
^{13}C NMR

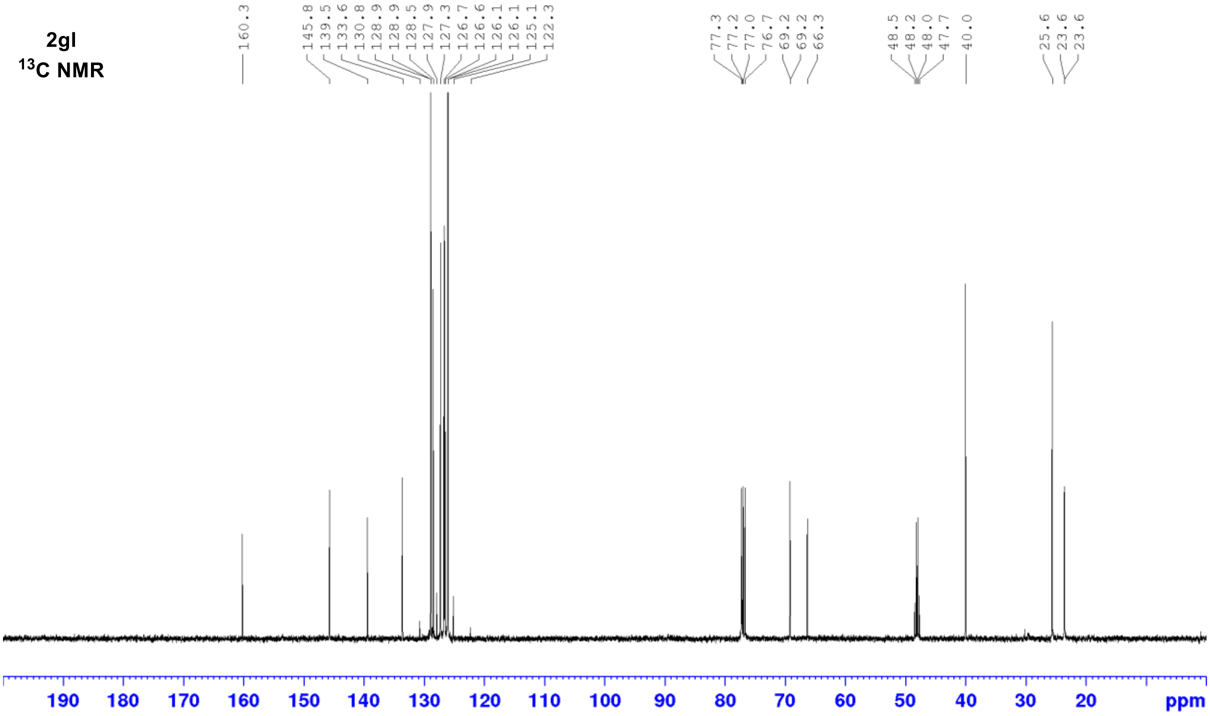

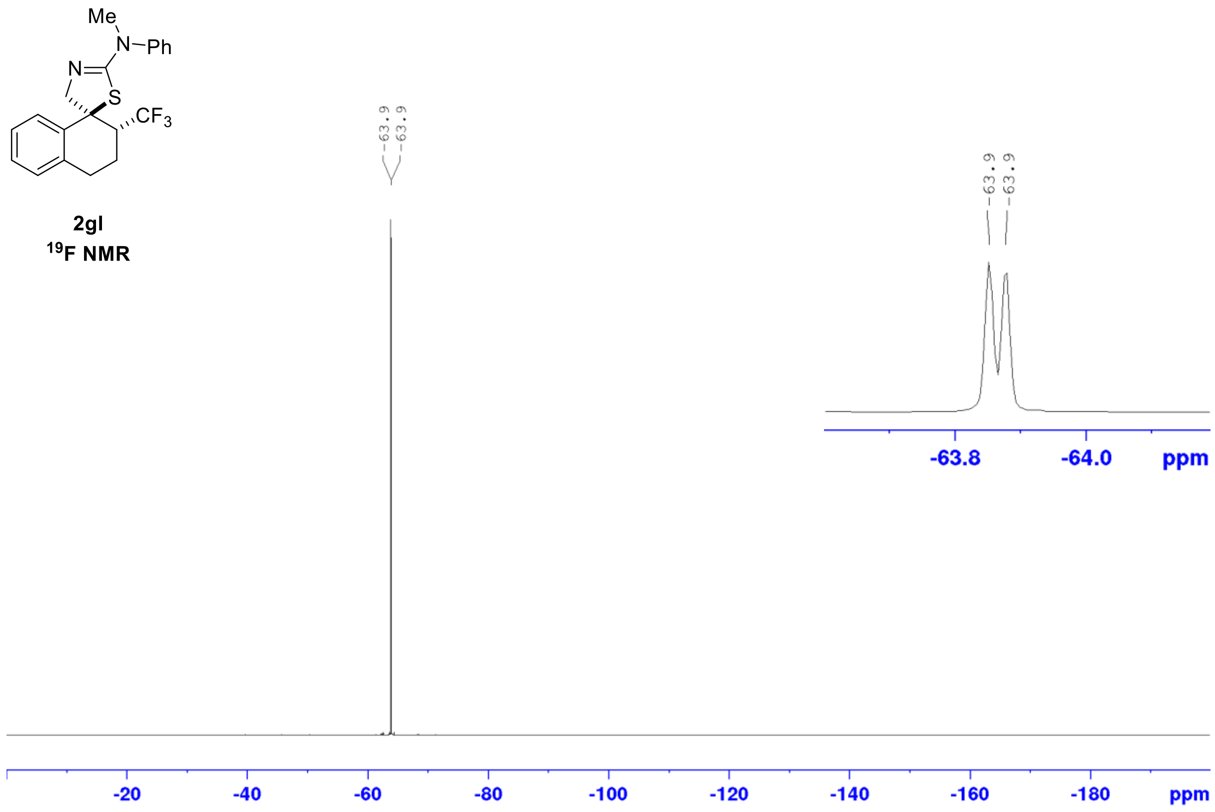

(1*S,2*S**)-2-(Trifluoromethyl)-3,4-dihydro-2H,4'H-spiro[naphthalene-1,5'-thiazol]-2'-amine (2gm)**

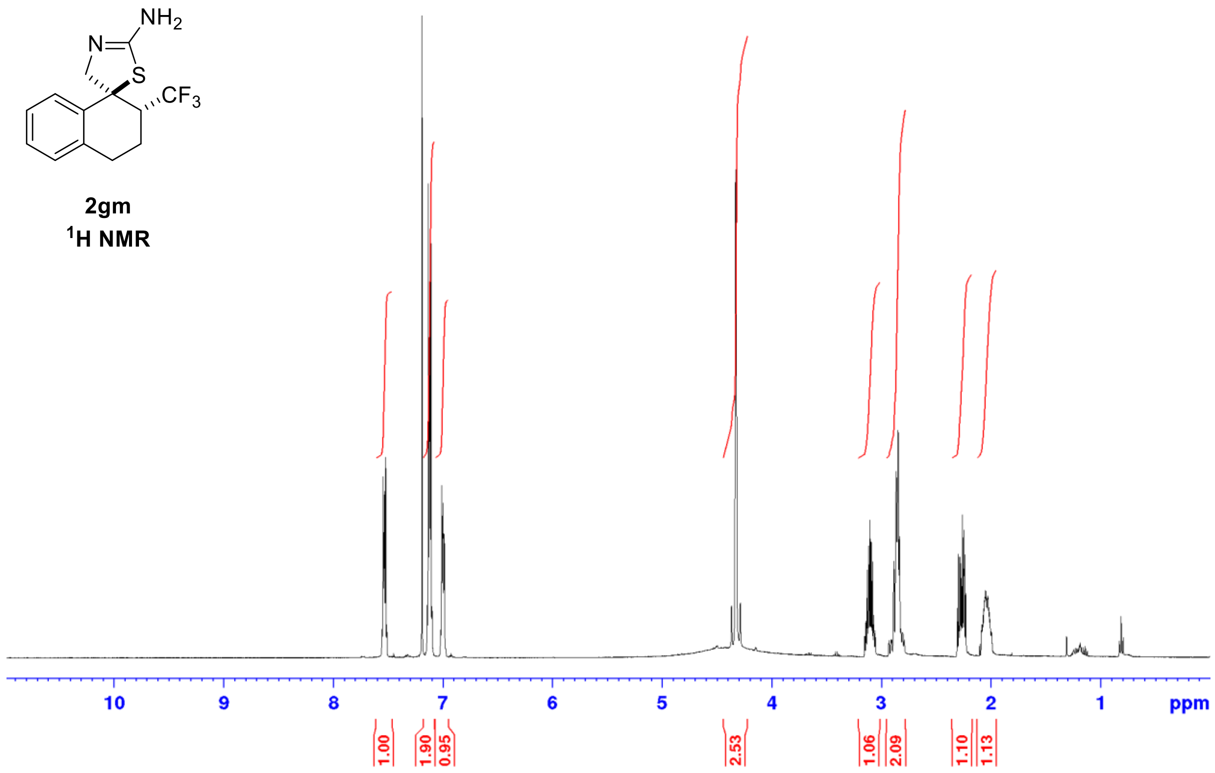

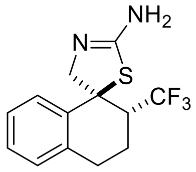

2gm
^{13}C NMR

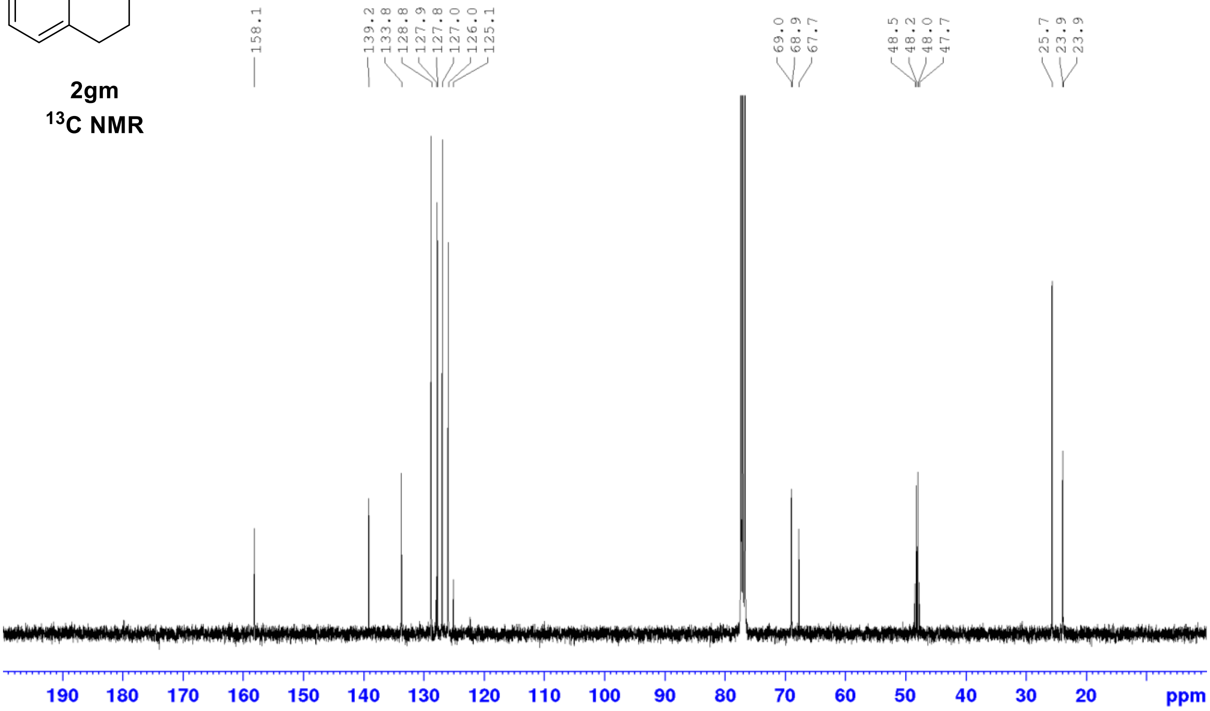

4'-Ethyl-N-phenyl-3,4-dihydro-2H,4'H-spiro[naphthalene-1,5'-thiazol]-2'-amine (2gn)

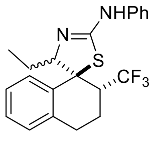

2gn

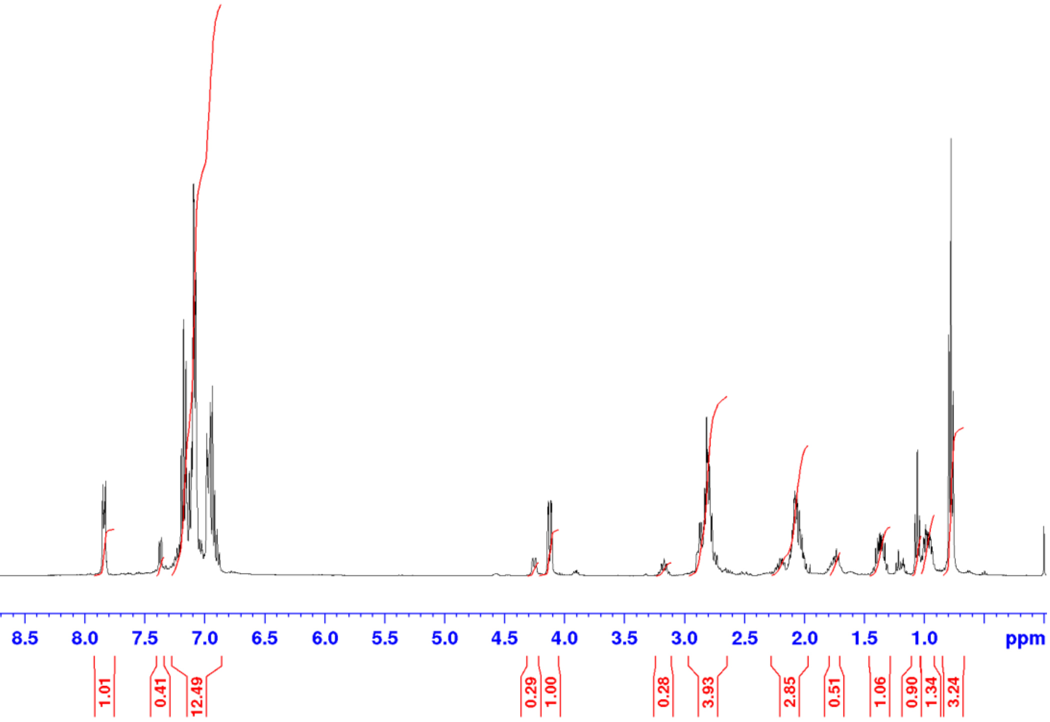

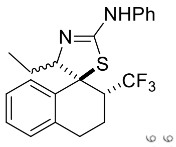
NHPh
N
S
CF3

2gn

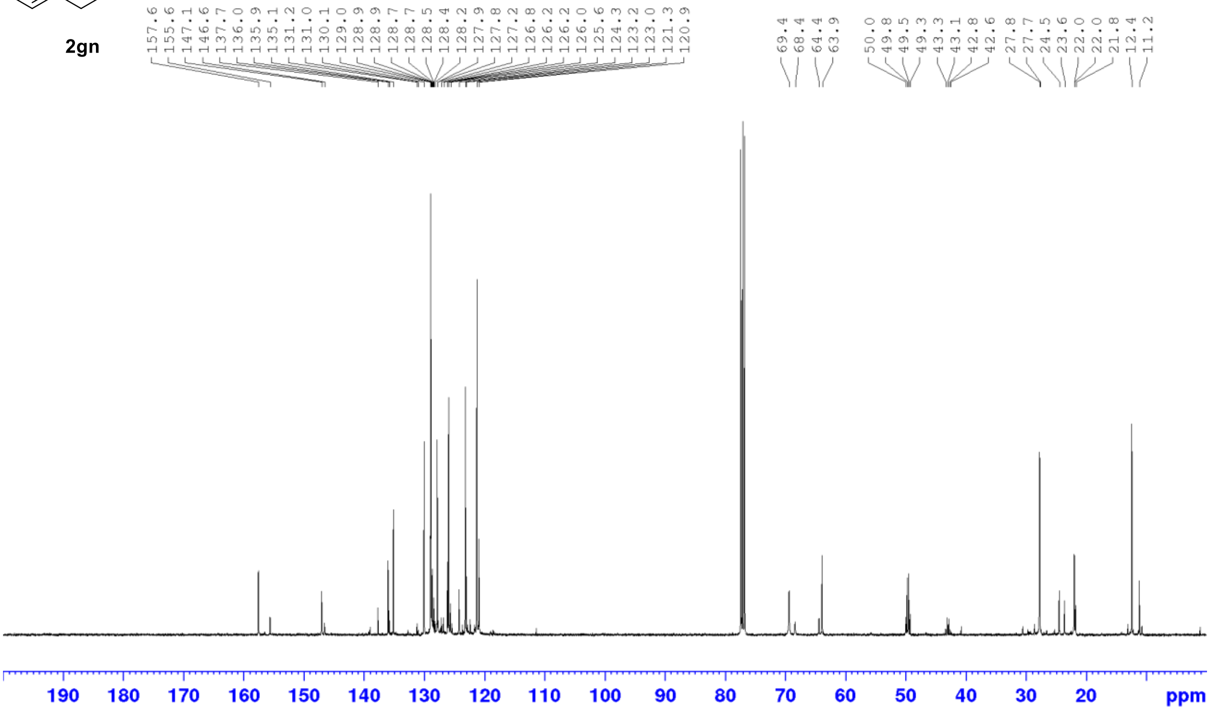
157.6
155.6
147.1
146.6
137.7
136.0
135.9
135.1
131.2
131.0
130.1
129.0
128.9
128.9
128.7
128.7
128.5
128.4
128.2
127.9
127.8
127.2
126.8
126.2
126.2
126.0
125.6
124.3
123.2
123.0
121.3
120.9
69.4
68.4
64.4
63.9
50.0
49.8
49.5
49.3
43.3
43.1
42.8
42.6
27.8
27.7
24.5
23.6
22.0
22.0
21.8
12.4
11.2
190 180 170 160 150 140 130 120 110 100 90 80 70 60 50 40 30 20 ppm

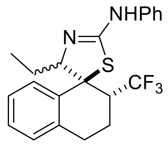
NHPh
N
S
CF3

2gn

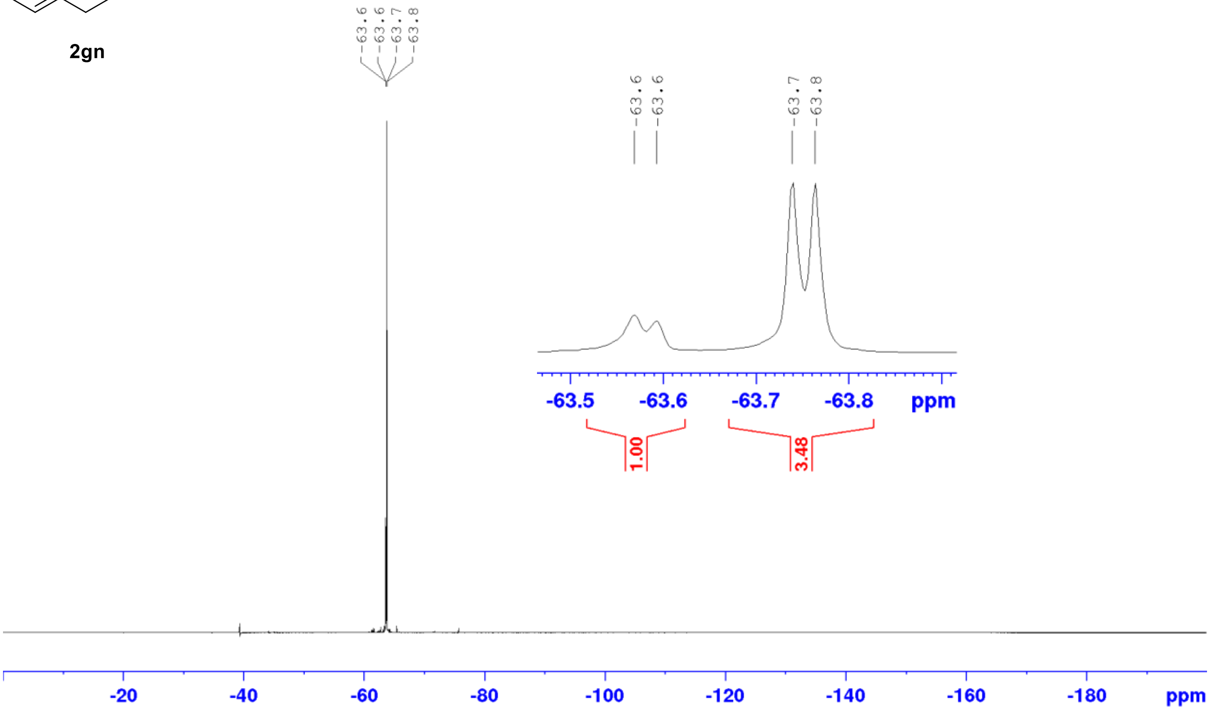
-63.6
-63.6
-63.7
-63.8
-63.6
-63.6
-63.7
-63.8
-63.5 -63.6 -63.7 -63.8 ppm
1.00
3.48
-20 -40 -60 -80 -100 -120 -140 -160 -180 ppm

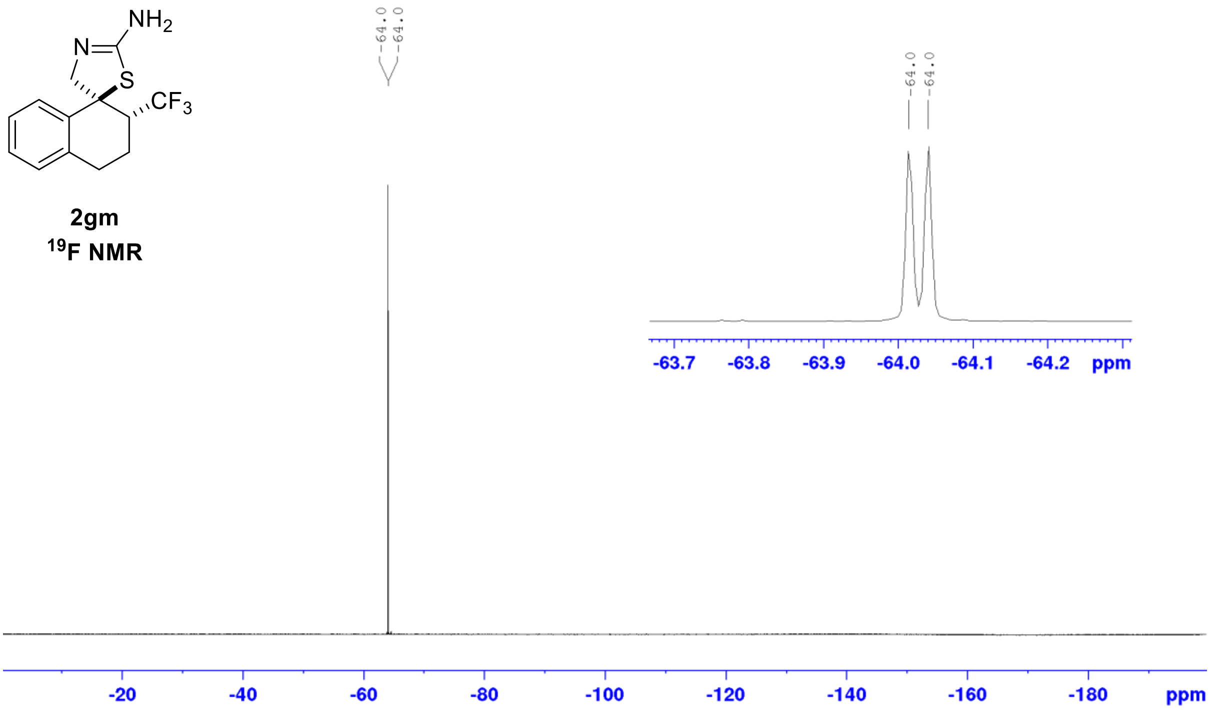

(3S*,4S*)-*N*-Phenyl-3-(trifluoromethyl)-4'H-spiro[chromane-4,5'-thiazol]-2'-amine (2ha)

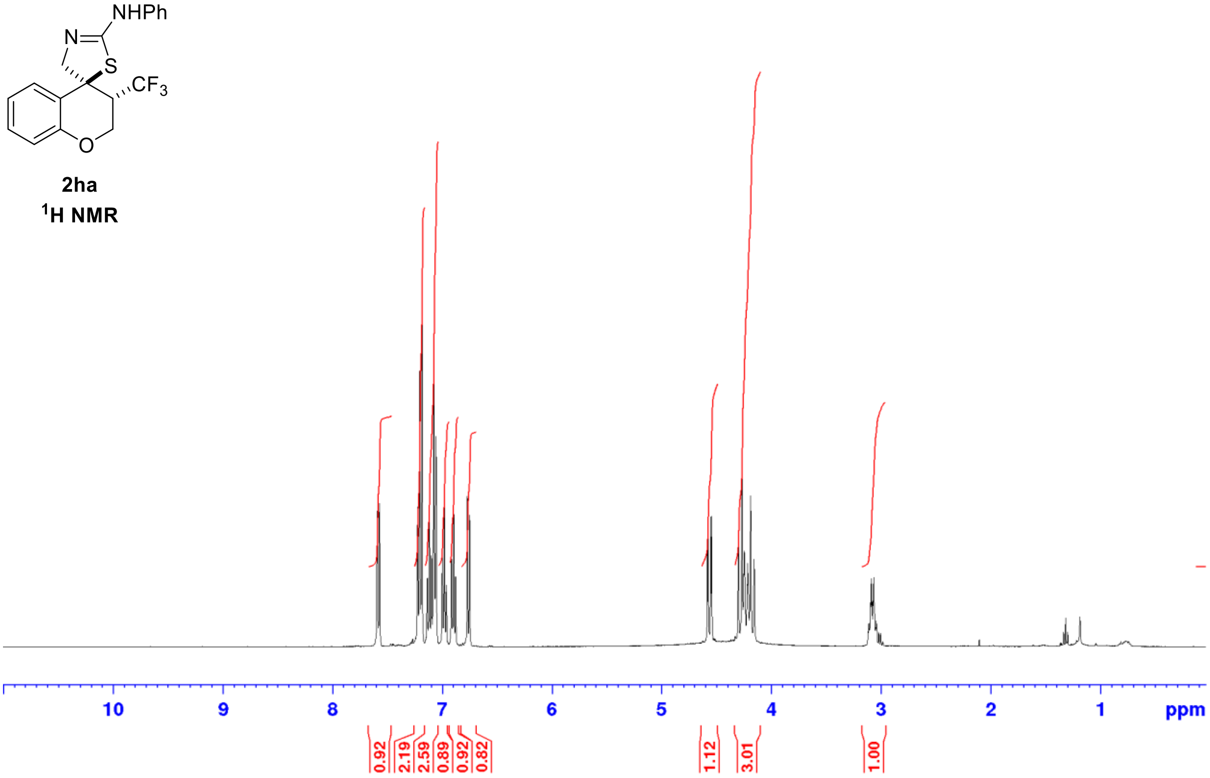

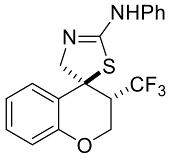

2ha
^{13}C NMR

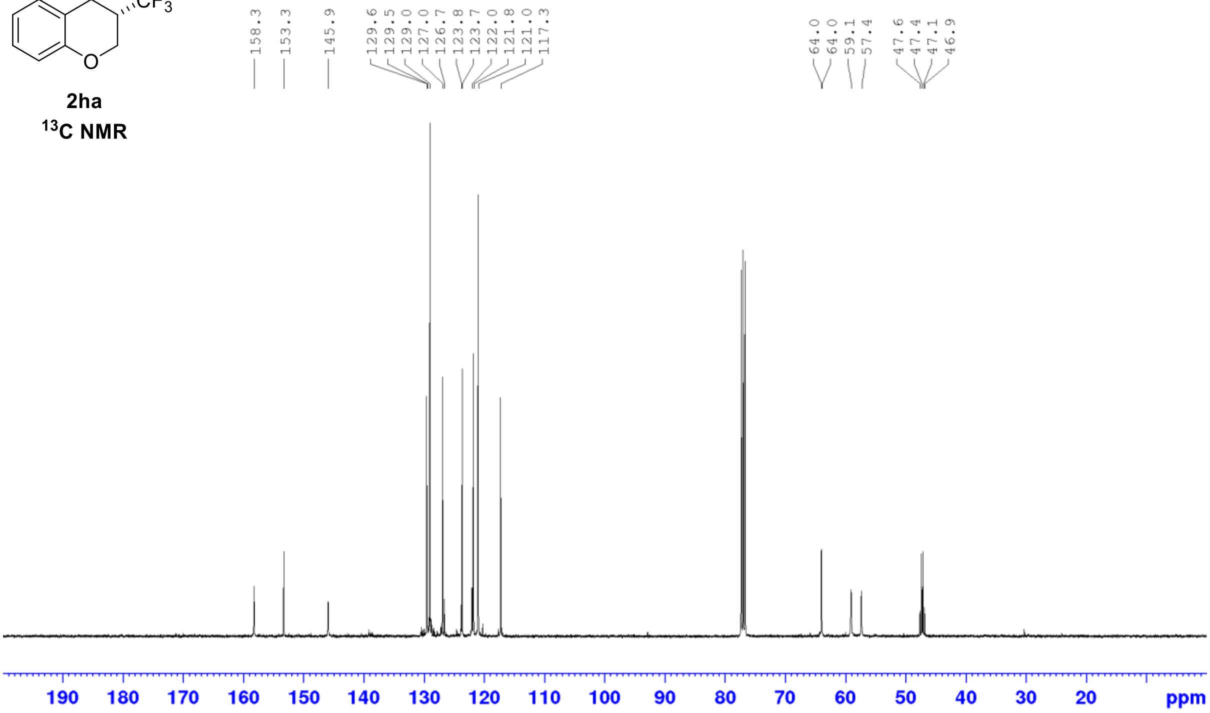

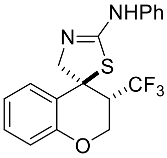

2ha
^{19}F NMR

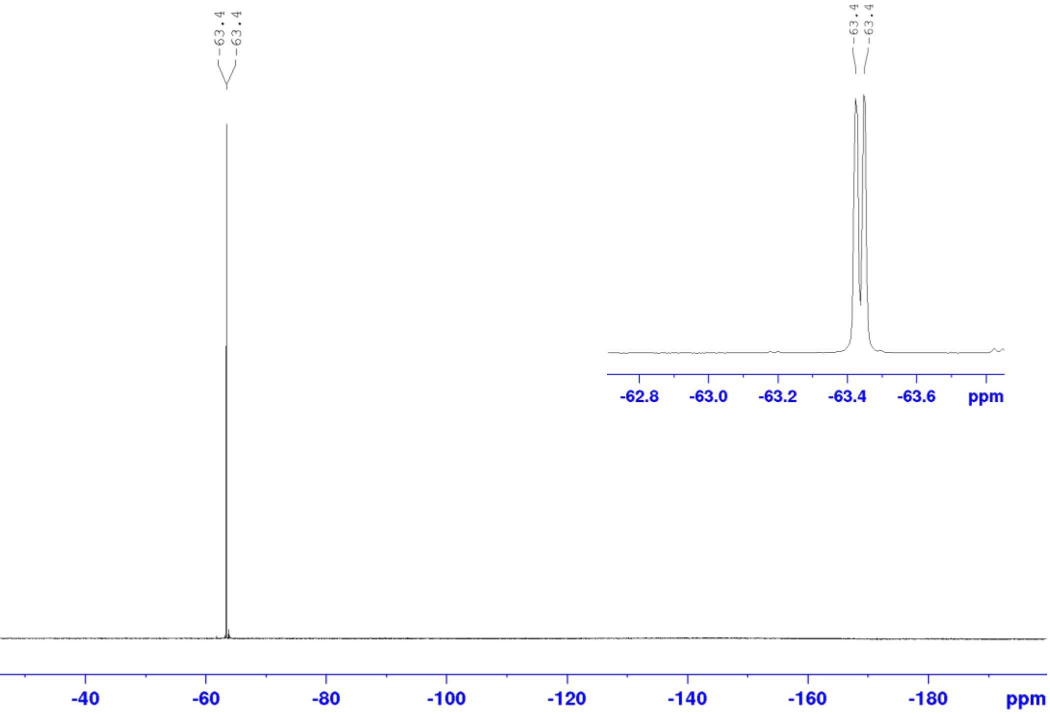

(3'R*,5S*)-*N*-Phenyl-3'-(trifluoromethyl)-4H-spiro[thiazole-5,4'-thiochroman]-2-amine (2ia)

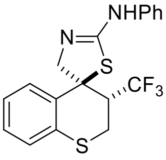

2ia
^{1}H NMR

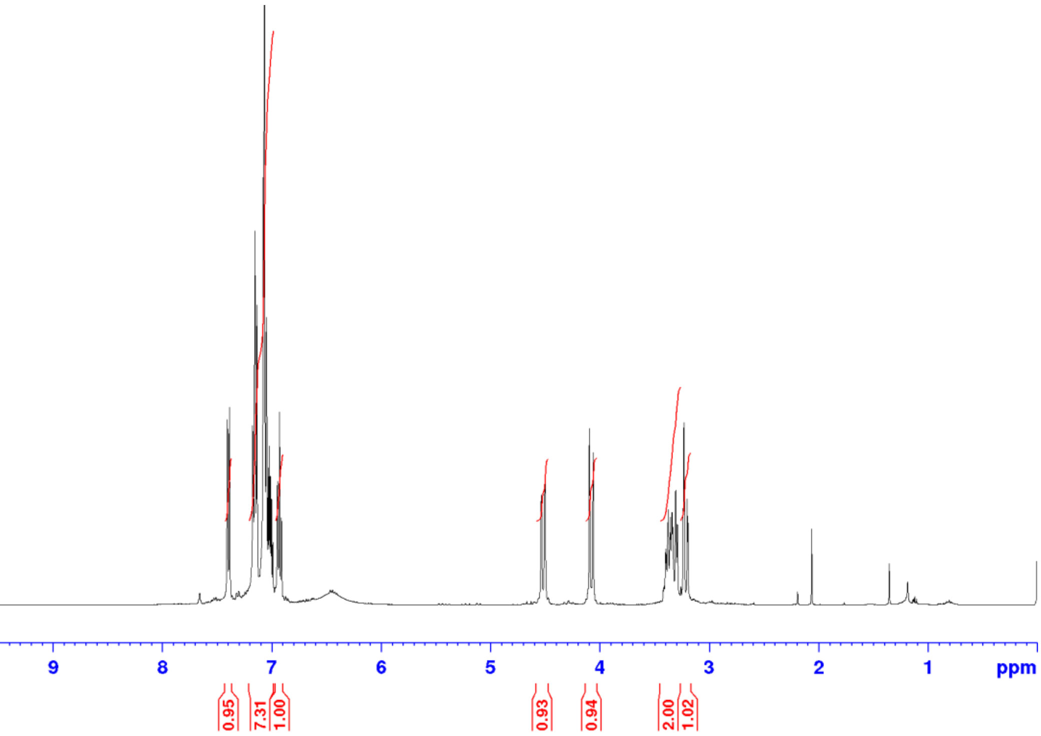

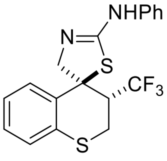

2ia
^{13}C NMR

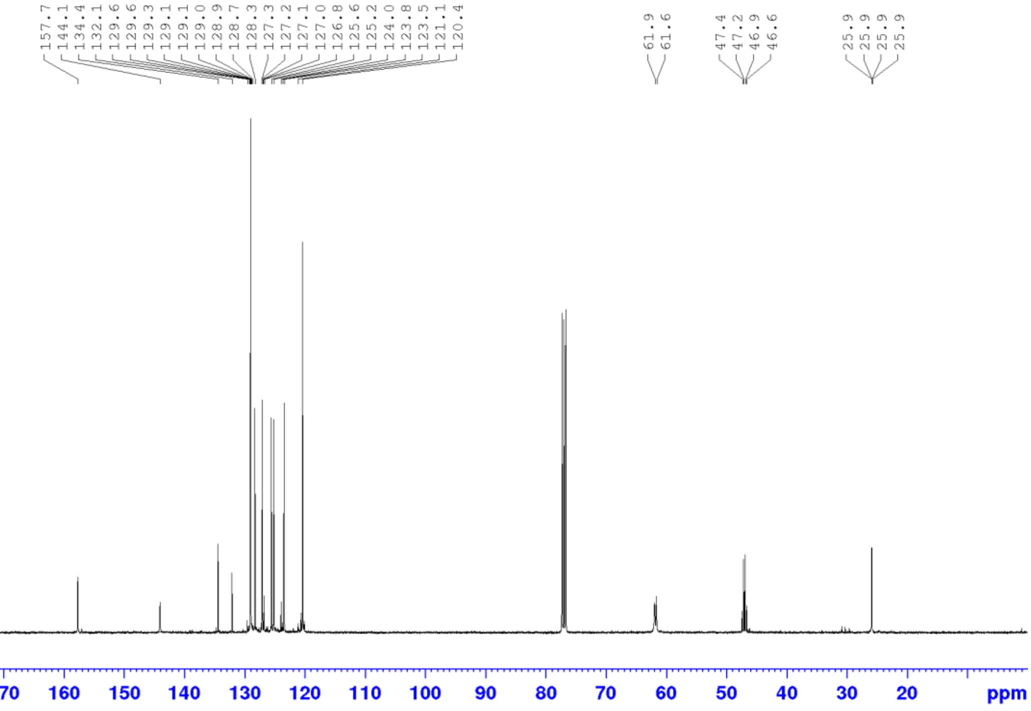

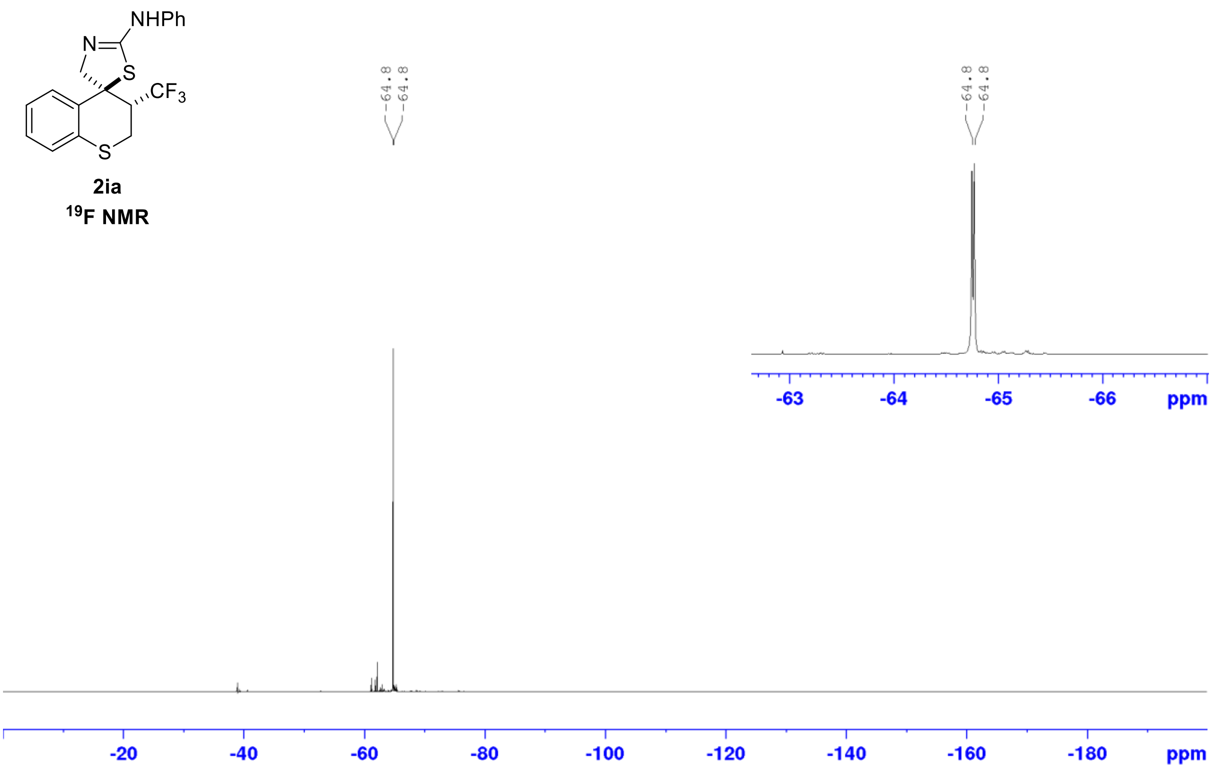

(1S*,2S*)-*N*-Phenyl-2-(trifluoromethyl)-2,3-dihydro-4'H-spiro[indene-1,5'-thiazol]-2'-amine (2ja)

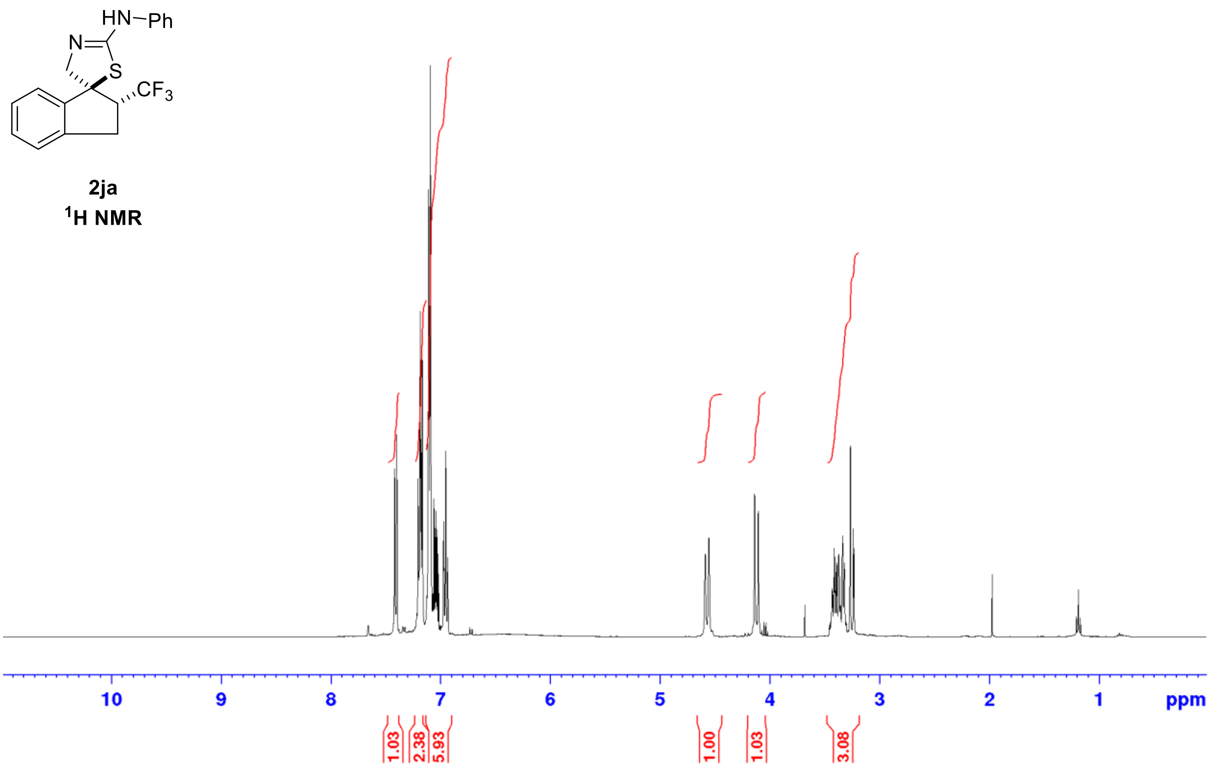

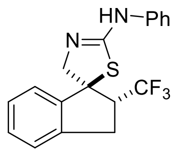

2ja
^{13}C NMR

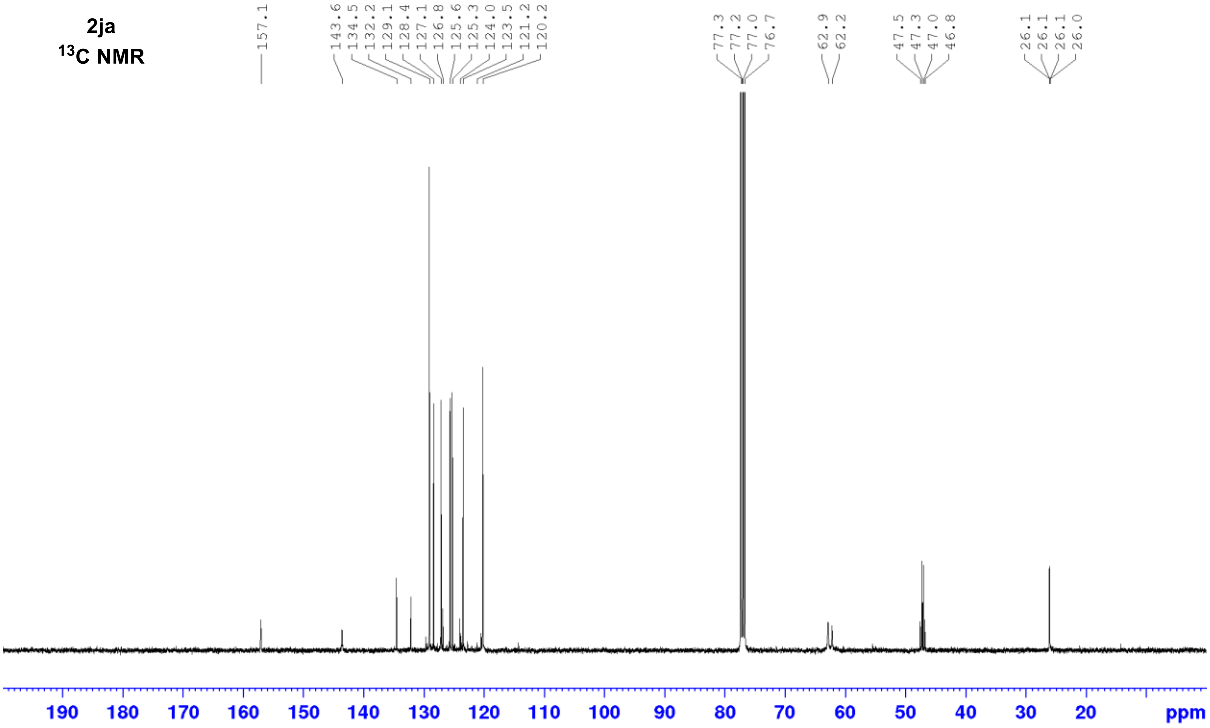

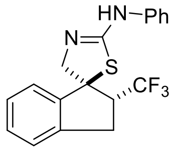

2ja
^{19}F NMR

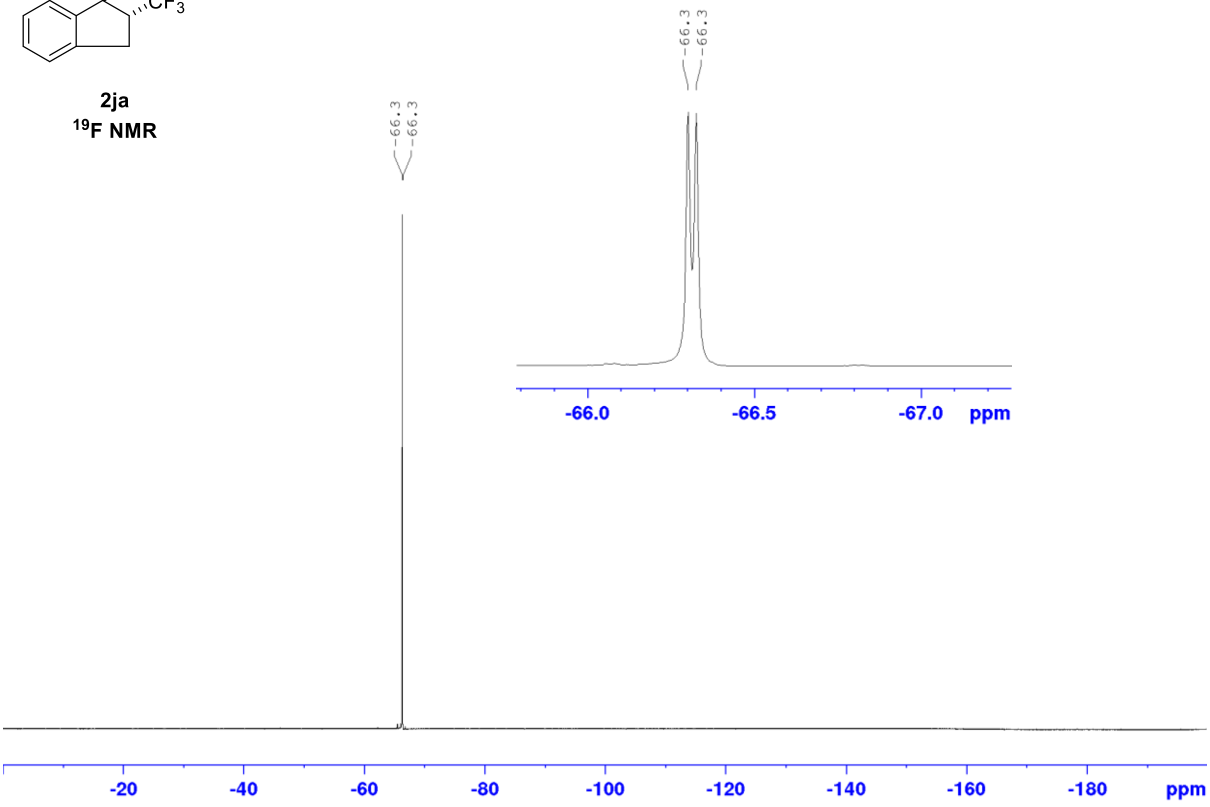

(1*S,2*S**)-*N*-Phenyl-2-(trifluoromethyl)-3,4,4',5'-tetrahydro-2H-spiro[naphthalene-1,6'-[1,3]thiazin]-2'-amine (2ka)**

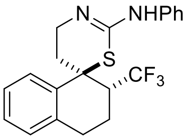

2ka
^{1}H NMR

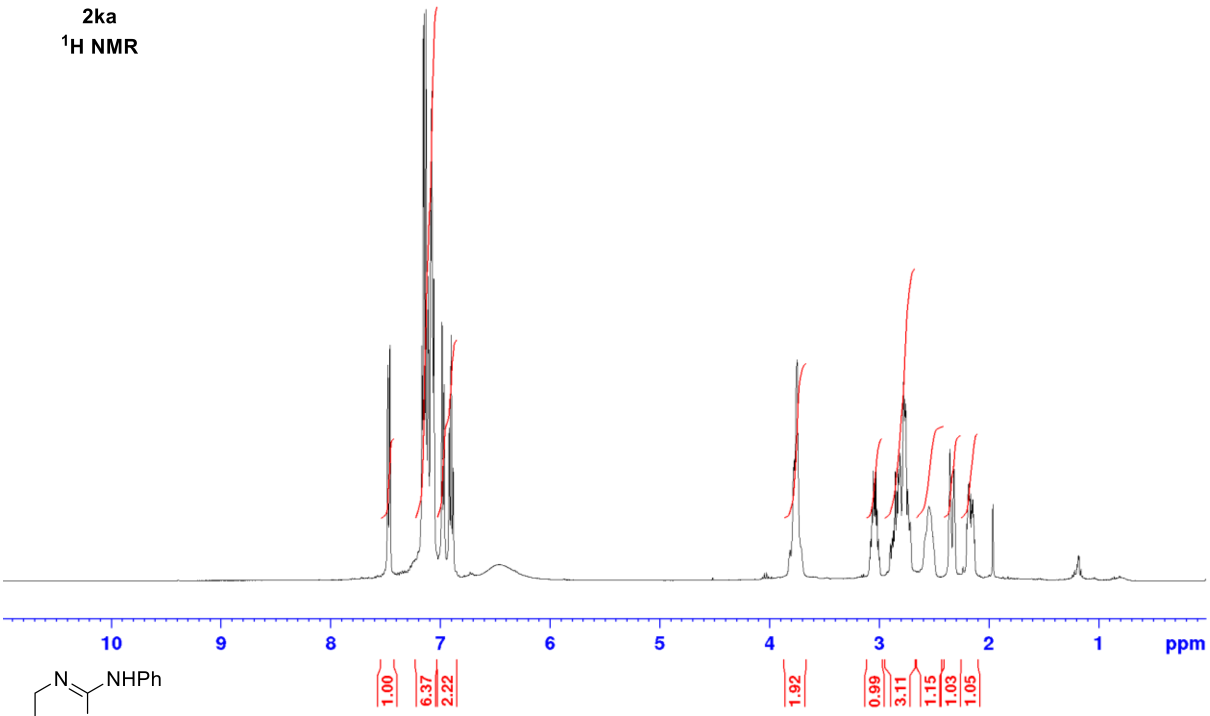

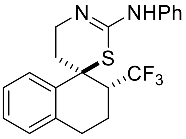

2ka
^{13}C NMR

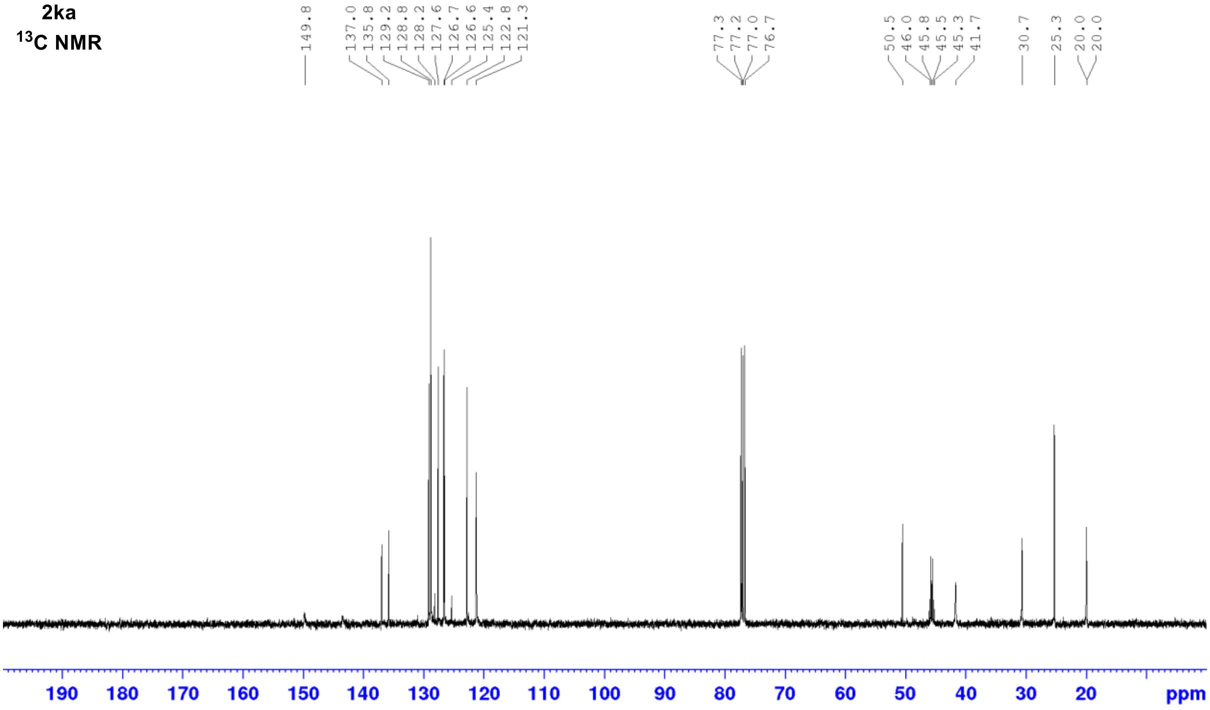

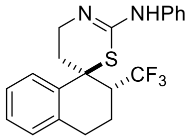

2ka

^{19}F NMR

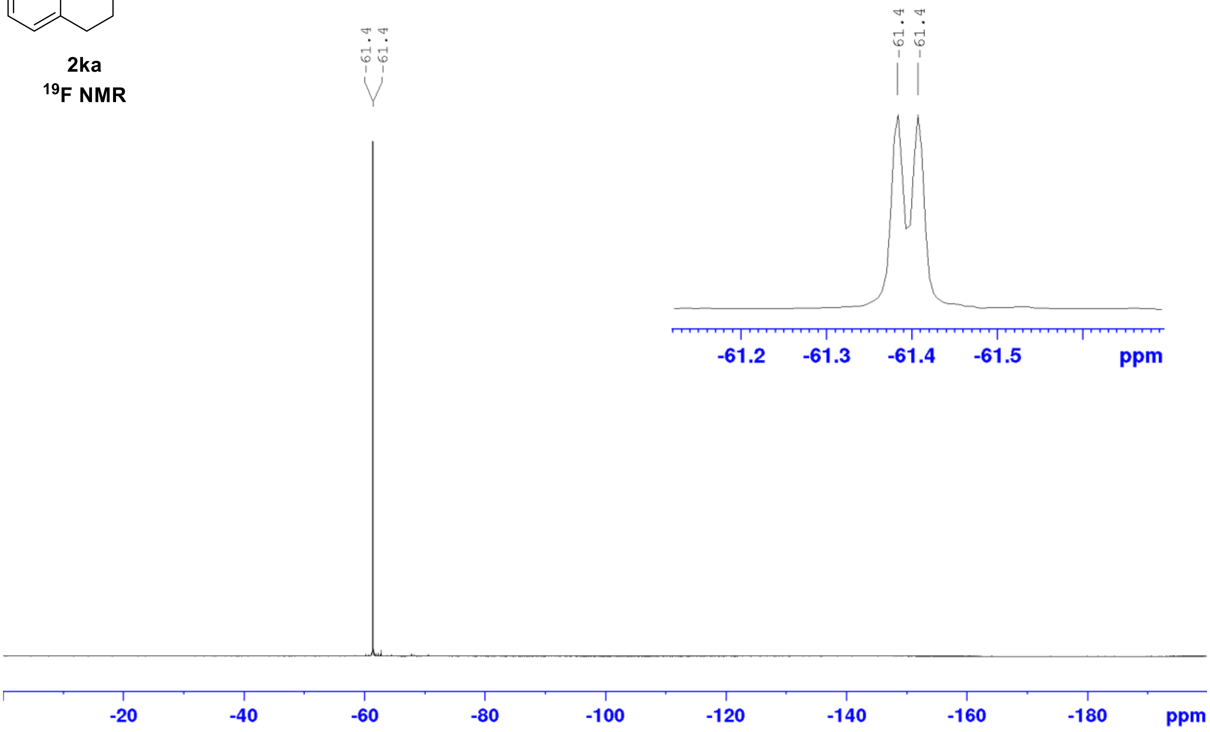

4-Methyl-*N*-phenyl-4-(2,2,2-trifluoroethyl)-4H-benzo[d][1,3]thiazin-2-amine (2la)

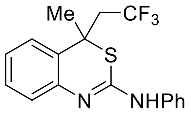

2la

^{1}H NMR

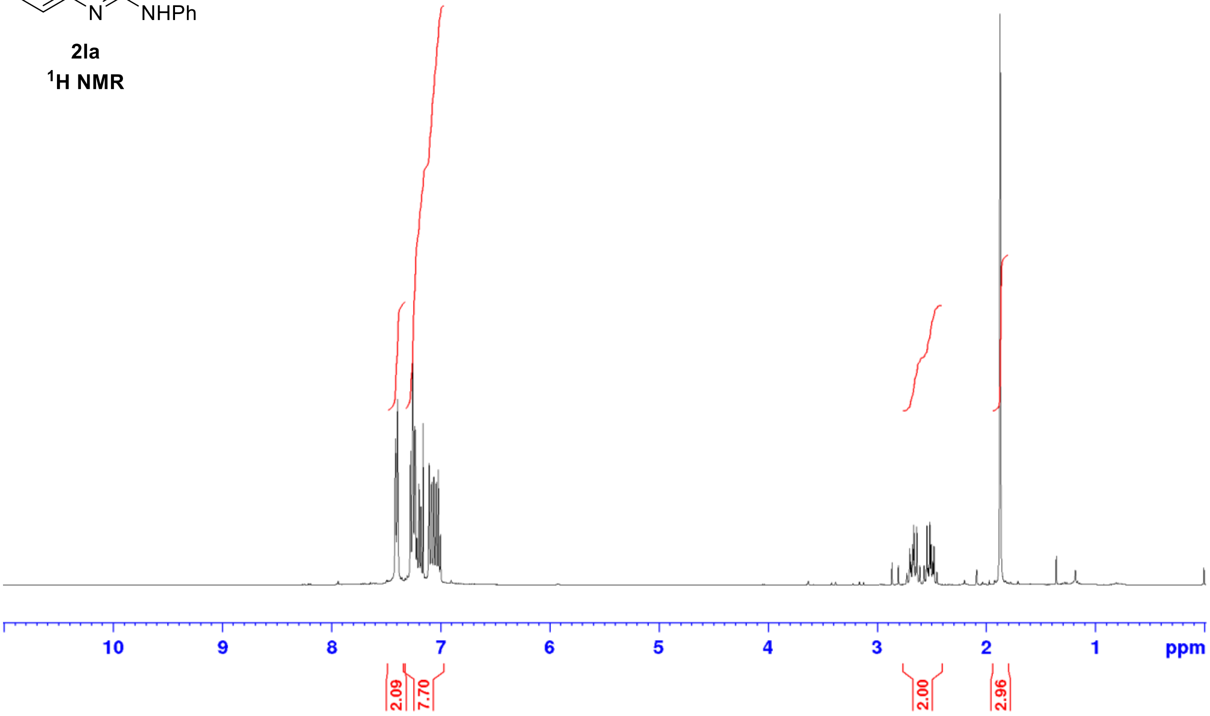

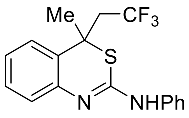

2la
^{13}C NMR

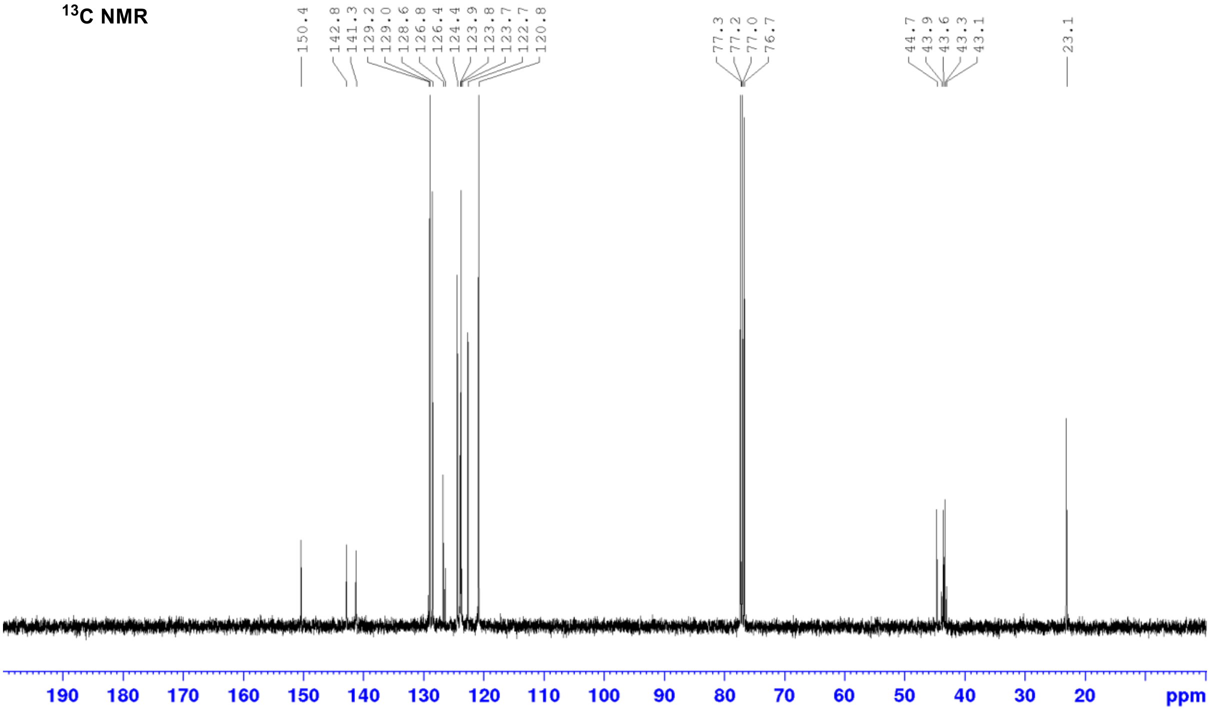

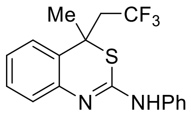

2la
^{19}F NMR

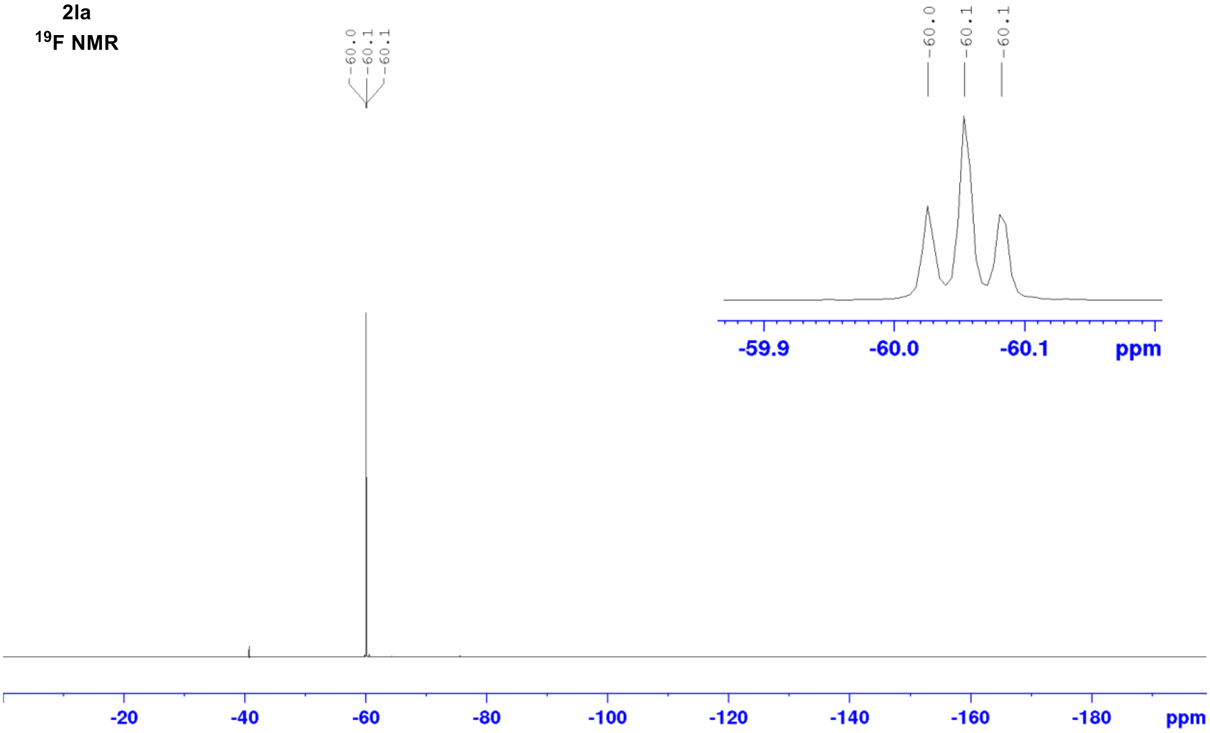

7-Fluoro-4-methyl-*N*-phenyl-4-(2,2,2-trifluoroethyl)-4H-benzo[d][1,3]thiazin-2-amine (2ma)

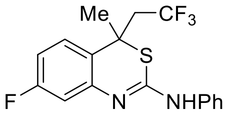

2ma
^{1}H NMR

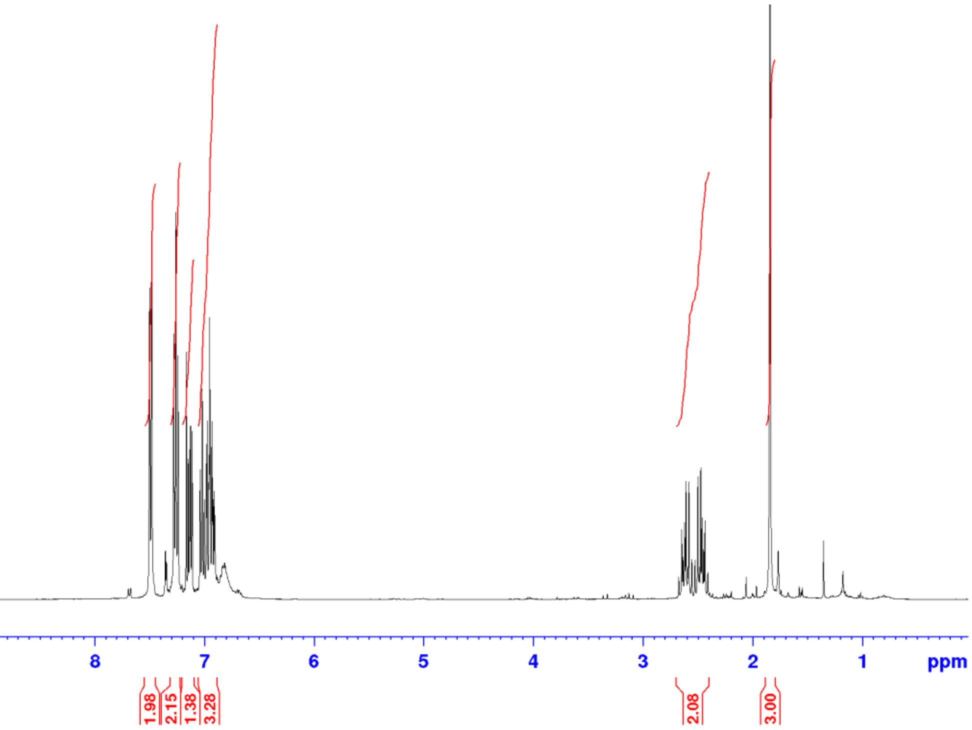

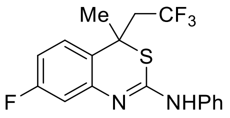

2ma
^{13}C NMR

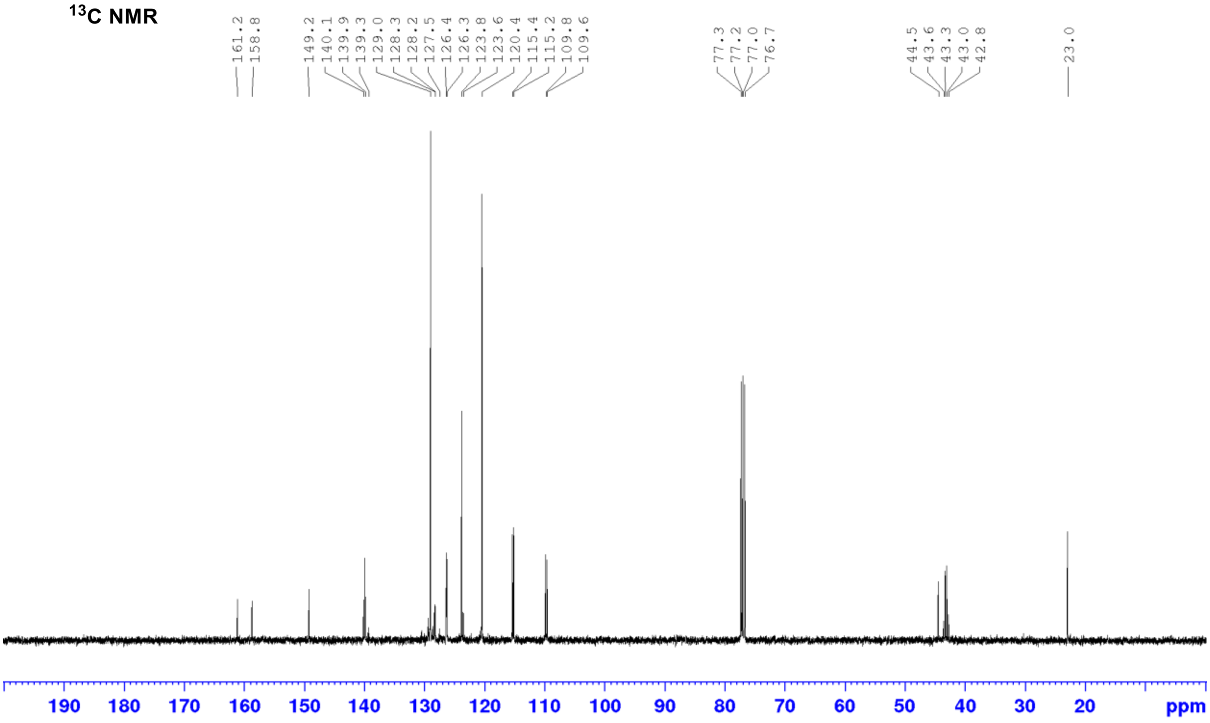

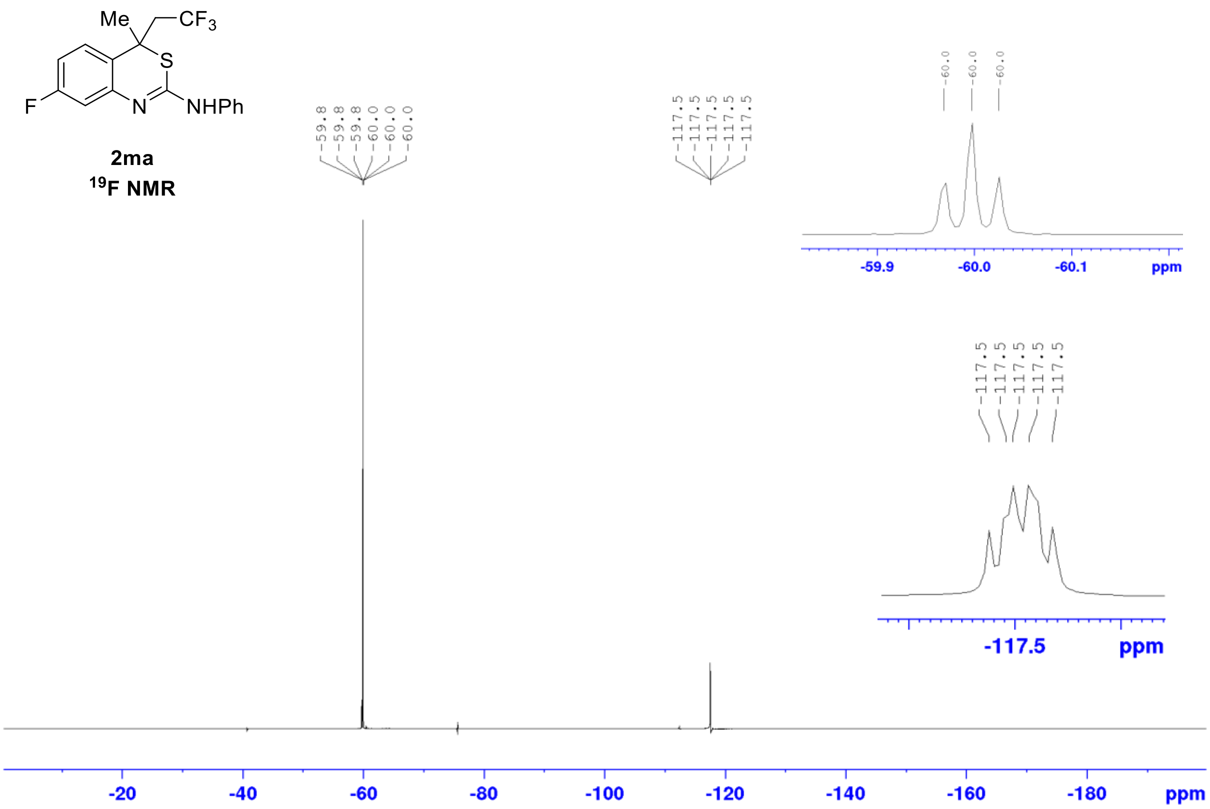

2ma
^{19}F NMR

(5*R**,6*S**)-*N*,6-Diphenyl-5-(trifluoromethyl)-5,6-dihydro-4H-1,3-thiazin-2-amine (2na)

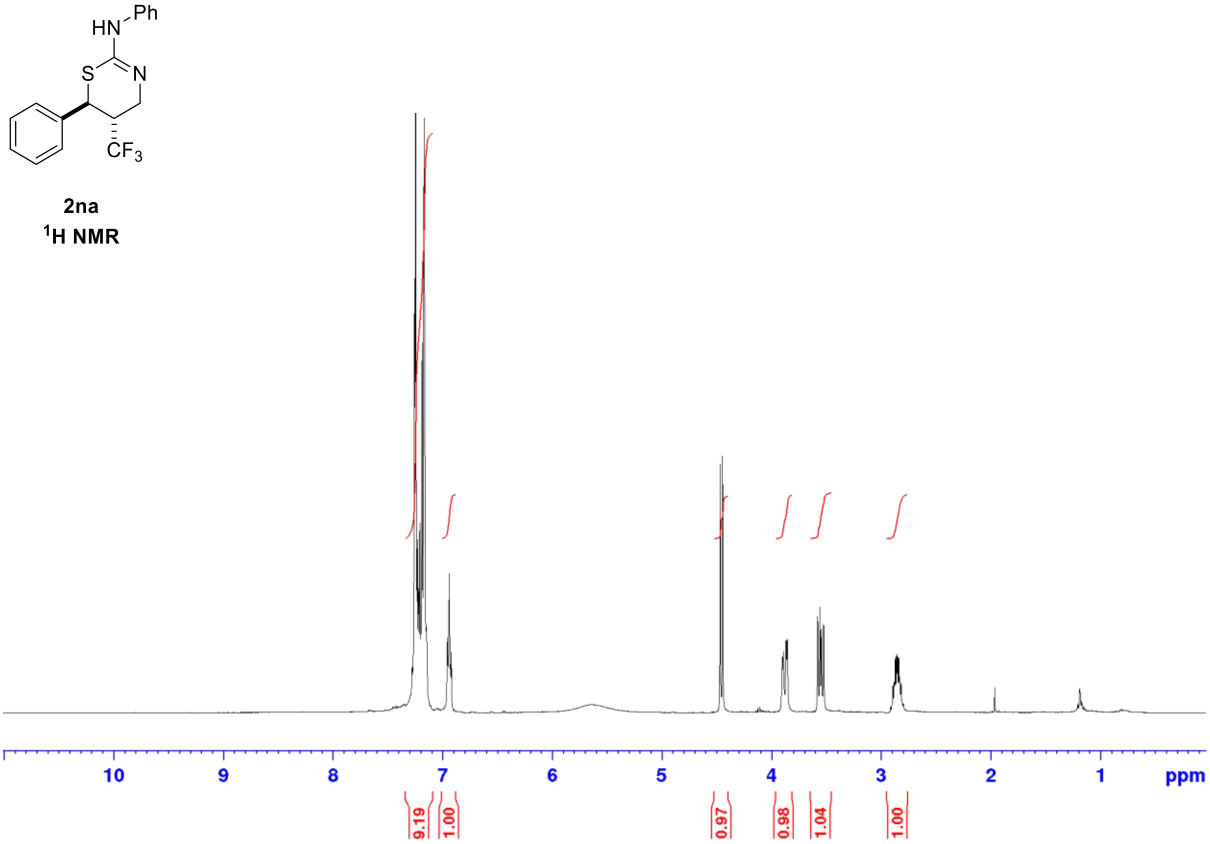

2na
^{1}H NMR

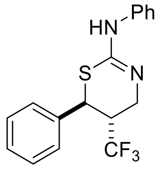

2na

^{13}C NMR

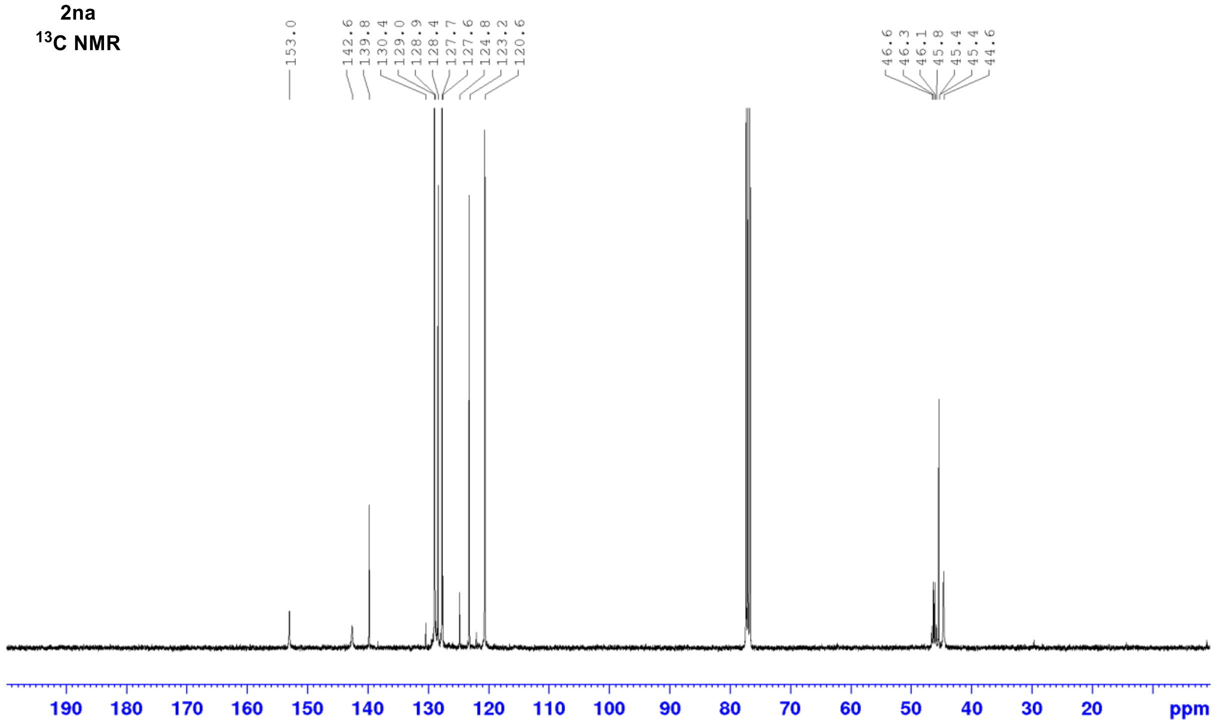

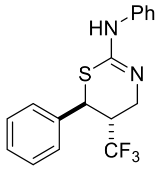

2na

^{19}F NMR

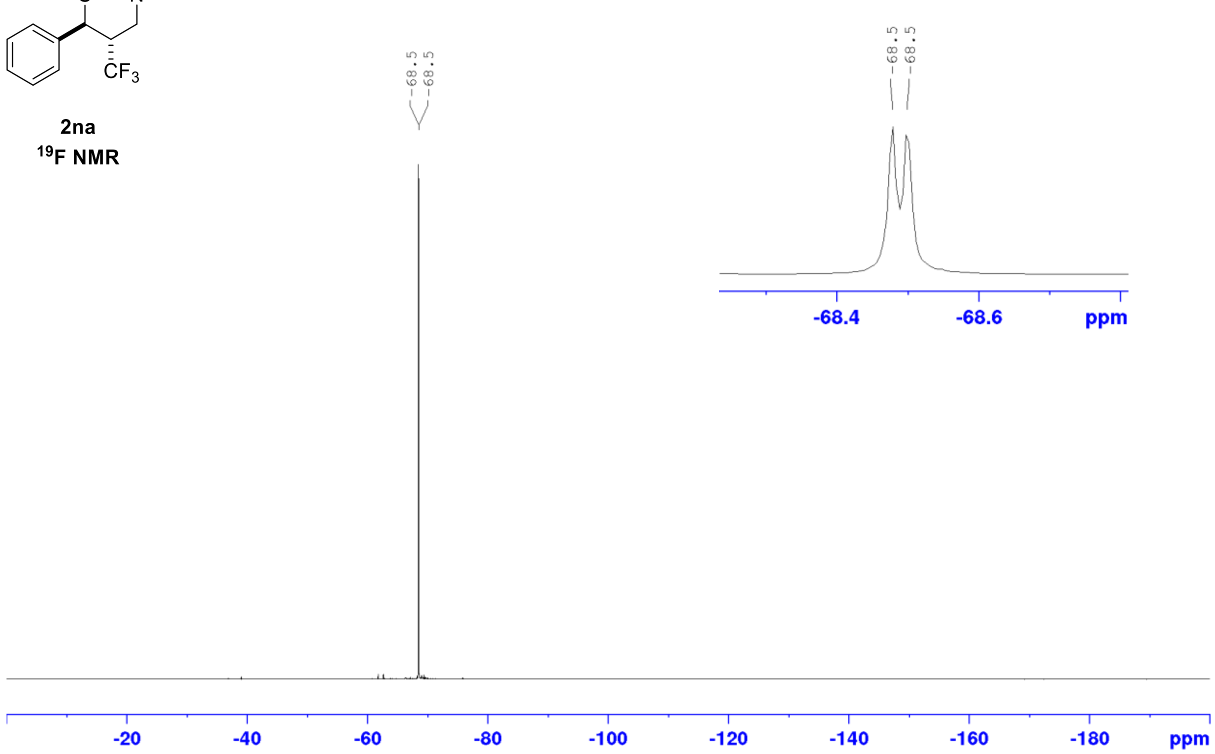

(5*R,6*S**)-6-(4-Methoxyphenyl)-*N*-phenyl-5-(trifluoromethyl)-5,6-dihydro-4H-1,3-thiazin-2-amine (2oa)**

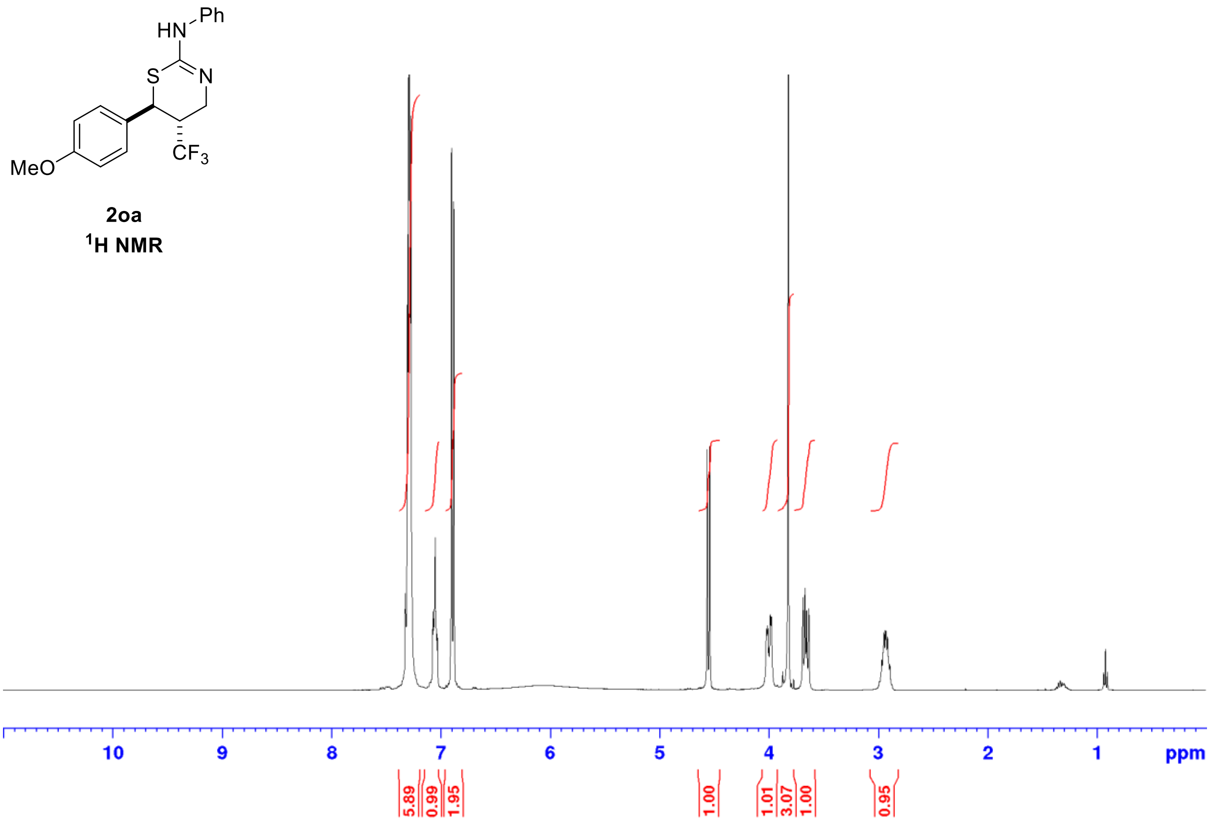

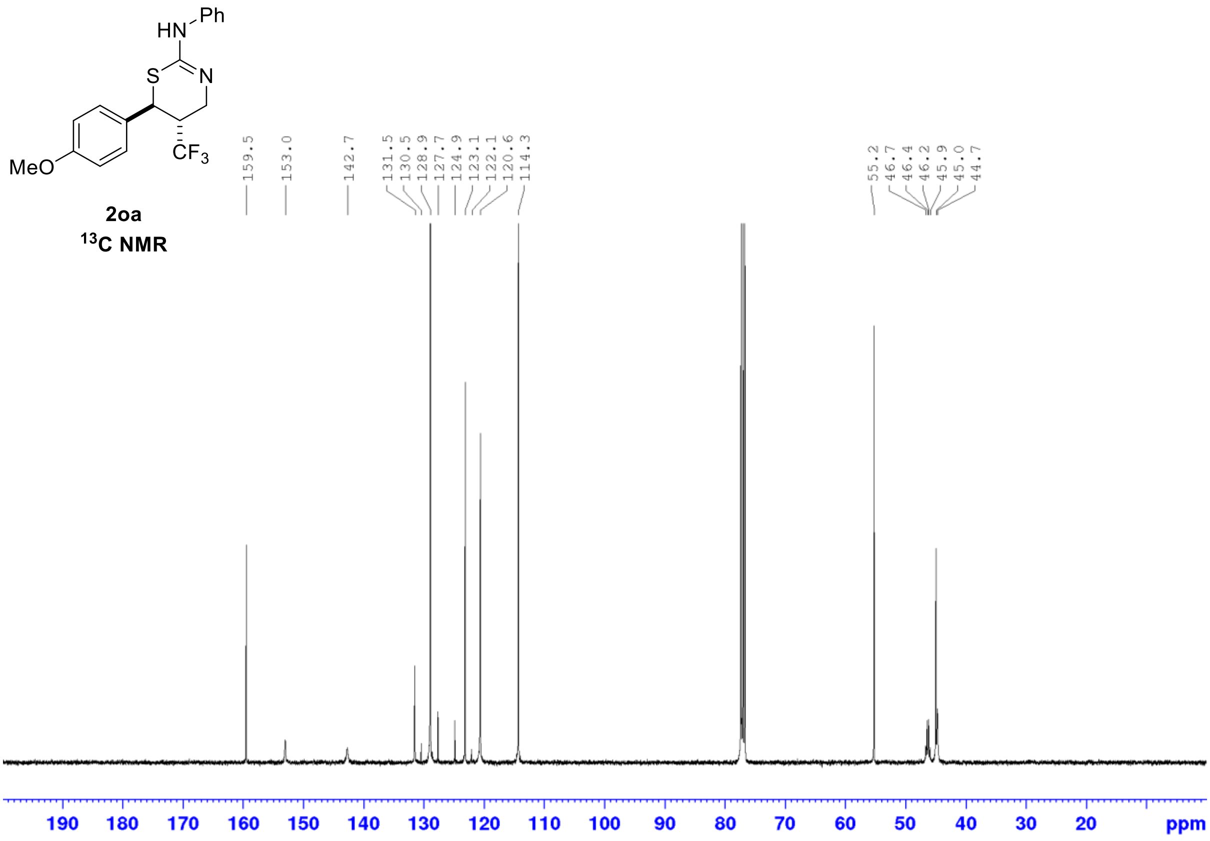

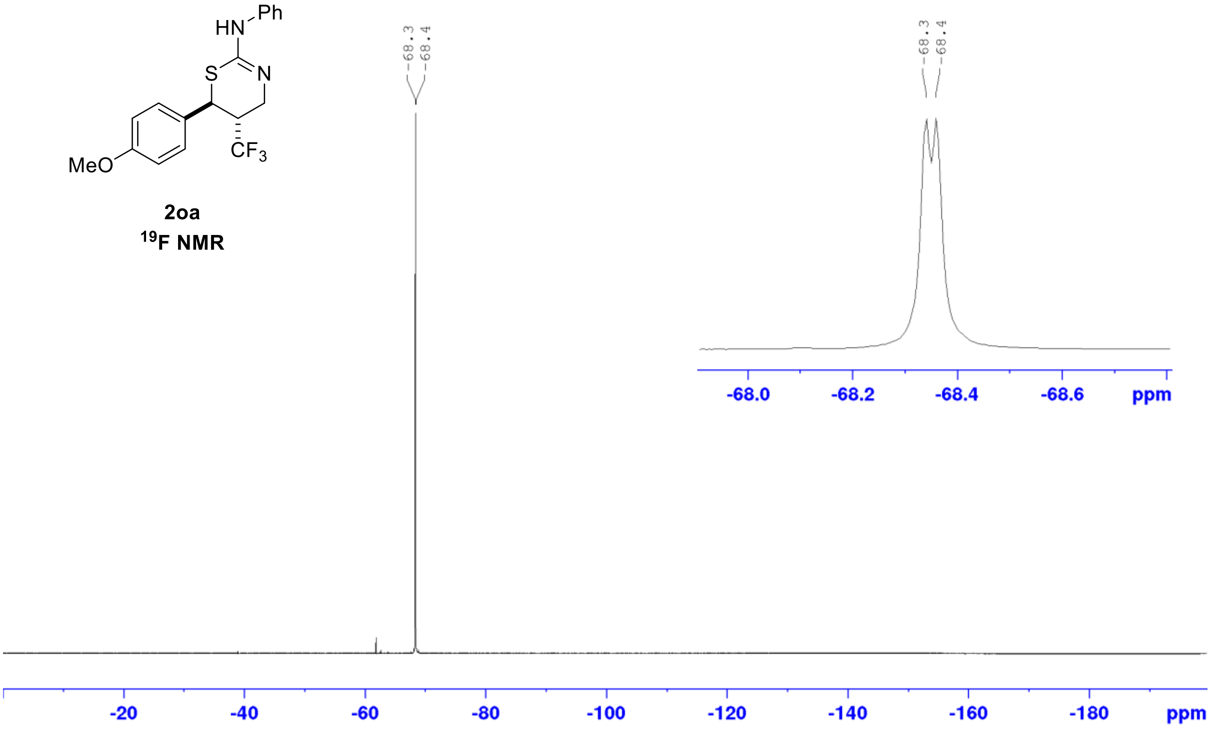

(5*R,6*S**)-*N*-Phenyl-6-(*p*-tolyl)-5-(trifluoromethyl)-5,6-dihydro-4H-1,3-thiazin-2-amine (2pa)**

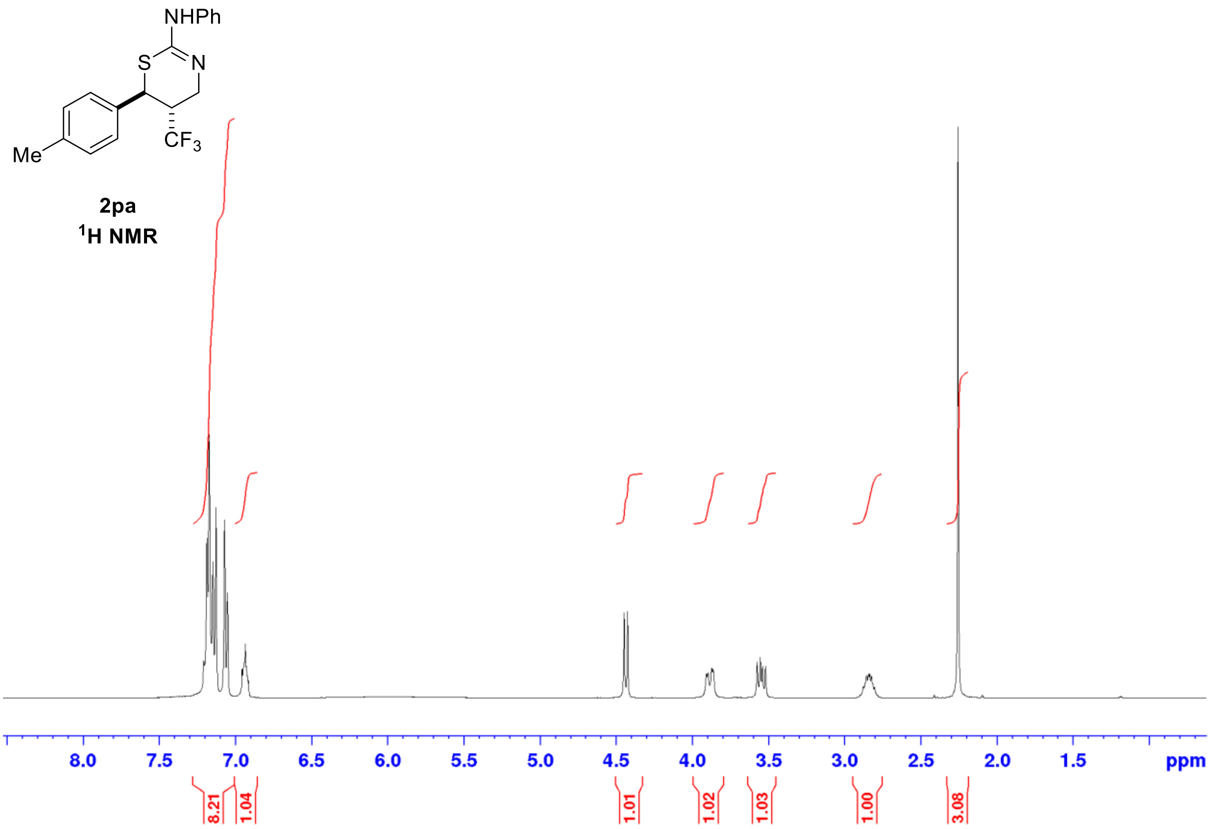

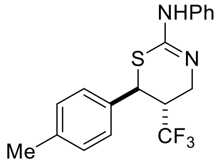

2pa
^{13}C NMR

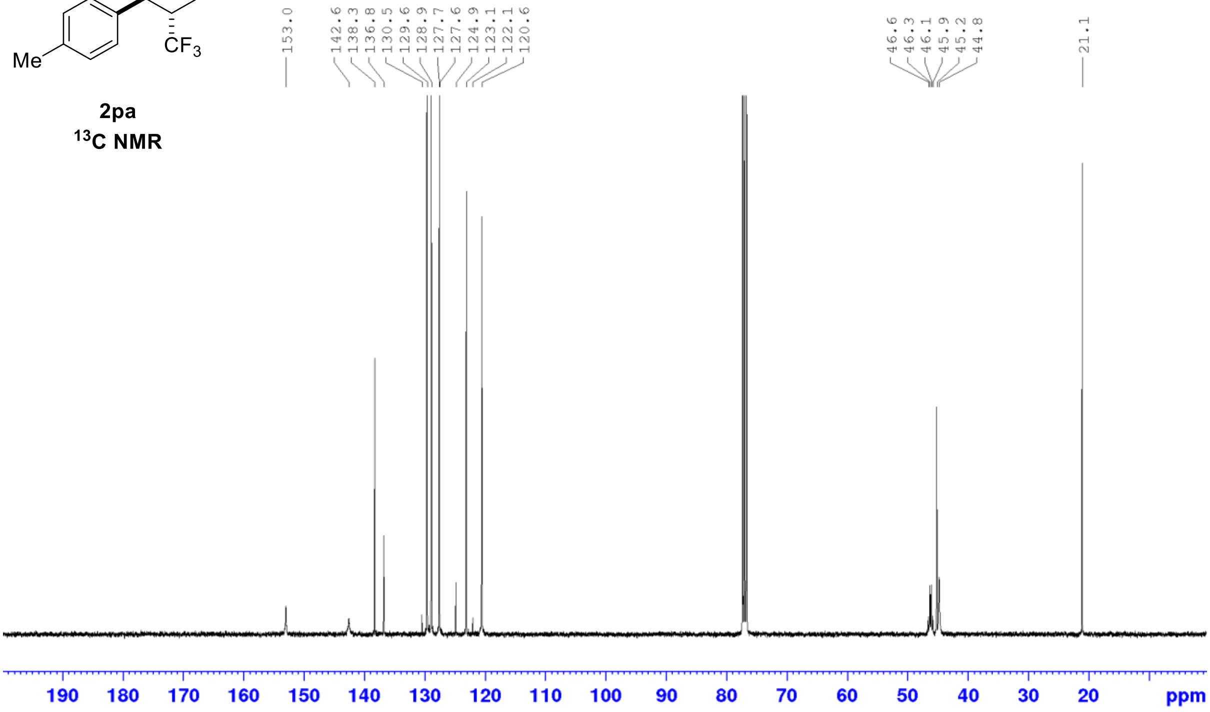

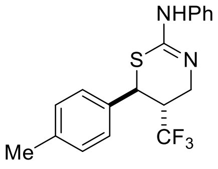

2pa
^{19}F NMR

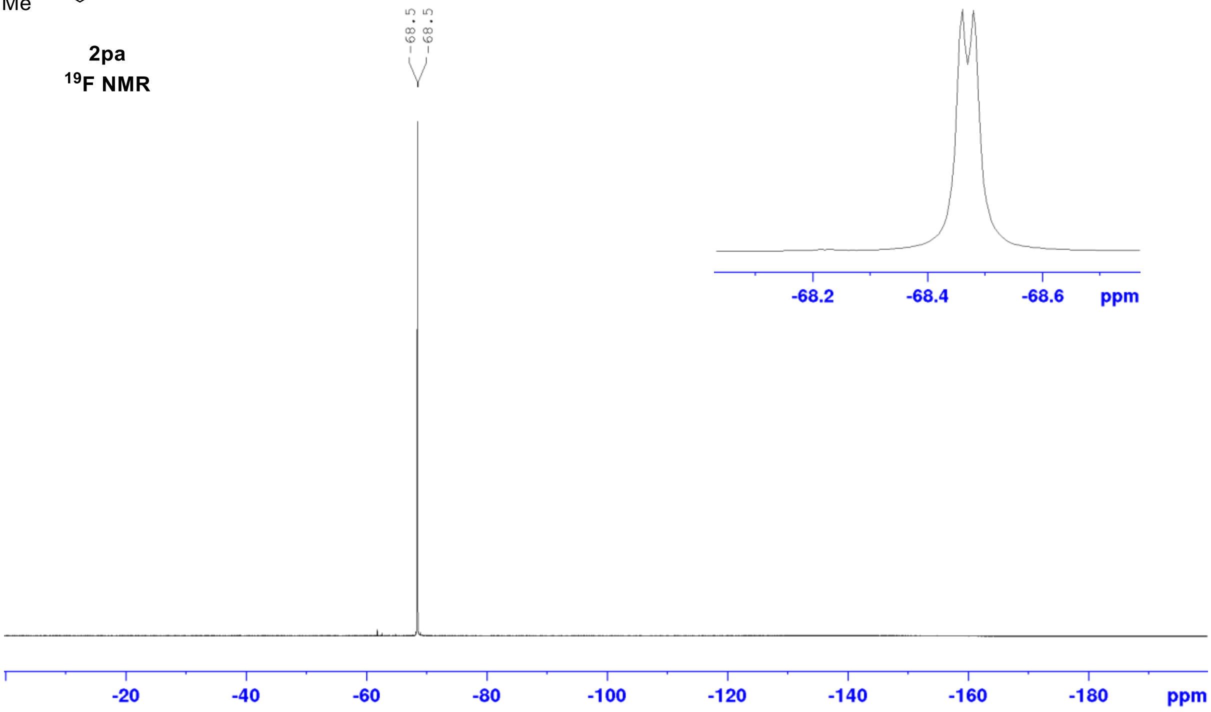

(5*R,6*S**)-6-(4-Chlorophenyl)-*N*-phenyl-5-(trifluoromethyl)-5,6-dihydro-4H-1,3-thiazin-2-amine (2qa)**

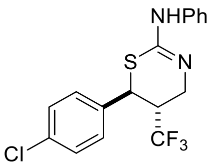

2qa

^{1}H NMR

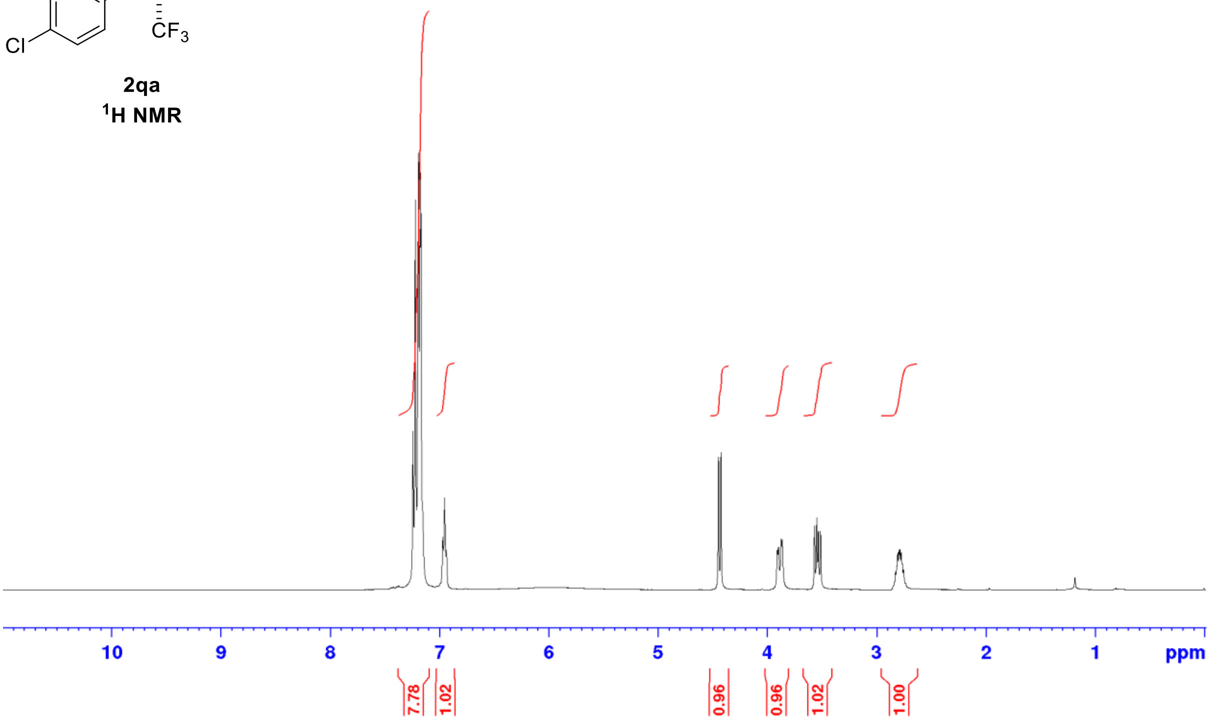

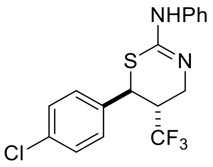

2qa

^{13}C NMR

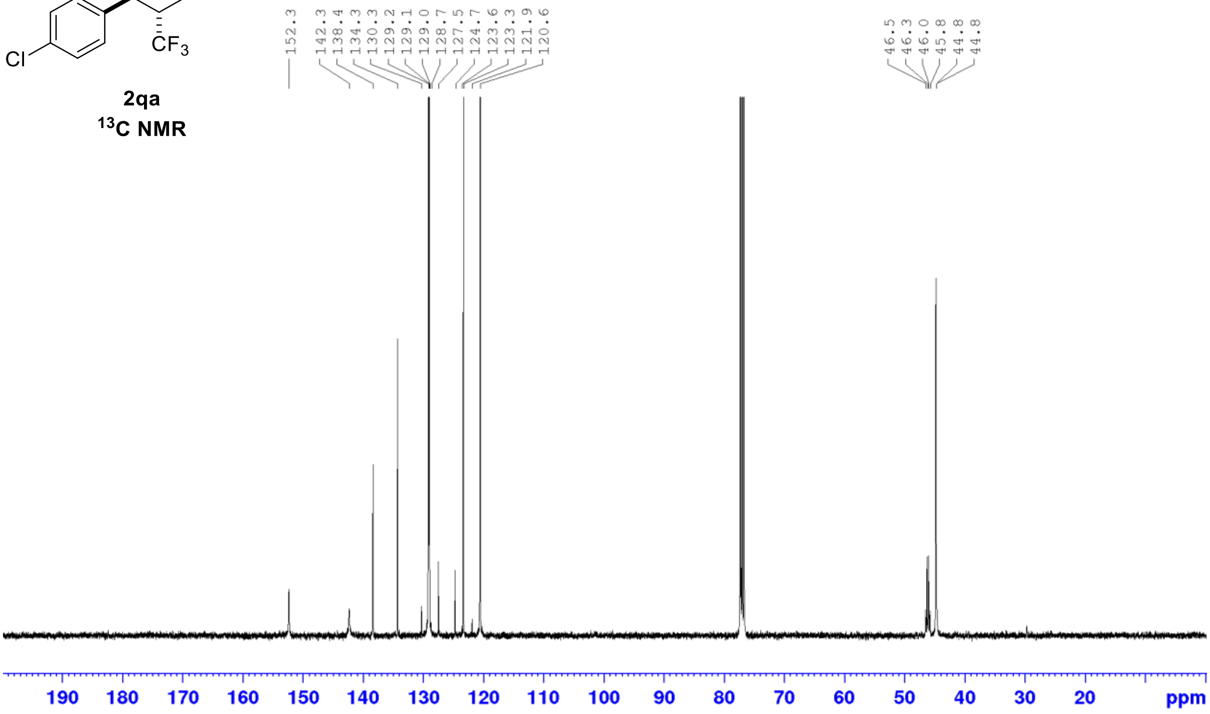

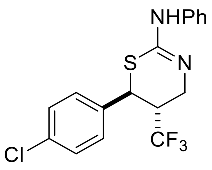

2qa
^{19}F NMR

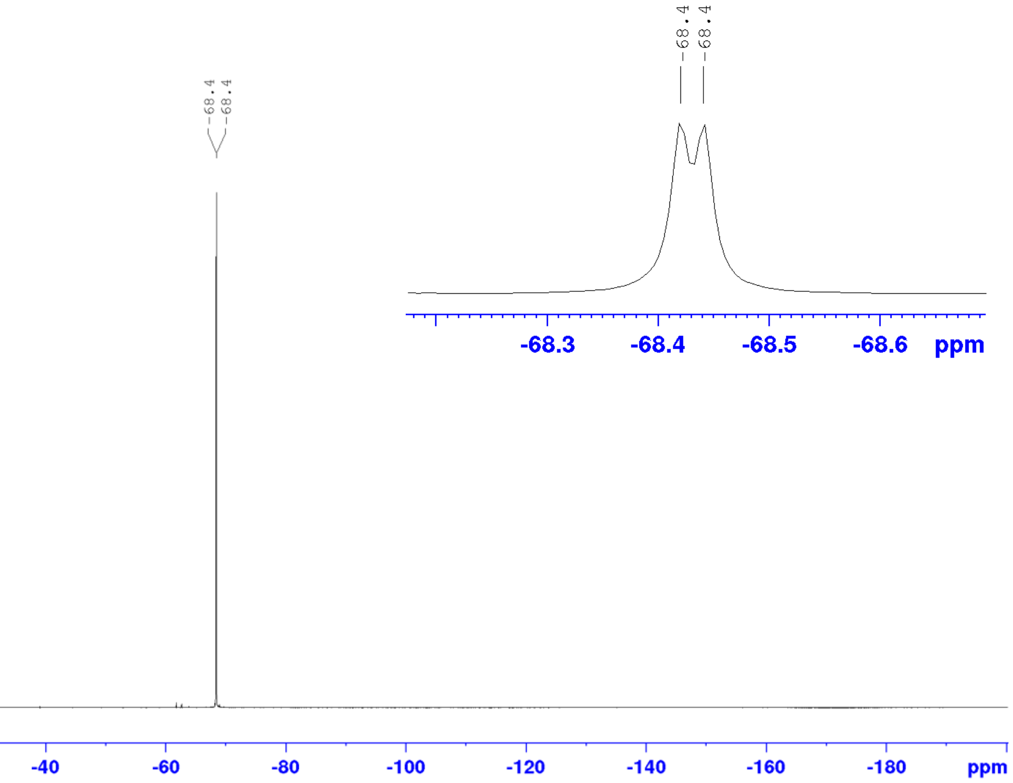

(5*R**,6*S**)-6-phenyl-*N*-(*p*-tolyl)-5-(trifluoromethyl)-5,6-dihydro-4H-1,3-thiazin-2-amine (2nb)

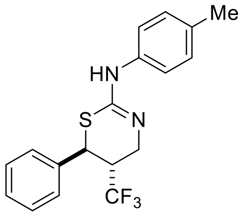

^{1}H NMR
2nb

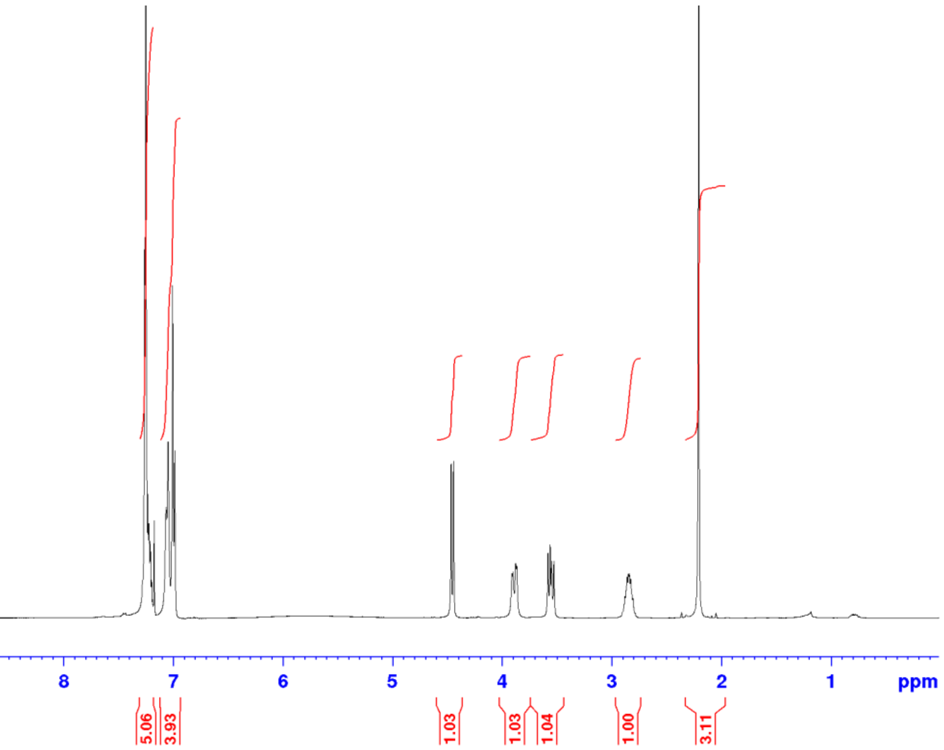

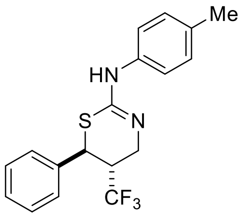

^{13}C NMR
2nb

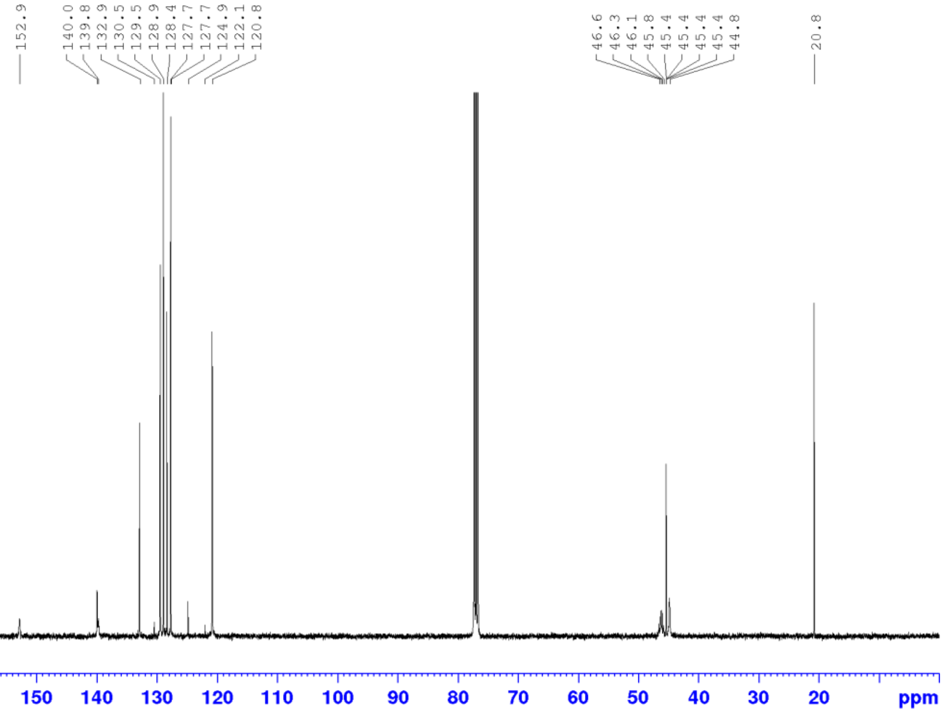

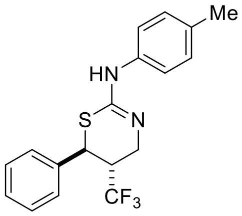

^{19}F NMR
2nb

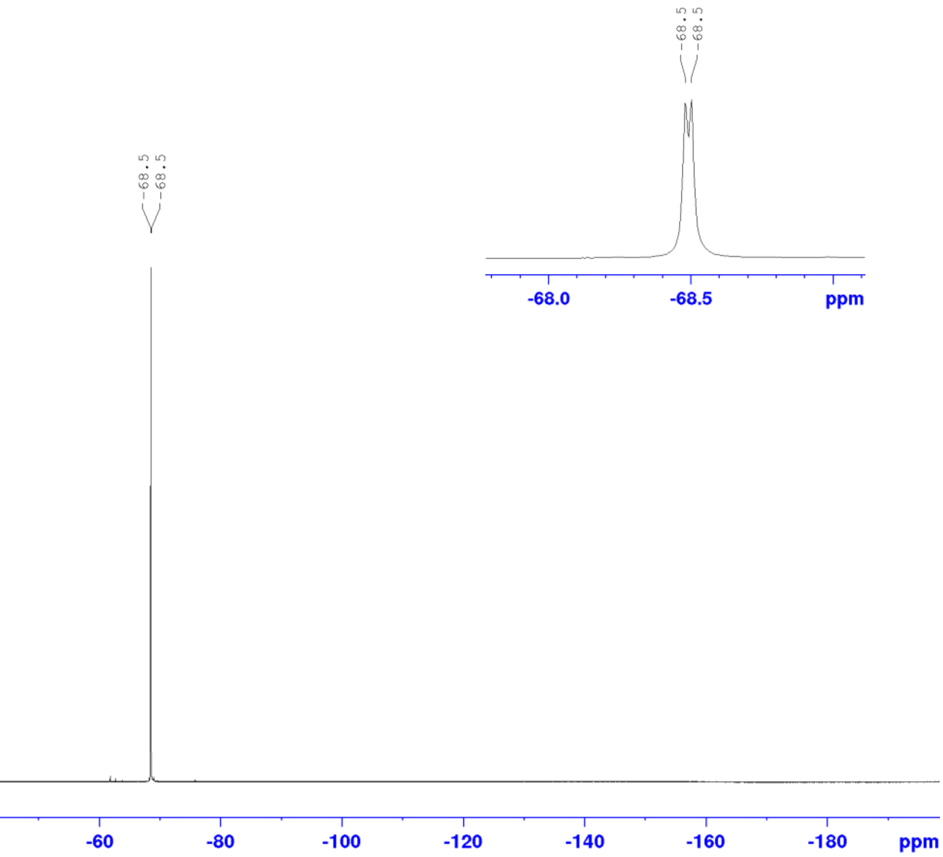

(5*R,6*S**)-*N*-(4-methoxyphenyl)-6-phenyl-5-(trifluoromethyl)-5,6-dihydro-4H-1,3-thiazin-2-amine (2nc)**

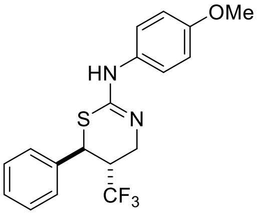

2nc
^{1}H NMR

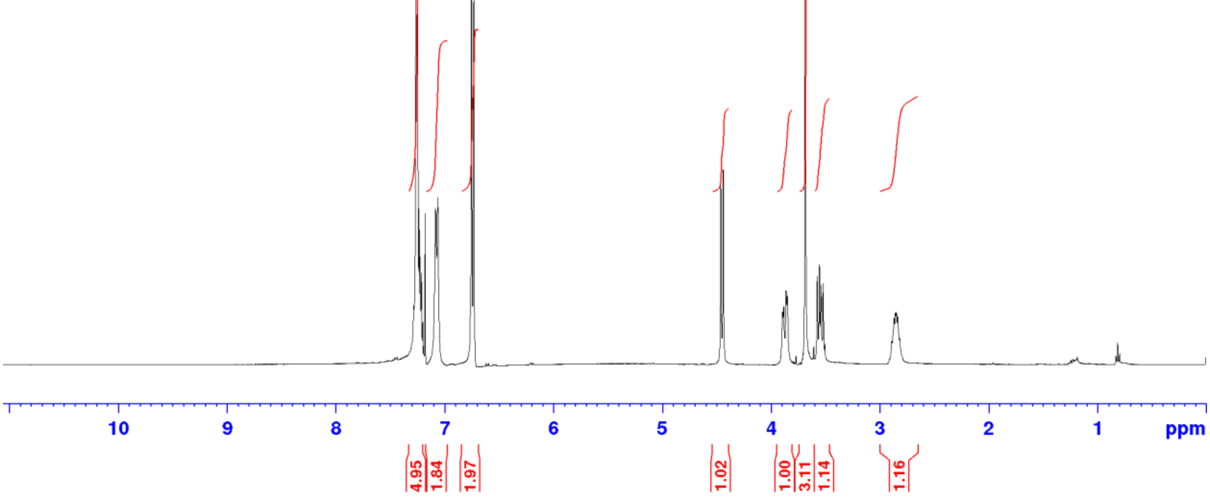

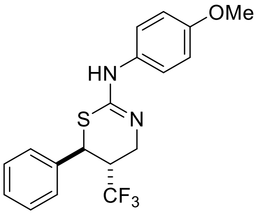

2nc
^{13}C NMR

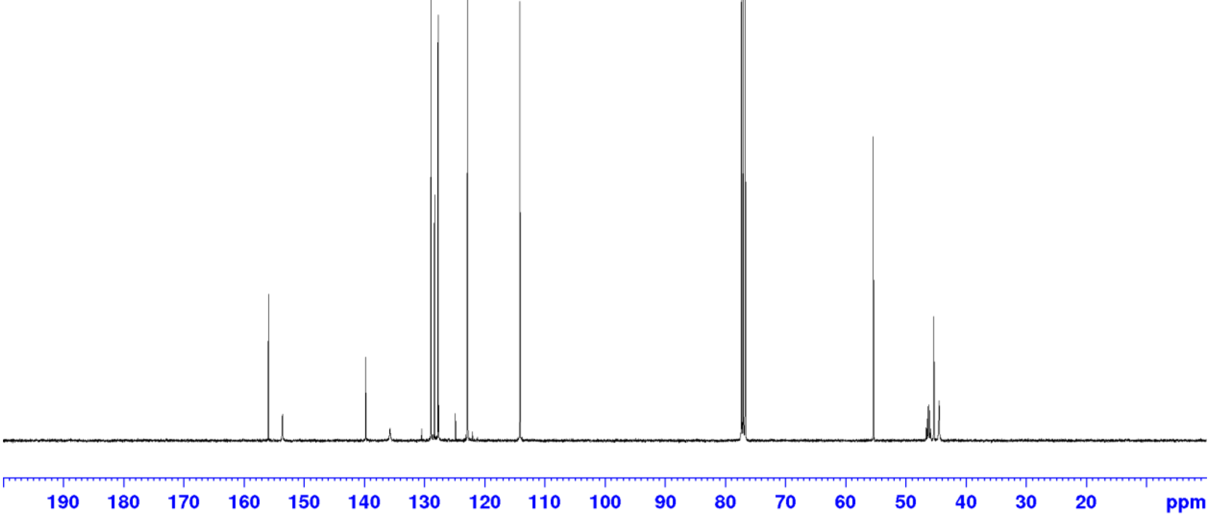

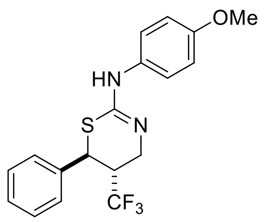

2nc
^{19}F NMR

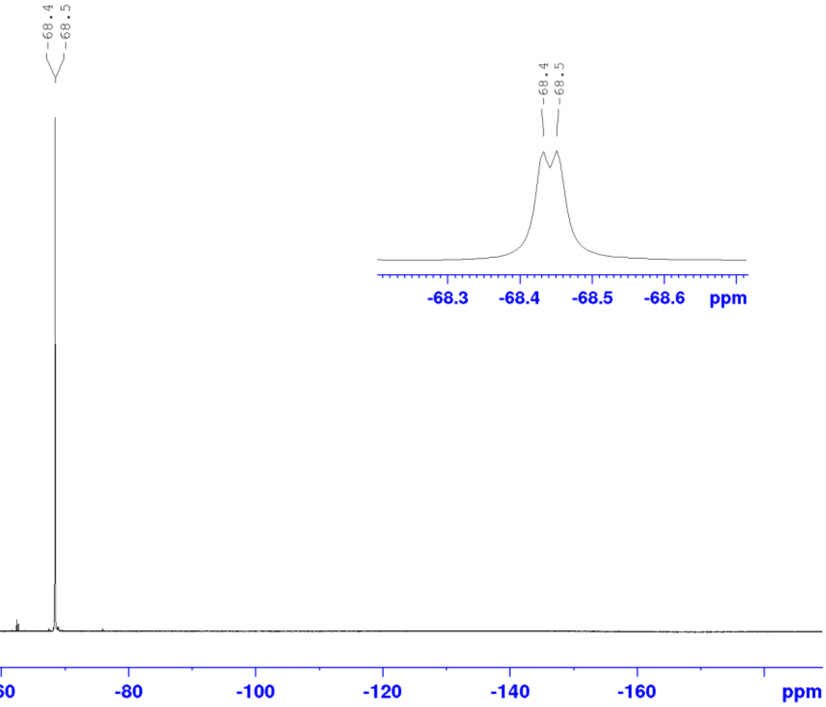

(5*R**,6*S**)-6-Phenyl-5-(trifluoromethyl)-*N*-(4-(trifluoromethyl)phenyl)-5,6-dihydro-4H-1,3-thiazin-2-amine (2nd)

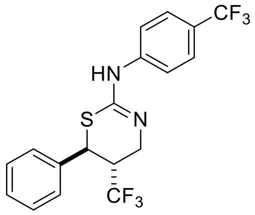

2nd
^{1}H NMR

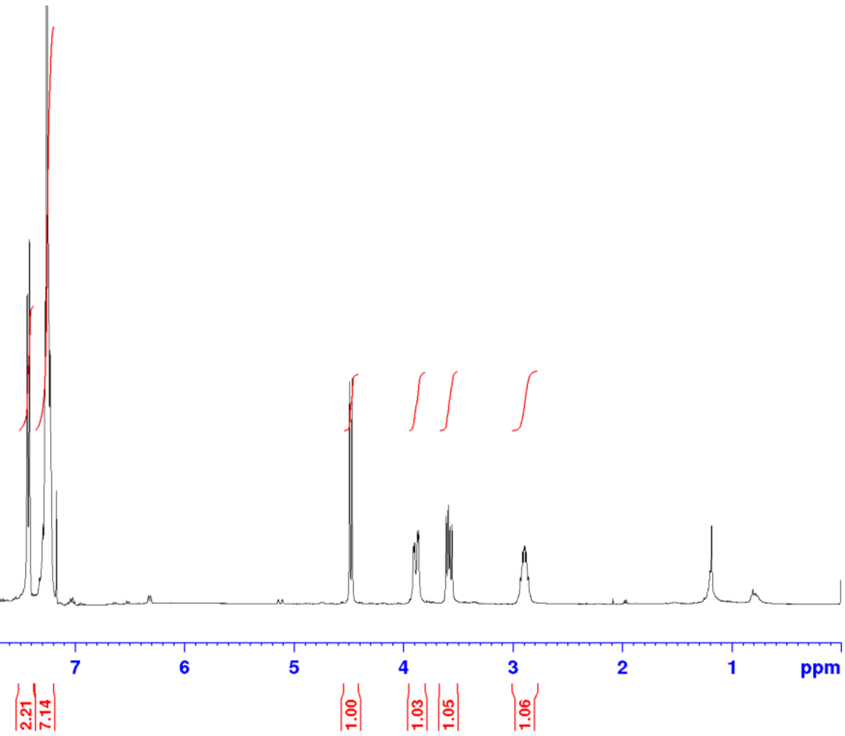

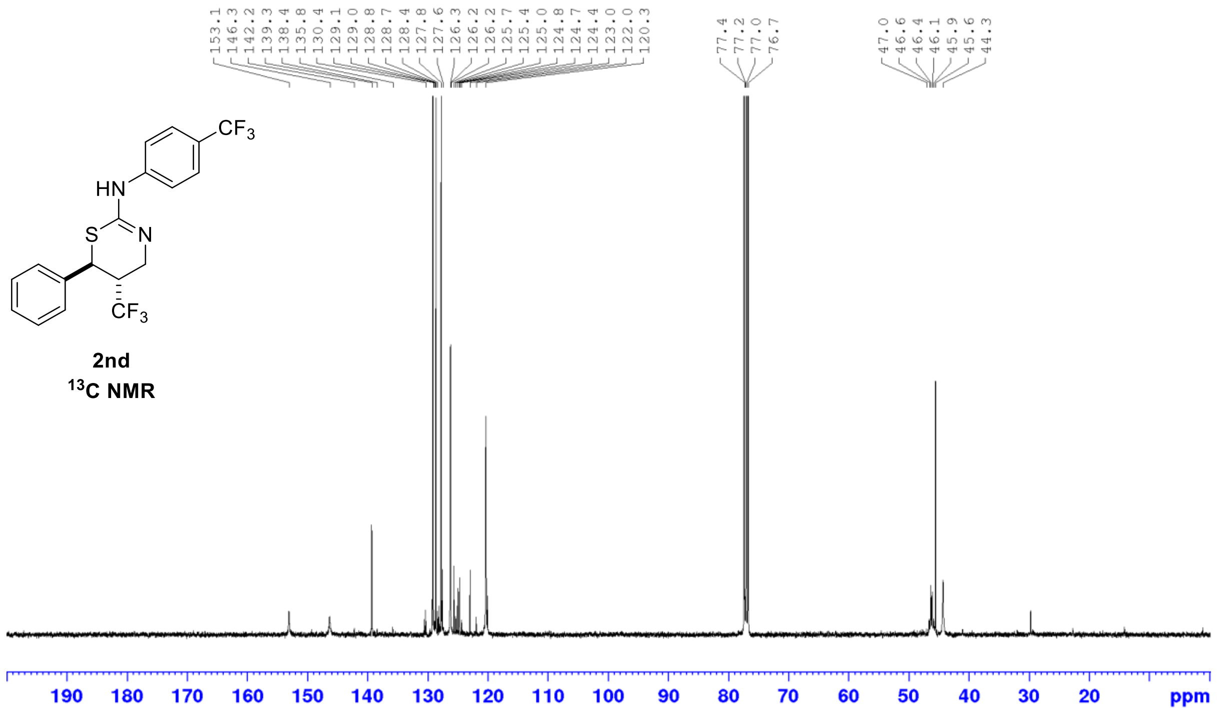

CF3
HN
S
N
CF3
2nd
13C NMR
153.1 146.3 142.2 139.3 138.4 135.8 130.4 129.1 129.0 128.8 128.7 128.4 127.8 127.6 126.3 126.2 126.2 125.7 125.4 125.0 124.8 124.7 124.4 123.0 122.0 120.3
77.4 77.2 77.0 76.7
47.0 46.6 46.4 46.1 45.9 45.6 44.3
190 180 170 160 150 140 130 120 110 100 90 80 70 60 50 40 30 20 ppm

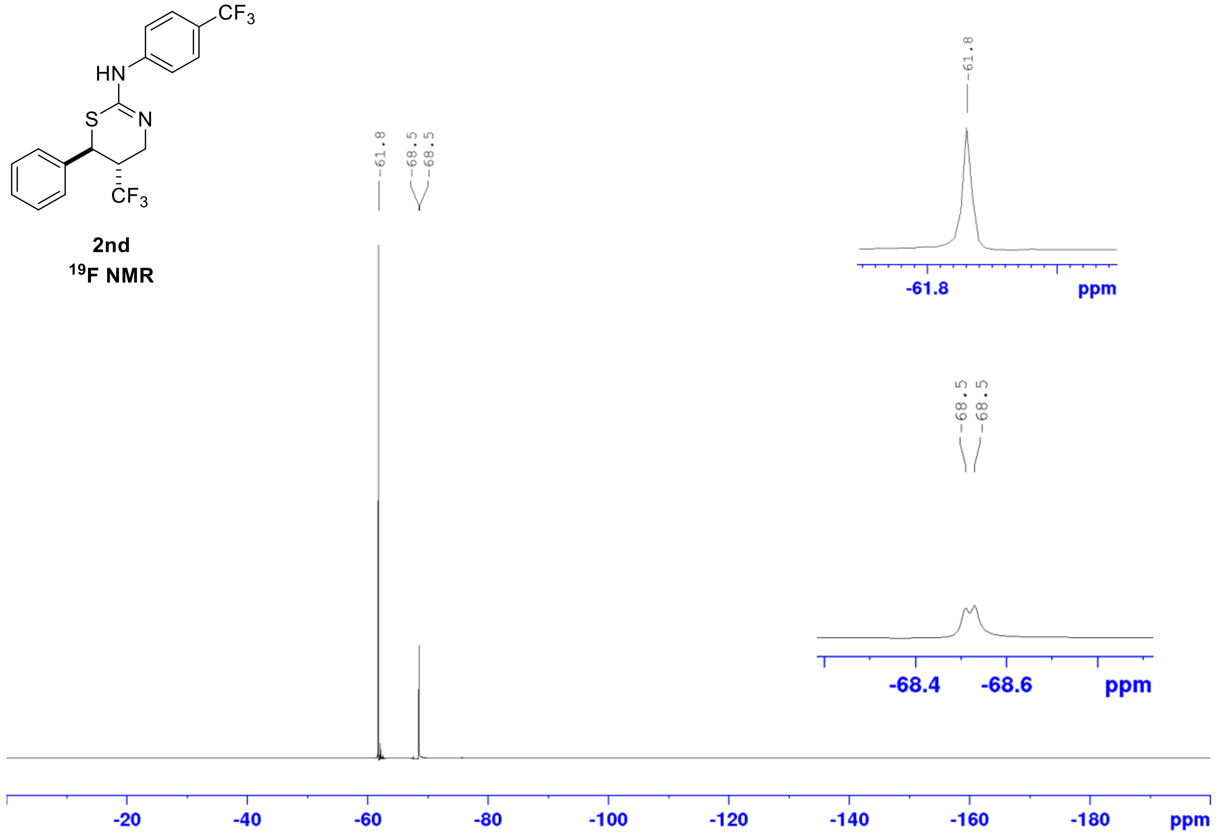

CF3
HN
S
N
CF3
2nd
19F NMR
-61.8
-68.5 -68.5
-61.8
ppm
-68.5 -68.5
-68.4 -68.6 ppm
-20 -40 -60 -80 -100 -120 -140 -160 -180 ppm

(5*R,6*S**)-*N*-(4-Fluorophenyl)-6-phenyl-5-(trifluoromethyl)-5,6-dihydro-4H-1,3-thiazin-2-amine (2ne)**

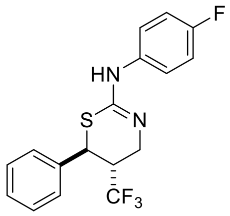

2ne
¹H NMR

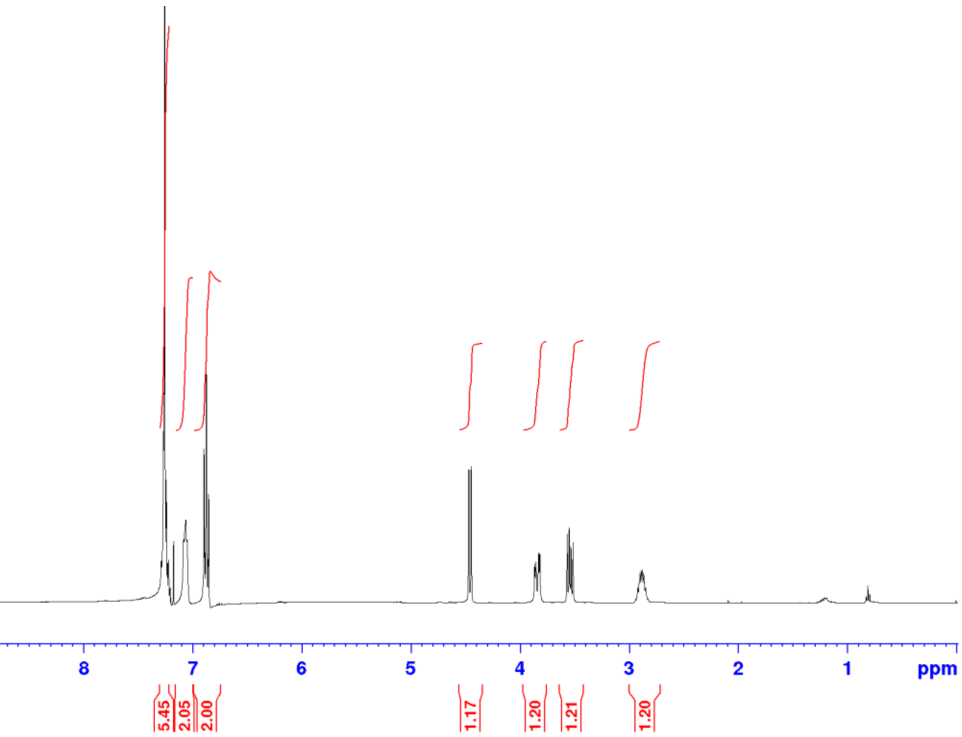

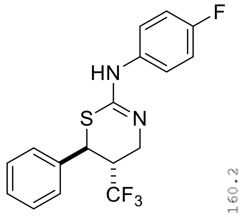

2ne
¹³C NMR

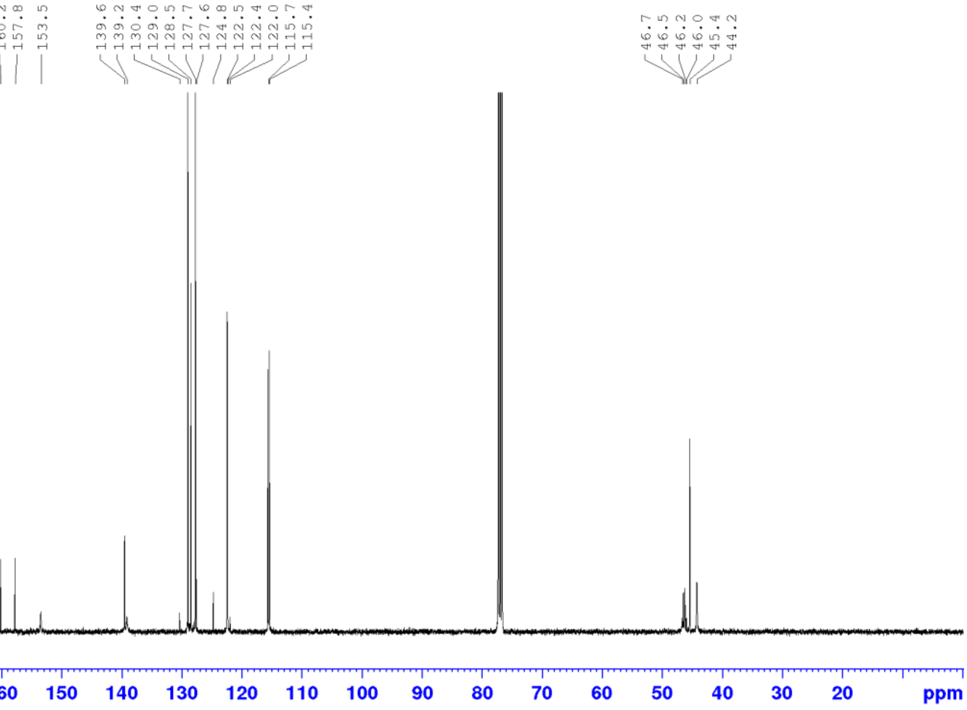

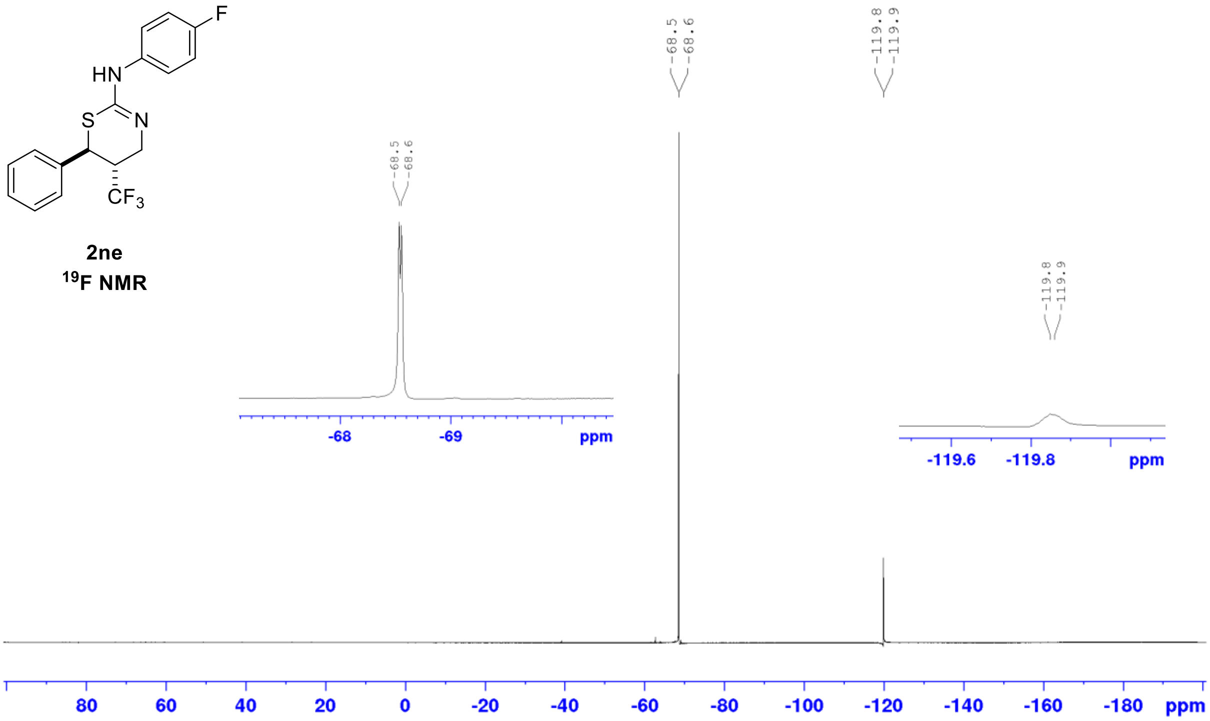

(6*S,7*S**)-*N*-Phenyl-7-(trifluoromethyl)-1-thia-3-azaspiro[5.5]undec-2-en-2-amine (2ra)**

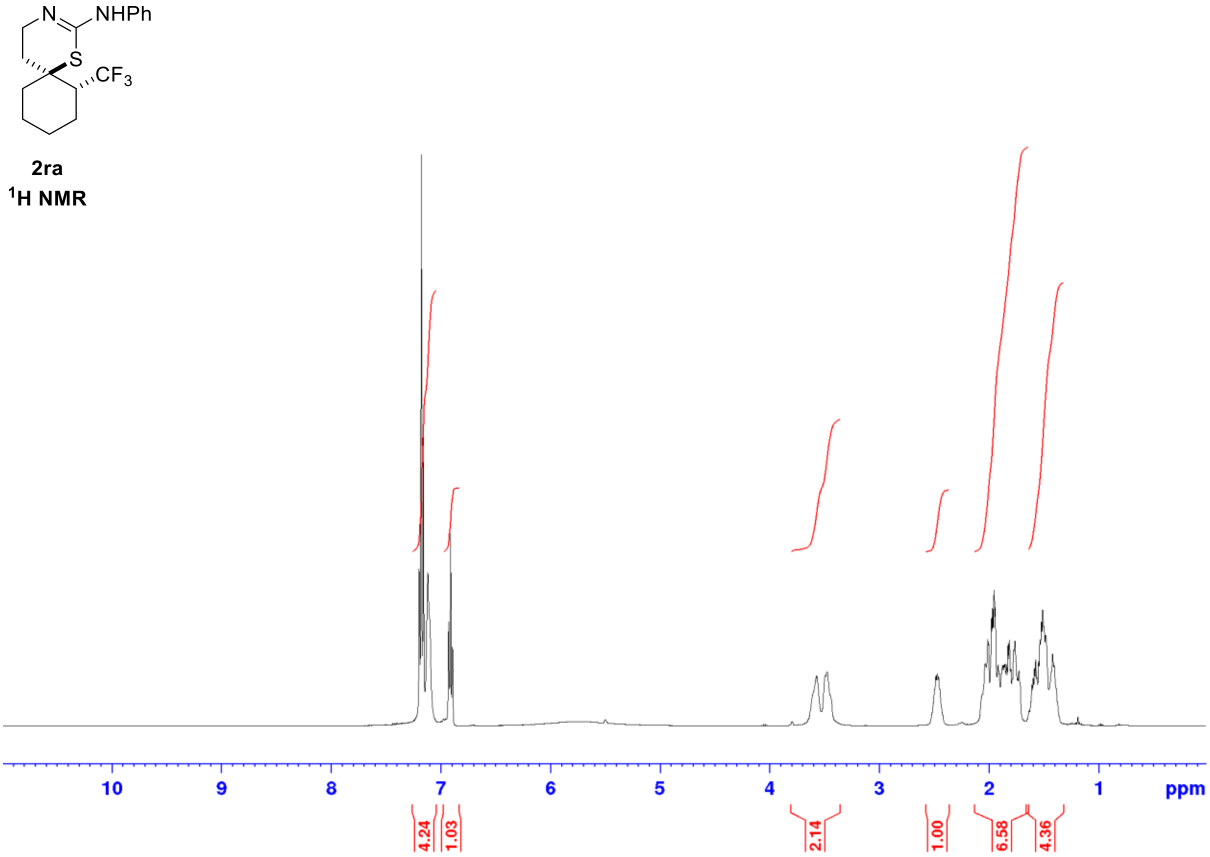

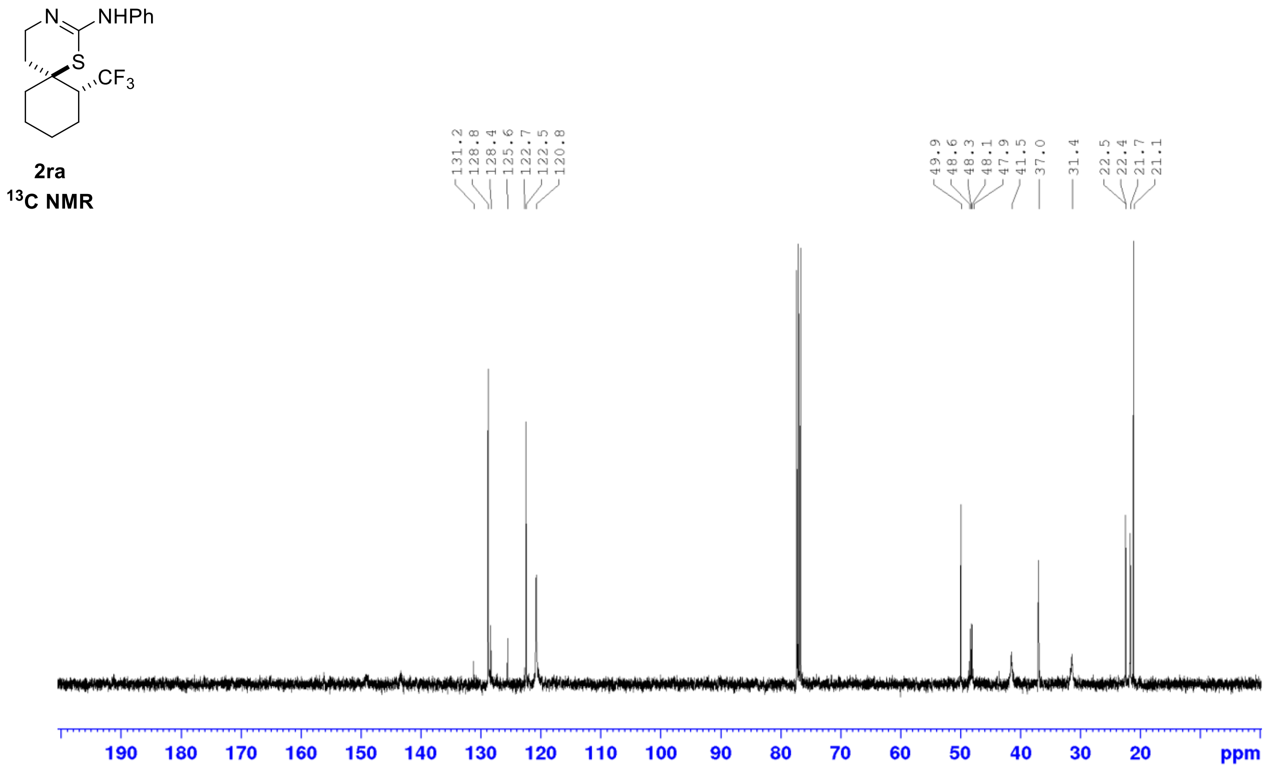

2ra
^{13}C NMR

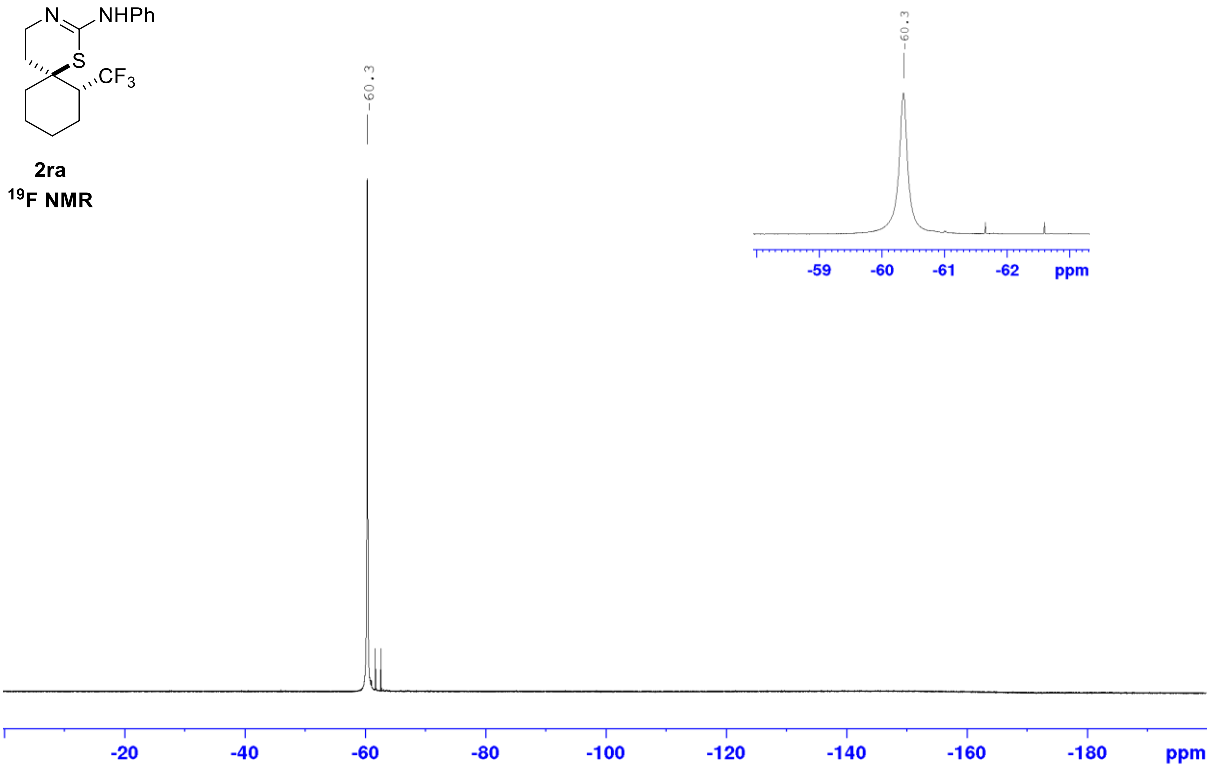

2ra
^{19}F NMR

(6S*,7S*)-N-(4-methoxybenzyl)-7-(trifluoromethyl)-1-thia-3-azaspiro[5.5]undec-2-en-2-amine (2rb)

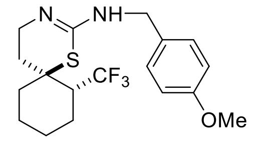

2rb

^{1}H NMR

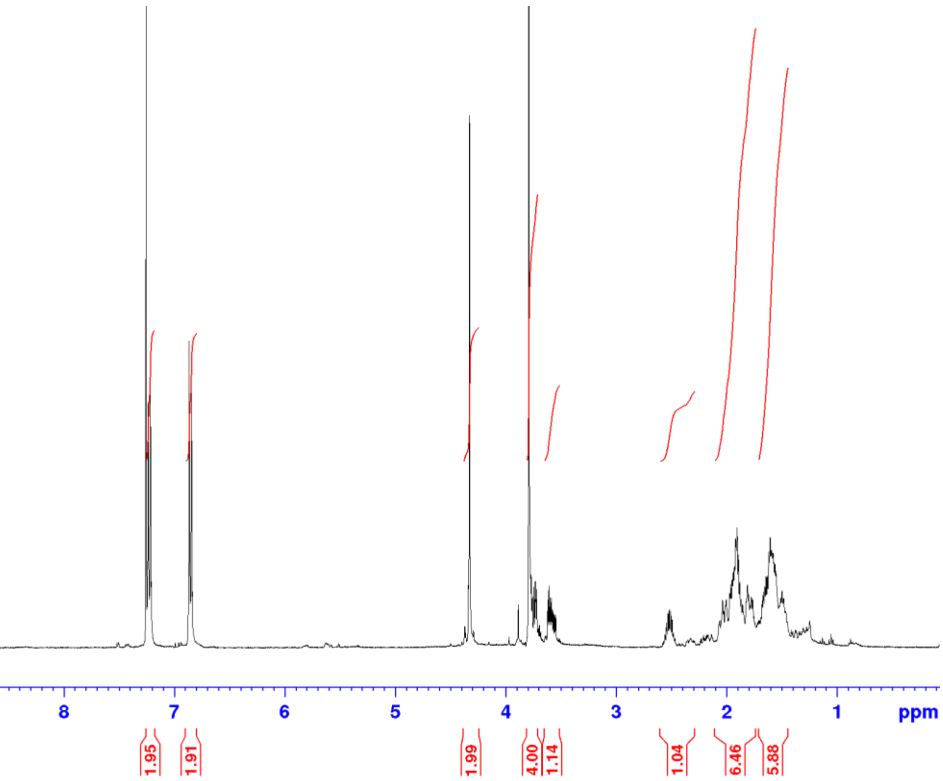

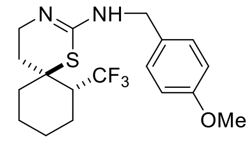

2rb

^{13}C NMR

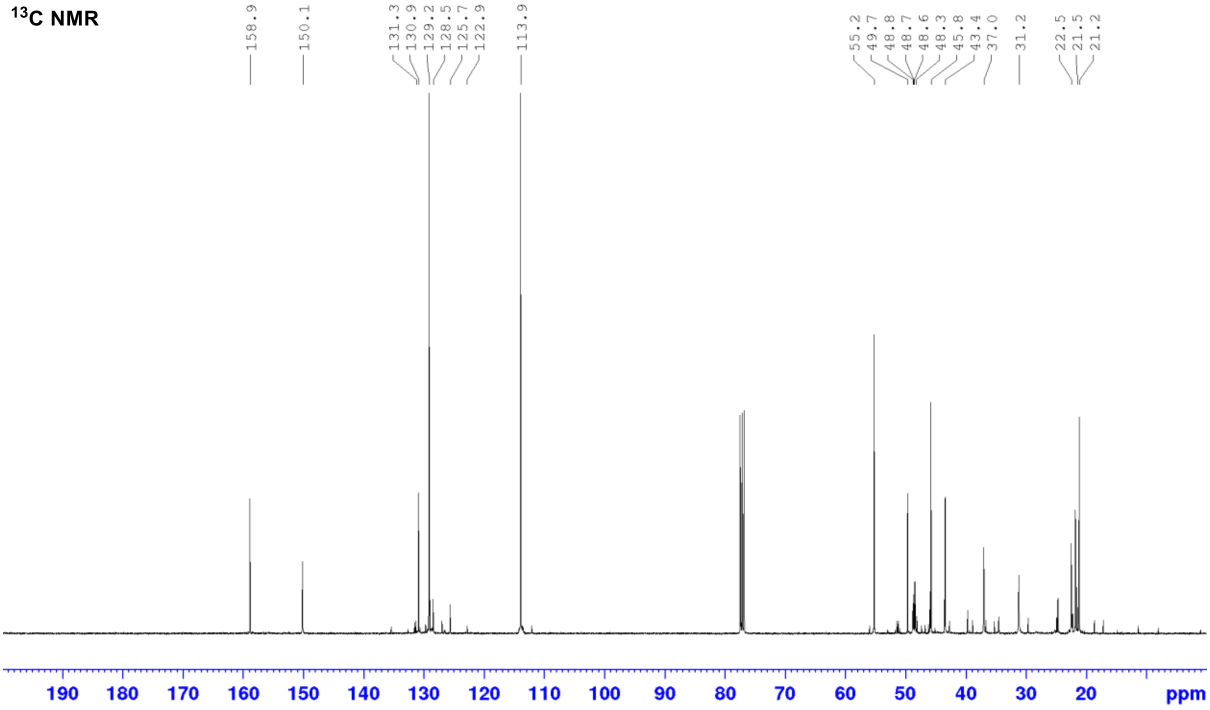

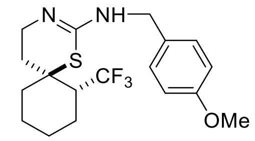

2rb
^{19}F NMR

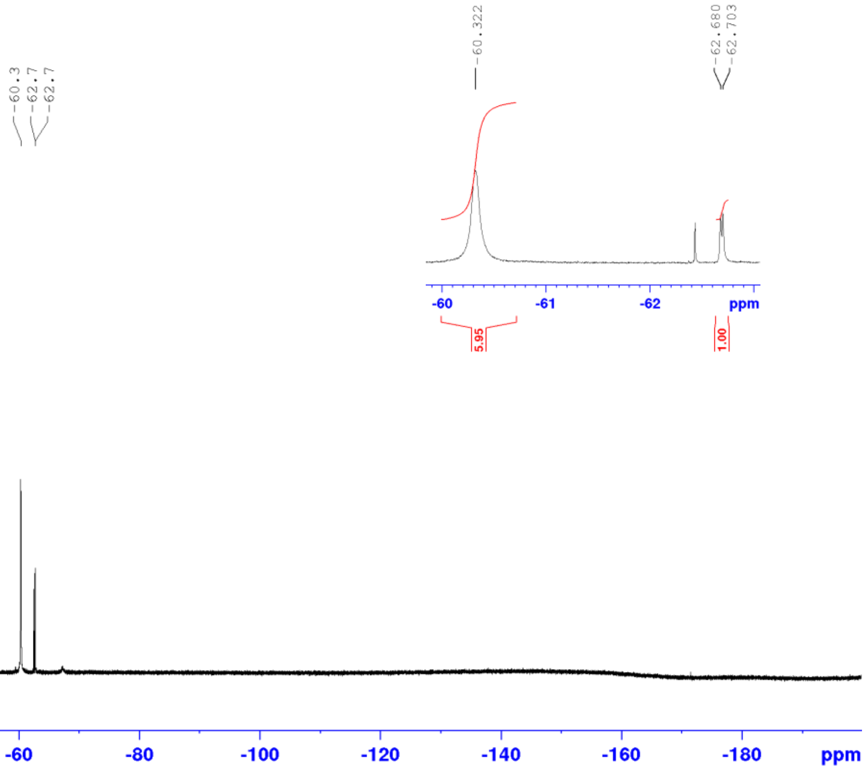

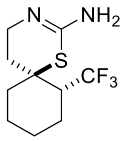

2rc
^{1}H NMR

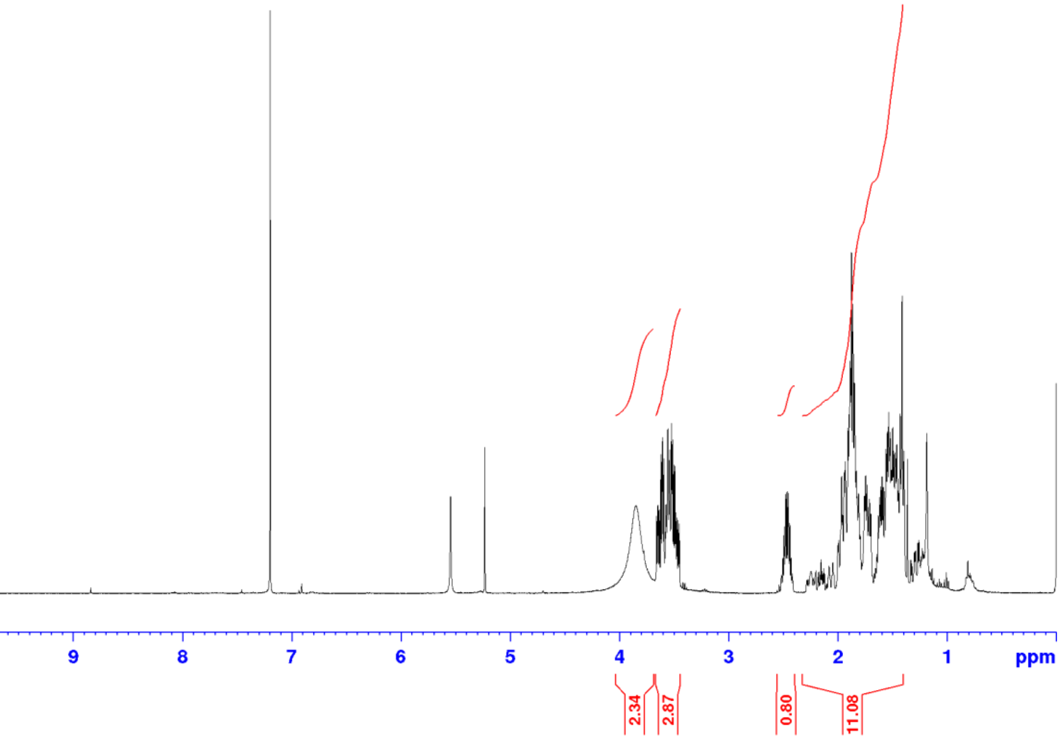

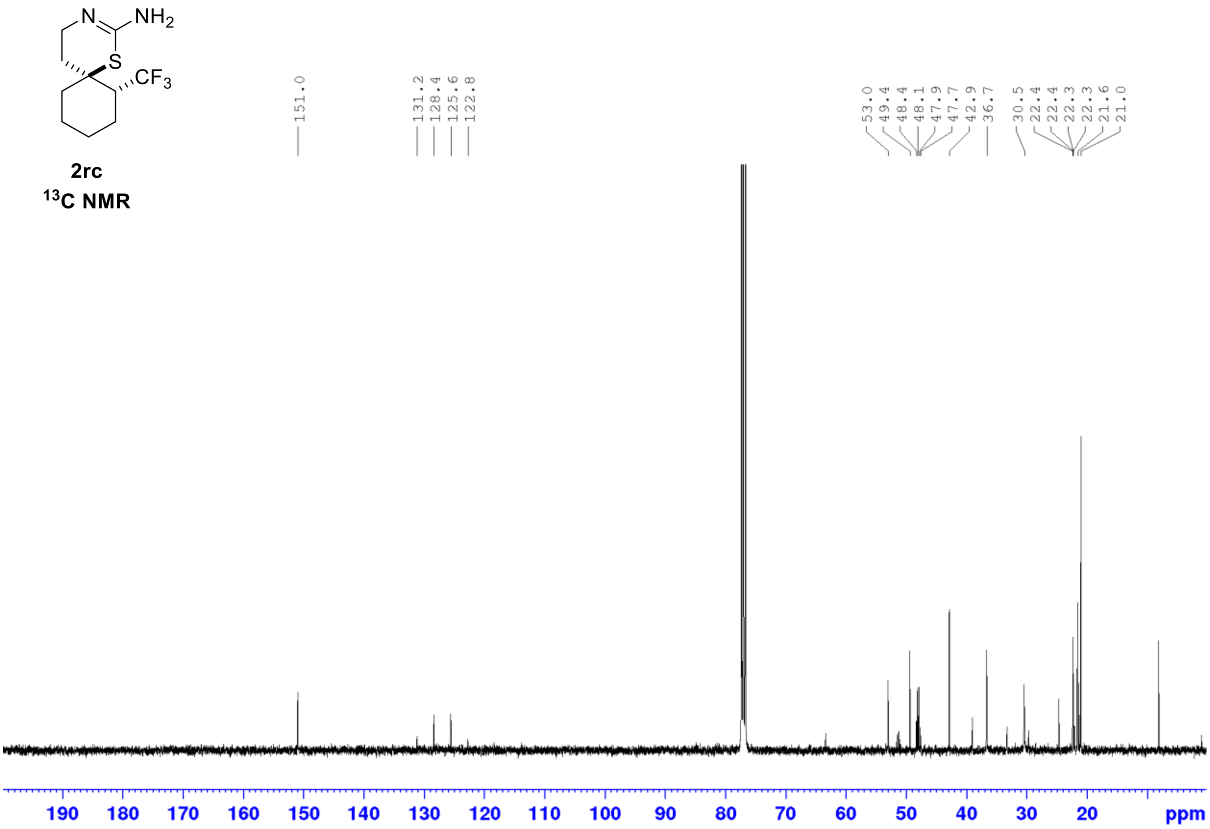

2rc
^{13}C NMR

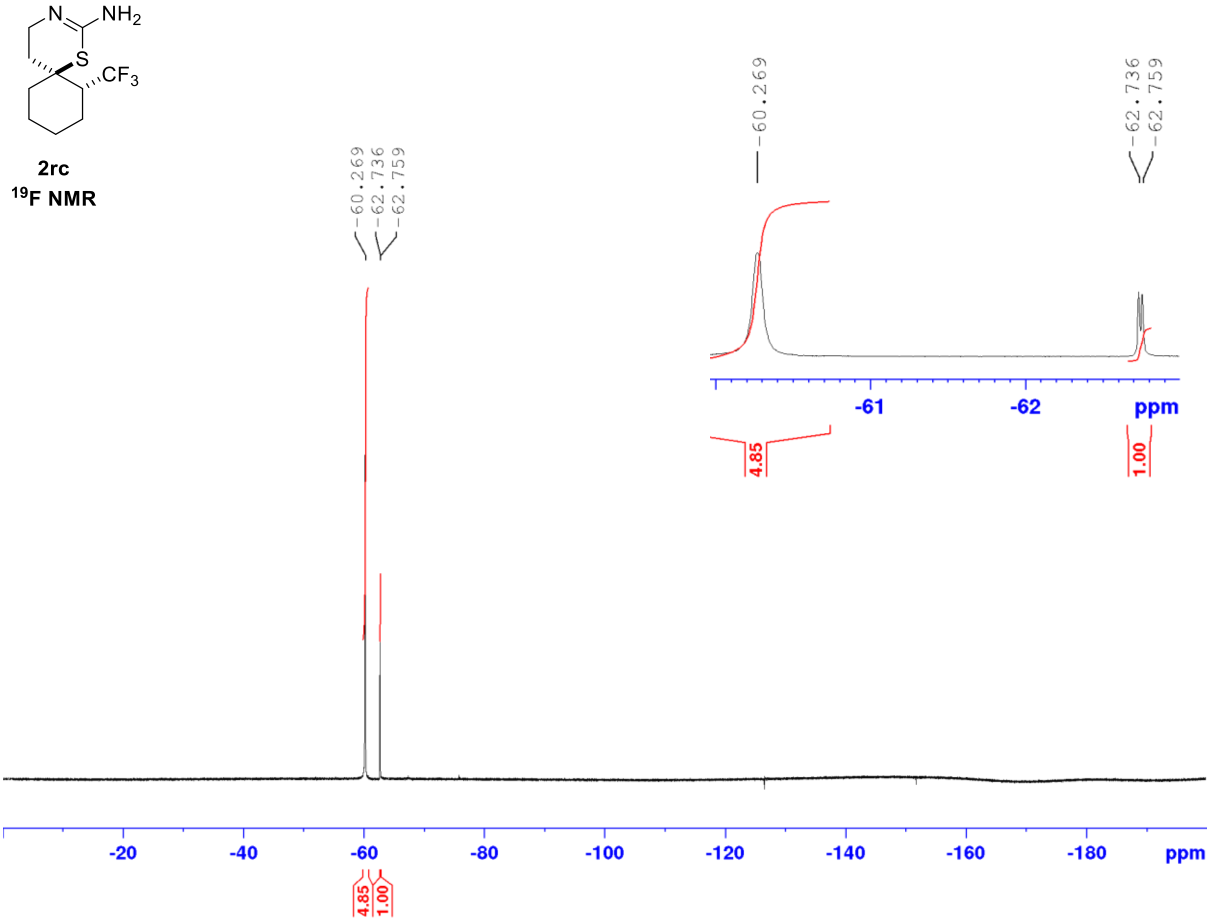

2rc
^{19}F NMR

7-Fluoro-*N*-phenyl-4-(2,2,2-trifluoroethyl)-4,5-dihydrobenzo[d][1,3]thiazepin-2-amine (2sa)

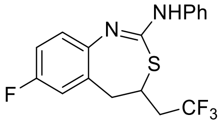

2sa

^{1}H NMR

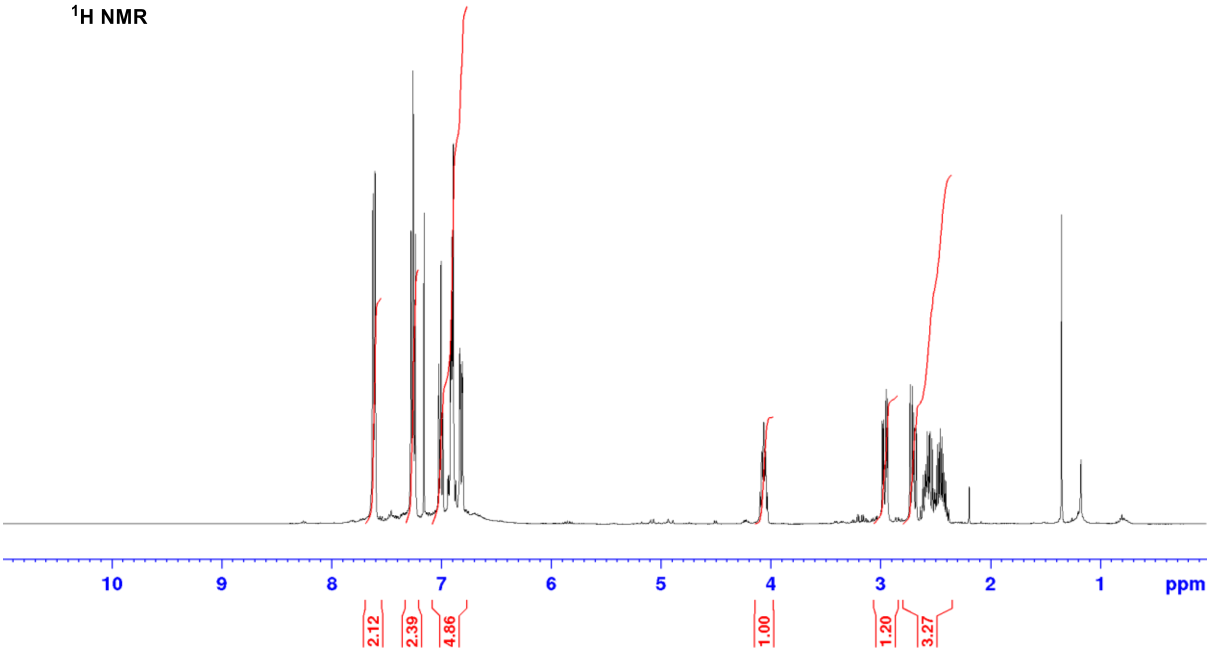

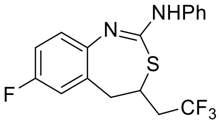

2sa

^{13}C NMR

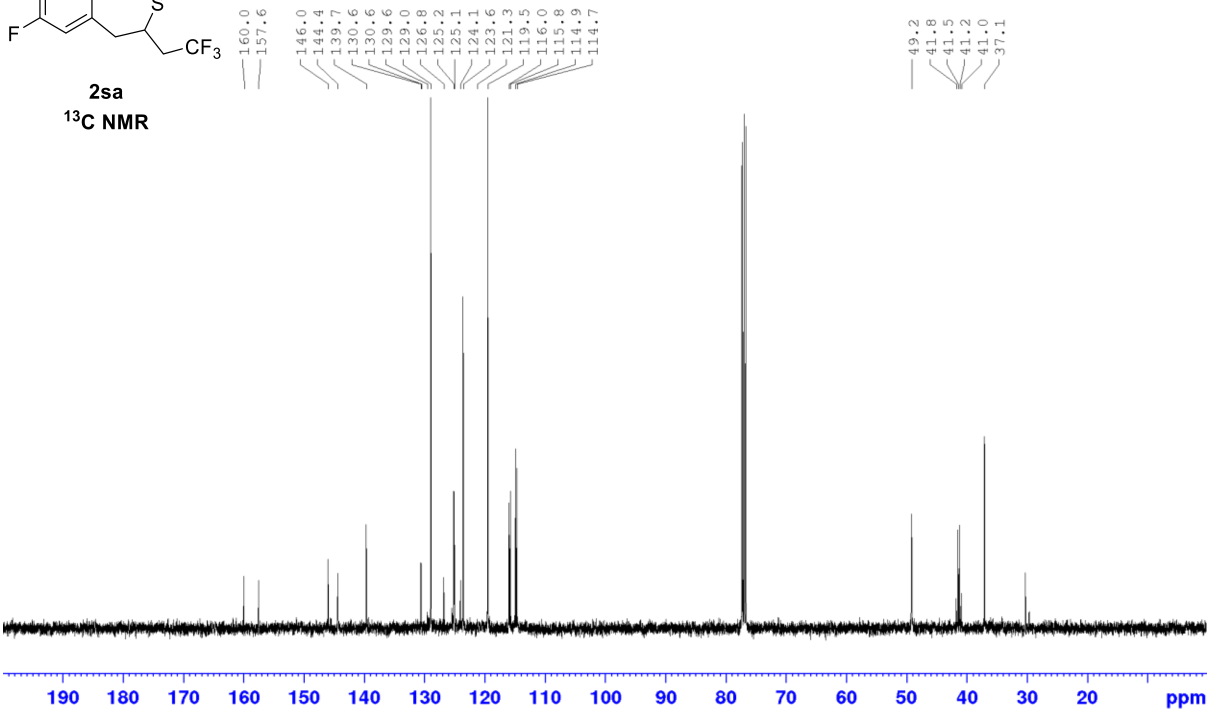

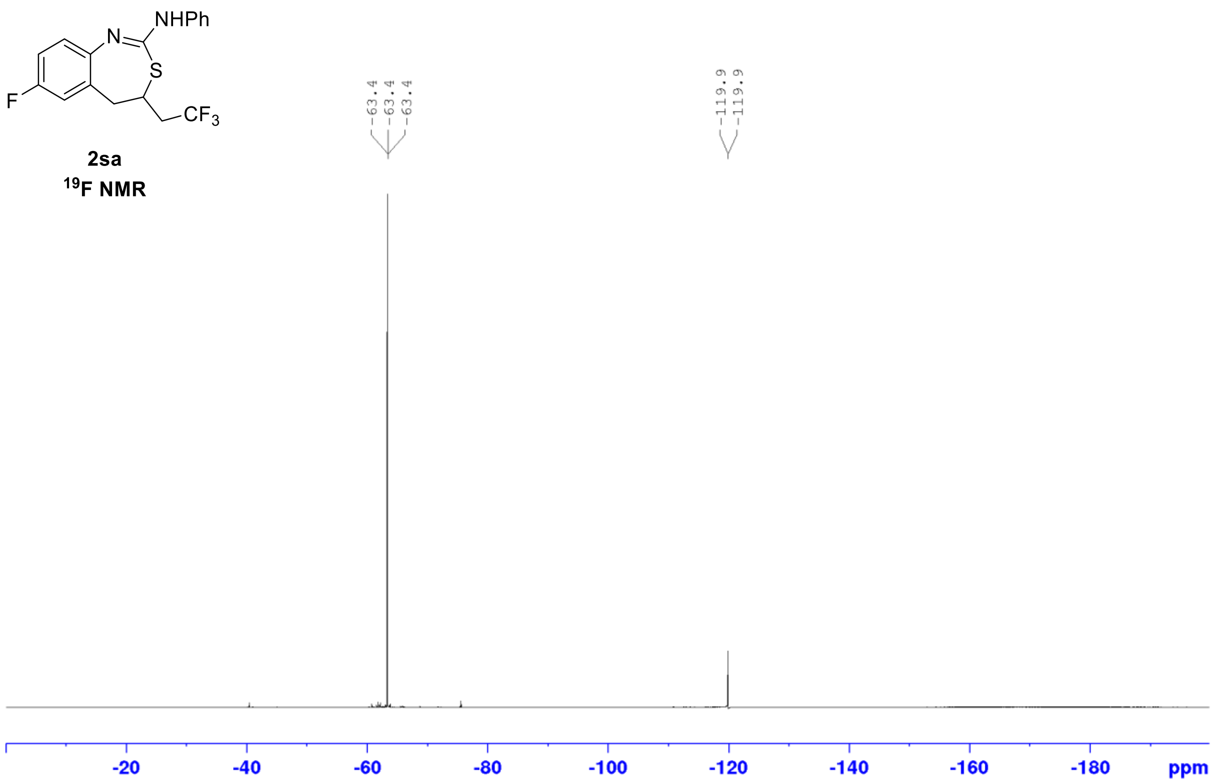

(1*S,2*S**,*Z*)-3'-((4-Nitrophenyl)sulfonyl)-N-phenyl-2-(trifluoromethyl)-3,4-dihydro-2H-spiro[naphthalene-1,5'-thiazolidin]-2'-imine (3)**

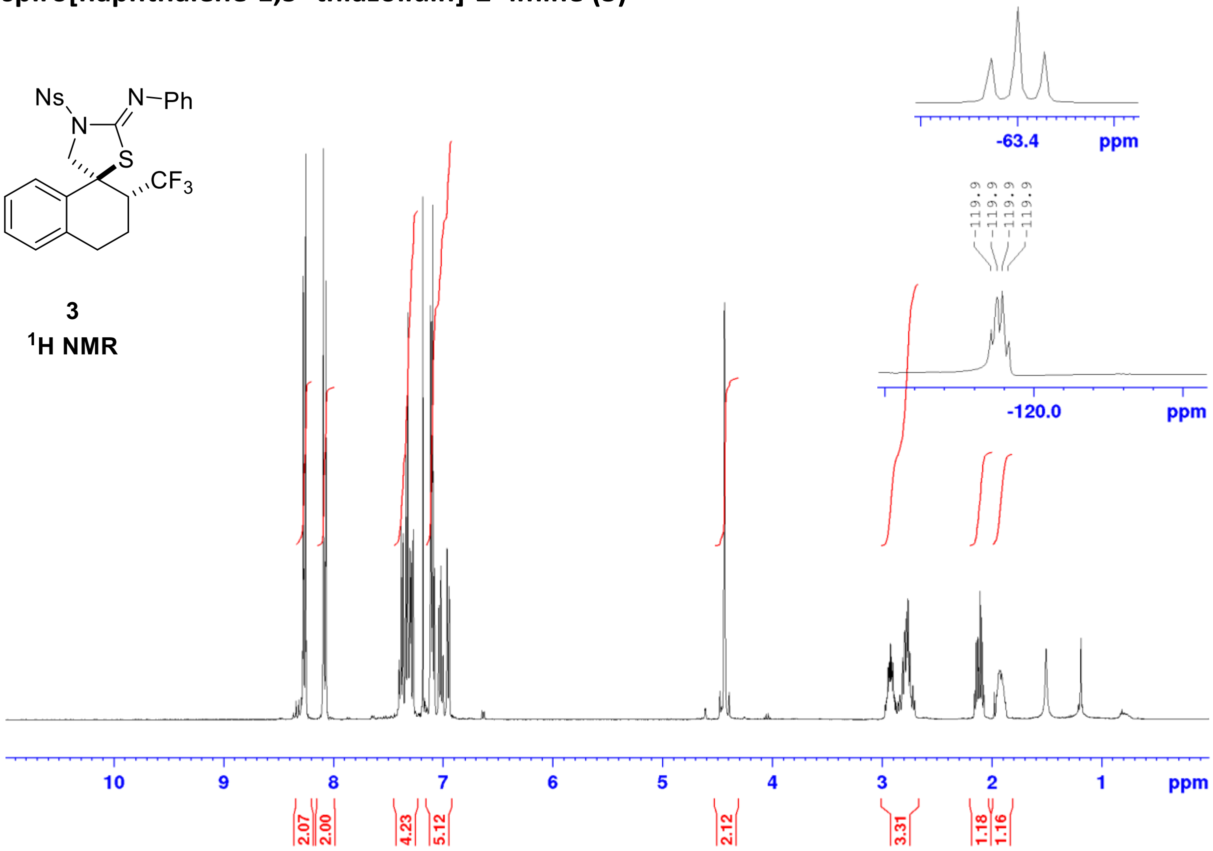

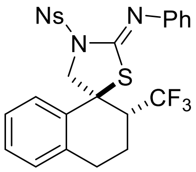

3
^{13}C NMR

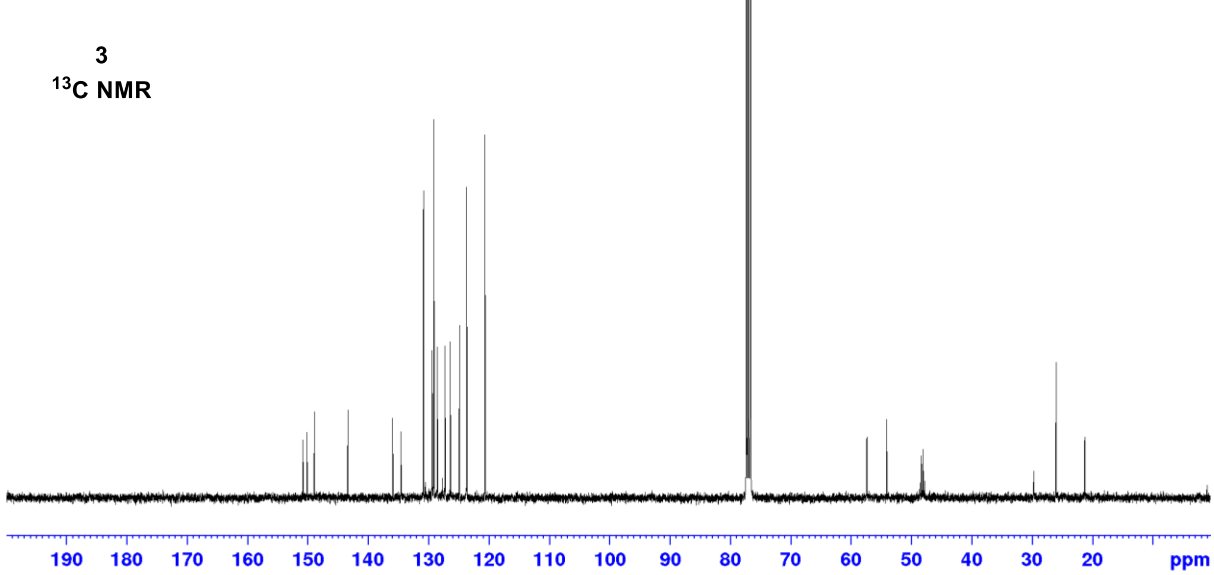

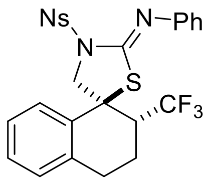

3
^{19}F NMR

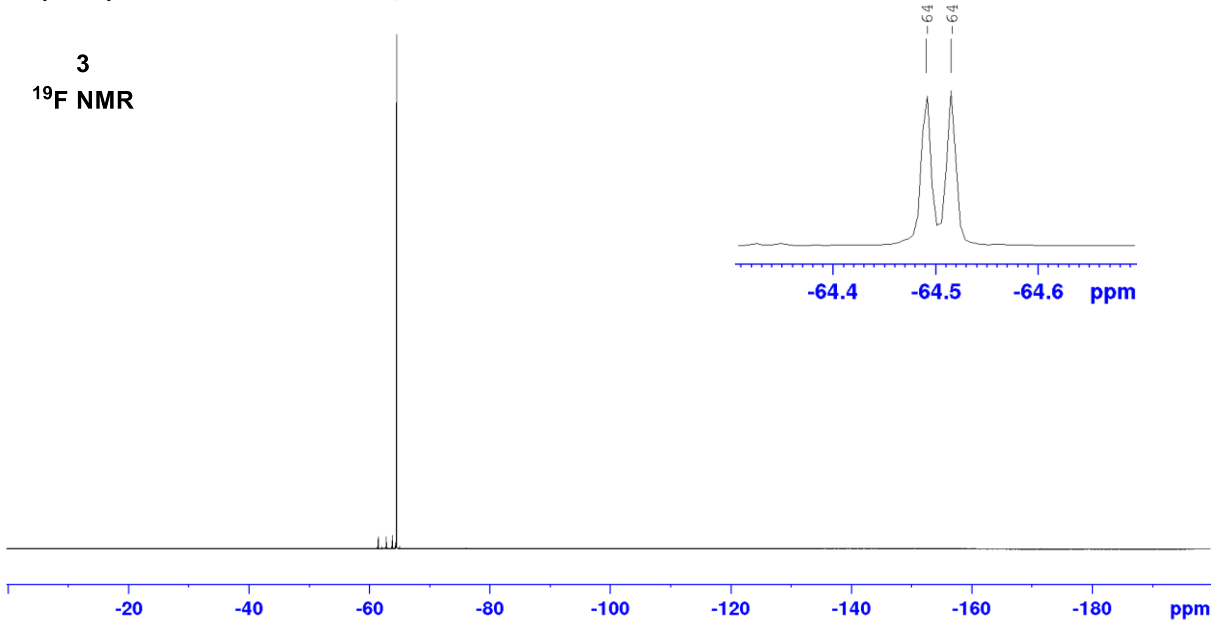

4-Nitro-*N*-phenyl-*N*-((1*S,2*S**)-2-(trifluoromethyl)-3,4,4',5'-tetrahydro-2H-spiro[naphthalene-1,6'-[1,3]thiazin]-2'-yl)benzenesulfonamide (4)**

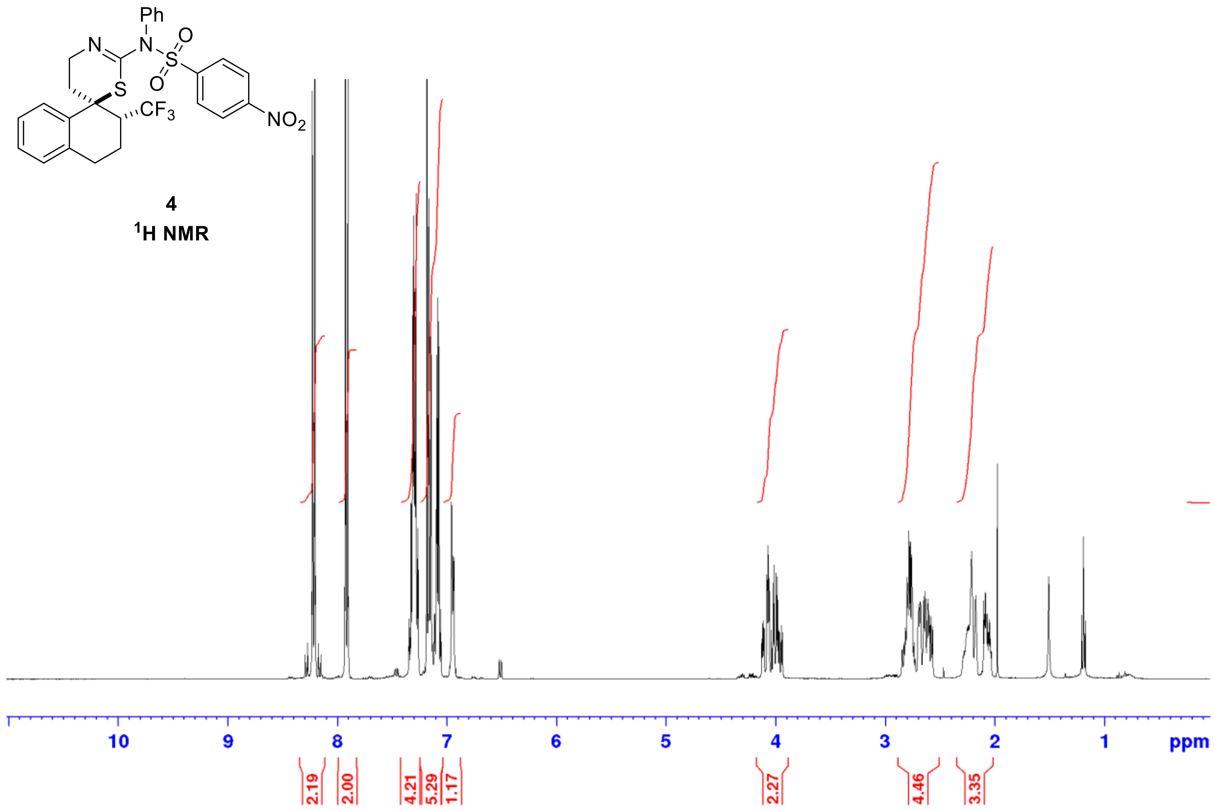

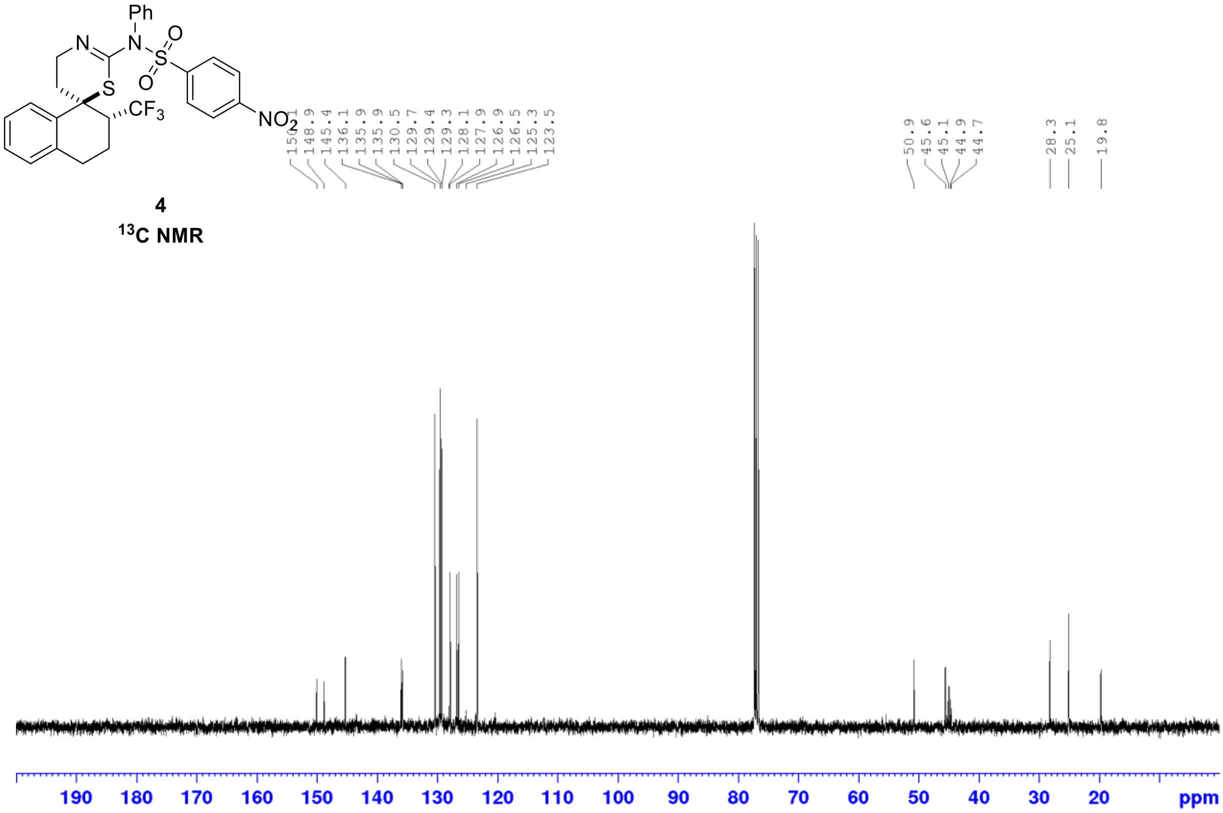

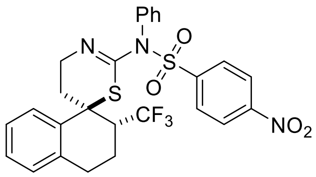

4
^{19}F NMR

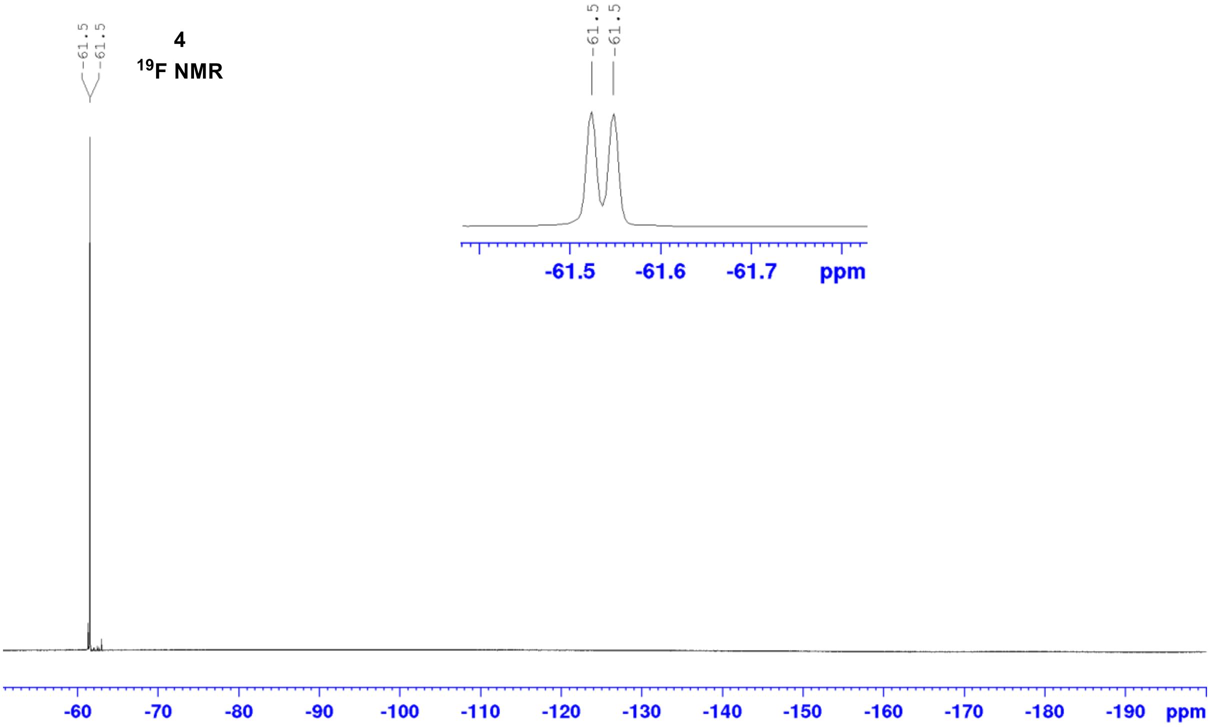

6. Crystallographic Data

6.1. General Information

Low temperature[17] (150 K) single-crystal X-ray diffraction data were collected using or an Oxford Diffraction (Agilent) SuperNova A diffractometer. Raw frame data were reduced using the instrument manufacturer supplied software CrysAlisPro.18 All structures could be solved *ab initio* using SuperFlip,19 and full-matrix least-squares refinement was carried out using CRYSTALS.20,21,22 All non-hydrogen atoms were refined using anisotropic displacement ellipsoids, and hydrogen atoms were visible in the difference map. Once the heavy atoms structure was complete, hydrogen atoms were positioned geometrically then refined separately using soft restraints prior to inclusion in the final refinement using a riding model.23 All structures have been deposited with the Cambridge Crystallographic Data Centre (reference codes CCDC 1474239-1474241); these data can be obtained free of charge via www.ccdc.cam.ac.uk/data_request/cif.

[17] J. Cosier and A. M. Glazer, *J. Appl. Cryst.,* 1986, **19**, 105–107.

[18]CrysAlisPro (Agilent Technologies, Oxford, 2011).

[19]L. Palatinus and G. Chapuis, *J. Appl. Crystallogr.,* 2007, **40**, 786–790.

[20]P. W. Betteridge, J. R. Carruthers, R. I. Cooper, K. Prout and D. J. Watkin, *J. Appl. Crystallogr.,* 2003, **36**, 1487.

[21]P. Parois, R. I. Cooper and A. L. Thompson, *Chem. Cent.*, in press.

[22]A. L. Thompson and D. J. Watkin, *J. Appl. Cryst.,* 2011, **44**, 1017–1022.

[23]R. I. Cooper, A. L. Thompson and D. J. Watkin, *J. Appl. Crystallogr.,* 2010, **43**, 1100–1107.

	**108lp16**	**109lp16**	**107lp16**
**Nr. in Paper**	**3**	**4**	**2oa**
**Name**			
**Formula**	$C_{25}H_{20}F_3N_3O_4S_2$	$C_{26}H_{22}F_3N_3O_4S_2$	$C_{18}H_{17}F_3N_2O_1S_1$
**Molecular Weight**	547.58	561.6	366.41
**Crystal System**	monoclinic	triclinic	monoclinic
**T [K]**	150	150	150
**Space Group**	P 21/n	P -1	P 21/n
**a [Å]**	6.8480(1)	7.4653(2)	15.2695(4)
**b [Å]**	20.4301(3)	9.9343(4)	5.9545(2)
**c [Å]**	17.0570(2)	17.6618(5)	19.0281(7)
**α [°]**	90	89.239(3)	90
**β [°]**	90.3040(11)	85.681(2)	91.540(3)
**γ [°]**	90	72.865(3)	90
**V [Å^3]**	2386.33(6)	1248.09(7)	1729.45(10)
**Z**	4	2	4
**D_{calc} [g cm^{-3}]**	1.524	1.494	1.407
**F(0 0 0)**	1128	580	760
**μ [mm^{-1}]**	2.586	2.487	2.022
**Crystal Size [mm]**	0.10 * 0.10 * 0.25	0.12 * 0.13 * 0.19	0.10 * 0.14 * 0.18
**Colour, Shape**	clear_pale_colourless, prism	clear_pale_colourless, block	clear_pale_colourless, needle
**R_{int}**	3.26	3.63	10.33
**$θ_{min}$, $θ_{max}$ [deg]**	3.375 - 74.753	4.685 - 74.757	3.664-76.067
**Total Reflections**	29782	25529	3587
**Reflections, Restraints, Parameters (I>-3.0/sigma(I))**	4958, 0, 334	5188, 0, 343	3575, 949, 422
**S (=GooF)**	1.000	1.004	0.983
**Min. and Max. Residual Density, [e/Å3]**	-0.39	-0.54	-0.86
	0.28	0.58	1.22
**Threshold Expression**	I>2sigma(I)	I>2sigma(I)	I>2sigma(I)
**R_1**	0.0313	0.0358	0.0976
**wR_2**	0.0812	0.0970	0.2580

(1*S,2*S**,*Z*)-3'-((4-Nitrophenyl)sulfonyl)-N-phenyl-2-(trifluoromethyl)-3,4-dihydro-2H-spiro[naphthalene-1,5'-thiazolidin]-2'-imine (3)**

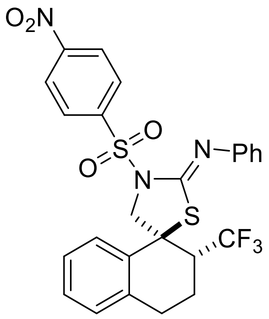

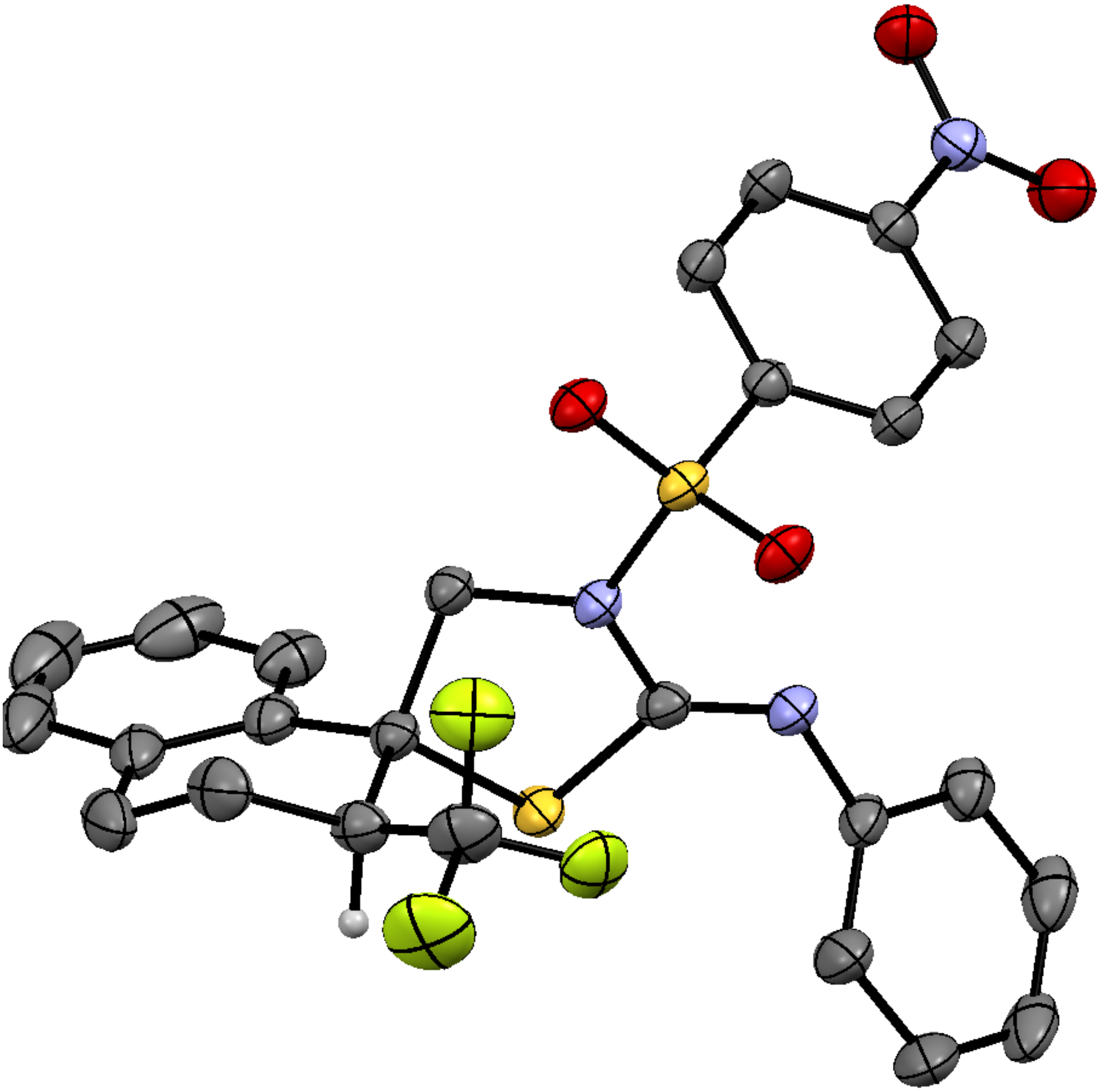

4-Nitro-*N*-phenyl-*N*-((1*S,2*S**)-2-(trifluoromethyl)-3,4,4',5'-tetrahydro-2H-spiro[naphthalene-1,6'-[1,3]thiazin]-2'-yl)benzenesulfonamide (4)**

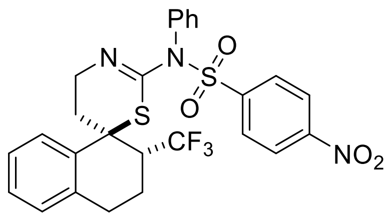

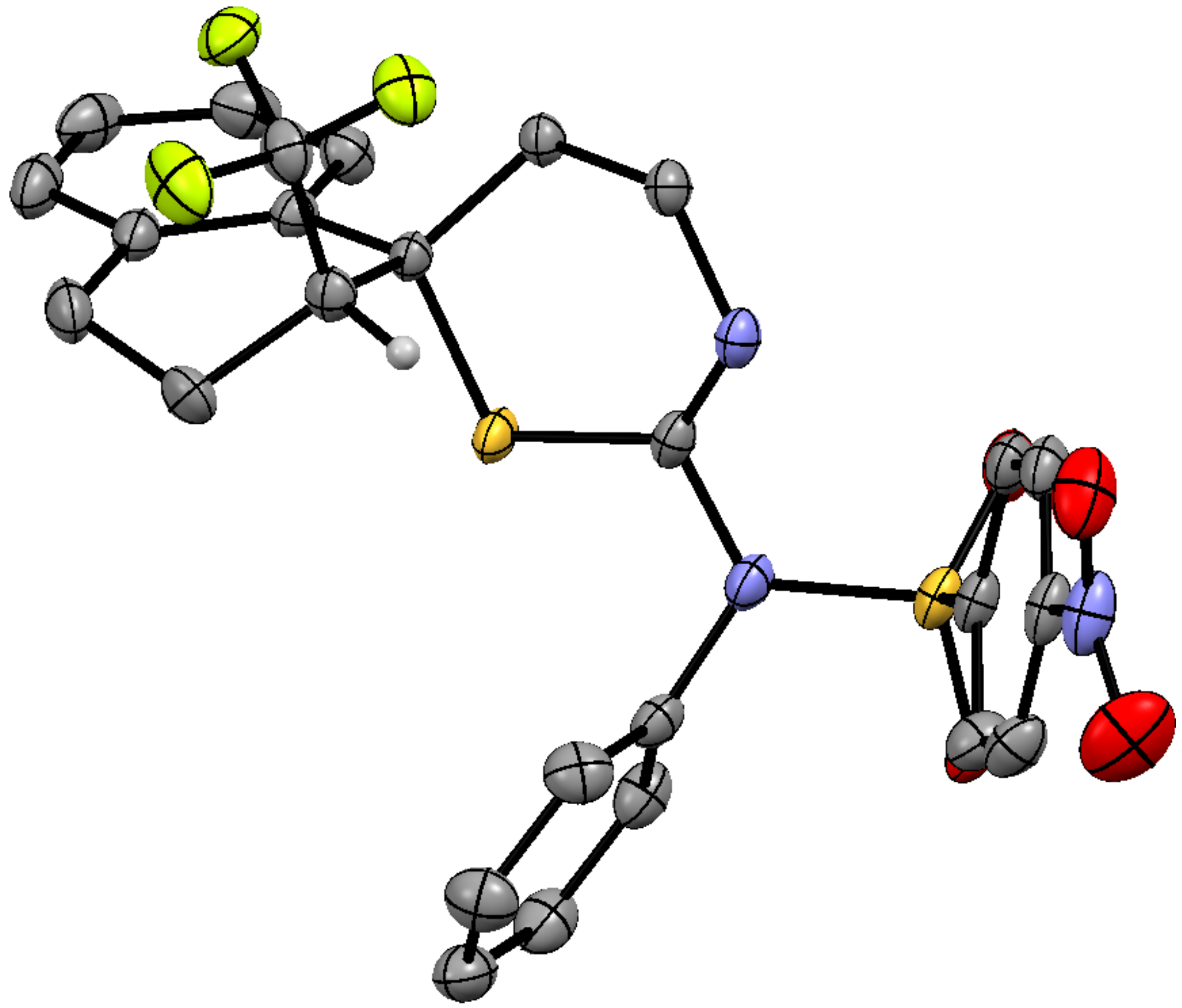

(5*R,6*S**)-6-(4-Methoxyphenyl)-*N*-phenyl-5-(trifluoromethyl)-5,6-dihydro-4H-1,3-thiazin-2-amine (2oa)**

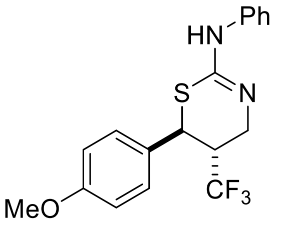

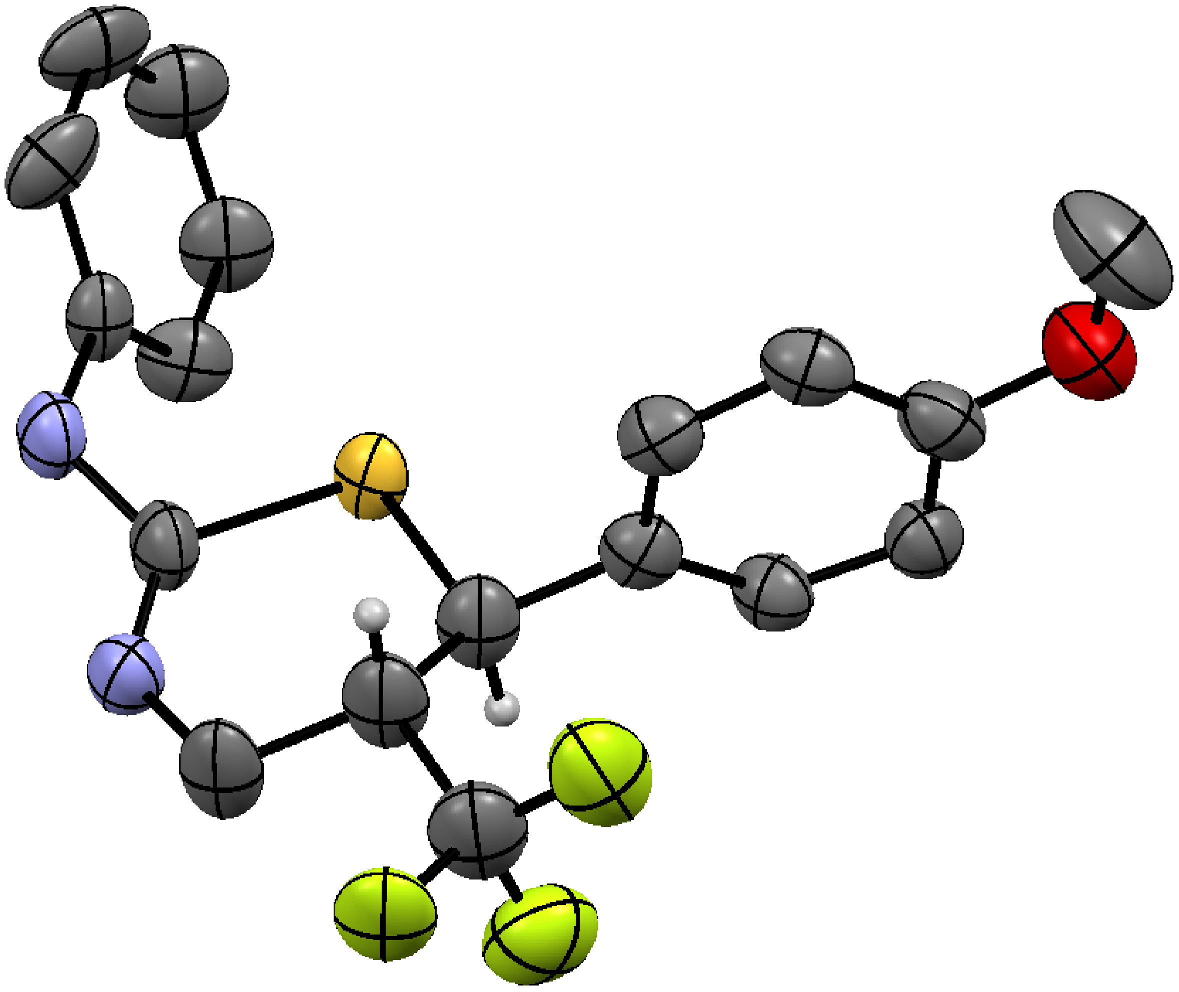

Supplement: Supplementary file 1 [file SC-008-C6SC02790C-s001.pdf]
